# Supplementary material for: Organozinc pivalates for cobalt-catalyzed difluoroalkylarylation of alkenes
Source: Nat Commun. 2021 Jul 16;12:4366. doi: 10.1038/s41467-021-24596-6 (PMC8285467; doi:10.1038/s41467-021-24596-6)
Supplement: Supplementary file 1 — Supplementary Information [file 41467_2021_24596_MOESM1_ESM.pdf]

Supplementary Information

Organozinc Pivalates for Cobalt-Catalyzed Difluoroalkylarylation of Alkenes

Xinyi Cheng,‡[1] Xingcheng Liu,‡[1] Shengchun Wang,‡[2] Ying Hu,[1] Binjing Hu,[1] Aiwen Lei*[2] and Jie Li*[1]

[1] Key Laboratory of Organic Synthesis of Jiangsu Province, College of Chemistry, Chemical Engineering and Materials Science, Soochow University, Ren-Ai Road 199, Suzhou 215123, People's Republic of China

[2] College of Chemistry and Molecular Sciences, the Institute for Advanced Studies (IAS), Wuhan University, Wuhan 430072, People's Republic of China

* E-mail: jjackli@suda.edu.cn; aiwenlei@whu.edu.cn

Contents

General Remarks······S-2

Optimization Studies······S-3

Additional Experiments······S-5

Representative Procedures······S-17

Characterization Data of Products **4** – **85**······S-18

Characterization Data of Products **95**, **97** – **98**······S-59

NMR Spectra······S-70

References······S-199

General Remarks

Catalytic reactions were carried out in Schlenk tubes under an argon atmosphere using pre–dried glassware. Anhydrous acetonitrile (MeCN) was dried and obtained from commercial sources. The following starting materials were synthesized according to previously described methods: vinylarenes **2**,[1] bromodifluoroalkanes **1**.[2] The solid arylzinc pivalates were stored in ampoules filled with inert gas. Other chemicals were obtained from commercial sources and were used without further purification. Yields refer to isolated compounds, estimated to be > 95% pure as determined by ^{1}H-NMR. Chromatography: Merck silica gel 60 (40-63 μm). NMR: Spectra were recorded on Bruker Avance III 400 in the solvent indicated; chemical shifts (δ) are given in ppm. All IR spectra were recorded on a Shimadzu IRTracer-100. High resolution mass spectrometry (HRMS) with Anilent 1200HPLC-6120MS. M. p.: Stuart melting point apparatus SMP3, Barlworld Scientific, values are uncorrected.

Optimization

Supplementary Table 1. Optimization for Cobalt-Catalyzed Difluoroalkylaryla-tion of 4-Methoxystyrene 2a.[a]

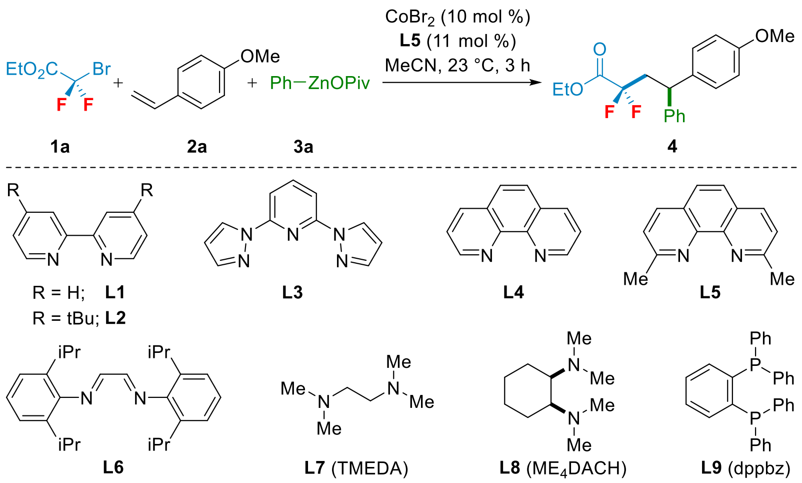

entry	modified conditions	yield (%)[b]
**1**	**no ligand**	**83**
2	**L1** instead of **L5**	trace
3	**L2** instead of **L5**	trace
4	**L3** instead of **L5**	62
5	**L4** instead of **L5**	16
6	***none***	***92***
7	**L6** instead of **L5**	80
8	**L7** instead of **L5**	53
9	**L8** instead of **L5**	51
10	**L9** instead of **L5**	62
11	THF instead of MeCN	12
12	DMF instead of MeCN	trace
13	NMP instead of MeCN	trace
14	PhZnCl instead of PhZnOPiv	42
15	$CoCl_2$ instead of $CoBr_2$	49
16	$CoCl_2(PPh_3)_2$ instead of $CoBr_2$	31
17	$NiBr_2$, $FeCl_2$, $CrCl_2$, or CuBr instead of $CoBr_2$	Trace

[a] Reaction conditions: **1a** (0.50 mmol, 2.0 equiv), **2a** (0.25 mmol, 1.0 equiv), **3a** (0.50 mmol, 2.0 equiv), $CoBr_2$ (10 mol %), **L5** (11 mol %), MeCN (2.0 mL), 23 °C, 3 h. [b] Isolated yields.

Supplementary Table 2. Optimization for Cobalt-Catalyzed Difluoroalkylarylation of unactivated alkene 2r.[a]

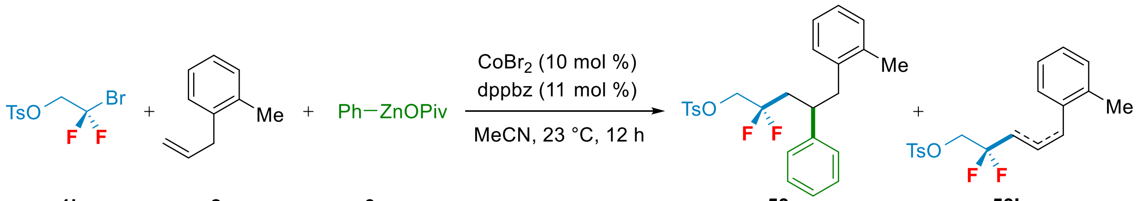

1k **2r** **3a** **53** **53'**

entry	modified conditions	Yield of **53** (%)[b]	Yield of **53'** (%)[c]
1	***none***	***54***	***33***
2	**L3** instead of **L9**	23	12
3	**L4** instead of **L9**	21	11
4	**L6** instead of **L9**	16	7
5	**L7** instead of **L9**	23	9

[a] Reaction conditions: **1k** (0.25 mmol, 1.0 equiv), **2r** (0.5 mmol, 2.0 equiv), **3a** (0.50 mmol, 2.0 equiv), $CoBr_2$ (10 mol %), **L9** (11 mol %), MeCN (2.0 mL), 23 °C, 12 h. [b] Isolated yields. [c] crude NMR yield.

Supplementary Table 3. Optimization for Cobalt-Catalyzed Difluoroalkylarylation of 1,3-Butadiene 2x.[a]

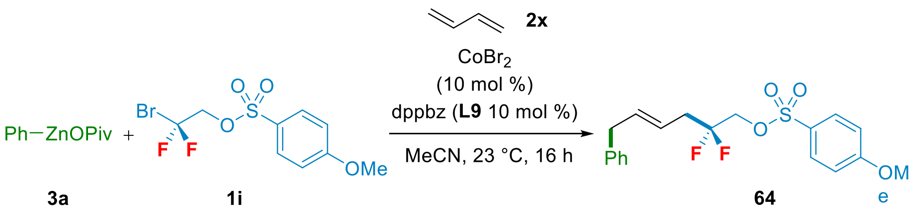

3a **1i** **64**

entry	modified conditions	yield (%)[b]
1	***none***	***80*** *(> 96:4 E:Z, r.r. > 10:1)*
2	**L1** instead of **L9**	<10
3	**L2** instead of **L9**	<10
4	**L3** instead of **L9**	71 (> 96:4 *E:Z*, r.r. > 10:1)
5	**L4** instead of **L9**	12 (> 96:4 *E:Z*, r.r. > 10:1)
6	**L5** instead of **L9**	21 (> 96:4 *E:Z*, r.r. > 10:1)
7	THF instead of MeCN	trace
8	DMF instead of MeCN	trace
9	NMP instead of MeCN	trace

[a] Reaction conditions: **1i** (0.25 mmol, 1.0 equiv), **2x** (0.5 mmol, 2.0 equiv), **3a** (0.50 mmol, 2.0 equiv), $CoBr_2$ (10 mol %), dppbz **L9** (11 mol %), MeCN (1.5 mL), 23 °C, 16 h. [b] Isolated yields.

Additional Expeiments:

a) Kinetic Experiments

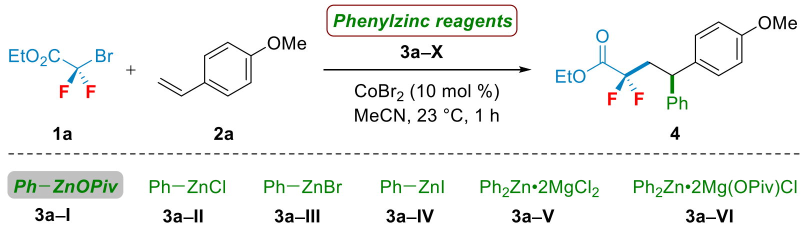

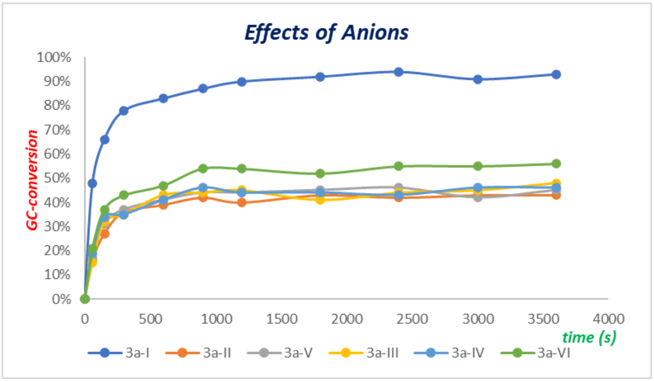

Supplementary Fig. 1 Kientic expeiments with different phenylzinc reagents of 3a–X.

procedures for Fig. S1:

Six parallel reactions with **3a–X** under the standard conditions proceeded: A suspension of $CoBr_2$ (5.4 mg, 10 mol %), 4-methyloxystyrene (34 mg, 0.25 mmol), 4-chlorostyrene (34 mg, 0.25 mmol), bromodifluoroactate (101 mg, 2.0 equiv) and Ar–ZnOPiv (0.5 mmol, 2.0 equiv) in degas MeCN (1.0 mL) was stirred at room temperature for 1 min, 2.5 min, 5 min, 10 min, 15 min, 20 min, 30 min, 40 min, 50 min, 60 min under an atmosphere of argon, respectively. The products **4** was monitored by GC analysis (with 0.25 mmol dodecane as an internal standard). These experiments indicated that the presence of $M(OPiv)_2$ (M = Mg or Zn) has made these new organozinc pivalates stand out amongst salt-supported

organometallics, thus displaying the distinct advantage of reacting well in our regioselective cobalt-catalyzed difluoroalkylarylation of olefins.

b) Mechanistic studies:

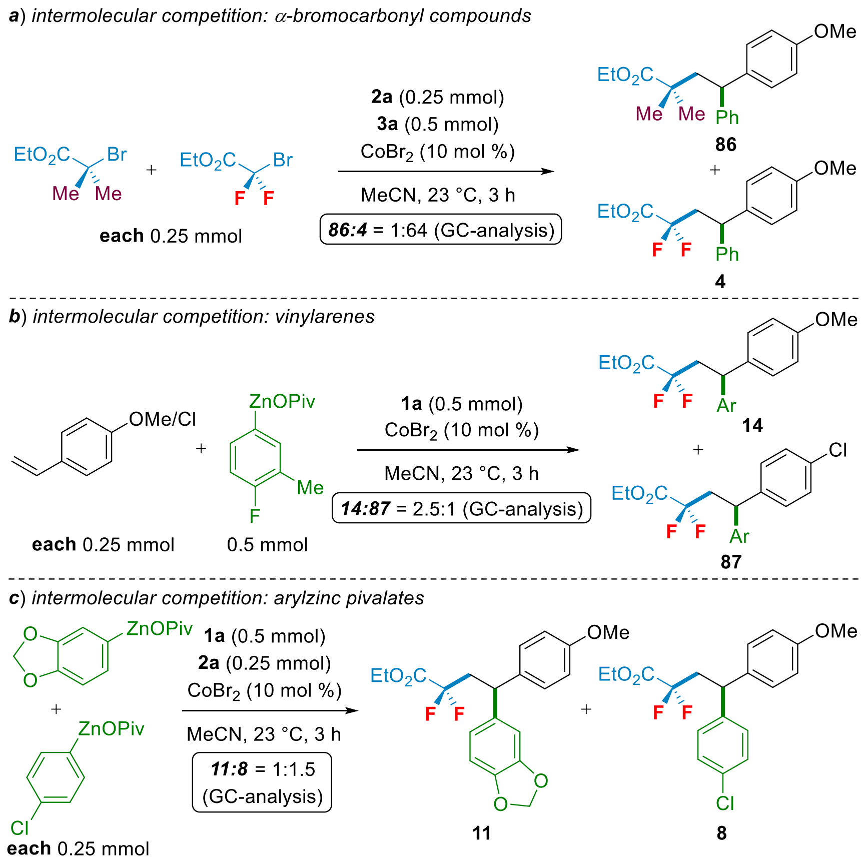

Supplementary Fig. 2 Competition experiments.

procedures for Fig. S2a:

A suspension of $CoBr_2$ (5.4 mg, 10 mol %), 4-methyloxystyrene (34 mg, 0.25 mmol), 4-chlorostyrene (34 mg, 0.25 mmol), bromodifluoroactate (101 mg, 2.0 equiv) and Ar–ZnOPiv (0.5 mmol, 2.0 equiv) in degas MeCN (1.0 mL) was stirred at room temperature for 3 h under an atmosphere of Ar. At ambient temperature, the reaction mixture was analyzed by GC-MS and GC.

These experiments demonstrated that only trace amount of product **86** was detected by GC, while product **4** was observed as the main product in this competition experiment. Importantly, the GC ration between **4** and **86** is 64:1.

procedures for Fig. S2b:

A suspension of $CoBr_2$ (5.4 mg, 10 mol %), 4-methyloxystyrene (34 mg, 0.25 mmol), bromodifluoroactate (101 mg, 2.0 equiv), 2-bromo-2-methylpropanoate (97 mg, 2.0 equiv) and Ph−ZnOPiv (0.5 mmol, 2.0 equiv) in degas MeCN (1.0 mL) was stirred at room temperature for 3 h under an atmosphere of Ar. At ambient temperature, the reaction mixture was analyzed by GC-MS and GC.

Importantly, the GC ration between **14** and **87** is 2.5:1. These experiments demonstrated that vinylarenes might not involve in the rate-determine step.

procedures for Fig. S2c:

A suspension of $CoBr_2$ (5.4 mg, 10 mol %), 4-methyloxystyrene (34 mg, 0.25 mmol), bromodifluoroactate (101 mg, 2.0 equiv), two different aryl zinc pivalates (each 0.5 mmol, 2.0 equiv) in degas MeCN (1.0 mL) was stirred at room temperature for 3 h under an atmosphere of Ar. At ambient temperature, the reaction mixture was analyzed by GC-MS and GC.

Importantly, the GC ration between **11** and **8** is 1:1.5. These experiments demonstrated that aryl zinc pivalates might not involve in the rate-determine step.

c) 4.0 mmol Scale:

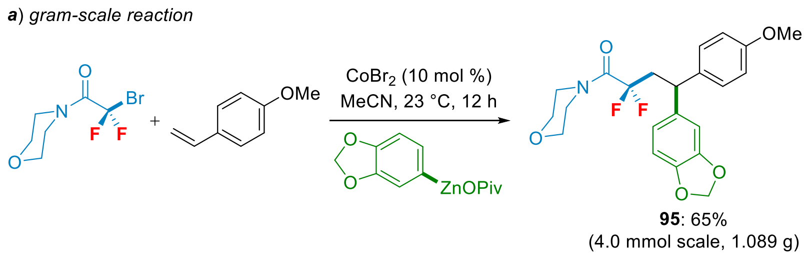

Supplementary Fig. 3 Gram-scale experiment.

procedures for Fig. S3:

A suspension of $CoBr_2$ (87 mg, 10 mol %), 4-methyloxystyrene (536 mg, 4 mmol), bromodifluoroactamide (1.944 g, 2.0 equiv), aryl zinc pivalates (8 mmol, 2.0 equiv) in

degas MeCN (16 mL) was stirred at room temperature for 12 h under an atmosphere of Ar. At ambient temperature, the solvent was evaporated *in vacuo* and the remaining residue was purified by column chromatography on silica gel (*n*-hexane/EtOAc 10:1→5:1) to yield product **95** (1.089 g, 62%).

d) Radical-clock experiments:

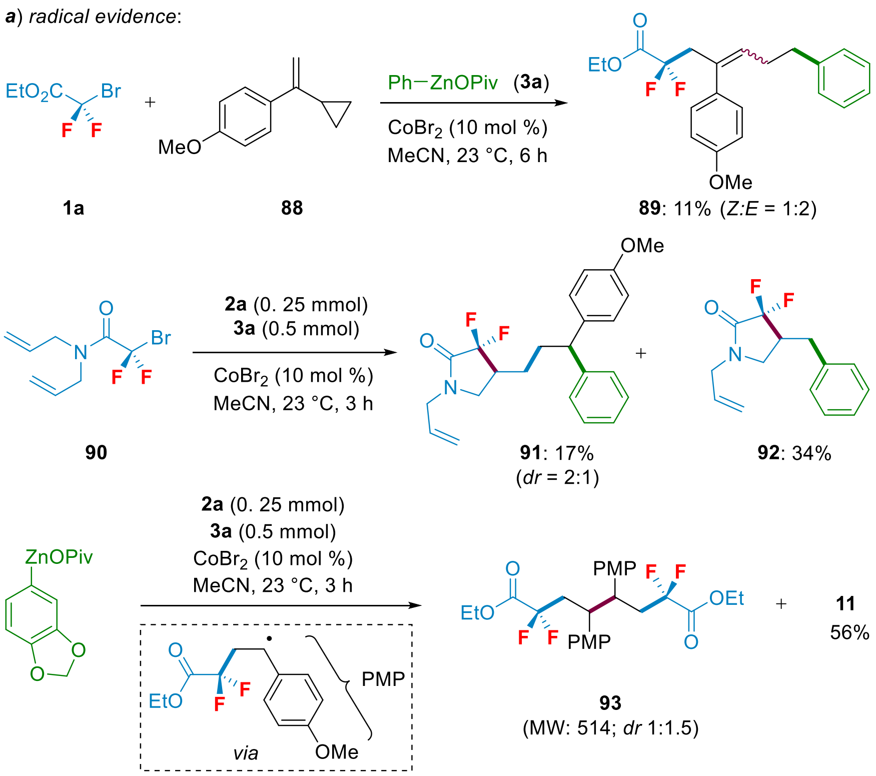

Supplementary Fig. 4 Radical-clock experiments.

procedures for Fig. S4a:

A suspension of $CoBr_2$ (5.4 mg, 10 mol %), vinylarene **88** (44 mg, 0.25 mmol), bromodifluoroacetate (101 mg, 2.0 equiv), Ph−ZnOPiv (0.5 mmol, 2.0 equiv) in degas MeCN (1 mL) was stirred at room temperature for 3 h under an atmosphere of Ar. At ambient temperature, the solvent was evaporated *in vacuo* and the remaining residue was purified by column chromatography on silica gel (*n*-hexane/EtOAc 20:1) to yield product **89** (11 mg, 11%).

a) *radical evidence*:

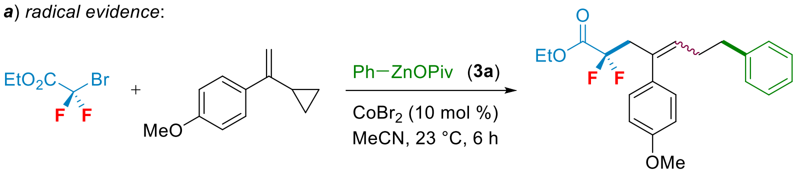

1a **88** **89**: 11% (*Z*:*E* = 1:2)

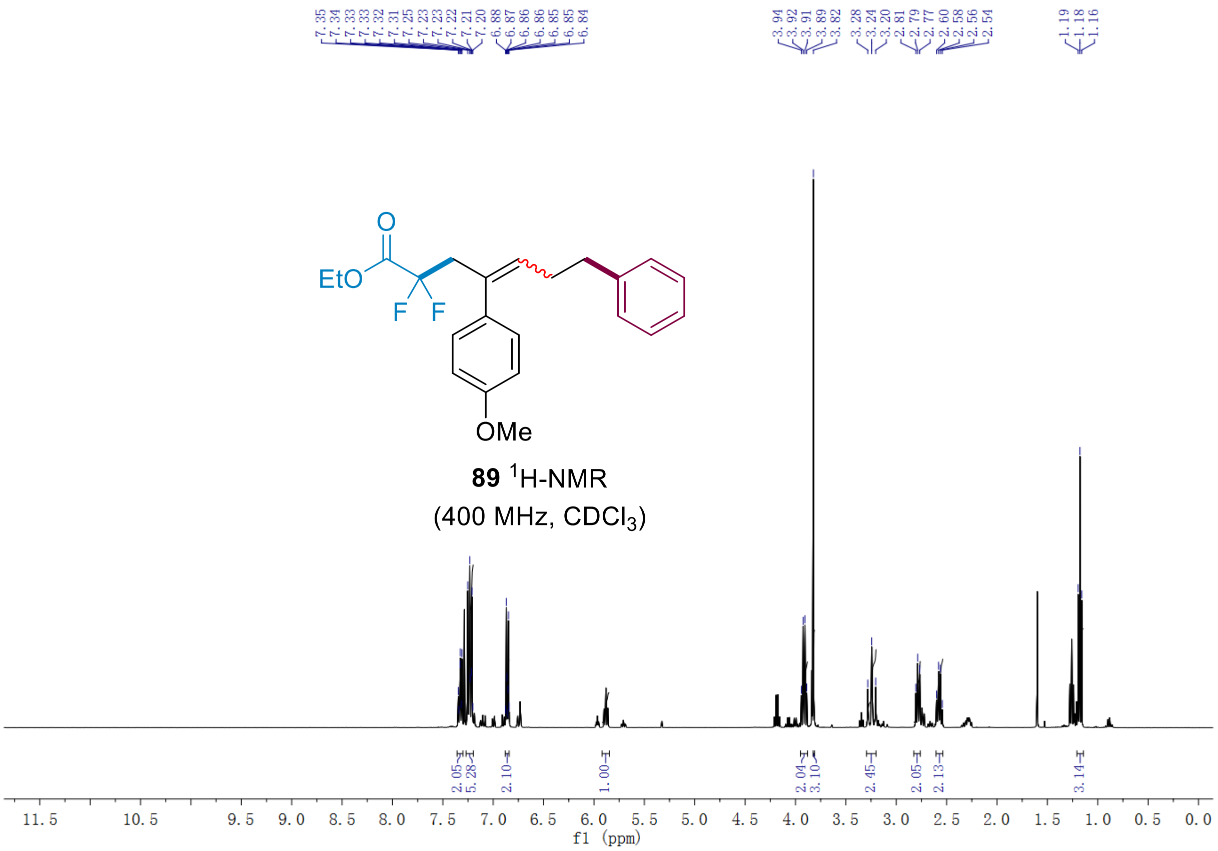

procedures for Fig. S4b:

A suspension of $CoBr_2$ (5.4 mg, 10 mol %), 4-methyloxystyrene (34 mg, 0.25 mmol), bromodifluoroacetamide (127 mg, 2.0 equiv), Ph−ZnOPiv (0.5 mmol, 2.0 equiv) in degas MeCN (1 mL) was stirred at room temperature for 3 h under an atmosphere of Ar. At ambient temperature, the solvent was evaporated *in vacuo* and the remaining residue was purified by column chromatography on silica gel (*n*-hexane/EtOAc 20:1→10:1) to yield product **92** (16 mg, 17%) and product **93** (21 mg, 34%), respectively.

b) *radical evidence*:

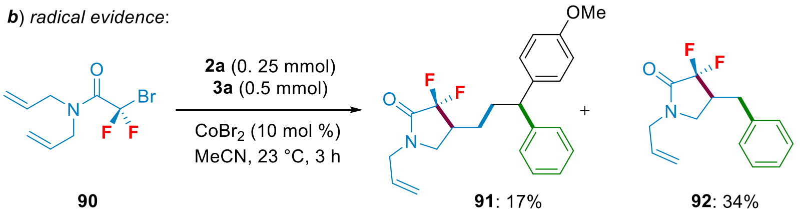

90 **91**: 17% (*dr* = 2:1) **92**: 34%

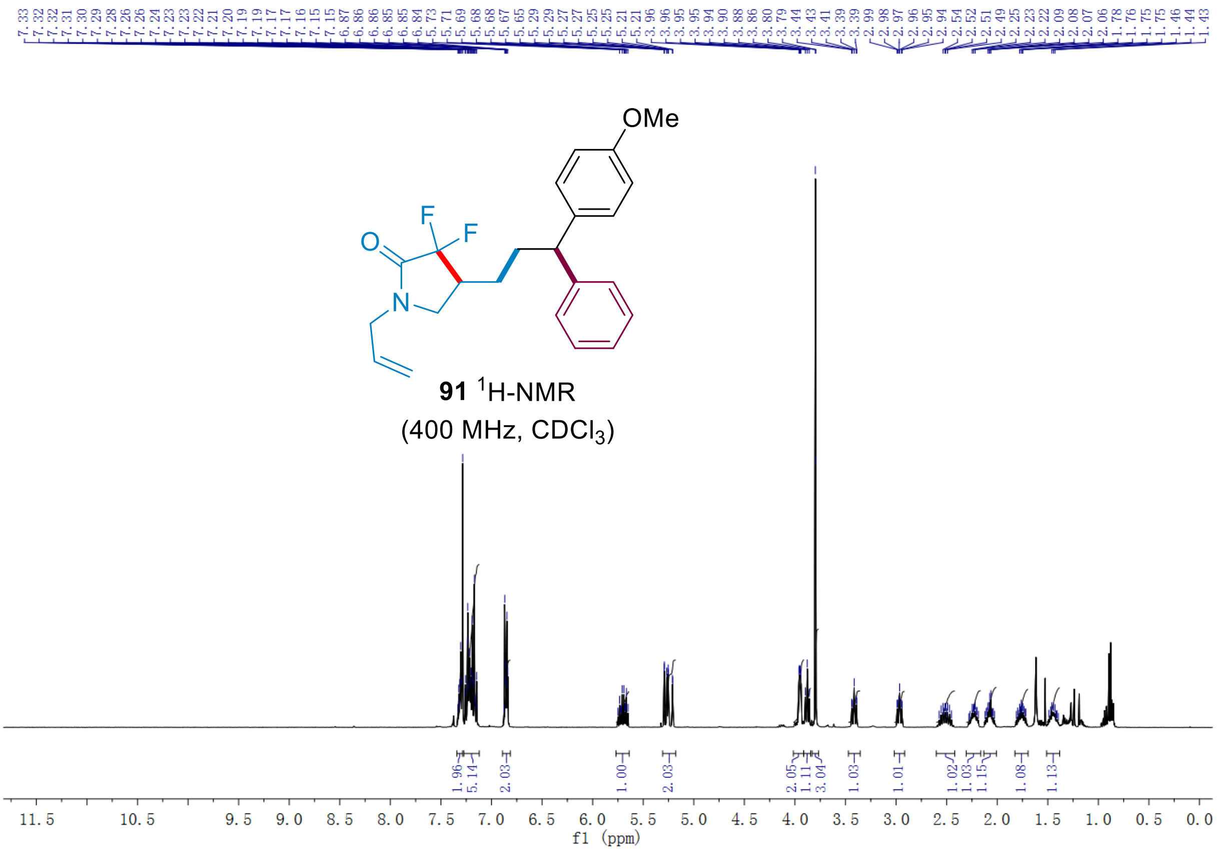

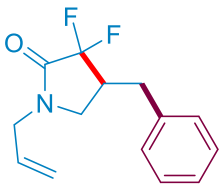

1-Allyl-4-benzyl-3,3-difluoropyrrolidin-2-one (92)

^{1}H-NMR ($CDCl_3$, 400 MHz): δ = 7.36 (ddd, J = 7.5, 4.4, 1.3 Hz, 2H), 7.31 – 7.26 (m, 1H), 7.26 – 7.19 (m, 2H), 5.70 (ddt, J = 16.4, 10.2, 6.2 Hz, 1H), 5.30 – 5.18 (m, 2H), 4.02 – 3.88 (m, 2H), 3.31 – 3.17 (m, 2H), 3.12 (ddd, J = 9.8, 4.9, 1.7 Hz, 1H), 2.93 – 2.76 (m, 1H), 2.76 – 2.67 (m, 1H). ^{13}C-NMR ($CDCl_3$, 100 MHz): δ = 163.4 (t, $J_{C\text{-}F}$ = 31.0 Hz), 137.1, 130.5, 128.9, 128.7, 127.0, 119.7, 116.5 (d, $J_{C\text{-}F}$ = 251.0 Hz), 46.6 (d, $J_{C\text{-}F}$ = 6.5 Hz), 45.9, 41.8 (t, $J_{C\text{-}F}$ = 21.1 Hz), 31.6 (d, $J_{C\text{-}F}$ = 7.7 Hz). ^{19}F-NMR ($CDCl_3$, 376 MHz): δ = -110.31 (dd, J = 14.7, 2.0 Hz), -111.02 (dd, J = 14.4, 1.9 Hz), -117.06 (d, J = 16.9 Hz), -117.77 (d, J = 16.6 Hz). IR (ATR): 2924, 1665, 1457, 1273, 1162 cm^{-1}. HR-MS (ESI) m/z calcd for $C_{14}H_{16}F_2NO$ $[M+H^+]$ 252.1200, found 252.1205 $[M+H^+]$.

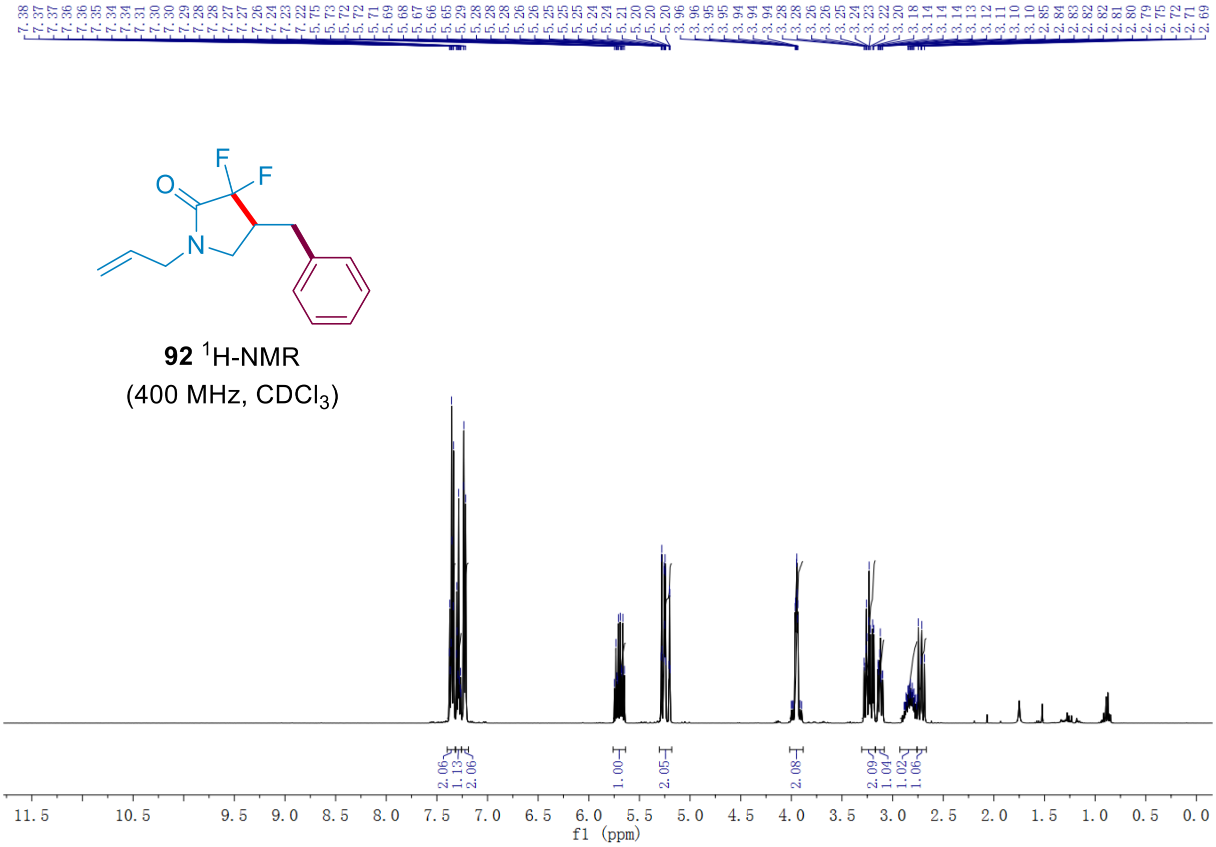

92 1H-NMR
(400 MHz, CDCl3)
f1 (ppm)

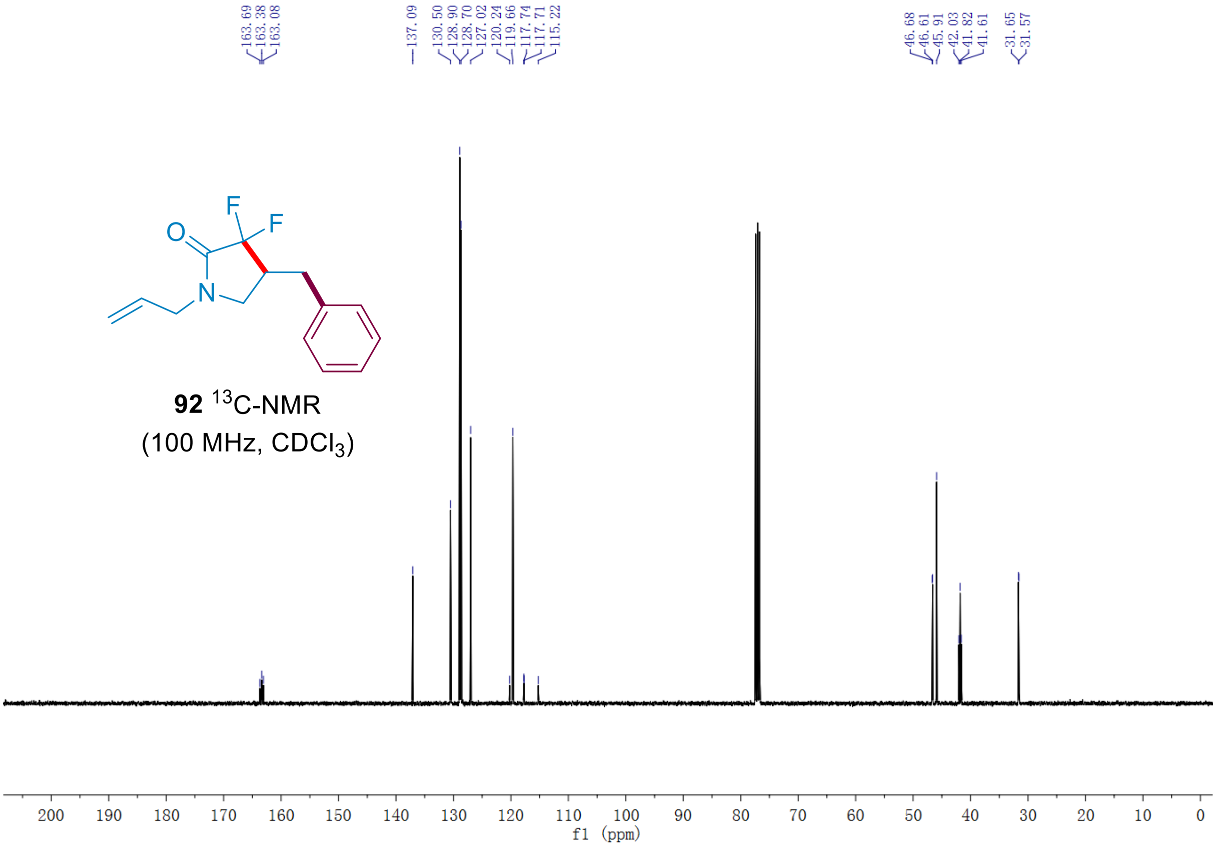

92 13C-NMR
(100 MHz, CDCl3)
f1 (ppm)

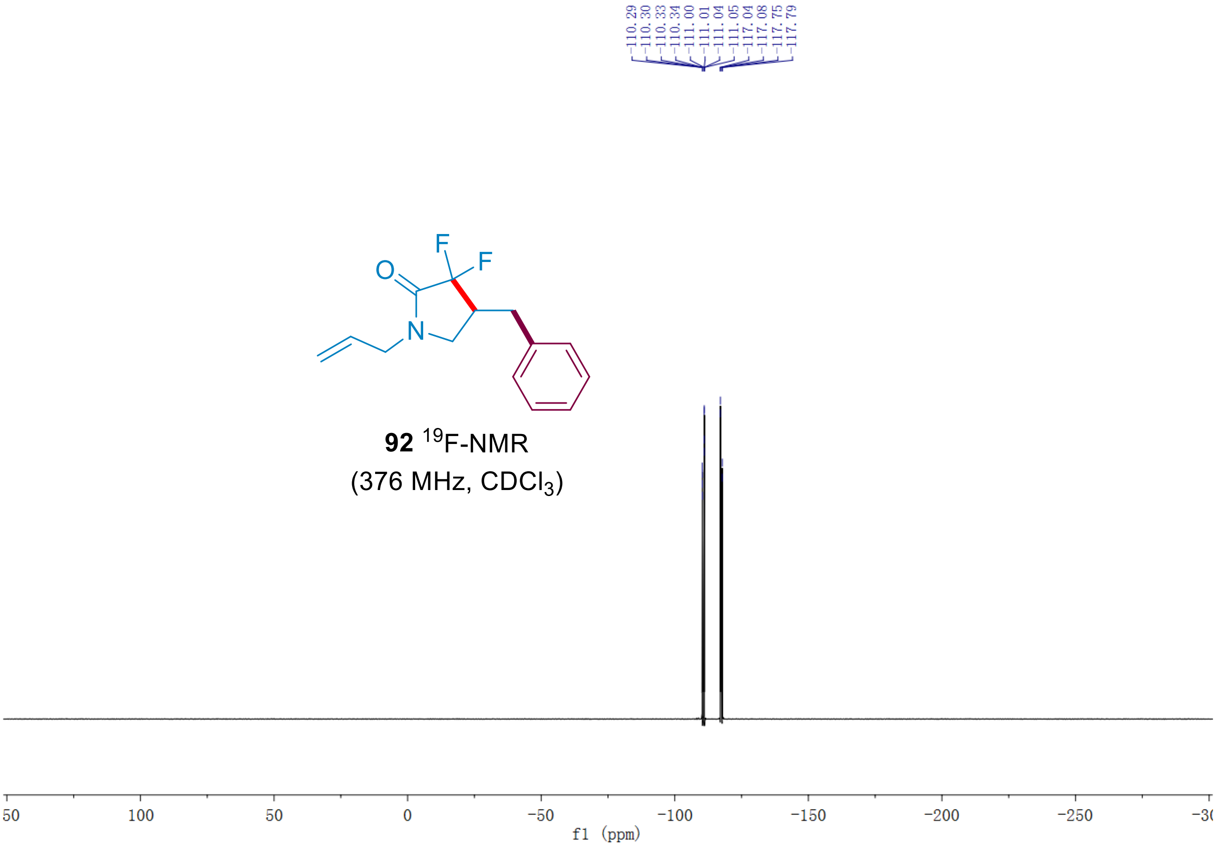

procedures for Fig. S4c:

A suspension of $CoBr_2$ (5.4 mg, 10 mol %), 4-methyloxystyrene (34 mg, 0.25 mmol), bromodifluoroacetate (101 mg, 2.0 equiv), aryl zinc pivalate (0.5 mmol, 2.0 equiv) in degas MeCN (1 mL) was stirred at room temperature for 3 h under an atmosphere of Ar. At ambient temperature, the solvent was evaporated *in vacuo* and the remaining residue was purified by column chromatography on silica gel (*n*-hexane/EtOAc 20:1→10:1) to yield product **11** (60 mg, 63%) and bis-product **93** was also detected by GC and GC-MS.

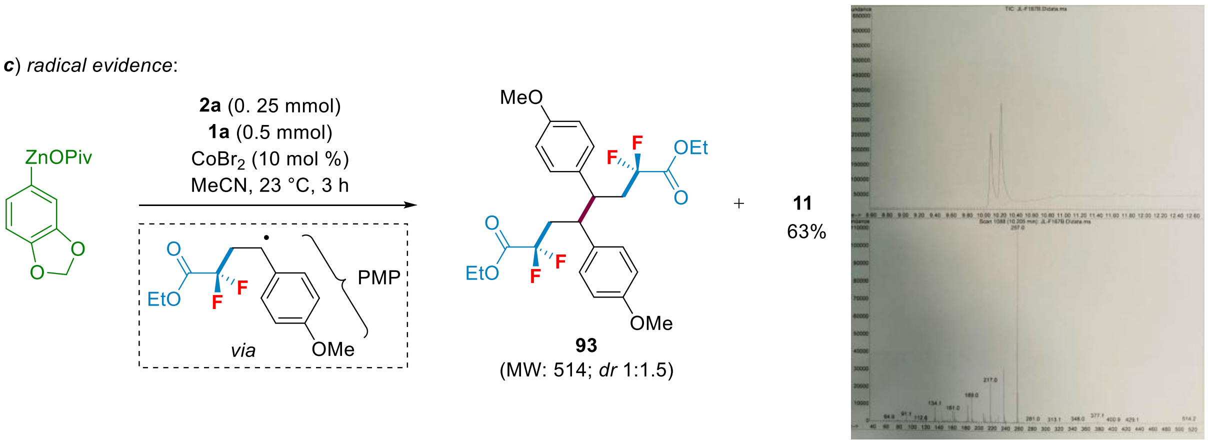

procedures for Fig. S5:

A suspension of $CoBr_2$ (22 mg, 0.1 mmol), 4-methyloxystyrene (34 mg, 0.25 mmol), aryl zinc pivalate (0.5 mmol, 2.0 equiv) in degas MeCN (1 mL) was stirred at room temperature for 30 min under an atmosphere of Ar. At ambient temperature, the solvent was evaporated *in vacuo* and the remaining residue was purified by column chromatography on silica gel (*n*-hexane/EtOAc 100:1) to yield product **94a** (9 mg, 0.041 mmol) or **94b** (11 mg, 0.045 mmol), respectively.

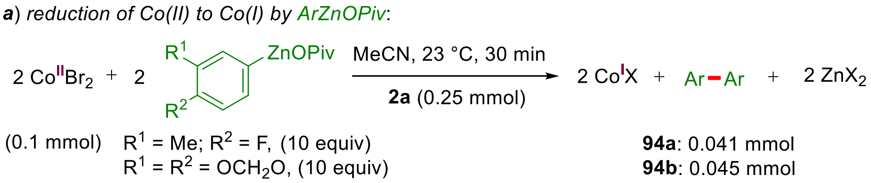

Supplementary Fig. 5 In situ generation of Co(I)-species.

procedures for Fig. S6:

A suspension of $CoCl(PPh_3)_3$ (22 mg, 0.01 mmol) or $Co_2(CO)_8$ (8.5 mg, 0.01 mmol), 4-methyloxystyrene (34 mg, 0.25 mmol), aryl zinc pivalate (0.5 mmol, 2.0 equiv) in degas MeCN (1 mL) was stirred at room temperature for 16 min under an atmosphere of Ar. At ambient temperature, the solvent was evaporated *in vacuo* and the remaining residue was purified by column chromatography on silica gel (*n*-hexane/EtOAc 30:1) to yield product **4** (55 mg, 66% yield; with $CoCl(PPh_3)_3$ as the catalyst) or (17.5 mg, 21% yield; with $Co_2(CO)_8$ as the catalyst).

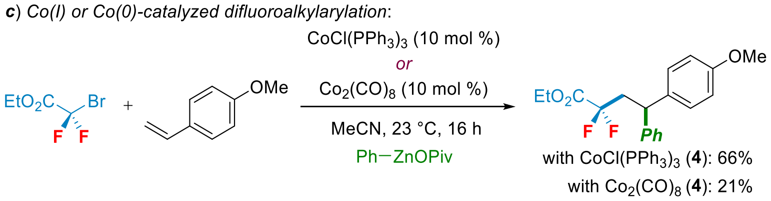

Supplementary Fig. 6 Well-defined Co(I)- or Co(0)-catalyzed difluoroalkylarylation of vinylarene.

procedures for Fig. S7:

A suspension of $CoBr_2$ (5.4 mg, 10 mol %), 4-methyloxystyrene (34 mg, 0.25 mmol), aryl

zinc pivalate (0.05 mmol) in degas MeCN (1 mL) was stirred at room temperature for 30 min under an atmosphere of Ar. Then, bromodifluoroacetate (101 mg, 2.0 equiv), another aryl zinc pivalate (0.5 mmol, 2.0 equiv) were added to the reaction mixture, which was stirred at room temperature for another 3 h. At ambient temperature, the solvent was evaporated *in vacuo* and the remaining residue was purified by column chromatography on silica gel (*n*-hexane/EtOAc 100:1) to yield product **4** (48 mg, 57%) or **11** (75 mg, 79%), respectively.

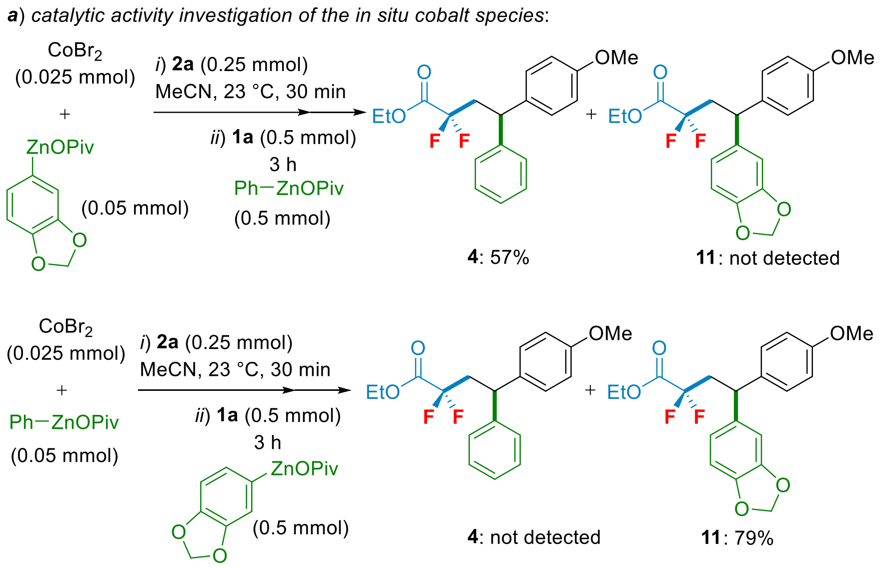

Supplementary Fig. 7 Catalytically active Co(I)-species for difluoroalkylarylation.

e) EPR experiments:

A series of EPR spin-trapping experiments show the existence of *C*-centered radicals trapped by DMPO (g=2.0066, A_N = 13.9 G, A_H = 19.3 G), which was considered to be •CF_2R. These results strongly supported the single electron transfer progress for the activation of $BrCF_2R$ was only promoted by the in situ formed Co(I)-species (Fig. 1).

***e**) EPR experiments*:

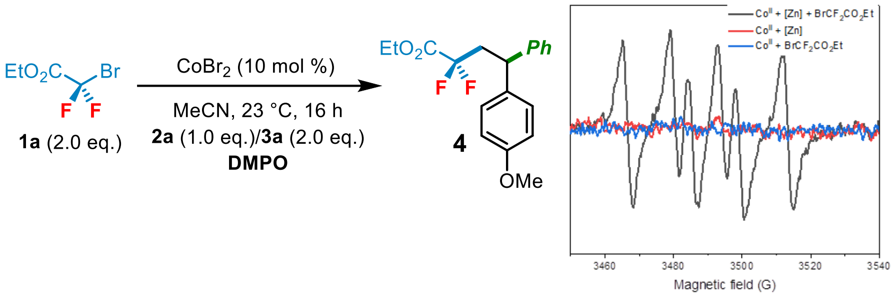

Supplementary Fig. 8 EPR spectra of cobalt-initiated single-electron-transfer process.

f) Substrate scope of unactivated alkenes:

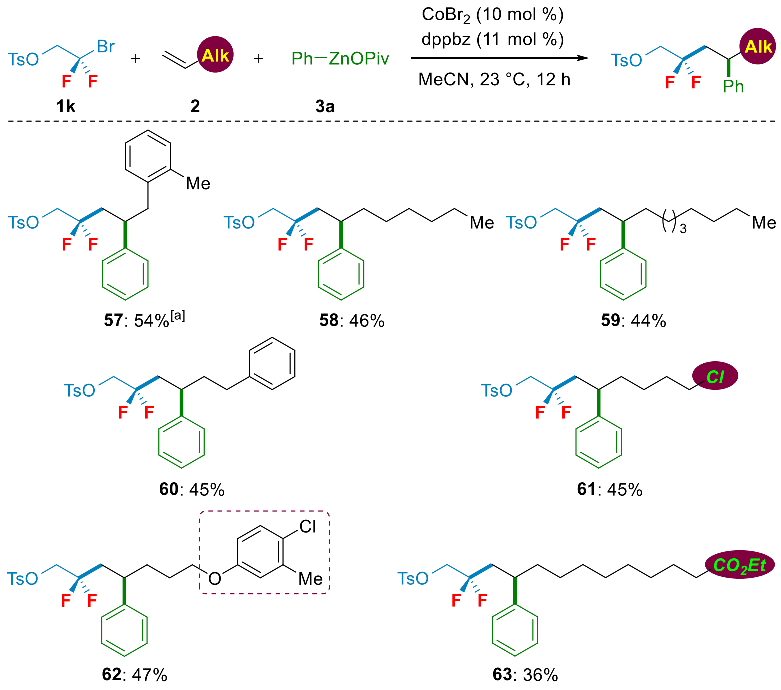

Supplementary Fig. 9 Substrate scope of unactivated alkenes. [a] 33% yield of Heck-type product was detected by ^{1}H-NMR.

g) Effects of directing groups:

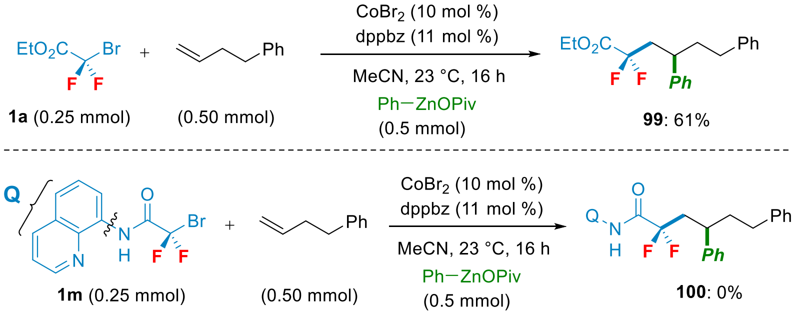

Supplementary Fig. 10. Effects of directing groups.

procedures for Fig. S9:

A suspension of $CoBr_2$ (5.4 mg, 10 mol %), 4-phenyl-1-butene (66 mg, 0.5 mmol), **1a** (0.25 mmol), phenylzinc pivalate (0.5 mmol, 2.0 equiv) in degas MeCN (1 mL) was stirred at room temperature for 16 min under an atmosphere of Ar. At ambient temperature, the solvent was evaporated *in vacuo* and the remaining residue was purified by column chromatography on silica gel (*n*-hexane/EtOAc 30:1) to yield product **99** (50.6 mg, 61% yield) as an oil.

^{1}H-NMR ($CDCl_3$, 400 MHz): δ = 7.32 (t, J = 7.4 Hz, 2H), 7.28 – 7.20 (m, 4H), 7.16 (t, J = 7.4 Hz, 3H), 7.07 (d, J = 7.3 Hz, 2H), 4.00 – 3.83 (m, 2H), 2.94 – 2.83 (m, 1H), 2.60 – 2.32 (m, 4H), 2.10 – 1.87 (m, 2H), 1.17 (t, J = 7.1 Hz, 3H). ^{13}C-NMR ($CDCl_3$, 100 MHz): δ = 163.9 (t, J_{C-F} = 32.9 Hz), 142.8, 141.6, 128.5, 128.4, 128.3, 127.9, 126.9, 125.9, 115.8 (dd, J_{C-F} = 252.2, 248.9 Hz), 62.6, 41.37 (t, J_{C-F} = 22.6 Hz), 39.2 (q, J_{C-F} = 2.9 Hz), 38.9, 33.4, 13.7. ^{19}F-NMR ($CDCl_3$, 376 MHz): δ = -100.19 (d, J = 260.0 Hz), -106.51 (d, J = 260.0 Hz). IR (ATR): 2994, 1726, 1566, 1433, 1211, 882 cm^{-1}. HR-MS (ESI) m/z calcd for $C_{20}H_{23}F_2O_2$ [M+H$^+$] 333.1666, found 333.1275 [M+H$^+$].

Representative Procedures

Preparation of $Zn(OPiv)_2$:

Pivalic acid (20.4 g, 22.6 mL, 200 mmol) was placed in a dry and argon-flushed 500 mL three-necked roundbottom flask, equipped with a magnetic stirring bar, a septum and a pressure equalizer, and was dissolved in dry THF (120 mL). The mixture was cooled to 0 °C, and a solution of Et_2Zn (13.0 g, 10.8 mL, 105 mmol) in dry THF (120 mL) was added over a period of 30 min under vigorous stirring. Then, the ice-bath was removed and stirring was continued at 25 °C for one additional hour at which point bubbling was ceased (a thick slurry was formed). The solvent was removed *in vacuo* and the solid residue was dried for at least 4 h longer. $Zn(OPiv)_2$ was obtained in quantitative yield, as a puffy amorphous white solid.

Typical procedure 1 (TP1) for the preparation of organozinc pivalates:

LiCl (1.5 equiv) was dried under high vacuum and allowed to cool to room temperature, then Mg turnings (1.2 equiv) and THF (1 M solution relating to the aryl bromide) were added. The reaction mixture was cooled to 0 °C and the corresponding aryl bromide (1.0 equiv) was then added. The reaction was stirred at room temperature until iodolysis and protolysis of a reaction aliquot indicated full consumption of the starting material. $Zn(OPiv)_2$ (1.1 equiv) is then added to afford a solution of the corresponding zinc pivalates solution. Evaporation of the THF leads to the solid zinc pivalates.

Representative Procedure (TP2) for cobalt-catalyzed difluoroalkylarylation: A suspension of $CoBr_2$ (10 mol %), olefin (0.25 mmol, 1.0 equiv), difluoroalkyl bromide (0.5 mmol, 2.0 equiv) and aryl zinc pivalates (0.5 mmol, 2.0 equiv) in degas MeCN (1.0 mL) was stirred at 23 °C for 3 h under an atmosphere of Ar. At ambient temperature, the solvent was evaporated *in vacuo* and the remaining residue was purified by column chromatography on silica gel (*n*-hexane/EtOAc) to yield products **4**–**52**, **78**–**86**, **96**–**100**.

Representative Procedure (TP3) for cobalt-catalyzed difluoroalkylarylation of unactivated alkenes: A suspension of $CoBr_2$ (10 mol %), dppbz (11 mol %), olefin (0.5 mmol, 2.0 equiv), difluoroalkyl bromide (0.25 mmol, 1.0 equiv) and aryl zinc pivalates (0.5 mmol, 2.0 equiv) in degas MeCN (1.0 mL) was stirred at 23 °C for 12 h under an atmosphere of Ar. At ambient temperature, the solvent was evaporated *in vacuo* and the remaining residue was purified by column chromatography on silica gel (*n*-hexane/EtOAc) to yield products **53**–**76**.

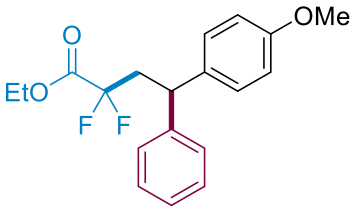

Ethyl 2,2-difluoro-4-(4-methoxyphenyl)-4-phenylbutanoate (4)

The general procedure **TP2** was followed using **1a** (101 mg, 0.5 mmol), **2a** (34 mg, 0.25 mmol), **L5** (5.7 mg) and **3a** (0.5 mmol) for 3 h. Purification by column chromatography (*n*-hexane/EtOAc 20:1) yielded **4** (78 mg, 93%) as an oil. ^{1}H-NMR ($CDCl_3$, 400 MHz): δ = 7.34 – 7.24 (m, 4H), 7.24 – 7.16 (m, 3H), 6.88 – 6.81 (m, 2H), 4.26 (t, J = 7.4 Hz, 1H), 3.89 (q, J = 7.2 Hz, 2H), 3.79 (s, 3H), 2.93 (td, J = 15.4, 7.4 Hz, 2H), 1.20 (t, J = 7.2 Hz, 3H). ^{13}C-NMR ($CDCl_3$, 100 MHz): δ = 163.8 (t, J_{C-F} = 32.6 Hz), 158.3, 143.3, 135.1, 128.7, 128.6, 127.6, 126.7, 115.7 (t, J_{C-F} = 250.6 Hz), 114.0, 62.7, 55.3, 44.0 (t, J_{C-F} = 4.8 Hz), 40.5 (t, J_{C-F} = 23.2 Hz), 13.7. ^{19}F-NMR ($CDCl_3$, 376 MHz): δ = -103.45 (q, J = 15.7 Hz). IR (ATR): 2996, 1723, 1568, 1430, 1216, 1128, 871 cm^{-1}. HR-MS (ESI) m/z calcd for $C_{19}H_{21}F_2O_3$ [M+H$^+$] 335.1459, found 335.1463 [M+H$^+$].

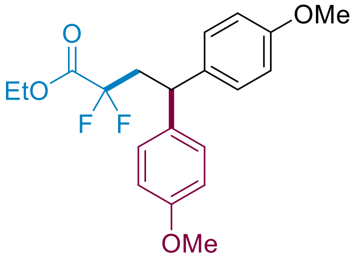

Ethyl 2,2-difluoro-4,4-bis(4-methoxyphenyl)butanoate (5)

The general procedure **TP2** was followed using **1a** (101 mg, 0.5 mmol), **2a** (34 mg, 0.25 mmol) and **3b** (0.5 mmol) for 3 h. Purification by column chromatography (*n*-hexane/EtOAc 20:1) yielded **5** (46 mg, 51%) as an oil. ^{1}H-NMR ($CDCl_3$, 400 MHz): δ = 7.22 – 7.11 (m, 4H), 6.88 – 6.78 (m, 4H), 4.21 (t, J = 7.4 Hz, 1H), 3.90 (q, J = 7.2 Hz, 2H), 3.78 (s, 6H), 2.89 (td, J = 15.4, 7.4 Hz, 2H), 1.21 (t, J = 7.2 Hz, 3H). ^{13}C-NMR ($CDCl_3$, 100 MHz): δ = 163.8 (t, J_{C-F} = 32.6 Hz), 158.3, 135.4, 128.6, 115.7 (t, J = 250.6 Hz), 113.9, 62.7, 55.3, 43.2 (t, J_{C-F} = 4.8 Hz), 40.6 (t, J_{C-F} = 23.1 Hz), 13.7. ^{19}F-NMR ($CDCl_3$, 376 MHz): δ = -103.44 (t, J = 15.4 Hz). IR (ATR): 2996, 1724, 1565, 1439, 1216, 1082 cm^{-1}. HR-MS (ESI) m/z calcd for $C_{20}H_{23}F_2O_4$ [M+H$^+$] 365.1564, found 365.1567 [M+H$^+$].

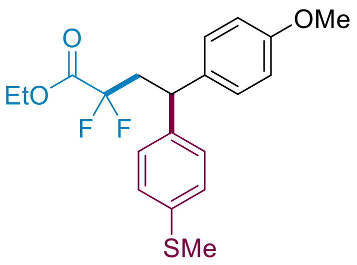

Ethyl 2,2-difluoro-4-(4-methoxyphenyl)-4-[4-(methylthio)phenyl]butanoate (6)

The general procedure **TP2** was followed using **1a** (101 mg, 0.5 mmol), **2a** (34 mg, 0.25 mmol) and **3c** (0.5 mmol) for 3 h. Purification by column chromatography (*n*-hexane/EtOAc 20:1) yielded **6** (38 mg, 40%) as an oil. ^{1}H-NMR ($CDCl_3$, 400 MHz): δ = 7.22 – 7.13 (m, 6H), 6.87 – 6.81 (m, 2H), 4.21 (t, J = 7.4 Hz, 1H), 3.91 (q, J = 7.2 Hz, 2H), 3.78 (s, 3H), 2.89 (td, J = 15.4, 7.4 Hz, 2H), 2.46 (s, 3H), 1.21 (t, J = 7.2 Hz, 3H). ^{13}C-NMR ($CDCl_3$, 100 MHz): δ = 163.8 (t, J_{C-F} = 32.6 Hz), 158.4, 140.3, 136.6, 134.9, 128.7, 128.1, 126.9, 115.7 (d, J_{C-F} = 250.7 Hz), 114.0, 62.8, 55.3, 43.5 (t, J_{C-F} = 4.8 Hz), 40.4 (t, J_{C-F} = 23.2 Hz), 16.0, 13.7. ^{19}F-NMR ($CDCl_3$, 376 MHz): δ = -102.74 (t, J = 15.3 Hz), -103.44 (t, J = 15.4 Hz), -103.53 (t, J = 15.5 Hz), -104.22 (t, J = 15.4 Hz). IR (ATR): 3013, 1697, 1437, 1223, 1121 cm^{-1}. HR-MS (ESI) m/z calcd for $C_{20}H_{23}F_2O_3S^+$ [M+H$^+$] 381.1336, found 381.1342 [M+H$^+$].

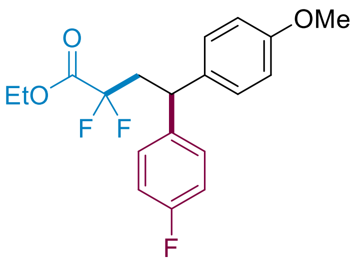

Ethyl 2,2-difluoro-4-(4-fluorophenyl)-4-(4-methoxyphenyl)butanoate (**7**)

The general procedure **TP2** was followed using **1a** (101 mg, 0.5 mmol), **2a** (34 mg, 0.25 mmol) and **3d** (0.5 mmol) for 3 h. Purification by column chromatography (*n*-hexane/EtOAc 20:1) yielded **7** (50 mg, 57%) as an oil. ^{1}H-NMR ($CDCl_3$, 400 MHz): δ = 7.24 – 7.18 (m, 2H), 7.18 – 7.12 (m, 2H), 7.03 – 6.95 (m, 2H), 6.88 – 6.82 (m, 2H), 4.25 (t, J = 7.4 Hz, 1H), 3.98 – 3.89 (m, 2H), 3.79 (s, 3H), 2.96 – 2.81 (m, 2H), 1.22 (t, J = 7.2 Hz, 3H). ^{13}C-NMR ($CDCl_3$, 100 MHz): δ = 163.8 (t, J_{C-F} = 32.6 Hz), 161.5 (d, J_{C-F} = 245.3 Hz), 158.4, 139.1 (d, J_{C-F} = 3.3 Hz), 134.8, 129.1 (d, J_{C-F} = 7.9 Hz), 128.6, 115.6 (t, J_{C-F} = 250.8 Hz), 115.4 (d, J_{C-F} = 21.3 Hz), 114.0, 62.8, 55.3, 43.3 (t, J_{C-F} = 4.7 Hz), 40.5 (t, J_{C-F} = 23.2 Hz), 13.7. ^{19}F-NMR ($CDCl_3$, 376 MHz): δ = -102.79 (t, J = 15.3 Hz), -103.49 (t, J = 15.3 Hz), -103.60 (t, J = 15.3 Hz), -104.28 (t, J = 15.3 Hz), -116.24 – -116.33 (m). IR (ATR): 3032, 2979, 1698, 1413, 1254, 1132 cm^{-1}. HR-MS (ESI) m/z calcd for $C_{19}H_{20}F_3O_3^+$ [M+H$^+$] 353.1365, found 353.1367 [M+H$^+$].

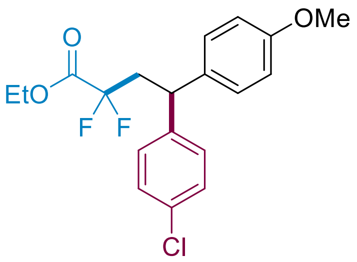

Ethyl 4-(4-chlorophenyl)-2,2-difluoro-4-(4-methoxyphenyl)butanoate (**8**)

The general procedure **TP2** was followed using **1a** (101 mg, 0.5 mmol), **2a** (34 mg, 0.25 mmol) and **3e** ($Ar_2Zn \cdot 2Mg(OPiv)Cl$; 0.5 mmol) for 3 h. Purification by column chromatography (*n*-hexane/EtOAc 20:1) yielded **8** (52 mg, 57%) as an oil. ^{1}H-NMR ($CDCl_3$, 400 MHz): δ = 7.27 (dt, J = 4.5, 1.8 Hz, 2H), 7.21 – 7.12 (m, 4H), 6.87 – 6.82 (m, 2H), 4.24 (t, J = 7.4 Hz, 1H), 3.94 (q, J = 7.2 Hz, 2H), 3.79 (s, 3H), 2.89 (dddd, J = 15.7, 14.7, 7.4, 3.2 Hz, 2H), 1.22 (t, J = 7.2 Hz, 3H). ^{13}C-NMR ($CDCl_3$, 100 MHz): δ = 163.7 (t, J_{C-F} = 32.5 Hz),

158.5, 141.9, 134.5, 132.5, 129.0, 128.7, 128.6, 115.5 (t, J_{C-F} = 250.5 Hz), 114.1, 62.8, 55.3, 43.4 (t, J_{C-F} = 4.7 Hz), 40.3 (t, J_{C-F} = 23.2 Hz), 13.7. ^{19}F-NMR ($CDCl_3$, 376 MHz): δ = -103.40 (t, J = 15.2 Hz), -103.68 (t, J = 15.6 Hz). IR (ATR): 2995, 1698, 1487, 1273, 1083 cm^{-1}. HR-MS (ESI) m/z calcd for $C_{19}H_{20}ClF_2O_3$ [M+H$^+$] 369.1069, found 369.1075 [M+H$^+$].

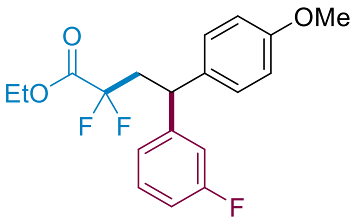

Ethyl 2,2-difluoro-4-(3-fluorophenyl)-4-(4-methoxyphenyl)butanoate (9)

The general procedure **TP2** was followed using **1a** (101 mg, 0.5 mmol), **2a** (34 mg, 0.25 mmol) and **3f** (0.5 mmol) for 3 h. Purification by column chromatography (*n*-hexane/EtOAc 20:1) yielded **9** (46 mg, 52%) as an oil. ^{1}H-NMR ($CDCl_3$, 400 MHz): δ = 7.26 – 7.18 (m, 1H), 7.17 – 7.08 (m, 2H), 7.00 (d, J = 7.7 Hz, 1H), 6.95 – 6.77 (m, 4H), 4.21 (t, J = 7.3 Hz, 1H), 3.97 – 3.85 (m, 2H), 3.75 (s, 3H), 2.94 – 2.78 (m, 2H), 1.19 (t, J = 7.2 Hz, 3H). ^{13}C-NMR ($CDCl_3$, 100 MHz): δ = 163.6, 162.8 (d, J_{C-F} = 246.0 Hz), 158.5, 146.0 (d, J_{C-F} = 6.8 Hz), 134.3, 130.0 (d, J_{C-F} = 8.3 Hz), 128.7, 123.2 (d, J_{C-F} = 2.8 Hz), 115.4 (t, J_{C-F} = 251.0 Hz), 114.5 (d, J_{C-F} = 21.7 Hz), 114.0, 113.5 (d, J_{C-F} = 21.1 Hz), 62.7, 55.2, 43.7 (d, J_{C-F} = 4.7 Hz), 40.2 (t, J_{C-F} = 23.2 Hz), 13.7. ^{19}F-NMR ($CDCl_3$, 376 MHz): δ = -102.60 – -102.80 (m), -103.40 (t, J = 15.2 Hz), -103.73 – -103.96 (m), -104.54 (dd, J = 16.4, 14.8 Hz), -112.77 (ddd, J = 10.0, 8.6, 6.1 Hz). IR (ATR): 3002, 2998, 1686, 1488, 1267, 1145 cm^{-1}. HR-MS (ESI) m/z calcd for $C_{19}H_{20}F_3O_3$ [M+H$^+$] 353.1365, found 353.1367 [M+H$^+$].

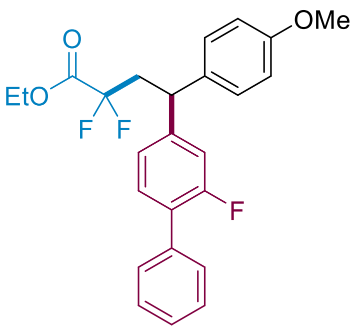

Ethyl 2,2-difluoro-4-(2-fluorobiphenyl-4-yl)-4-(4-methoxyphenyl)butanoate (10)

The general procedure **TP2** was followed using **1a** (101 mg, 0.5 mmol), **2a** (34 mg, 0.25 mmol) and **3g** (0.5 mmol) for 3 h. Purification by column chromatography (*n*-hexane/EtOAc 20:1) yielded **10** (37 mg, 34%) as an oil. ^{1}H-NMR ($CDCl_3$, 400 MHz): δ = 7.55 – 7.50 (m, 2H), 7.48 – 7.42 (m, 2H), 7.40 – 7.35 (m, 2H), 7.25 – 7.18 (m, 2H), 7.12 (dd, J = 8.0, 1.8 Hz, 1H), 7.04 (dd, J = 11.7, 1.7 Hz, 1H), 6.91 – 6.85 (m, 2H), 4.29 (t, J = 7.3 Hz, 1H), 3.97 (q, J = 7.2 Hz, 2H), 3.80 (s, 3H), 2.94 (ddd, J = 16.7, 14.7, 7.4 Hz, 2H), 1.24 (t, J = 7.2 Hz, 3H). ^{13}C-NMR ($CDCl_3$, 100 MHz): δ = 163.7 (t, $J_{C\text{-}F}$ = 32.5 Hz), 159.7 (d, $J_{C\text{-}F}$ = 248.1 MHz), 158.6, 145.0 (d, $J_{C\text{-}F}$ = 7.2 Hz), 135.4, 134.3, 130.8 (d, $J_{C\text{-}F}$ = 4.0 Hz), 128.9 (d, $J_{C\text{-}F}$ = 2.9 Hz), 128.7, 128.5, 127.7, 127.3 (d, $J_{C\text{-}F}$ = 13.6 Hz), 123.6 (d, $J_{C\text{-}F}$ = 3.2 Hz), 115.5 (t, $J_{C\text{-}F}$ = 251.0 Hz), 115.2 (d, $J_{C\text{-}F}$ = 23.5 Hz), 114.1, 62.8, 55.3, 43.6, 40.3 (t, $J_{C\text{-}F}$ = 23.3 Hz), 13.7. ^{19}F-NMR ($CDCl_3$, 376 MHz): δ = -102.64 (t, J = 15.7 Hz), -103.3 (t, J = 15.7 Hz), -103.8 (t, J = 15.7 Hz), -104.51 (t, J = 15.7 Hz), -117.42 – -117.50 (m). IR (ATR): 3021, 2983, 1696, 1470, 1274, 964 cm^{-1}. HR-MS (ESI) m/z calcd for $C_{25}H_{24}F_3O_3$ [M+H$^+$] 429.1678, found 429.1683 [M+H$^+$].

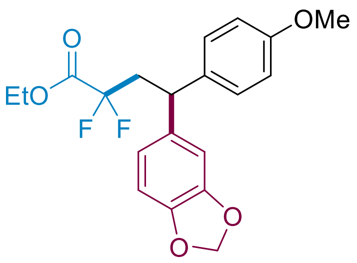

Ethyl 4-(benzo[*d*][1,3]dioxol-5-yl)-2,2-difluoro-4-(4-methoxyphenyl)butanoate (11)

The general procedure **TP2** was followed using **1a** (101 mg, 0.5 mmol), **2a** (34 mg, 0.25 mmol) and **3h** (0.5 mmol) for 3 h. Purification by column chromatography (*n*-hexane/EtOAc 20:1) yielded **11** (60 mg, 63%) as an oil. ^{1}H-NMR ($CDCl_3$, 400 MHz): δ = 7.21 – 7.12 (m, 2H), 6.89 – 6.81 (m, 2H), 6.78 – 6.68 (m, 3H), 5.95 – 5.89 (m, 2H), 4.18 (t, J = 7.4 Hz, 1H), 3.95 (q, J = 7.2 Hz, 2H), 3.79 (s, 3H), 2.97 – 2.78 (m, 2H), 1.23 (t, J = 7.2 Hz, 3H). ^{13}C-NMR ($CDCl_3$, 100 MHz): δ = 163.8 (t, $J_{C\text{-}F}$ = 32.6 Hz), 158.4, 147.8, 146.2, 137.4, 135.1, 128.6, 120.6, 115.7 (t, $J_{C\text{-}F}$ = 250.7 Hz), 114.0, 108.2, 108.1, 101, 62.7, 55.3, 43.9 (t, $J_{C\text{-}F}$ = 4.8 Hz), 40.5 (t, $J_{C\text{-}F}$ = 23.2 Hz), 13.7. ^{19}F-NMR ($CDCl_3$, 376 MHz): δ = -103.43 (t, J =

15.3 Hz), -103.55 (t, J = 15.5 Hz). IR (ATR): 2986, 1679, 1467, 1268, 1082 cm^{-1}. HR-MS (ESI) m/z calcd for $C_{20}H_{21}F_2O_5$ [M+H$^+$] 379.1357, found 379.1361 [M+H$^+$].

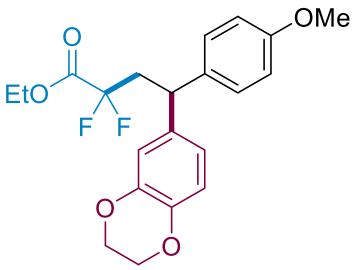

Ethyl 4-(2,3-dihydrobenzo[*b*][1,4]dioxin-6-yl)-2,2-difluoro-4-(4-methoxyphenyl)butanoate (**12**)

The general procedure **TP2** was followed using **1a** (101 mg, 0.5 mmol), **2a** (34 mg, 0.25 mmol) and **3i** (0.5 mmol) for 3 h. Purification by column chromatography (*n*-hexane/EtOAc 20:1) yielded **12** (55 mg, 56%) as an oil. ^{1}H-NMR ($CDCl_3$, 400 MHz): δ = 7.20 – 7.13 (m, 2H), 6.87 – 6.81 (m, 2H), 6.79 (d, J = 8.2 Hz, 1H), 6.76 – 6.69 (m, 2H), 4.27 – 4.19 (m, 4H), 4.14 (t, J = 7.4 Hz, 1H), 3.97 – 3.87 (m, 2H), 3.78 (s, 3H), 2.96 – 2.75 (m, 2H), 1.22 (t, J = 7.2 Hz, 3H). ^{13}C-NMR ($CDCl_3$, 100 MHz): δ = 163.8 (t, J_{C-F} = 32.6 Hz), 158.3, 143.3, 142.2, 136.8, 135.1, 128.7, 120.5, 117.3, 116.3, 115.7 (t, J_{C-F} = 250.0 Hz), 113.9, 64.4, 64.3, 62.7, 55.3, 43.3 (t, J_{C-F} = 4.8 Hz), 40.5 (t, J_{C-F} = 23.1 Hz), 13.7. ^{19}F-NMR ($CDCl_3$, 376 MHz): δ = -102.62 (dd, J = 16.0, 14.2 Hz), -103.24 – -103.36 (m), -103.69 – -103.82 (m), -104.45 (dd, J = 17.0, 14.3 Hz). IR (ATR): 2989, 1684, 1478, 1251, 1146 cm^{-1}. HR-MS (ESI) m/z calcd for $C_{21}H_{23}F_2O_5$ [M+H$^+$] 393.1514, found 393.1518 [M+H$^+$].

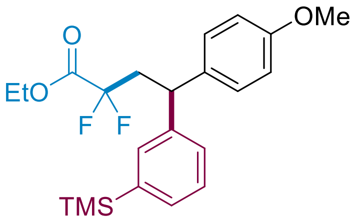

Ethyl 2,2-difluoro-4-(4-methoxyphenyl)-4-[3-(trimethylsilyl)phenyl]butanoate (**13**)

The general procedure **TP2** was followed using **1a** (101 mg, 0.5 mmol), **2a** (34 mg, 0.25 mmol) and **3j** (0.5 mmol) for 3 h. Purification by column chromatography (*n*-hexane/EtOAc 20:1) yielded **13** (55 mg, 54%) as an oil. ^{1}H-NMR ($CDCl_3$, 400 MHz): δ =

7.42 – 7.33 (m, 2H), 7.32 – 7.26 (m, 1H), 7.25 – 7.16 (m, 3H), 6.88 – 6.81 (m, 2H), 4.24 (t, *J* = 7.3 Hz, 1H), 3.84 (q, *J* = 7.2 Hz, 2H), 3.79 (s, 3H), 3.04 – 2.80 (m, 2H), 1.17 (t, *J* = 7.2 Hz, 3H), 0.32 – 0.22 (m, 9H). ^{13}C-NMR ($CDCl_3$, 100 MHz): δ = 163.8 (t, J_{C-F} = 32.6 Hz), 158.3, 142.5, 141.0, 135.0, 132.5, 131.7, 128.8, 128.0, 127.9, 115.7 (d, J_{C-F} = 250.5 Hz), 113.9, 62.7, 55.3, 44.2 (t, J_{C-F} = 4.8 Hz), 40.6 (t, J_{C-F} = 23.2 Hz), 13.7, -1.1. ^{19}F-NMR ($CDCl_3$, 376 MHz): δ = -102.51 (dd, *J* = 17.0, 13.2 Hz), -103.20 (dd, *J* = 16.8, 13.4 Hz), -103.67 (dd, *J* = 17.5, 13.7 Hz), -104.36 (dd, *J* = 17.7, 13.5 Hz). IR (ATR): 2987, 1674, 1489, 1266, 1126 cm^{-1}. HR-MS (ESI) m/z calcd for $C_{22}H_{29}F_2O_2Si$ [M+H$^+$] 407.1854, found 407.1856 [M+H$^+$].

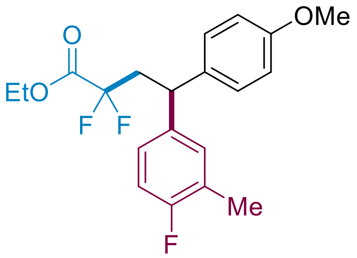

Ethyl 2,2-difluoro-4-(4-fluoro-3-methylphenyl)-4-(4-methoxyphenyl)butanoate (**14**)

The general procedure **TP2** was followed using **1a** (101 mg, 0.5 mmol), **2a** (34 mg, 0.25 mmol) and **3k** ($Ar_2Zn \cdot 2Mg(OPiv)Cl$; 0.5 mmol) for 3 h. Purification by column chromatography (*n*-hexane/EtOAc 20:1) yielded **14** (56 mg, 61%) as an oil. ^{1}H-NMR ($CDCl_3$, 400 MHz): δ = 7.19 – 7.13 (m, 2H), 7.08 – 7.00 (m, 2H), 6.92 (dd, *J* = 11.5, 6.1 Hz, 1H), 6.88 – 6.82 (m, 2H), 4.20 (t, *J* = 7.4 Hz, 1H), 3.91 (q, *J* = 7.2 Hz, 2H), 3.79 (s, 3H), 2.88 (dddd, *J* = 15.9, 14.6, 7.4, 3.2 Hz, 2H), 2.25 (d, *J* = 1.8 Hz, 3H), 1.22 (t, *J* = 7.2 Hz, 3H). ^{13}C-NMR ($CDCl_3$, 100 MHz): δ = 163.8 (t, J_{C-F} = 32.6 Hz), 160.1 (d, J_{C-F} = 243.9 Hz), 158.4, 138.8 (d, J_{C-F} = 3.7 Hz), 135.0, 130.7 (d, J_{C-F} = 5.1 Hz), 128.6, 126.2 (d, J_{C-F} = 7.9 Hz), 124.9 (d, J_{C-F} = 17.4 Hz), 115.6 (t, J_{C-F} = 250.7 Hz), 115.0 (d, J_{C-F} = 22.3 Hz), 114.0, 62.8, 55.3, 43.3 (t, J_{C-F} = 4.8 Hz), 40.5 (t, J_{C-F} = 23.2 Hz), 14.7 (d, J_{C-F} = 3.5 Hz), 13.7. ^{19}F-NMR ($CDCl_3$, 376 MHz): δ = -103.46 (t, *J* = 15.5 Hz), -103.61 (t, *J* = 15.7 Hz), -120.43 – -120.72 (m). IR (ATR): 3181, 2993, 1687, 1464, 1272, 935 cm^{-1}. HR-MS (ESI) m/z calcd for $C_{20}H_{22}F_3O_3$ [M+H$^+$] 367.1521, found 367.1522 [M+H$^+$].

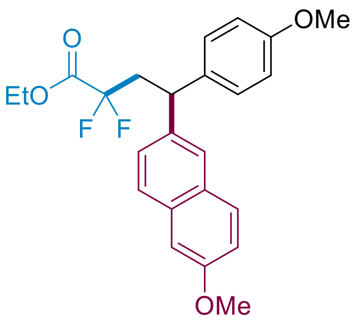

Ethyl 2,2-difluoro-4-(6-methoxynaphthalen-2-yl)-4-(4-methoxyphenyl)butanoate (**15**)

The general procedure **TP2** was followed using **1a** (101 mg, 0.5 mmol), **2a** (34 mg, 0.25 mmol) and **3l** (0.5 mmol) for 3 h. Purification by column chromatography (*n*-hexane/EtOAc 20:1→10:1) yielded **15** (54 mg, 52%) as an oil. ^{1}H-NMR ($CDCl_3$, 400 MHz): δ = 7.71 (d, J = 9.0 Hz, 1H), 7.66 (d, J = 8.5 Hz, 1H), 7.63 (s, 1H), 7.30 (dd, J = 8.5, 1.8 Hz, 1H), 7.26 – 7.19 (m, 2H), 7.15 (dd, J = 8.9, 2.5 Hz, 1H), 7.10 (d, J = 2.4 Hz, 1H), 6.89 – 6.81 (m, 2H), 4.39 (t, J = 7.4 Hz, 1H), 3.91 (d, J = 5.5 Hz, 3H), 3.83 – 3.69 (m, 5H), 3.13 – 2.90 (m, 2H), 1.12 (t, J = 7.2 Hz, 3H). ^{13}C-NMR ($CDCl_3$, 100 MHz): δ = 163.8 (t, $J_{C\text{–}F}$ = 32.6 Hz), 158.3, 157.6, 138.3, 135.1, 133.4, 129.2, 128.9, 128.8, 127.2, 126.8, 125.6, 119.0, 115.8 (d, $J_{C\text{–}F}$ = 250.6 Hz), 114.0, 105.6, 62.7, 55.3, 55.3, 43.9 (t, $J_{C\text{–}F}$ = 4.8 Hz), 40.4 (t, $J_{C\text{–}F}$ = 23.2 Hz), 13.6. ^{19}F-NMR ($CDCl_3$, 376 MHz): δ = -102.47 – -102.61 (m), -103.18 – -103.28 (m), -103.28 – -103.37 (m), -103.96 – -104.07 (m). IR (ATR): 3362, 2987, 1712, 1464, 1282 cm^{-1}. HR-MS (ESI) m/z calcd for $C_{24}H_{25}F_2O_4$ [M+H$^+$] 415.1721, found 415.1727 [M+H$^+$].

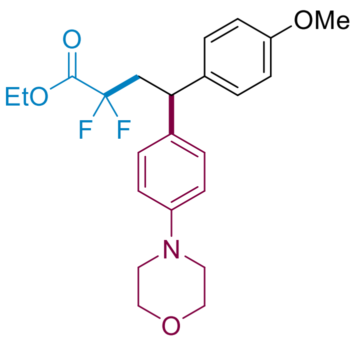

Ethyl 2,2-difluoro-4-(4-methoxyphenyl)-4-(4-morpholinophenyl)butanoate (**16**)

The general procedure **TP2** was followed using **1a** (101 mg, 0.5 mmol), **2a** (34 mg, 0.25 mmol) and **3m** (0.5 mmol) for 3 h. Purification by column chromatography (*n*-hexane/EtOAc 20:1→10:1→5:1) yielded **16** (56 mg, 53%) as an oil. ^{1}H-NMR ($CDCl_3$, 400 MHz): δ = 7.23 – 7.08 (m, 4H), 6.94 – 6.75 (m, 4H), 4.18 (t, J = 7.4 Hz, 1H), 3.96 – 3.82 (m,

6H), 3.78 (s, 3H), 3.17 – 3.06 (m, 4H), 2.99 – 2.75 (m, 2H), 1.20 (t, J = 7.2 Hz, 3H). ^{13}C-NMR ($CDCl_3$, 100 MHz): δ = 163.9 (t, J_{C-F} = 32.6 Hz), 158.2, 149.9, 135.4, 134.7, 128.7, 128.3, 115.8 (d, J_{C-F} = 250.5 Hz), 115.7, 113.9, 66.9, 62.7, 55.3, 49.3, 43.1 (t, J_{C-F} = 4.8 Hz), 40.6 (t, J_{C-F} = 23.0 Hz), 13.7. ^{19}F-NMR ($CDCl_3$, 376 MHz): δ = -102.67 (t, J = 15.3 Hz), -103.36 (t, J = 15.2 Hz), -103.58 (t, J = 15.5 Hz), -104.27 (t, J = 15.5 Hz). IR (ATR): 3288, 2987, 1776, 1434, 1278 cm^{-1}. HR-MS (ESI) m/z calcd for $C_{23}H_{28}F_2NO_4$ [M+H$^+$] 420.1986, found 420.1984 [M+H$^+$].

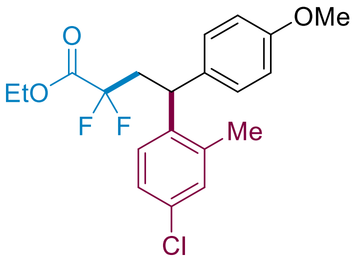

Ethyl 4-(4-chloro-2-methylphenyl)-2,2-difluoro-4-(4-methoxyphenyl)butanoate (**17**)

The general procedure **TP2** was followed using **1a** (101 mg, 0.5 mmol), **2a** (34 mg, 0.25 mmol) and **3n** (0.5 mmol) for 3 h. Purification by column chromatography (*n*-hexane/EtOAc 20:1) yielded **17** (59 mg, 62%) as an oil. ^{1}H-NMR ($CDCl_3$, 400 MHz): δ = 7.19 (dt, J = 8.4, 5.2 Hz, 2H), 7.15 – 7.08 (m, 3H), 6.85 – 6.79 (m, 2H), 4.44 (t, J = 7.3 Hz, 1H), 4.00 – 3.84 (m, 2H), 3.78 (s, 3H), 2.96 – 2.74 (m, 2H), 2.31 (s, 3H), 1.21 (t, J = 7.2 Hz, 3H). ^{13}C-NMR ($CDCl_3$, 100 MHz): δ = 163.8 (t, J_{C-F} = 32.6 Hz), 158.4, 139.7, 138.0, 133.9, 132.1, 130.6, 128.9, 127.9, 126.1, 115.6 (t, J_{C-F} = 250.9 Hz), 114.0, 62.8, 55.3, 40.5 (t, J_{C-F} = 23.1 Hz), 38.9 (t, J_{C-F} = 4.7 Hz), 19.7, 13.7. ^{19}F-NMR ($CDCl_3$, 376 MHz): δ = -102.63 – -102.77 (m), -103.32 – -103.45 (m), -103.64 – -103.77 (m), -104.33 – -104.46 (m). IR (ATR): 2989, 1696, 1478, 1274, 1084 cm^{-1}. HR-MS (ESI) m/z calcd for $C_{20}H_{22}ClF_2O_3$ [M+H$^+$] 383.1226, found 383.1229 [M+H$^+$].

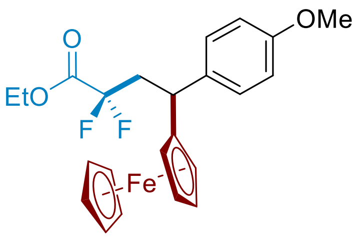

Ethyl 2,2-difluoro-4-(4-methoxyphenyl)-4-(ferrocenyl)butanoate (**18**)

The general procedure **TP2** was followed using **1a** (101 mg, 0.5 mmol), **2a** (34 mg, 0.25 mmol) and **3o** (0.5 mmol) for 3 h. Purification by column chromatography (*n*-hexane/EtOAc 20:1) yielded **18** (51 mg, 46%) as an oil. ^{1}H-NMR ($CDCl_3$, 400 MHz): δ = 7.13 – 7.07 (m, 2H), 6.82 – 6.76 (m, 2H), 4.12 – 4.08 (m, 7H), 4.06 (dd, J = 4.1, 2.0 Hz, 1H), 3.97 – 3.79 (m, 4H), 3.76 (s, 3H), 2.92 (qd, J = 14.5, 3.0 Hz, 1H), 2.78 (ddt, J = 22.4, 14.4, 11.1 Hz, 1H), 1.11 (t, J = 7.1 Hz, 3H). ^{13}C-NMR ($CDCl_3$, 100 MHz): δ = 163.8 (t, J_{C-F} = 32.6 Hz), 158.3, 135.16 (s), 129.0, 115.8 (td, J_{C-F} = 252.0, 248.4 Hz), 113.6, 93.6, 68.7, 67.9, 67.5, 67.3, 66.1, 62.7, 55.2, 41.6 (t, J_{C-F} = 23.0 Hz), 38.9 (dd, J_{C-F} = 7.0, 3.0 Hz), 13.7. ^{19}F-NMR ($CDCl_3$, 376 MHz): δ = -99.84 (d, J = 260.1 Hz), -106.61 (d, J = 260.1 Hz). IR (ATR): 2989, 1695, 1478, 1274, 1084 cm^{-1}. HR-MS (ESI) m/z calcd for $C_{23}H_{25}F_2FeO_3$ [M+H$^+$] 443.1121, found 443.1125 [M+H$^+$].

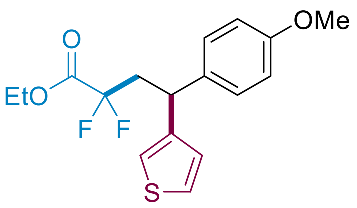

Ethyl 2,2-difluoro-4-(4-methoxyphenyl)-4-(thiophen-3-yl)butanoate (**19**)

The general procedure **TP2** was followed using **1a** (101 mg, 0.5 mmol), **2a** (34 mg, 0.25 mmol) and **3p** (0.5 mmol) for 3 h. Purification by column chromatography (*n*-hexane/EtOAc 20:1) yielded **19** (24 mg, 28%) as an oil. ^{1}H-NMR ($CDCl_3$, 400 MHz): δ = 7.26 (dd, J = 5.0, 2.9 Hz, 1H), 7.20 – 7.14 (m, 2H), 7.03 – 6.99 (m, 1H), 6.91 (dd, J = 5.0, 1.3 Hz, 1H), 6.88 – 6.82 (m, 2H), 4.32 (t, J = 7.3 Hz, 1H), 3.92 (dtt, J = 10.7, 7.0, 3.6 Hz, 2H), 3.80 (s, 3H), 2.96 – 2.82 (m, 2H), 1.22 (t, J = 7.2 Hz, 3H). ^{13}C-NMR ($CDCl_3$, 100 MHz): δ = 163.8 (t, J_{C-F} = 32.5 Hz), 158.4, 144.3, 134.4, 128.9, 127.4, 126.0, 120.6, 115.6 (t, J_{C-F} = 252.4 Hz), 113.9, 62.8, 55.3, 40.9 (t, J_{C-F} = 23.2 Hz), 39.8 (d, J = 5.2 Hz), 13.8. ^{19}F-NMR ($CDCl_3$, 376 MHz): δ = -102.28 (dd, J = 16.8, 13.1 Hz), -102.98 (dd, J = 17.1, 12.8 Hz), -104.07 (dd, J = 18.2, 13.2 Hz), -104.76 (dd, J = 17.9, 13.5 Hz). IR (ATR): 2986, 1687, 1476, 1266, 842 cm^{-1}. HR-MS (ESI) m/z calcd for $C_{17}H_{19}F_2O_3S$ [M+H$^+$] 341.1023, found 341.1026 [M+H$^+$].

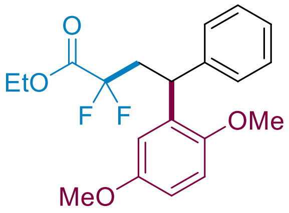

Ethyl 4-(2,5-dimethoxyphenyl)-2,2-difluoro-4-phenylbutanoate (20)

The general procedure **TP2** was followed using **1a** (101 mg, 0.5 mmol), **2b** (26 mg, 0.25 mmol) and **3r** (0.5 mmol) for 3 h. Purification by column chromatography (*n*-hexane/EtOAc 20:1) yielded **20** (83 mg, 91%) as an oil. ^{1}H-NMR ($CDCl_3$, 400 MHz): δ = 7.34 – 7.25 (m, 4H), 7.22 – 7.16 (m, 1H), 6.79 (dd, J = 8.2, 5.9 Hz, 2H), 6.71 (dd, J = 8.9, 3.0 Hz, 1H), 4.71 (t, J = 7.3 Hz, 1H), 3.89 (q, J = 7.2 Hz, 2H), 3.77 (s, 3H), 3.76 (s, 3H), 2.91 (ddd, J = 16.7, 14.6, 7.4 Hz, 2H), 1.20 (dd, J = 9.9, 4.4 Hz, 3H). ^{13}C-NMR ($CDCl_3$, 100 MHz): δ = 163.9 (t, J_{C-F} = 32.7 Hz), 153.5, 151.0, 142.3, 132.7, 128.3, 128.1, 126.5, 115.8 (t, J_{C-F} = 250.8 Hz), 115.0, 111.9, 111.3, 62.6, 56.1, 55.6, 39.3 (t, J_{C-F} = 23.3 Hz), 38.0 (t, J_{C-F} = 5.0 Hz), 13.7. ^{19}F-NMR ($CDCl_3$, 376 MHz): δ = -102.46 (t, J = 15.2 Hz), -103.14 (t, J = 15.2 Hz), -103.88 (t, J = 15.8 Hz), -104.49 – -104.64 (m). IR (ATR): 2988, 1696, 1478, 1272, 942 cm^{-1}. HR-MS (ESI) m/z calcd for $C_{20}H_{23}F_2O_4$ [M+H$^+$] 365.1564, found 365.1571 [M+H$^+$].

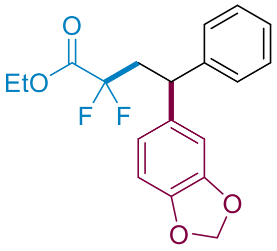

Ethyl 4-(benzo[*d*][1,3]dioxol-5-yl)-2,2-difluoro-4-phenylbutanoate (21)

The general procedure **TP2** was followed using **1a** (101 mg, 0.5 mmol), **2b** (26 mg, 0.25 mmol) and **3h** (0.5 mmol) for 3 h. Purification by column chromatography (*n*-hexane/EtOAc 20:1) yielded **21** (50 mg, 57%) as an oil. ^{1}H-NMR ($CDCl_3$, 400 MHz): δ = 7.35 – 7.19 (m, 5H), 6.74 (dd, J = 4.4, 0.9 Hz, 3H), 5.94 – 5.90 (m, 2H), 4.23 (t, J = 7.3 Hz, 1H), 3.94 (q, J = 7.2 Hz, 2H), 2.90 (tdd, J = 15.2, 7.4, 2.1 Hz, 2H), 1.22 (t, J = 7.2 Hz, 3H). ^{13}C-NMR ($CDCl_3$, 100 MHz): δ = 163.8 (t, J_{C-F} = 32.6 Hz), 147.8, 146.3, 143.0, 137.0, 128.6, 127.6, 126.8, 120.7, 115.6 (t, J_{C-F} = 250.3 Hz), 108.2, 101.0, 62.7, 44.5 (t, J_{C-F} = 4.7 Hz), 40.3 (t, J_{C-F} = 23.3 Hz), 13.7. ^{19}F-NMR ($CDCl_3$, 376 MHz): δ = -103.51 (td, J = 15.4, 9.1 Hz). IR

(ATR): 2992, 1693, 1487, 1271, 1088 cm^{-1}. HR-MS (ESI) m/z calcd for $C_{19}H_{19}F_2N_4$ [M+H$^+$] 349.1251, found 349.1252 [M+H$^+$].

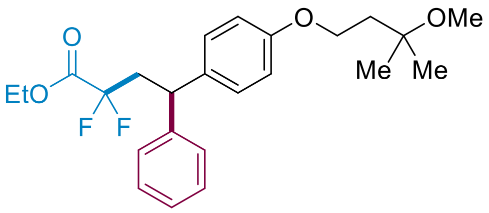

Ethyl 2,2-difluoro-4-[4-(3-methoxy-3-methylbutoxy)phenyl]-4-phenylbutanoate (22)

The general procedure **TP2** was followed using **1a** (101 mg, 0.5 mmol), **2c** (55 mg, 0.25 mmol) and **3a** (0.5 mmol) for 3 h. Purification by column chromatography (*n*-hexane/EtOAc 20:1) yielded **22** (82 mg, 78%) as an oil. ^{1}H-NMR ($CDCl_3$, 400 MHz): δ = 7.33 – 7.14 (m, 7H), 6.85 – 6.82 (m, 2H), 4.25 (t, J = 7.3 Hz, 1H), 4.04 (t, J = 7.1 Hz, 2H), 3.88 (q, J = 7.2 Hz, 2H), 3.23 (s, 3H), 2.92 (td, J = 15.4, 7.4 Hz, 2H), 2.00 (t, J = 7.1 Hz, 2H), 1.25 (s, 6H), 1.20 (t, J = 7.2 Hz, 3H). ^{13}C-NMR ($CDCl_3$, 100 MHz): δ = 163.8 (t, $J_{C\text{-}F}$ = 32.6 Hz), 157.7, 143.4, 134.9, 129.6, 128.7, 128.6, 127.6, 126.7, 120.6, 115.7 (t, $J_{C\text{-}F}$ = 250.6 Hz), 115.3, 114.5, 73.8, 64.2, 62.7, 49.3, 44.0 (t, $J_{C\text{-}F}$ = 4.8 Hz), 40.5 (t, $J_{C\text{-}F}$ = 23.2 Hz), 38.9, 25.4, 13.7. ^{19}F-NMR ($CDCl_3$, 376 MHz): δ = -103.43 (td, J = 15.3, 6.3 Hz). IR (ATR): 3342, 2978, 1676, 1466, 1274, 1146 cm^{-1}. HR-MS (ESI) m/z calcd for $C_{24}H_{31}F_2O_4$ [M+H$^+$] 421.2190, found 421.2199 [M+H$^+$].

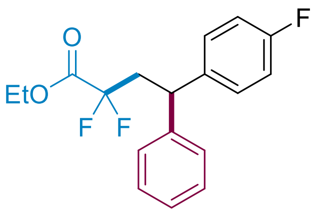

Ethyl 2,2-difluoro-4-(4-fluorophenyl)-4-phenylbutanoate (23)

The general procedure **TP2** was followed using **1a** (101 mg, 0.5 mmol), **2d** (31 mg, 0.25 mmol) and **3a** (0.5 mmol) for 3 h. Purification by column chromatography (*n*-hexane/EtOAc 20:1) yielded **23** (71 mg, 88%) as an oil. ^{1}H-NMR ($CDCl_3$, 400 MHz): δ = 7.38 – 7.29 (m, 2H), 7.23 (tdd, J = 5.9, 4.2, 1.5 Hz, 5H), 7.05 – 6.95 (m, 2H), 4.31 (t, J = 7.3 Hz, 1H), 3.92 (q, J = 7.2 Hz, 2H), 3.04 – 2.80 (m, 2H), 1.25 – 1.18 (m, 3H). ^{13}C-NMR ($CDCl_3$, 100 MHz): δ = 163.7 (t, $J_{C\text{-}F}$ = 32.5 Hz), 161.6 (d, $J_{C\text{-}F}$ = 245.5 Hz), 142.8, 138.7 (d, J_{C}

$_{-F}$ = 3.3 Hz), 129.3 (d, J_{C-F} = 8.0 Hz), 128.7, 127.6, 126.9, 115.5 (t, J_{C-F} = 251.0 Hz), 115.4 (d, J_{C-F} = 21.3 Hz), 62.8, 44.1 (t, J_{C-F} = 4.7 Hz), 40.3 (t, J_{C-F} = 23.3 Hz), 13.7. ^{19}F-NMR (CDCl$_3$, 376 MHz): δ = -103.53 (td, J = 15.8, 8.9 Hz), -116.09 (tt, J = 8.8, 5.4 Hz). IR (ATR): 3122, 2973, 1676, 1457, 1269, 769 cm^{-1}. HR-MS (ESI) m/z calcd for $C_{18}H_{18}F_3O_2$ [M+H$^+$] 323.1259, found 323.1263 [M+H$^+$].

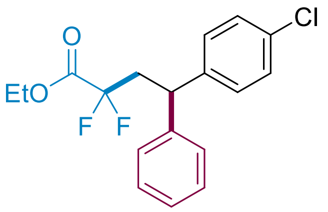

Ethyl 4-(4-chlorophenyl)-2,2-difluoro-4-phenylbutanoate (**24**)

The general procedure **TP2** was followed using **1a** (101 mg, 0.5 mmol), **2e** (35 mg, 0.25 mmol) and **3a** (0.5 mmol) for 3 h. Purification by column chromatography (*n*-hexane/EtOAc 20:1) yielded **24** (42 mg, 50%) as an oil. ^{1}H-NMR (CDCl$_3$, 400 MHz): δ = 7.37 – 7.18 (m, 9H), 4.29 (t, J = 7.3 Hz, 1H), 3.93 (q, J = 7.2 Hz, 2H), 3.03 – 2.83 (m, 2H), 1.22 (t, J = 7.2 Hz, 3H). ^{13}C-NMR (CDCl$_3$, 100 MHz): δ = 163.7 (t, J_{C-F} = 32.5 Hz), 142.5, 141.5, 132.6, 129.1, 128.8, 127.6, 127.0, 115.5 (t, J_{C-F} = 251.0 Hz), 62.8, 44.2 (t, J_{C-F} = 4.7 Hz), 40.1 (t, J_{C-F} = 23.3 Hz), 13.7. ^{19}F-NMR (CDCl$_3$, 376 MHz): δ = -102.77 (t, J = 15.3 Hz), -103.47 (t, J = 15.3 Hz), -103.61 (t, J = 15.5 Hz), -104.31 (t, J = 15.5 Hz). IR (ATR): 2988, 1689, 1492, 1273, 843 cm^{-1}. HR-MS (ESI) m/z calcd for $C_{18}H_{18}ClF_2O_2$ [M+H$^+$] 339.0963, found 339.0967 [M+H$^+$].

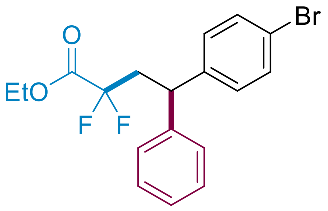

Ethyl 4-(4-bromophenyl)-2,2-difluoro-4-phenylbutanoate (**25**)

The general procedure **TP2** was followed using **1a** (101 mg, 0.5 mmol), **2f** (46 mg, 0.25 mmol) and **3a** (0.5 mmol) for 3 h. Purification by column chromatography (*n*-hexane/EtOAc 20:1) yielded **25** (73 mg, 76%) as an oil. ^{1}H-NMR (CDCl$_3$, 400 MHz): δ =

7.47 – 7.40 (m, 2H), 7.34 – 7.29 (m, 2H), 7.27 – 7.20 (m, 3H), 7.18 – 7.13 (m, 2H), 4.28 (t, *J* = 7.3 Hz, 1H), 3.93 (q, *J* = 7.2 Hz, 2H), 3.03 – 2.82 (m, 2H), 1.22 (t, *J* = 7.2 Hz, 3H). ^{13}C-NMR ($CDCl_3$, 100 MHz): δ = 163.7 (t, J_{C-F} = 32.5 Hz), 142.4, 142.0, 131.7, 129.5, 128.8, 127.6, 127.0, 120.7, 115.5 (t, J_{C-F} = 251.1 Hz), 62.8, 44.3 (t, J_{C-F} = 4.7 Hz), 40.0 (t, J_{C-F} = 23.3 Hz), 13.7. ^{19}F-NMR ($CDCl_3$, 376 MHz): δ = -102.76 (t, *J* = 15.3 Hz), -103.45 (t, *J* = 15.3 Hz), -103.60 (t, *J* = 15.5 Hz), -104.30 (t, *J* = 15.5 Hz). IR (ATR): 2939, 1684, 1478, 1264, 1132 cm^{-1}. HR-MS (ESI) m/z calcd for $C_{18}H_{18}ClF_2O_2$ [M+H$^+$] 383.0458, found 383.0461 [M+H$^+$].

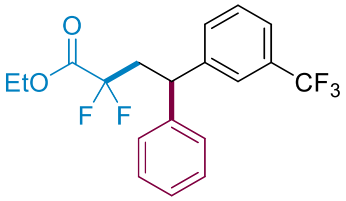

Ethyl 2,2-difluoro-4-phenyl-4-[3-(trifluoromethyl)phenyl]butanoate (26)

The general procedure **TP2** was followed using **1a** (101 mg, 0.5 mmol), **2g** (43 mg, 0.25 mmol) and **3a** (0.5 mmol) for 3 h. Purification by column chromatography (*n*-hexane/EtOAc 20:1) yielded **26** (62 mg, 67%) as an oil. ^{1}H-NMR ($CDCl_3$, 400 MHz): δ = 7.53 (s, 1H), 7.46 (tt, *J* = 15.0, 7.4 Hz, 3H), 7.37 – 7.30 (m, 2H), 7.28 – 7.22 (m, 3H), 4.38 (t, *J* = 7.3 Hz, 1H), 3.93 (q, *J* = 7.2 Hz, 2H), 3.07 – 2.86 (m, 2H), 1.21 (t, *J* = 7.2 Hz, 3H). ^{13}C-NMR ($CDCl_3$, 100 MHz): δ = 163.6 (t, J_{C-F} = 32.5 Hz), 144.0, 142.0, 131.2, 131.0 (q, J_{C-F} = 32.2 Hz), 129.2, 128.9, 127.7, 127.2, 124.4 (q, J_{C-F} = 3.8 Hz), 124.0 (q, J_{C-F} = 272.1 Hz), 123.7 (q, J_{C-F} = 3.8 Hz), 115.4 (t, J_{C-F} = 251.2 Hz), 62.9, 44.7 (t, J_{C-F} = 4.6 Hz), 40.1 (t, J_{C-F} = 23.4 Hz), 13.7. ^{19}F-NMR ($CDCl_3$, 376 MHz): δ = -62.55 (s), -102.67 – -102.78 (m), -103.42 (t, *J* = 15.5 Hz), -103.65 – -103.80 (m), -104.36 – -104.49 (m). IR (ATR): 3334, 2989, 1664, 1457, 1276, 647 cm^{-1}. HR-MS (ESI) m/z calcd for $C_{19}H_{18}F_5O_2$ [M+H$^+$] 373.1227, found 373.1224 [M+H$^+$].

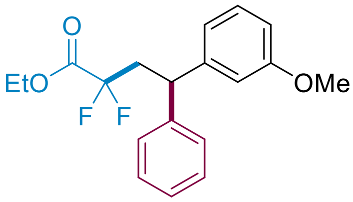

Ethyl 2,2-difluoro-4-(3-methoxyphenyl)-4-phenylbutanoate (27)

The general procedure **TP2** was followed using **1a** (101 mg, 0.5 mmol), **2h** (34 mg, 0.25 mmol) and **3a** (0.5 mmol) for 3 h. Purification by column chromatography (*n*-hexane/EtOAc 20:1) yielded **27** (78 mg, 93%) as an oil. ^{1}H-NMR (CDCl$_3$, 400 MHz): δ = 7.34 – 7.18 (m, 6H), 6.87 (d, J = 7.8 Hz, 1H), 6.81 (t, J = 2.0 Hz, 1H), 6.75 (dd, J = 8.2, 2.0 Hz, 1H), 4.27 (t, J = 7.3 Hz, 1H), 3.89 (q, J = 7.2 Hz, 2H), 3.79 (s, 3H), 2.94 (td, J = 15.4, 7.3 Hz, 2H), 1.20 (t, J = 7.2 Hz, 3H). ^{13}C-NMR (CDCl$_3$, 100 MHz): δ = 163.8 (t, J_{C-F} = 32.6 Hz), 159.7, 144.6, 142.8, 129.6, 128.6, 127.7, 126.8, 120.0, 115.6 (t, J_{C-F} = 250.7 Hz), 113.9, 111.7, 62.7, 55.2, 44.8 (t, J_{C-F} = 4.7 Hz), 40.2 (t, J_{C-F} = 23.3 Hz), 13.7. ^{19}F-NMR (CDCl$_3$, 376 MHz): δ = -103.49 (dt, J = 27.0, 15.4 Hz). IR (ATR): 2996, 1726, 1574, 1434, 1224, 1128, 1092 cm^{-1}. HR-MS (ESI) m/z calcd for $C_{19}H_{21}F_2O_3$ [M+H$^+$] 335.1459, found 335.1464 [M+H$^+$].

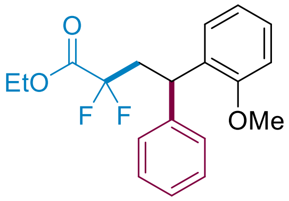

Ethyl 2,2-difluoro-4-(2-methoxyphenyl)-4-phenylbutanoate (28)

The general procedure **TP2** was followed using **1a** (101 mg, 0.5 mmol), **2i** (34 mg, 0.25 mmol) and **3a** (0.5 mmol) for 3 h. Purification by column chromatography (*n*-hexane/EtOAc 20:1) yielded **28** (52 mg, 62%) as an oil. ^{1}H-NMR (CDCl$_3$, 400 MHz): δ =7.34 – 7.25 (m, 4H), 7.20 (tdt, J = 7.5, 5.8, 1.7 Hz, 3H), 6.93 (td, J = 7.5, 1.1 Hz, 1H), 6.85 (d, J = 8.1 Hz, 1H), 4.74 (t, J = 7.3 Hz, 1H), 3.87 (q, J = 7.2 Hz, 2H), 3.82 (s, 3H), 2.94 (td, J = 15.6, 7.3 Hz, 2H), 1.20 (t, J = 7.2 Hz, 3H). ^{13}C-NMR (CDCl$_3$, 100 MHz): δ = 164.0 (t, J_{C-F} = 32.6 Hz), 156.7, 142.6, 131.4, 128.2, 128.1, 128.0, 127.9, 126.4, 120.5, 115.9 (t, J_{C-F} = 250.6 Hz), 110.9, 62.6, 55.4, 39.3 (t, J_{C-F} = 23.2 Hz), 37.9 (t, J_{C-F} = 5.0 Hz), 13.7. ^{19}F-NMR (CDCl$_3$, 376 MHz): δ = -102.53 (t, J = 15.3 Hz), -103.21 (t, J = 15.3 Hz), -103.78 (t, J = 15.7 Hz), -104.47 (t, J = 15.7 Hz). IR (ATR): 2998, 1718, 1576, 1451, 1225, 1138, 1082 cm^{-1}. HR-MS (ESI) m/z calcd for $C_{19}H_{21}F_2O_3$ [M+H$^+$] 335.1459, found 335.1466 [M+H$^+$].

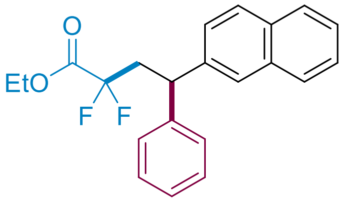

Ethyl 2,2-difluoro-4-(naphthalen-2-yl)-4-phenylbutanoate (29)

The general procedure **TP2** was followed using **1a** (101 mg, 0.5 mmol), **2j** (39 mg, 0.25 mmol) and **3a** ($Ph_2Zn \cdot 2Mg(OPiv)Cl$; 0.5 mmol) for 3 h. Purification by column chromatography (*n*-hexane/EtOAc 20:1) yielded **29** (50 mg, 56%) as an oil. ^{1}H-NMR ($CDCl_3$, 400 MHz): δ = 7.86 – 7.72 (m, 4H), 7.52 – 7.44 (m, 2H), 7.37 (dd, *J* = 8.5, 1.9 Hz, 1H), 7.35 – 7.29 (m, 4H), 7.22 (ddd, *J* = 8.6, 5.5, 2.6 Hz, 1H), 4.48 (t, *J* = 7.3 Hz, 1H), 3.84 – 3.69 (m, 2H), 3.18 – 2.96 (m, 2H), 1.11 (t, *J* = 7.2 Hz, 3H). ^{13}C-NMR ($CDCl_3$, 100 MHz): δ = 163.8 (t, J_{C-F} = 32.6 Hz), 142.8, 140.3, 133.4, 132.3, 128.7, 128.4, 127.9, 127.8, 127.6, 126.9, 126.3, 126.3, 125.9, 125.9, 115.7 (t, J_{C-F} = 250.8 Hz), 62.7, 44.9 (t, J_{C-F} = 4.8 Hz), 40.1 (t, J_{C-F} = 23.3 Hz), 13.6. ^{19}F-NMR ($CDCl_3$, 376 MHz): δ = -102.60 (t, *J* = 15.3 Hz), -103.31 (q, *J* = 15.5 Hz), -104.03 (t, *J* = 15.4 Hz). IR (ATR): 3332, 2971, 1668, 1428, 1234, 842 cm^{-1}. HR-MS (ESI) m/z calcd for $C_{22}H_{21}F_2O_2$ [M+H$^+$] 355.1510, found 355.1517 [M+H$^+$].

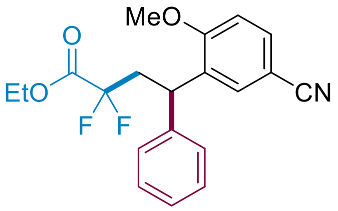

Ethyl 4-(5-cyano-2-methoxyphenyl)-2,2-difluoro-4-phenylbutanoate (30)

The general procedure **TP2** was followed using **1a** (101 mg, 0.5 mmol), **2k** (40 mg, 0.25 mmol) and **3a** (0.5 mmol) for 3 h. Purification by column chromatography (*n*-hexane/EtOAc 20:1→10:1) yielded **30** (49 mg, 55%) as an oil. ^{1}H-NMR ($CDCl_3$, 400 MHz): δ = 7.53 (dd, *J* = 8.5, 2.1 Hz, 1H), 7.48 (d, *J* = 2.0 Hz, 1H), 7.36 – 7.29 (m, 2H), 7.28 – 7.21 (m, 3H), 6.90 (d, *J* = 8.5 Hz, 1H), 4.74 (t, *J* = 7.3 Hz, 1H), 3.97 (q, *J* = 7.2 Hz, 2H), 3.90 (s, 3H), 3.01 – 2.77 (m, 2H), 1.24 (t, *J* = 7.2 Hz, 3H). ^{13}C-NMR ($CDCl_3$, 100 MHz): δ = 163.7 (t, J_{C-F} = 32.6 Hz), 160.0, 141.0, 133.2, 132.7, 131.8, 128.6, 128.0, 127.1, 119.2, 115.4 (t, J_{C-F} = 251.6 Hz), 111.3, 103.9, 62.8, 55.9, 38.9 (t, J_{C-F} = 23.4 Hz), 37.5 (t, J_{C-F} = 5.2 Hz), 13.8. ^{19}F-

NMR ($CDCl_3$, 376 MHz): δ = -102.26 (t, J = 15.1 Hz), -102.96 (t, J = 15.1 Hz), -104.81 (dd, J = 17.9, 15.3 Hz), -105.50 (dd, J = 17.9, 15.3 Hz). IR (ATR): 2979, 1682, 1476, 1263, 1131 cm^{-1}. HR-MS (ESI) m/z calcd for $C_{20}H_{20}F_2NO_3$ [M+H$^+$] 360.1411, found 360.1416 [M+H$^+$].

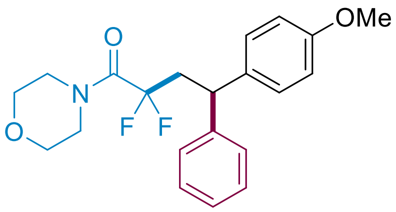

2,2-Difluoro-4-(4-methoxyphenyl)-1-morpholino-4-phenylbutan-1-one (**31**)

The general procedure **TP2** was followed using **1b** (130 mg, 0.5 mmol), **2a** (34 mg, 0.25 mmol) and **3a** (0.5 mmol) for 3 h. Purification by column chromatography (*n*-hexane/EtOAc 10:1) yielded **31** (92 mg, 98%) as an oil. ^{1}H-NMR ($CDCl_3$, 400 MHz): δ = 7.35 – 7.26 (m, 4H), 7.26 – 7.17 (m, 3H), 6.89 – 6.82 (m, 2H), 4.40 (t, J = 6.9 Hz, 1H), 3.79 (s, 3H), 3.63 (qd, J = 9.6, 4.5 Hz, 8H), 3.01 (td, J = 18.2, 6.9 Hz, 2H). ^{13}C-NMR ($CDCl_3$, 100 MHz): δ = 161.9 (t, J_{C-F} = 29.2 Hz), 158.1, 144.5, 136.3, 128.6, 128.6, 127.6, 126.4, 119.0 (t, J_{C-F} = 255.5 Hz), 113.9, 66.7, 66.7, 55.2, 46.5 (t, J_{C-F} = 6.3 Hz), 43.8 (t, J_{C-F} = 3.4 Hz), 43.3, 40.4 (t, J_{C-F} = 21.8 Hz). ^{19}F-NMR ($CDCl_3$, 376 MHz,): δ = -97.80 (t, J = 18.2 Hz). IR (ATR): 3333, 2973, 1674, 1436, 1262, 819 cm^{-1}. HR-MS (ESI) m/z calcd for $C_{21}H_{24}F_2NO_3$ [M+H$^+$] 376.1724, found 376.1729 [M+H$^+$].

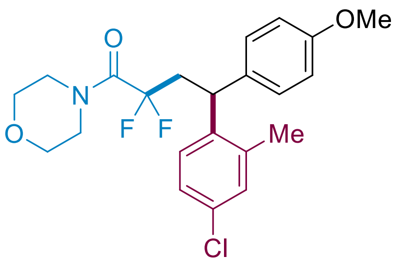

4-(4-Chloro-2-methylphenyl)-2,2-difluoro-4-(4-methoxyphenyl)-1-morpholinobutan-1-one (**32**)

The general procedure **TP2** was followed using **1b** (130 mg, 0.5 mmol), **2a** (34 mg, 0.25 mmol) and **3n** (0.5 mmol) for 3 h. Purification by column chromatography (*n*-hexane/EtOAc 10:1) yielded **32** (68 mg, 67%) as an oil. ^{1}H-NMR ($CDCl_3$, 400 MHz): δ = 7.25 (d, J = 8.4 Hz, 1H), 7.21 – 7.11 (m, 4H), 6.86 – 6.80 (m, 2H), 4.59 (t, J = 6.9 Hz, 1H),

3.78 (s, 3H), 3.75 – 3.56 (m, 8H), 3.02 – 2.87 (m, 2H), 2.33 (s, 3H). ^{13}C-NMR ($CDCl_3$, 100 MHz): δ = 161.9 (t, J_{C-F} = 29.2 Hz), 158.1, 140.8, 137.7, 135.1, 131.7, 130.5, 128.9, 128.1, 126.1, 118.9 (t, J_{C-F} = 255.8 Hz), 113.9, 66.7, 66.7, 55.2, 46.5 (t, J_{C-F} = 6.3 Hz), 43.4, 40.3 (t, J_{C-F} = 21.7 Hz), 38.6 (t, J_{C-F} = 3.3 Hz), 19.7. ^{19}F-NMR ($CDCl_3$, 376 MHz): δ = -98.01 (dd, J = 36.7, 17.3 Hz). IR (ATR): 3329, 2976, 1674, 1467, 1274, 1072 cm^{-1}. HR-MS (ESI) m/z calcd for $C_{22}H_{25}ClF_2NO_3$ [M+H$^+$] 424.1491, found 424.1494 [M+H$^+$].

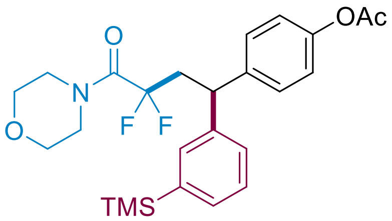

4-{3,3-Difluoro-4-morpholino-4-oxo-1-[3-(trimethylsilyl)phenyl]butyl}phenyl acetate (**33**)

The general procedure **TP2** was followed using **1b** (130 mg, 0.5 mmol), **21** (41 mg, 0.25 mmol) and **3j** (0.5 mmol) for 3 h. Purification by column chromatography (*n*-hexane/EtOAc 10:1→5:1) yielded **33** (62 mg, 52%) as an oil. ^{1}H-NMR ($CDCl_3$, 400 MHz): δ = 7.42 (s, 1H), 7.40 – 7.36 (m, 1H), 7.34 – 7.25 (m, 4H), 7.06 – 7.00 (m, 2H), 4.44 (t, J = 6.9 Hz, 1H), 3.71 – 3.66 (m, 2H), 3.64 (s, 4H), 3.60 – 3.54 (m, 2H), 3.10 – 2.96 (m, 2H), 2.30 (s, 3H), 0.27 (s, 9H). ^{13}C-NMR ($CDCl_3$, 100 MHz): δ = 169.6, 161.8 (t, J_{C-F} = 29.2 Hz), 149.1, 142.8, 141.7, 141.0, 132.7, 131.7, 128.7, 128.0, 128.0, 121.6, 118.9 (t, J = 255.6 Hz), 66.7, 46.4 (t, J_{C-F} = 6.2 Hz), 44.2 (d, J_{C-F} = 3.4 Hz), 43.3, 40.4 (t, J_{C-F} = 21.9 Hz), 21.2, -1.1. ^{19}F-NMR ($CDCl_3$, 376 MHz): δ = -97.71 (t, J = 18.6 Hz). IR (ATR): 2988, 1674, 1482, 1267, 1135 cm^{-1}. HR-MS (ESI) m/z calcd for $C_{25}H_{32}F_2NO_4Si$ [M+H$^+$] 476.2069, found 476.2072 [M+H$^+$].

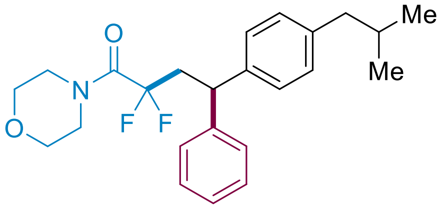

2,2-Difluoro-4-(4-isobutylphenyl)-1-morpholino-4-phenylbutan-1-one (**34**)

The general procedure **TP2** was followed using **1b** (130 mg, 0.5 mmol), **2m** (40 mg, 0.25 mmol) and **3a** (0.5 mmol) for 3 h. Purification by column chromatography (*n*-hexane/EtOAc 10:1) yielded **34** (83 mg, 83%) as an oil. ^{1}H-NMR ($CDCl_3$, 400 MHz): δ = 7.26 (t, J = 5.1 Hz, 4H), 7.21 – 7.13 (m, 3H), 7.04 (d, J = 8.1 Hz, 2H), 4.37 (t, J = 6.9 Hz, 1H), 3.59 (qd, J = 9.5, 4.5 Hz, 8H), 2.99 (td, J = 18.2, 6.9 Hz, 2H), 2.40 (d, J = 7.2 Hz, 2H), 1.81 (dp, J = 13.6, 6.7 Hz, 1H), 0.86 (d, J = 6.6 Hz, 6H). ^{13}C-NMR ($CDCl_3$, 100 MHz): δ = 161.9 (t, J_{C-F} = 29.3 Hz), 144.3, 141.3, 139.8, 129.2, 128.5, 127.7, 127.3, 126.3, 119.0 (t, J_{C-F} = 255.2 Hz), 66.6, 66.6, 46.4 (t, J_{C-F} = 6.4 Hz), 45.0, 44.2 (t, J_{C-F} = 3.5 Hz), 43.3, 40.3 (t, J_{C-F} = 21.9 Hz), 30.1, 22.4. ^{19}F-NMR ($CDCl_3$, 376 MHz): δ = -97.82 (q, J = 18.8 Hz). IR (ATR): 3432, 2987, 1684, 1446, 1277, 843 cm^{-1}. HR-MS (ESI) m/z calcd for $C_{24}H_{30}F_2NO_2$ [M+H$^+$] 402.2245, found 402.2248 [M+H$^+$].

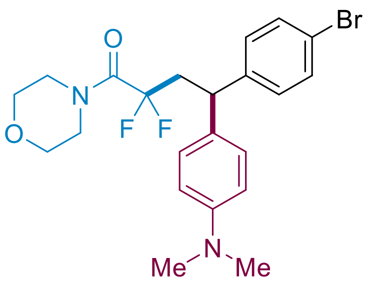

4-(4-Bromophenyl)-4-[4-(dimethylamino)phenyl]-2,2-difluoro-1-morpholinobutan-1-one (35)

The general procedure **TP2** was followed using **1b** (130 mg, 0.5 mmol), **2f** (46 mg, 0.25 mmol) and **3s** (0.5 mmol) for 3 h. Purification by column chromatography (*n*-hexane/EtOAc 10:1) yielded **35** (47 mg, 40%) as an oil. ^{1}H-NMR ($CDCl_3$, 400 MHz): δ = 7.40 (d, J = 8.5 Hz, 2H), 7.16 (d, J = 8.4 Hz, 2H), 7.12 (d, J = 8.7 Hz, 2H), 6.69 (d, J = 8.6 Hz, 2H), 4.31 (t, J = 6.9 Hz, 1H), 3.74 – 3.67 (m, 2H), 3.65 (s, 4H), 3.61 – 3.55 (m, 2H), 3.06 – 2.89 (m, 2H), 2.91 (s, 6H). ^{13}C-NMR ($CDCl_3$, 100 MHz): δ = 161.9 (d, J_{C-F} = 29.2 Hz), 149.3, 144.0, 131.5, 131.3 (s), 129.4, 128.2, 120.0, 119.0 (t, J_{C-F} = 255.0 Hz), 112.7, 66.7, 66.7, 46.5 (d, J_{C-F} = 6.2 Hz), 43.3, 43.1 (t, J_{C-F} = 3.6 Hz), 40.7, 40.1 (t, J_{C-F} = 21.8 Hz), 38.5. ^{19}F-NMR ($CDCl_3$, 376 MHz): δ = -97.07 (t, J = 18.6 Hz), -97.85 (dt, J = 28.1, 18.1 Hz), -98.63 (t, J = 18.3 Hz). IR (ATR): 3017, 2939, 1656, 1478, 1266, 1132 cm^{-1}. HR-MS (ESI) m/z calcd for $C_{22}H_{26}BrF_2N_2O_2$ [M+H$^+$] 467.1146, found 467.1149 [M+H$^+$].

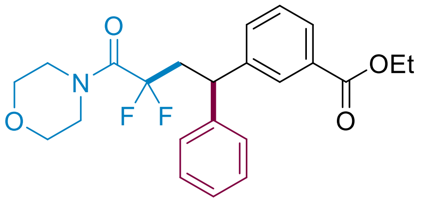

Ethyl 3-(3,3-difluoro-4-morpholino-4-oxo-1-phenylbutyl)benzoate (36)

The general procedure **TP2** was followed using **1b** (130 mg, 0.5 mmol), **2n** (44 mg, 0.25 mmol) and **3a** (0.5 mmol) for 3 h. Purification by column chromatography (*n*-hexane/EtOAc 10:1→5:1) yielded **36** (68 mg, 65%) as an oil. ^{1}H-NMR ($CDCl_3$, 400 MHz): δ = 8.02 (t, J = 1.6 Hz, 1H), 7.90 (dt, J = 7.7, 1.3 Hz, 1H), 7.53 – 7.47 (m, 1H), 7.38 (t, J = 7.7 Hz, 1H), 7.35 – 7.29 (m, 4H), 7.26 – 7.17 (m, 1H), 4.51 (t, J = 7.0 Hz, 1H), 4.39 (q, J = 7.1 Hz, 2H), 3.75 – 3.62 (m, 6H), 3.62 – 3.54 (m, 2H), 3.16 – 2.98 (m, 2H), 1.41 (t, J = 7.1 Hz, 3H). ^{13}C-NMR ($CDCl_3$, 100 MHz): δ = 166.6, 161.8 (t, J_{C-F} = 29.2 Hz), 144.4, 143.6, 132.3, 130.8, 128.7, 128.7, 128.7, 127.8, 127.6, 126.7, 118.9 (t, J_{C-F} = 255.9 Hz), 66.7, 66.7, 61.1, 46.4 (t, J_{C-F} = 6.2 Hz), 44.5 (t, J_{C-F} = 3.3 Hz), 43.3, 39.9 (t, J_{C-F} = 21.8 Hz), 14.4. ^{19}F-NMR ($CDCl_3$, 376 MHz): δ = -96.94 – -97.11 (m), -97.78 (t, J = 18.1 Hz), -97.94 (t, J = 18.2 Hz), -98.62 – -98.77 (m). IR (ATR): 2989, 1657, 1514, 1267, 1086 cm^{-1}. HR-MS (ESI) m/z calcd for $C_{23}H_{26}F_2NO_4$ [M+H$^+$] 418.1830, found 418.1837 [M+H$^+$].

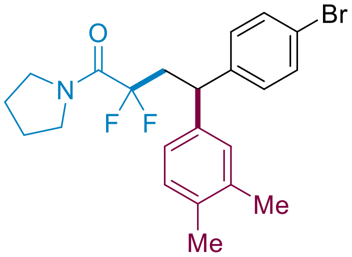

4-(4-Bromophenyl)-4-(3,4-dimethylphenyl)-2,2-difluoro-1-(pyrrolidin-1-yl)butan-1-one (37)

The general procedure **TP2** was followed using **1c** (122 mg, 0.5 mmol), **2f** (46 mg, 0.25 mmol) and **3t** (0.5 mmol) for 3 h. Purification by column chromatography (*n*-hexane/EtOAc 10:1) yielded **37** (78 mg, 72%) as an oil. ^{1}H-NMR ($CDCl_3$, 400 MHz): δ = 7.44 – 7.38 (m, 2H), 7.22 – 7.14 (m, 2H), 7.07 (d, J = 7.7 Hz, 1H), 7.00 (dd, J = 11.4, 3.7 Hz, 2H), 4.31 (t, J = 7.2 Hz, 1H), 3.54 (t, J = 6.7 Hz, 2H), 3.38 (q, J = 7.2 Hz, 2H), 2.98 (td, J =

17.2, 7.2 Hz, 2H), 2.23 (d, J = 7.4 Hz, 6H), 1.92 – 1.82 (m, 2H), 1.82 – 1.72 (m, 2H). ^{13}C-NMR ($CDCl_3$, 100 MHz): δ = 162.0 (t, J_{C-F} = 29.6 Hz), 143.3, 140.8, 136.8, 135.0, 131.5, 129.9, 129.5, 128.9, 124.8, 120.2, 118.5 (t, J_{C-F} = 254.3 Hz), 47.5, 46.5 (t, J_{C-F} = 6.5 Hz), 43.9 (t, J_{C-F} = 3.9 Hz), 39.7 (t, J_{C-F} = 22.5 Hz), 27.1, 26.5, 23.2, 19.9, 19.4. ^{19}F-NMR ($CDCl_3$, 376 MHz): δ = -100.86 (td, J = 17.1, 8.9 Hz). IR (ATR): 2986, 1765, 1497, 1276, 1046 cm^{-1}. HR-MS (ESI) m/z calcd for $C_{22}H_{25}BrF_2NO$ [M+H$^+$] 369.0705, found 436.1088 [M+H$^+$], 436.1091 [M+H$^+$].

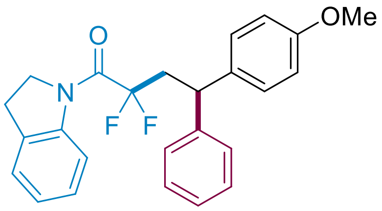

2,2-Difluoro-1-(indolin-1-yl)-4-(4-methoxyphenyl)-4-phenylbutan-1-one (38)

The general procedure **TP2** was followed using **1d** (138 mg, 0.5 mmol), **2a** (34 mg, 0.25 mmol) and **3a** (0.5 mmol) for 3 h. Purification by column chromatography (*n*-hexane/EtOAc 10:1) yielded **38** (92 mg, 90%) as an oil. ^{1}H-NMR ($CDCl_3$, 400 MHz): δ = 8.19 (d, J = 8.0 Hz, 1H), 7.36 – 7.16 (m, 9H), 7.16 – 7.07 (m, 1H), 6.87 – 6.79 (m, 2H), 4.46 (t, J = 7.1 Hz, 1H), 4.22 (t, J = 8.3 Hz, 2H), 3.75 (s, 3H), 3.20 – 3.03 (m, 4H). ^{13}C-NMR ($CDCl_3$, 100 MHz): δ = 161.4 (t, J_{C-F} = 30.1 Hz), 158.1, 144.4, 142.6, 136.0, 131.7, 128.7, 128.6, 127.6, 127.5, 126.4, 125.0, 124.6, 118.8 (t, J_{C-F} = 256.1 Hz), 118.0, 113.9, 55.2, 47.9 (t, J_{C-F} = 7.8 Hz), 44.0 (t, J_{C-F} = 3.6 Hz), 40.0 (t, J_{C-F} = 22.1 Hz), 28.6. ^{19}F-NMR ($CDCl_3$, 376 MHz): δ = -100.10 (t, J = 17.5 Hz), -100.84 (t, J = 17.5 Hz), -100.96 (t, J = 17.8 Hz), -101.70 (t, J = 17.7 Hz). IR (ATR): 3032, 2987, 1663, 1459, 1261, 1134 cm^{-1}. HR-MS (ESI) m/z calcd for $C_{25}H_{24}F_2NO_2$ [M+H$^+$] 408.1775, found 408.1777 [M+H$^+$].

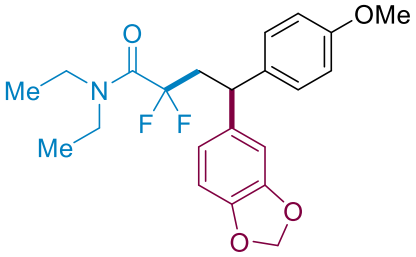

4-(Benzo[*d*][1,3]dioxol-5-yl)-*N*,*N*-diethyl-2,2-difluoro-4-(4-methoxyphenyl)butanamide (39)

The general procedure **TP2** was followed using **1e** (115 mg, 0.5 mmol), **2a** (34 mg, 0.25 mmol) and **3h** (0.5 mmol) for 3 h. Purification by column chromatography (*n*-hexane/EtOAc 10:1) yielded **39** (76 mg, 75%) as an oil. ^{1}H-NMR ($CDCl_3$, 400 MHz): δ = 7.26 – 7.16 (m, 2H), 6.90 – 6.80 (m, 2H), 6.80 – 6.70 (m, 3H), 5.92 (q, J = 1.4 Hz, 2H), 4.32 (t, J = 7.0 Hz, 1H), 3.79 (s, 3H), 3.45 (q, J = 7.0 Hz, 2H), 3.35 (q, J = 7.1 Hz, 2H), 2.95 (tdd, J = 18.1, 7.0, 2.2 Hz, 2H), 1.2 (t, J = 7.1 Hz, 3H), 1.1 (t, J = 7.1 Hz, 3H). ^{13}C-NMR ($CDCl_3$, 100 MHz): δ = 162.7 (t, J_{C-F} = 29.0 Hz), 158.0, 147.7, 145.9, 138.8, 136.5, 128.5, 120.5, 119.1 (d, J_{C-F} = 256.0 Hz), 113.9, 108.2, 108.2, 100.9, 55.2, 43.6 (t, J_{C-F} = 3.5 Hz), 41.9 (t, J_{C-F} = 6.3 Hz), 41.6, 40.5 (t, J_{C-F} = 22.0 Hz), 14.3, 12.3. ^{19}F-NMR ($CDCl_3$, 376 MHz): δ = -98.57 (t, J = 18.1 Hz). IR (ATR): 3332, 2923, 1683, 1468, 1238, 1045 cm^{-1}. HR-MS (ESI) m/z calcd for $C_{22}H_{26}F_2NO_4$ [M+H$^+$] 406.1830, found 406.1834 [M+H$^+$].

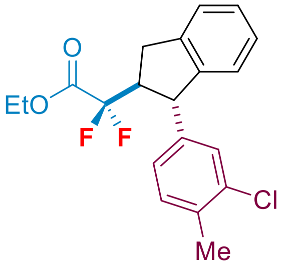

Ethyl 2-[1-(3-chloro-4-methylphenyl)-2,3-dihydro-1*H*-inden-2-yl]-2,2-difluoroacetate (40)

The general procedure **TP2** was followed using **1a** (101 mg, 0.5 mmol), **2o** (29 mg, 0.25 mmol) and **3u** (0.5 mmol) for 3 h. Purification by column chromatography (*n*-hexane/EtOAc 20:1) yielded **40** (50 mg, 55%; *dr* > 20:1) as an oil. ^{1}H-NMR ($CDCl_3$, 400 MHz): δ = 7.32 – 7.21 (m, 2H), 7.21 – 7.12 (m, 3H), 6.98 (dd, J = 7.8, 1.7 Hz, 1H), 6.84 (d, J = 7.5 Hz, 1H), 4.53 – 4.45 (m, 1H), 4.07 (dq, J = 10.7, 7.2 Hz, 1H), 3.97 (dq, J = 10.7, 7.1 Hz, 1H), 3.35 – 3.21 (m, 3H), 2.38 (s, 3H), 1.19 (t, J = 7.2 Hz, 3H). ^{13}C-NMR ($CDCl_3$, 100 MHz): δ = 162.6 (t, J_{C-F} = 32.8 Hz), 143.5, 141.6, 139.4, 133.6, 133.4, 130.0, 127.9, 126.5, 126.2, 125.9, 124.0, 123.3, 115.2 (t, J_{C-F} = 252.5 Hz), 61.8, 51.5 (t, J_{C-F} = 22.3 Hz), 49.6 (dd, J_{C-F} = 5.7, 2.5 Hz), 30.72 (t, J_{C-F} = 3.9 Hz), 28.7, 18.6, 12.6. ^{19}F-NMR ($CDCl_3$, 376 MHz): δ = -

108.42 (d, J = 255.0 Hz), -112.60 (d, J = 255.0 Hz). IR (ATR): 3319, 2979, 1682, 1434, 1285, 842 cm^{-1}. HR-MS (ESI) m/z calcd for $C_{20}H_{20}ClF_2O_2$ [M+H$^+$] 365.1120, found 365.1126 [M+H$^+$].

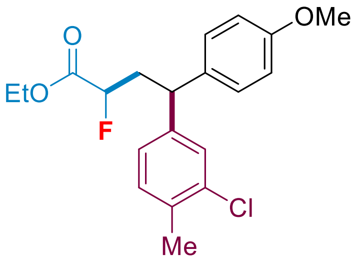

Ethyl -4-(3-chloro-4-methylphenyl)-2-fluoro-4-(4-methoxyphenyl)butanoate (**41**)

The general procedure **TP2** was followed using **1f** (92 mg, 0.5 mmol), **2a** (34 mg, 0.25 mmol) and **3u** (0.5 mmol) for 3 h. Purification by column chromatography (*n*-hexane/EtOAc 20:1) yielded **41** (31 mg, 34%; *dr* = 1:1) as an oil. ^{1}H-NMR ($CDCl_3$, 400 MHz): δ = 7.25 (d, J = 1.7 Hz, 1H), 7.23 (d, J = 1.6 Hz, 1H), 7.22 – 7.12 (m, 6H), 7.08 (dd, J = 7.8, 1.7 Hz, 1H), 7.04 (dd, J = 7.9, 1.7 Hz, 1H), 6.91 – 6.87 (m, 2H), 6.87 – 6.82 (m, 2H), 4.77 (dt, J = 8.2, 3.9 Hz, 1H), 4.69 – 4.61 (m, 1H), 4.28 – 4.15 (m, 6H), 3.80 (s, 3H), 3.79 (s, 3H), 2.67 – 2.47 (m, 4H), 2.34 (s, 3H), 2.33 (s, 3H), 1.33 – 1.29 (m, 6H). ^{13}C-NMR ($CDCl_3$, 100 MHz): δ = 169.9 (d, J_{C-F} = 23.3 Hz), 169.9 (d, J_{C-F} = 23.3 Hz), 158.5, 158.3, 143.4, 142.2, 135.3, 134.7, 134.4, 134.1, 133.9, 131.3, 131.0, 129.1, 128.6, 128.0, 126.3, 125.9, 114.3, 114.1, 88.0 (d, J_{C-F} = 184.6 Hz), 87.0 (d, J_{C-F} = 184.6 Hz), 61.6, 55.3, 44.7 (d, J_{C-F} = 2.6 Hz), 38.3 (d, J_{C-F} = 21.0 Hz), 19.6, 19.6, 14.1. ^{19}F-NMR ($CDCl_3$, 376 MHz): δ = -193.39 (s), -193.79 (s). IR (ATR): 3031, 2929, 1642, 1464, 1281, 841 cm^{-1}. HR-MS (ESI) m/z calcd for $C_{20}H_{23}ClFO_3$ [M+H$^+$] 365.1320, found 365.1324 [M+H$^+$].

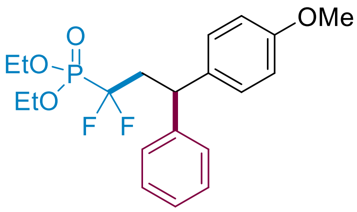

Diethyl 1,1-difluoro-3-(4-methoxyphenyl)-3-phenylpropylphosphonate (**42**)

The general procedure **TP2** was followed using **1g** (133 mg, 0.5 mmol), **2a** (34 mg, 0.25 mmol) and **3a** (0.5 mmol) for 3 h. Purification by column chromatography (*n*-

hexane/EtOAc 10:1→5:1) yielded **42** (97 mg, 97%) as an oil. ^{1}H-NMR ($CDCl_3$, 400 MHz): δ = 7.35 – 7.26 (m, 4H), 7.25 – 7.16 (m, 3H), 6.88 – 6.82 (m, 2H), 4.50 (t, J = 7.0 Hz, 1H), 4.33 – 4.18 (m, 4H), 3.78 (s, 3H), 2.90 (tdd, J = 19.8, 7.0, 4.4 Hz, 2H), 1.37 (t, J = 7.1 Hz, 6H). ^{13}C-NMR ($CDCl_3$, 100 MHz): δ = 158.1, 144.4, 136.2, 128.6, 128.6, 127.6, 126.4, 120.5 (td, J_{C-F} = 261.5, 215.4 Hz), 113.9, 64.6, 64.5, 55.2, 43.0 (dt, J_{C-F} = 3.6 Hz), 39.3 (td, J_{C-F} = 19.6, 14.6 Hz), 16.4, 16.4. ^{19}F-NMR ($CDCl_3$, 376 MHz): δ = -110.75 (td, J = 19.3, 3.7 Hz), -111.03 (td, J = 19.3, 3.6 Hz). IR (ATR): 3337, 2919, 1642, 1454, 1281, 841 cm^{-1}. HR-MS (ESI) m/z calcd for $C_{20}H_{26}F_2O_4P$ [M+H$^+$] 399.1537, found 399.1532 [M+H$^+$].

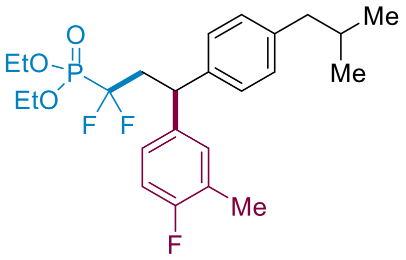

Diethyl 1,1-difluoro-3-(4-fluoro-3-methylphenyl)-3-(4-isobutylphenyl)propylphosphonate (43)

The general procedure **TP2** was followed using **1g** (133 mg, 0.5 mmol), **2m** (40 mg, 0.25 mmol) and **3l** (0.5 mmol) for 3 h. Purification by column chromatography (*n*-hexane/EtOAc 10:1→5:1) yielded **43** (60 mg, 53%) as an oil. ^{1}H-NMR ($CDCl_3$, 400 MHz): δ = 7.15 – 7.09 (m, 2H), 7.08 – 6.99 (m, 4H), 6.93 – 6.83 (m, 1H), 4.40 (t, J = 7.0 Hz, 1H), 4.31 – 4.14 (m, 4H), 2.93 – 2.73 (m, 2H), 2.39 (d, J = 7.2 Hz, 2H), 2.21 (d, J = 1.8 Hz, 3H), 1.80 (dp, J = 13.7, 6.8 Hz, 1H), 1.33 (t, J = 7.1 Hz, 6H), 0.86 (d, J = 6.6 Hz, 6H). ^{13}C-NMR ($CDCl_3$, 100 MHz): δ = 159.9 (d, J_{C-F} = 243.4 Hz), 141.2, 139.8, 139.5 (d, J_{C-F} = 3.6 Hz), 130.6 (d, J_{C-F} = 5.0 Hz), 129.3, 127.1, 126.2 (d, J_{C-F} = 7.9 Hz), 124.6 (d, J_{C-F} = 17.3 Hz), 120.4 (td, J_{C-F} = 260.4, 215.4 Hz), 114.8 (d, J_{C-F} = 22.3 Hz), 64.6 (d, J_{C-F} = 2.0 Hz), 64.5 (d, J_{C-F} = 2.2 Hz), 44.9, 42.7 (dt, J_{C-F} = 3.6 Hz), 39.4 (td, J_{C-F} = 19.6, 14.5 Hz), 30.1, 22.3, 16.4, 16.3, 14.6 (d, J_{C-F} = 3.5 Hz). ^{19}F-NMR ($CDCl_3$, 376 MHz): δ = -110.04 (ddd, J = 107.6, 22.9, 16.5 Hz), -110.55 – -111.32 (m), -111.85 (ddd, J = 107.4, 22.8, 16.6 Hz), -121.11 – -121.34 (m). IR (ATR): 3332, 2927, 1672, 1454, 1265, 842 cm^{-1}. HR-MS (ESI) m/z calcd for $C_{24}H_{33}F_3N_3P$ [M+H$^+$] 457.2119, found 457.2114 [M+H$^+$].

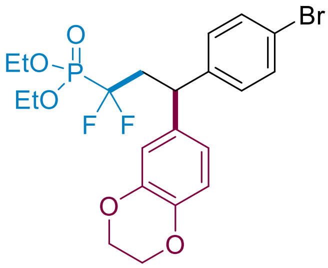

Diethyl 3-(4-bromophenyl)-3-(2,3-dihydrobenzo[*b*][1,4]dioxin-6-yl)-1,1-difluoropropylphosphonate (**44**)

The general procedure **TP2** was followed using **1g** (133 mg, 0.5 mmol), **2f** (46 mg, 0.25 mmol) and **3i** (0.5 mmol) for 3 h. Purification by column chromatography (*n*-hexane/EtOAc 10:1→5:1) yielded **44** (84 mg, 67%) as an oil. ^{1}H-NMR ($CDCl_3$, 400 MHz): δ = 7.44 – 7.38 (m, 2H), 7.19 – 7.13 (m, 2H), 6.79 (d, J = 8.2 Hz, 1H), 6.73 (dt, J = 8.2, 2.1 Hz, 2H), 4.38 (t, J = 7.0 Hz, 1H), 4.29 – 4.20 (m, 8H), 2.92 – 2.73 (m, 2H), 1.37 (t, J = 7.1 Hz, 6H). ^{13}C-NMR ($CDCl_3$, 100 MHz): δ = 143.4, 143.1, 142.2, 136.8, 131.6, 129.3, 120.4, 120.3, 120.3 (td, $J_{C–F}$ = 260.9, 215.2 Hz), 117.4, 116.2, 64.7 (d, $J_{C–F}$ = 1.8 Hz), 64.6 (d, $J_{C–F}$ = 1.8 Hz), 64.4, 64.3, 42.8 – 42.5 (m), 39.0 (td, $J_{C–F}$ = 19.6, 14.6 Hz), 16.5, 16.4. ^{19}F-NMR ($CDCl_3$, 376 MHz): δ = -109.94 (dd, J = 25.0, 14.1 Hz), -110.22 (dd, J = 24.9, 14.1 Hz), -110.73 (dd, J = 20.6, 18.3 Hz), -110.94 – -111.10 (m), -111.23 – -111.38 (m), -111.81 (dd, J = 24.7, 14.5 Hz), -112.10 (dd, J = 24.7, 14.6 Hz). IR (ATR): 3329, 2931, 1657, 1458, 1263, 1141 cm^{-1}. HR-MS (ESI) m/z calcd for $C_{21}H_{25}BrF_2O_2P$ [M+H$^+$] 505.0591, found 505.0596 [M+H$^+$].

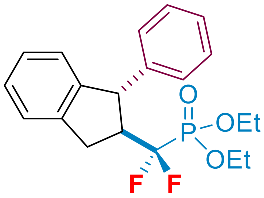

Diethyl [difluoro(1-phenyl-2,3-dihydro-1*H*-inden-2-yl)methyl]phosphonate (**45**)

The general procedure **TP2** was followed using **1g** (133 mg, 0.5 mmol), **2o** (29 mg, 0.25 mmol) and **3a** (0.5 mmol) for 3 h. Purification by column chromatography (*n*-hexane/EtOAc 10:1) yielded **45** (48.5 mg, 51%; *dr* > 20:1) as an oil. ^{1}H-NMR ($CDCl_3$, 400 MHz): δ = 7.35 – 7.18 (m, 7H), 7.14 (t, J = 7.3 Hz, 1H), 6.86 (d, J = 7.5 Hz, 1H), 4.79 (d, J = 7.3 Hz, 1H), 4.33 – 4.15 (m, 4H), 3.45 – 3.20 (m, 3H), 1.36 (td, J = 7.1, 2.5 Hz, 6H). ^{13}C-

NMR ($CDCl_3$, 100 MHz): δ = 145.2, 144.6, 141.0, 128.5, 128.5, 127.1, 127.0, 126.6, 125.2, 124.2, 121.6 (td, J_{C-F} = 261.8, 212.4 Hz), 64.5 (dd, J_{C-F} = 16.6, 7.0 Hz), 52.4 (td, J_{C-F} = 19.5, 14.5 Hz), 50.9 (p, J_{C-F} = 3.2 Hz), 32.7 (dd, J_{C-F} = 6.7, 3.6 Hz), 29.7, 16.4 (d, J_{C-P} = 2.1 Hz), 16.4 (d, J_{C-P} = 2.3 Hz). ^{19}F-NMR ($CDCl_3$, 376 MHz): δ = -109.92 (dd, J = 299.6, 108.7 Hz), -117.41 (dd, J = 299.6, 108.9 Hz). IR (ATR): 3331, 2919, 1642, 1474, 1258, 833 cm^{-1}. HR-MS (ESI) m/z calcd for $C_{20}H_{24}F_2O_3P$ [M+H$^+$] 381.1431, found 381.1432 [M+H$^+$].

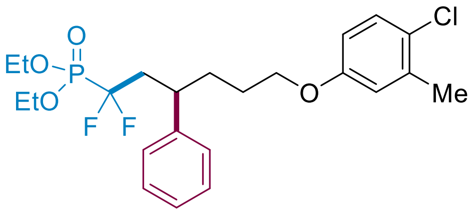

Diethyl [6-(4-chloro-3-methylphenoxy)-1,1-difluoro-3-phenylhexyl]phosphonate (**46**)

The general procedure **TP2** was followed using **1g** (133 mg, 0.5 mmol), **2p** (53 mg, 0.25 mmol) and **3a** (0.5 mmol) for 3 h. Purification by column chromatography (*n*-hexane/EtOAc 10:1) yielded **46** (40 mg, 34%) as an oil. ^{1}H-NMR ($CDCl_3$, 400 MHz): δ = 7.34 – 7.27 (m, 2H), 7.24 – 7.15 (m, 4H), 6.70 (d, J = 2.9 Hz, 1H), 6.59 (dd, J = 8.7, 3.0 Hz, 1H), 4.27 – 4.18 (m, 4H), 3.82 (t, J = 6.4 Hz, 2H), 3.14 (td, J = 11.2, 6.4 Hz, 1H), 2.50 – 2.36 (m, 2H), 2.31 (s, 3H), 1.98 (dtd, J = 15.4, 10.4, 5.2 Hz, 1H), 1.82 – 1.71 (m, 1H), 1.63 – 1.49 (m, 2H), 1.35 (td, J = 7.1, 3.4 Hz, 6H). ^{13}C-NMR ($CDCl_3$, 100 MHz): δ = 157.5, 144.2, 136.9, 129.5, 128.6, 127.5, 126.6, 125.6, 120.3 (td, J_{C-F} = 260.7, 254.5 Hz), 117.0, 113.1, 67.9, 64.5, 64.4, 40.4 (td, J_{C-F} = 19.5, 14.0 Hz), 38.5 (dt, J_{C-F} = 6.3, 3.3 Hz), 33.5, 27.1, 20.3, 16.4, 16.4. ^{19}F-NMR ($CDCl_3$, 376 MHz): δ = -110.67 (d, J = 108.2 Hz). IR (ATR): 3297, 2917, 1662, 1454, 1293, 792 cm^{-1}. HR-MS (ESI) m/z calcd for $C_{23}H_{31}ClF_2O_4P$ [M+H$^+$] 475.1617, found 475.1619 [M+H$^+$].

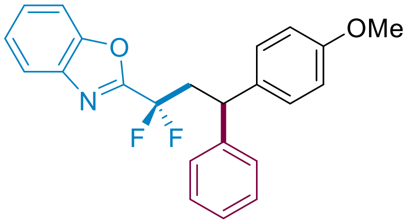

2-[1,1-Difluoro-3-(4-methoxyphenyl)-3-phenylpropyl]benzo[*d*]oxazole (**47**)

The general procedure **TP2** was followed using **1h** (124 mg, 0.5 mmol), **2a** (34 mg, 0.25 mmol) and **3a** (0.5 mmol) for 3 h. Purification by column chromatography (*n*-hexane/EtOAc 20:1) yielded **47** (52 mg, 55%) as an oil. ^{1}H-NMR ($CDCl_3$, 400 MHz): δ = 7.68 (dd, J = 6.7, 2.2 Hz, 1H), 7.42 (dd, J = 6.9, 2.0 Hz, 1H), 7.39 – 7.29 (m, 2H), 7.18 (ddd, J = 15.3, 10.9, 4.7 Hz, 4H), 7.12 – 7.06 (m, 2H), 7.06 – 6.99 (m, 1H), 6.67 – 6.57 (m, 2H), 4.36 (t, J = 7.3 Hz, 1H), 3.60 (s, 3H), 3.32 – 3.16 (m, 2H). ^{13}C-NMR ($CDCl_3$, 100 MHz): δ = 158.1, 157.6 (t, J_{C-F} = 33.8 Hz), 150.4, 143.3, 139.9, 134.8, 128.6, 128.5, 127.4, 126.5, 126.4, 125.0, 121.1, 116.5 (t, J_{C-F} = 243.3 Hz), 113.8, 111.2, 55.1, 44.2 (t, J_{C-F} = 4.1 Hz), 42.2 (t, J_{C-F} = 23.7 Hz). ^{19}F-NMR ($CDCl_3$, 376 MHz): δ = -96.80 (dd, J = 1041.9, 273.4 Hz). IR (ATR): 3032, 2918, 1692, 1474, 1281, 841 cm^{-1}. HR-MS (ESI) m/z calcd for $C_{23}H_{20}F_2NO_2$ [M+H$^+$] 380.1462, found 380.1468 [M+H$^+$].

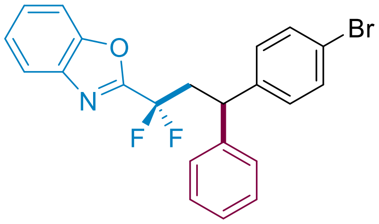

2-[1,1-Difluoro-3-(4-bromophenyl)-3-phenylpropyl]benzo[*d*]oxazole (48)

The general procedure **TP2** was followed using **1h** (124 mg, 0.5 mmol), **2f** (46 mg, 0.25 mmol) and **3a** (0.5 mmol) for 3 h. Purification by column chromatography (*n*-hexane/EtOAc 20:1) yielded **48** (55 mg, 51%) as an oil. ^{1}H-NMR ($CDCl_3$, 400 MHz): δ = 7.70 (dd, J = 6.9, 1.8 Hz, 1H), 7.45 (dd, J = 7.1, 1.8 Hz, 1H), 7.42 – 7.32 (m, 2H), 7.27 – 7.21 (m, 2H), 7.21 – 7.15 (m, 4H), 7.06 (dd, J = 8.4, 4.0 Hz, 3H), 4.38 (t, J = 7.3 Hz, 1H), 3.24 (td, J = 15.5, 7.4 Hz, 2H). ^{13}C-NMR ($CDCl_3$, 100 MHz): δ = 157.4 (t, J_{C-F} = 33.7 Hz), 150.3, 142.4, 141.8, 139.8, 131.5, 129.4, 128.6, 127.4, 126.8, 125.3, 121.1, 120.5, 116.3 (t, J_{C-F} = 243.5 Hz), 111.2, 44.5 (t, J_{C-F} = 4.0 Hz), 41.8 (t, J_{C-F} = 23.8 Hz). ^{19}F-NMR ($CDCl_3$, 376 MHz): δ = -95.88 (d, J = 274.2 Hz), -97.79 (d, J = 274.3 Hz). IR (ATR): 3019, 2917, 1682, 1454, 1278, 766 cm^{-1}. HR-MS (ESI) m/z calcd for $C_{22}H_{17}BrF_2NO$ [M+H$^+$] 428.0462, found 428.0469 [M+H$^+$].

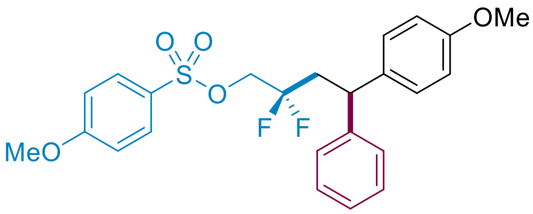

2,2-Difluoro-4-(4-methoxyphenyl)-4-phenylbutyl 4-methoxybenzenesulfonate (**49**)

The general procedure **TP2** was followed using **1i** (165 mg, 0.5 mmol), **2a** (34 mg, 0.25 mmol) and **3a** ($Ph_2Zn \cdot 2Mg(OPiv)Cl$; 0.5 mmol) for 3 h. Purification by column chromatography (*n*-hexane/EtOAc 40:1) yielded **49** (84 mg, 73%) as an oil. ^{1}H-NMR ($CDCl_3$, 400 MHz): δ = 7.79 – 7.74 (m, 2H), 7.30 – 7.23 (m, 2H), 7.22 – 7.17 (m, 3H), 7.14 – 7.10 (m, 2H), 7.01 – 6.96 (m, 2H), 6.83 – 6.78 (m, 2H), 4.20 (t, J = 7.4 Hz, 1H), 3.91 – 3.82 (m, 5H), 3.76 (s, 3H), 2.69 (td, J = 16.2, 7.4 Hz, 2H). ^{13}C-NMR ($CDCl_3$, 100 MHz): δ = 164.1, 158.3, 143.7, 135.4, 130.3, 128.7, 128.5, 127.4, 126.6, 126.4, 120.3 (t, J_{C-F} = 254.5 Hz), 114.6, 114.1, 68.1 (t, J_{C-F} = 35.2 Hz), 55.8, 55.2, 44.0 (t, J_{C-F} = 4.3 Hz), 39.4 (t, J_{C-F} = 22.9 Hz). ^{19}F-NMR ($CDCl_3$, 376 MHz): δ = -102.33 (dd, J = 327.1, 259.0 Hz). IR (ATR): 3329, 2917, 1674, 1465, 1281, 840 cm^{-1}. HR-MS (ESI) m/z calcd for $C_{24}H_{25}F_2O_5S$ [M+H$^+$] 463.1391, found 463.1395 [M+H$^+$].

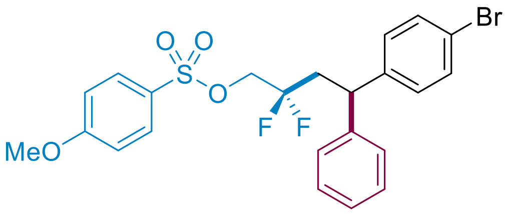

2,2-Difluoro-4-(4-bromophenyl)-4-phenylbutyl 4-methoxybenzenesulfonate (**50**)

The general procedure **TP2** was followed using **1i** (165 mg, 0.5 mmol), **2f** (46 mg, 0.25 mmol) and **3a** (0.5 mmol) for 3 h. Purification by column chromatography (*n*-hexane/EtOAc 40:1) yielded **50** (92 mg, 72%) as an oil. ^{1}H-NMR ($CDCl_3$, 400 MHz): δ = 7.80 – 7.75 (m, 2H), 7.42 – 7.36 (m, 2H), 7.31 – 7.26 (m, 2H), 7.24 – 7.16 (m, 3H), 7.12 – 7.06 (m, 2H), 7.02 – 6.97 (m, 2H), 4.23 (t, J = 7.4 Hz, 1H), 3.94 – 3.83 (m, 5H), 2.69 (td, J = 16.4, 7.3 Hz, 2H). ^{13}C-NMR ($CDCl_3$, 100 MHz): δ = 164.2, 142.8, 142.4, 131.8, 130.3, 129.3, 128.9, 127.4, 126.9, 126.3, 120.6, 120.1 (t, J_{C-F} = 241.3 Hz), 114.6, 68.11 (t, J_{C-F} = 35.9 Hz), 55.8, 44.2, 38.8 (d, J_{C-F} = 23.1 Hz). ^{19}F-NMR ($CDCl_3$, 376 MHz): δ = -102.52 (dd, J = 327.1,

259.0 Hz). IR (ATR): 3327, 2916, 1673, 1464, 1282, 763 cm^{-1}. HR-MS (ESI) m/z calcd for $C_{23}H_{22}BrF_2O_4S$ [M+H+] 511.0390, found 511.0395 [M+H+].

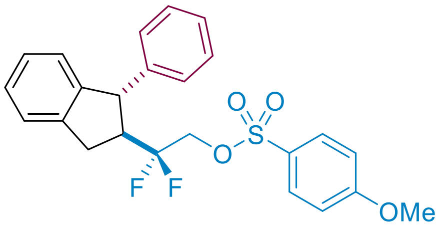

2,2-Difluoro-2-[(*trans*)-1-phenyl-2,3-dihydro-1*H*-inden-2-yl]ethyl 4-methoxybenzenesulfonate (51)

The general procedure **TP2** was followed using **1i** (165 mg, 0.5 mmol), **2o** (29 mg, 0.25 mmol) and **3a** ($Ph_2Zn \cdot 2Mg(OPiv)Cl$; 0.5 mmol) for 3 h. Purification by column chromatography (*n*-hexane/EtOAc 40:1) yielded **51** (60 mg, 54%; *dr* > 20:1) as an oil. ^{1}H-NMR ($CDCl_3$, 400 MHz): δ = 7.78 – 7.70 (m, 2H), 7.37 – 7.25 (m, 4H), 7.22 (t, J = 7.3 Hz, 1H), 7.19 – 7.10 (m, 3H), 7.03 – 6.95 (m, 2H), 6.78 (d, J = 7.6 Hz, 1H), 4.49 (d, J = 8.5 Hz, 1H), 4.15 (dd, J = 23.8, 12.4 Hz, 1H), 4.01 (t, J = 11.6 Hz, 1H), 3.91 (s, 3H), 3.27 – 3.09 (m, 3H). ^{13}C-NMR ($CDCl_3$, 100 MHz): δ = 164.1, 145.3, 143.5, 140.4, 130.3, 128.8, 128.5, 127.3, 127.1, 127.0, 126.4, 125.0, 124.2, 121.2 (t, J_{C-F} = 245.7 Hz), 114.5, 67.6 (t, J_{C-F} = 34.2 Hz), 55.8, 51.8 (t, J_{C-F} = 21.8 Hz), 51.4 (t, J_{C-F} = 4.1 Hz), 32.1 (d, J = 4.4 Hz). ^{19}F-NMR ($CDCl_3$, 376 MHz): δ = -109.50 (d, J = 23.3 Hz). IR (ATR): 3334, 2923, 1683, 1465, 1276, 693 cm^{-1}. HR-MS (ESI) m/z calcd for $C_{24}H_{23}F_2O_4S$ [M+H+] 445.1285, found 445.1289 [M+H+].

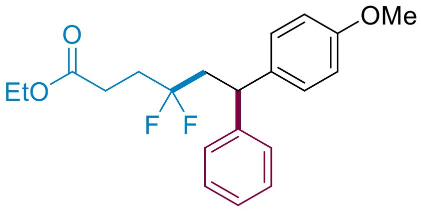

Ethyl 4,4-difluoro-6-(4-methoxyphenyl)-6-phenylhexanoate (52)

The general procedure **TP2** was followed using **1j** (115 mg, 0.5 mmol), **2a** (34 mg, 0.25 mmol) and **3a** ($Ph_2Zn \cdot 2Mg(OPiv)Cl$; 0.5 mmol) for 3 h. Purification by column chromatography (*n*-hexane/EtOAc 40:1) yielded **52** (48 mg, 53%) as an oil. ^{1}H-NMR ($CDCl_3$, 400 MHz): δ = 7.34 – 7.25 (m, 4H), 7.24 – 7.17 (m, 3H), 6.89 – 6.82 (m, 2H), 4.29 (t,

J = 7.1 Hz, 1H), 4.13 (q, J = 7.1 Hz, 2H), 3.79 (s, 3H), 2.75 – 2.62 (m, 2H), 2.44 (dd, J = 9.5, 6.1 Hz, 2H), 2.07 (ddd, J = 17.1, 16.5, 7.8 Hz, 2H), 1.33 – 1.21 (m, 4H). ^{13}C-NMR ($CDCl_3$, 100 MHz): δ = 172.3, 158.1, 144.5, 136.2, 128.6, 128.5, 127.5, 126.4, 114.0, 60.7, 55.2, 44.6 (t, J_{C-F} = 4.3 Hz), 42.6 (t, J_{C-F} = 24.5 Hz), 32.2 (t, J_{C-F} = 25.4 Hz), 27.1 (t, J_{C-F} = 4.8 Hz), 14.1. ^{19}F-NMR ($CDCl_3$, 376 MHz): δ = -97.24 (p, J = 16.4 Hz). IR (ATR): 3332, 2918, 1678, 1482, 1123 cm^{-1}. HR-MS (ESI) m/z calcd for $C_{21}H_{25}F_2O_3$ [M+H$^+$] 363.1772, found 363.1773 [M+H$^+$].

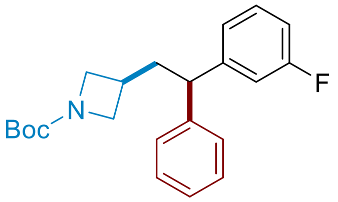

***tert*-Butyl-3-[2-(3-fluorophenyl)-2-phenylethyl]azetidine-1-carboxylate (53)**

The general procedure **TP2** was followed using **1-boc-3-iodoazetidine** (141 mg, 0.5 mmol), **2r** (31 mg, 0.25 mmol) and **3a** (0.5 mmol) for 16 h. Purification by column chromatography (*n*-hexane/EtOAc 40:1) yielded **53** (21.3 mg, 24%) as an oil. ^{1}H-NMR ($CDCl_3$, 400 MHz): δ = 7.29 (t, J = 7.4 Hz, 2H), 7.25 – 7.13 (m, 4H), 6.99 (d, J = 7.7 Hz, 1H), 6.88 (t, J = 10.1 Hz, 2H), 3.94 – 3.85 (m, 2H), 3.81 (t, J = 7.7 Hz, 1H), 3.53 (dd, J = 13.7, 8.1 Hz, 2H), 2.46 – 2.35 (m, 1H), 2.32 (t, J = 7.5 Hz, 2H), 1.41 (s, 9H). ^{13}C-NMR ($CDCl_3$, 100 MHz): δ = 162.9 (d, J_{C-F} = 241.3 Hz), 156.3, 146.9 (d, J_{C-F} = 6.9 Hz), 143.4, 130.03 (s), 129.9, 128.7, 127.7, 126.7, 123.4 (d, J_{C-F} = 2.5 Hz), 114.6 (d, J_{C-F} = 21.4 Hz), 113.4 (d, J_{C-F} = 21.1 Hz), 79.3, 49.0, 49.0, 40.1, 28.4, 27.4. ^{19}F-NMR ($CDCl_3$, 376 MHz): δ = -112.93 (s). IR (ATR): 3334, 2922, 1666, 1472, 1256, 1123, 802 cm^{-1}. HR-MS (ESI) m/z calcd for $C_{22}H_{27}FNO_2$ [M+H$^+$] 356.2026, found 356.2033 [M+H$^+$].

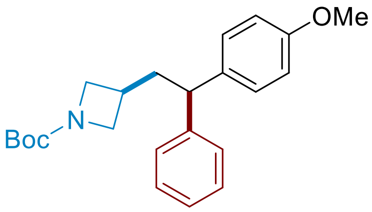

***tert*-Butyl-3-[2-(4-methoxyphenyl)-2-phenylethyl]azetidine-1-carboxylate (54)**

The general procedure **TP2** was followed using **1-boc-3-iodoazetidine** (141 mg, 0.5 mmol), **2a** (34 mg, 0.25 mmol) and **3a** (0.5 mmol) for 16 h. Purification by column chromatography (*n*-hexane/EtOAc 40:1) yielded **54** (54.1 mg, 59%) as an oil. ^{1}H-NMR ($CDCl_3$, 400 MHz): δ = 7.30 – 7.23 (m, 2H), 7.17 (dd, J = 10.1, 4.2 Hz, 3H), 7.11 (d, J = 8.6 Hz, 2H), 6.85 – 6.79 (m, 2H), 3.88 (t, J = 8.3 Hz, 2H), 3.80 – 3.73 (m, 4H), 3.52 (dd, J = 8.5, 5.7 Hz, 2H), 2.40 (tt, J = 7.9, 4.0 Hz, 1H), 2.29 (t, J = 7.7 Hz, 2H), 1.41 (s, 9H). ^{13}C-NMR ($CDCl_3$, 100 MHz): δ = 158.1, 156.3, 144.6, 136.3, 128.7, 128.5, 127.7, 126.3, 113.9, 79.2, 55.2, 48.4, 40.5, 28.4, 27.5. IR (ATR): 3333, 2921, 1642, 1482, 1266, 1077 cm^{-1}. HR-MS (ESI) m/z calcd for $C_{23}H_{30}NO_3$ [M+H$^+$] 368.2226, found 368.2237 [M+H$^+$].

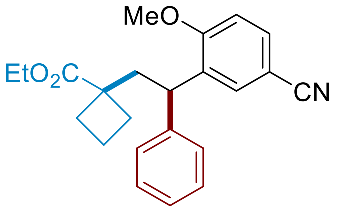

Ethyl 1-[2-(5-cyano-2-methoxyphenyl)-2-phenylethyl]cyclobutanecarboxylate (55)

The general procedure **TP2** was followed using **1-iodocyclobutane-1-carboxylate** (127 mg, 0.5 mmol), **2a** (40 mg, 0.25 mmol) and **3a** (0.5 mmol) for 16 h. Purification by column chromatography (*n*-hexane/EtOAc 40:1→20:1) yielded **55** (28.2 mg, 31%) as an oil. ^{1}H-NMR ($CDCl_3$, 400 MHz): δ = 7.53 (d, J = 2.1 Hz, 1H), 7.48 (dd, J = 8.5, 2.1 Hz, 1H), 7.32 – 7.23 (m, 4H), 7.22 – 7.16 (m, 1H), 6.85 (d, J = 8.5 Hz, 1H), 4.45 (t, J = 7.1 Hz, 1H), 3.87 (s, 3H), 3.72 – 3.60 (m, 2H), 2.57 (ddd, J = 41.0, 13.7, 7.1 Hz, 2H), 2.40 – 2.26 (m, 2H), 1.98 – 1.80 (m, 4H), 1.14 (t, J = 7.1 Hz, 3H). ^{13}C-NMR ($CDCl_3$, 100 MHz): δ = 176.4, 160.1, 143.1, 135.0, 132.1, 131.9, 128.3, 128.3, 126.5, 119.6, 111.0, 103.6, 60.2, 55.7, 47.4, 42.7, 39.6, 30.7, 15.9, 14.0. IR (ATR): 3331, 2916, 1676, 1446, 1226, 767 cm^{-1}. HR-MS (ESI) m/z calcd for $C_{22}H_{27}O_3$ [M+H$^+$] 339.1960, found 339.1968 [M+H$^+$].

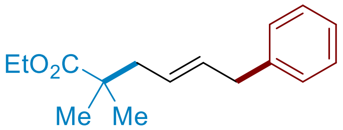

Ethyl -2,2-dimethyl-6-phenylhex-4-enoate (56)

The general procedure **TP3** was followed using **2-iodo-2-methylpropionate** (121 mg, 0.5 mmol), **2x** (27 mg, 0.5 mmol) and **3a** (0.5 mmol) for 16 h. Purification by column chromatography (*n*-hexane/EtOAc 60:1) yielded **56** (25.2 mg, 41%) as an oil. ^{1}H-NMR ($CDCl_3$, 400 MHz): δ = 7.20 (d, J = 7.5 Hz, 2H), 7.10 (t, J = 8.9 Hz, 3H), 5.59 – 5.48 (m, 1H), 5.38 (dt, J = 14.8, 7.3 Hz, 1H), 4.01 (q, J = 7.1 Hz, 2H), 3.26 (d, J = 6.7 Hz, 2H), 2.18 (d, J = 7.3 Hz, 2H), 1.14 (d, J = 7.1 Hz, 3H), 1.09 (s, 6H). ^{13}C-NMR ($CDCl_3$, 100 MHz): δ = 177.6, 140.7, 132.4, 128.5, 128.4, 127.2, 125.9, 60.3, 43.5, 42.5, 39.1, 24.9, 14.2. IR (ATR): 2919, 1722, 1676, 1446, 1112 cm^{-1}. HR-MS (ESI) m/z calcd for $C_{16}H_{23}O_2$ [M+H$^+$] 247.1698, found 247. [M+H$^+$].

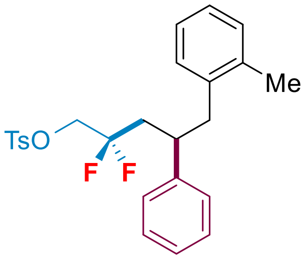

2,2-Difluoro-4-phenyl-5-(o-tolyl)pentyl 4-methylbenzenesulfonate (57)

The general procedure **TP3** was followed using **1k** (78 mg, 0.25 mmol), **2r** (66 mg, 0.5 mmol) and **3a** (0.5 mmol) for 12 h. Purification by column chromatography (*n*-hexane/EtOAc 40:1) yielded **57** (56.6 mg, 51%) as an oil. ^{1}H-NMR ($CDCl_3$, 400 MHz): δ = 7.71 (d, J = 8.3 Hz, 2H), 7.34 (d, J = 8.0 Hz, 2H), 7.28 – 7.18 (m, 3H), 7.14 – 7.06 (m, 4H), 7.06 – 7.00 (m, 1H), 6.87 (d, J = 7.3 Hz, 1H), 3.91 (dd, J = 23.1, 11.2 Hz, 1H), 3.78 (dt, J = 14.3, 10.6 Hz, 1H), 3.21 – 3.10 (m, 1H), 2.97 (dd, J = 13.7, 7.1 Hz, 1H), 2.85 (dd, J = 13.7, 7.9 Hz, 1H), 2.47 (s, 3H), 2.42 – 2.30 (m, 2H), 2.22 (s, 3H). ^{13}C-NMR ($CDCl_3$, 100 MHz): δ = 145.3, 143.3, 137.4, 136.3, 132.1, 130.3, 130.0, 130.0, 128.5, 128.0, 127.4, 126.8, 126.4, 125.7, 120.4 (t, J_{C-F} = 245.1 Hz), 68.2 (dd, J_{C-F} = 36.2, 34.1 Hz), 41.5, 40.3 (t, J_{C-F} = 3.5 Hz), 38.59 (t, J_{C-F} = 22.6 Hz), 21.7, 19.4. ^{19}F-NMR ($CDCl_3$, 376 MHz): δ = -100.4 (d, J = 257.9 Hz), -104.3 (d, J = 257.9 Hz). IR (ATR): 3329, 2923, 1662, 1464, 1122, 973 cm^{-1}. HR-MS (ESI) m/z calcd for $C_{25}H_{27}F_2O_3S$ [M+H$^+$] 445.1649, found 445.1641 [M+H$^+$].

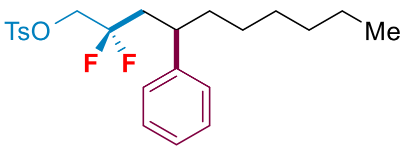

2,2-Difluoro-4,6-diphenylhexyl 4-methylbenzenesulfonate (58)

The general procedure **TP3** was followed using **1k** (78 mg, 0.25 mmol), **2s** (56 mg, 0.5 mmol) and **3a** (0.5 mmol) for 12 h. Purification by column chromatography (*n*-hexane/EtOAc 40:1) yielded **58** (49.8 mg, 47%) as an oil. ^{1}H-NMR ($CDCl_3$, 400 MHz): δ = 7.74 – 7.68 (m, 2H), 7.33 (d, J = 8.0 Hz, 2H), 7.27 (qd, J = 3.0, 1.3 Hz, 2H), 7.22 – 7.16 (m, 1H), 7.10 (dd, J = 5.2, 3.2 Hz, 2H), 3.90 (q, J = 11.3 Hz, 1H), 3.83 – 3.72 (m, 1H), 2.86 – 2.75 (m, 1H), 2.45 (s, 3H), 2.30 – 2.15 (m, 2H), 1.69 –1.50 (m, 2H), 1.28 – 1.13 (m, 7H), 1.10 – 0.98 (m, 1H), 0.83 (t, J = 7.0 Hz, 3H). ^{13}C-NMR ($CDCl_3$, 100 MHz): δ = 145.4, 144.1, 132.1, 130.0, 128.5, 128.0, 127.4, 126.5, 120.5 (t, J_{C-F} = 245.1 Hz), 68.2 (dd, J_{C-F} = 36.5, 34.1 Hz), 40.2 (t, J_{C-F} = 22.5 Hz), 39.6 (t, J_{C-F} = 3.7 Hz), 37.4, 31.7, 29.1, 27.2, 22.6, 21.7, 14.0. ^{19}F-NMR ($CDCl_3$, 376 MHz): δ = -100.43 (d, J = 257.3 Hz), -104.61 (d, J = 257.3 Hz). IR (ATR): 3332, 2938, 1572, 1463, 1088, 877 cm^{-1}. HR-MS (ESI) m/z calcd for $C_{23}H_{31}F_2O_3S$ [M+H$^+$] 425.1962, found 425.1954 [M+H$^+$].

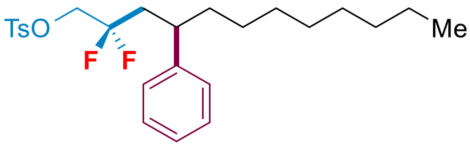

2,2-Difluoro-4-phenyldodecyl 4-methylbenzenesulfonate (59)

The general procedure **TP3** was followed using **1k** (78 mg, 0.25 mmol), **2t** (70 mg, 0.5 mmol) and **3a** (0.5 mmol) for 12 h. Purification by column chromatography (*n*-hexane/EtOAc 40:1) yielded **59** (59.9 mg, 53%) as an oil. ^{1}H-NMR ($CDCl_3$, 400 MHz): δ = 7.74 (d, J = 8.3 Hz, 2H), 7.36 (d, J = 8.0 Hz, 2H), 7.32 – 7.26 (m, 2H), 7.25 – 7.19 (m, 1H), 7.16 – 7.10 (m, 2H), 3.92 (q, J = 11.3 Hz, 1H), 3.80 (dt, J = 14.5, 10.5 Hz, 1H), 2.89 – 2.77 (m, 1H), 2.48 (s, 3H), 2.33 – 2.15 (m, 2H), 1.71 – 1.54 (m, 2H), 1.33 – 1.03 (m, 12H), 0.88 (t, J = 7.0 Hz, 3H). ^{13}C-NMR ($CDCl_3$, 100 MHz): δ = 145.4, 144.1, 132.2, 130.0, 128.6, 128.0, 127.4, 126.5, 120.5 (d, J_{C-F} = 244.2 Hz), 68.3 (dd, J_{C-F} = 36.6, 34.4 Hz), 40.2 (t, J_{C-F} = 22.5 Hz), 39.6 (t, J_{C-F} = 3.5 Hz), 37.4, 31.8, 29.4, 29.4, 29.2, 27.2, 22.6, 21.7, 14.1. ^{19}F-NMR ($CDCl_3$, 376

MHz): δ = -100.41 (d, J = 257.9 Hz), -104.52 (d, J = 256.8 Hz). IR (ATR): 3324, 2931, 1568, 1472, 1088, 868 cm^{-1}. HR-MS (ESI) m/z calcd for $C_{25}H_{35}F_2O_3S$ [M+H$^+$] 453.2275, found 453.2269 [M+H$^+$].

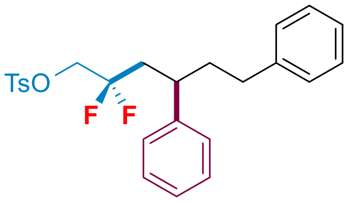

2,2-Difluoro-4,6-diphenylhexyl 4-methylbenzenesulfonate (60)

The general procedure **TP3** was followed using **1k** (78 mg, 0.25 mmol), **2u** (66 mg, 0.5 mmol) and **3a** (0.5 mmol) for 12 h. Purification by column chromatography (n-hexane/EtOAc 40:1) yielded **60** (51 mg, 46%) as an oil. ^{1}H-NMR ($CDCl_3$, 400 MHz): δ = 7.74 – 7.67 (m, 2H), 7.35 – 7.28 (m, 4H), 7.25 – 7.20 (m, 3H), 7.16 (ddd, J = 13.0, 6.8, 4.1 Hz, 3H), 7.10 – 7.03 (m, 2H), 3.90 (q, J = 11.2 Hz, 1H), 3.77 (ddd, J = 14.9, 10.9, 9.8 Hz, 1H), 2.93 – 2.82 (m, 1H), 2.44 (s, 3H), 2.39 (t, J = 8.0 Hz, 2H), 2.33 – 2.17 (m, 2H), 2.07 – 1.96 (m, 1H), 1.96 – 1.82 (m, 1H). ^{13}C-NMR ($CDCl_3$, 100 MHz): δ = 145.4, 143.5, 141.7, 132.1, 130.0, 128.7, 128.4, 128.3, 128.0, 127.5, 126.8, 125.9, 120.4 (t, J_{C-F} = 245.1 Hz), 68.3 (dd, J_{C-F} = 36.2, 34.1 Hz), 40.3 (t, J_{C-F} = 22.5 Hz), 39.2 t, (J_{C-F} = 3.5 Hz), 38.8, 33.4, 21.7. ^{19}F-NMR ($CDCl_3$, 376 MHz): δ = -100.42 (d, J = 257.7 Hz), -104.73 (d, J = 257.7 Hz). IR (ATR): 3329, 2928, 1581, 1466, 1083, 891 cm^{-1}. HR-MS (ESI) m/z calcd for $C_{25}H_{27}F_2O_3S$ [M+H$^+$] 445.1649, found 445.1643 [M+H$^+$].

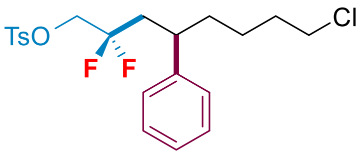

8-Chloro-2,2-difluoro-4-phenyloctyl 4-methylbenzenesulfonate (61)

The general procedure **TP3** was followed using **1k** (78 mg, 0.25 mmol), **2v** (59 mg, 0.5 mmol) and **3a** (0.5 mmol) for 12 h. Purification by column chromatography (n-hexane/EtOAc 40:1) yielded **61** (46.2 mg, 43%) as an oil. ^{1}H-NMR ($CDCl_3$, 400 MHz): δ = 7.75 – 7.68 (m, 2H), 7.34 (d, J = 8.0 Hz, 2H), 7.31 – 7.26 (m, 2H), 7.24 – 7.17 (m, 1H), 7.11

(dd, J = 5.2, 3.2 Hz, 2H), 3.92 (dd, J = 22.9, 11.0 Hz, 1H), 3.79 (ddd, J = 14.7, 10.9, 9.7 Hz, 1H), 3.43 (td, J = 6.7, 3.7 Hz, 2H), 2.89 – 2.77 (m, 1H), 2.46 (s, 3H), 2.31 – 2.16 (m, 2H), 1.75 – 1.58 (m, 4H), 1.18 (ddd, J = 13.3, 9.4, 4.9 Hz, 2H). ^{13}C-NMR ($CDCl_3$, 100 MHz): δ = 145.4, 143.5, 132.1, 130.0, 128.7, 128.0, 127.4, 126.7, 120.4 (t, J_{C-F} = 245.3 Hz), 68.3 (dd, J_{C-F} = 36.5, 34.4 Hz), 44.7, 40.2 (t, J_{C-F} = 22.5 Hz), 39.4 (t, J_{C-F} = 3.5 Hz), 36.5, 32.3, 29.7, 24.6, 21.7. ^{19}F-NMR ($CDCl_3$, 376 MHz): δ = -100.63 (d, J = 257.9 Hz), -104.7 (d, J = 257.9 Hz). IR (ATR): 3328, 2921, 1716, 1572, 1078, 877 cm^{-1}. HR-MS (ESI) m/z calcd for $C_{21}H_{26}ClF_2O_3S$ [M+H$^+$] 431.1259, found 431.1266 [M+H$^+$].

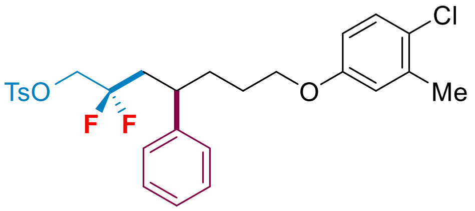

7-(4-Chloro-3-methylphenoxy)-2,2-difluoro-4-phenylheptyl 4-methylbenzenesulfonate (62)

The general procedure **TP3** was followed using **1k** (78 mg, 0.25 mmol), **2q** (105 mg, 0.5 mmol) and **3a** (0.5 mmol) for 12 h. Purification by column chromatography (*n*-hexane/EtOAc 40:1) yielded **62** (62.6 mg, 48%) as an oil. ^{1}H-NMR ($CDCl_3$, 400 MHz): δ = 7.74 (d, J = 8.3 Hz, 2H), 7.38 – 7.29 (m, 4H), 7.27 – 7.12 (m, 4H), 6.73 (d, J = 2.9 Hz, 1H), 6.62 (dd, J = 8.7, 3.0 Hz, 1H), 3.95 (q, J = 11.1 Hz, 1H), 3.88 – 3.78 (m, 3H), 2.93 (dt, J = 12.7, 6.8 Hz, 1H), 2.47 (s, 3H), 2.34 (s, 3H), 2.33 – 2.23 (m, 2H), 1.91 (ddd, J = 15.3, 10.5, 5.4 Hz, 1H), 1.74 (dtd, J = 14.7, 9.9, 5.0 Hz, 1H), 1.68 – 1.47 (m, 2H). ^{13}C-NMR ($CDCl_3$, 100 MHz): δ = 157.5, 145.4, 143.4, 136.9, 132.1, 130.0, 129.5, 128.7, 128.0, 127.4, 126.8, 125.7, 120.4 (t, J_{C-F} = 244.2 Hz), 117.0, 113.0, 68.3 (dd, J_{C-F} = 36.2, 34.5 Hz), 67.7, 40.2 (d, J_{C-F} = 22.6 Hz), 39.3 (t, J_{C-F} = 3.5 Hz), 33.6, 27.0, 21.7, 20.3. ^{19}F-NMR ($CDCl_3$, 376 MHz): δ = -100.54 (d, J = 258.0 Hz), -104.6 (d, J = 258.0 Hz). IR (ATR): 3329, 2924, 1715, 1572, 1083, 868 cm^{-1}. HR-MS (ESI) m/z calcd for $C_{27}H_{30}ClF_2O_4S$ [M+H$^+$] 523.1521, found 523.1514 [M+H$^+$].

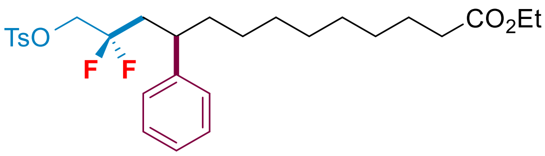

Ethyl 12,12-difluoro-10-phenyl-13-(tosyloxy)tridecanoate (63)

The general procedure **TP3** was followed using **1k** (78 mg, 0.25 mmol), **2w** (106 mg, 0.5 mmol) and **3a** (0.5 mmol) for 12 h. Purification by column chromatography (*n*-hexane/EtOAc 40:1) yielded **63** (45.9 mg, 35%) as an oil. ^{1}H-NMR ($CDCl_3$, 400 MHz): δ = 7.74 – 7.68 (m, 2H), 7.33 (d, J = 8.0 Hz, 2H), 7.27 (ddd, J = 6.2, 3.3, 1.2 Hz, 2H), 7.22 – 7.16 (m, 1H), 7.10 (dd, J = 5.2, 3.2 Hz, 2H), 4.11 (q, J = 7.1 Hz, 2H), 3.90 (q, J = 11.3 Hz, 1H), 3.77 (dt, J = 14.5, 10.5 Hz, 1H), 2.86 – 2.74 (m, 1H), 2.45 (s, 3H), 2.31 – 2.16 (m, 4H), 1.68 – 1.59 (m, 2H), 1.55 (dd, J = 9.7, 5.5 Hz, 2H), 1.35 – 1.13 (m, 13H). ^{13}C-NMR ($CDCl_3$, 100 MHz): δ = 173.9, 145.4, 144.0, 132.1, 130.0, 128.6, 128.0, 127.4, 126.6, 120.5 (t, J_{C-F} = 244.2 Hz), 68.3 (dd, J_{C-F} = 36.6, 34.4 Hz), 60.2, 40.2 (t, J_{C-F} = 22.4 Hz), 39.6 (t, J_{C-F} = 3.5 Hz), 37.4, 34.4, 29.3, 29.2, 29.2, 29.1, 27.1, 24.9, 21.7, 14.3. ^{19}F-NMR ($CDCl_3$, 376 MHz): δ = -100.43 (d, J = 257.3 Hz), -104.6 (d, J = 257.3 Hz). IR (ATR): 3334, 2932, 1723, 1581, 1242, 1121, 864 cm^{-1}. HR-MS (ESI) m/z calcd for $C_{28}H_{39}F_2O_5S$ [M+H$^+$] 525.2486, found 525.2478 [M+H$^+$].

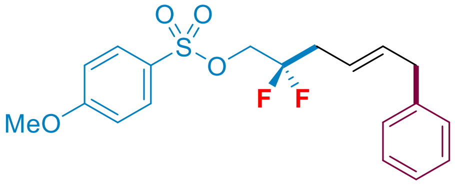

(*E*)-2,2-Difluoro-6-phenylhex-4-en-1-yl 4-methoxybenzenesulfonate (64)

The general procedure **TP3** was followed using **1i** (83 mg, 0.25 mmol), **2x** (27 mg, 0.5 mmol) and **3a** (0.5 mmol) for 12 h. Purification by column chromatography (*n*-hexane/EtOAc 40:1) yielded **64** (76 mg, 80%) as an oil. ^{1}H-NMR (400 MHz, $CDCl_3$): δ = 7.86 – 7.80 (m, 2H), 7.33$_2$ – 7.26 (m, 2H), 7.24 – 7.18 (m, 1H), 7.17 – 7.11 (m, 2H), 7.03 – 6.97 (m, 2H), 5.77 (dt, J = 14.0, 6.8 Hz, 1H), 5.44 – 5.32 (m, 1H), 4.09 (t, J = 11.4 Hz, 2H), 3.88 (s, 3H), 3.34 (d, J = 6.8 Hz, 2H), 2.64 (td, J = 16.1, 7.2 Hz, 2H). ^{13}C-NMR (100 MHz, $CDCl_3$): δ = 163.1, 138.6, 135.4, 129.3, 127.5, 127.4, 125.4, 125.2, 119.4 (t, J_{C-F} = 5.6 Hz), 118.9 (t, J_{C-F} = 244.5 Hz), 113.6, 66.3 (t, J_{C-F} = 35.8 Hz), 54.7, 37.9, 35.9 (t, J_{C-F} = 24.1 Hz).

^{13}F-NMR (376 MHz, $CDCl_3$): δ = 104.81 (s). IR (ATR): 2923, 1727, 1644, 1266, 1049, 759 cm^{-1}. HR-MS (EI) m/z calcd for $C_{19}H_{21}F_2O_4S$ [M+H$^+$] 383.1129, found 383.1138

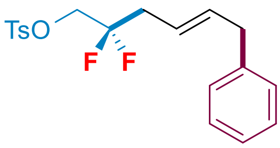

(*E*)-2,2-Difluoro-6-phenylhex-4-en-1-yl 4-methylbenzenesulfonate (65)

The general procedure **TP3** was followed using **1k** (79 mg, 0.25 mmol), **2x** (27 mg, 0.5 mmol) and **3a** (0.5 mmol) for 12 h. Purification by column chromatography (*n*-hexane/EtOAc 40:1) yielded **65** (90 mg, 98%) as an oil. ^{1}H-NMR (400 MHz, $CDCl_3$): δ = 7.78 (d, J = 8.3 Hz, 2H), 7.35 (d, J = 8.2 Hz, 2H), 7.29 (t, J = 7.3 Hz, 2H), 7.21 (dd, J = 8.4, 6.3 Hz, 1H), 7.14 (d, J = 7.1 Hz, 2H), 5.82 – 5.70 (m, 1H), 5.44 – 5.31 (m, 1H), 4.10 (t, J = 11.3 Hz, 2H), 3.34 (d, J = 6.8 Hz, 2H), 2.64 (td, J = 16.1, 7.2 Hz, 2H), 2.45 (s, 3H). ^{13}C-NMR (100 MHz, $CDCl_3$): δ = 145.5, 139.7, 136.4, 132.2, 130.0, 128.5, 128.5, 128.1, 126.2, 120.4, 119.9 (t, J_{C-F} = 244.6 Hz), 67.5 (t, J_{C-F} = 35.7 Hz), 38.9, 37.0 (t, J_{C-F} = 24.0 Hz), 21.7. ^{13}F-NMR (376 MHz, $CDCl_3$): δ = -104.82 (s). IR (ATR): 2921, 1719, 1637, 1271, 1051, 751 cm^{-1}. HR-MS (EI) m/z calcd for $C_{19}H_{21}F_2O_3S$ [M+H$^+$] 367.1179, found 367.1176.

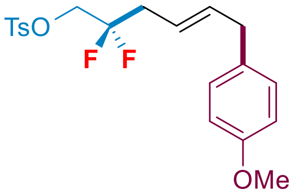

(*E*)-2,2-Difluoro-6-(4-methoxyphenyl)hex-4-en-1-yl 4-methylbenzenesulfonate (66)

The general procedure **TP3** was followed using **1k** (79 mg, 0.25 mmol), **2x** (27 mg, 0.5 mmol) and **3b** (0.5 mmol) for 12 h. Purification by column chromatography (*n*-hexane/EtOAc 40:1) yielded **66** (62 mg, 63%) as an oil. ^{1}H-NMR (400 MHz, $CDCl_3$): δ = 7.78 (d, J = 8.3 Hz, 2H), 7.35 (d, J = 8.1 Hz, 2H), 7.07 – 7.02 (m, 2H), 6.83 (d, J = 8.6 Hz, 2H), 5.80 – 5.67 (m, 1H), 5.41 – 5.29 (m, 1H), 4.10 (t, J = 11.4 Hz, 2H), 3.79 (s, 3H), 3.27 (d, J = 6.8 Hz, 2H), 2.63 (td, J = 16.2, 7.2 Hz, 2H), 2.45 (s, 3H). ^{13}C-NMR (100 MHz, $CDCl_3$): δ = 158.1, 145.5, 136.9, 132.2, 131.7, 130.0, 129.4, 128.1, 120.0 (t, J_{C-F} = 5.7 Hz), 119.9 (t, J_{C-F} = 243.6 Hz), 113.9, 67.5 (t, J_{C-F} = 35.8 Hz), 55.3, 38.0, 36.9 (t, J_{C-F} = 24.1 Hz), 21.7. ^{19}F-NMR

(376 MHz, $CDCl_3$): δ = -104.82 (s). IR (ATR): 2918, 1715, 1633, 1226, 1067, 758 cm^{-1}. HR-MS (EI) m/z calcd for $C_{20}H_{23}F_2O_4S$ [M+H$^+$] 397.1285, found 397.1281.

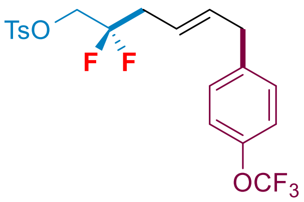

(*E*)-2,2-Difluoro-6-[4-(trifluoromethoxy)phenyl]hex-4-en-1-yl 4-methylbenzenesulfonate (67)

The general procedure **TP3** was followed using **1k** (79 mg, 0.25 mmol), **2x** (27 mg, 0.5 mmol) and **3u** (0.5 mmol) for 12 h. Purification by column chromatography (*n*-hexane/EtOAc 40:1) yielded **67** (49 mg, 43%) as an oil. ^{1}H-NMR (400 MHz, $CDCl_3$): 7.79 (d, *J* = 8.3 Hz, 2H), 7.36 (d, *J* = 8.3 Hz, 2H), 7.21 – 7.09 (m, 4H), 5.84 – 5.69 (m, 1H), 5.41 (dt, *J* = 14.7, 7.3 Hz, 1H), 4.10 (t, *J* = 11.3 Hz, 2H), 3.35 (d, *J* = 6.8 Hz, 2H), 2.66 (td, *J* = 16.2, 7.2 Hz, 2H), 2.45 (s, 3H). ^{13}C-NMR (100 MHz, $CDCl_3$): δ = 147.7, 145.5, 138.4, 135.7, 132.1, 130.0, 129.7, 128.1, 121.1, 121.0 (t, J_{C-F} = 5.6 Hz), 119.9 (t, J_{C-F} = 243.8 Hz), 119.2 (t, J_{C-F} = 259.6 Hz), 67.5 (t, J_{C-F} = 36.1 Hz), 38.2, 36.9 (t, J_{C-F} = 24.1 Hz), 21.7. ^{19}F-NMR (376 MHz, $CDCl_3$): δ = -57.92 (s), -104.81 (s). IR (ATR): 2933, 1714, 1626, 1178, 1042, 776 cm^{-1}. HR-MS (EI) m/z calcd for $C_{20}H_{20}F_5O_4S$ [M+H$^+$] 451.1002, found 451.1012.

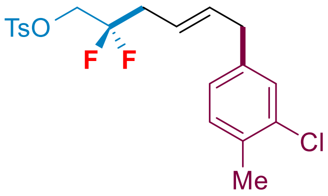

(*E*)-6-(3-Chloro-4-methylphenyl)-2,2-difluorohex-4-en-1-yl 4-methylbenzenesulfonate (68)

The general procedure **TP3** was followed using **1k** (79 mg, 0.25 mmol), **2x** (27 mg, 0.5 mmol) and **3t** (0.5 mmol) for 12 h. Purification by column chromatography (*n*-hexane/EtOAc 40:1) yielded **68** (59 mg, 57%) as an oil. ^{1}H-NMR (400 MHz, $CDCl_3$): δ = 7.82 – 7.76 (m, 2H), 7.36 (d, *J* = 8.0 Hz, 2H), 7.16 – 7.09 (m, 2H), 6.93 (dt, *J* = 7.7, 3.7 Hz, 1H), 5.72 (dt, *J* = 13.9, 6.8 Hz, 1H), 5.43 – 5.32 (m, 1H), 4.10 (t, *J* = 11.3 Hz, 2H), 3.27 (d, *J*

= 6.8 Hz, 2H), 2.64 (td, *J* = 16.2, 7.2 Hz, 2H), 2.45 (s, 3H), 2.34 (s, 3H). ^{13}C-NMR (100 MHz, $CDCl_3$): δ = 145.5, 138.9, 135.8, 134.3, 133.8, 132.1, 131.0, 130.0, 128.9, 128.1, 126.7, 120.8 (t, J_{C-F} = 5.6 Hz), 119.9 (d, J_{C-F} = 244.2 Hz), 67.5 (t, J_{C-F} = 35.9 Hz), 38.0, 36.9 (t, J_{C-F} = 24.1 Hz), 21.7, 19.6. ^{13}F-NMR (376 MHz, $CDCl_3$): δ = -104.80 (s). IR (ATR): 2923, 1732, 1638, 1148, 1028, 788 cm^{-1}. HR-MS (EI) m/z calcd for $C_{20}H_{22}ClF_2O_3S$ [M+H$^+$] 415.0946, found 415.0958.

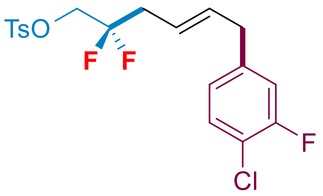

(*E*)-6-(4-Chloro-3-fluorophenyl)-2,2-difluorohex-4-en-1-yl 4-methylbenzenesulfonate (69)

The general procedure **TP3** was followed using **1k** (79 mg, 0.25 mmol), **2x** (27 mg, 0.5 mmol) and **3v** (0.5 mmol) for 12 h. Purification by column chromatography (*n*-hexane/EtOAc 40:1) yielded **69** (72 mg, 69%) as an oil. ^{1}H-NMR (400 MHz, $CDCl_3$): δ = 7.81 – 7.77 (m, 2H), 7.37 (d, *J* = 8.0 Hz, 2H), 7.17 (dd, *J* = 7.0, 2.1 Hz, 1H), 7.06 (t, *J* = 8.6 Hz, 1H), 7.03 – 6.97 (m, 1H), 5.79 – 5.67 (m, 1H), 5.46 – 5.35 (m, 1H), 4.09 (t, *J* = 11.2 Hz, 2H), 3.30 (d, *J* = 6.7 Hz, 2H), 2.66 (td, *J* = 16.0, 6.9 Hz, 2H), 2.46 (s, 3H). ^{13}C-NMR (100 MHz, $CDCl_3$): δ = 156.7S5 (d, J_{C-F} = 247.0 Hz), 145.6, 136.6, 136.6, 135.4, 132.1, 130.4, 130.1, 128.1, 121.3 (t, J_{C-F} = 5.6 Hz), 120.7 (d, J_{C-F} = 17.5 Hz), 119.8 (t, J_{C-F} = 243.4 Hz), 116.5 (d, J_{C-F} = 20.8 Hz), 67.5 (t, J_{C-F} = 36.1 Hz), 37.8, 36.9 (t, J_{C-F} = 24.2 Hz), 21.7. ^{19}F-NMR (376 MHz, $CDCl_3$): δ = -104.79 (s), -109.36 (s). IR (ATR): 2923, 1724, 1568, 1153, 1019, 817 cm^{-1}. HR-MS (EI) m/z calcd for $C_{19}H_{19}ClF_3O_3S$ [M$^+$] 419.0696, found 419.0706.

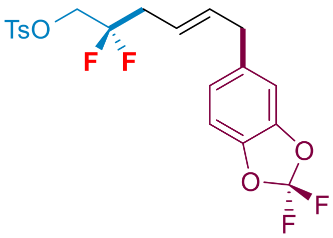

(*E*)-6-(2,2-Difluorobenzo[*d*][1,3]dioxol-5-yl)-2,2-difluorohex-4-en-1-yl 4-

methylbenzenesulfonate (70)

The general procedure **TP3** was followed using **1k** (79 mg, 0.25 mmol), **2x** (27 mg, 0.5 mmol) and **3w** (0.5 mmol) for 12 h. Purification by column chromatography (*n*-hexane/EtOAc 40:1) yielded **70** (57 mg, 51%) as an oil. ^{1}H-NMR (400 MHz, $CDCl_3$): δ = 7.81 – 7.77 (m, 2H), 7.37 (d, J = 8.0 Hz, 2H), 6.96 (dd, J = 7.7, 0.9 Hz, 1H), 6.88 – 6.82 (m, 2H), 5.80 – 5.68 (m, 1H), 5.47 – 5.35 (m, 1H), 4.10 (t, J = 11.3 Hz, 2H), 3.33 (d, J = 6.8 Hz, 2H), 2.66 (td, J = 16.2, 7.2 Hz, 2H), 2.46 (s, 3H). ^{13}C-NMR (100 MHz, $CDCl_3$): δ = 145.6, 143.9, 142.2, 135.8, 135.7, 132.1, 131.7 (t, J_{C-F} = 245.2 Hz), 130.1, 128.1, 123.3, 121.2 (t, J_{C-F} = 5.5 Hz), 119.8 (t, J_{C-F} = 244.5 Hz), 109.7, 109.3, 67.5 (t, J_{C-F} = 36.1 Hz), 38.6, 36.9 (t, J_{C-F} = 24.1 Hz), 21.7. ^{13}F-NMR (376 MHz, $CDCl_3$): δ = -50.06 (s), -104.79 (s). IR (ATR): 2918, 1729, 1566, 1271, 1153, 769 cm^{-1}. HR-MS (EI) m/z calcd for $C_{20}H_{19}F_4O_5S$ [M+H$^+$] 447.0889, found 447.0897.

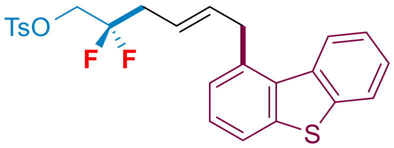

(*E*)-6-(Dibenzo[*b,d*]thiophen-1-yl)-2,2-difluorohex-4-en-1-yl 4-methylbenzenesulfonate (71)

The general procedure **TP3** was followed using **1k** (79 mg, 0.25 mmol), **2x** (27 mg, 0.5 mmol) and **3x** (0.5 mmol) for 12 h. Purification by column chromatography (*n*-hexane/EtOAc 40:1) yielded **71** (84 mg, 71%) as an oil. ^{1}H-NMR (400 MHz, $CDCl_3$): δ = 8.21 – 8.15 (m, 1H), 8.08 (dd, J = 7.9, 0.9 Hz, 1H), 7.94 – 7.88 (m, 1H), 7.81 – 7.73 (m, 2H), 7.52 – 7.44 (m, 3H), 7.35 – 7.25 (m, 3H), 5.93 – 5.82 (m, 1H), 5.61 – 5.49 (m, 1H), 4.16 (t, J = 11.4 Hz, 2H), 3.63 (d, J = 6.7 Hz, 2H), 2.70 (td, J = 16.1, 7.3 Hz, 2H), 2.43 (s, 3H). ^{13}C-NMR (100 MHz, $CDCl_3$): δ = 145.5, 139.2, 139.1, 136.0, 135.9, 133.9, 133.9, 132.1, 130.0, 128.0, 126.8, 126.4, 125.0, 124.5, 122.9, 121.8 (t, J_{C-F} = 5.7 Hz), 121.8, 119.9 (t, J_{C-F} = 243.6 Hz), 119.8, 67.5 (t, J_{C-F} = 35.5 Hz), 38.1, 37.0 (t, J_{C-F} = 24.2 Hz), 21.7. ^{19}F-NMR (376 MHz, $CDCl_3$): δ = -104.60 (s). IR (ATR): 2923, 1726, 1473, 1244, 923 cm^{-1}. HR-MS (EI) m/z calcd for $C_{25}H_{23}F_2O_3S_2$ [M+H$^+$] 473.1057, found 473.1052.

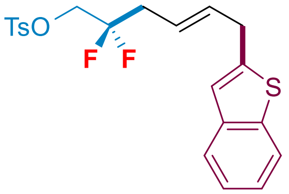

(*E*)-6-(Benzo[*b*]thiophen-2-yl)-2,2-difluorohex-4-en-1-yl 4-methylbenzenesulfonate (72)

The general procedure **TP3** was followed using **1k** (79 mg, 0.25 mmol), **2x** (27 mg, 0.5 mmol) and **3y** (0.5 mmol) for 12 h. Purification by column chromatography (*n*-hexane/EtOAc 40:1) yielded **72** (50 mg, 44%) as an oil. ^{1}H-NMR (400 MHz, $CDCl_3$): δ = 7.88 – 7.83 (m, 1H), 7.79 – 7.74 (m, 2H), 7.71 – 7.67 (m, 1H), 7.41 – 7.31 (m, 4H), 7.08 (s, 1H), 5.93 – 5.81 (m, 1H), 5.53 – 5.41 (m, 1H), 4.09 (t, *J* = 11.3 Hz, 2H), 3.55 (d, *J* = 6.5 Hz, 2H), 2.66 (td, *J* = 15.9, 7.0 Hz, 2H), 2.42 (s, 3H). ^{13}C-NMR (100 MHz, $CDCl_3$): δ = 145.5, 140.6, 138.7, 134.6, 134.2, 132.1, 130.1, 128.1, 124.3, 124.0, 122.9, 122.2, 121.7, 121.3 (t, J_{C-F} = 5.6 Hz), 119.9 (t, J_{C-F} = 244.2 Hz), 67.5 (t, J_{C-F} = 36.0 Hz), 36.9 (t, J_{C-F} = 24.1 Hz), 31.9, 21.7. ^{19}F-NMR (376 MHz, $CDCl_3$): δ = -104.70 (s). IR (ATR): 2921, 1716, 1547, 1138, 913 cm^{-1}. HR-MS (EI) m/z calcd for $C_{17}H_{21}O_4$ [M+H$^+$] 289.1440, found 289.1434.

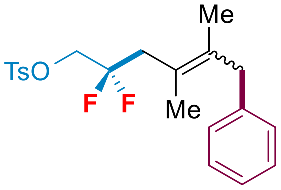

2,2-Difluoro-4,5-dimethyl-6-phenylhex-4-en-1-yl 4-methylbenzenesulfonate (73)

The general procedure **TP3** was followed using **1k** (79 mg, 0.25 mmol), **2y** (41 mg, 0.5 mmol) and **3a** (0.5 mmol) for 12 h. Purification by column chromatography (*n*-hexane/EtOAc 40:1) yielded **73** (80 mg, 81%) as an oil. ^{1}H-NMR (400 MHz, $CDCl_3$): δ = 7.87 – 7.76 (m, 2H), 7.37 (t, *J* = 8.0 Hz, 2H), 7.33 – 7.25 (m, 2H), 7.25 – 7.18 (m, 1H), 7.12 (ddd, *J* = 13.9, 7.8, 0.9 Hz, 2H), 4.13 (td, *J* = 11.5, 2.2 Hz, 2H), 3.43 (d, *J* = 23.9 Hz, 2H), 2.80 (td, *J* = 17.4, 12.0 Hz, 2H), 2.48 (d, *J* = 5.1 Hz, 3H), 1.89 – 1.76 (m, 3H), 1.62 (d, *J* = 0.8 Hz, 3H). ^{13}C-NMR (100 MHz, $CDCl_3$): δ = 145.5 (2C), 139.8, 139.8, 134.5, 134.1, 132.2, 132.2, 130.0, 128.5, 128.4, 128.4, 128.3, 128.1, 126.0, 126.0, 121.0 (t, J_{C-F} = 2.9 Hz), 120.9 (t, J_{C-F} = 245.2 Hz), 120.8 (t, J_{C-F} = 2.9 Hz), 120.6 (t, J_{C-F} = 245.2 Hz), 68.2 (t, J_{C-F} = 35.6 Hz), 40.3, 40.1, 38.2 (t, J_{C-F} = 23.0 Hz; 2C), 21.7 (2C), 20.1 (t, J_{C-F} = 1.8 Hz), 20.0 (t, J_{C-F} = 1.8 Hz),

19.2, 18.8. ^{19}F-NMR (376 MHz, $CDCl_3$): δ = -102.51 (s), -102.71 (s). IR (ATR): 2923, 1729, 1533, 1277, 1082, 926 cm^{-1}. HR-MS (EI) m/z calcd for $C_{21}H_{25}F_2O_3S$ [M+H$^+$] 395.1492, found 395.1499.

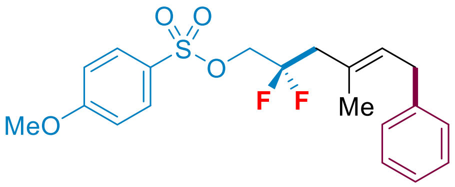

(*E*)-2,2-Difluoro-4-methyl-6-phenylhex-4-en-1-yl 4-methoxybenzenesulfonate (74)

The general procedure **TP3** was followed using **1i** (83 mg, 0.25 mmol), **2z** (34 mg, 0.5 mmol) and **3a** (0.5 mmol) for 12 h. Purification by column chromatography (*n*-hexane/EtOAc 40:1) yielded **74** (88 mg, 89%) as an oil.^{1}H-NMR (400 MHz, $CDCl_3$): δ = 7.84 – 7.79 (m, 2H), 7.32 – 7.26 (m, 2H), 7.23 – 7.13 (m, 3H), 7.00 – 6.96 (m, 2H), 5.51 (t, *J* = 7.1 Hz, 1H), 4.08 (t, *J* = 11.5 Hz, 2H), 3.87 (s, 3H), 3.36 (d, *J* = 7.4 Hz, 2H), 2.62 (t, *J* = 16.7 Hz, 2H), 1.77 (s, 3H). ^{13}C-NMR (100 MHz, $CDCl_3$): δ = 164.1, 141.4, 140.5, 139.3, 130.9, 130.3, 128.5, 128.3, 126.0, 120.4 (t, $J_{C\text{-}F}$ = 245.4 Hz), 114.6, 67.5 (t, $J_{C\text{-}F}$ = 35.6 Hz), 55.8, 43.2 (t, $J_{C\text{-}F}$ = 22.9 Hz), 34.3, 17.3. ^{19}F-NMR (376 MHz, $CDCl_3$): δ = -102.90 (s), -104.36 (s). IR (ATR): 2933, 1716, 1498, 1275, 933, 782 cm^{-1}. HR-MS (EI) m/z calcd for $C_{20}H_{23}F_2O_4S$ [M+H$^+$] 397.1285, found 397.1297.

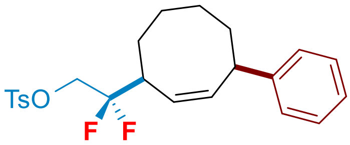

2,2-Difluoro-2-[(*trans*, *Z*)-4-phenylcyclooct-2-en-1-yl]ethyl 4-methylbenzenesulfonate (75)

The general procedure **TP3** was followed using **1k** (79 mg, 0.25 mmol), **2aa** (54 mg, 0.5 mmol) and **3a** (0.5 mmol) for 12 h. Purification by column chromatography (*n*-hexane/EtOAc 40:1) yielded **75** (57 mg, 54%) as an oil. ^{1}H-NMR (400 MHz, $CDCl_3$): δ = 7.81 (d, *J* = 8.3 Hz, 2H), 7.38 – 7.28 (m, 4H), 7.25 – 7.17 (m, 3H), 5.84 (dd, *J* = 11.9, 5.3 Hz, 1H), 5.33 (dd, *J* = 11.5, 7.5 Hz, 1H), 4.25 – 4.15 (m, 2H), 3.70 – 3.49 (m, 2H), 2.45 (s, 3H), 2.22 – 2.09 (m, 1H), 1.83 – 1.71 (m, 4H), 1.70 – 1.65 (m, 1H). ^{13}C-NMR (100 MHz, $CDCl_3$):

δ = 145.5, 145.4, 137.1, 132.3, 130.0, 128.6, 128.1, 127.5, 126., 121.4 (t, J_{C-F} = 245.1 Hz), 121.3 (dd, J_{C-F} = 7.3, 4.0 Hz), 67.4 (d, J_{C-F} = 37.3 Hz), 47.5, 40.7 (t, J_{C-F} = 21.4 Hz), 34.6, 27.3, 25.5 (t, J_{C-F} = 4.2 Hz), 23.0, 21.7. ^{19}F-NMR (376 MHz, $CDCl_3$): δ = -112.07 (d, J = 255.5 Hz), -115.42 (d, J = 255.6 Hz). IR (ATR): 3321, 2920, 1723, 1644, 1477, 1236, 1162, 858 cm^{-1}. HR-MS (EI) m/z calcd for $C_{23}H_{27}F_2O_3S$ [M+H$^+$] 421.1649, found 421.1655.

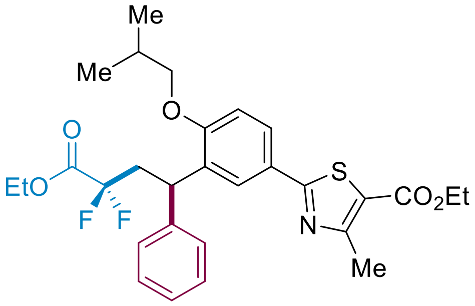

Ethyl 2-[3-(4-ethoxy-3,3-difluoro-4-oxo-1-phenylbutyl)-4-isobutoxyphenyl]-4-methylthiazole-5-carboxylate (**76**)

The general procedure **TP2** was followed using **1a** (101 mg, 0.5 mmol), **2q** (86 mg, 0.25 mmol) and **3a** (0.5 mmol) for 3 h. Purification by column chromatography (*n*-hexane/EtOAc 40:1→20:1) yielded **76** (93 mg, 68%) as an oil. ^{1}H-NMR ($CDCl_3$, 400 MHz): δ = 7.80 (d, J = 2.2 Hz, 1H), 7.70 (dd, J = 8.6, 2.2 Hz, 1H), 7.27 – 7.15 (m, 4H), 7.10 (ddd, J = 7.1, 3.9, 1.3 Hz, 1H), 6.76 (d, J = 8.6 Hz, 1H), 4.70 (t, J = 7.3 Hz, 1H), 4.27 (q, J = 7.1 Hz, 2H), 3.78 (q, J = 7.0 Hz, 2H), 3.72 – 3.62 (m, 2H), 2.98 – 2.83 (m, 2H), 2.69 (s, 3H), 2.05 (dt, J = 13.2, 6.6 Hz, 1H), 1.31 (t, J = 7.1 Hz, 3H), 1.08 (t, J = 7.2 Hz, 3H), 0.97 (dd, J = 8.1, 6.8 Hz, 6H). ^{13}C-NMR ($CDCl_3$, 100 MHz): δ = 169.9, 163.8 (t, J_{C-F} = 32.6 Hz), 162.4, 161.1, 158.7, 141.9, 132.1, 128.4, 128.2, 126.9, 126.7, 126.5, 125.4, 120.8, 115.7 (t, J_{C-F} = 250.3 Hz), 111.7, 74.9, 62.7, 61.2, 39.3 (t, J_{C-F} = 23.3 Hz), 37.9 (t, J_{C-F} = 5.2 Hz), 28.3, 19.4, 19.3, 17.6, 14.4, 13.7. ^{19}F-NMR ($CDCl_3$, 376 MHz): δ = -102.59 (d, J = 259.8 Hz), -104.61 (d, J = 259.8 Hz). IR (ATR): 3329, 2932, 1672, 1457, 1282, 873 cm^{-1}. HR-MS (ESI) m/z calcd for $C_{29}H_{34}F_2O_5S$ [M+H$^+$] 546.2126, found 546.2128 [M+H$^+$].

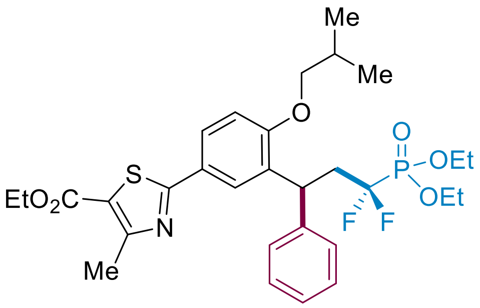

Ethyl 2-{3-[3-(diethoxyphosphoryl)-3,3-difluoro-1-phenylpropyl]-4-isobutoxyphenyl}-4-methylthiazole-5-carboxylate (**77**)

The general procedure **TP2** was followed using **1g** (133 mg, 0.5 mmol), **2q** (86 mg, 0.25 mmol) and **3a** (0.5 mmol) for 3 h. Purification by column chromatography (*n*-hexane/EtOAc 20:1→10:1) yielded **77** (114 mg, 75%) as an oil. ^{1}H-NMR ($CDCl_3$, 400 MHz): δ = 7.90 (d, *J* = 2.2 Hz, 1H), 7.77 (dd, *J* = 8.6, 2.2 Hz, 1H), 7.32 (dd, *J* = 13.0, 7.7 Hz, 2H), 7.28 – 7.21 (m, 2H), 7.16 (t, *J* = 7.2 Hz, 1H), 6.84 (d, *J* = 8.6 Hz, 1H), 4.98 (t, *J* = 7.0 Hz, 1H), 4.34 (q, *J* = 7.1 Hz, 2H), 4.24 (pd, *J* = 7.2, 1.9 Hz, 4H), 3.79 – 3.70 (m, 2H), 3.11 – 2.83 (m, 2H), 2.76 (s, 3H), 2.12 (dt, *J* = 13.2, 6.6 Hz, 1H), 1.41 – 1.31 (m, 9H), 1.03 (t, *J* = 7.0 Hz, 6H). ^{13}C-NMR ($CDCl_3$, 100 MHz): δ = 170.1, 162.5, 161.0, 158.6, 143.1, 132.9, 128.3, 127.9, 126.8, 126.7, 126.4, 125.4, 120.5 (td, J_{C-F} = 261.5, 254.5 Hz), 120.7, 111.7, 74.9, 64.5 (t, J_{C-F} = 6.6 Hz), 61.1, 38.0 (dd, J_{C-F} = 34.2, 19.6 Hz), 37.1 (dt, J_{C-F} = 7.1 , 3.1 Hz), 28.3, 19.4, 19.3, 17.6, 16.4, 16.4, 14.4. ^{19}F-NMR ($CDCl_3$, 376 MHz): δ = -110.74 (d, *J* = 108.1 Hz), -111.58 (dd, *J* = 107.9, 40.1 Hz), -112.42 (d, *J* = 107.7 Hz). IR (ATR): 3372, 2935, 1683, 1474, 1281, 963 cm^{-1}. HR-MS (ESI) m/z calcd for $C_{30}H_{39}F_2NO_6PS$ [M+H$^+$] 610.2204, found 610.2209 [M+H$^+$].

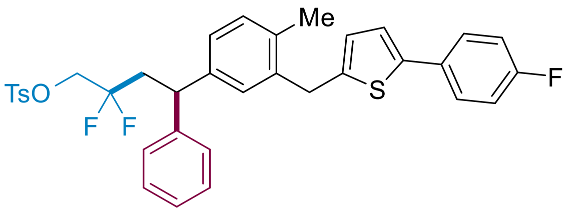

2,2-Difluoro-4-(3-{[5-(4-fluorophenyl)thiophen-2-yl]methyl}-4-methylphenyl)-4-phenylbutyl 4-methylbenzenesulfonate (**78**)

The general procedure **TP2** was followed using **1a** (101 mg, 0.5 mmol), **2r** (77 mg, 0.25 mmol) and **3a** (0.5 mmol) for 3 h. Purification by column chromatography (*n*-hexane/EtOAc 20:1) yielded **78** (122 mg, 96%) as an oil. ^{1}H-NMR ($CDCl_3$, 400 MHz): δ =

7.73 (d, J = 8.2 Hz, 2H), 7.50 (dd, J = 8.6, 5.3 Hz, 2H), 7.36 – 7.28 (m, 3H), 7.28 – 7.16 (m, 4H), 7.11 (d, J = 7.4 Hz, 2H), 7.09 – 7.01 (m, 4H), 6.62 (d, J = 3.4 Hz, 1H), 4.24 (t, J = 7.4 Hz, 1H), 4.10 (s, 2H), 3.91 (t, J = 11.9 Hz, 2H), 2.74 (td, J = 16.2, 7.4 Hz, 2H), 2.46 (s, 3H), 2.28 (s, 3H). ^{13}C-NMR ($CDCl_3$, 100 MHz): δ = 162.1 (d, J_{C-F} = 246.7 Hz), 145.4, 143.5, 143.4, 141.5, 141.2, 138.4, 134.9, 132.1, 130.8, 130.0, 128.9, 128.7, 128.0, 127.5, 127.1 (d, J_{C-F} = 8.0 Hz), 126.6, 126.0, 125.9, 122.7, 120.3 (t, J_{C-F} = 245.6 Hz), 115.7 (d, J_{C-F} = 21.7 Hz), 68.2 (t, J_{C-F} = 35.0 Hz), 44.4 (t, J_{C-F} = 4.7 Hz), 39.2 (t, J_{C-F} = 22.8 Hz), 34.2, 21.7, 19.0. ^{19}F-NMR ($CDCl_3$, 376 MHz): δ = -102.19 (s), -115.20 (s). IR (ATR): 2922, 1697, 1474, 1366, 1182 cm^{-1}. HR-MS (ESI) m/z calcd for $C_{35}H_{32}F_3O_3S$ [M+H$^+$] 621.1745, found 621.1757 [M+H$^+$].

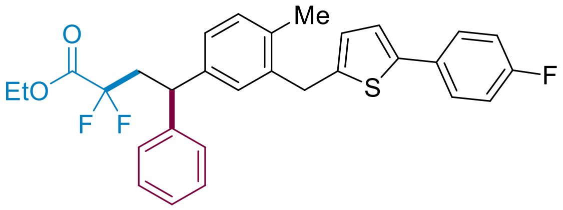

Ethyl 2,2-difluoro-4-(3-{[5-(4-fluorophenyl)thiophen-2-yl]methyl}-4-methylphenyl)-4-phenylbutanoate (79)

The general procedure **TP2** was followed using **1a** (154 mg, 0.5 mmol), **2r** (234 mg, 0.75 mmol) and **3a** (1.0 mmol) for 16 h. Purification by column chromatography (*n*-hexane/EtOAc 20:1) yielded **79** (282 mg, 91%) as an oil. ^{1}H-NMR ($CDCl_3$, 400 MHz): δ = 7.54 – 7.47 (m, 2H), 7.33 – 7.25 (m, 4H), 7.24 – 7.18 (m, 1H), 7.17 – 7.03 (m, 6H), 6.65 (d, J = 3.6 Hz, 1H), 4.28 (t, J = 7.3 Hz, 1H), 4.10 (s, 2H), 3.96 – 3.84 (m, 2H), 3.06 – 2.84 (m, 2H), 2.28 (s, 3H), 1.19 (t, J = 7.2 Hz, 3H). ^{13}C-NMR ($CDCl_3$, 100 MHz): δ = 163.8 (t, J_{C-F} = 32.6 Hz), 162.1 (d, J_{C-F} = 246.6 Hz), 143.3, 143.1, 141.5, 141.1, 138.3, 134.8, 130.9 (d, J_{C-F} = 3.4 Hz), 130.7, 129.0, 128.6, 127.8, 127.1 (d, J_{C-F} = 8.0 Hz), 126.7, 126.1, 125.9, 122.7 (d, J_{C-F} = 1.0 Hz), 115.7 (t, J_{C-F} = 250.9 Hz), 115.7 (d, J_{C-F} = 21.6 Hz), 62.7, 44.4 (t, J_{C-F} = 4.6 Hz), 40.3 (t, J_{C-F} = 23.1 Hz), 34.2, 19.0, 13.7. ^{19}F-NMR ($CDCl_3$, 376 MHz): δ = -102.09 (dd, J = 15.7, 14.1 Hz), -102.71 – -102.86 (m), -104.10 (dd, J = 17.6, 14.6 Hz), -104.79 (dd, J = 17.8, 14.5 Hz), -115.19 (tt, J = 8.6, 5.3 Hz). IR (ATR): 2920, 1697, 1476, 1268, 1183 cm^{-1}. HR-MS (ESI) m/z calcd for $C_{30}H_{28}F_3O_2S$ [M+H$^+$] 509.1762, found 509.1765 [M+H$^+$].

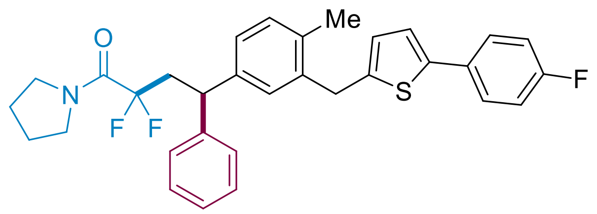

2,2-Difluoro-4-(3-{[5-(4-fluorophenyl)thiophen-2-yl]methyl}-4-methylphenyl)-4-phenyl-1-(pyrrolidin-1-yl)butan-1-one (80)

The general procedure **TP2** was followed using **1c** (114 mg, 0.5 mmol), **2r** (77 mg, 0.25 mmol) and **3a** (0.5 mmol) for 3 h. Purification by column chromatography (*n*-hexane/EtOAc 20:1) yielded **80** (111 mg, 83%) as an oil. ^{1}H-NMR ($CDCl_3$, 400 MHz): δ = 7.54 – 7.45 (m, 2H), 7.35 – 7.26 (m, 4H), 7.23 – 7.16 (m, 2H), 7.15 – 7.09 (m, 2H), 7.09 – 7.01 (m, 3H), 6.64 (d, J = 3.6 Hz, 1H), 4.39 (t, J = 7.2 Hz, 1H), 4.10 (s, 2H), 3.53 (t, J = 6.7 Hz, 2H), 3.38 (t, J = 6.9 Hz, 2H), 3.14 – 2.89 (m, 2H), 2.28 (s, 3H), 1.89 – 1.69 (m, 4H). ^{13}C-NMR ($CDCl_3$, 100 MHz): δ = 162.1 (t, J_{C-F} = 29.7 Hz), 162.1 (d, J_{C-F} = 246.6 Hz), 144.0, 143.5, 141.9, 141.4, 138.2, 134.5, 130.9 (d, J_{C-F} = 3.4 Hz), 130.6, 129.1, 128.5, 127.7, 127.1 (d, J_{C-F} = 7.9 Hz), 126.4, 126.2, 125.9, 122.7, 118.6 (t, J_{C-F} = 254.0 Hz), 115.7 (d, J_{C-F} = 21.7 Hz), 47.4, 46.5 (t, J_{C-F} = 6.5 Hz), 44.4 (t, J_{C-F} = 3.8 Hz), 40.0 (t, J_{C-F} = 22.5 Hz), 34.2, 23.1, 19.0. ^{19}F-NMR ($CDCl_3$, 376 MHz): δ = -99.59 – -101.71 (m), -115.25 (tt, J = 8.6, 5.3 Hz). IR (ATR): 3332, 2923, 1759, 1546, 1251, 1128, 1063 cm^{-1}. HR-MS (ESI) m/z calcd for $C_{32}H_{31}F_3NOS$ [M+H$^+$] 534.2078, found 534.2082 [M+H$^+$].

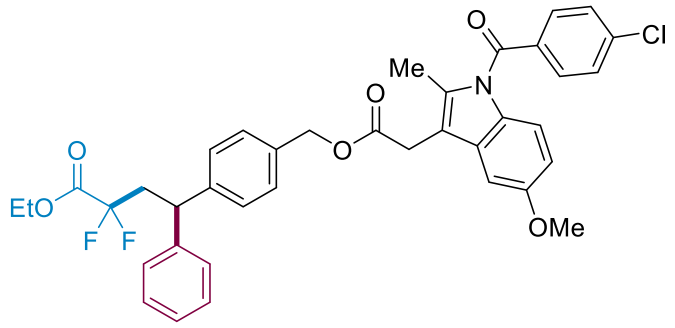

Ethyl 4-(4-({2-[1-(4-chlorobenzoyl)-5-methoxy-2-methyl-1*H*-indol-3-yl]acetoxy}methyl)phenyl)-2,2-difluoro-4-phenylbutanoate (81)

The general procedure **TP2** was followed using **1a** (101 mg, 0.5 mmol), **2s** (118 mg, 0.25 mmol) and **3a** (0.5 mmol) for 3 h. Purification by column chromatography (*n*-hexane/EtOAc 20:1) yielded **81** (50 mg, 30%) as an oil. ^{1}H-NMR ($CDCl_3$, 400 MHz): δ =

7.70 – 7.64 (m, 2H), 7.52 – 7.45 (m, 2H), 7.36 – 7.17 (m, 9H), 6.93 (d, *J* = 2.5 Hz, 1H), 6.89 (d, *J* = 9.0 Hz, 1H), 6.69 (dd, *J* = 9.0, 2.5 Hz, 1H), 5.10 (s, 2H), 4.30 (t, *J* = 7.3 Hz, 1H), 3.87 (q, *J* = 7.2 Hz, 2H), 3.72 (d, *J* = 9.6 Hz, 5H), 3.06 – 2.84 (m, 2H), 2.39 (s, 3H), 1.19 (dd, *J* = 7.6, 6.7 Hz, 3H). ^{13}C-NMR (CDCl$_3$, 100 MHz): δ = 170.7, 168.3, 163.4 (t, J_{C-F} = 32.5 Hz), 156.0, 143.3, 142.6, 139.3, 136.0, 134.2, 133.9, 131.2, 130.8, 130.6, 129.2, 128.7, 128.5, 127.9, 127.7, 127.0, 115.6 (t, J_{C-F} = 251.0 Hz), 115.0, 112.4, 111.9, 101.1, 66.4, 62.8, 55.6, 44.6 (t, J_{C-F} = 4.6 Hz), 40.1 (t, J_{C-F} = 23.3 Hz), 30.4, 13.7, 13.4. ^{19}F-NMR (CDCl$_3$, 376 MHz): δ = -101.84 – -105.26 (m). IR (ATR): 3317, 2982, 1743, 1677, 1457, 1217, 845 cm^{-1}. HR-MS (ESI) m/z calcd for $C_{38}H_{35}ClF_2NO_6$ [M+H$^+$] 674.2121, found 674.2127 [M+H$^+$].

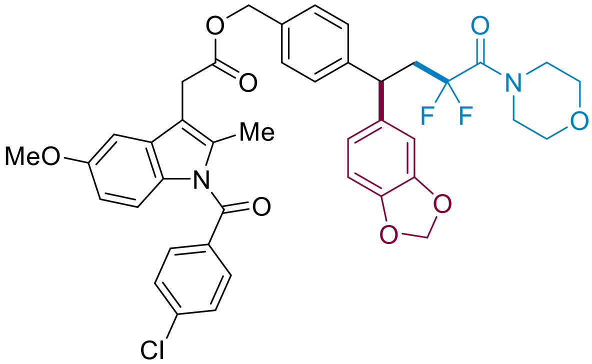

4-[1-(Benzo[*d*][1,3]dioxol-5-yl)-3,3-difluoro-4-morpholino-4-oxobutyl]benzyl 2-[1-(4-chlorobenzoyl)-5-methoxy-2-methyl-1*H*-indol-3-yl]acetate (82)

The general procedure **TP2** was followed using **1b** (122 mg, 0.5 mmol), **2s** (118 mg, 0.25 mmol) and **3h** (0.5 mmol) for 3 h. Purification by column chromatography (*n*-hexane/EtOAc 20:1→10:1) yielded **82** (57 mg, 30%) as an oil. ^{1}H-NMR (CDCl$_3$, 400 MHz): δ = 7.71 – 7.63 (m, 2H), 7.52 – 7.44 (m, 2H), 7.25 (s, 4H), 6.93 (d, *J* = 2.5 Hz, 1H), 6.89 (d, *J* = 9.0 Hz, 1H), 6.75 (s, 3H), 6.68 (dd, *J* = 9.0, 2.5 Hz, 1H), 5.93 (dd, *J* = 2.3, 1.4 Hz, 2H), 5.11 (s, 2H), 4.36 (t, *J* = 6.9 Hz, 1H), 3.75 (s, 3H), 3.75 – 3.54 (m, 10H), 2.97 (tdd, *J* = 18.3, 6.9, 2.3 Hz, 2H), 2.38 (s, 3H). ^{13}C-NMR (CDCl$_3$, 100 MHz): δ = 170.7, 168.3, 161.8 (t, J_{C-F} = 29.2 Hz), 156.0, 147.8, 146.1, 144.5, 139.3, 137.9, 136.0, 133.9, 133.9, 131.2, 130.8, 130.6, 129.1, 128.5, 127.7, 120.6, 117.7 (d, J_{C-F} = 256.0 Hz), 115.0, 112.5, 111.9, 108.3, 108.1, 101.1, 101.0, 66.7, 66.7, 66.5, 55.6, 46.5 (t, J_{C-F} = 6.0 Hz), 44.0, 43.3, 40.1 (t, J_{C-F} = 21.9 Hz), 30.4, 13.4. ^{19}F-NMR (CDCl$_3$, 376 MHz): δ = -97.94 (t, *J* = 18.2 Hz). IR (ATR): 3323, 2921, 1687, 1468,

1274, 1183 cm^{-1}. HR-MS (ESI) m/z calcd for $C_{41}H_{38}ClF_2N_2O_8$ [M+H+] 759.2285, found 759.2286 [M+H+].

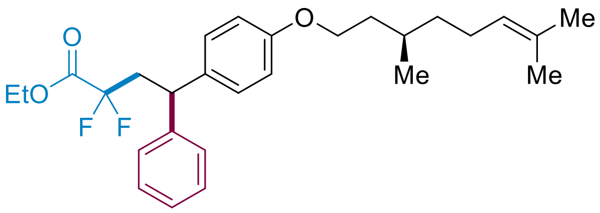

Ethyl 4-{4-[(*R*)-3,7-dimethyloct-6-enyloxy]phenyl}-2,2-difluoro-4-phenylbutanoate (83)

The general procedure **TP2** was followed using **1a** (101 mg, 0.5 mmol), **2t** (65 mg, 0.25 mmol) and **3a** (0.5 mmol) for 3 h. Purification by column chromatography (*n*-hexane/EtOAc 20:1) yielded **83** (65 mg, 57%) as an oil. ^{1}H-NMR ($CDCl_3$, 400 MHz): δ = 7.35 (d, *J* = 4.2 Hz, 1H), 7.32 – 7.24 (m, 3H), 7.23 – 7.13 (m, 3H), 6.83 (d, *J* = 8.5 Hz, 2H), 5.12 (t, *J* = 6.8 Hz, 1H), 4.25 (t, *J* = 7.1 Hz, 1H), 3.96 (dd, *J* = 11.4, 5.8 Hz, 2H), 3.88 (q, *J* = 7.2 Hz, 2H), 2.92 (td, *J* = 15.3, 7.4 Hz, 2H), 2.07 – 1.97 (m, 2H), 1.82 (dt, *J* = 12.8, 6.8 Hz, 1H), 1.76 – 1.65 (m, 5H), 1.62 (s, 3H), 1.41 (dddd, *J* = 19.5, 15.4, 11.8, 7.3 Hz, 2H), 1.20 (t, *J* = 7.2 Hz, 3H), 0.96 (d, *J* = 6.4 Hz, 3H). ^{13}C-NMR ($CDCl_3$, 100 MHz): δ = 163.8 (t, J_{C-F} = 32.6 Hz), 157.9, 143.4, 134.8, 131.3, 128.7, 128.6, 127.6, 126.6, 124.7, 115.7 (t, J_{C-F} = 250.6 Hz), 114.5, 66.3, 62.7, 44.0 (t, J_{C-F} = 4.7 Hz), 40.5 (t, J_{C-F} = 23.2 Hz), 37.1, 36.1, 29.5, 25.7, 25.5, 19.5, 17.7, 13.7. ^{19}F-NMR ($CDCl_3$, 376 MHz): δ = -103.45 (q, *J* = 15.1 Hz). IR (ATR): 3376, 2929, 1682, 1437, 1264, 1096 cm^{-1}. HR-MS (ESI) m/z calcd for $C_{28}H_{37}F_2O_3$ [M+H+] 459.2711, found 459.2718 [M+H+].

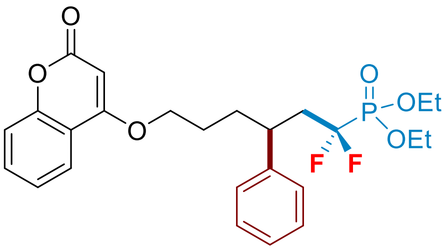

Diethyl [difluoro(1-phenyl-2,3-dihydro-1*H*-inden-2-yl)methyl]phosphonate (84)

The general procedure **TP2** was followed using **1g** (133 mg, 0.5 mmol), **2u** (58 mg, 0.25 mmol) and **3a** (0.5 mmol) for 3 h. Purification by column chromatography (*n*-hexane/EtOAc 10:1→5:1) yielded **84** (33 mg, 27%) as an oil. ^{1}H-NMR ($CDCl_3$, 400 MHz): δ

= 7.77 (dd, J = 7.9, 1.5 Hz, 1H), 7.59 – 7.50 (m, 1H), 7.36 – 7.29 (m, 3H), 7.28 – 7.19 (m, 4H), 5.59 (s, 1H), 4.25 (dqd, J = 10.8, 7.1, 3.5 Hz, 4H), 4.02 (ddd, J = 9.5, 6.5, 3.3 Hz, 2H), 3.19 (td, J = 10.8, 6.4 Hz, 1H), 2.55 – 2.38 (m, 2H), 2.14 – 2.04 (m, 1H), 1.84 (ddd, J = 16.4, 12.0, 6.9 Hz, 2H), 1.73 (s, 1H), 1.36 (td, J = 7.1, 2.8 Hz, 6H). ^{13}C-NMR ($CDCl_3$, 100 MHz): δ = 165.6, 163.0, 153.3, 143.9, 132.4, 128.7, 127.4, 126.8, 123.9, 123.0, 122.0 (td, J_{C-F} = 261.8, 216.3 Hz), 116.8, 115.7, 90.4, 69.0, 64.6 (dd, J_{C-F} = 6.9, 2.5 Hz), 40.3 (td, J_{C-F} = 19.7, 14.1 Hz), 38.5 (p, J_{C-F} = 3.1 Hz), 33.1, 29.7, 26.4, 16.4, 16.4. ^{19}F-NMR ($CDCl_3$, 376 MHz): δ = -109.86 (d, J = 108.1 Hz), -110.56 (d, J = 40.0 Hz), -110.84 (d, J = 40.0 Hz), -111.6 (d, J = 108.1 Hz). IR (ATR): 3367, 2921, 1684, 1439, 1282, 762 cm^{-1}. HR-MS (ESI) m/z calcd for $C_{25}H_{30}F_3O_6P$ [M+H$^+$] 495.1748, found 495.1751 [M+H$^+$].

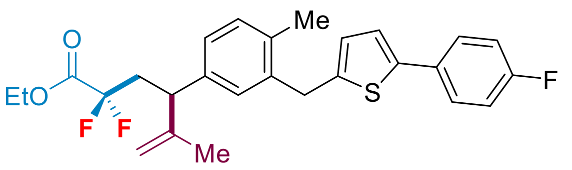

Ethyl 2,2-difluoro-4-{3-[(5-(4-fluorophenyl)thiophen-2-yl)methyl]-4-methylphenyl}-5-methylhex-5-enoate (85)

The general procedure **TP2** was followed using **1a** (101 mg, 0.5 mmol), **2r** (77 mg, 0.25 mmol) and **3u** (0.5 mmol) for 1 h. Thereafter a second-round of **1a** (101 mg, 0.5 mmol) and **3u** (0.5 mmol) was added to the reaction, and the reaction mixture stirred at room temperature for another 3 h. Purification by column chromatography (*n*-hexane/EtOAc 40:1) yielded **85** (25 mg, 21%) as an oil. ^{1}H-NMR ($CDCl_3$, 400 MHz): δ = 7.49 – 7.45 (m, 2H), 7.10 (d, J = 7.7 Hz, 1H), 7.06 (d, J = 1.7 Hz, 1H), 7.02 (dd, J = 4.9, 3.6 Hz, 4H), 6.62 (d, J = 3.6 Hz, 1H), 4.91 (s, 1H), 4.86 – 4.81 (m, 1H), 4.09 (s, 2H), 4.04 (dtd, J = 10.7, 7.2, 3.6 Hz, 2H), 3.54 (t, J = 7.2 Hz, 1H), 2.75 – 2.52 (m, 2H), 2.27 (s, 3H), 1.60 (s, 3H), 1.24 (t, J = 7.2 Hz, 3H). ^{13}C-NMR ($CDCl_3$, 100 MHz): δ = 164.0 (t, J_{C-F} = 32.6 Hz), 162.1 (d, J_{C-F} = 246.4 Hz), 146.5, 143.5, 141.4, 139.3, 138.0, 135.0, 130.9 (d, J_{C-F} = 3.7 Hz), 130.5, 129.3, 127.1 (d, J_{C-F} = 7.9 Hz), 126.3, 125.8, 122.7, 115.9 (t, J_{C-F} = 254.0 Hz), 115.7 (d, J_{C-F} = 21.9 Hz), 111.0, 62.7, 45.6, 37.9, 34.2, 20.9, 19.0, 13.8. ^{19}F-NMR ($CDCl_3$, 376 MHz): δ = -102.7 (d, J = 259.6 Hz), -104.09 (d, J = 259.6 Hz), -115.24 (s). IR (ATR): 2917, 1762, 1544, 1246, 1152, 1067,

1012, 772 cm^{-1}. HR-MS (ESI) m/z calcd for $C_{27}H_{28}F_3O_3S$ [M+H$^+$] 473.1762, found 473.1764 [M+H$^+$].

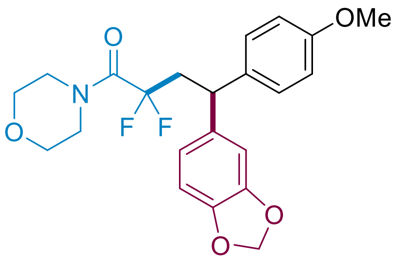

4-(Benzo[*d*][1,3]dioxol-5-yl)-2,2-difluoro-4-(4-methoxyphenyl)-1-morpholinobutan-1-one (95)

A suspension of $CoBr_2$ (87 mg, 10 mol %), 4-methyloxystyrene (536 mg, 4 mmol), bromodifluoroactamide (1.944 g, 2.0 equiv), aryl zinc pivalates (8 mmol, 2.0 equiv) in degas MeCN (16 mL) was stirred at room temperature for 12 h under an atmosphere of Ar. At ambient temperature, the solvent was evaporated *in vacuo* and the remaining residue was purified by column chromatography on silica gel (*n*-hexane/EtOAc 10:1→5:1) to yield product **95** (1.089 g, 62%) as an oil. ^{1}H-NMR ($CDCl_3$, 400 MHz): δ = 7.24 – 7.17 (m, 2H), 6.88 – 6.82 (m, 2H), 6.75 (dd, J = 4.4, 3.5 Hz, 3H), 5.92 (q, J = 1.4 Hz, 2H), 4.32 (t, J = 6.9 Hz, 1H), 3.79 (s, 3H), 3.65 (qd, J = 9.5, 4.5 Hz, 8H), 2.95 (tdd, J = 18.4, 7.0, 2.3 Hz, 2H). ^{13}C-NMR ($CDCl_3$, 100 MHz): δ = 161.9 (t, J_{C-F} = 29.3 Hz), 158.1, 147.7, 146.0, 138.6, 136.3, 128.5, 120.5, 119.0 (d, J_{C-F} = 255.7 Hz), 113.9, 108.2, 108.1, 101.0, 66.7, 66.7, 55.3, 46.5 (t, J_{C-F} = 6.2 Hz), 43.4 (t, J_{C-F} = 3.4 Hz), 43.3, 40.4 (t, J_{C-F} = 21.7 Hz). ^{19}F-NMR ($CDCl_3$, 376 MHz): δ = -97.92 (t, J = 18.2 Hz). IR (ATR): 3332, 2917, 1673, 1464, 1252, 1171 cm^{-1}. HR-MS (ESI) m/z calcd for $C_{22}H_{24}F_2NO_5$ [M+H$^+$] 420.2623, found 420.2624 [M+H$^+$].

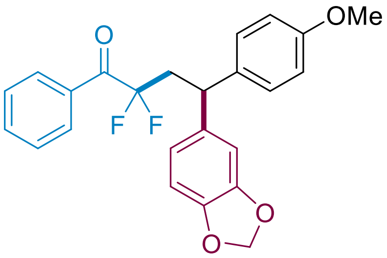

4-(Benzo[*d*][1,3]dioxol-5-yl)-2,2-difluoro-4-(4-methoxyphenyl)-1-phenylbutan-1-one (97a)

To a suspension of **95** (105 mg, 0.25 mmol) in anhydrous THF (4 mL) was dropwise added PhMgCl (2.0 M, 0.625 mmol) at 0 °C. The reaction mixture was stirred at 0 °C under an atmosphere of N_2 for 2 h. After filtration and removal of the solvents *in vacuo*, the residue was purified by column chromatography (*n*-Hex/EtOAc = 40:1) to give **97a** (57.4 mg, 56%) as an oil. ^{1}H-NMR ($CDCl_3$, 400 MHz): δ = 7.97 (d, J = 7.5 Hz, 2H), 7.67 – 7.58 (m, 1H), 7.45 (dd, J = 10.8, 4.9 Hz, 2H), 7.22 – 7.12 (m, 2H), 6.84 – 6.78 (m, 2H), 6.76 – 6.67 (m, 3H), 5.91 (s, 2H), 4.32 (t, J = 7.2 Hz, 1H), 3.78 (s, 3H), 3.00 (td, J = 17.2, 7.2 Hz, 2H). ^{13}C-NMR ($CDCl_3$, 100 MHz): δ = 189.1 (t, J_{C-F} = 30.5 Hz), 158.2, 147.7, 146.1, 138.0, 135.8, 134.2, 132.0 (t, J_{C-F} = 2.2 Hz), 130.0 (t, J_{C-F} = 3.3 Hz), 129.7, 128.5, 120.6, 119.2 (t, J_{C-F} = 254.9 Hz), 113.9, 108.2, 108.1, 101.0, 55.2, 43.5 (t, J_{C-F} = 3.9 Hz), 40.0 (t, J_{C-F} = 22.1 Hz). ^{19}F-NMR ($CDCl_3$, 376 MHz): δ = -98.34 (td, J = 17.1, 6.0 Hz). IR (ATR): 2976, 1682, 1457, 1266, 767 cm^{-1}. HR-MS (ESI) m/z calcd for $C_{24}H_{21}F_2O_4$ [M+H$^+$] 411.1408, found 411.1412 [M+H$^+$].

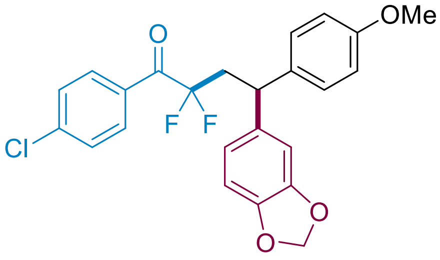

4-(Benzo[*d*][1,3]dioxol-5-yl)-1-(4-chlorophenyl)-2,2-difluoro-4-(4-methoxyphenyl)butan-1-one (**97b**)

To a suspension of **95** (105 mg, 0.25 mmol) in anhydrous THF (4 mL) was dropwise added ArMgCl (0.74 M, 0.625 mmol) at 0 °C. The reaction mixture was stirred at 0 °C under an atmosphere of N_2 for 2 h. After filtration and removal of the solvents *in vacuo*, the residue was purified by column chromatography (*n*-Hex/EtOAc = 40:1) to give **98b** (59 mg, 53%) as an oil. ^{1}H-NMR ($CDCl_3$, 400 MHz): δ = 7.90 (d, J = 8.7 Hz, 2H), 7.46 – 7.40 (m, 2H), 7.19 – 7.12 (m, 2H), 6.85 – 6.77 (m, 2H), 6.74 – 6.65 (m, 3H), 5.91 (s, 2H), 4.29 (t, J = 7.2 Hz, 1H), 3.78 (s, 3H), 2.99 (td, J = 17.3, 7.2 Hz, 2H). ^{13}C-NMR ($CDCl_3$, 100 MHz): δ = 188.0 (t, J_{C-F} = 30.9 Hz), 158.2, 147.7, 146.1, 140.8, 137.8, 135.6, 131.4 (t, J_{C-F} = 3.4 Hz), 130.3, 128.9, 128.5, 120.6, 119.2 (t, J_{C-F} = 254.1 Hz), 113.9 108.2, 108.1, 101.0, 55.3, 43.5 (t, J_{C-F} = 3.9 Hz), 40.0 (t, J_{C-F} = 22.0 Hz). ^{19}F-NMR ($CDCl_3$, 376 MHz): δ = -98.25 (td, J = 17.1, 10.6 Hz). IR

(ATR): 2967, 1715, 1673, 1477, 1264, 677 cm^{-1}. HR-MS (ESI) m/z calcd for $C_{24}H_{20}ClF_2O_4$ [M+H$^+$] 445.1018, found 445.1023 [M+H$^+$].

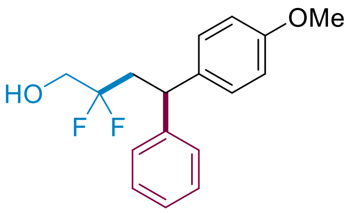

2,2-Difluoro-4-(4-methoxyphenyl)-4-phenylbutan-1-ol (**98**)

To a suspension of **4** (167 mg, 0.5 mmol) in EtOH (10 mL) was added $NaBH_4$ (285 mg, 15 equiv or 38 mg, 2.0 equiv) at room temperature. The reaction mixture was stirred at 90 °C under an atmosphere of N_2 for 3 h. After filtration and removal of the solvents *in vacuo*, the residue was purified by column chromatography (*n*-Hex/EtOAc = 8:1) to give **98** (143 mg, 98% or 134 mg, 92%) as an oil. ^{1}H-NMR ($CDCl_3$, 400 MHz): δ = 7.36 – 7.29 (m, 4H), 7.27 – 7.19 (m, 3H), 6.90 – 6.83 (m, 2H), 4.31 (t, J = 7.3 Hz, 1H), 3.79 (s, 3H), 3.52 (t, J = 13.0 Hz, 2H), 2.78 (td, J = 16.1, 7.3 Hz, 2H), 1.76 (br s, 1H). ^{13}C-NMR ($CDCl_3$, 100 MHz): δ = 158.2, 144.3, 136.0, 128.7, 128.6, 127.5, 126.6, 123.0 (t, J_{C-F} = 243.1 Hz), 114.1, 64.1 (t, J_{C-F} = 31.7 Hz), 55.3, 44.3 (t, J_{C-F} = 4.6 Hz), 39.2 (t, J_{C-F} = 23.6 Hz). ^{19}F-NMR ($CDCl_3$, 376 MHz): δ = -104.21 – -104.39 (m), -104.97 (dq, J = 16.0, 13.2 Hz), -105.14 (tt, J = 16.2, 13.0 Hz), -105.81 (tt, J = 16.3, 13.0 Hz). IR (ATR): 2987, 1643, 1468, 1276, 762 cm^{-1}. HR-MS (ESI) m/z calcd for $C_{17}H_{19}F_2O_2$ [M+H$^+$] 293.1353, found 293.1358 [M+H$^+$].

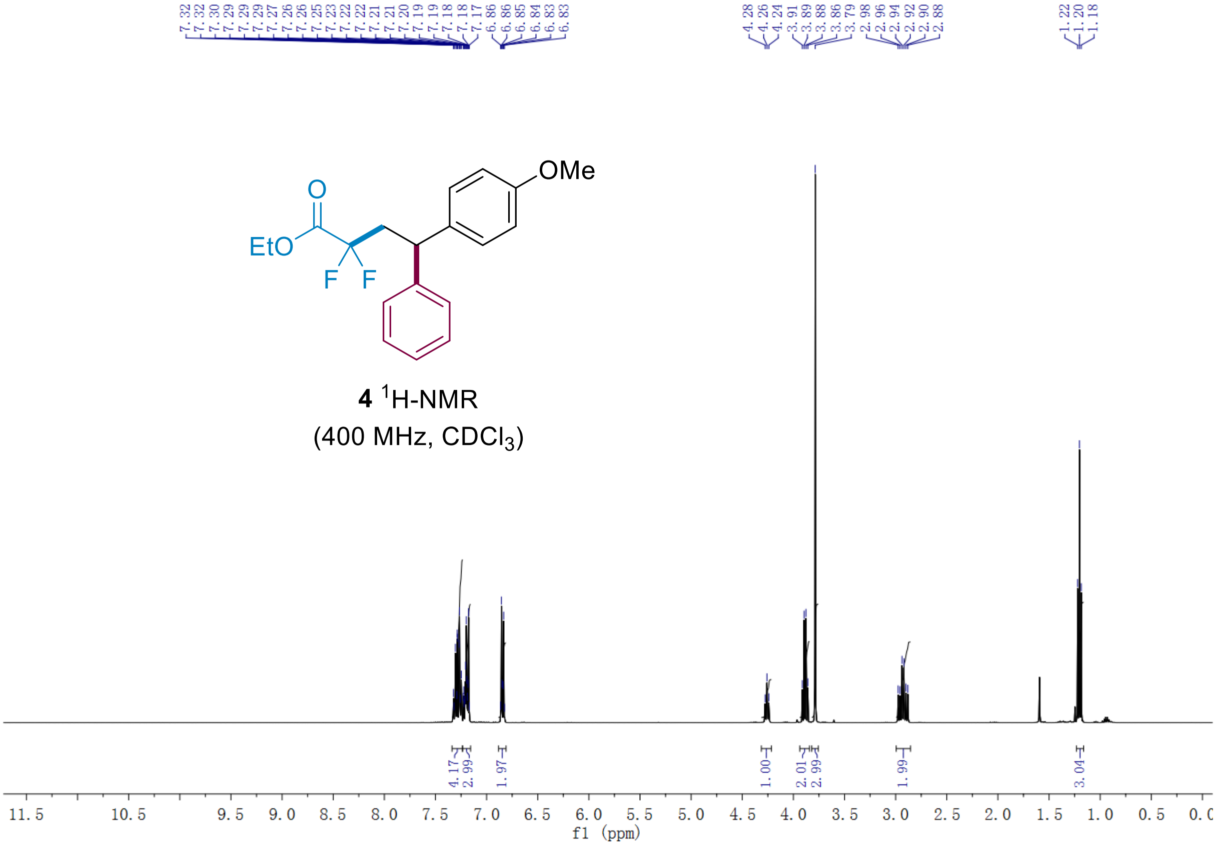

4 ^{1}H-NMR
(400 MHz, $CDCl_3$)

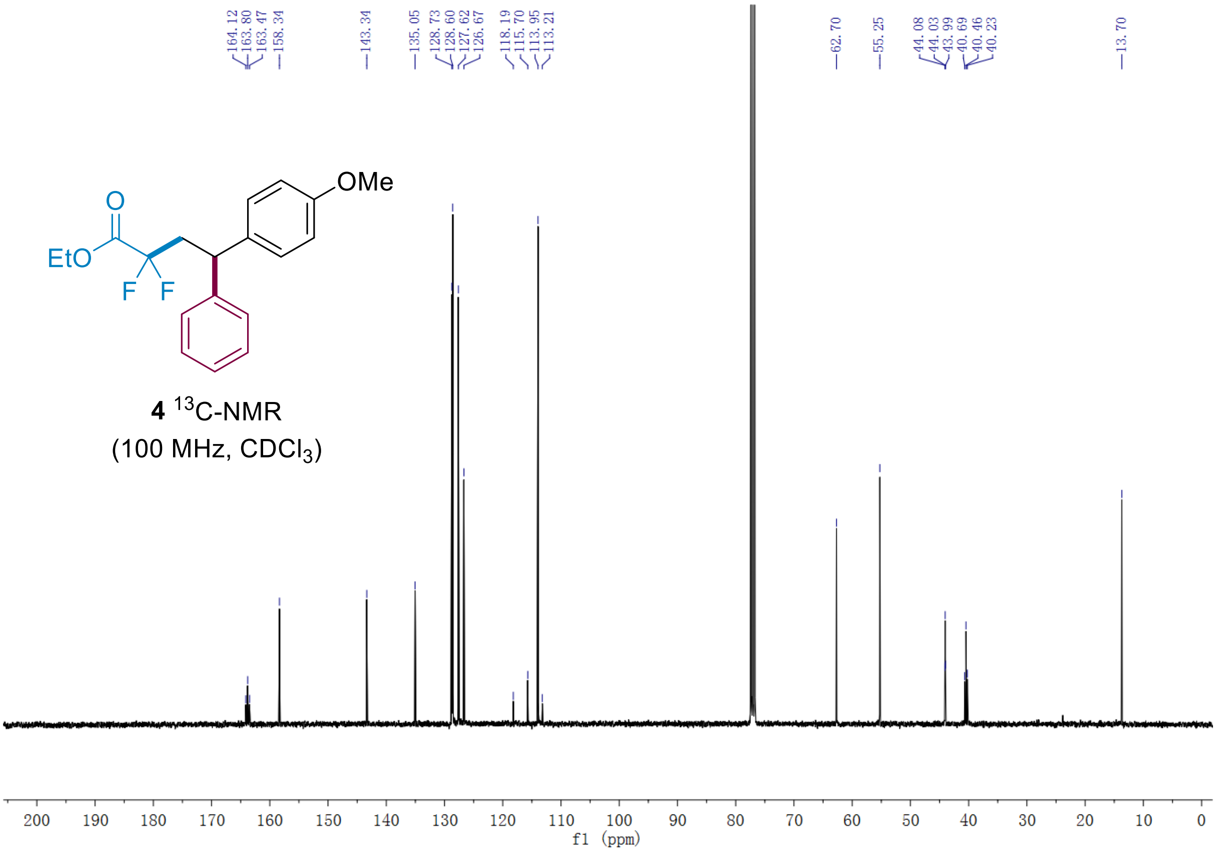

4 ^{13}C-NMR
(100 MHz, $CDCl_3$)

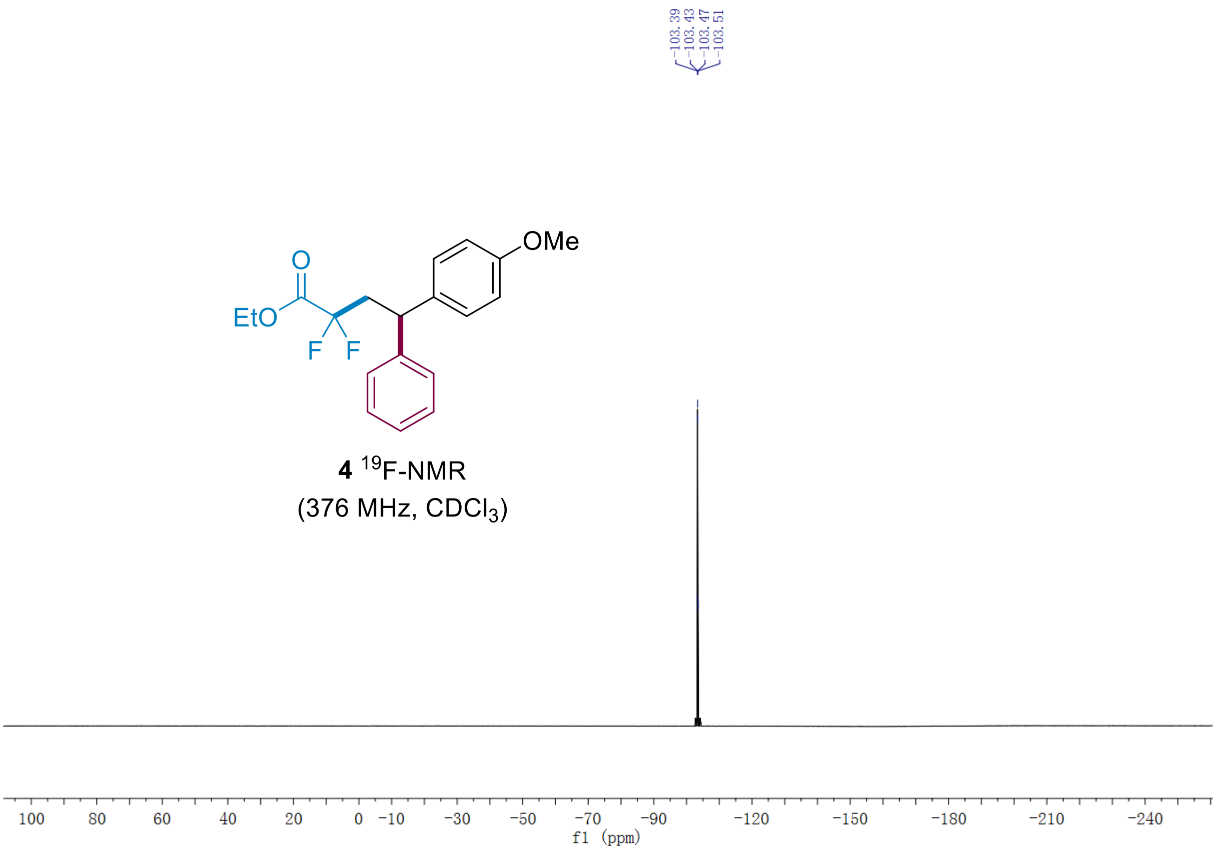
-103.39
-103.43
-103.47
-103.51
OMe
O
EtO
F F
4 19F-NMR
(376 MHz, CDCl3)
f1 (ppm)

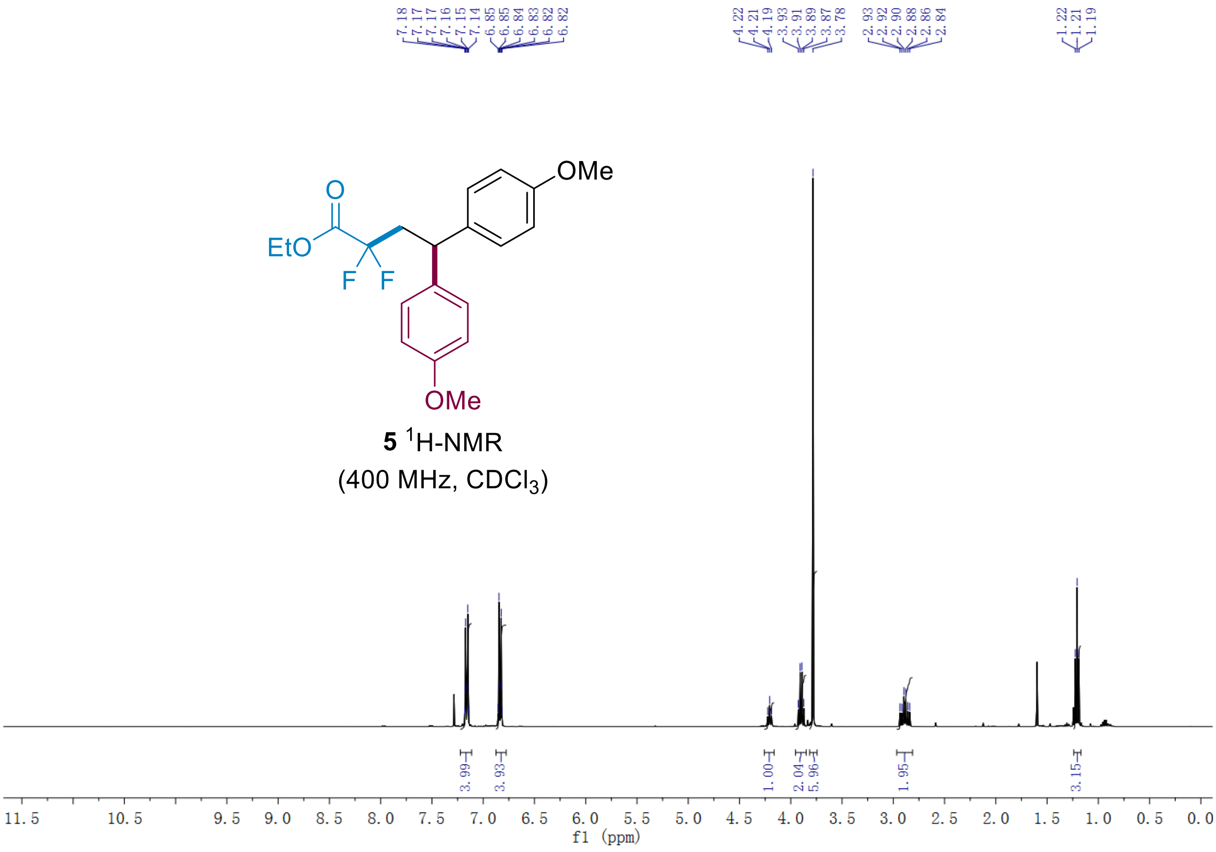
7.18
7.17
7.17
7.16
7.15
7.14
6.85
6.85
6.84
6.83
6.82
6.82
4.22
4.21
4.19
3.93
3.91
3.89
3.87
3.78
2.93
2.92
2.90
2.88
2.86
2.84
1.22
1.21
1.19
OMe
O
EtO
F F
OMe
5 1H-NMR
(400 MHz, CDCl3)
3.99
3.93
1.00
2.04
5.96
1.95
3.15
f1 (ppm)

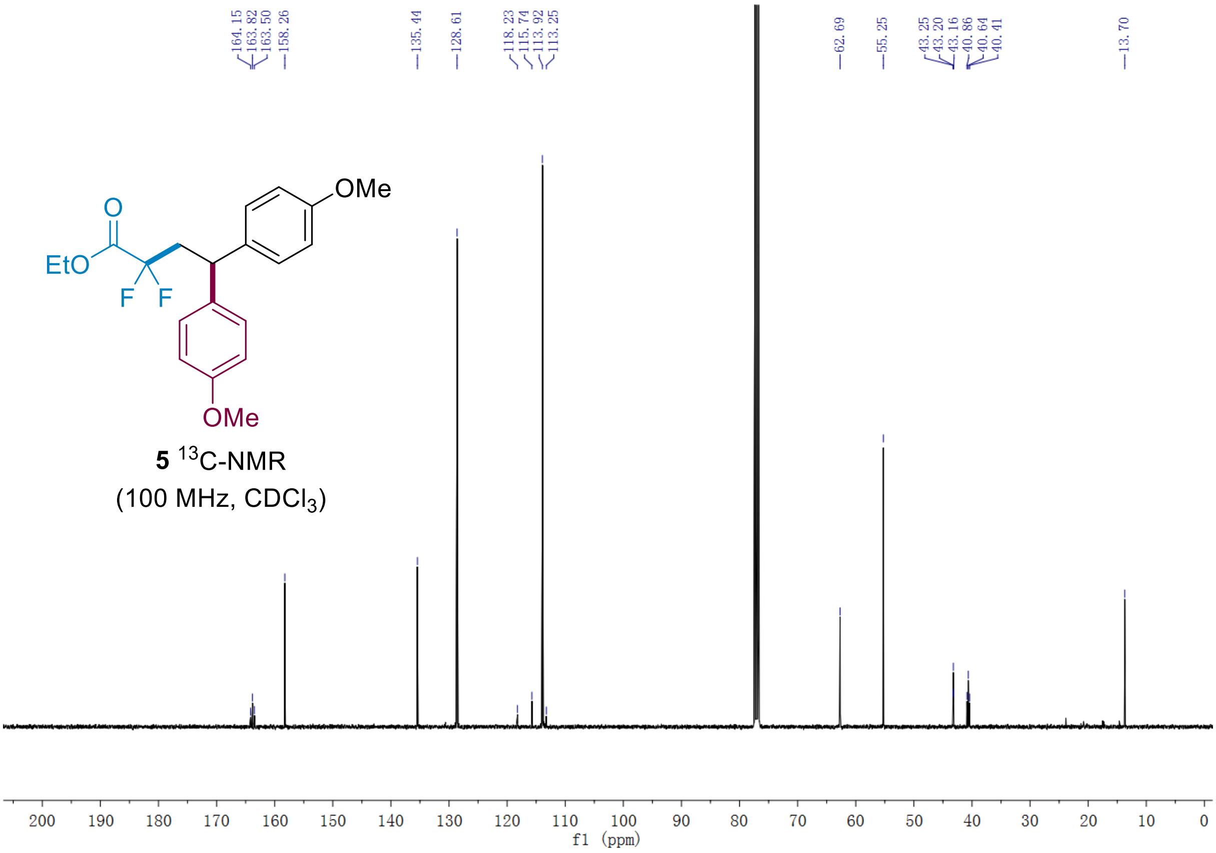
164.15
163.82
163.50
158.26
135.44
128.61
118.23
115.74
113.92
113.25
62.69
55.25
43.25
43.20
43.16
40.86
40.64
40.41
13.70
OMe
O
EtO
F F
OMe
5 13C-NMR
(100 MHz, CDCl3)
f1 (ppm)

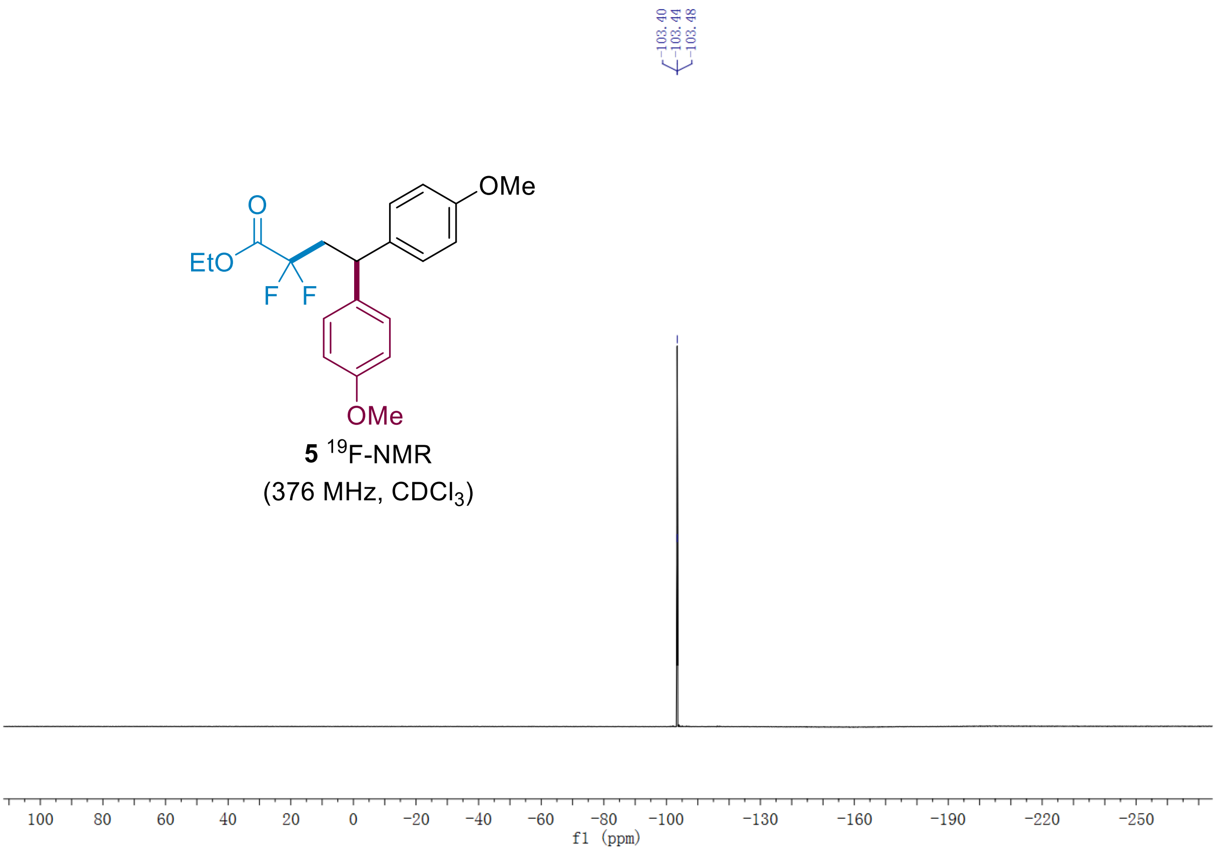
-103.40
-103.44
-103.48
OMe
O
EtO
F F
OMe
5 19F-NMR
(376 MHz, CDCl3)
f1 (ppm)

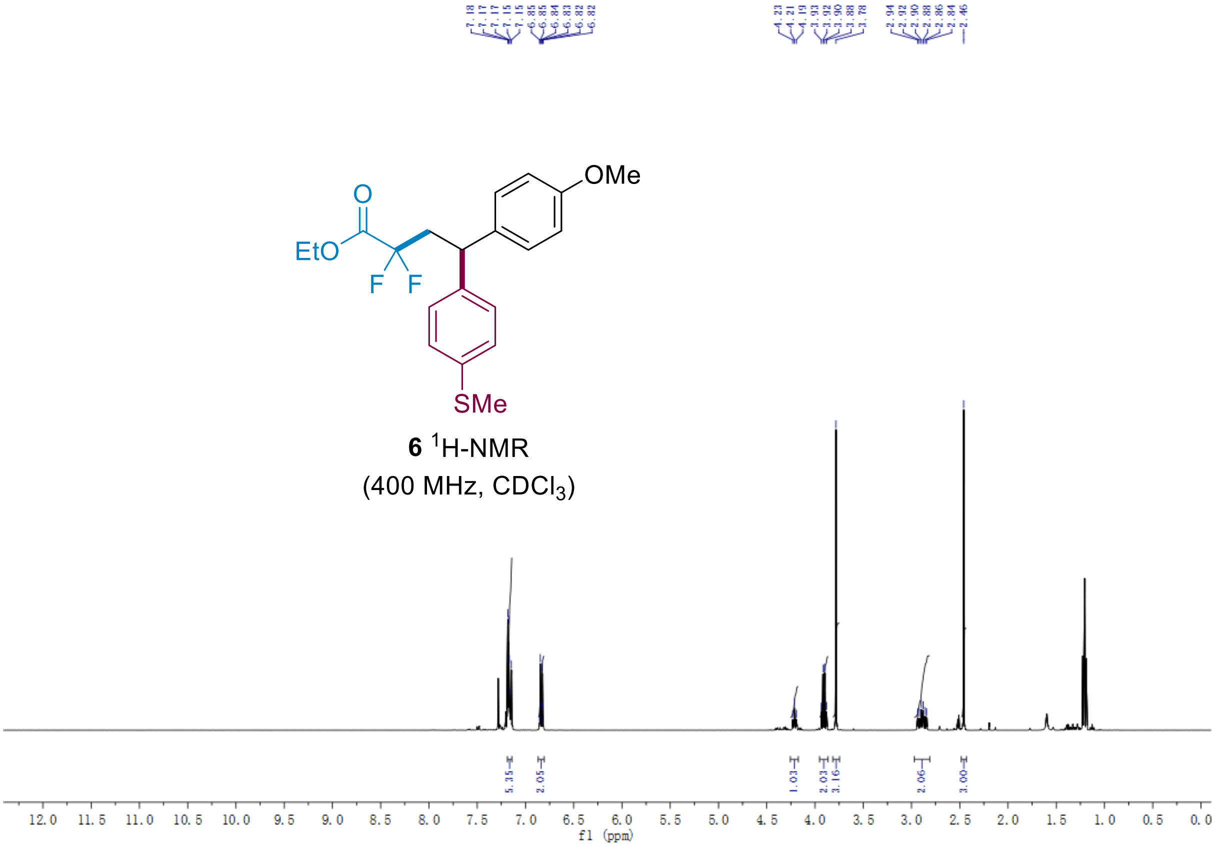
OMe
O
EtO
F F
SMe
6 1H-NMR
(400 MHz, CDCl3)
f1 (ppm)

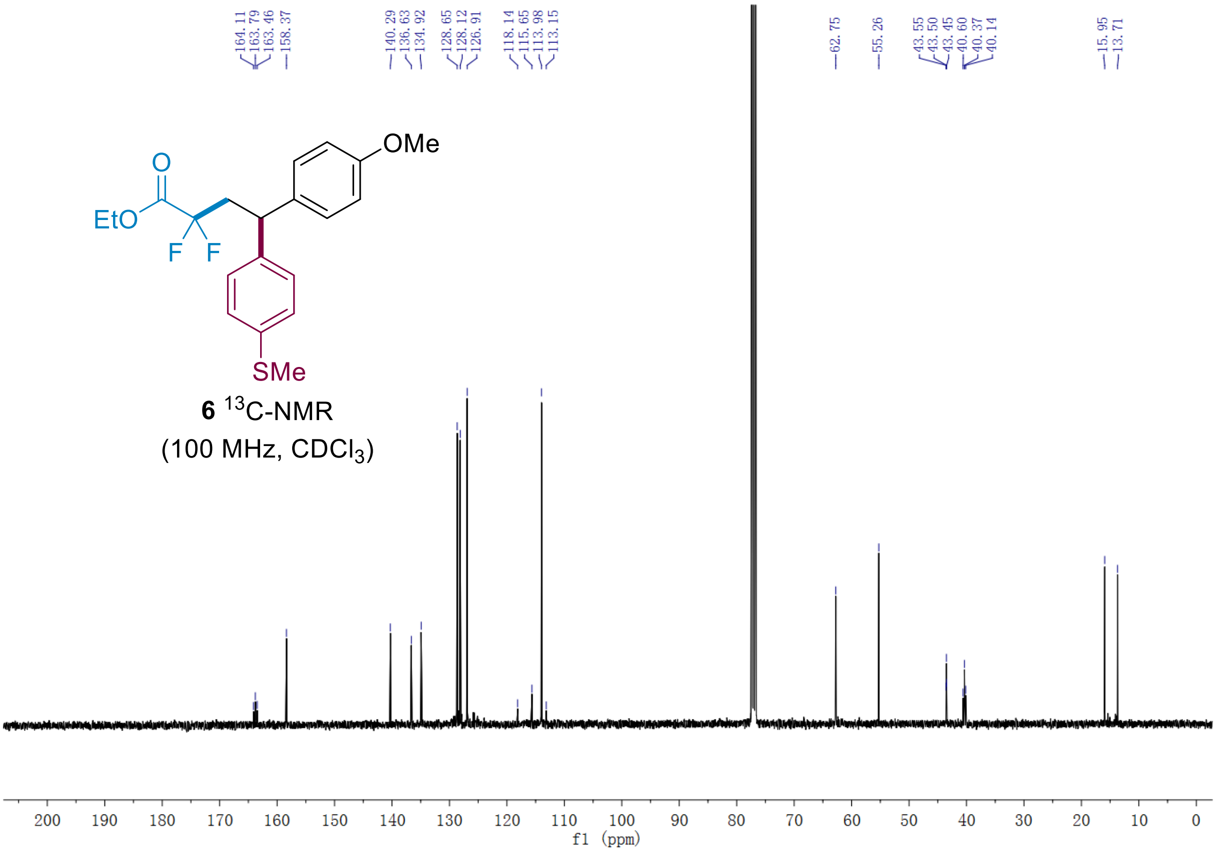
OMe
O
EtO
F F
SMe
6 13C-NMR
(100 MHz, CDCl3)
f1 (ppm)

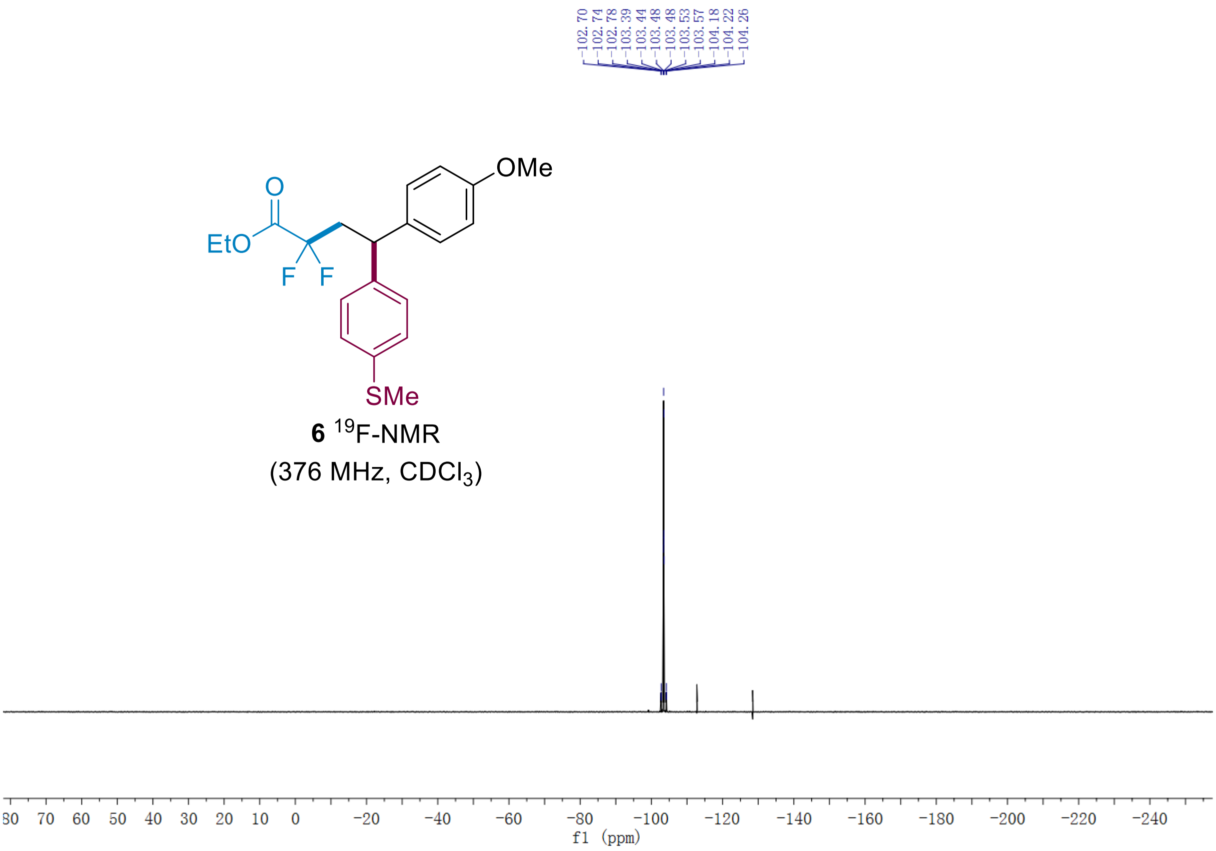

6 ^{19}F-NMR
(376 MHz, $CDCl_3$)

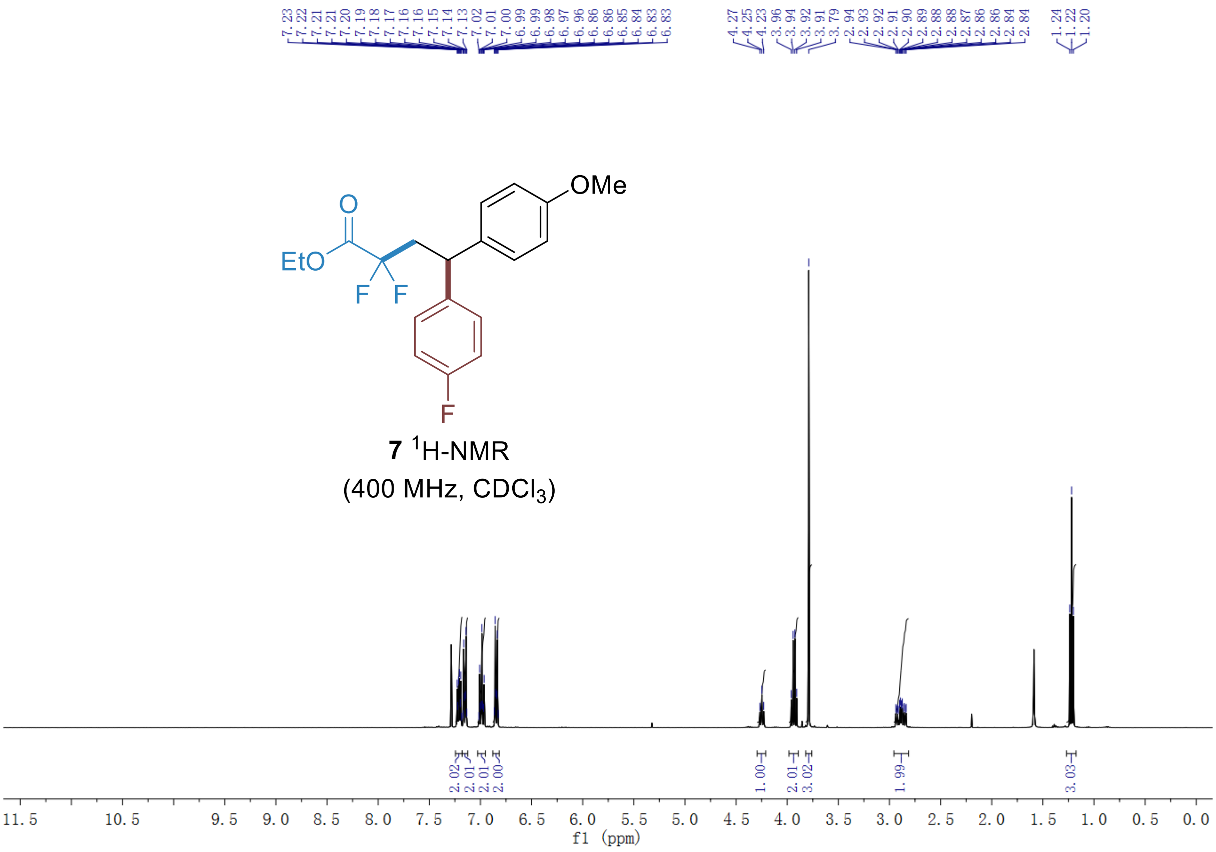

7 ^{1}H-NMR
(400 MHz, $CDCl_3$)

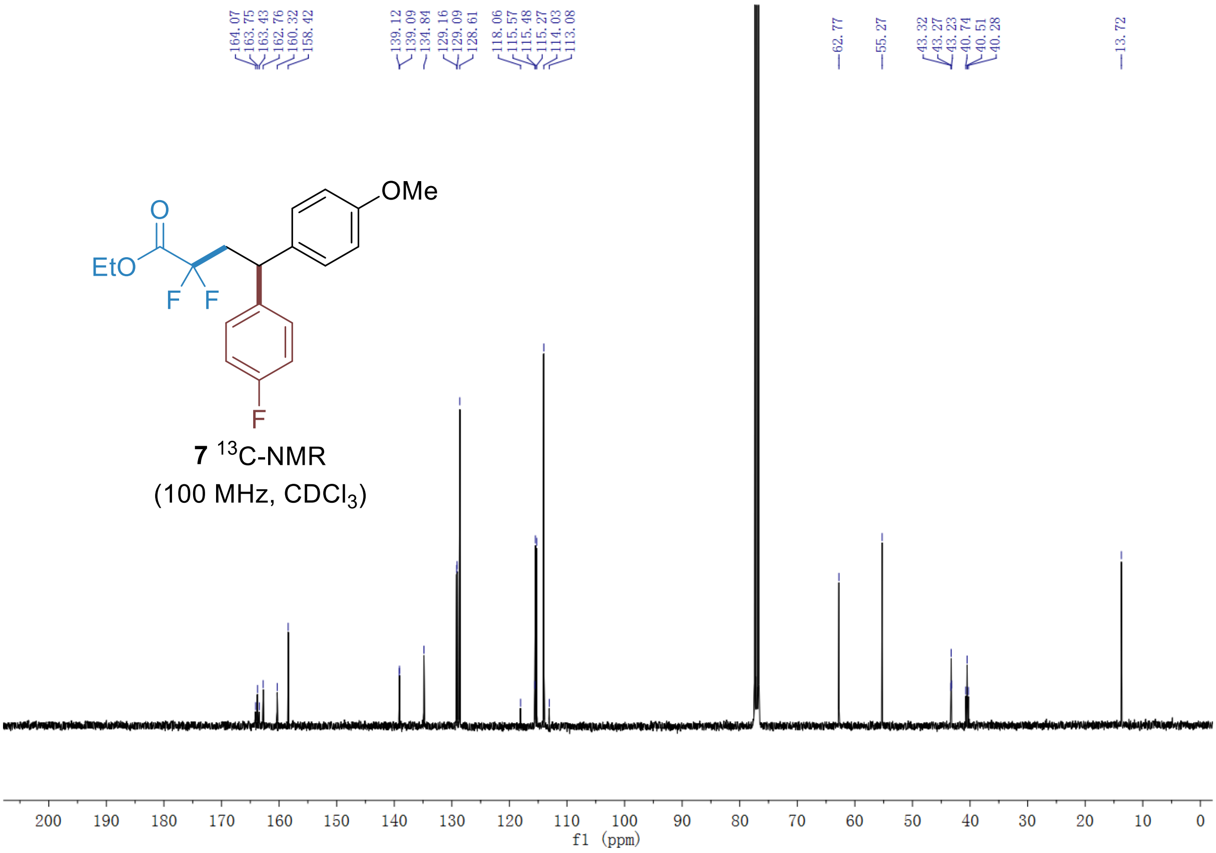
164.07
163.75
163.43
162.76
160.32
158.42
139.12
139.09
134.84
129.16
129.09
128.61
118.06
115.57
115.48
115.27
114.03
113.08
62.77
55.27
43.32
43.27
43.23
40.74
40.51
40.28
13.72
OMe
O
EtO
F F
F
7 13C-NMR
(100 MHz, CDCl3)
200 190 180 170 160 150 140 130 120 110 100 90 80 70 60 50 40 30 20 10 0
f1 (ppm)

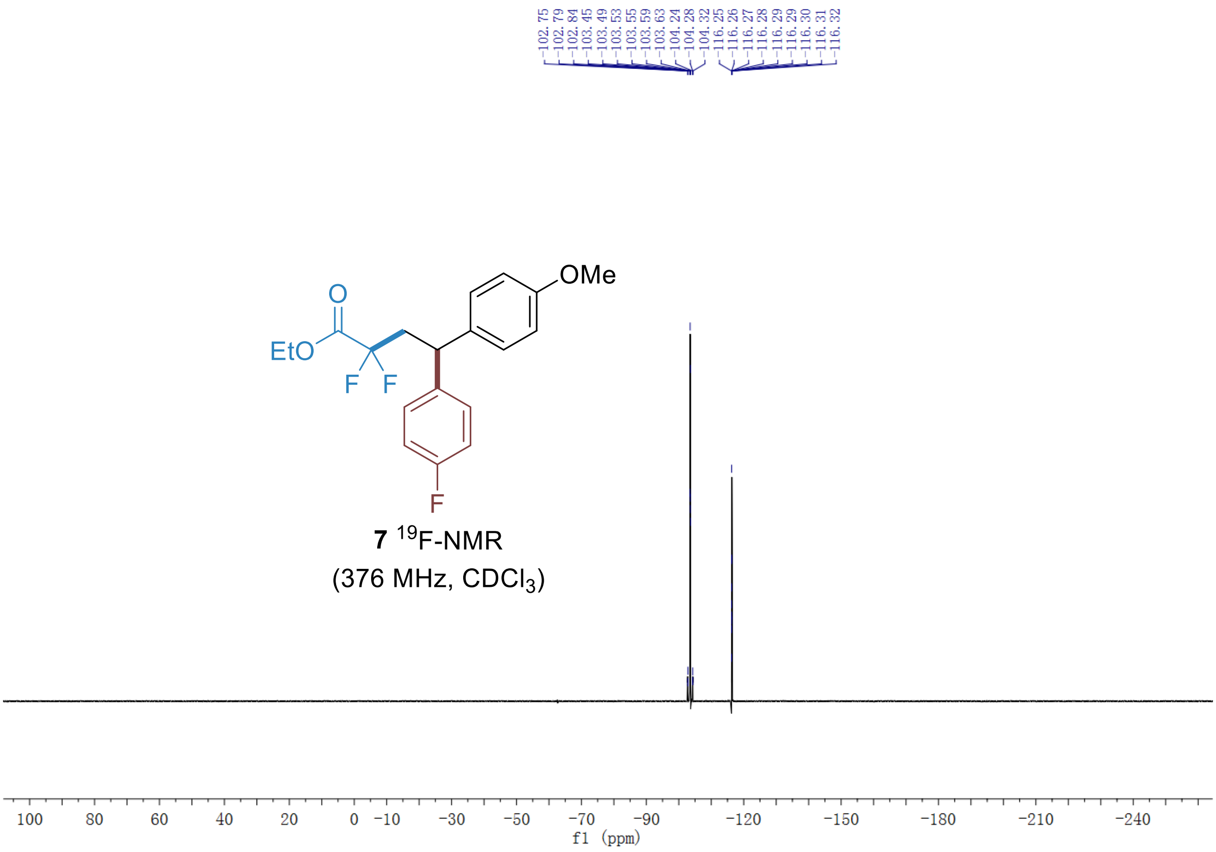
-102.75
-102.79
-102.84
-103.45
-103.49
-103.53
-103.55
-103.59
-103.63
-104.24
-104.28
-104.32
-116.25
-116.26
-116.27
-116.28
-116.29
-116.30
-116.31
-116.32
OMe
O
EtO
F F
F
7 19F-NMR
(376 MHz, CDCl3)
100 80 60 40 20 0 -10 -30 -50 -70 -90 -120 -150 -180 -210 -240
f1 (ppm)

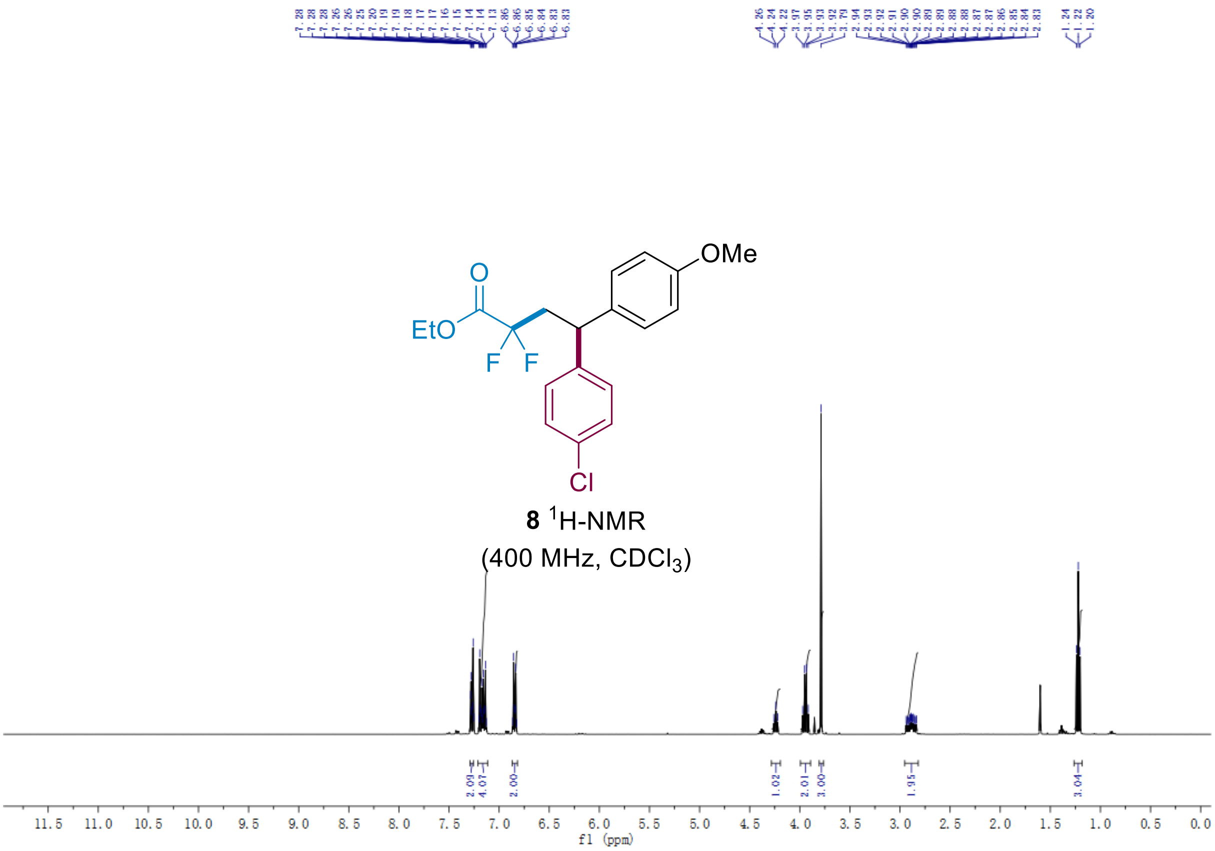
OMe
EtO
O
F F
Cl
8 1H-NMR
(400 MHz, CDCl3)
f1 (ppm)

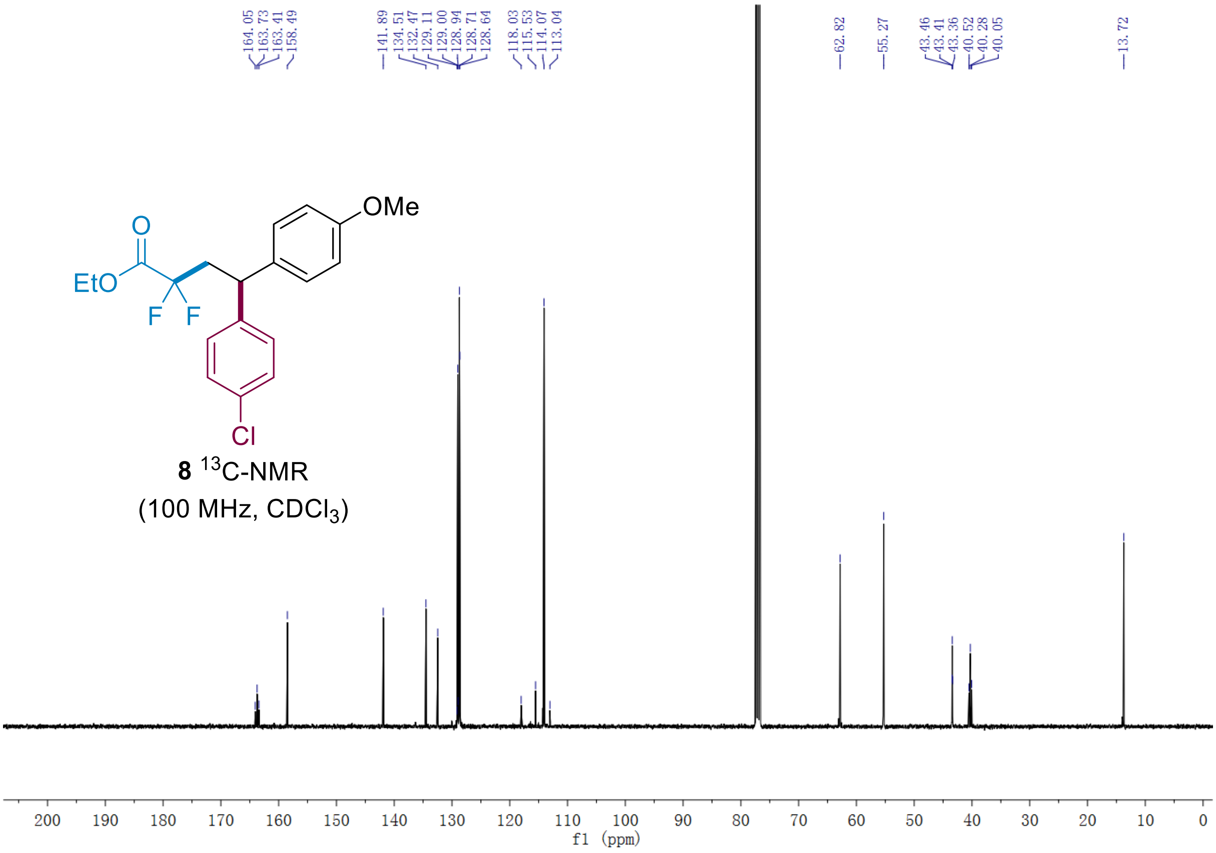
164.05
163.73
163.41
158.49
141.89
134.51
132.47
129.11
129.00
128.94
128.71
128.64
118.03
115.53
114.07
113.04
62.82
55.27
43.46
43.41
43.36
40.52
40.28
40.05
13.72
OMe
EtO
O
F F
Cl
8 13C-NMR
(100 MHz, CDCl3)
f1 (ppm)

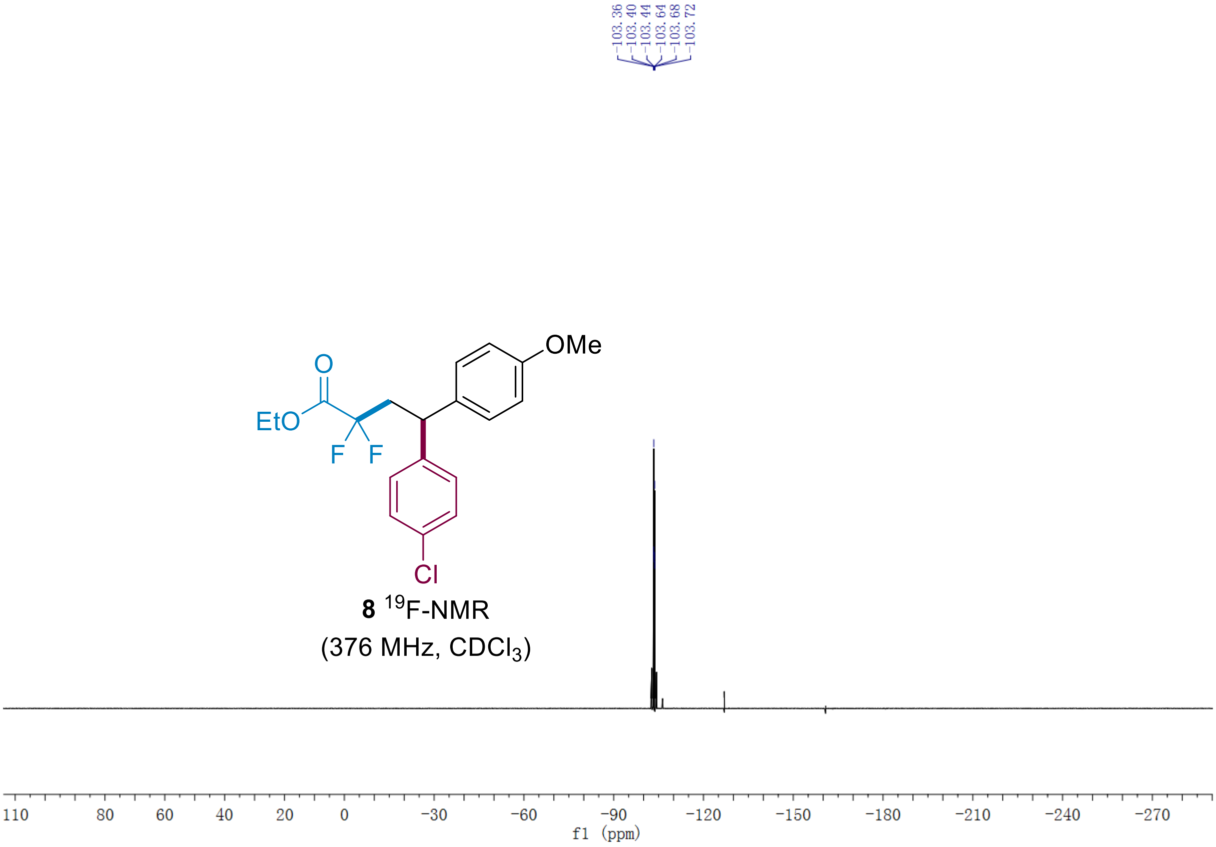
-103.36
-103.40
-103.44
-103.64
-103.68
-103.72
OMe
O
EtO
F F
Cl
8 19F-NMR
(376 MHz, CDCl3)
f1 (ppm)

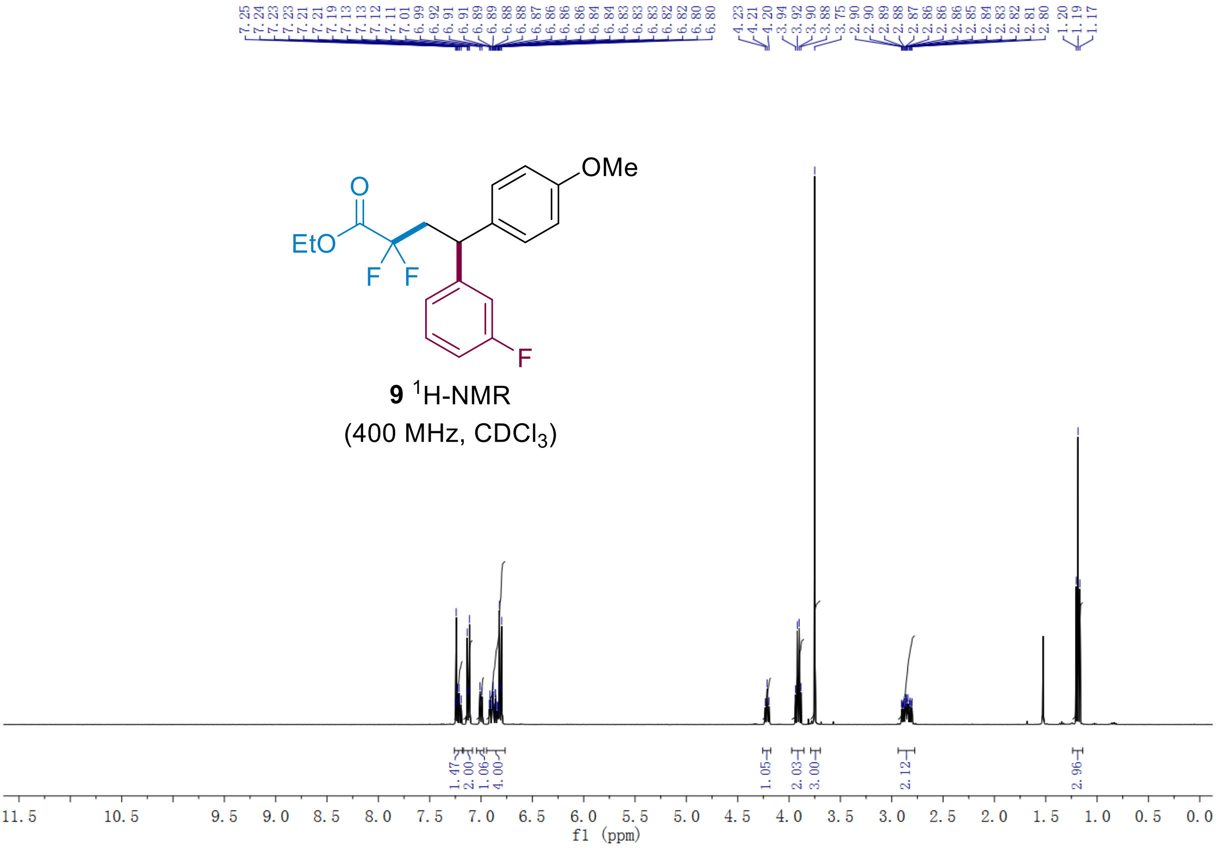
OMe
O
EtO
F F
F
9 1H-NMR
(400 MHz, CDCl3)
f1 (ppm)

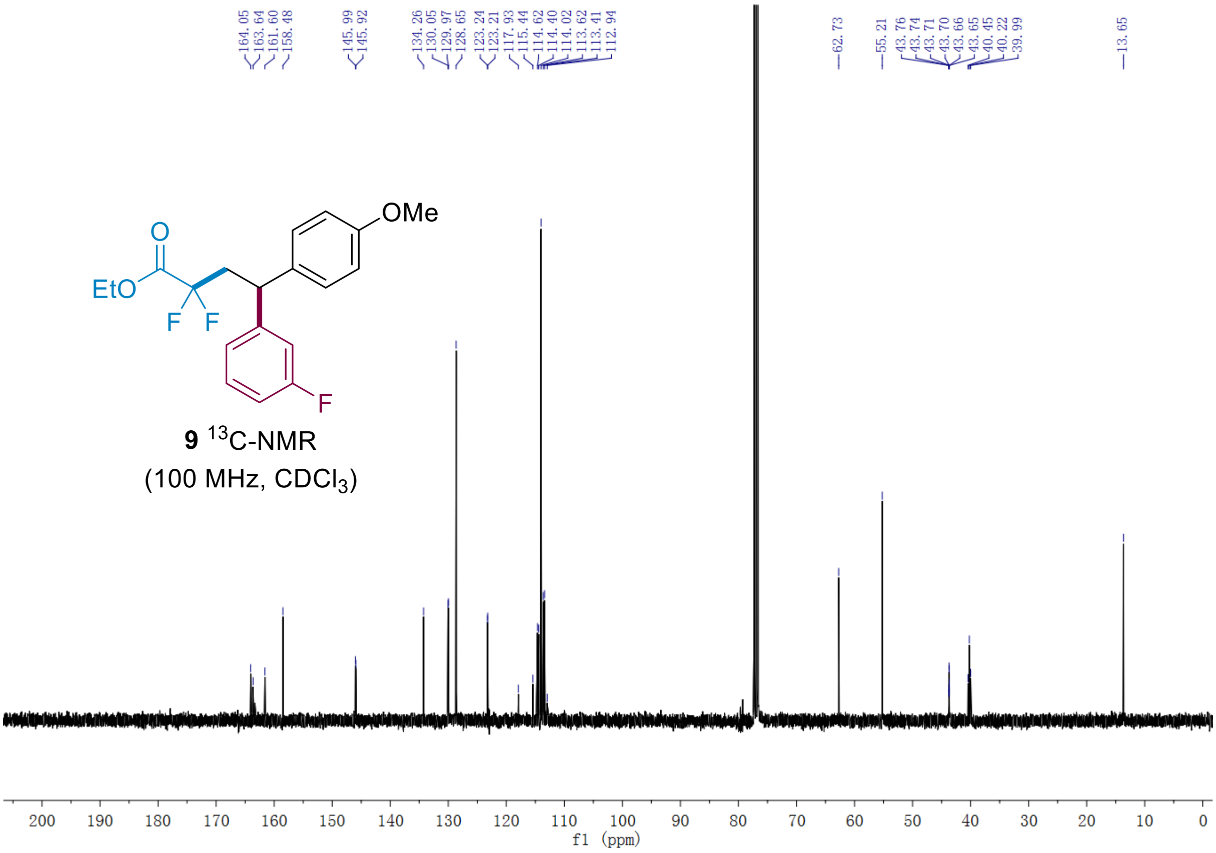
164.05
163.64
161.60
158.48
145.99
145.92
134.26
130.05
129.97
128.65
123.24
123.21
117.93
115.44
114.62
114.40
114.02
113.62
113.41
112.94
62.73
55.21
43.76
43.74
43.71
43.70
43.66
43.65
40.45
40.22
39.99
13.65
OMe
O
EtO
F F
F
9 ^{13}C-NMR
(100 MHz, $CDCl_3$)
f1 (ppm)

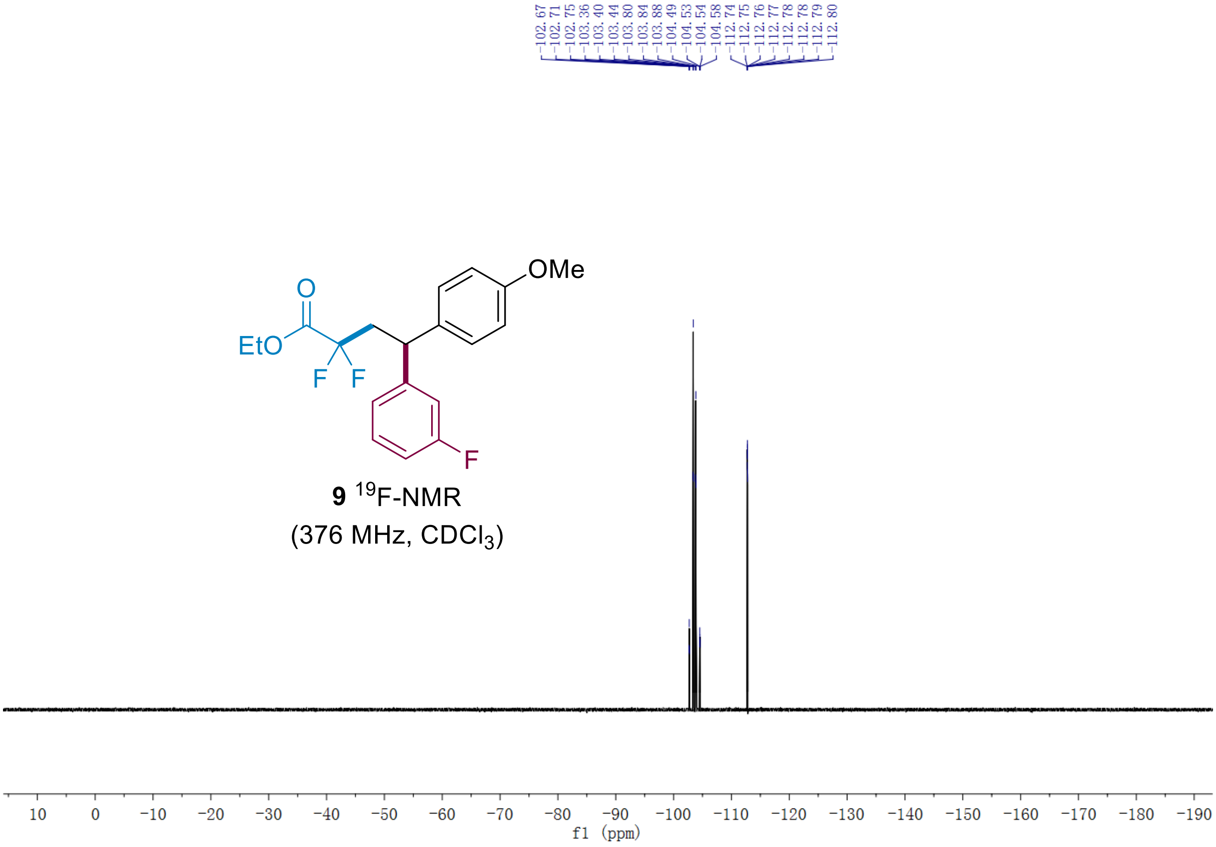
-102.67
-102.71
-102.75
-103.36
-103.40
-103.44
-103.80
-103.84
-103.88
-104.49
-104.53
-104.54
-104.58
-112.74
-112.75
-112.76
-112.77
-112.78
-112.78
-112.79
-112.80
OMe
O
EtO
F F
F
9 ^{19}F-NMR
(376 MHz, $CDCl_3$)
f1 (ppm)

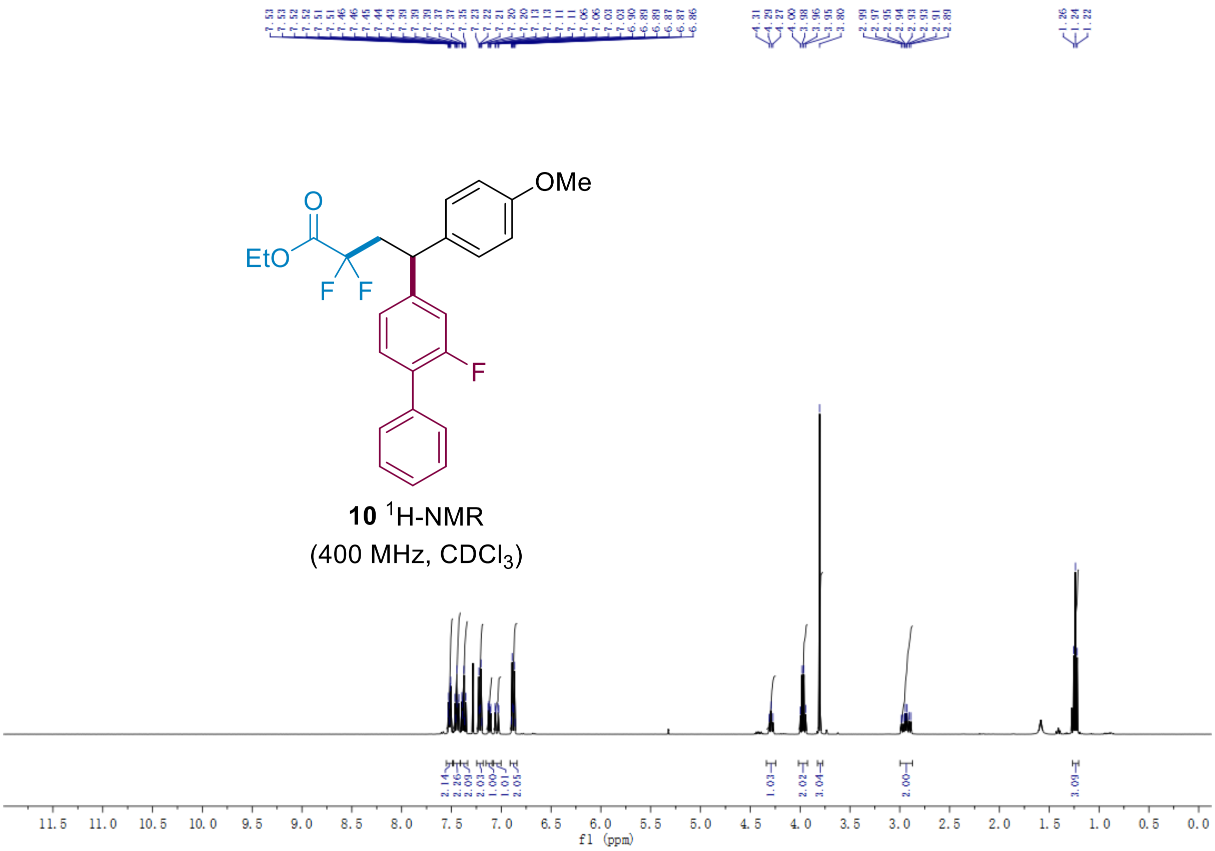

10 ^{1}H-NMR
(400 MHz, $CDCl_3$)

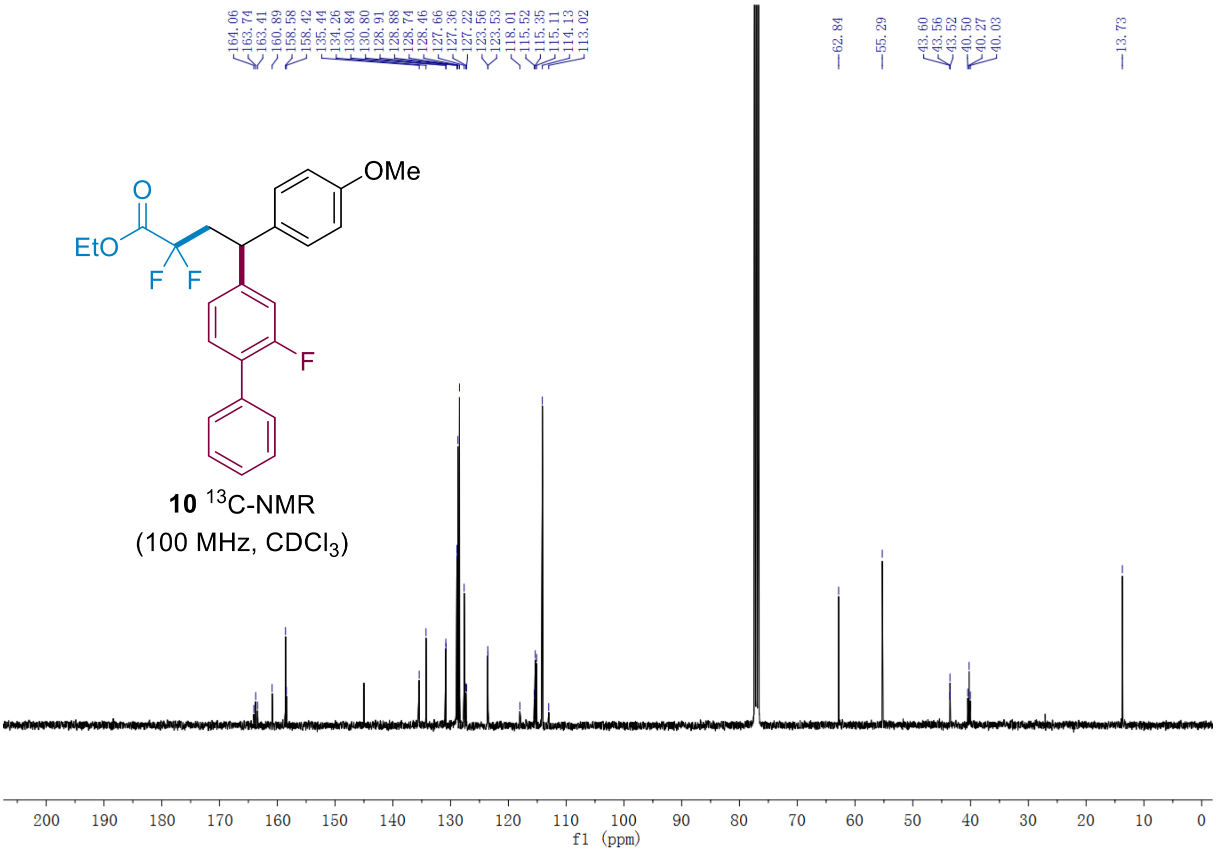

10 ^{13}C-NMR
(100 MHz, $CDCl_3$)

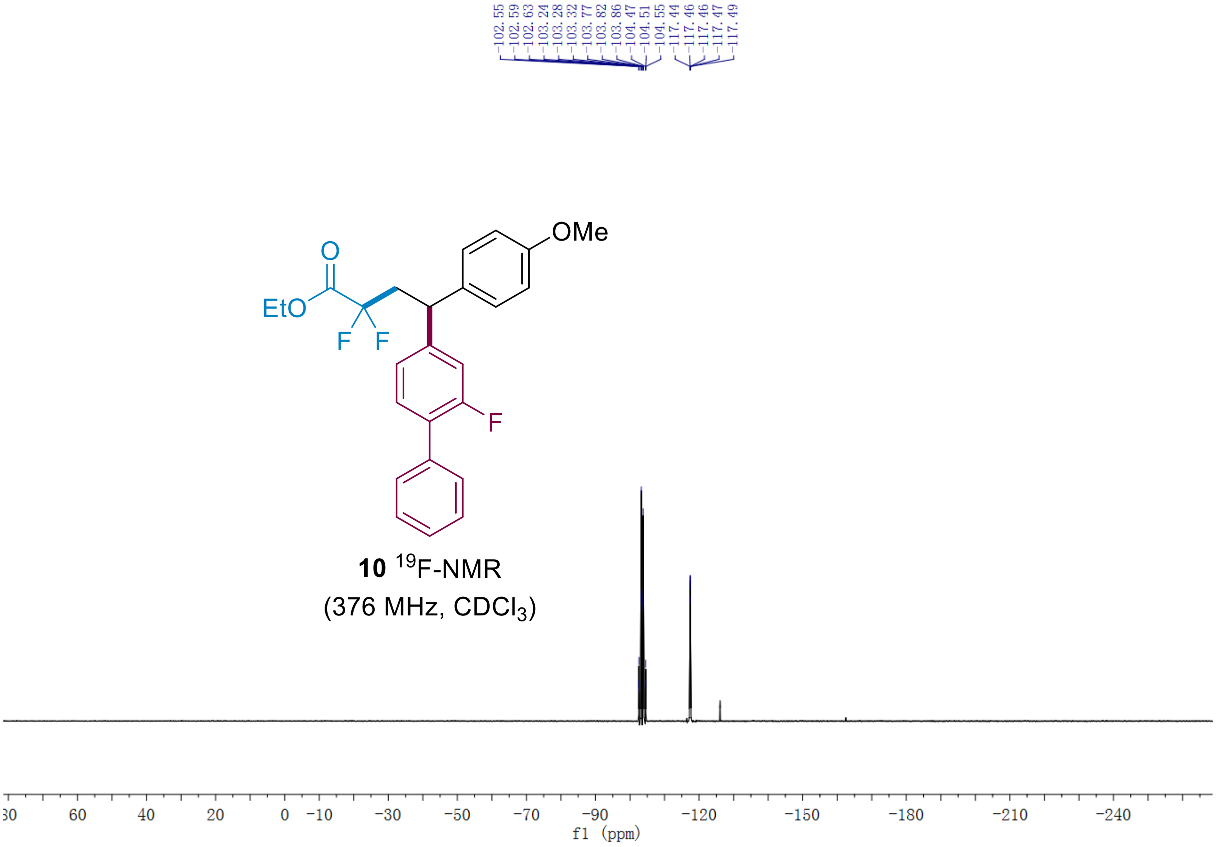

10 ^{19}F-NMR (376 MHz, $CDCl_3$)

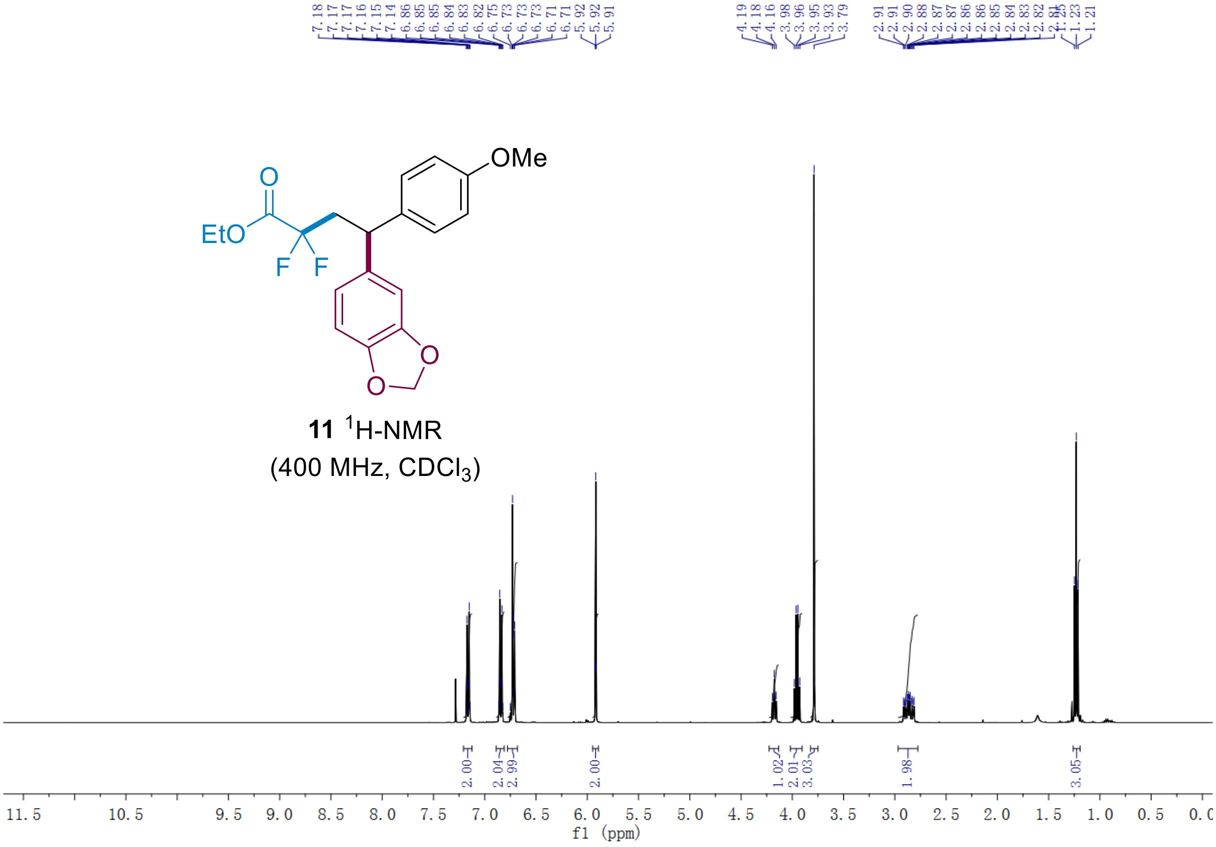

11 ^{1}H-NMR (400 MHz, $CDCl_3$)

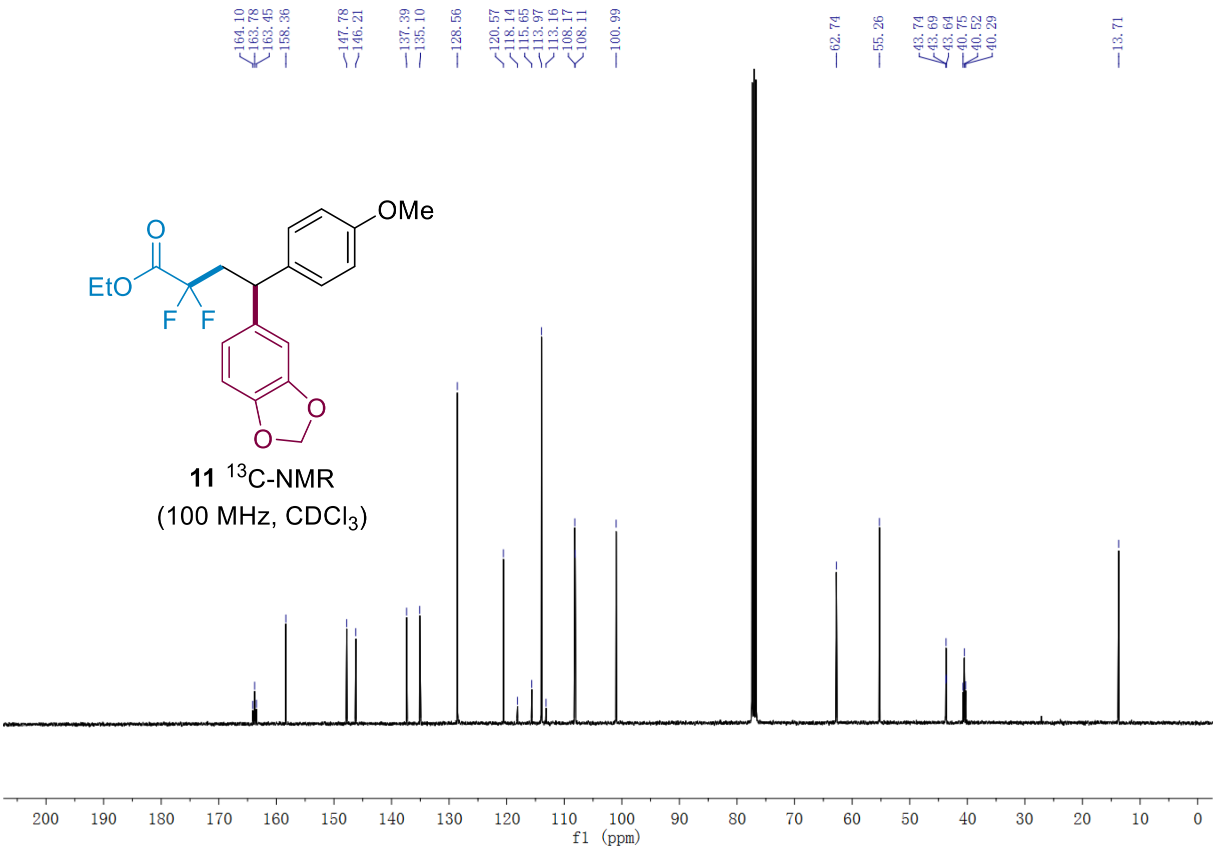
164.10
163.78
163.45
158.36
147.78
146.21
137.39
135.10
128.56
120.57
118.14
115.65
113.97
113.16
108.17
108.11
100.99
62.74
55.26
43.74
43.69
43.64
40.75
40.52
40.29
13.71
OMe
O
EtO
F F
O
O
11 ^{13}C-NMR
(100 MHz, $CDCl_3$)
200 190 180 170 160 150 140 130 120 110 100 90 80 70 60 50 40 30 20 10 0
f1 (ppm)

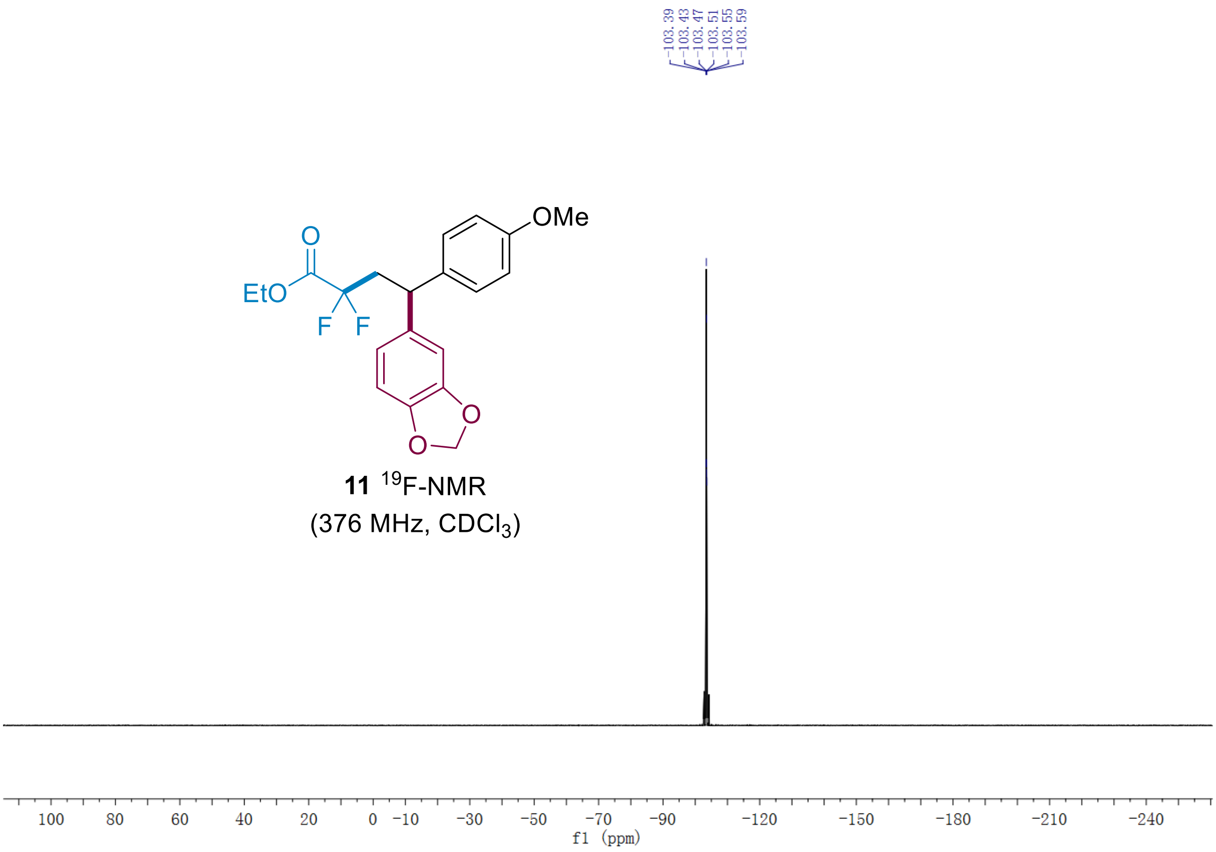
-103.39
-103.43
-103.47
-103.51
-103.55
-103.59
OMe
O
EtO
F F
O
O
11 ^{19}F-NMR
(376 MHz, $CDCl_3$)
100 80 60 40 20 0 -10 -30 -50 -70 -90 -120 -150 -180 -210 -240
f1 (ppm)

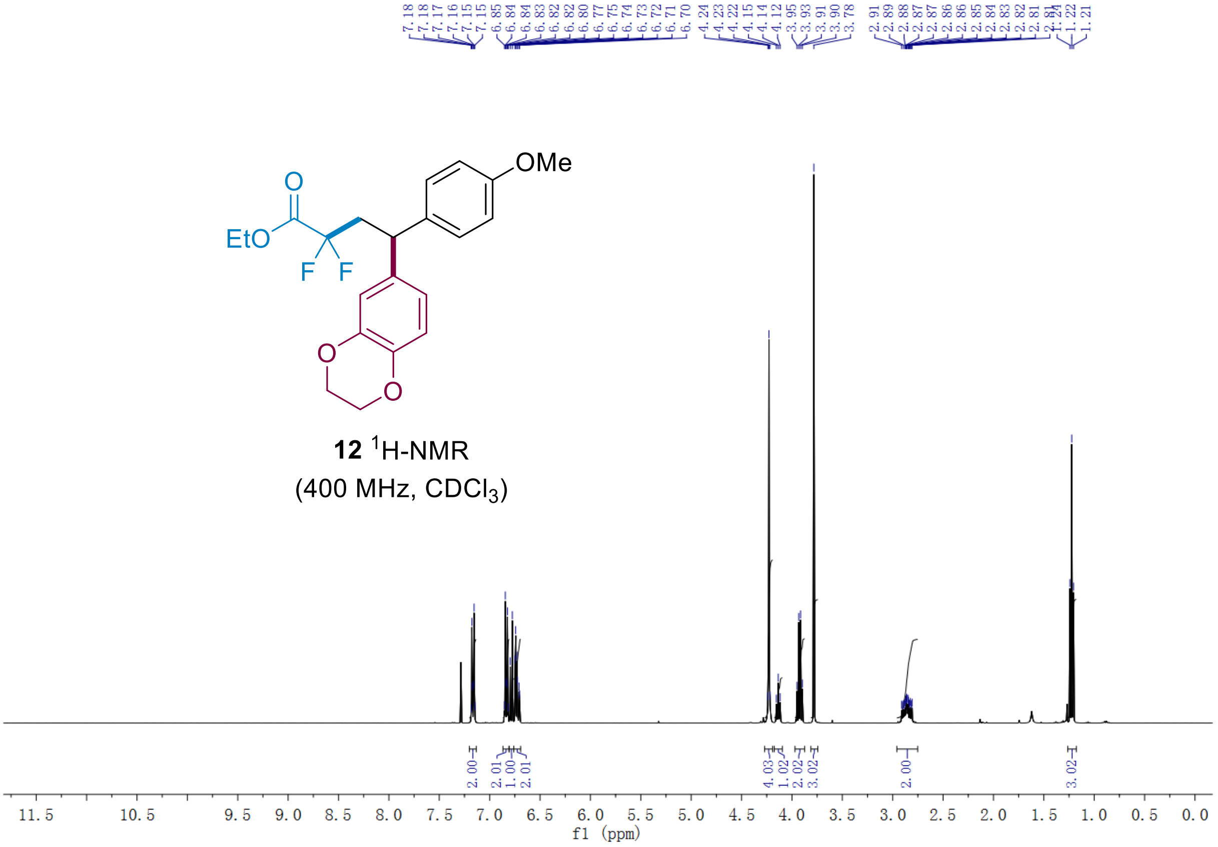

12 ^{1}H-NMR (400 MHz, $CDCl_3$)

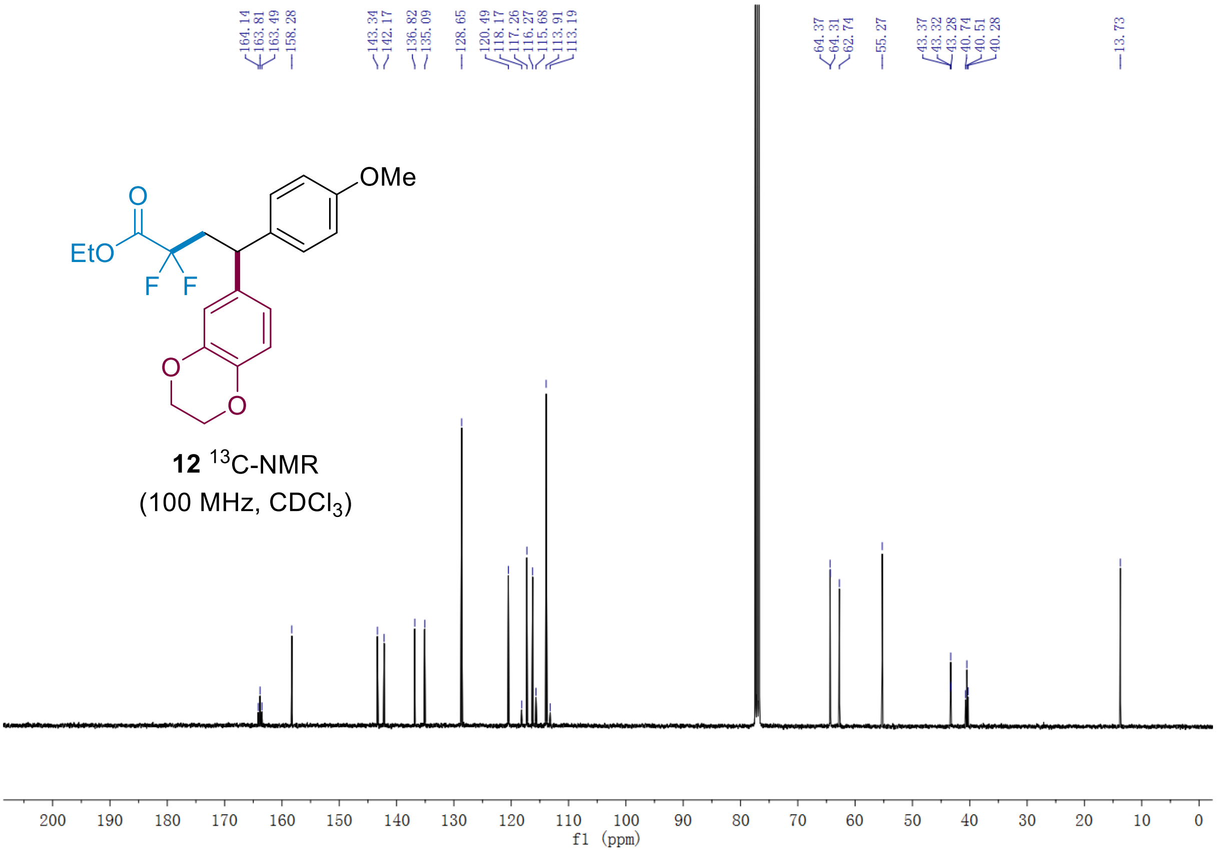

12 ^{13}C-NMR (100 MHz, $CDCl_3$)

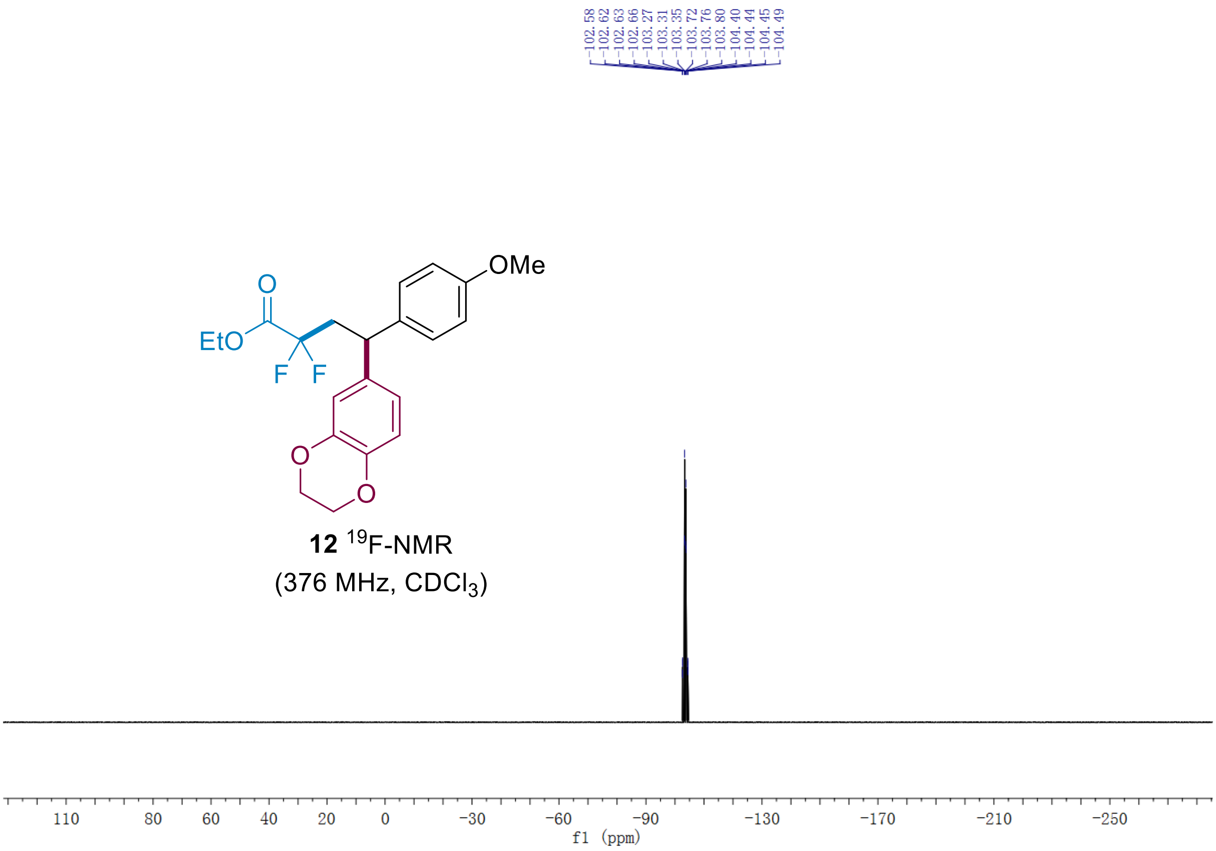

-102.58
-102.62
-102.63
-102.66
-103.27
-103.31
-103.35
-103.72
-103.76
-103.80
-104.40
-104.44
-104.45
-104.49
OMe
O
EtO
F F
O
O
12 19F-NMR
(376 MHz, CDCl3)
110 80 60 40 20 0 -30 -60 -90 -130 -170 -210 -250
f1 (ppm)

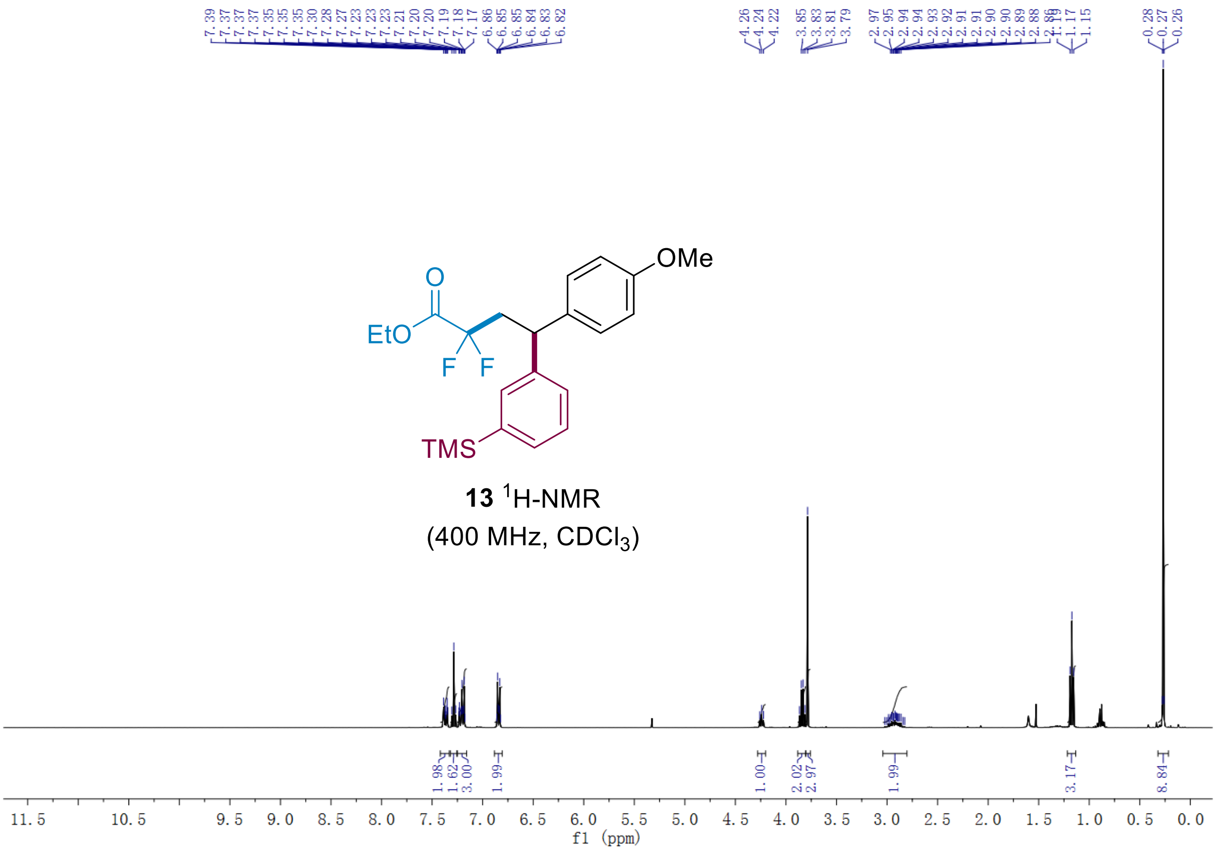

7.39
7.37
7.37
7.37
7.35
7.35
7.30
7.28
7.27
7.23
7.23
7.23
7.21
7.20
7.20
7.19
7.18
7.17
6.86
6.85
6.85
6.84
6.83
6.82
4.26
4.24
4.22
3.85
3.83
3.81
3.79
2.97
2.95
2.94
2.94
2.93
2.92
2.91
2.91
2.90
2.89
2.88
1.19
1.17
1.15
0.28
0.27
0.26
OMe
O
EtO
F F
TMS
13 1H-NMR
(400 MHz, CDCl3)
1.98
1.62
3.00
1.99
1.00
2.02
2.97
1.99
3.17
8.84
11.5 10.5 9.5 9.0 8.5 8.0 7.5 7.0 6.5 6.0 5.5 5.0 4.5 4.0 3.5 3.0 2.5 2.0 1.5 1.0 0.5 0.0
f1 (ppm)

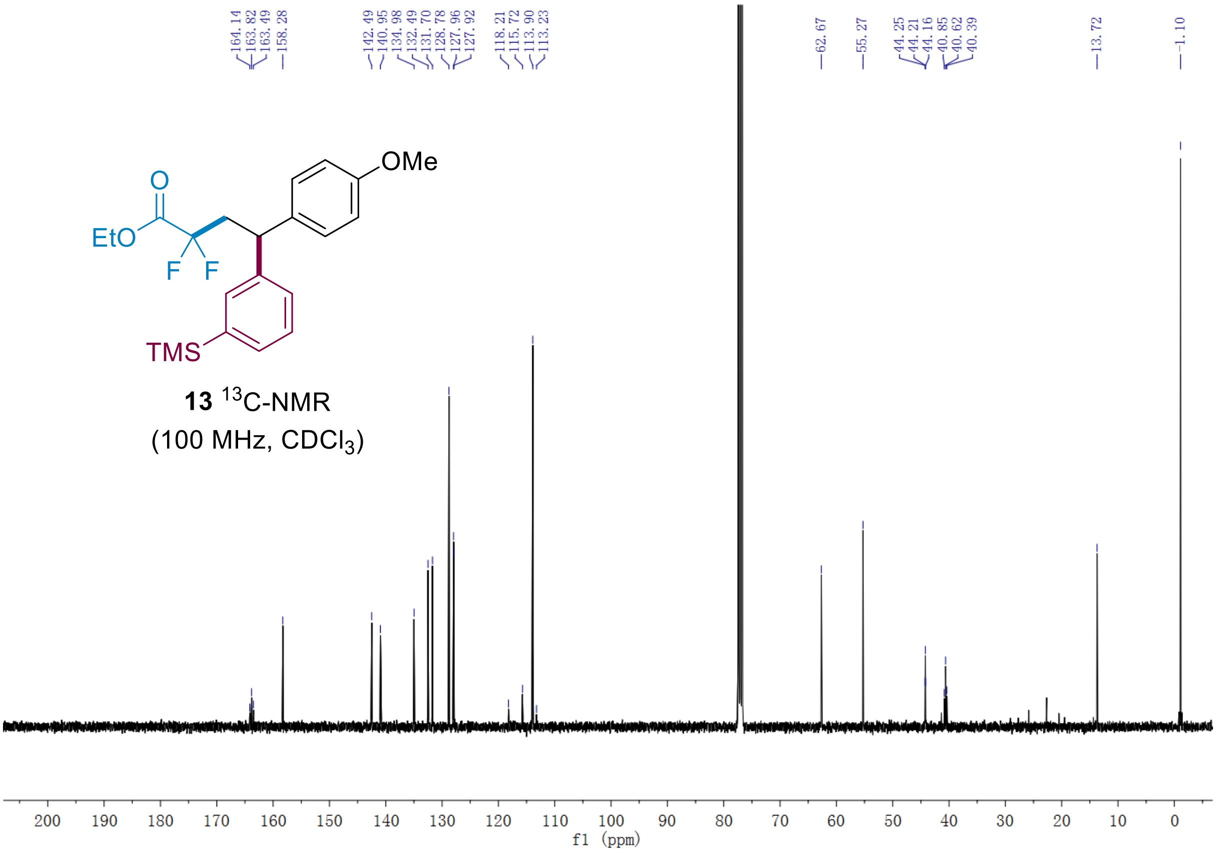

13 ^{13}C-NMR (100 MHz, $CDCl_3$)

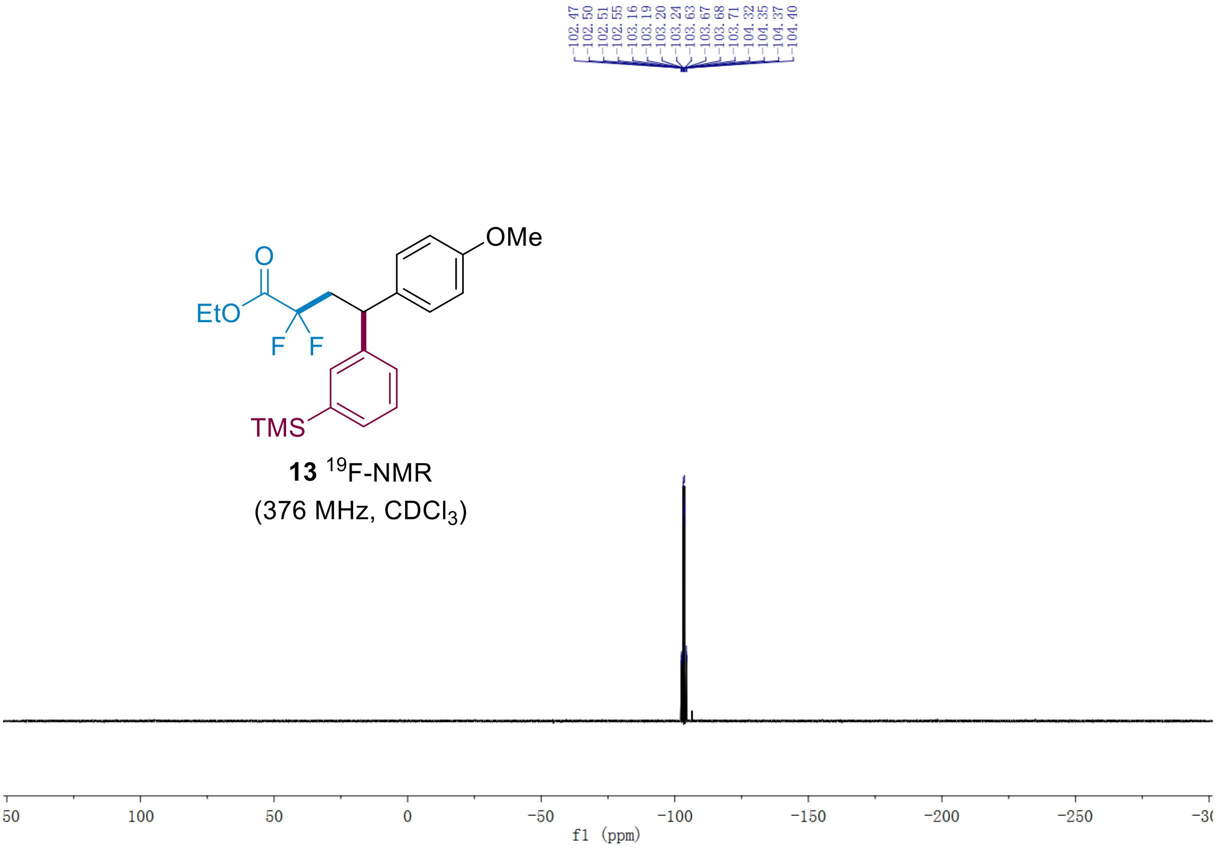

13 ^{19}F-NMR (376 MHz, $CDCl_3$)

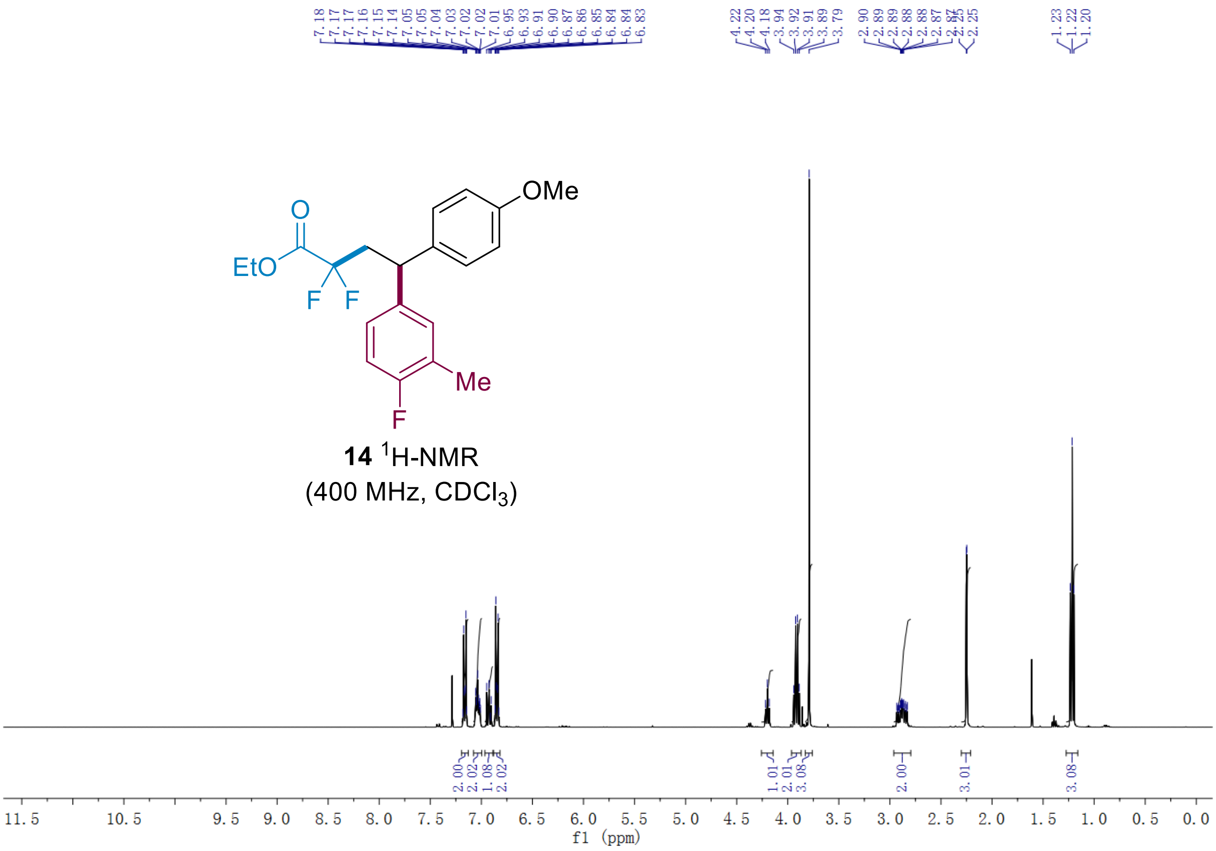
OMe
EtO
O
F F
Me
F
14 ¹H-NMR
(400 MHz, CDCl3)
f1 (ppm)

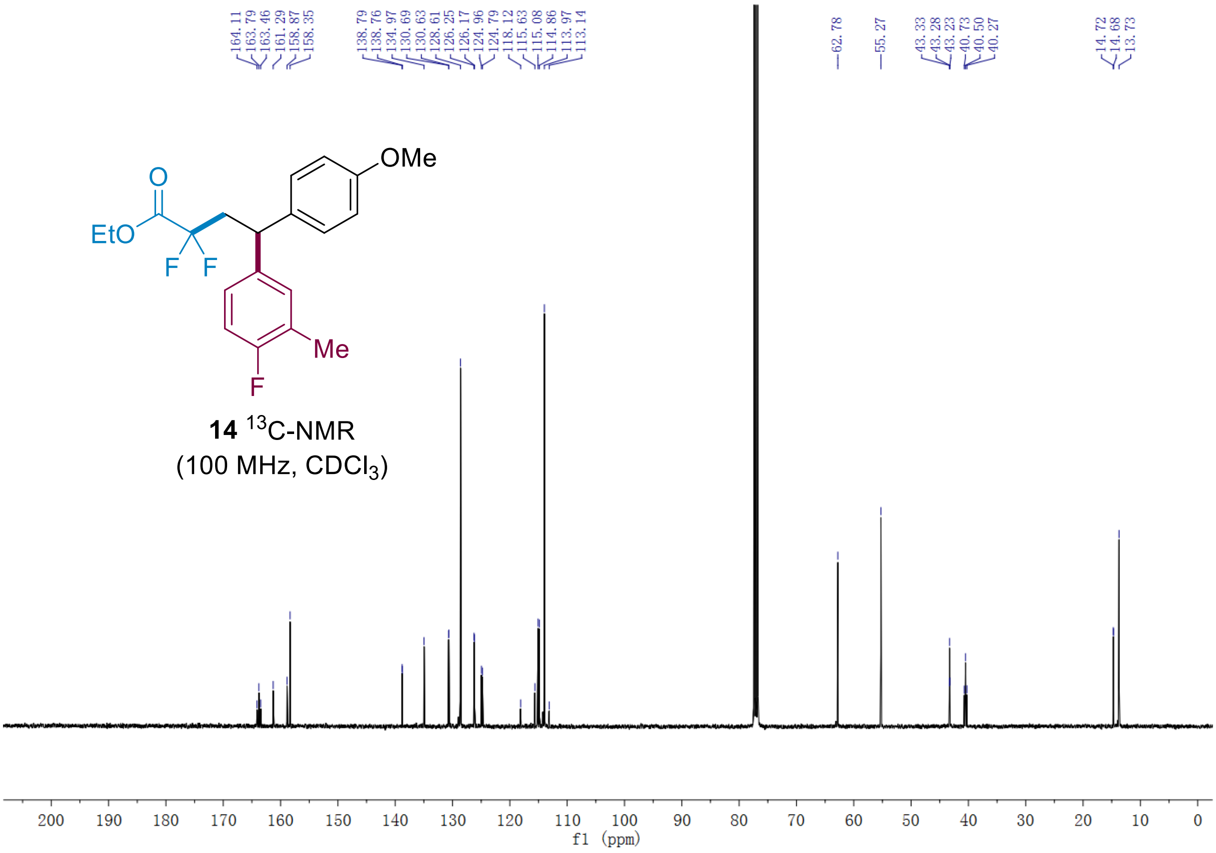
OMe
EtO
O
F F
Me
F
14 ¹³C-NMR
(100 MHz, CDCl3)
f1 (ppm)

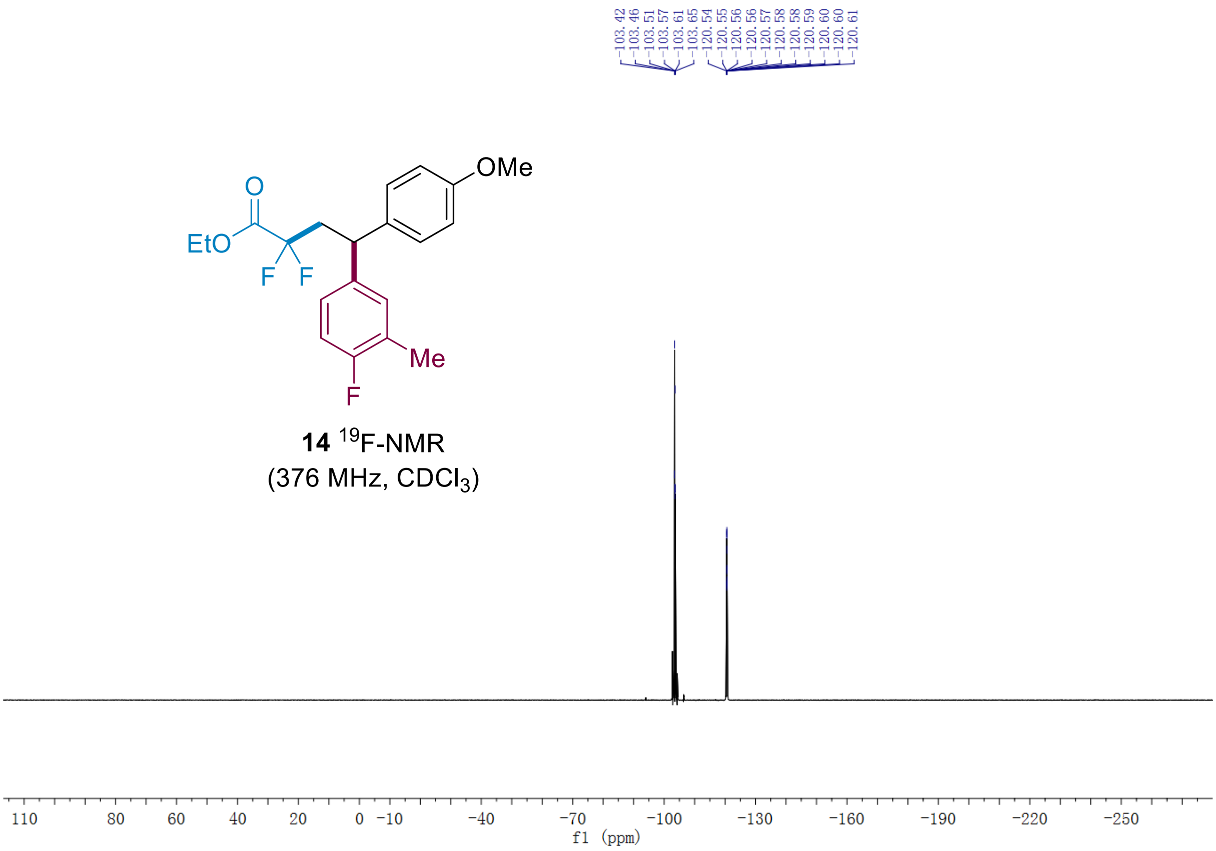

14 ^{19}F-NMR
(376 MHz, $CDCl_3$)

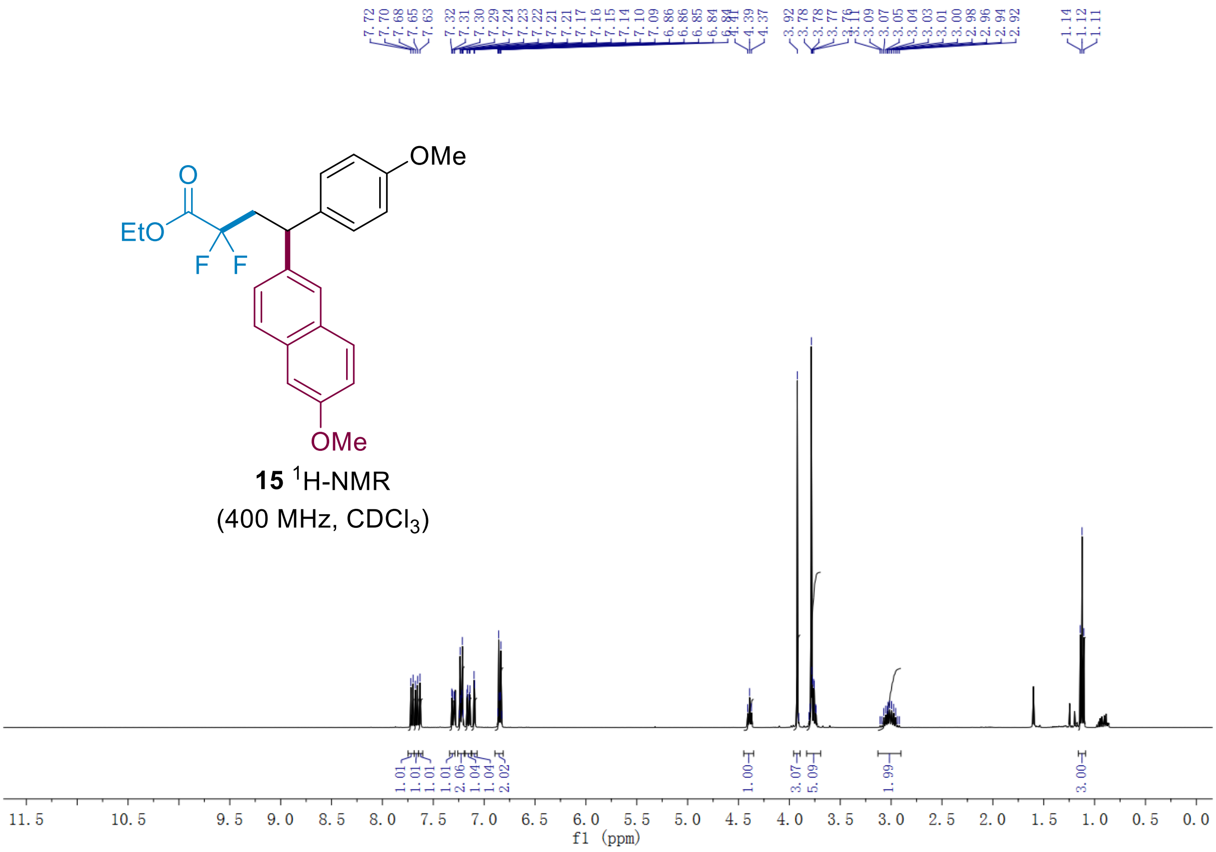

15 ^{1}H-NMR
(400 MHz, $CDCl_3$)

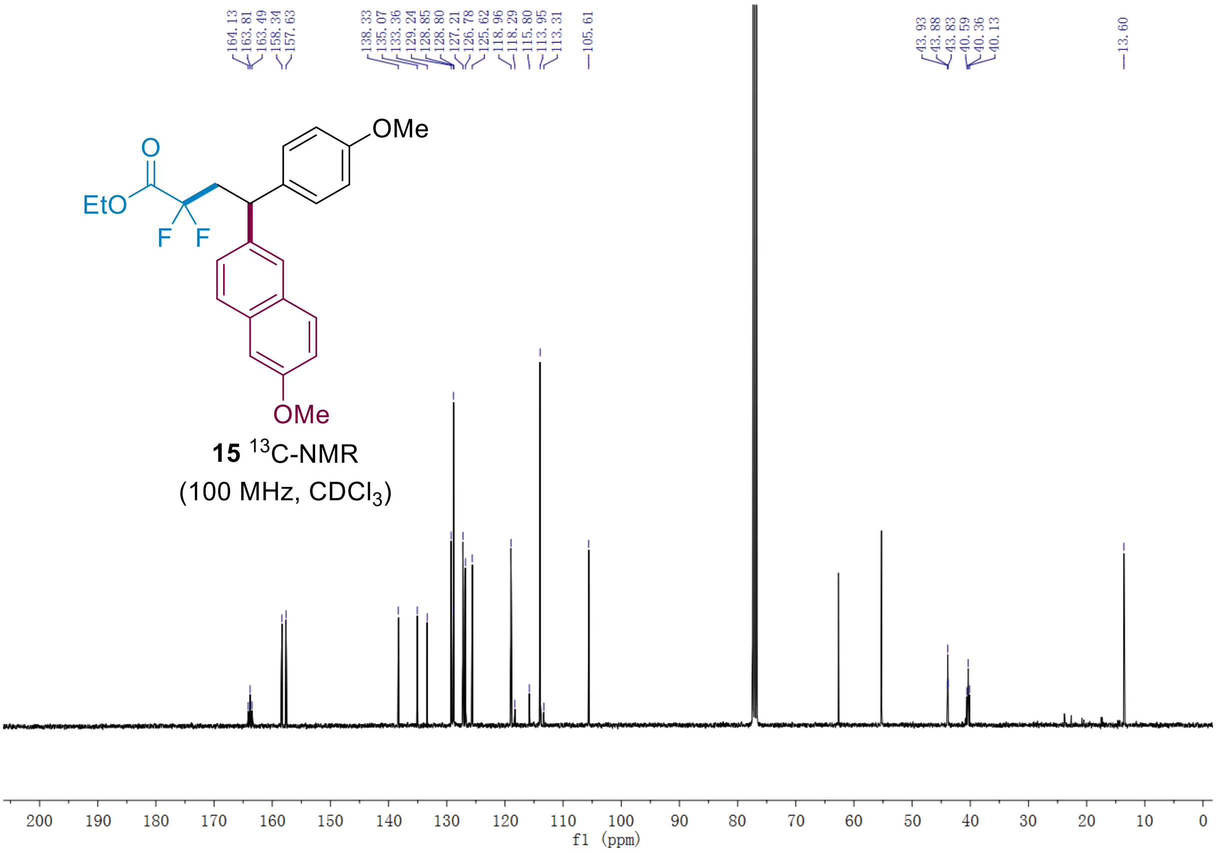
164.13
163.81
163.49
158.34
157.63
138.33
135.07
133.36
129.24
128.85
128.80
127.21
126.78
125.62
118.96
118.29
115.80
113.95
113.31
105.61
43.93
43.88
43.83
40.59
40.36
40.13
13.60
OMe
O
EtO
F F
OMe
15 13C-NMR
(100 MHz, CDCl3)
f1 (ppm)

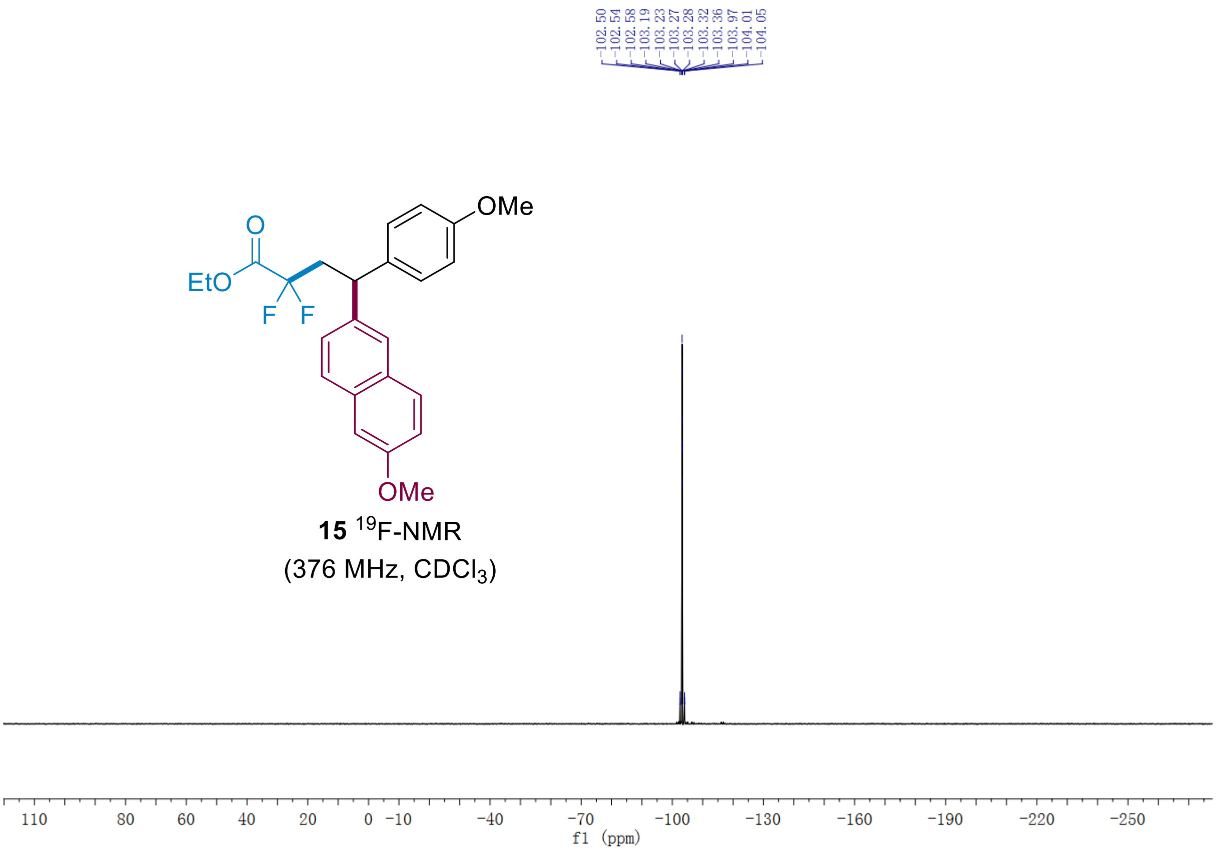
-102.50
-102.54
-102.58
-103.19
-103.23
-103.27
-103.28
-103.32
-103.36
-103.97
-104.01
-104.05
OMe
O
EtO
F F
OMe
15 19F-NMR
(376 MHz, CDCl3)
f1 (ppm)

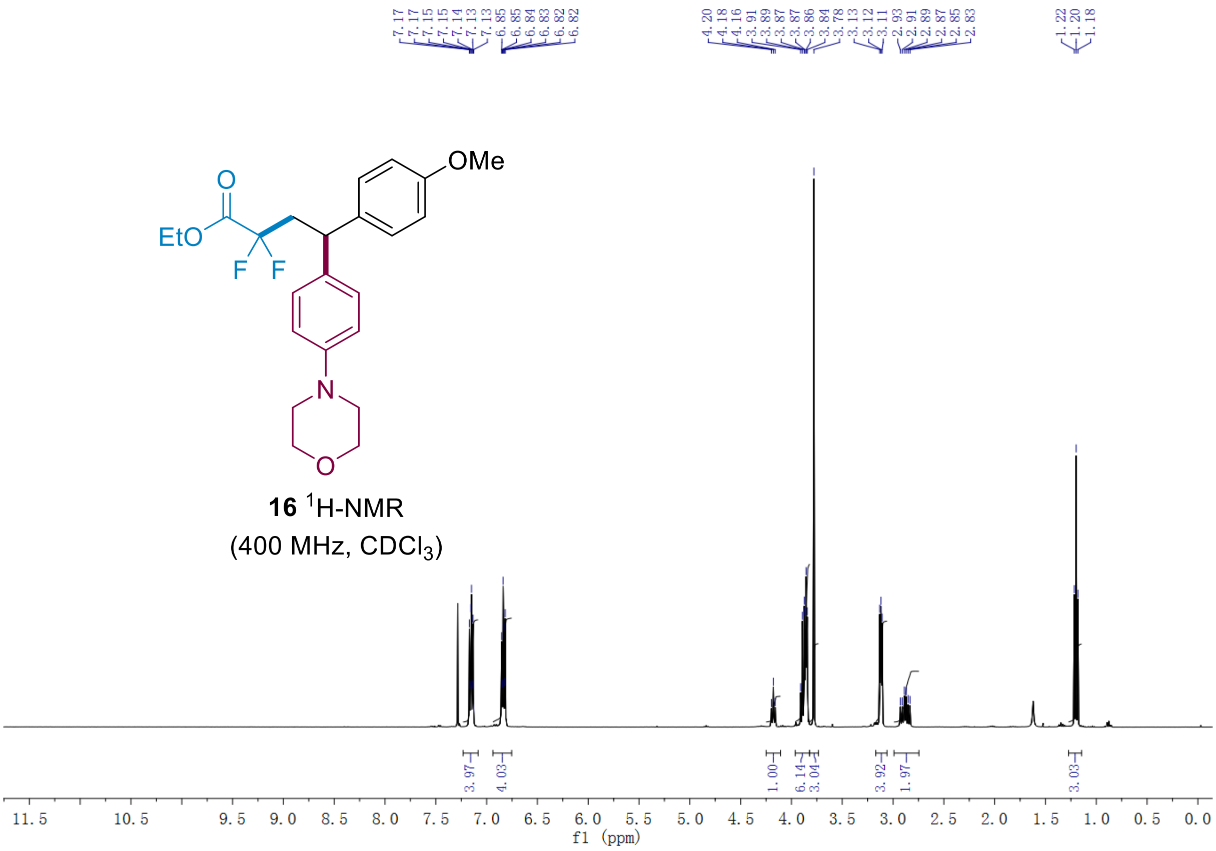
OMe
EtO
O
F
F
N
O
16 1H-NMR
(400 MHz, CDCl3)
f1 (ppm)

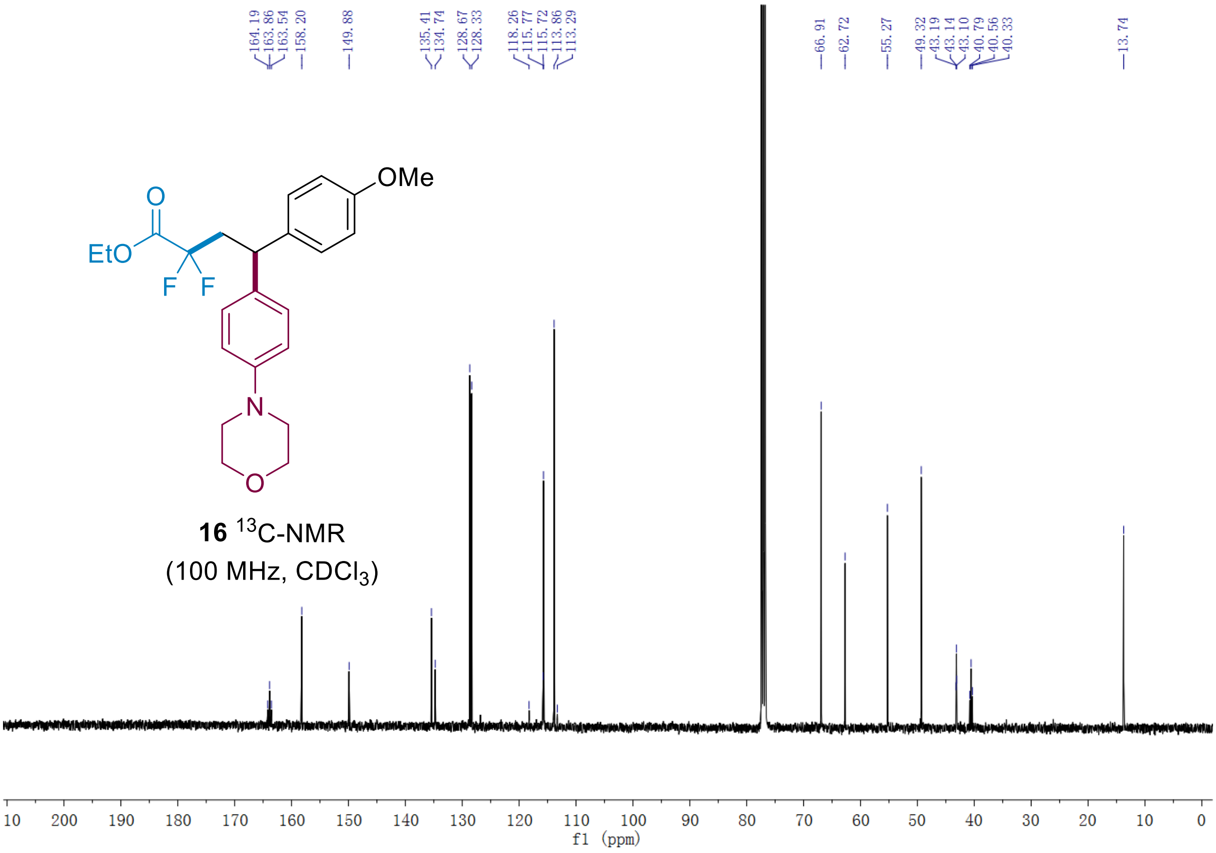
OMe
EtO
O
F
F
N
O
16 13C-NMR
(100 MHz, CDCl3)
f1 (ppm)

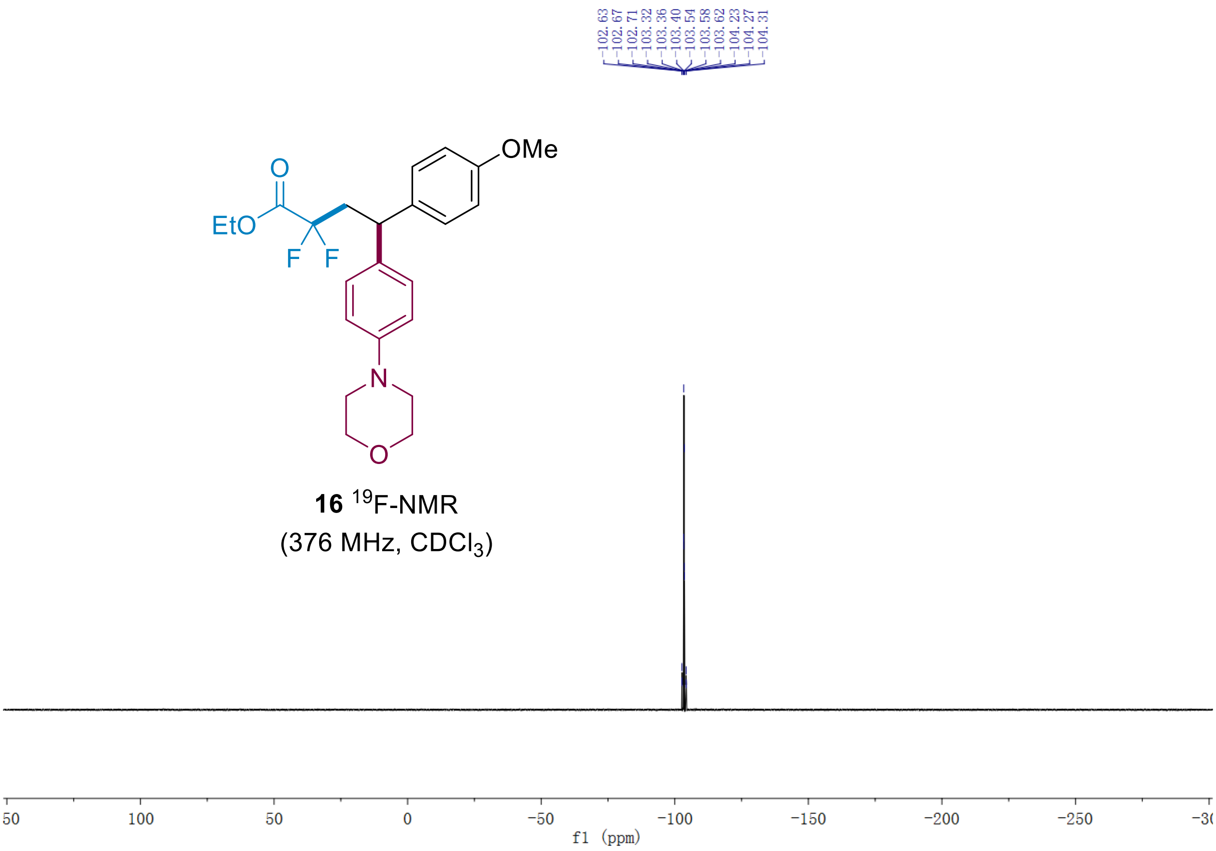

16 ^{19}F-NMR
(376 MHz, $CDCl_3$)

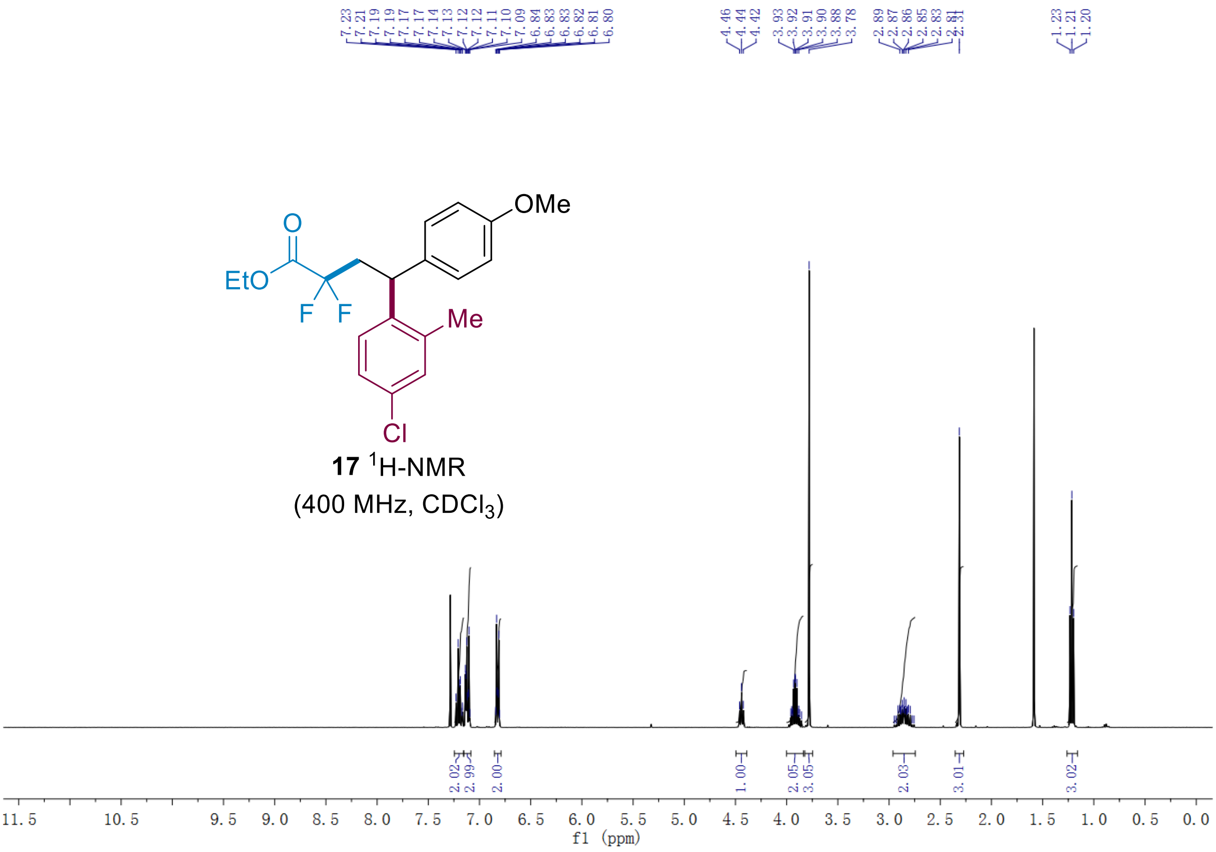

17 ^{1}H-NMR
(400 MHz, $CDCl_3$)

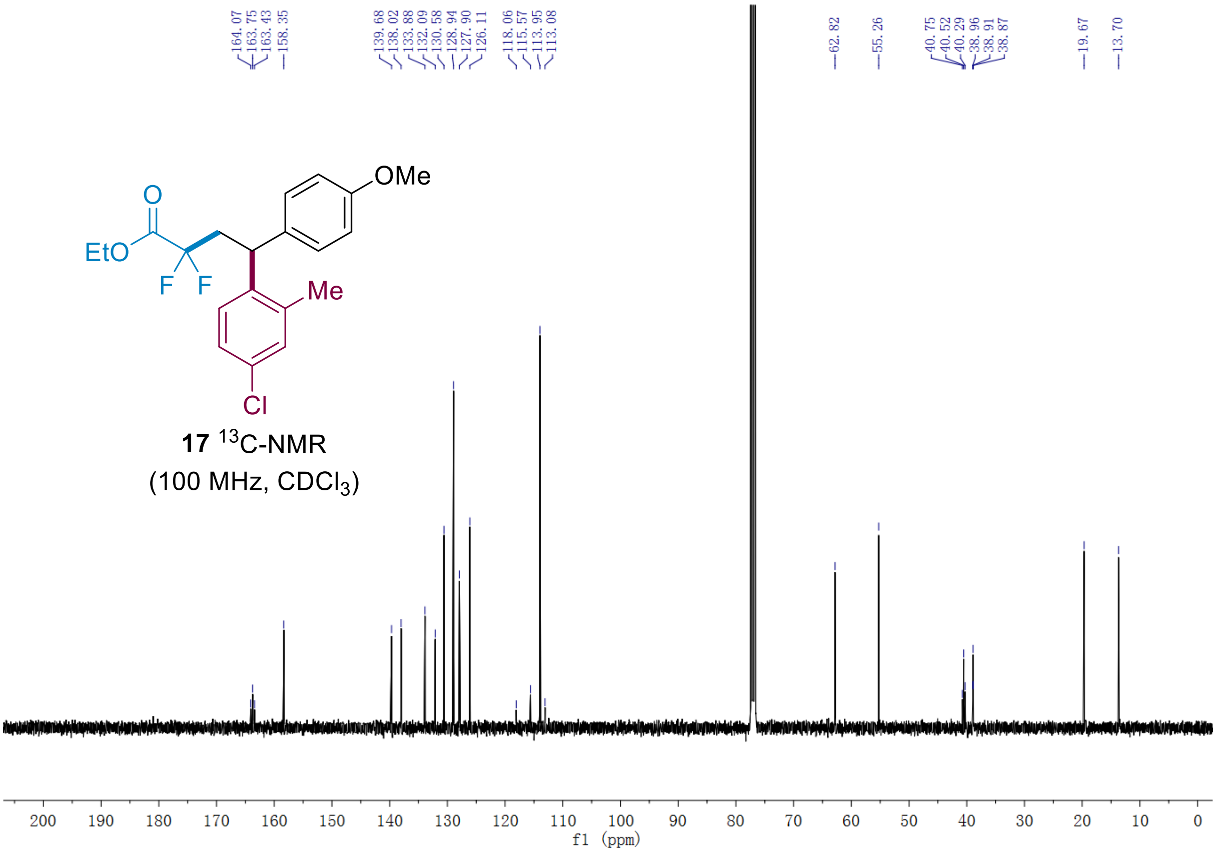
164.07
163.75
163.43
158.35
139.68
138.02
133.88
132.09
130.58
128.94
127.90
126.11
118.06
115.57
113.95
113.08
62.82
55.26
40.75
40.52
40.29
38.96
38.91
38.87
19.67
13.70
OMe
O
EtO
F F
Me
Cl
17 ¹³C-NMR
(100 MHz, CDCl₃)
200 190 180 170 160 150 140 130 120 110 100 90 80 70 60 50 40 30 20 10 0
f1 (ppm)

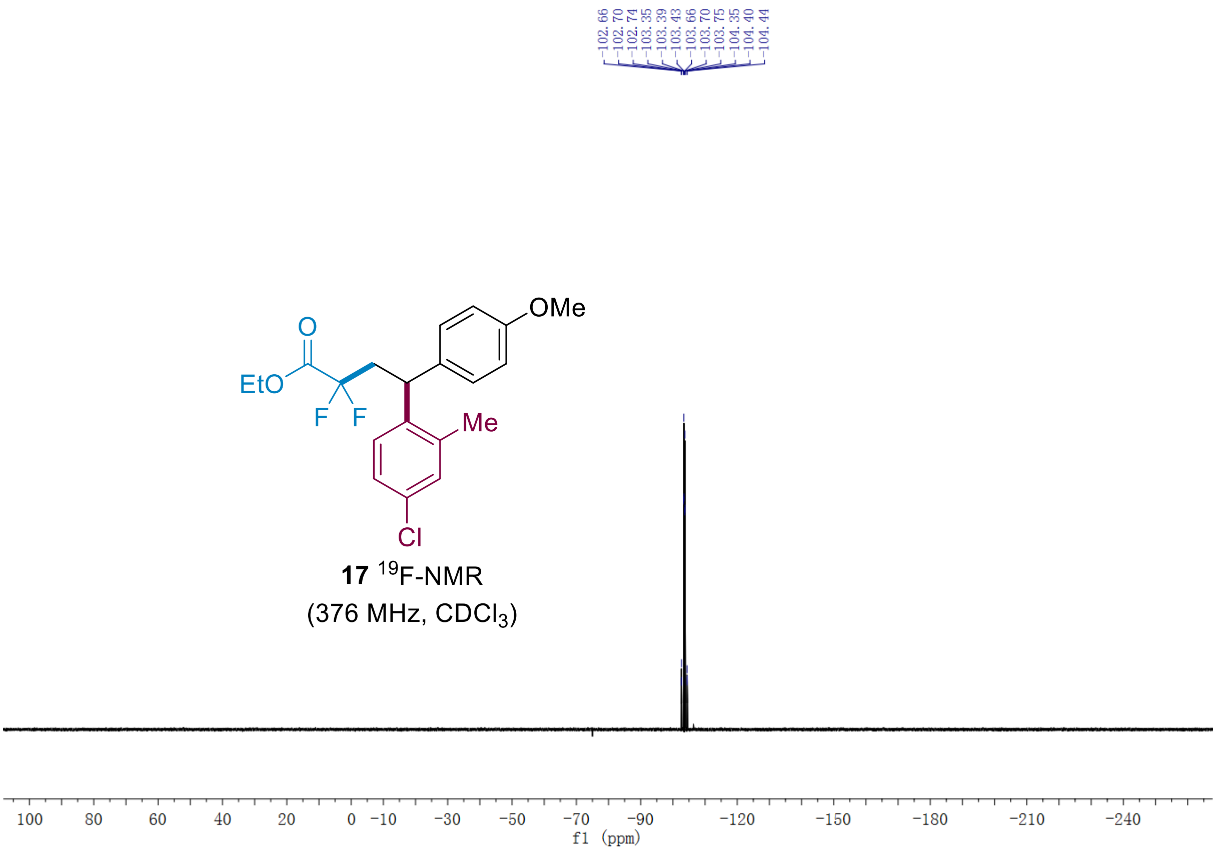
-102.66
-102.70
-102.74
-103.35
-103.39
-103.43
-103.66
-103.70
-103.75
-104.35
-104.40
-104.44
OMe
O
EtO
F F
Me
Cl
17 ¹⁹F-NMR
(376 MHz, CDCl₃)
100 80 60 40 20 0 -10 -30 -50 -70 -90 -120 -150 -180 -210 -240
f1 (ppm)

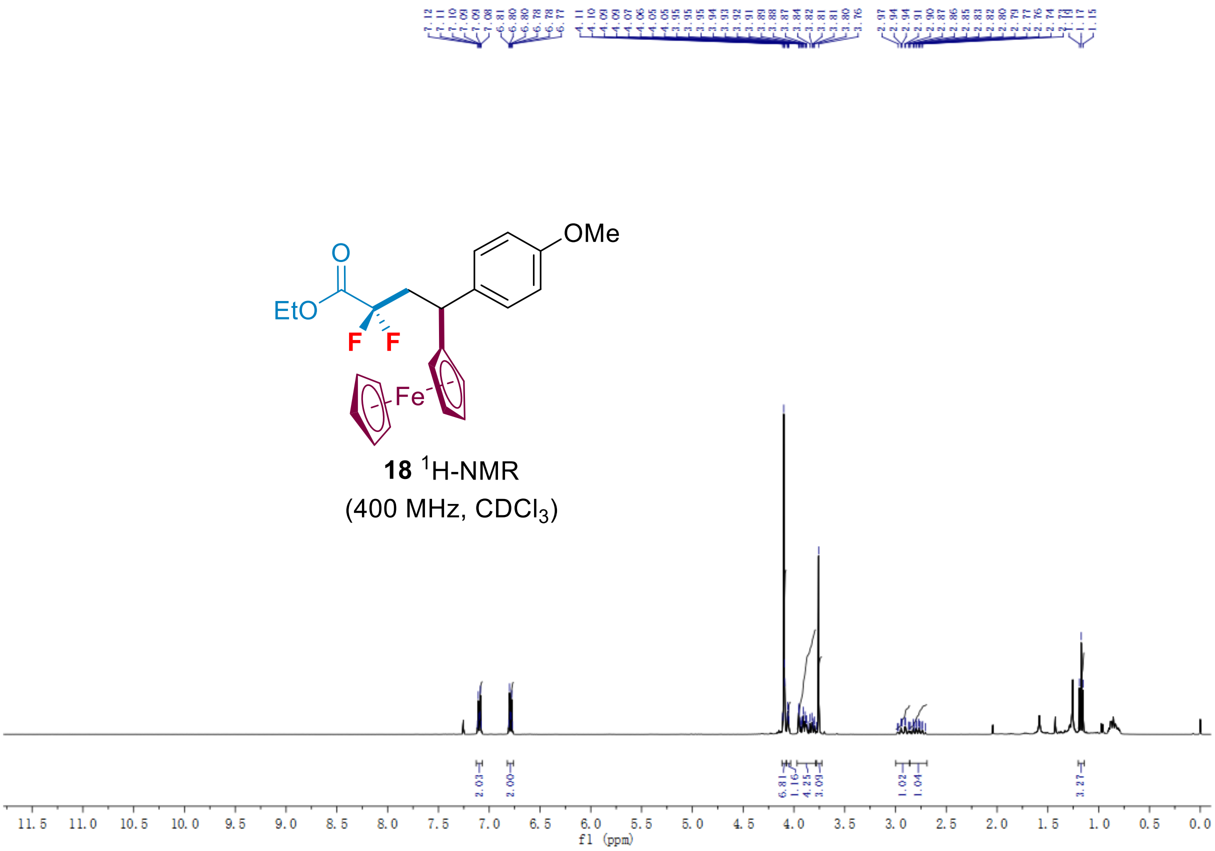
OMe
O
EtO
F F
Fe
18 1H-NMR
(400 MHz, CDCl3)
f1 (ppm)

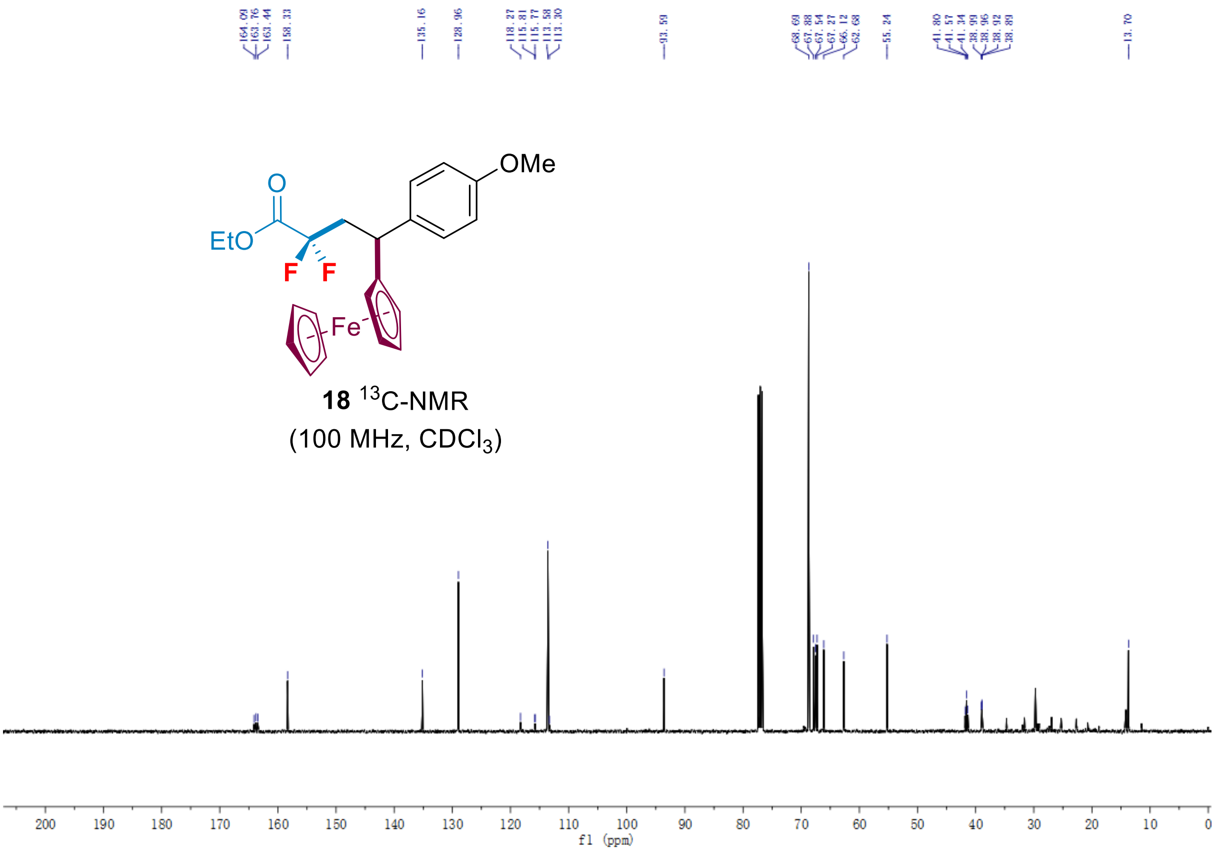
OMe
O
EtO
F F
Fe
18 13C-NMR
(100 MHz, CDCl3)
f1 (ppm)

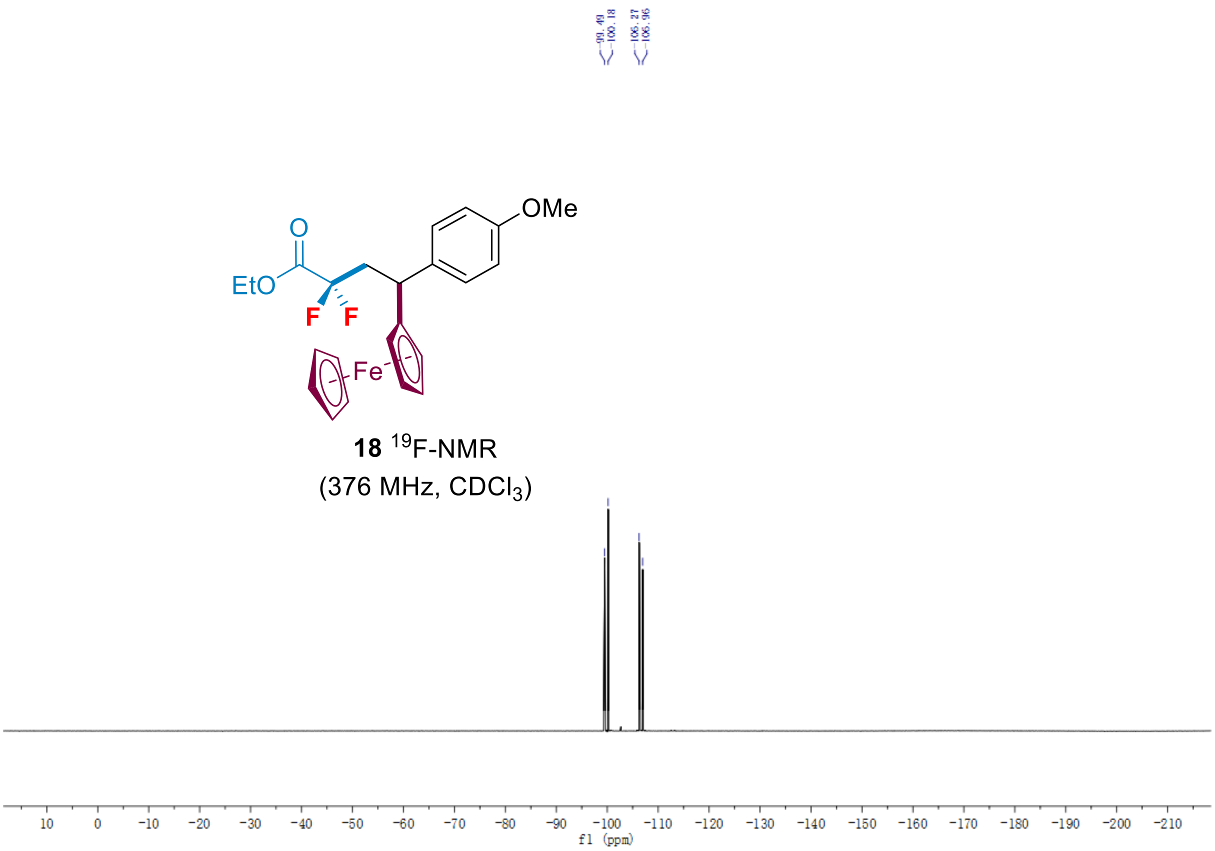
OMe
EtO
O
F
F
Fe
18 19F-NMR
(376 MHz, CDCl3)
f1 (ppm)

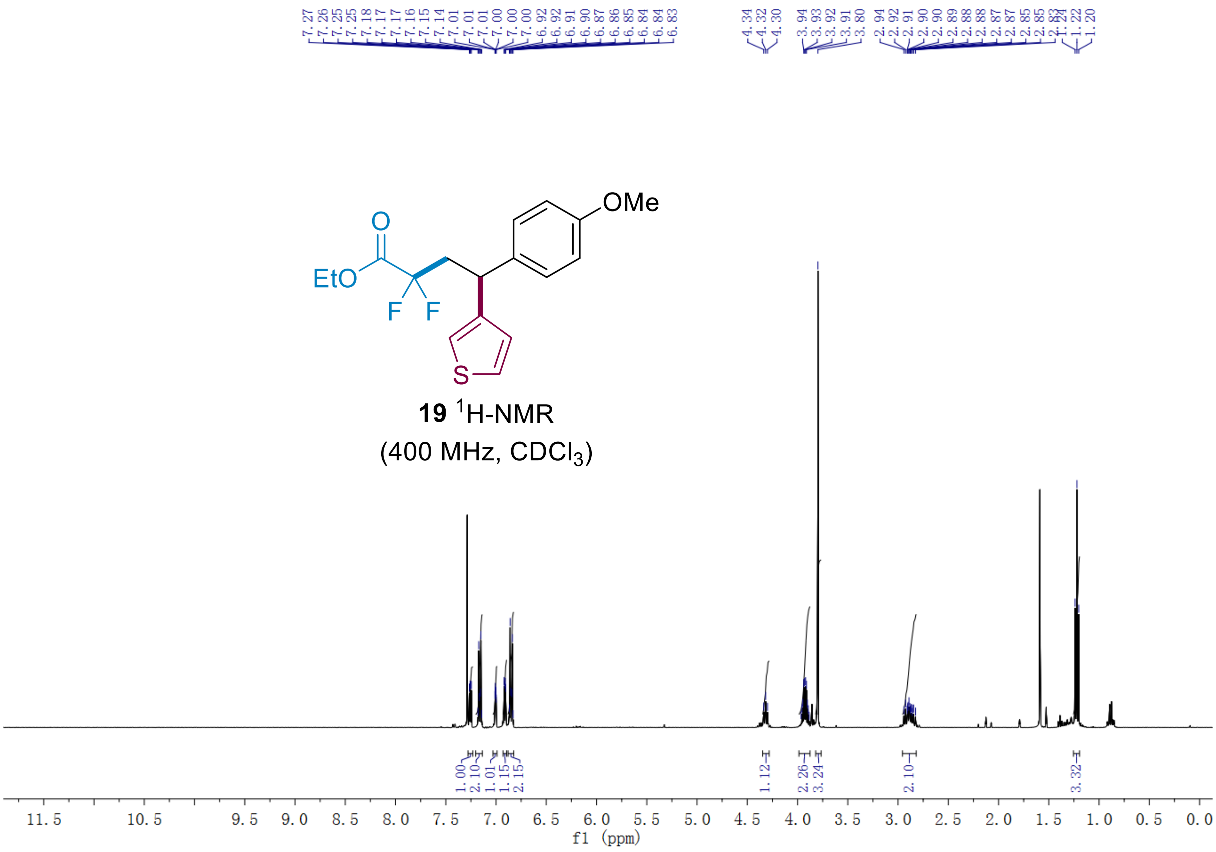
OMe
EtO
O
F
F
S
19 1H-NMR
(400 MHz, CDCl3)
f1 (ppm)

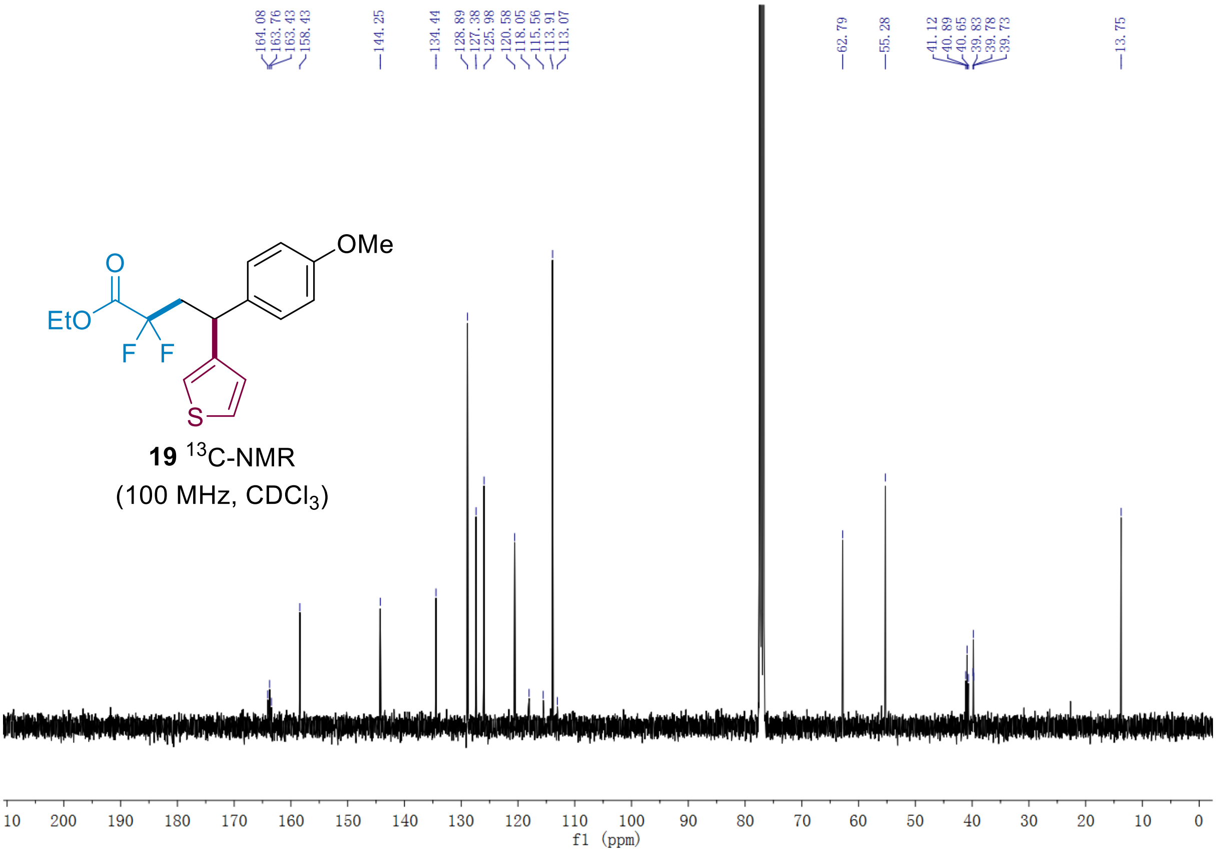

164.08
163.76
163.43
158.43
144.25
134.44
128.89
127.38
125.98
120.58
118.05
115.56
113.91
113.07
62.79
55.28
41.12
40.89
40.65
39.83
39.78
39.73
13.75
OMe
O
EtO
F F
S
19 13C-NMR
(100 MHz, CDCl3)
f1 (ppm)

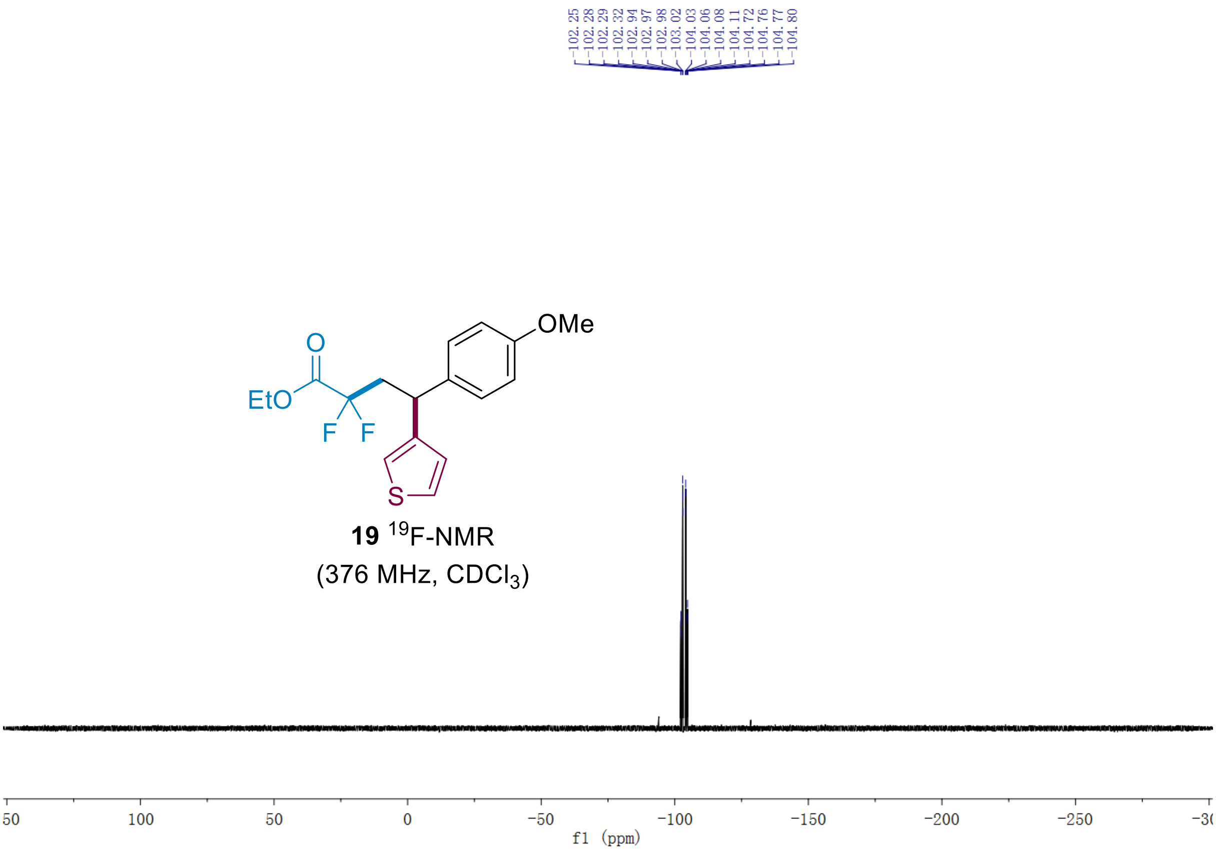

-102.25
-102.28
-102.29
-102.32
-102.94
-102.97
-102.98
-103.02
-104.03
-104.06
-104.08
-104.11
-104.72
-104.76
-104.77
-104.80
OMe
O
EtO
F F
S
19 19F-NMR
(376 MHz, CDCl3)
f1 (ppm)

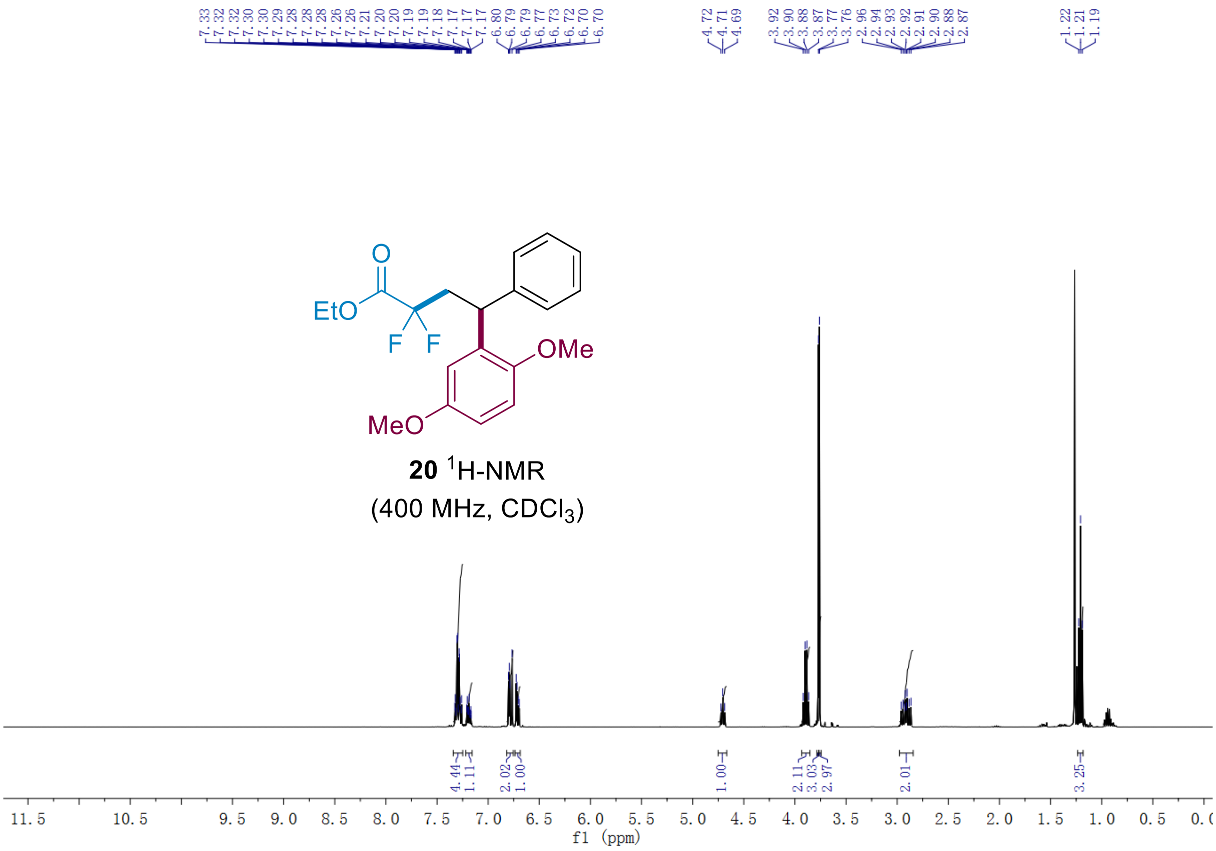

EtO
O
F F
OMe
MeO
20 ¹H-NMR
(400 MHz, CDCl₃)
f1 (ppm)

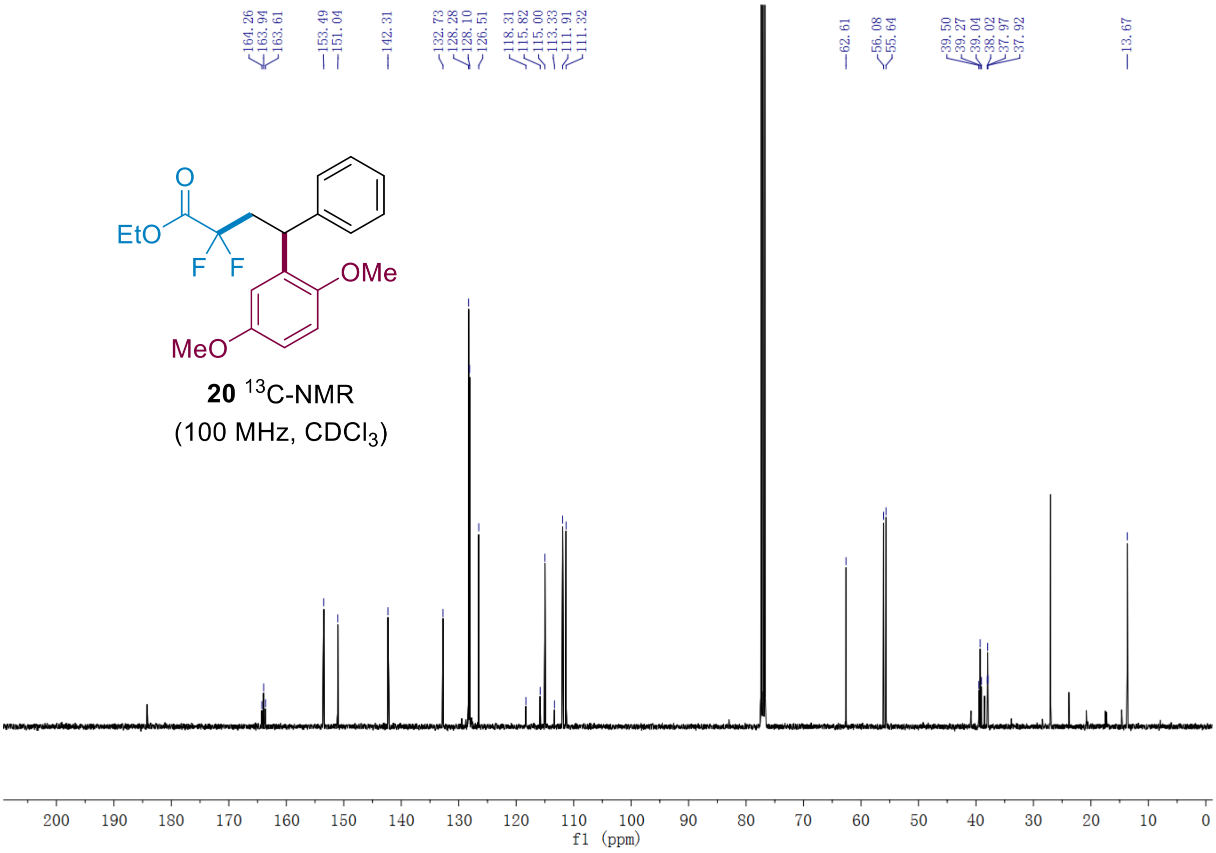

EtO
O
F F
OMe
MeO
20 ¹³C-NMR
(100 MHz, CDCl₃)
f1 (ppm)

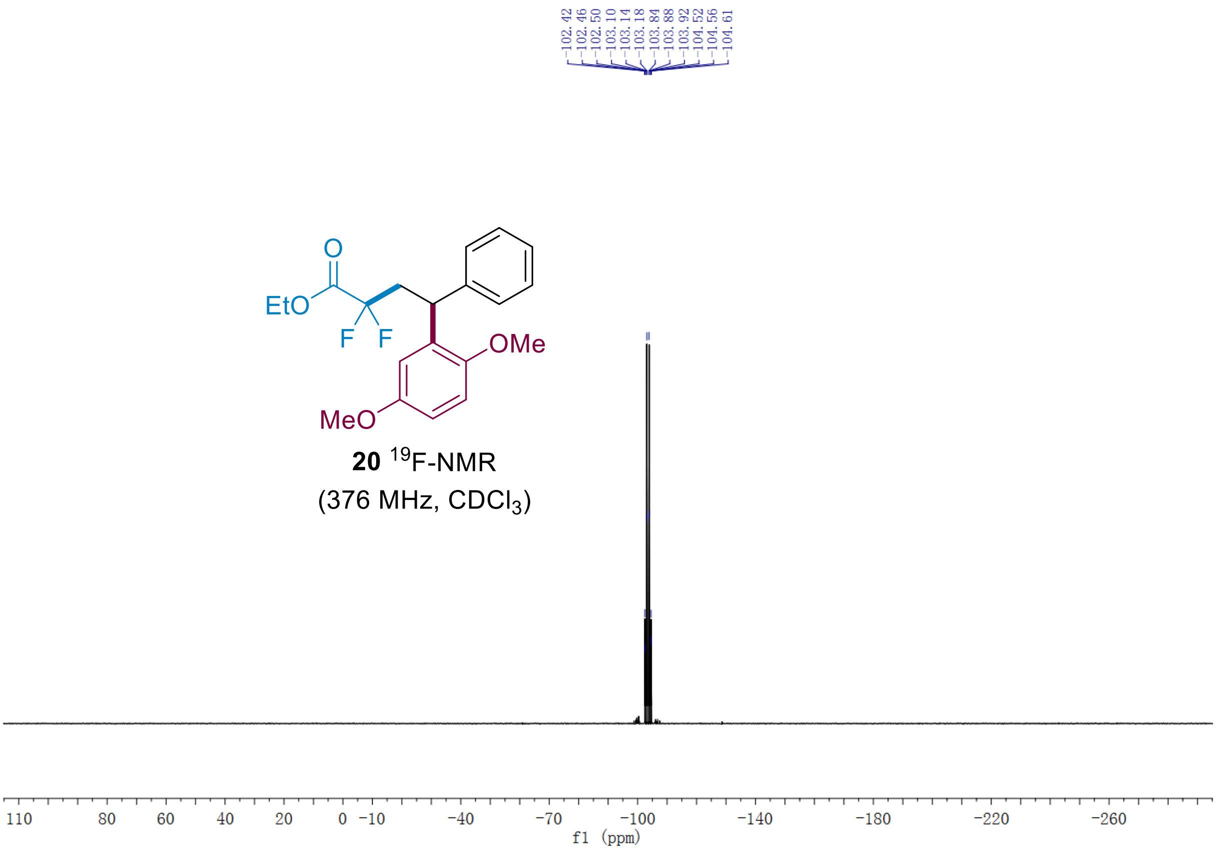

20 ^{19}F-NMR
(376 MHz, $CDCl_3$)

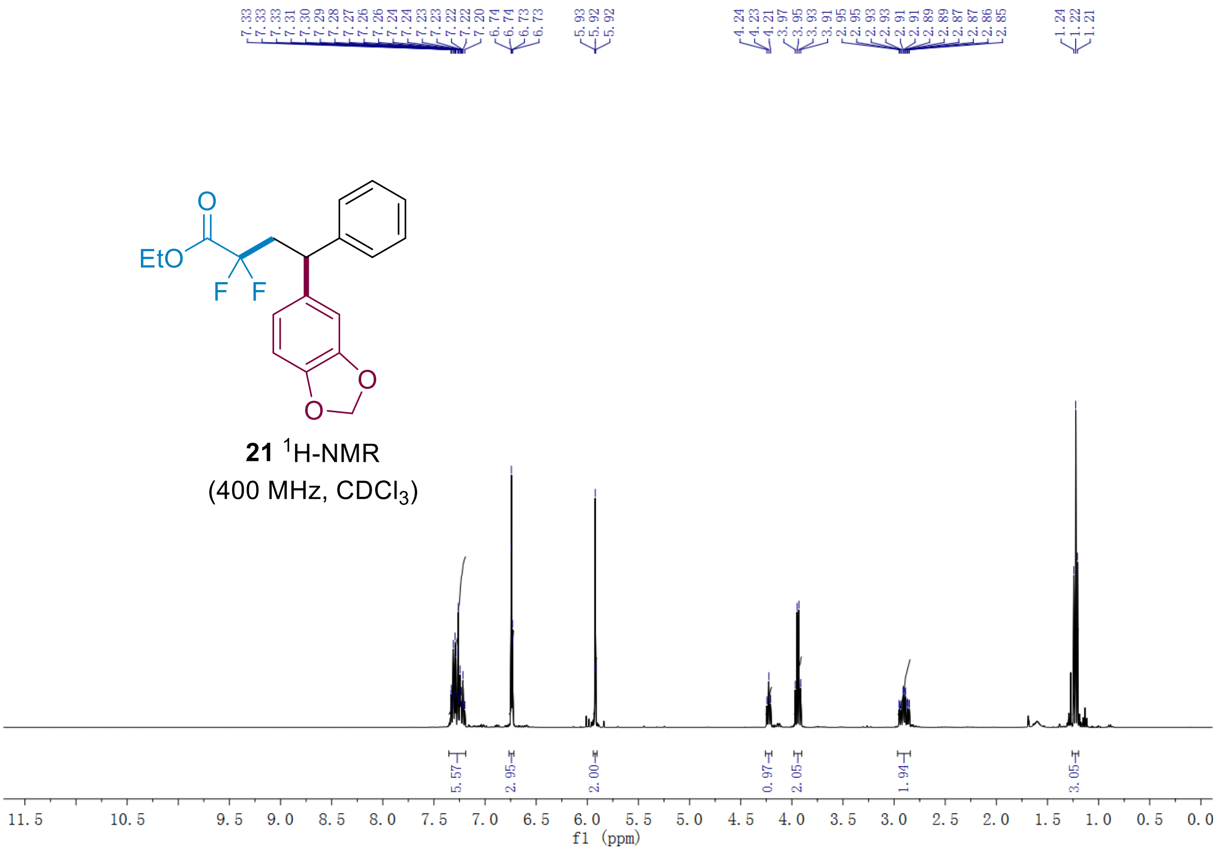

21 ^{1}H-NMR
(400 MHz, $CDCl_3$)

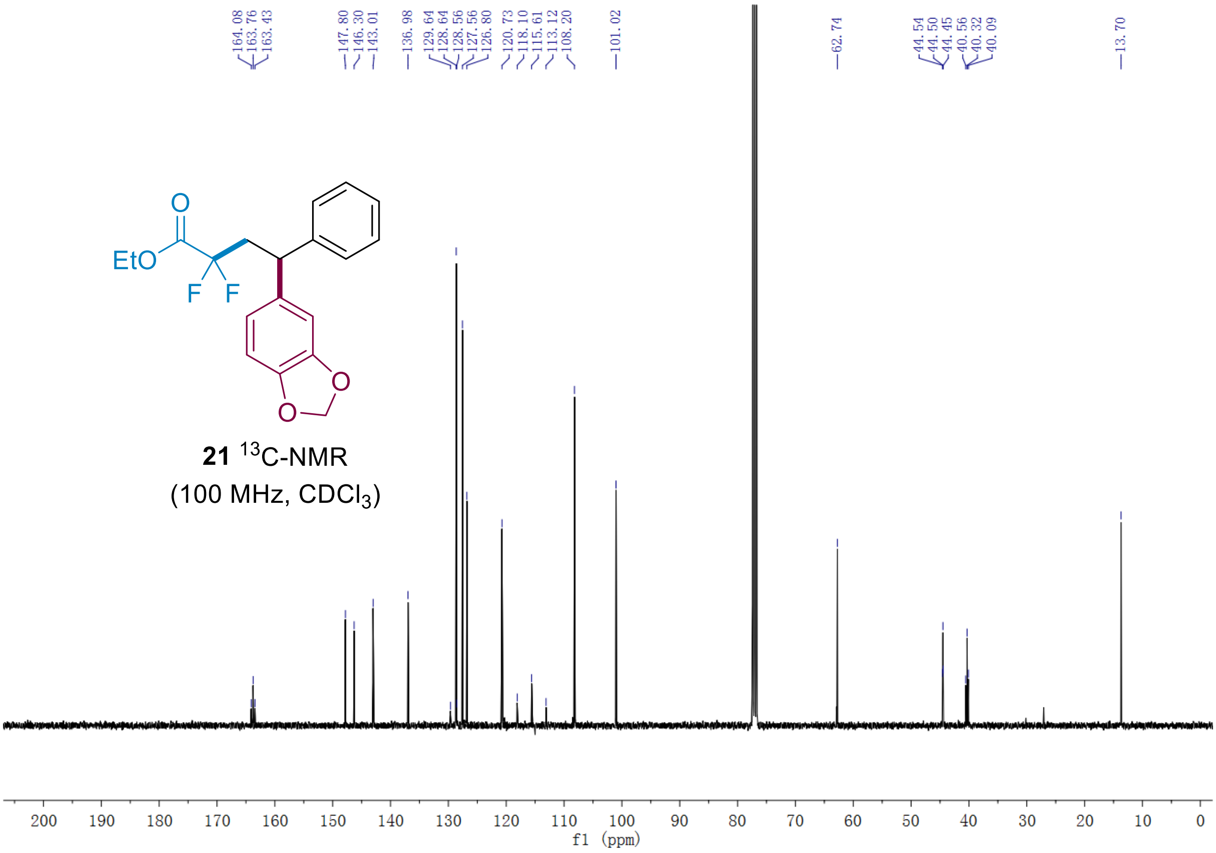
164.08
163.76
163.43
147.80
146.30
143.01
136.98
129.64
128.64
128.56
127.56
126.80
120.73
118.10
115.61
113.12
108.20
101.02
62.74
44.54
44.50
44.45
40.56
40.32
40.09
13.70
EtO
O
F F
O
O
21 ^{13}C-NMR
(100 MHz, $CDCl_3$)
f1 (ppm)

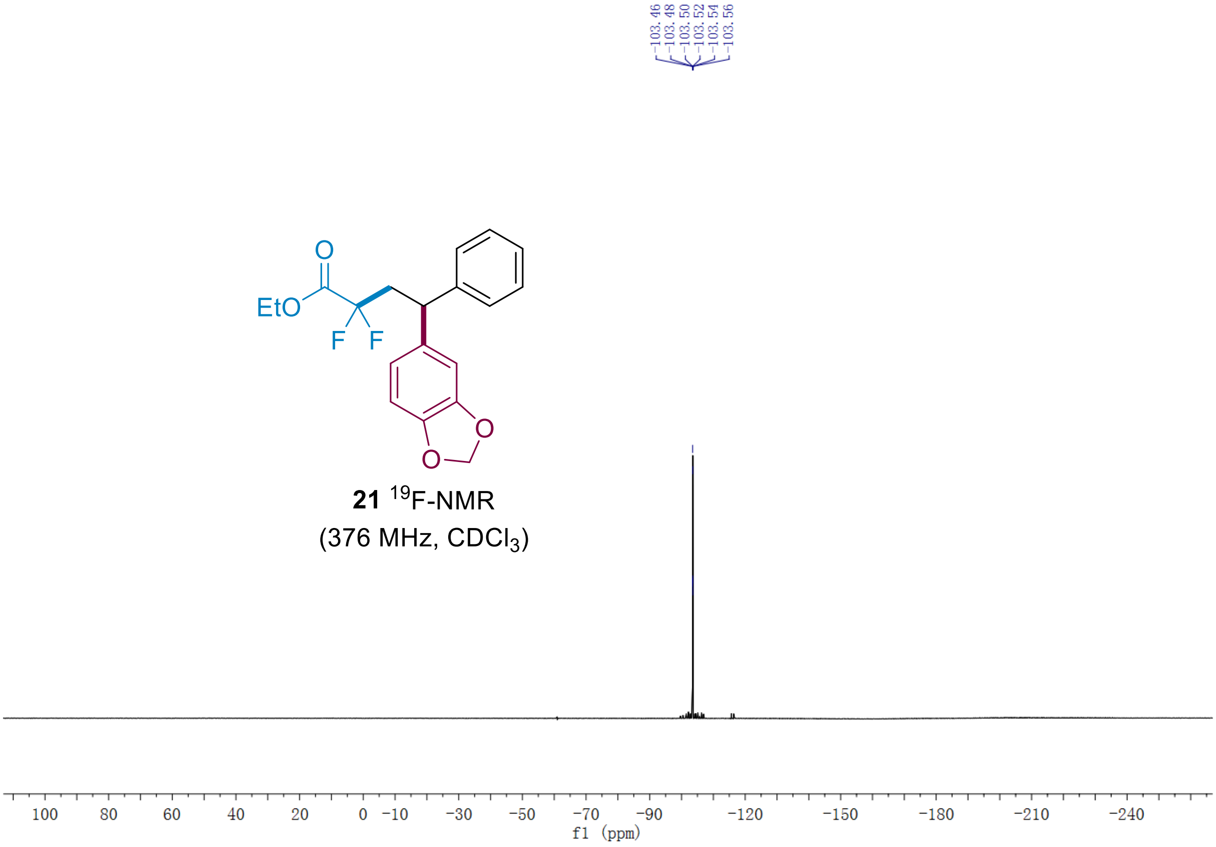
-103.46
-103.48
-103.50
-103.52
-103.54
-103.56
EtO
O
F F
O
O
21 ^{19}F-NMR
(376 MHz, $CDCl_3$)
f1 (ppm)

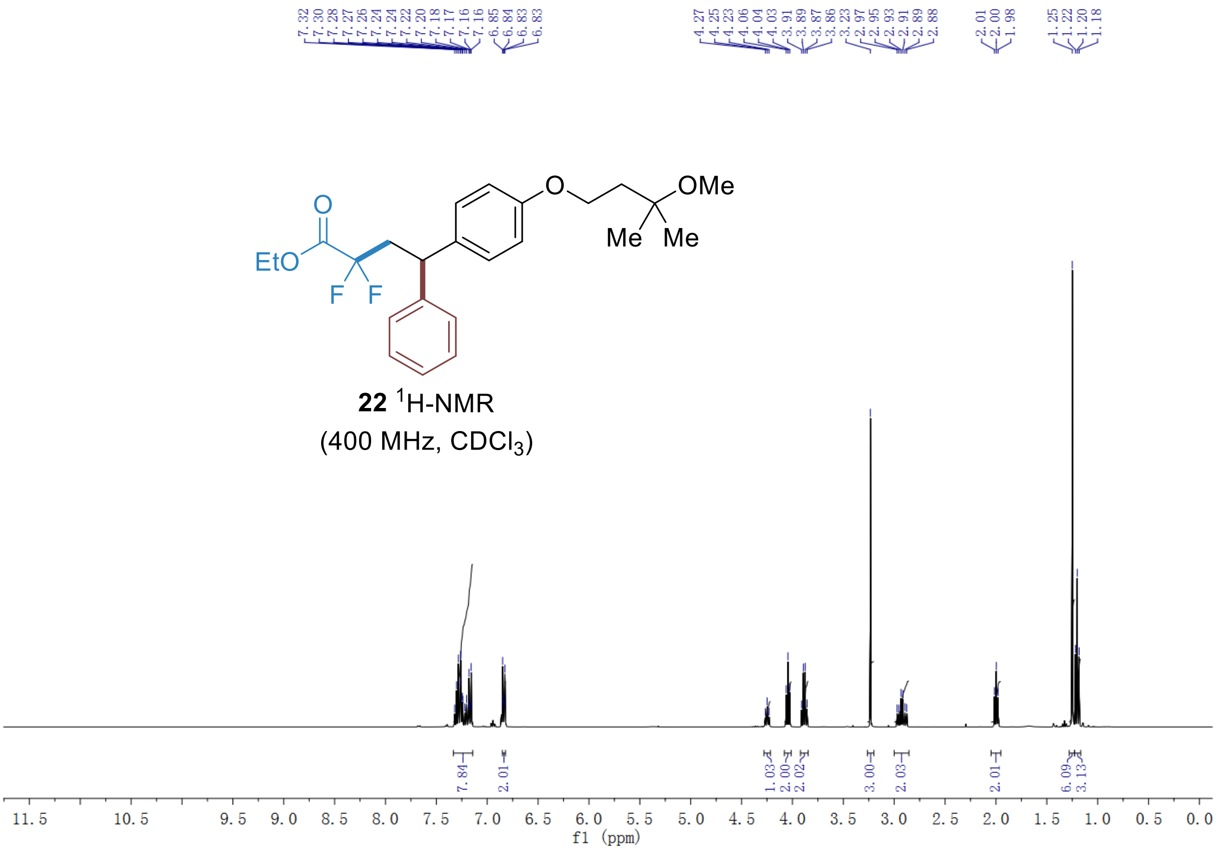
7.32 7.30 7.28 7.27 7.26 7.24 7.24 7.22 7.20 7.18 7.17 7.16 7.16 6.85 6.84 6.83 6.83
4.27 4.25 4.23 4.06 4.04 4.03 3.91 3.89 3.87 3.86 3.23 2.97 2.95 2.93 2.91 2.89 2.88
2.01 2.00 1.98
1.25 1.22 1.20 1.18
EtO
O
F F
O
OMe
Me Me
22 ¹H-NMR
(400 MHz, CDCl₃)
7.84
2.01
1.03 2.00 2.02
3.00
2.03
2.01
6.09 3.13
f1 (ppm)

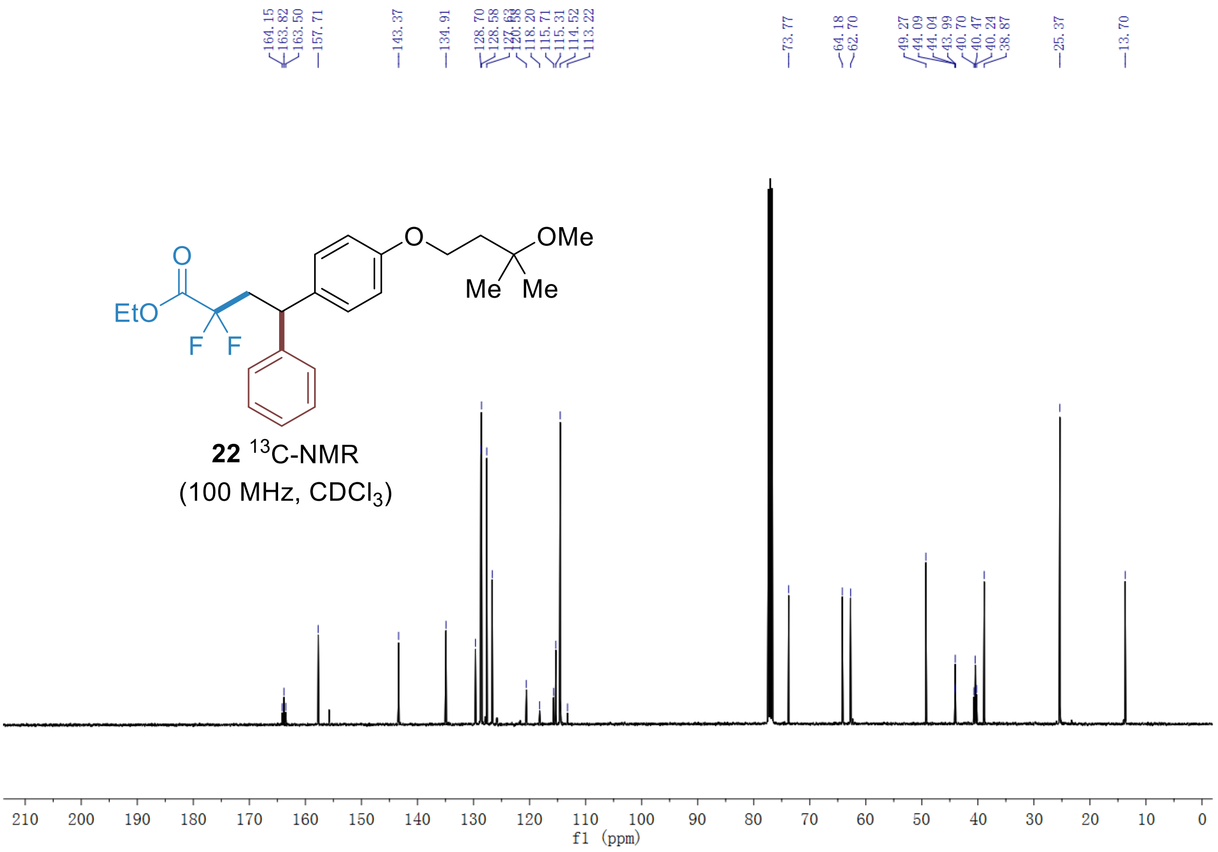
164.15 163.82 163.50 157.71
143.37
134.91
128.70 128.58 118.20 115.71 115.31 114.52 113.22
73.77
64.18 62.70
49.27 44.09 44.04 43.99 40.70 40.47 40.24 38.87
25.37
13.70
EtO
O
F F
O
OMe
Me Me
22 ¹³C-NMR
(100 MHz, CDCl₃)
f1 (ppm)

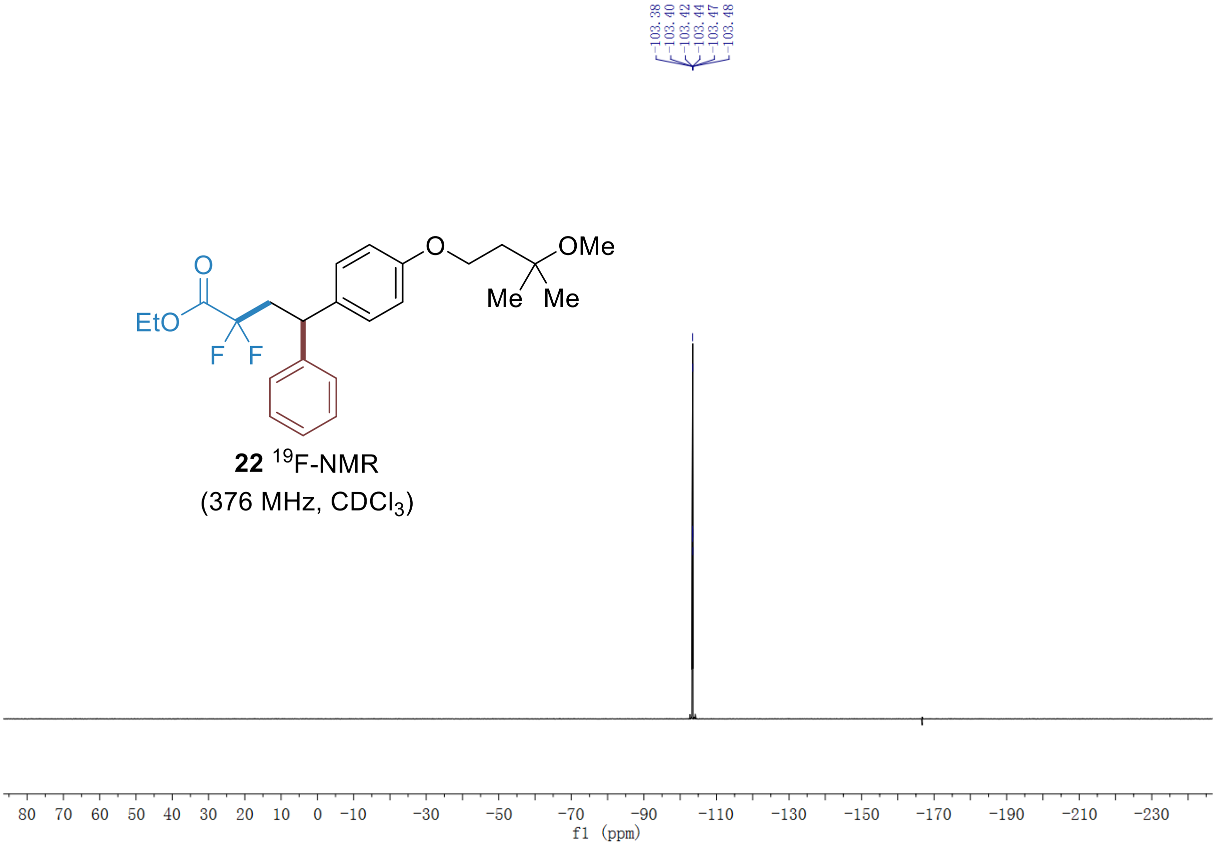
-103.38
-103.40
-103.42
-103.44
-103.47
-103.48
OMe
EtO
O
Me Me
F F
22 19F-NMR
(376 MHz, CDCl3)
f1 (ppm)

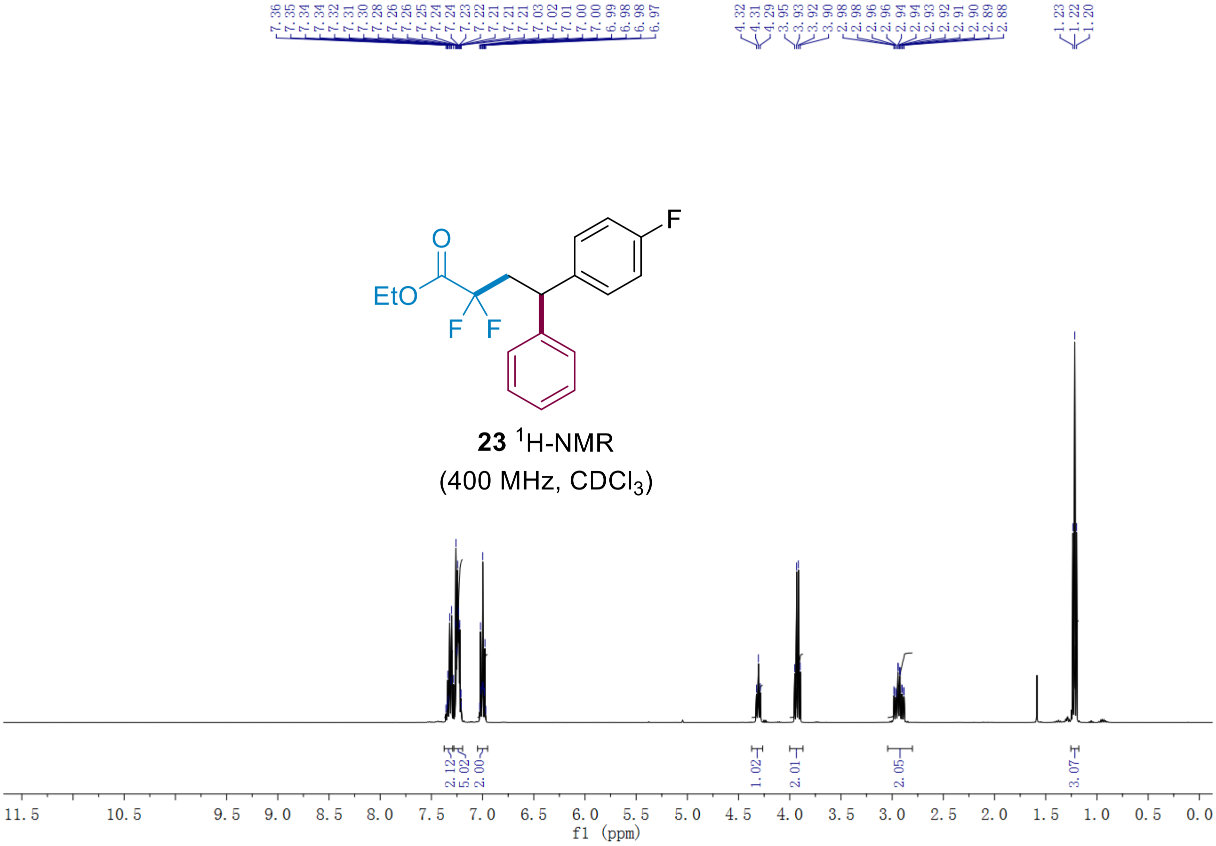
7.36 7.35 7.34 7.32 7.31 7.30 7.28 7.26 7.25 7.24 7.24 7.23 7.22 7.21 7.21 7.21 7.03 7.02 7.01 7.00 6.99 6.98 6.98 6.97
4.32 4.31 4.29 3.95 3.93 3.92 3.90 2.98 2.98 2.96 2.96 2.94 2.94 2.93 2.92 2.91 2.90 2.89 2.88
1.23 1.22 1.20
EtO
O
F
F F
23 1H-NMR
(400 MHz, CDCl3)
2.12 5.02 2.00 1.02 2.01 2.05 3.07
f1 (ppm)

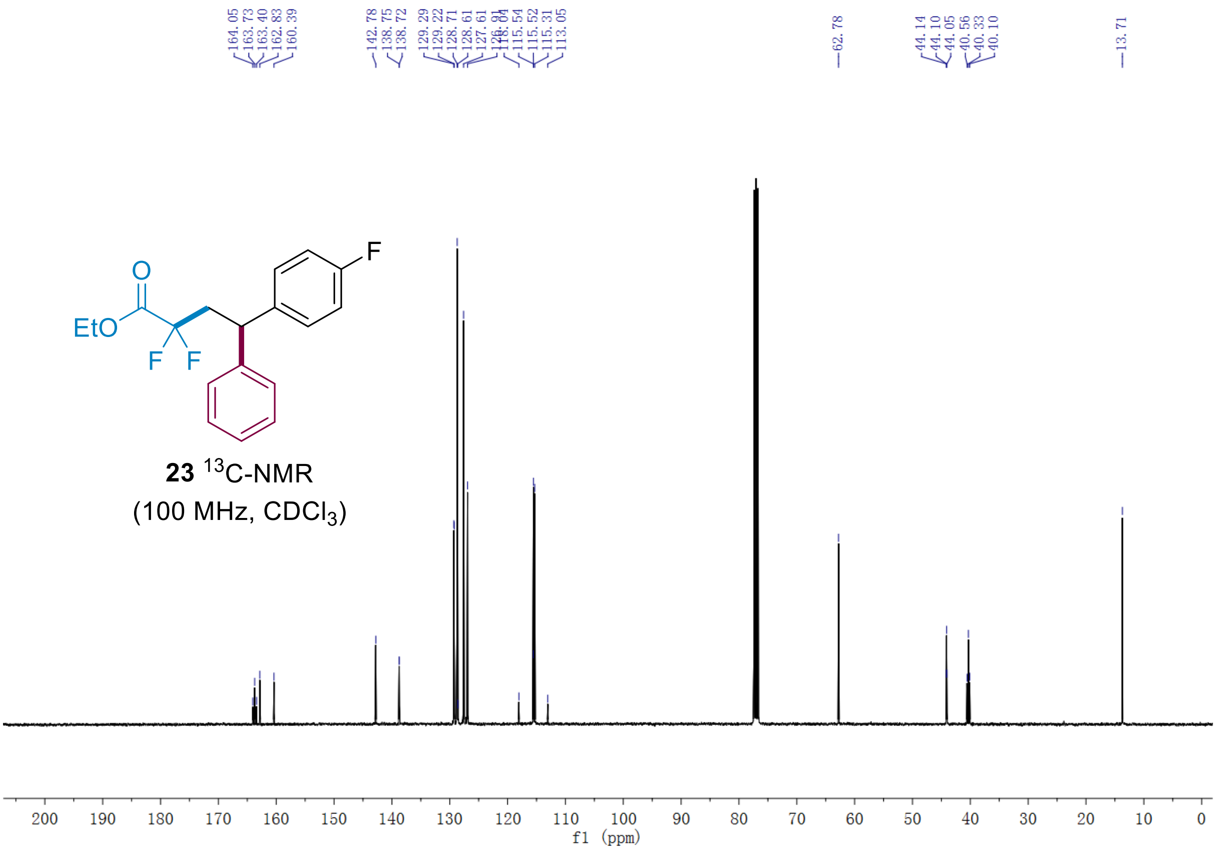
164.05
163.73
163.40
162.83
160.39
142.78
138.75
138.72
129.29
129.22
128.71
128.61
127.61
126.91
118.04
115.54
115.52
115.31
113.05
62.78
44.14
44.10
44.05
40.56
40.33
40.10
13.71
EtO
O
F
F
F
23 13C-NMR
(100 MHz, CDCl3)
f1 (ppm)

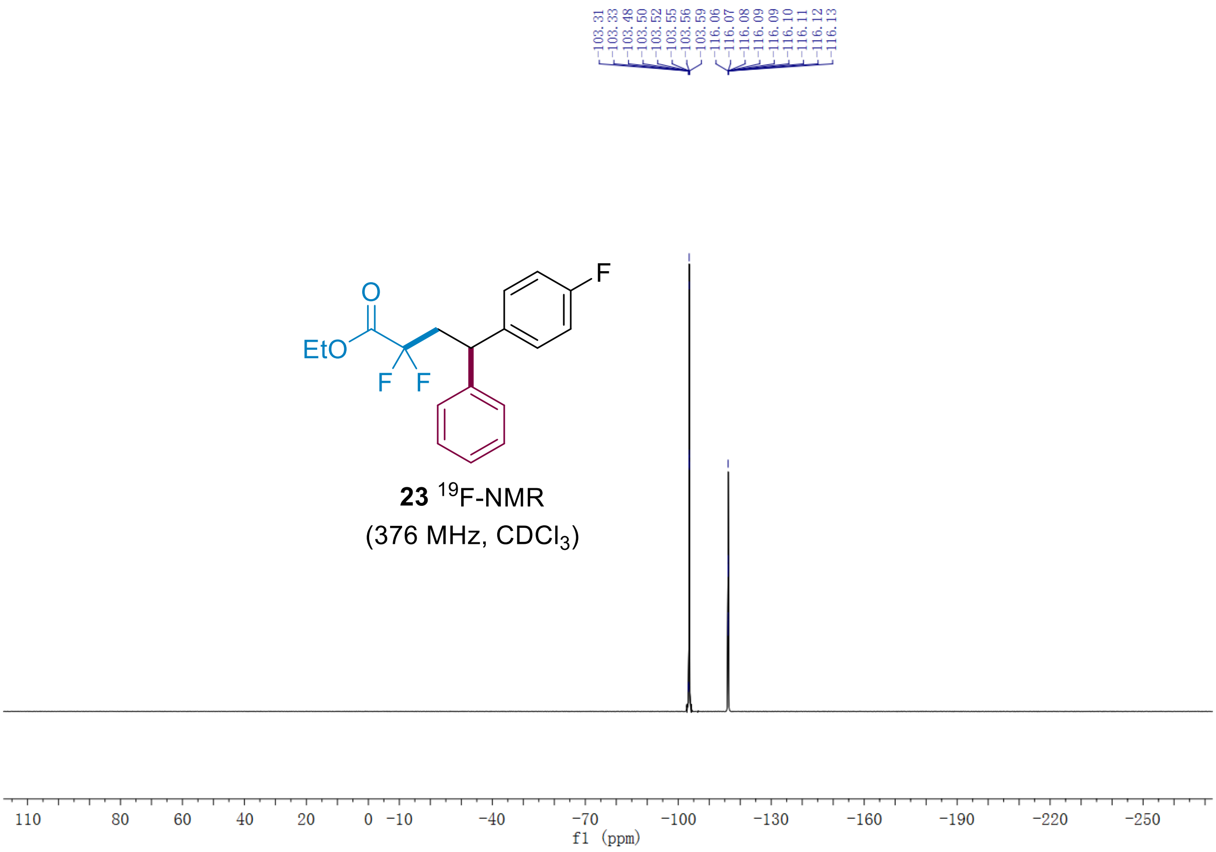
-103.31
-103.33
-103.48
-103.50
-103.52
-103.55
-103.56
-103.59
-116.06
-116.07
-116.08
-116.09
-116.10
-116.11
-116.12
-116.13
EtO
O
F
F
F
23 19F-NMR
(376 MHz, CDCl3)
f1 (ppm)

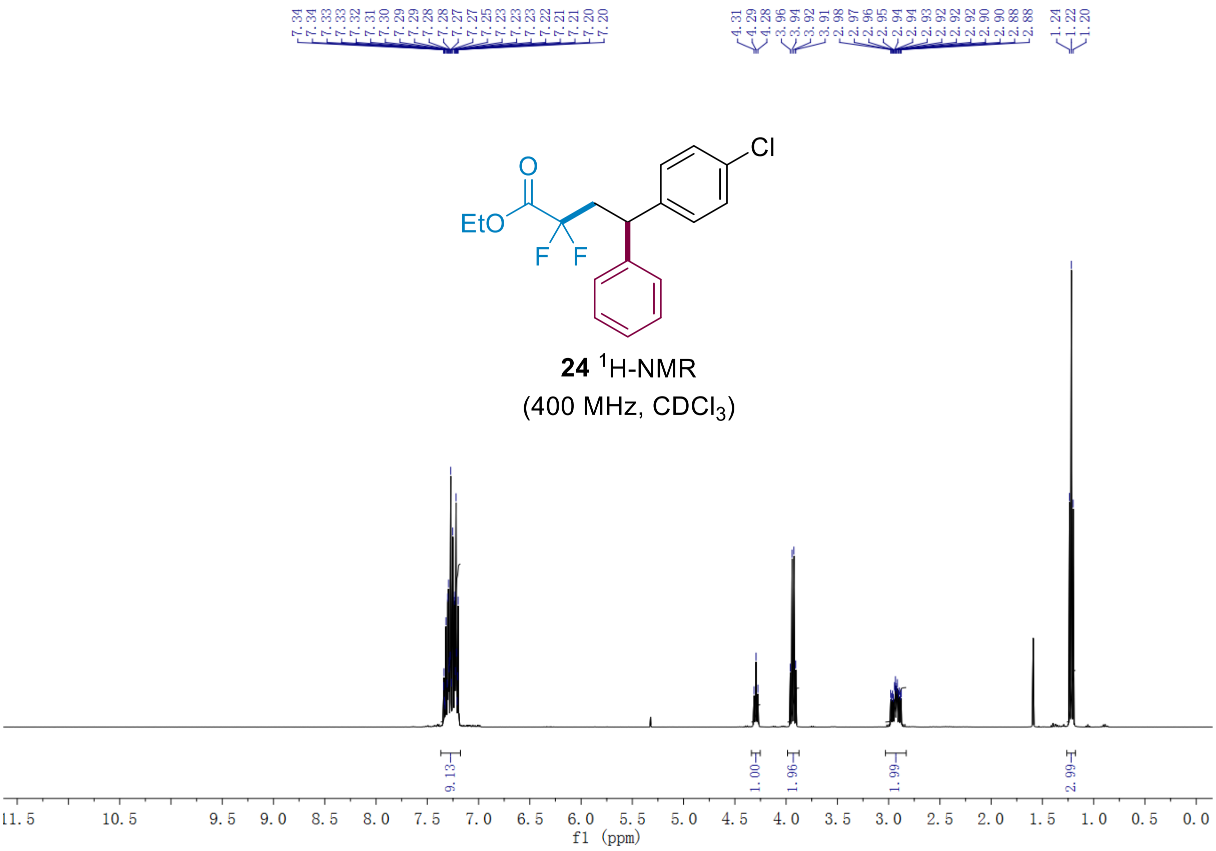
O
EtO
F F
Cl
24 1H-NMR
(400 MHz, CDCl3)
f1 (ppm)

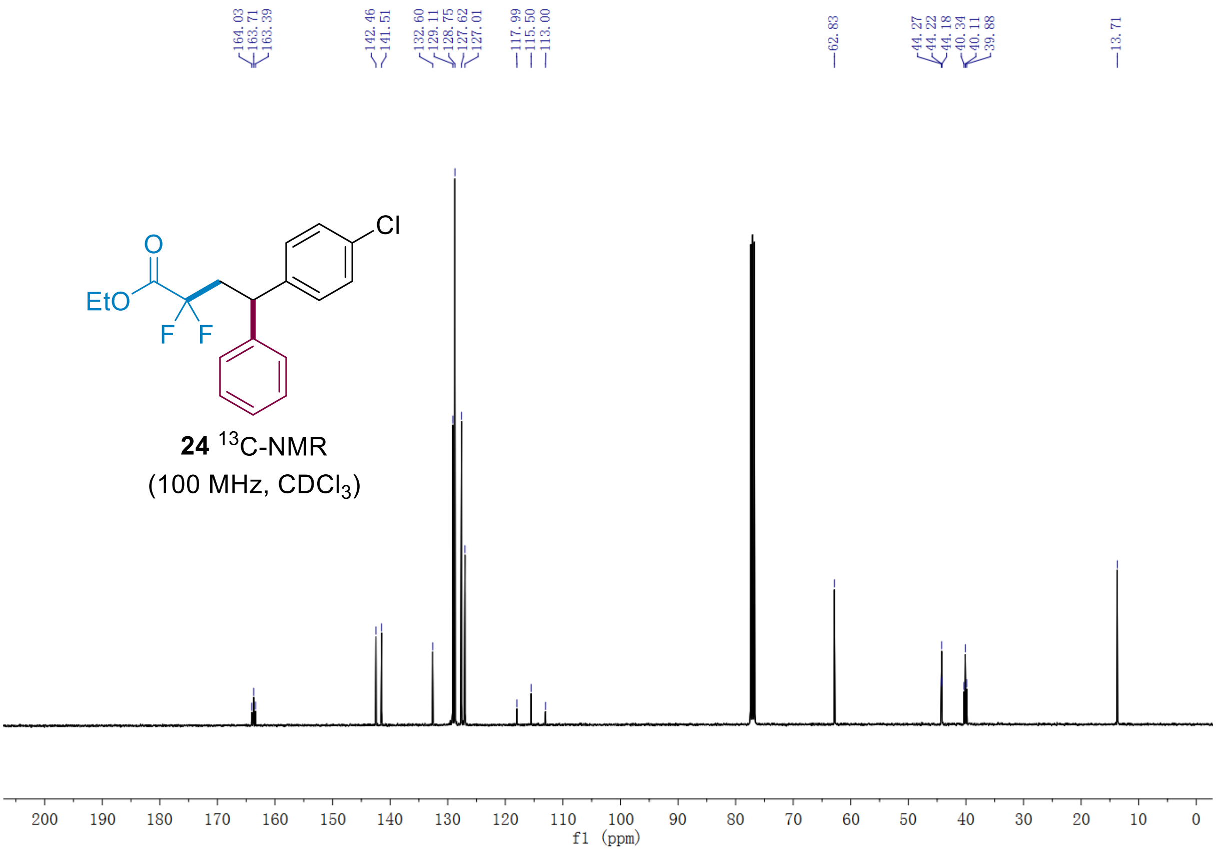
O
EtO
F F
Cl
24 13C-NMR
(100 MHz, CDCl3)
f1 (ppm)

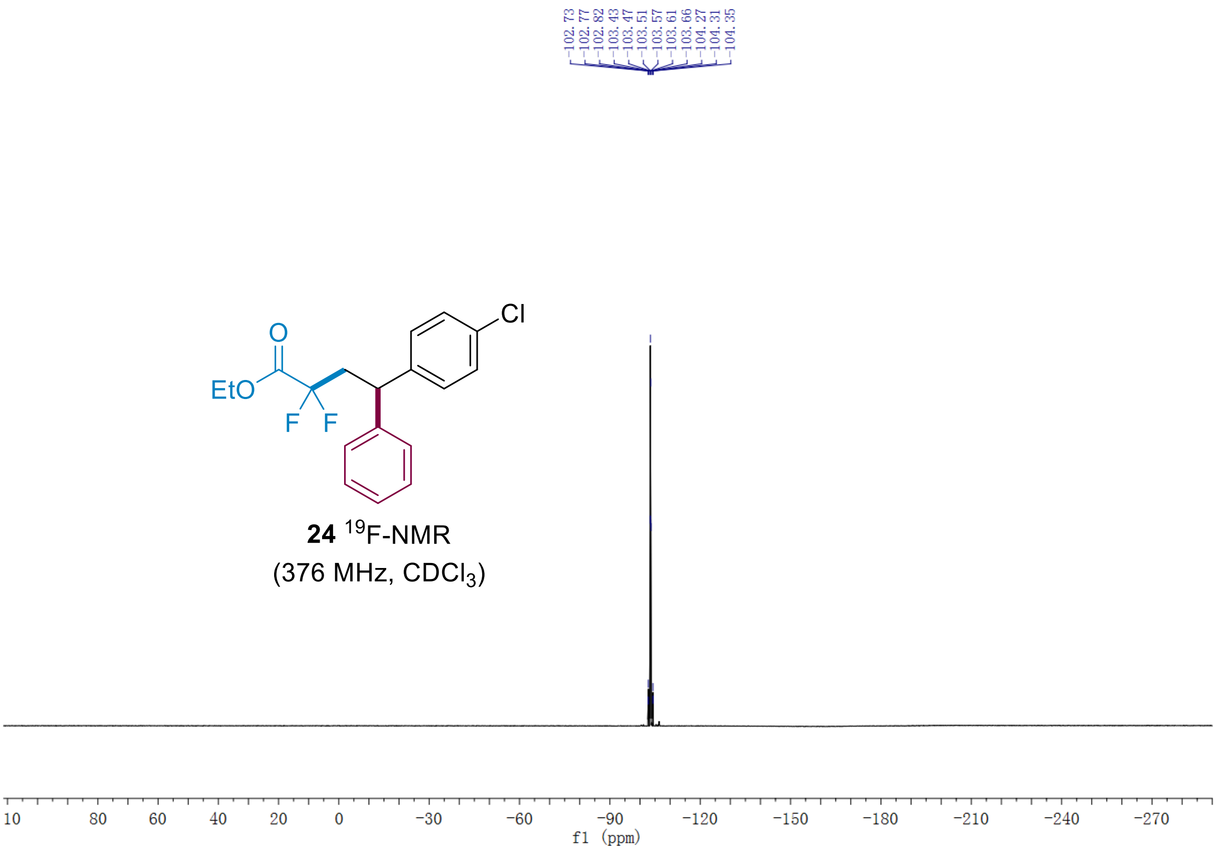

Cl
EtO
O
F F
24 19F-NMR
(376 MHz, CDCl3)
f1 (ppm)

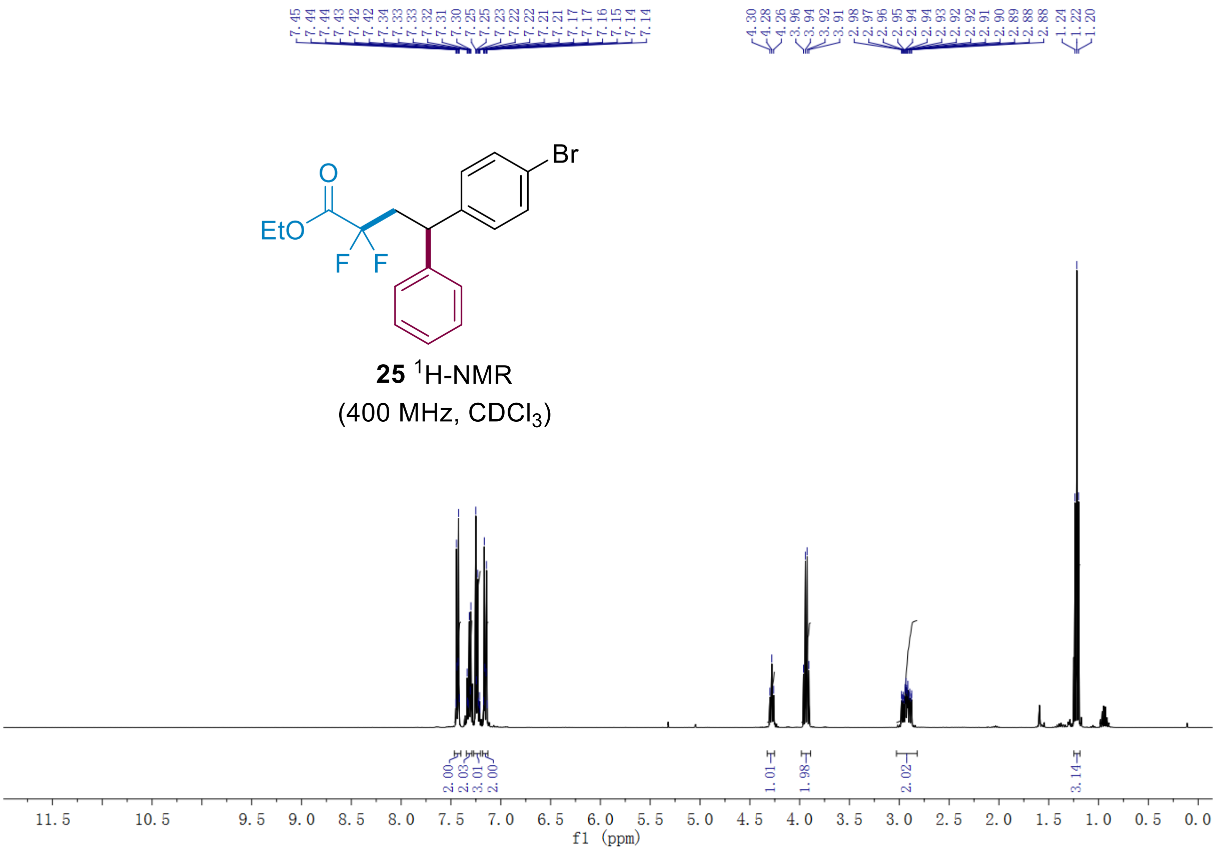

Br
EtO
O
F F
25 1H-NMR
(400 MHz, CDCl3)
f1 (ppm)

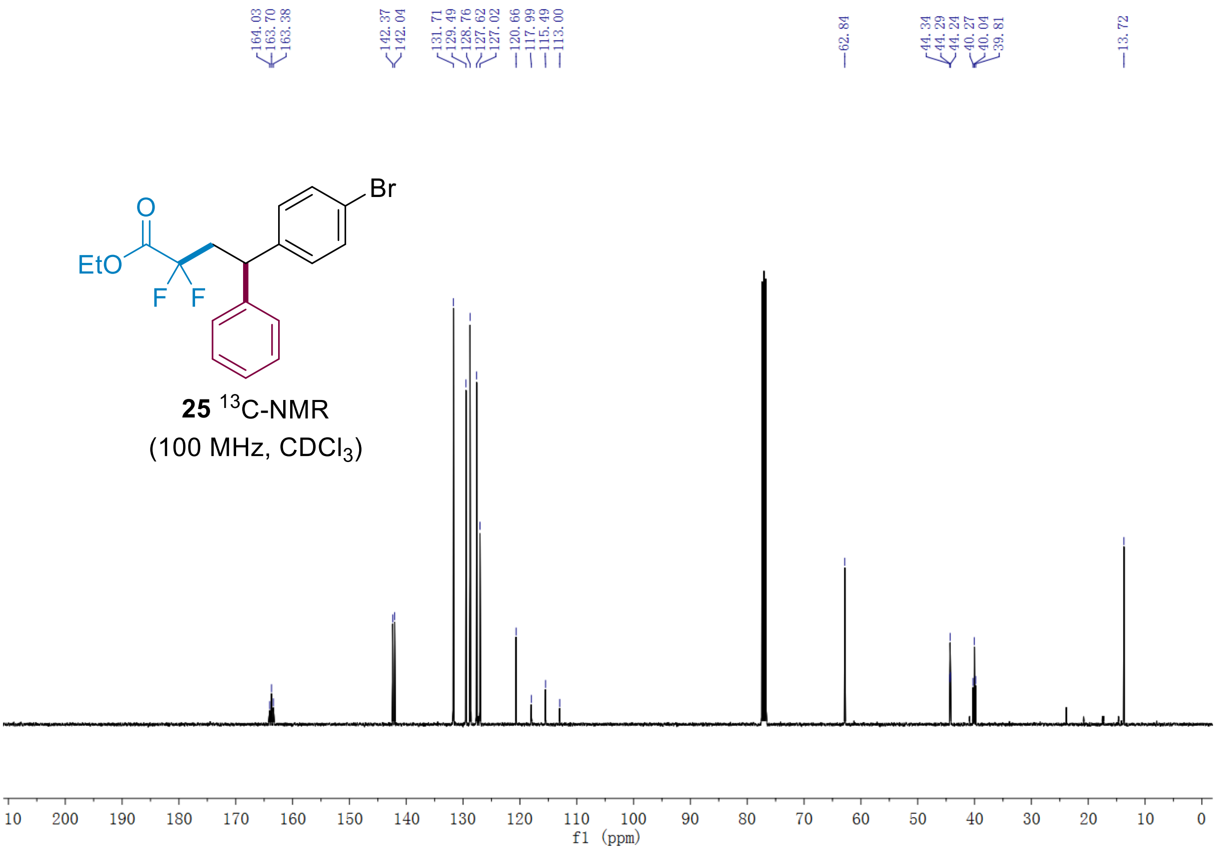
164.03
163.70
163.38
142.37
142.04
131.71
129.49
128.76
127.62
127.02
120.66
117.99
115.49
113.00
62.84
44.34
44.29
44.24
40.27
40.04
39.81
13.72
Br
O
EtO
F F
25 ^{13}C-NMR
(100 MHz, $CDCl_3$)
f1 (ppm)

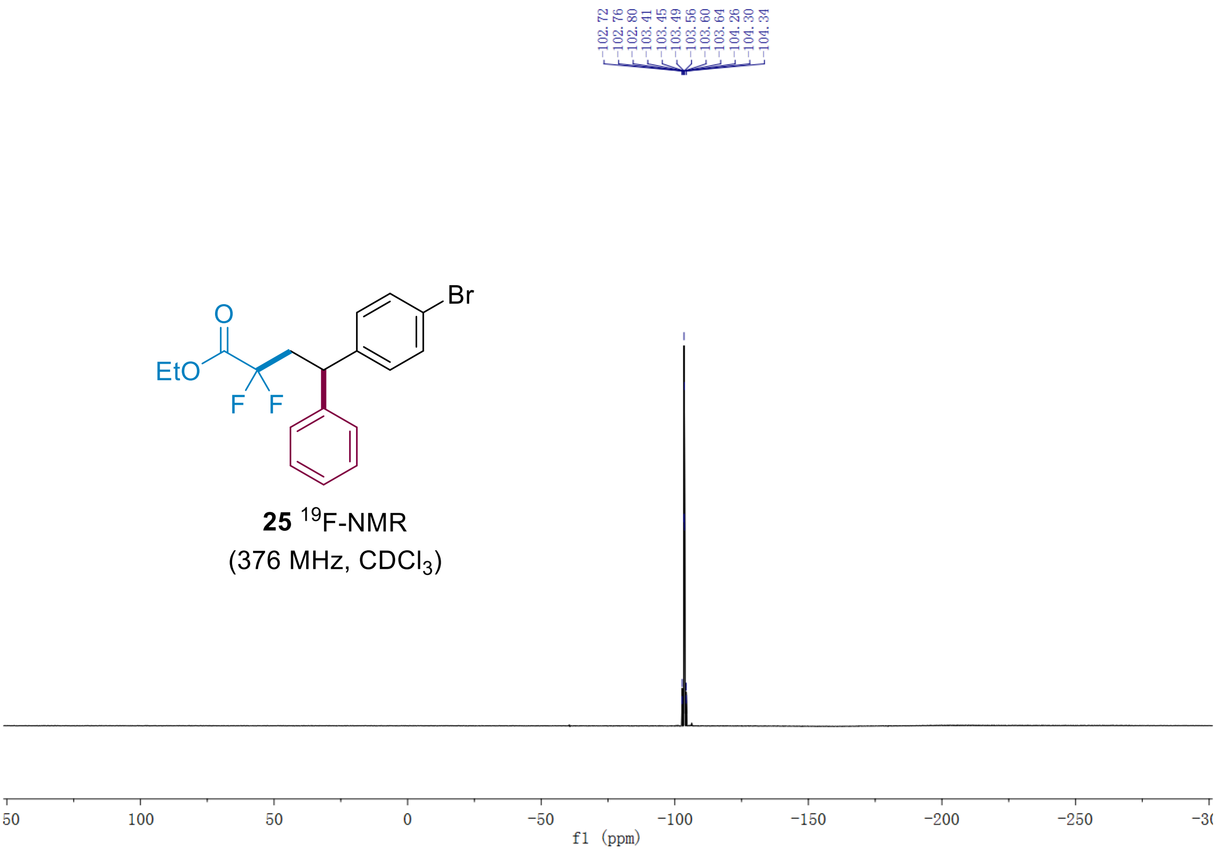
-102.72
-102.76
-102.80
-103.41
-103.45
-103.49
-103.56
-103.60
-103.64
-104.26
-104.30
-104.34
Br
O
EtO
F F
25 ^{19}F-NMR
(376 MHz, $CDCl_3$)
f1 (ppm)

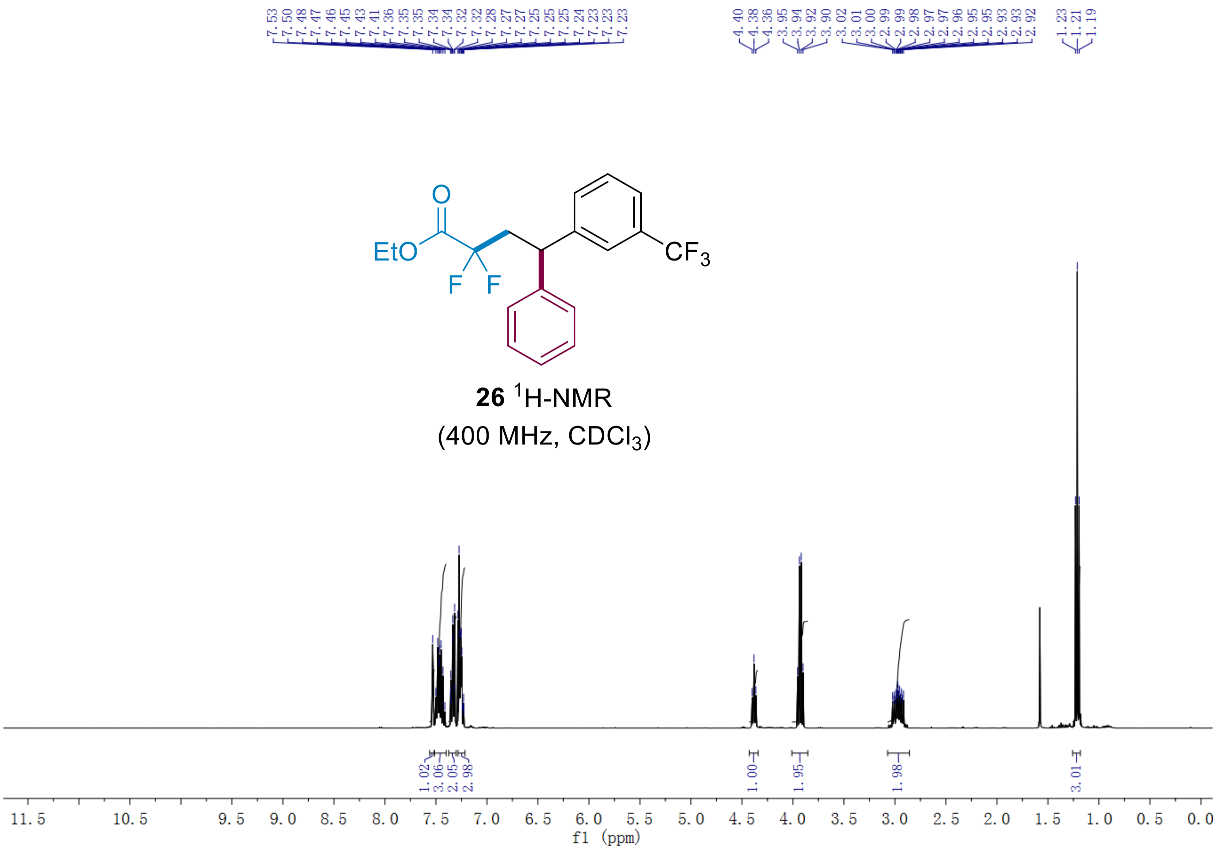
7.53
7.50
7.48
7.47
7.46
7.45
7.43
7.41
7.36
7.35
7.35
7.34
7.34
7.32
7.32
7.28
7.27
7.27
7.25
7.25
7.24
7.23
7.23
7.23
4.40
4.38
4.36
3.95
3.94
3.92
3.90
3.02
3.01
3.00
2.99
2.99
2.98
2.97
2.96
2.95
2.95
2.93
2.93
2.92
1.23
1.21
1.19
EtO
O
F
F
CF3
26 1H-NMR
(400 MHz, CDCl3)
1.02
3.06
2.05
2.98
1.00
1.95
1.98
3.01
f1 (ppm)

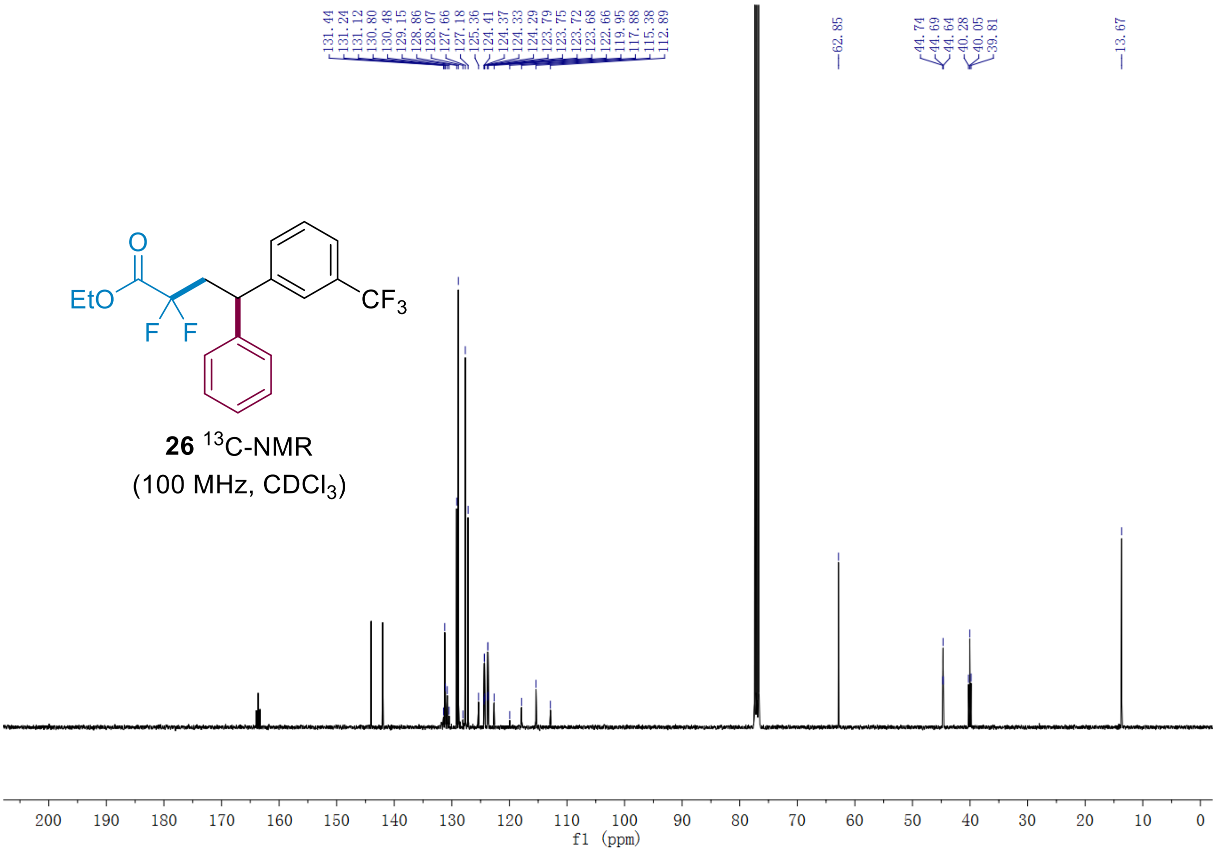
131.44
131.24
131.12
130.80
130.48
129.15
128.86
128.07
127.66
127.18
125.36
124.41
124.37
124.33
124.29
123.79
123.75
123.72
123.68
122.66
119.95
117.88
115.38
112.89
62.85
44.74
44.69
44.64
40.28
40.05
39.81
13.67
EtO
O
F
F
CF3
26 13C-NMR
(100 MHz, CDCl3)
f1 (ppm)

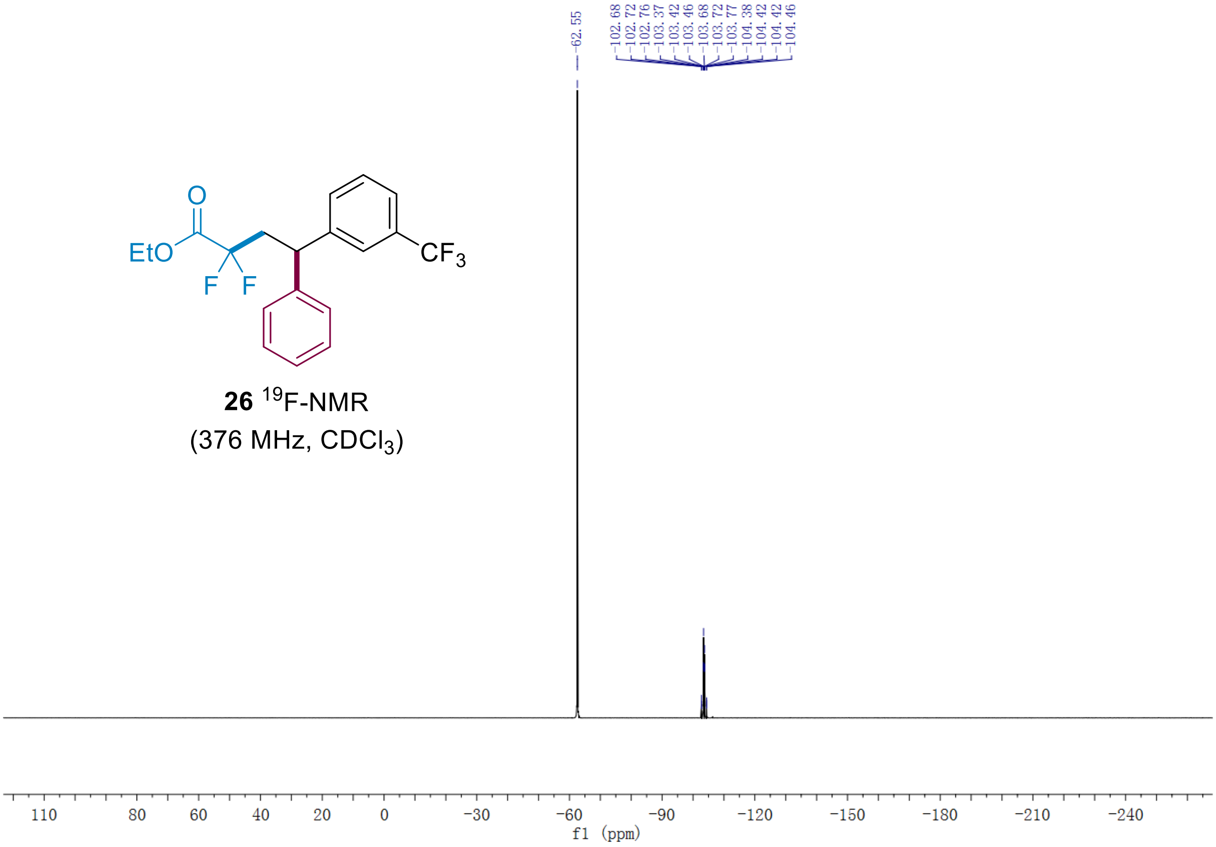
-62.55
-102.68
-102.72
-102.76
-103.37
-103.42
-103.46
-103.68
-103.72
-103.77
-104.38
-104.42
-104.42
-104.46
EtO
O
F F
CF_3
26 ^{19}F-NMR
(376 MHz, $CDCl_3$)
110 80 60 40 20 0 -30 -60 -90 -120 -150 -180 -210 -240
f1 (ppm)

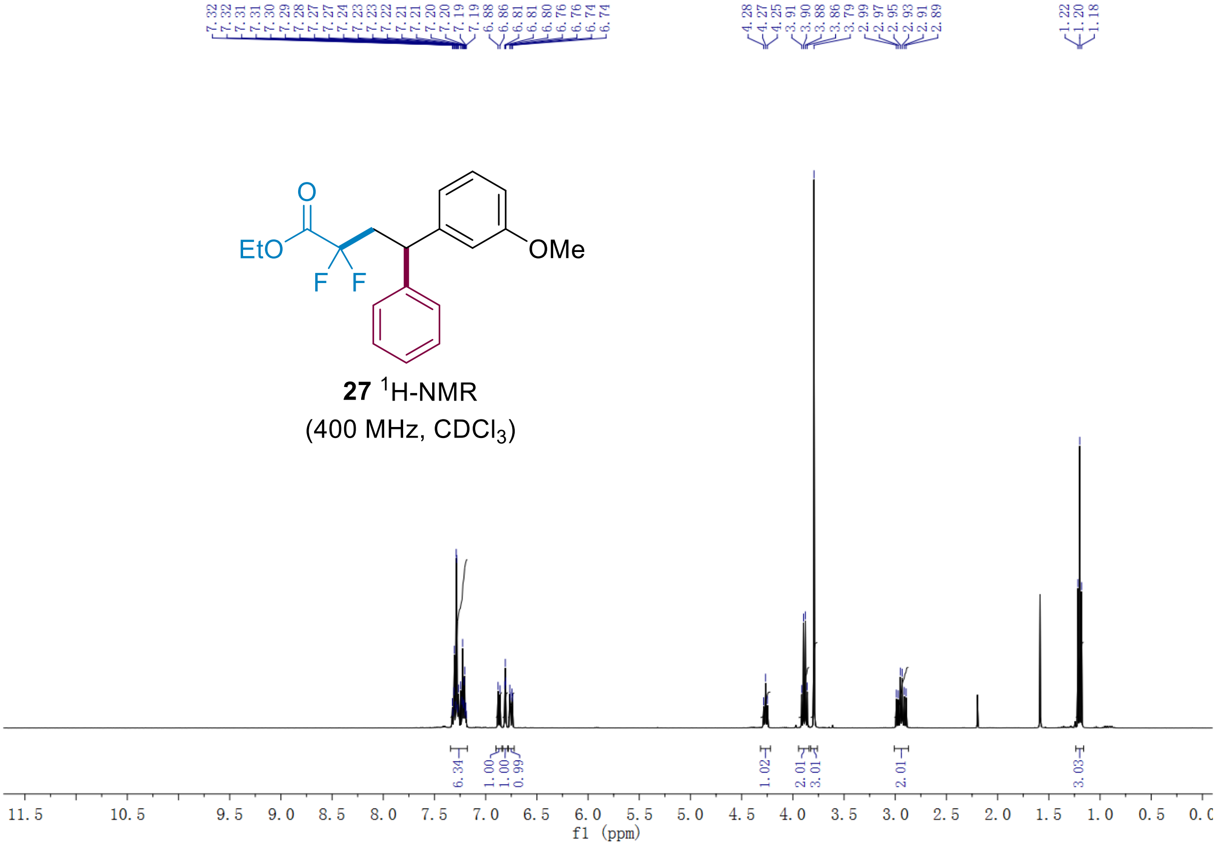
7.32
7.32
7.31
7.30
7.29
7.28
7.27
7.27
7.24
7.23
7.23
7.22
7.21
7.21
7.20
7.19
6.88
6.86
6.81
6.80
6.76
6.74
6.74
4.28
4.27
4.25
3.91
3.90
3.88
3.86
3.79
2.99
2.97
2.95
2.93
2.91
2.89
1.22
1.20
1.18
EtO
O
F F
OMe
27 ^{1}H-NMR
(400 MHz, $CDCl_3$)
6.34
1.00
1.00
0.99
1.02
2.01
3.01
2.01
3.03
11.5 10.5 9.5 9.0 8.5 8.0 7.5 7.0 6.5 6.0 5.5 5.0 4.5 4.0 3.5 3.0 2.5 2.0 1.5 1.0 0.5 0.0
f1 (ppm)

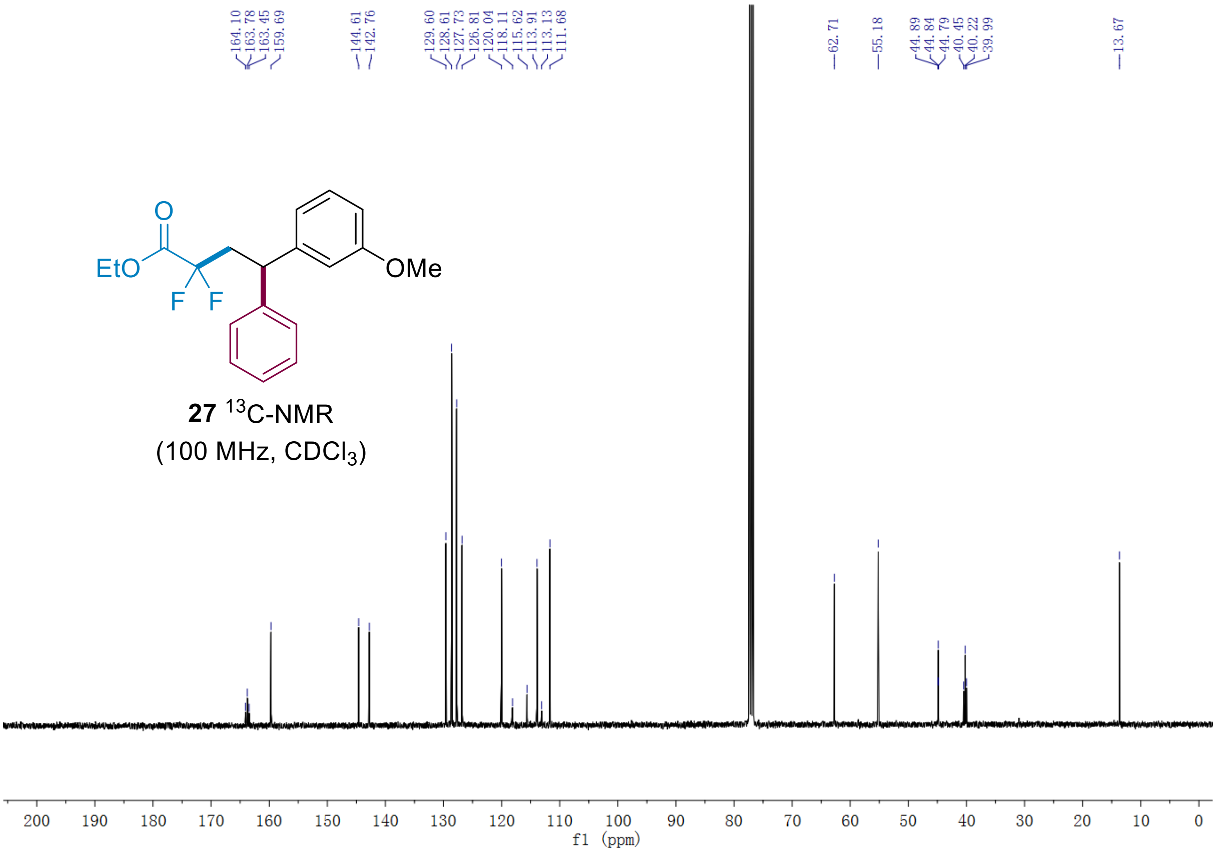
164.10
163.78
163.45
159.69
144.61
142.76
129.60
128.61
127.73
126.81
120.04
118.11
115.62
113.91
113.13
111.68
62.71
55.18
44.89
44.84
44.79
40.45
40.22
39.99
13.67
EtO
O
F F
OMe
27 ^{13}C-NMR
(100 MHz, $CDCl_3$)
f1 (ppm)

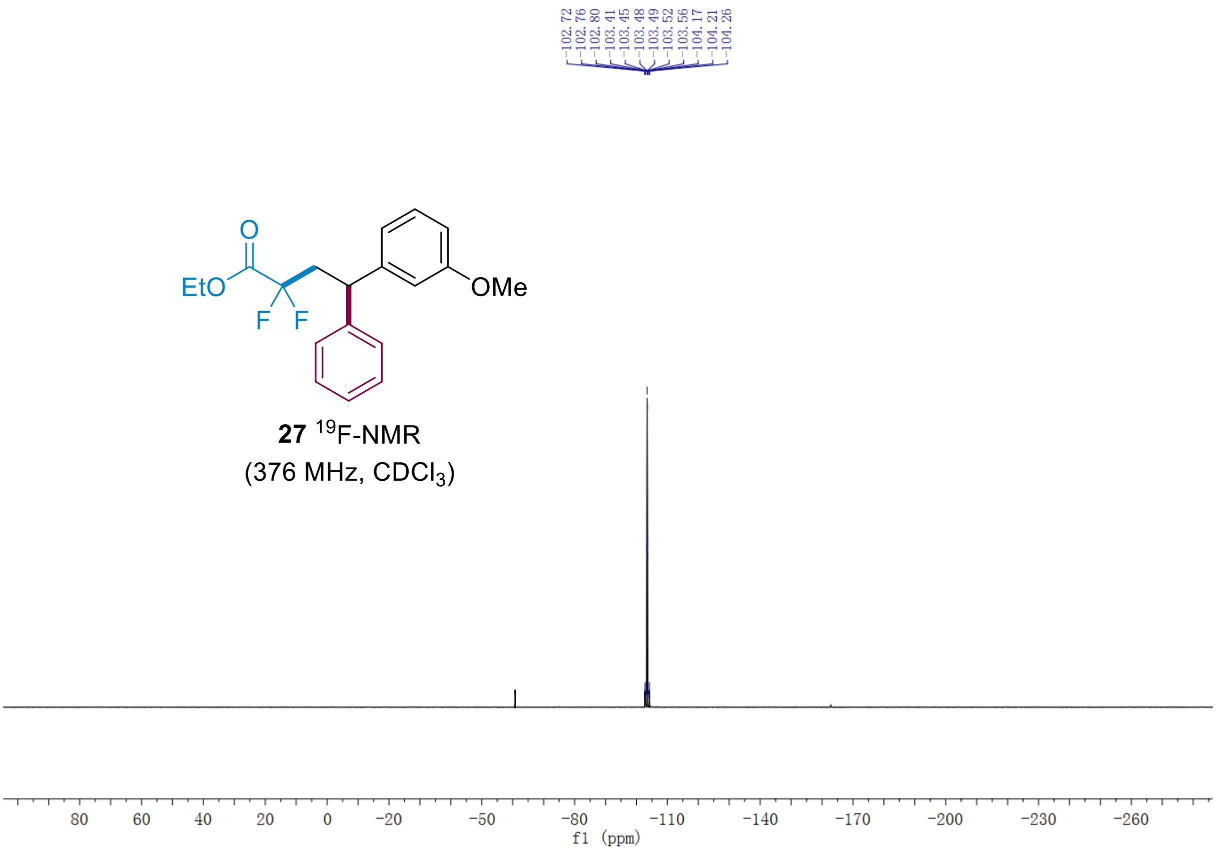
-102.72
-102.76
-102.80
-103.41
-103.45
-103.48
-103.49
-103.52
-103.56
-104.17
-104.21
-104.26
EtO
O
F F
OMe
27 ^{19}F-NMR
(376 MHz, $CDCl_3$)
f1 (ppm)

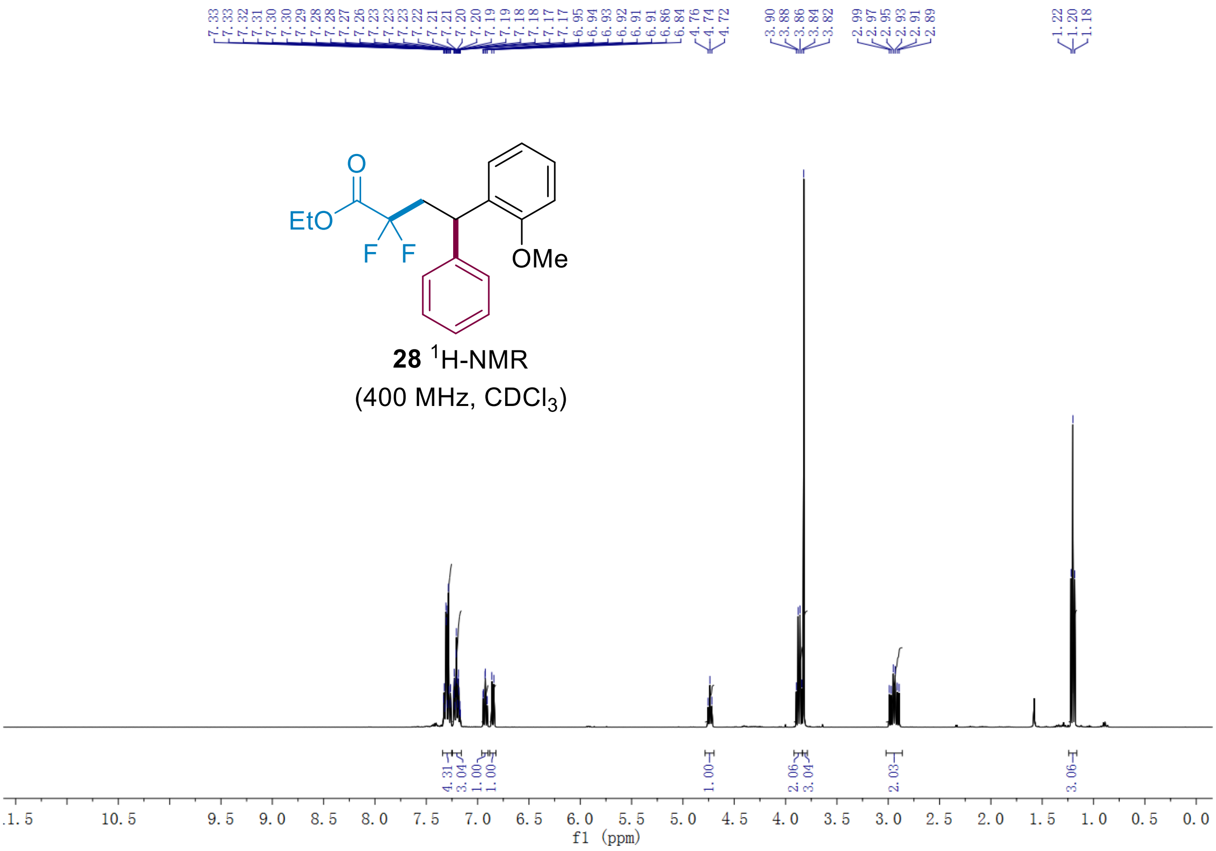
EtO
O
F F
OMe
28 ¹H-NMR
(400 MHz, CDCl3)
f1 (ppm)

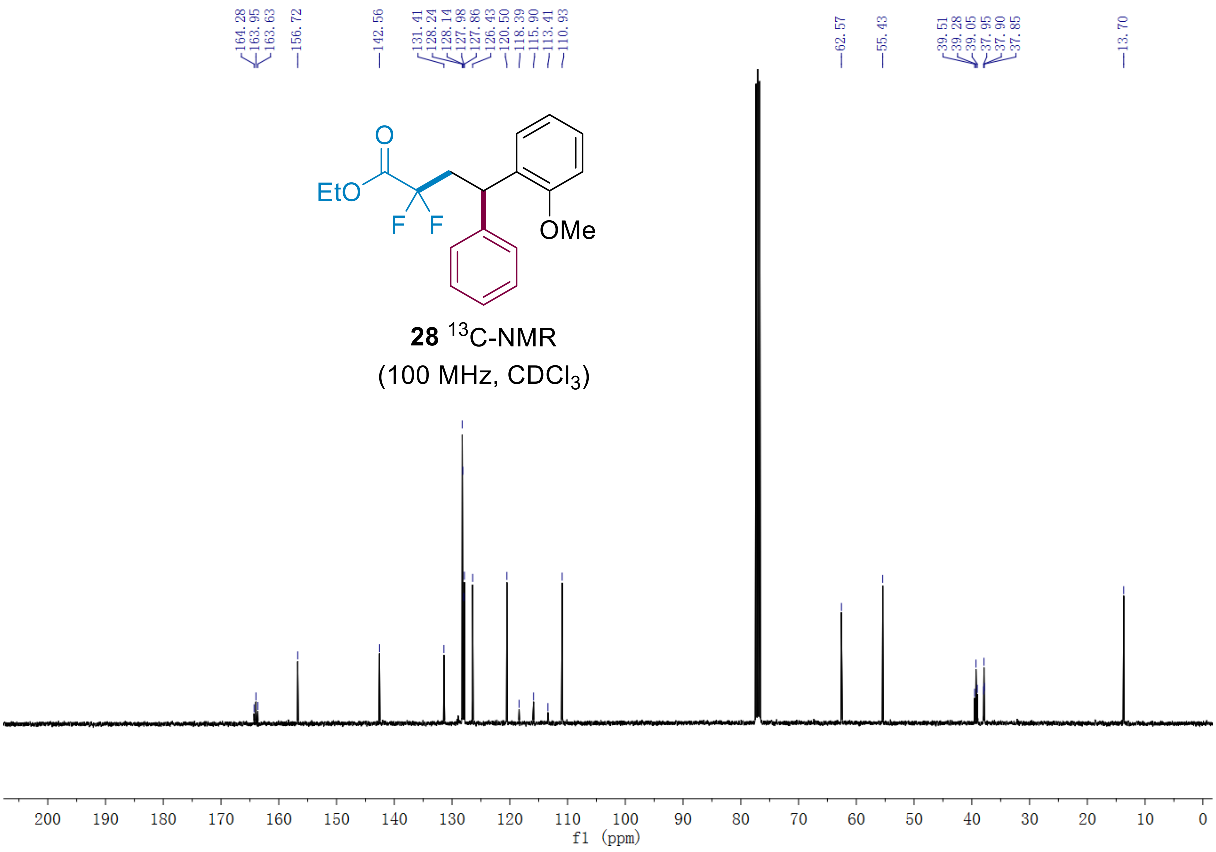
EtO
O
F F
OMe
28 ¹³C-NMR
(100 MHz, CDCl3)
f1 (ppm)

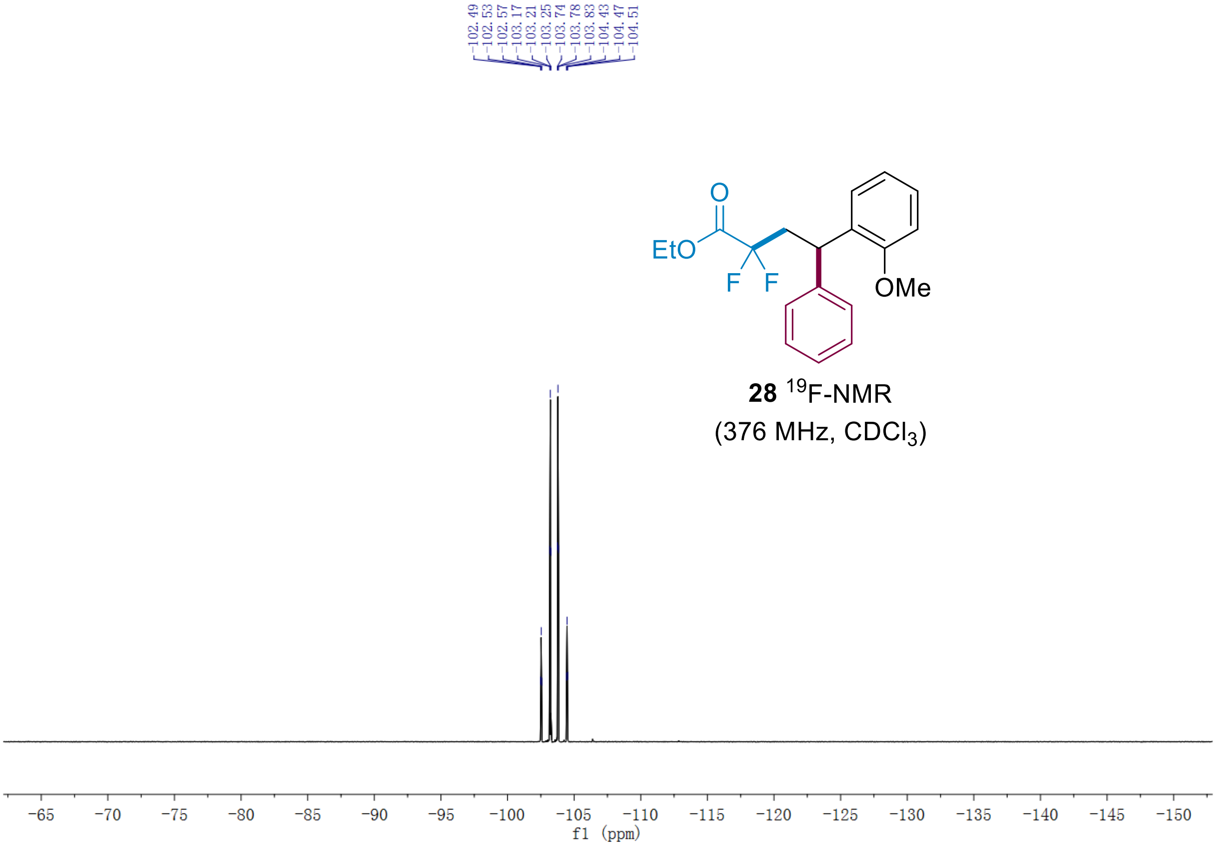
EtO
O
F F
OMe
28 19F-NMR
(376 MHz, CDCl3)
f1 (ppm)

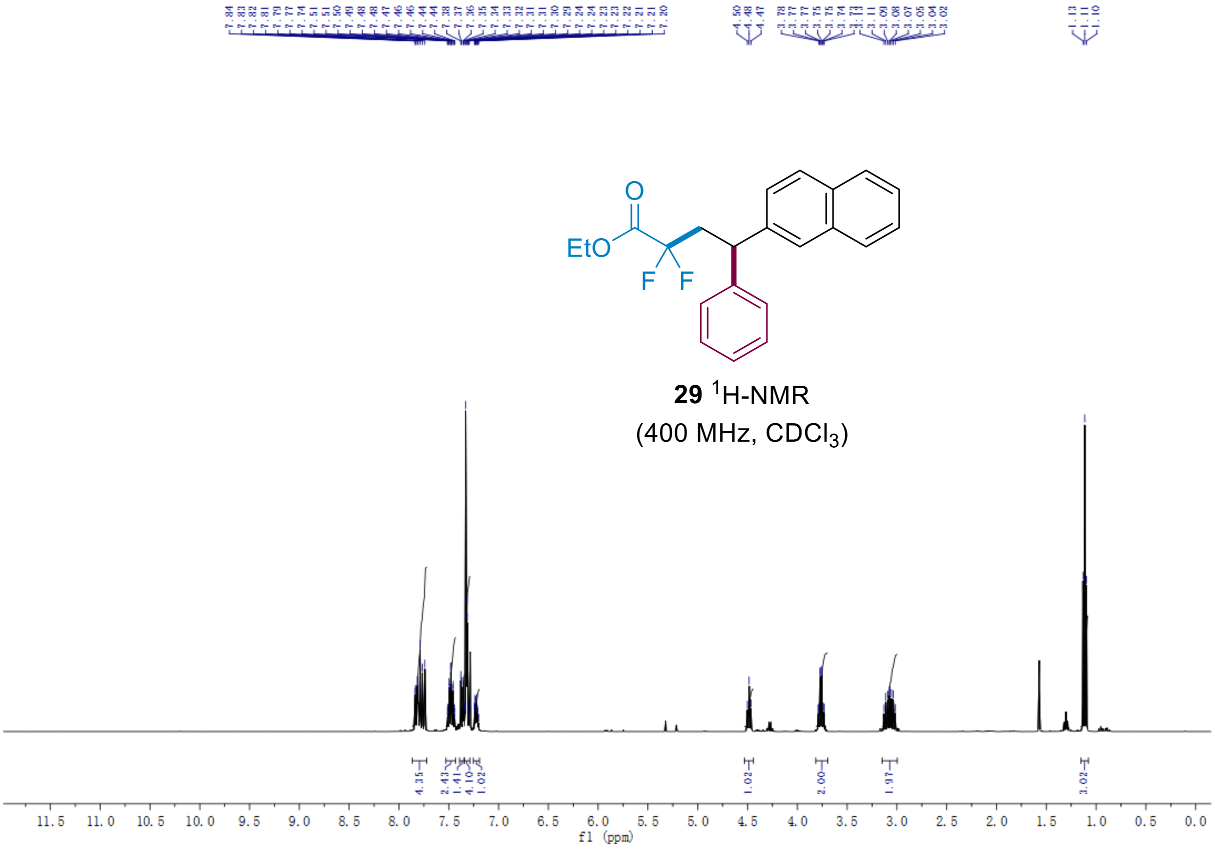
EtO
O
F F
29 1H-NMR
(400 MHz, CDCl3)
f1 (ppm)

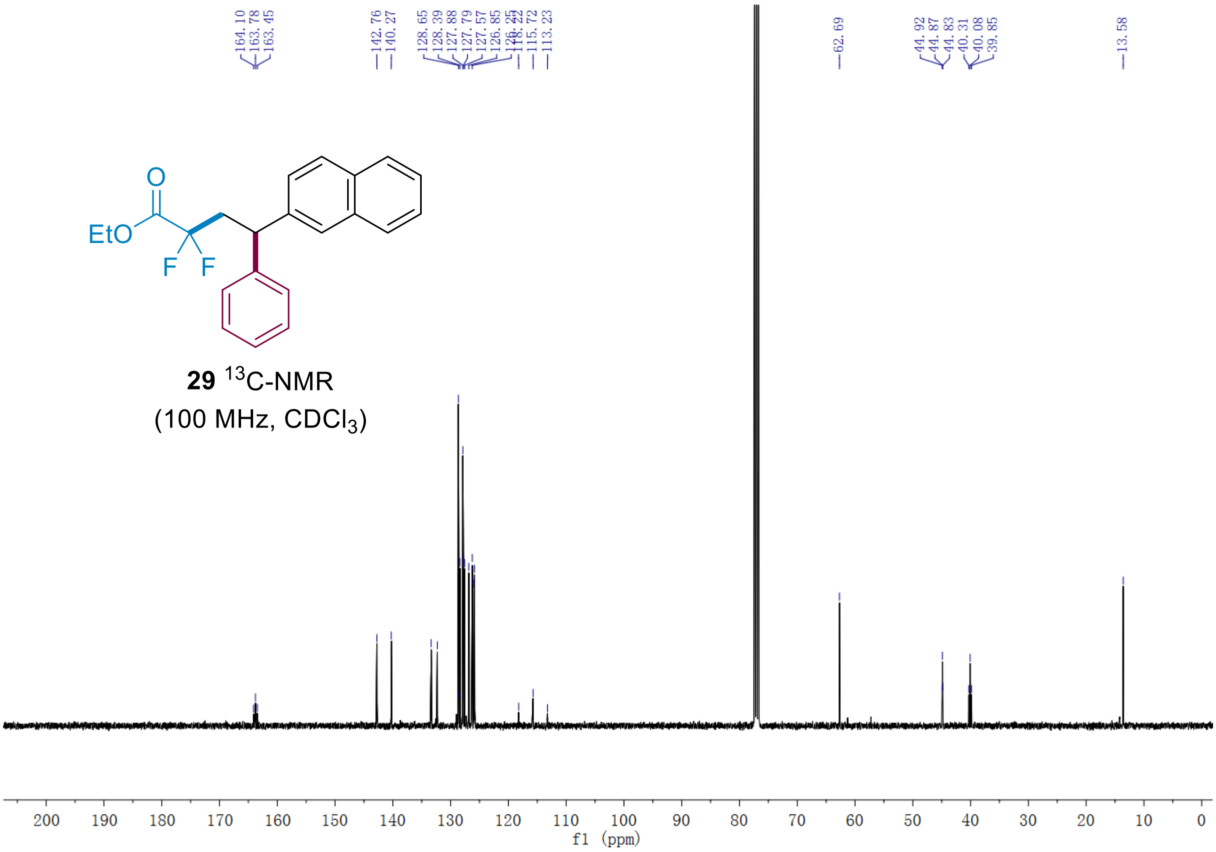
164.10
163.78
163.45
142.76
140.27
128.65
128.39
127.88
127.79
127.57
126.85
115.72
113.23
62.69
44.92
44.87
44.83
40.31
40.08
39.85
13.58
EtO
O
F F
29 ^{13}C-NMR
(100 MHz, $CDCl_3$)
f1 (ppm)

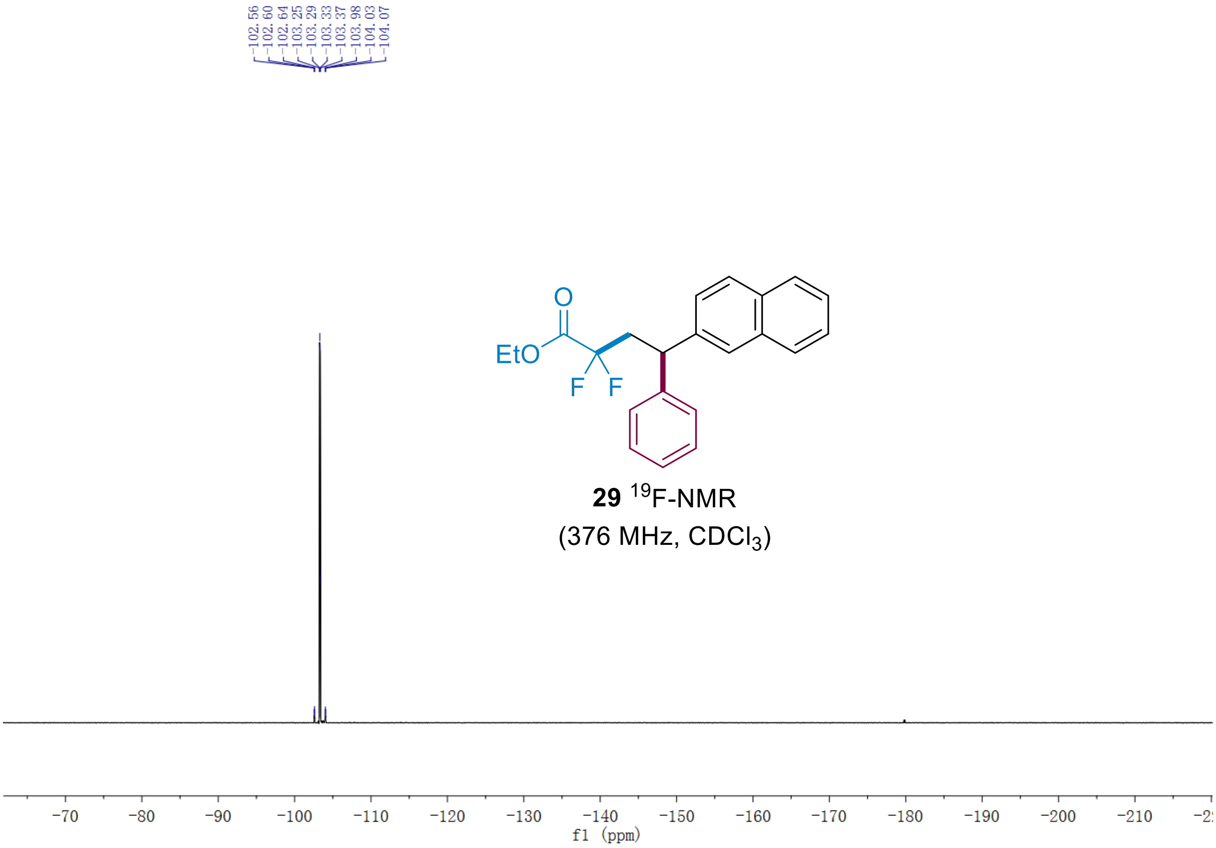
-102.56
-102.60
-102.64
-103.25
-103.29
-103.33
-103.37
-103.98
-104.03
-104.07
EtO
O
F F
29 ^{19}F-NMR
(376 MHz, $CDCl_3$)
f1 (ppm)

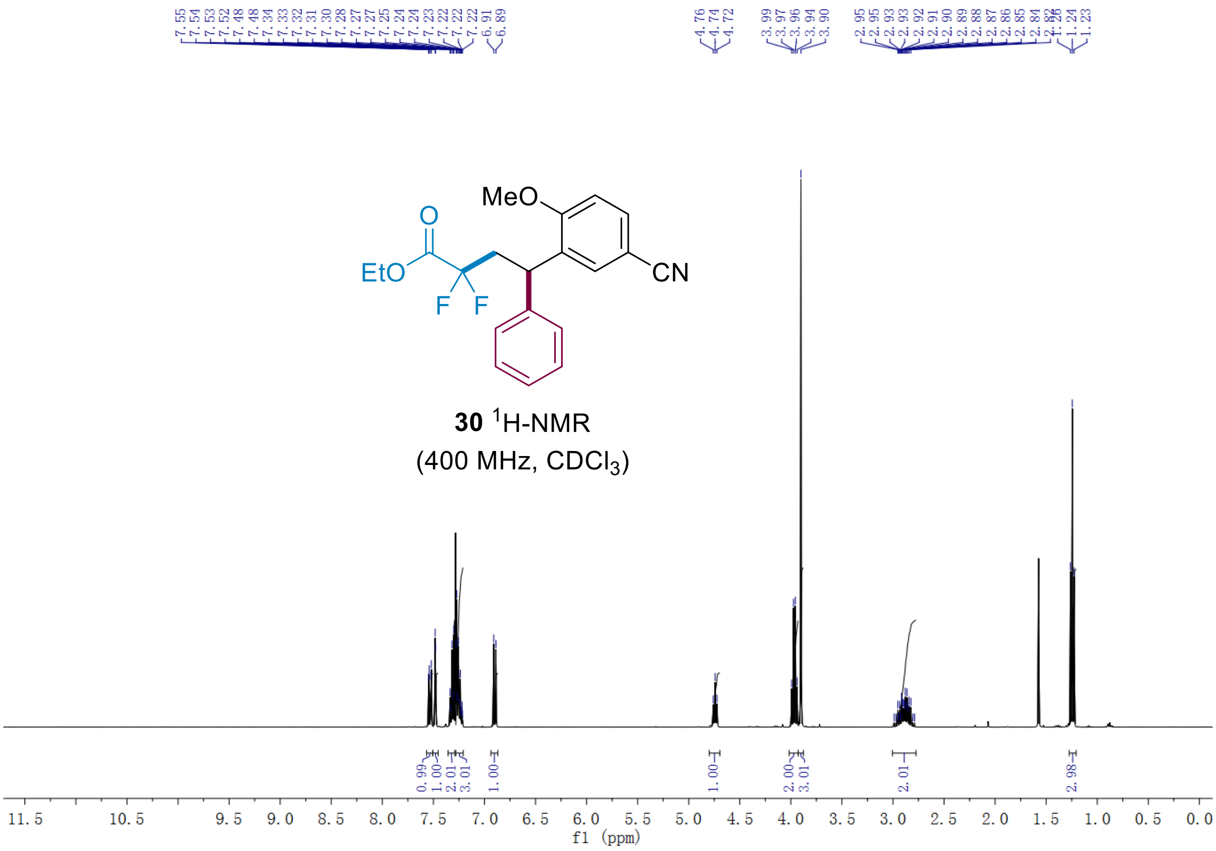
MeO
O
EtO
F F
CN
30 1H-NMR
(400 MHz, CDCl3)
f1 (ppm)

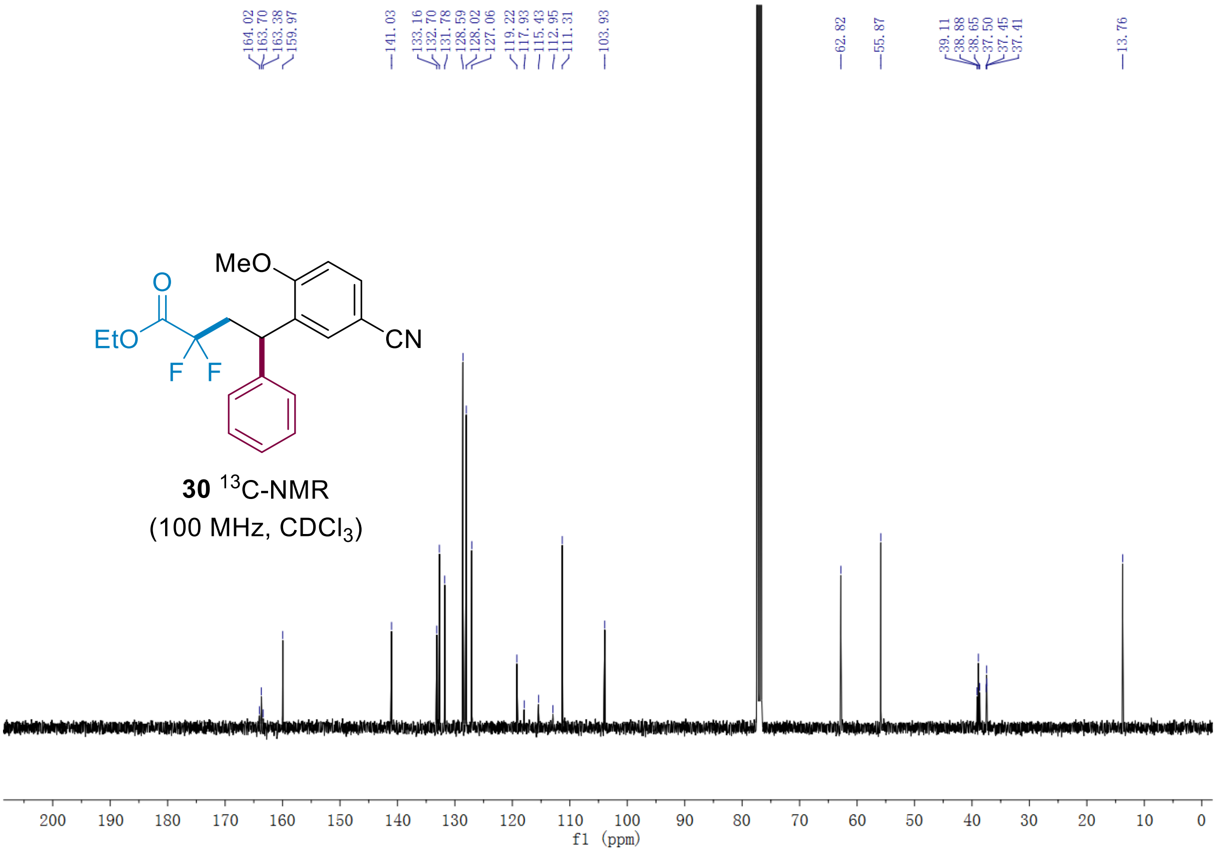
MeO
O
EtO
F F
CN
30 13C-NMR
(100 MHz, CDCl3)
f1 (ppm)

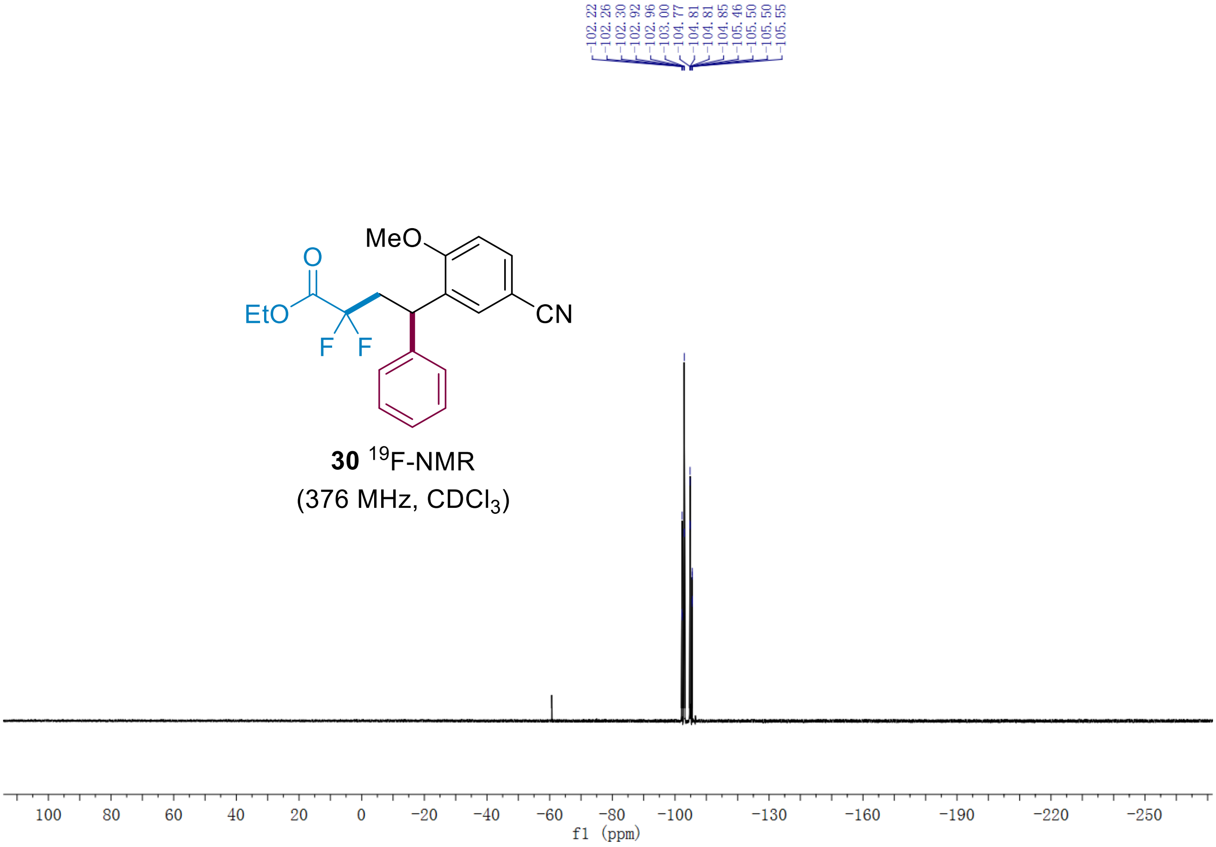
MeO
EtO
O
F F
CN
30 19F-NMR
(376 MHz, CDCl3)
f1 (ppm)

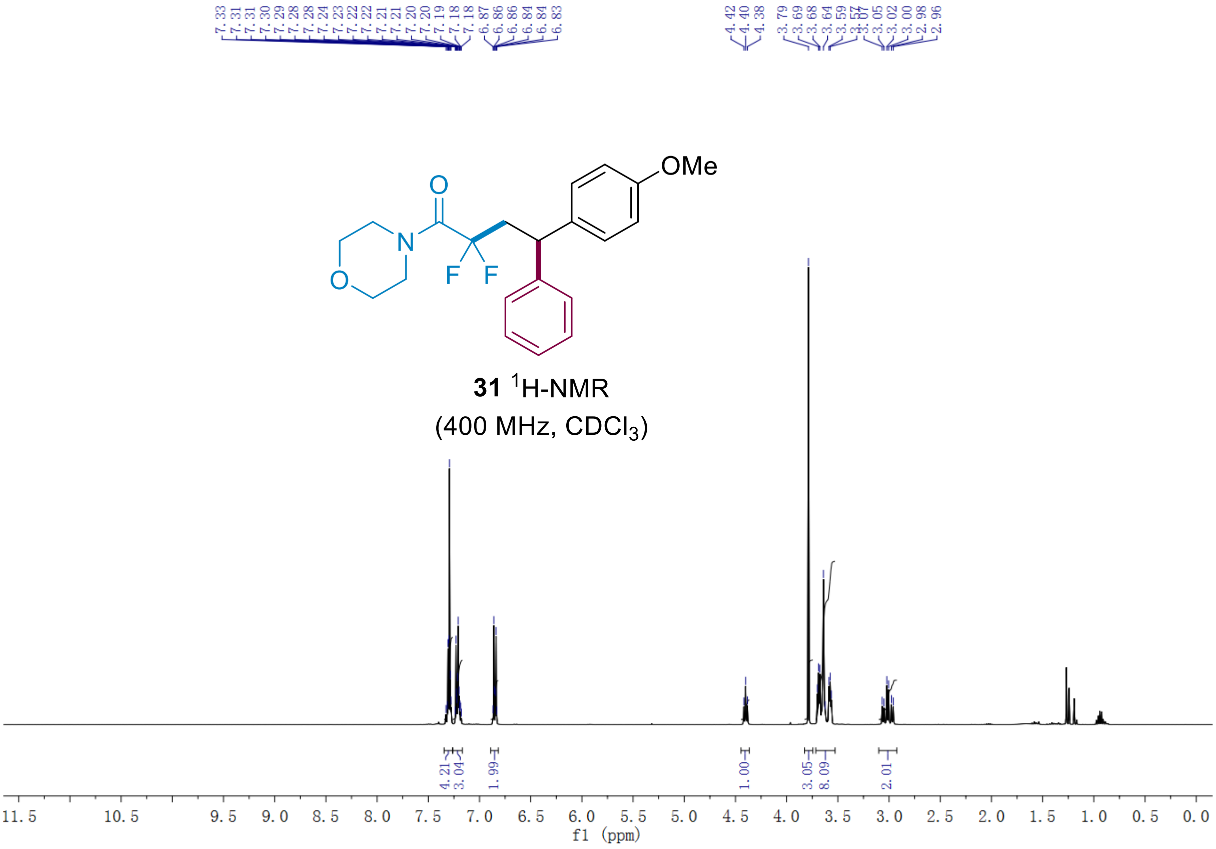
OMe
O
N
O
F F
31 1H-NMR
(400 MHz, CDCl3)
f1 (ppm)

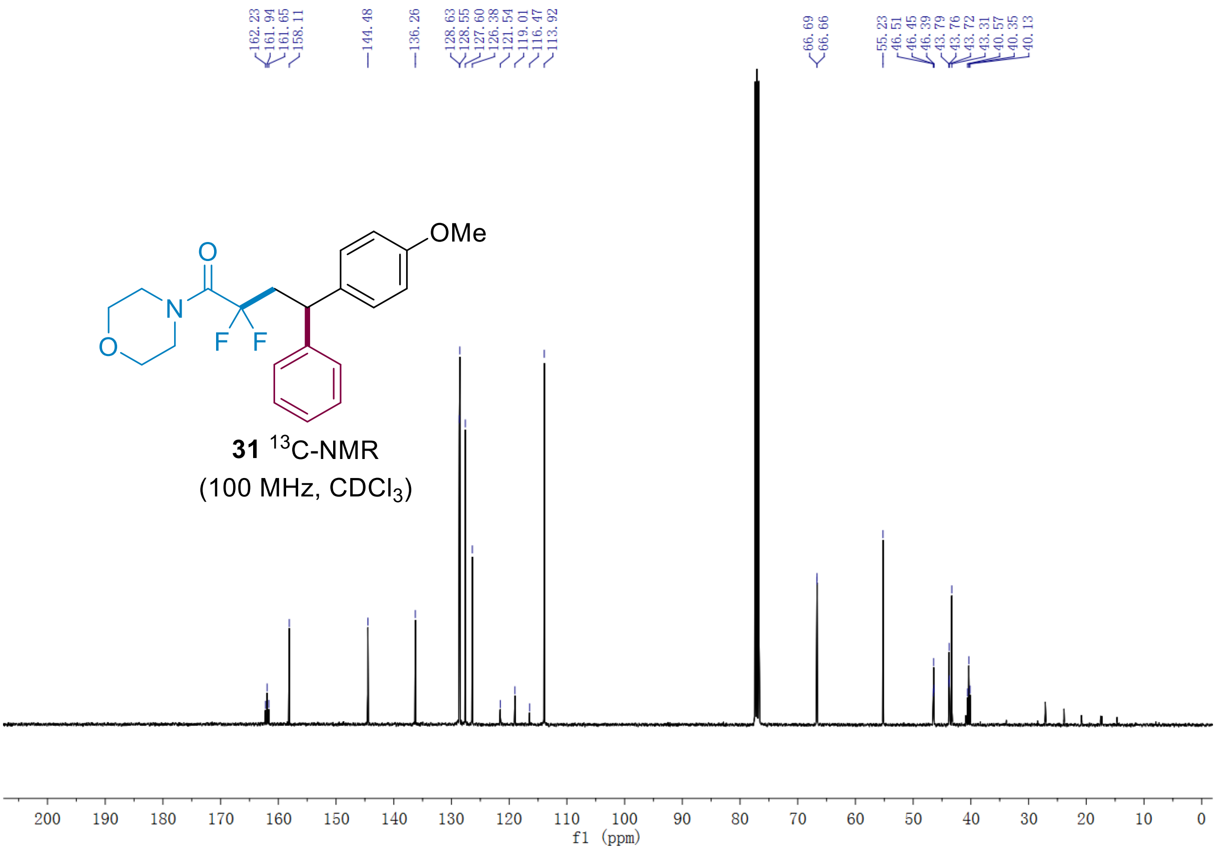

31 ^{13}C-NMR (100 MHz, $CDCl_3$)

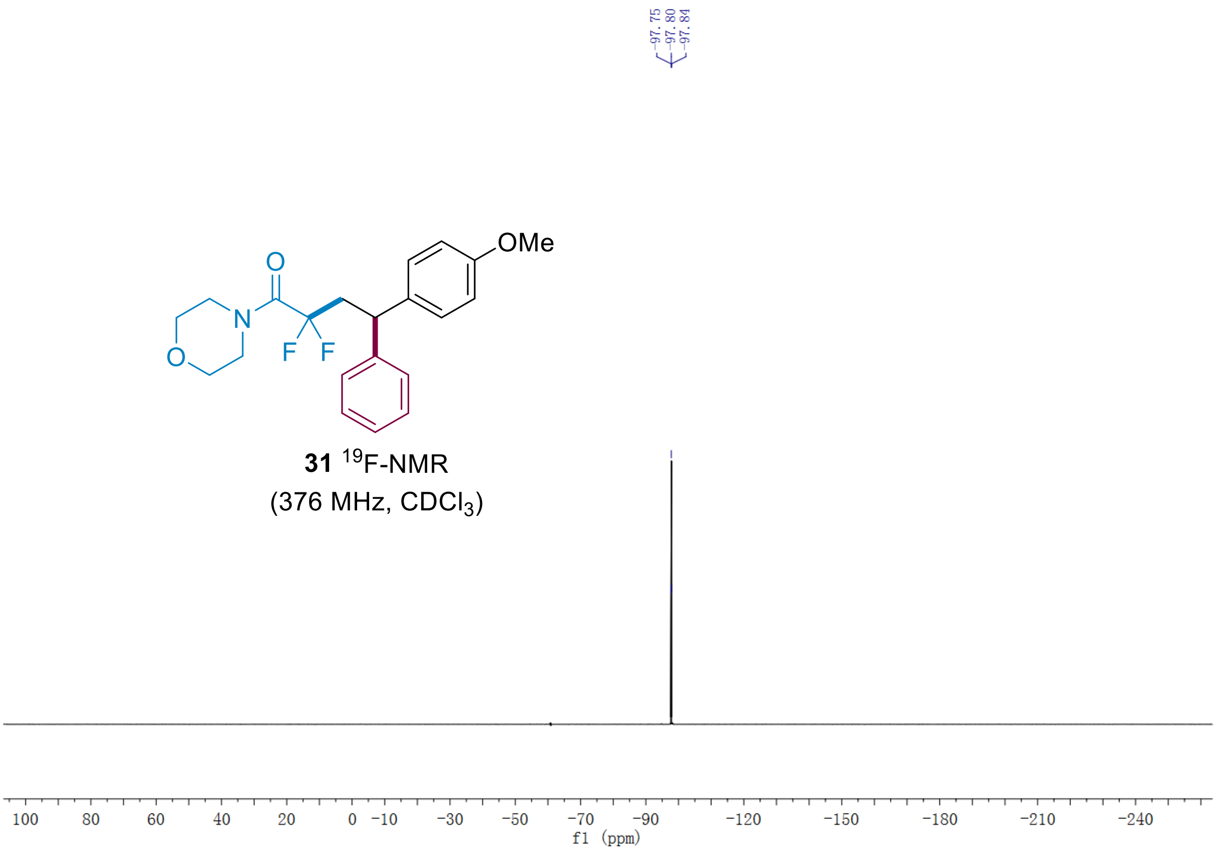

31 ^{19}F-NMR (376 MHz, $CDCl_3$)

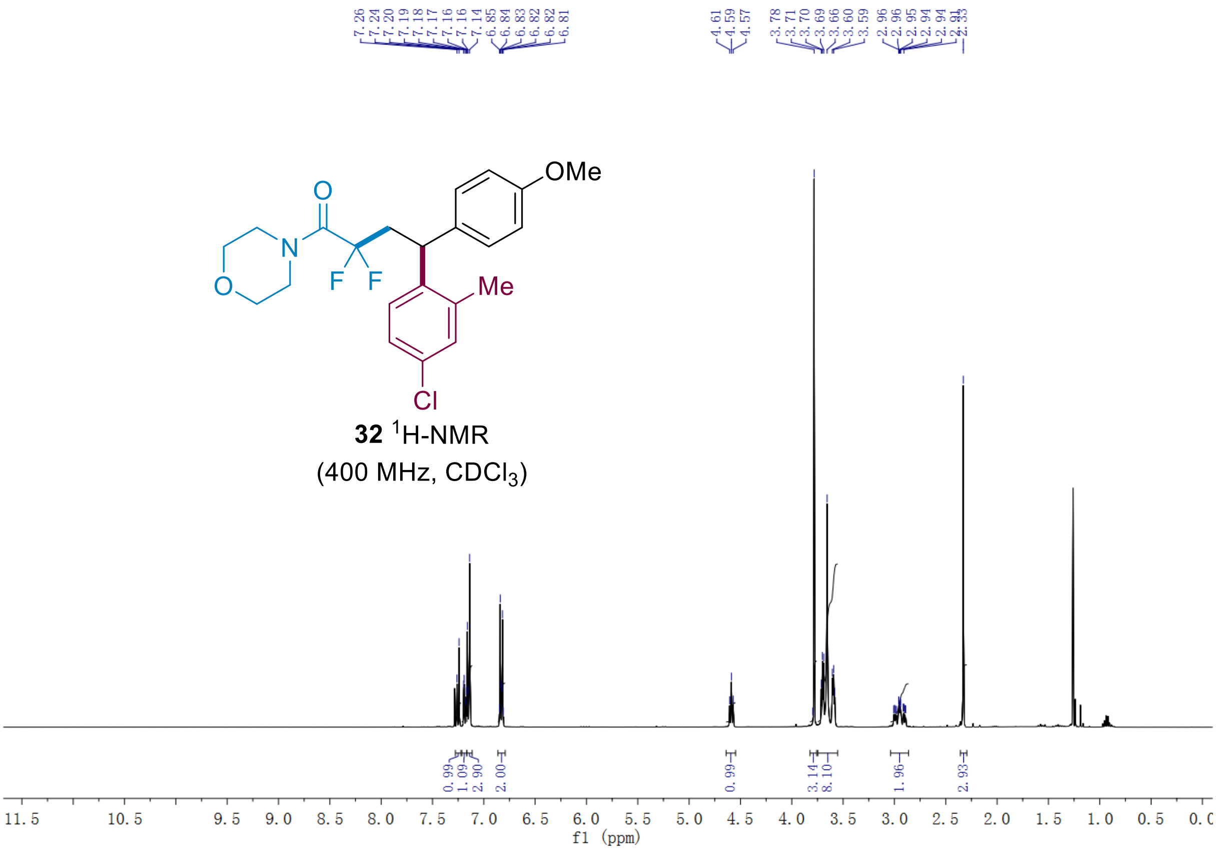
7.26 7.24 7.20 7.19 7.18 7.17 7.16 7.16 7.14 6.85 6.84 6.83 6.82 6.82 6.81
4.61 4.59 4.57 3.78 3.71 3.70 3.69 3.66 3.60 3.59 2.96 2.96 2.95 2.94 2.94 2.91 2.33
OMe
O
N
O
F F
Me
Cl
32 1H-NMR
(400 MHz, CDCl3)
0.99 1.09 2.90 2.00 0.99 3.14 8.10 1.96 2.93
11.5 10.5 9.5 9.0 8.5 8.0 7.5 7.0 6.5 6.0 5.5 5.0 4.5 4.0 3.5 3.0 2.5 2.0 1.5 1.0 0.5 0.0
f1 (ppm)

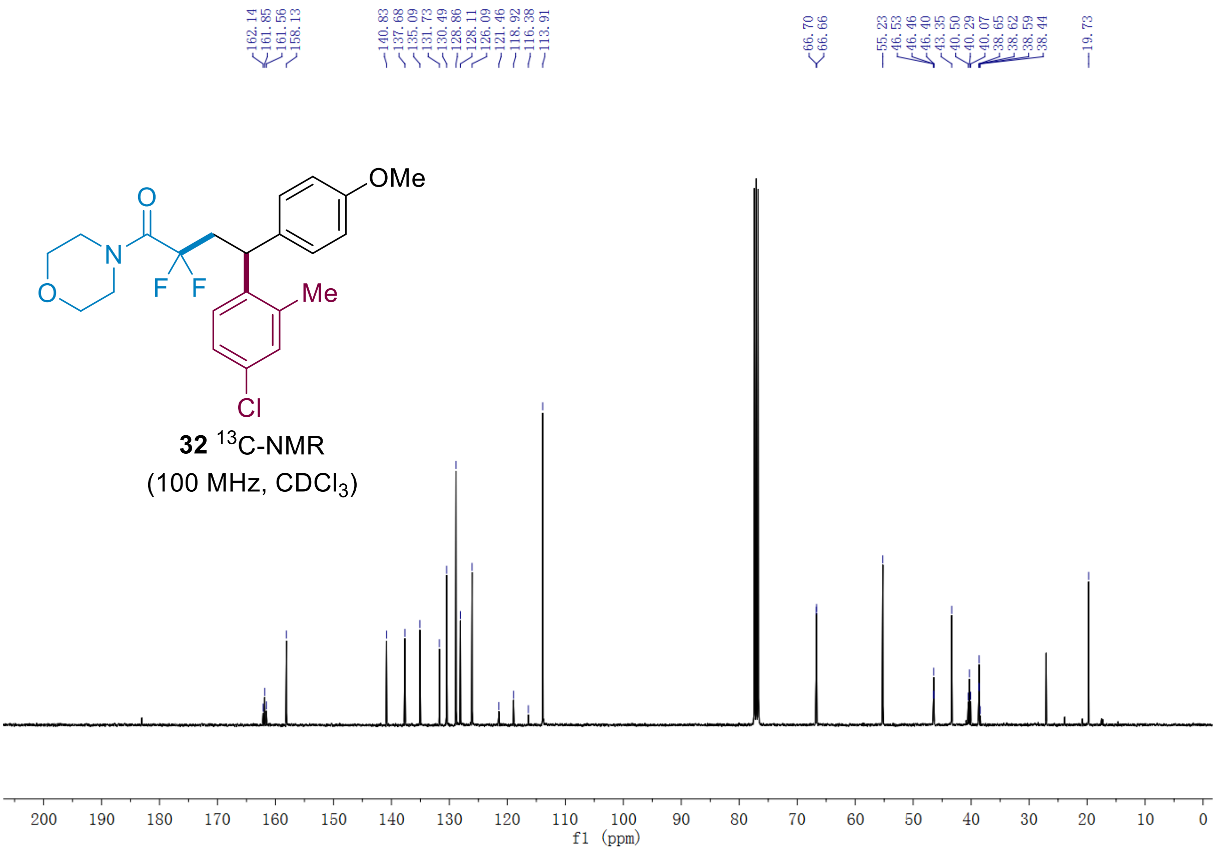
162.14 161.85 161.56 158.13
140.83 137.68 135.09 131.73 130.49 128.86 128.11 126.09 121.46 118.92 116.38 113.91
66.70 66.66
55.23 46.53 46.46 46.40 43.35 40.50 40.29 40.07 38.65 38.62 38.59 38.44
19.73
OMe
O
N
O
F F
Me
Cl
32 13C-NMR
(100 MHz, CDCl3)
200 190 180 170 160 150 140 130 120 110 100 90 80 70 60 50 40 30 20 10 0
f1 (ppm)

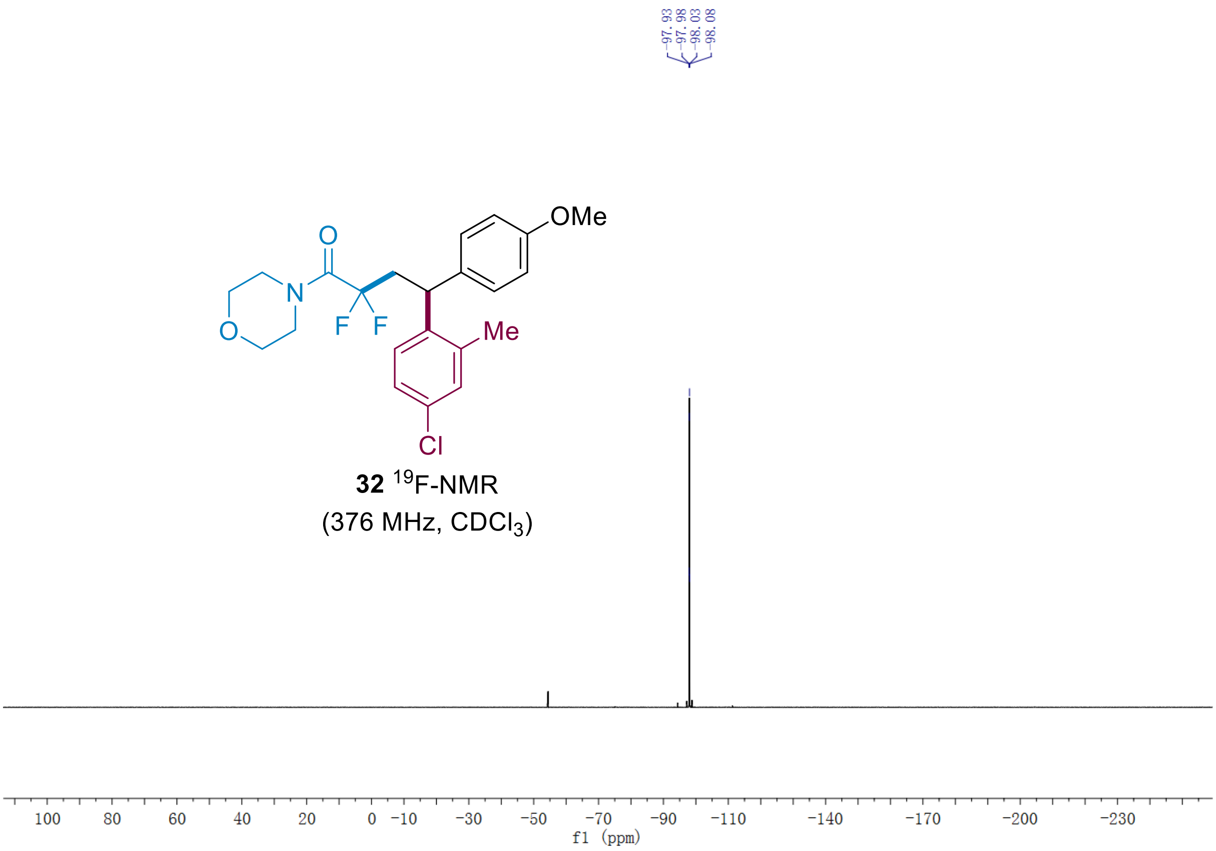

OMe
O
N
O
F F
Me
Cl
32 19F-NMR
(376 MHz, CDCl3)
-97.93
-97.98
-98.03
-98.08
f1 (ppm)

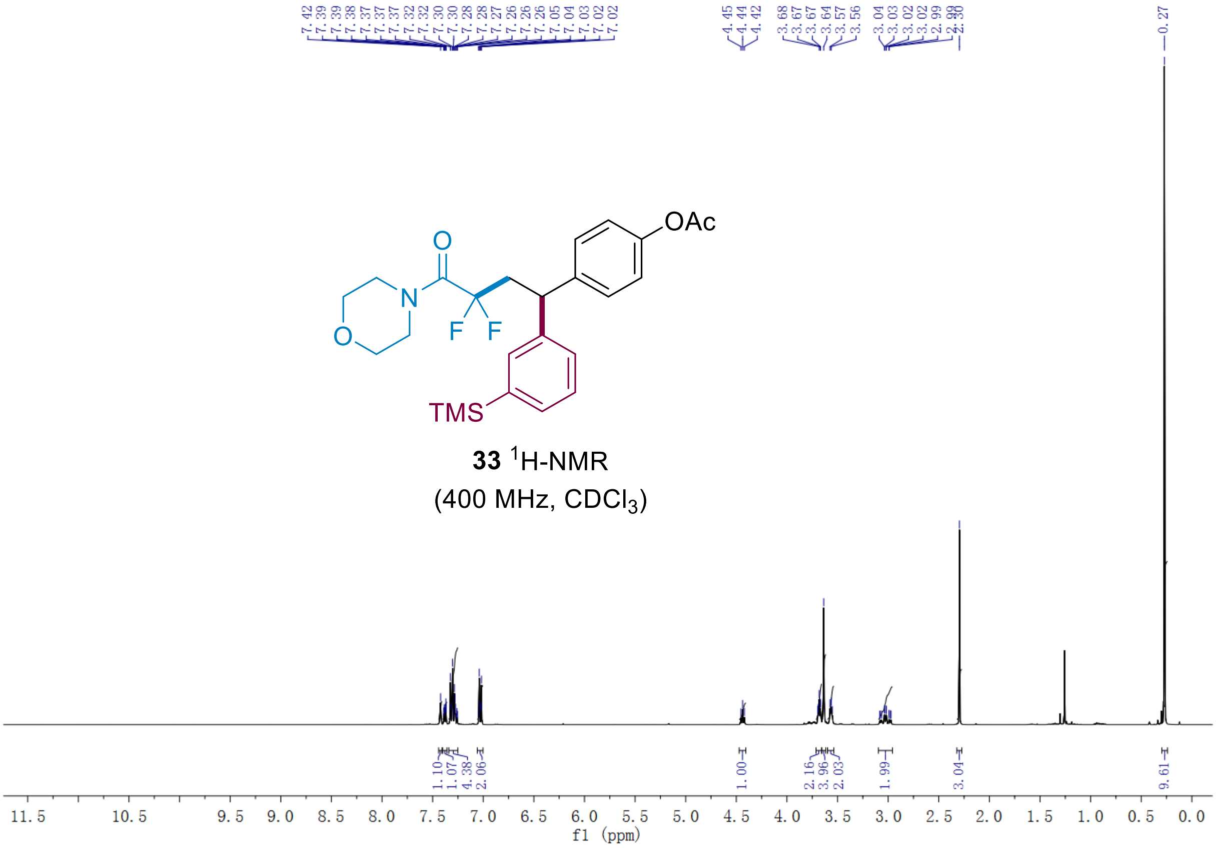

OAc
O
N
O
F F
TMS
33 1H-NMR
(400 MHz, CDCl3)
0.27
f1 (ppm)

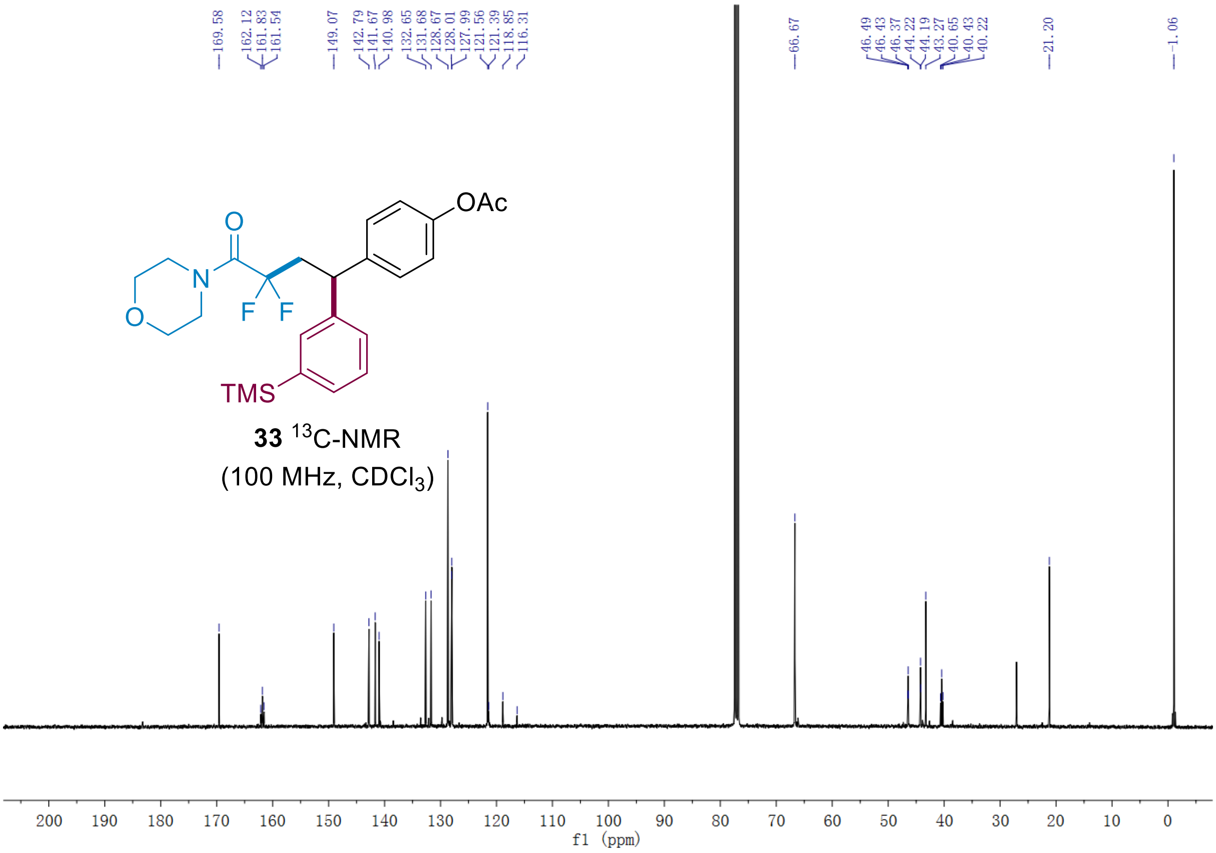
169.58
162.12
161.83
161.54
149.07
142.79
141.67
140.98
132.65
131.68
128.67
128.01
127.99
121.56
121.39
118.85
116.31
66.67
46.49
46.43
46.37
44.22
44.19
43.27
40.65
40.43
40.22
21.20
-1.06
OAc
O
N
O
F F
TMS
33 13C-NMR
(100 MHz, CDCl3)
f1 (ppm)

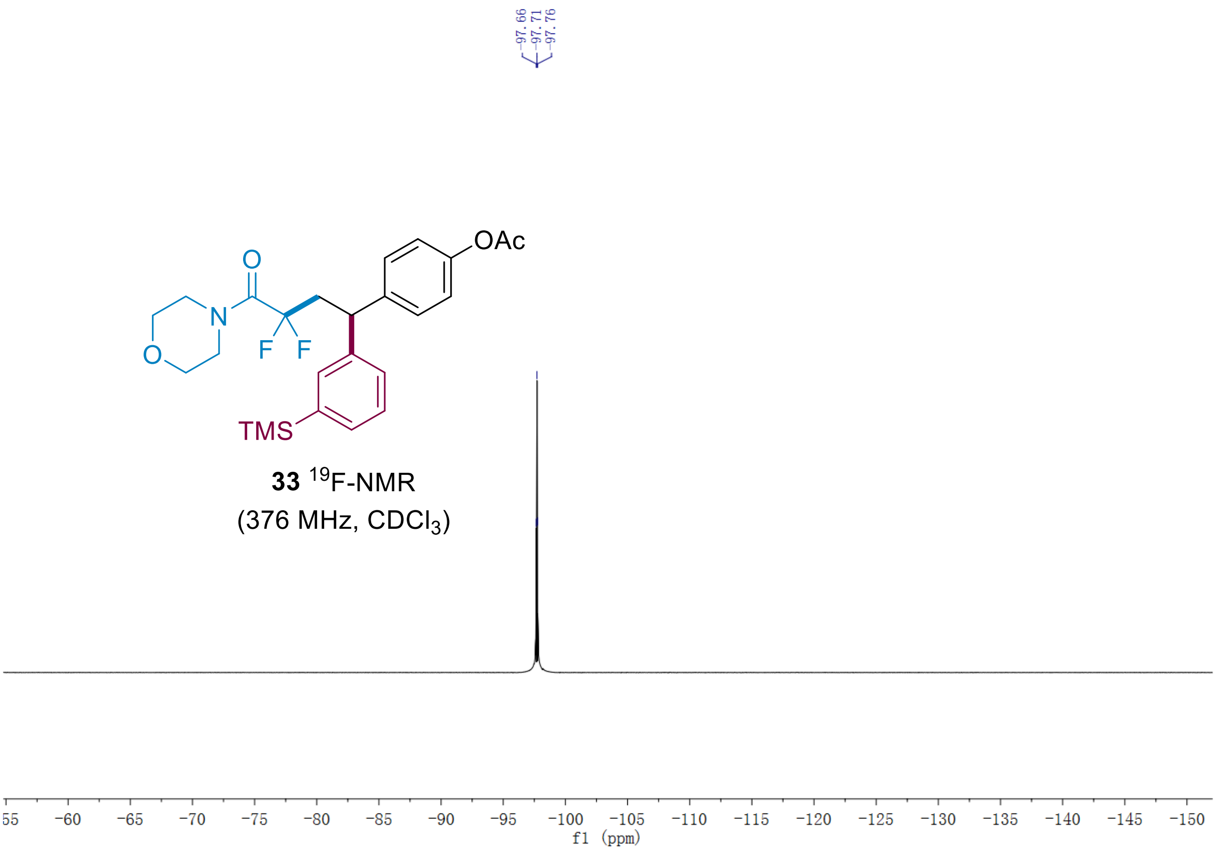
-97.66
-97.71
-97.76
OAc
O
N
O
F F
TMS
33 19F-NMR
(376 MHz, CDCl3)
f1 (ppm)

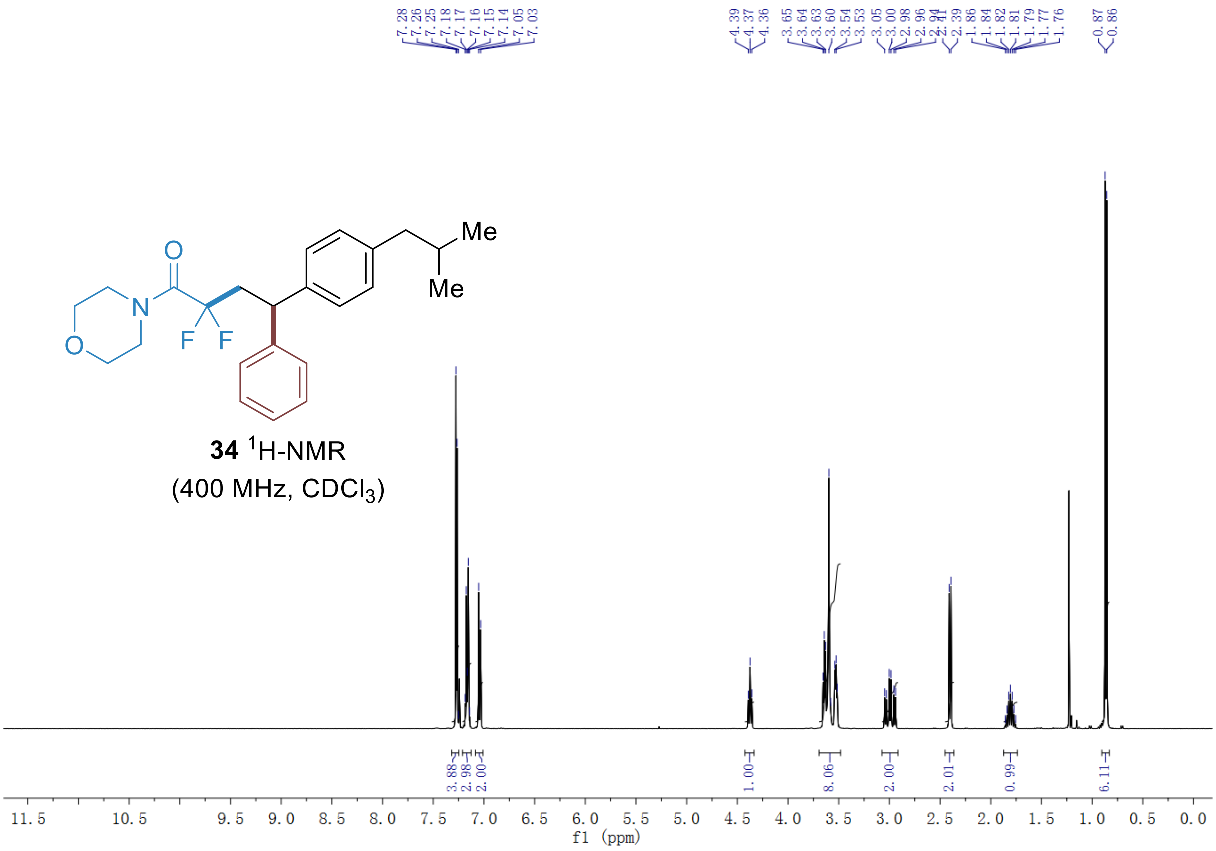

34 ¹H-NMR
(400 MHz, CDCl3)
f1 (ppm)

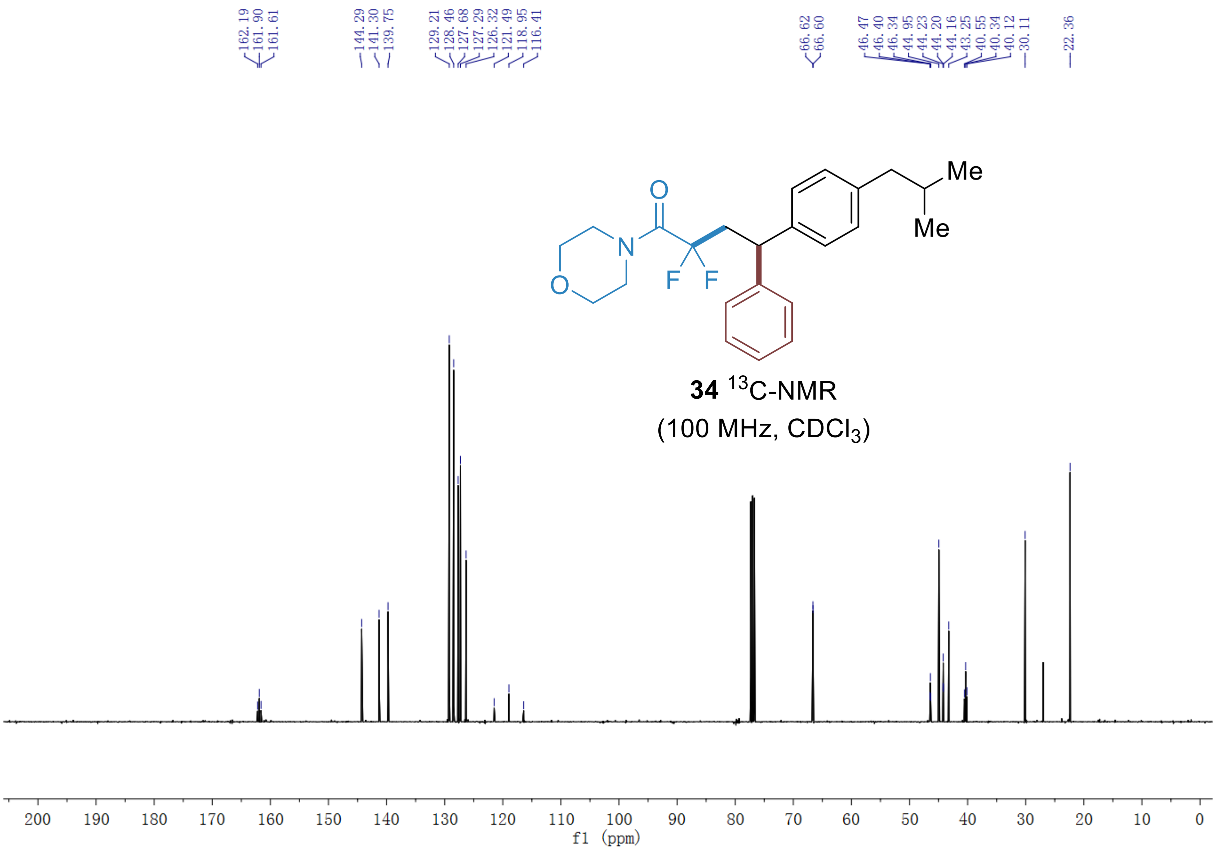

34 ¹³C-NMR
(100 MHz, CDCl3)
f1 (ppm)

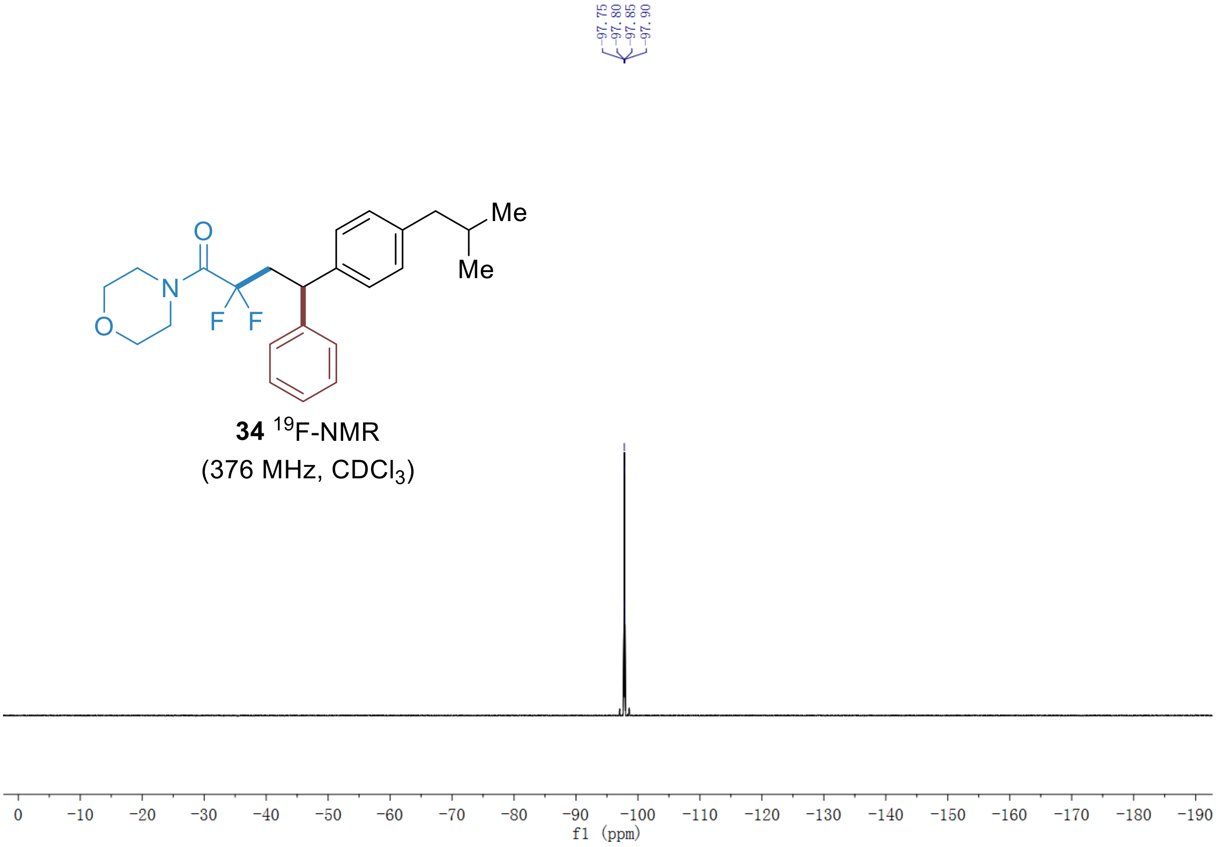
-97.75
-97.80
-97.85
-97.90
Me
Me
O
N
O
F F
34 19F-NMR
(376 MHz, CDCl3)
f1 (ppm)

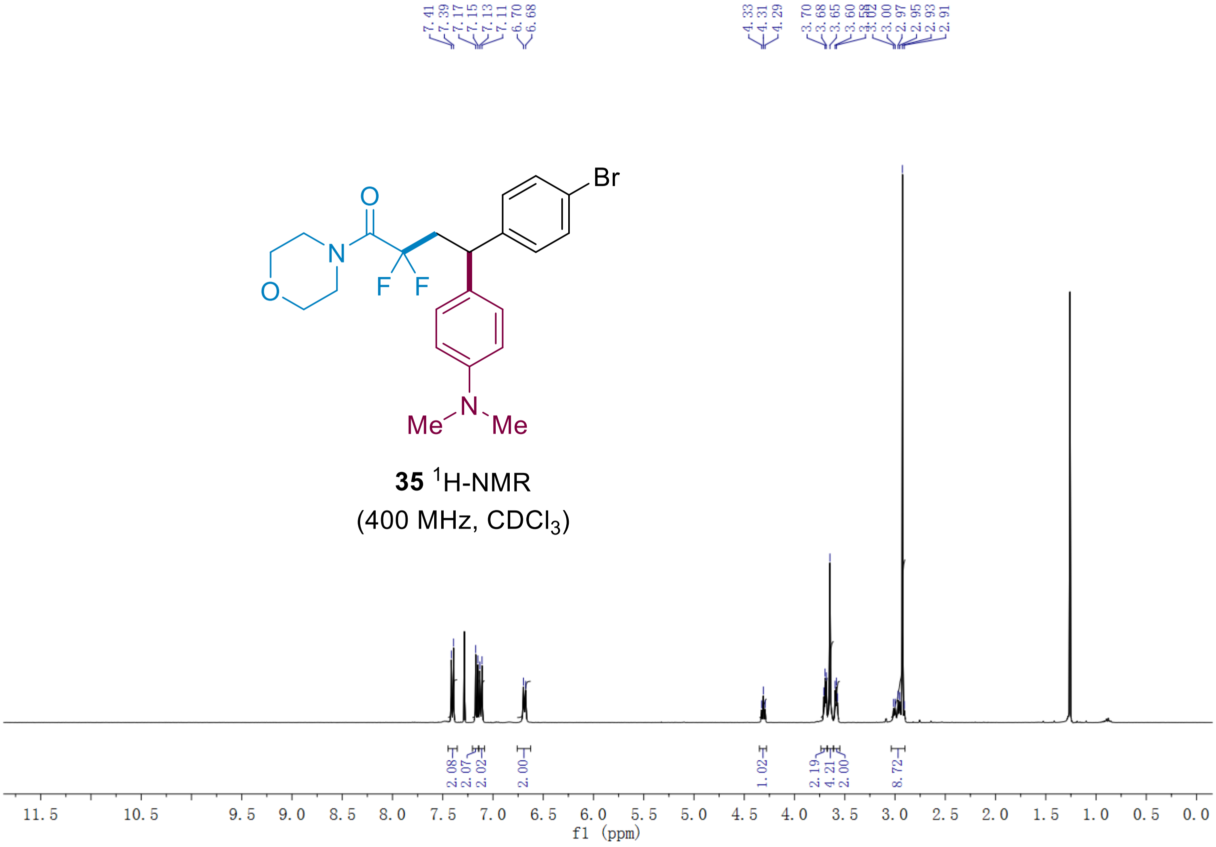
7.41
7.39
7.17
7.15
7.13
7.11
6.70
6.68
4.33
4.31
4.29
3.70
3.68
3.65
3.60
3.00
2.97
2.95
2.93
2.91
Br
O
N
O
F F
Me
N
Me
35 1H-NMR
(400 MHz, CDCl3)
2.08
2.07
2.02
2.00
1.02
2.19
4.21
2.00
8.72
f1 (ppm)

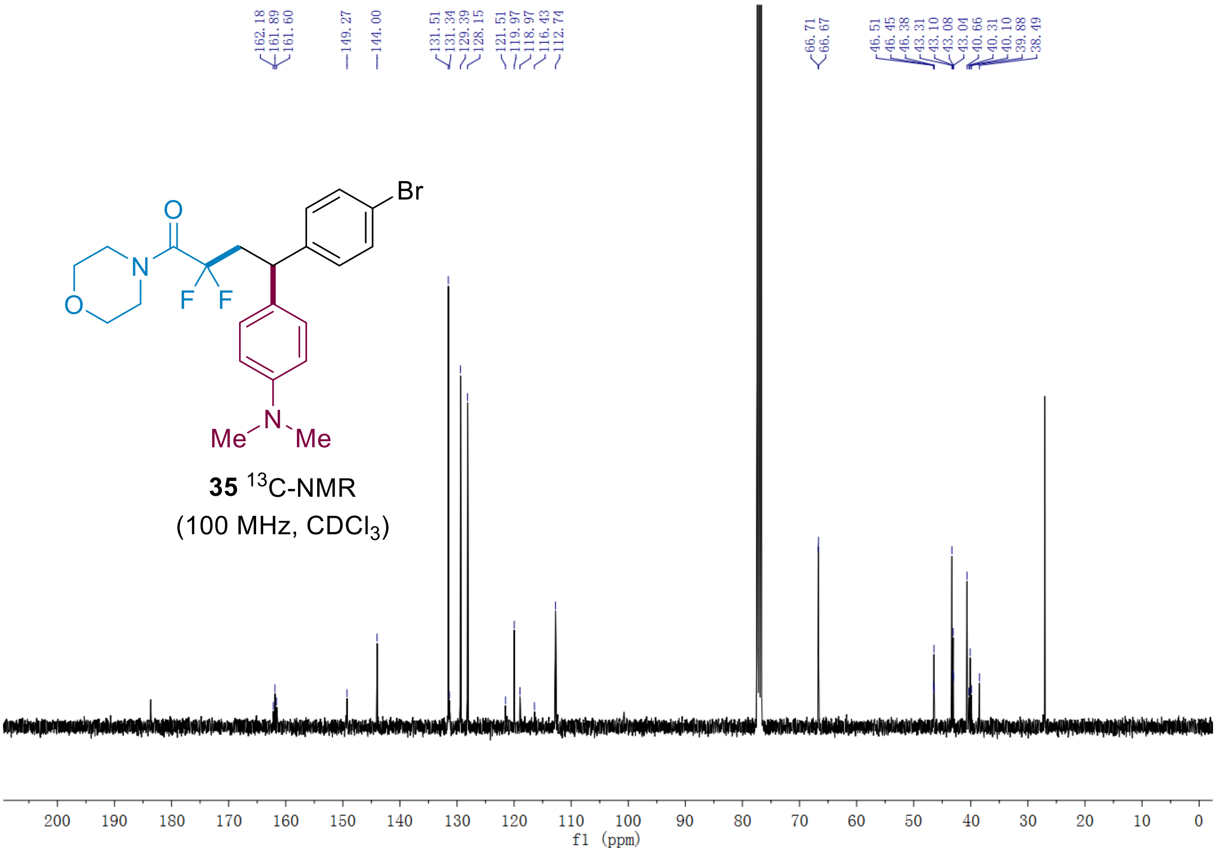
162.18
161.89
161.60
149.27
144.00
131.51
131.34
129.39
128.15
121.51
119.97
118.97
116.43
112.74
66.71
66.67
46.51
46.45
46.38
43.31
43.10
43.08
43.04
40.66
40.31
40.10
39.88
38.49
Br
O
N
O
F F
N
Me Me
35 13C-NMR
(100 MHz, CDCl3)
f1 (ppm)

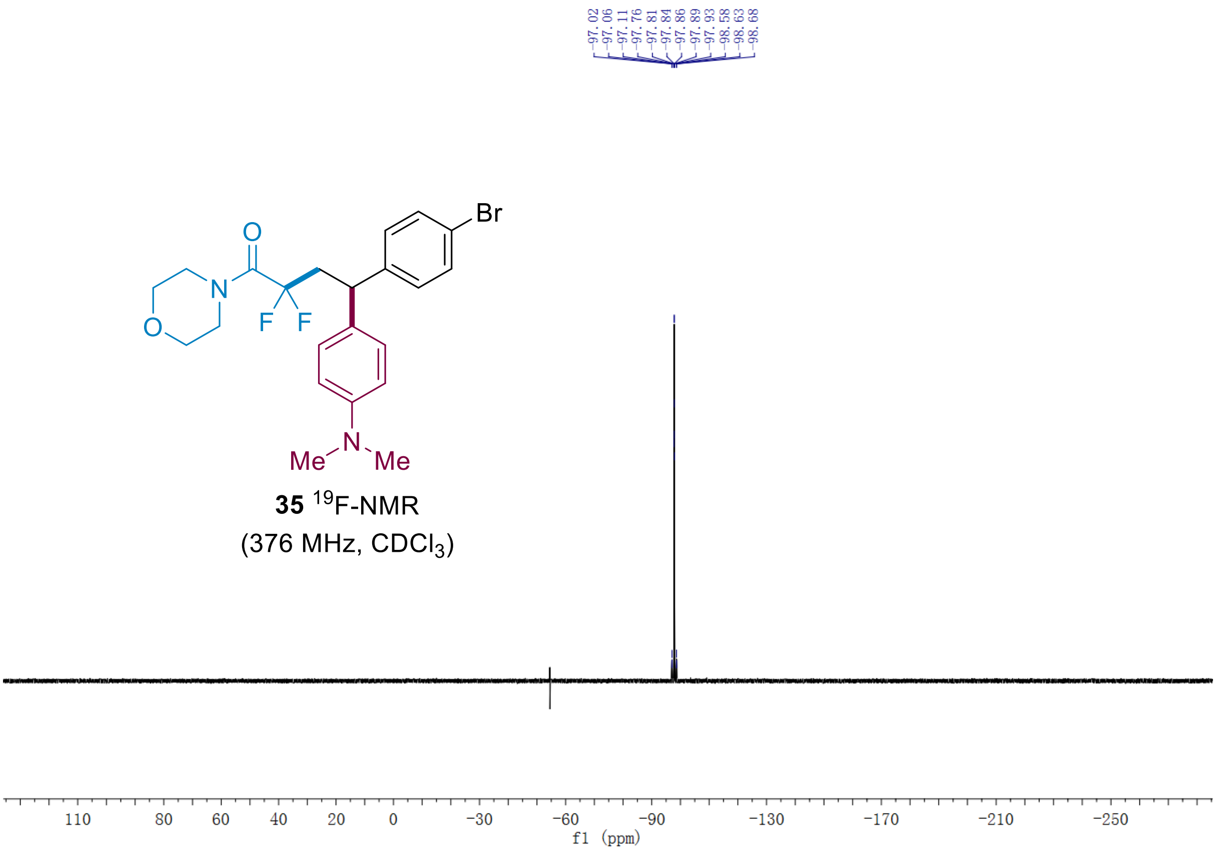
-97.02
-97.06
-97.11
-97.76
-97.81
-97.84
-97.86
-97.89
-97.93
-98.58
-98.63
-98.68
Br
O
N
O
F F
N
Me Me
35 19F-NMR
(376 MHz, CDCl3)
f1 (ppm)

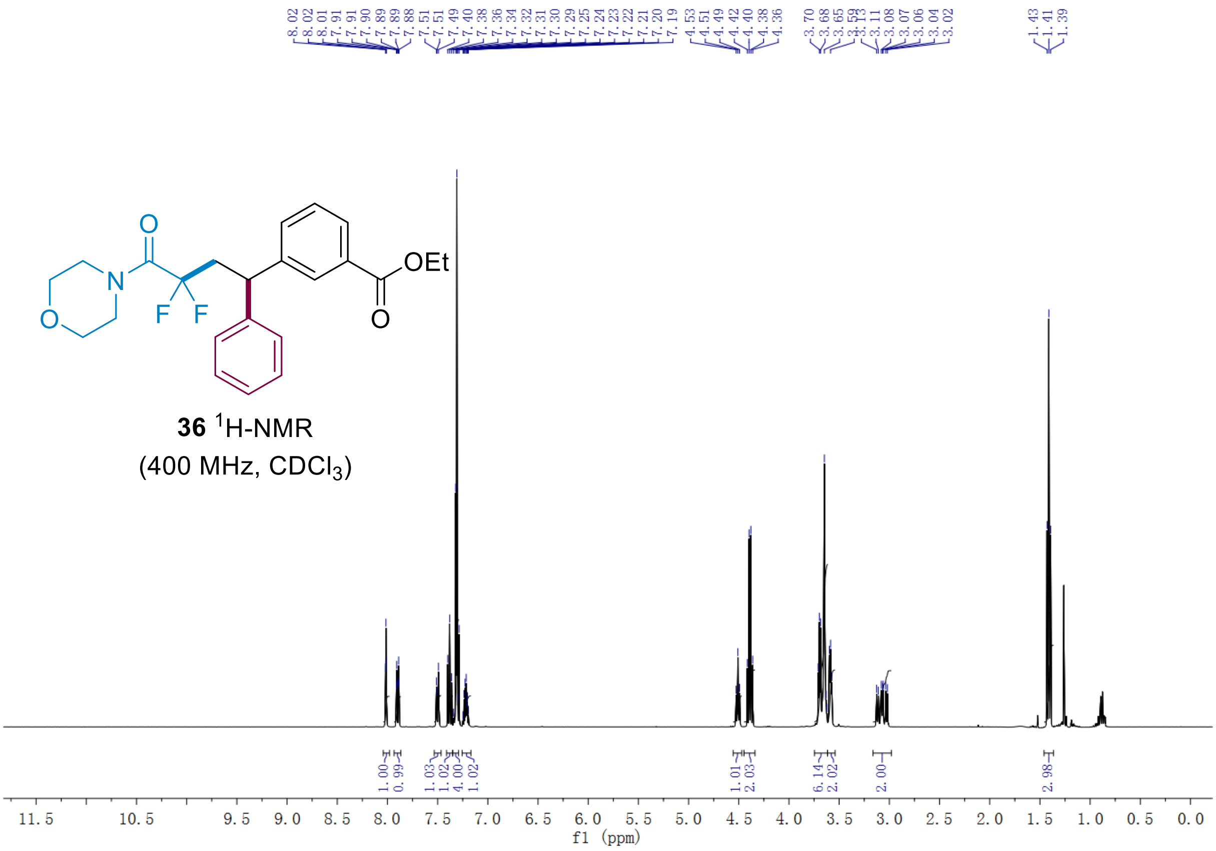
8.02 8.02 8.01 7.91 7.91 7.90 7.89 7.89 7.88 7.51 7.51 7.49 7.40 7.38 7.36 7.34 7.32 7.31 7.30 7.29 7.25 7.24 7.23 7.22 7.21 7.20 7.19 4.53 4.51 4.49 4.42 4.40 4.38 4.36 3.70 3.68 3.65 3.59 3.13 3.11 3.08 3.07 3.06 3.04 3.02 1.43 1.41 1.39
OEt
O
N
O
F F
O
36 1H-NMR
(400 MHz, CDCl3)
1.00 0.99 1.03 1.02 4.00 1.02 1.01 2.03 6.14 2.02 2.00 2.98
f1 (ppm)

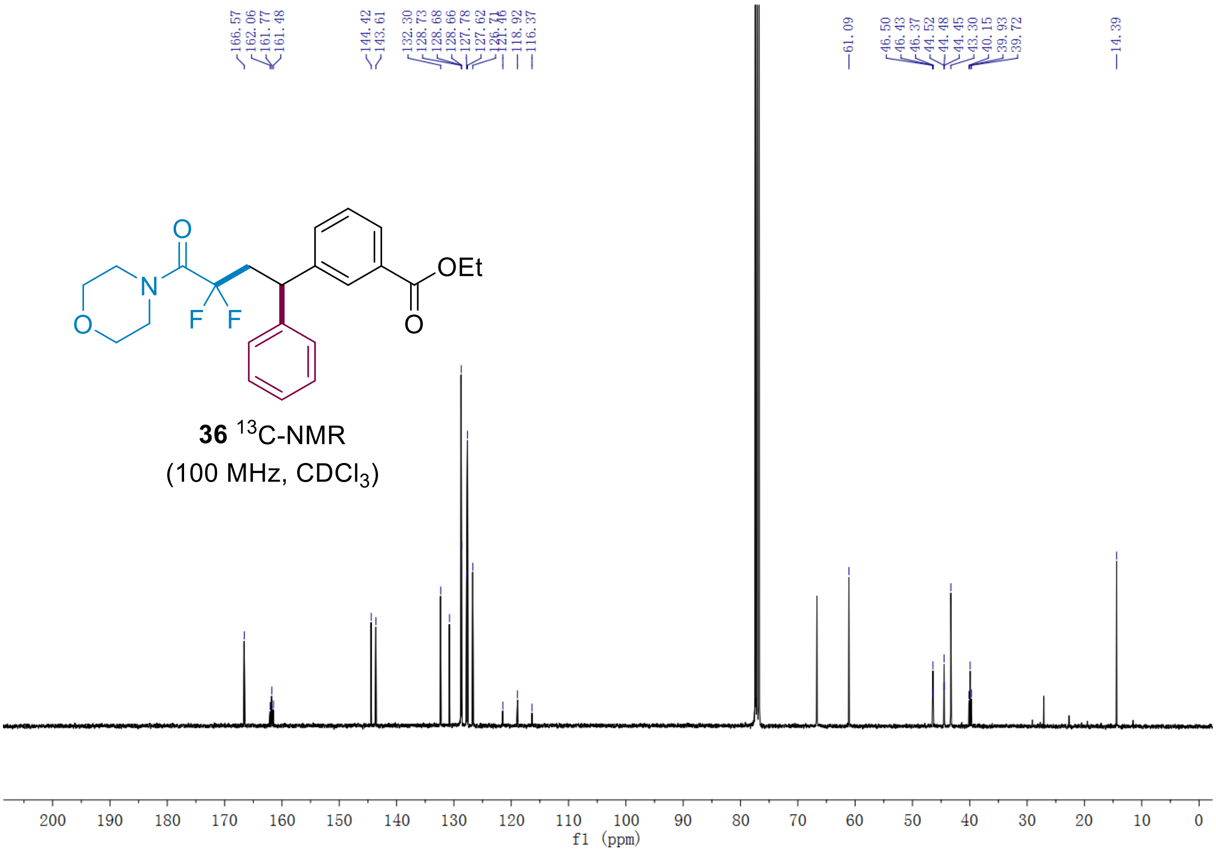
166.57 162.06 161.77 161.48 144.42 143.61 132.30 128.73 128.68 128.66 127.78 127.62 126.71 121.46 118.92 116.37 61.09 46.50 46.43 46.37 44.52 44.48 44.45 43.30 40.15 39.93 39.72 14.39
OEt
O
N
O
F F
O
36 13C-NMR
(100 MHz, CDCl3)
f1 (ppm)

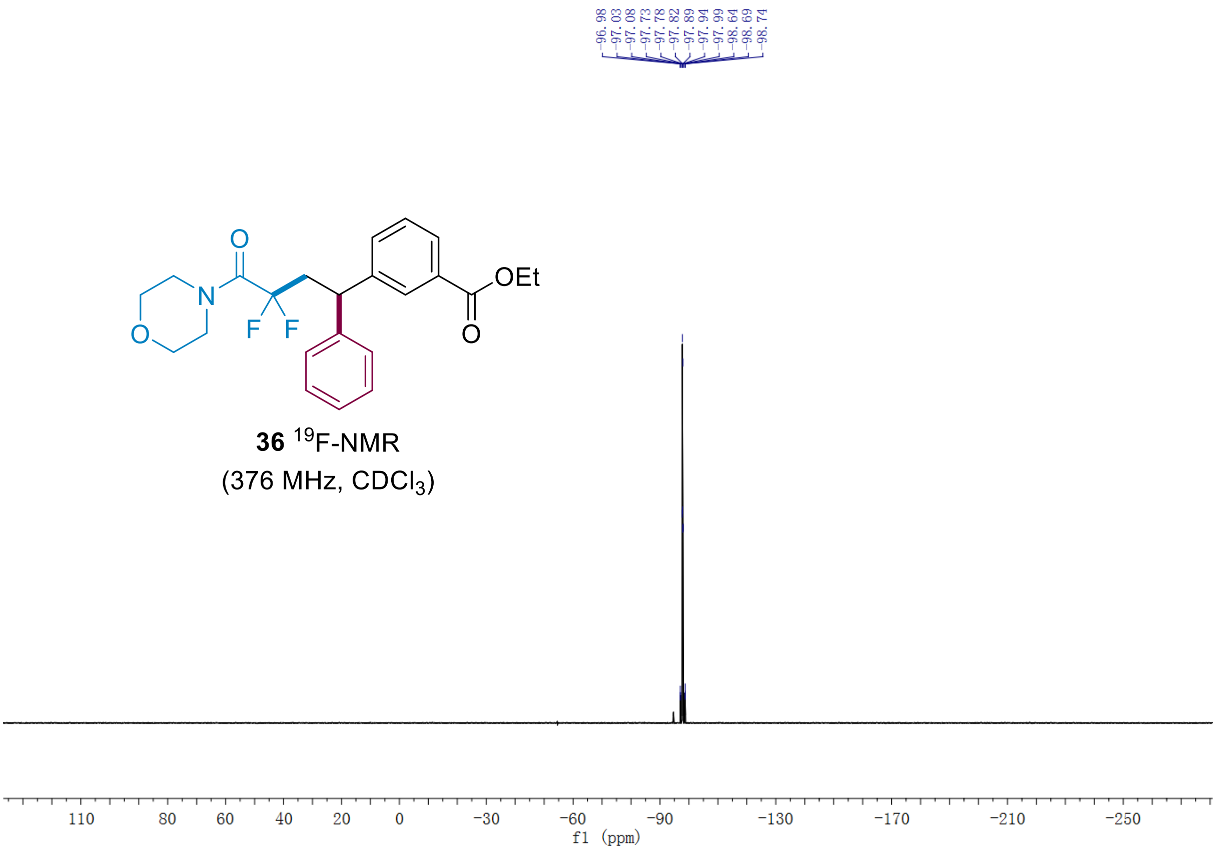
-96.98
-97.03
-97.08
-97.73
-97.78
-97.82
-97.89
-97.94
-97.99
-98.64
-98.69
-98.74
O
N
O
F F
OEt
O
36 19F-NMR
(376 MHz, CDCl3)
110
80
60
40
20
0
-30
-60
-90
-130
-170
-210
-250
f1 (ppm)

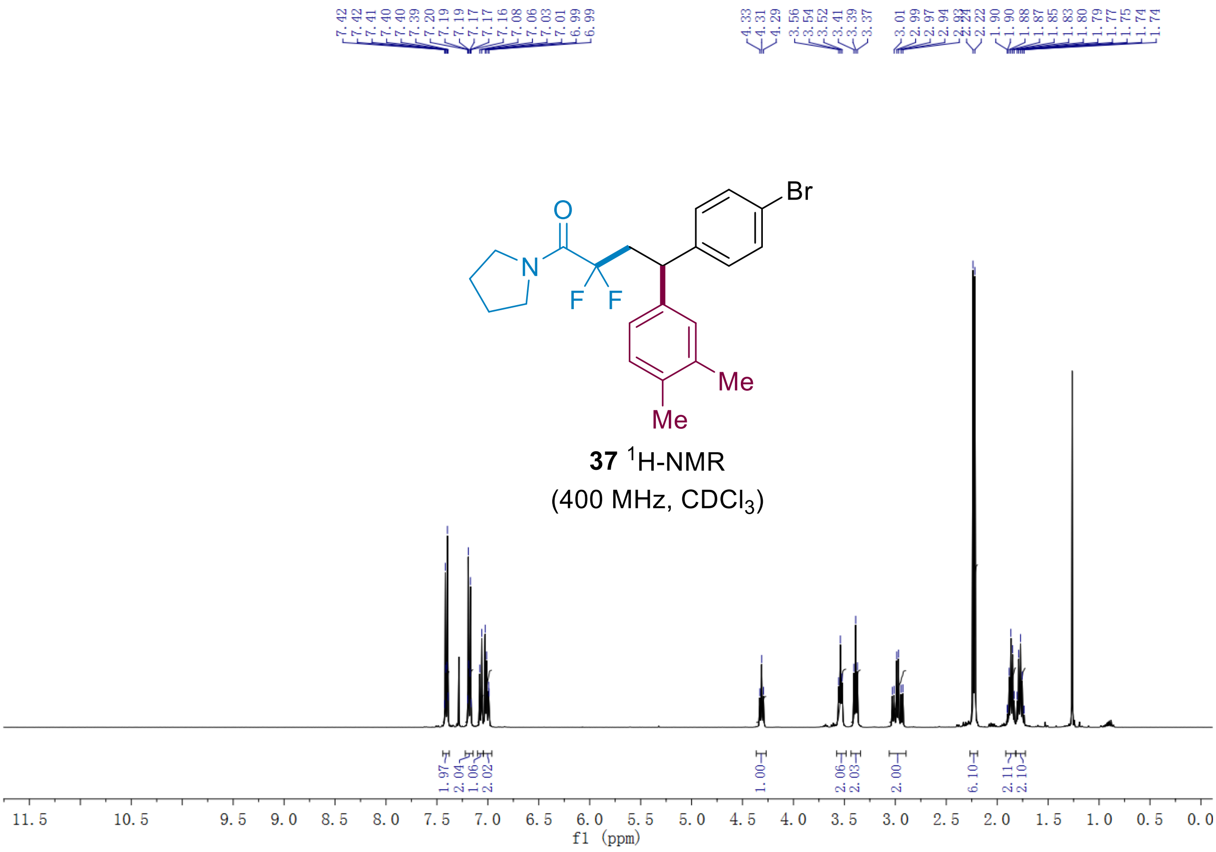
7.42
7.42
7.41
7.40
7.39
7.20
7.19
7.17
7.16
7.08
7.06
7.03
7.01
6.99
6.99
4.33
4.31
4.29
3.56
3.54
3.52
3.41
3.39
3.37
3.01
2.99
2.97
2.94
2.22
1.90
1.88
1.87
1.85
1.83
1.80
1.79
1.77
1.75
1.74
1.74
Br
O
N
F F
Me
Me
37 1H-NMR
(400 MHz, CDCl3)
1.97
2.04
1.06
2.02
1.00
2.06
2.03
2.00
6.10
2.11
2.10
11.5
10.5
9.5
9.0
8.5
8.0
7.5
7.0
6.5
6.0
5.5
5.0
4.5
4.0
3.5
3.0
2.5
2.0
1.5
1.0
0.5
0.0
f1 (ppm)

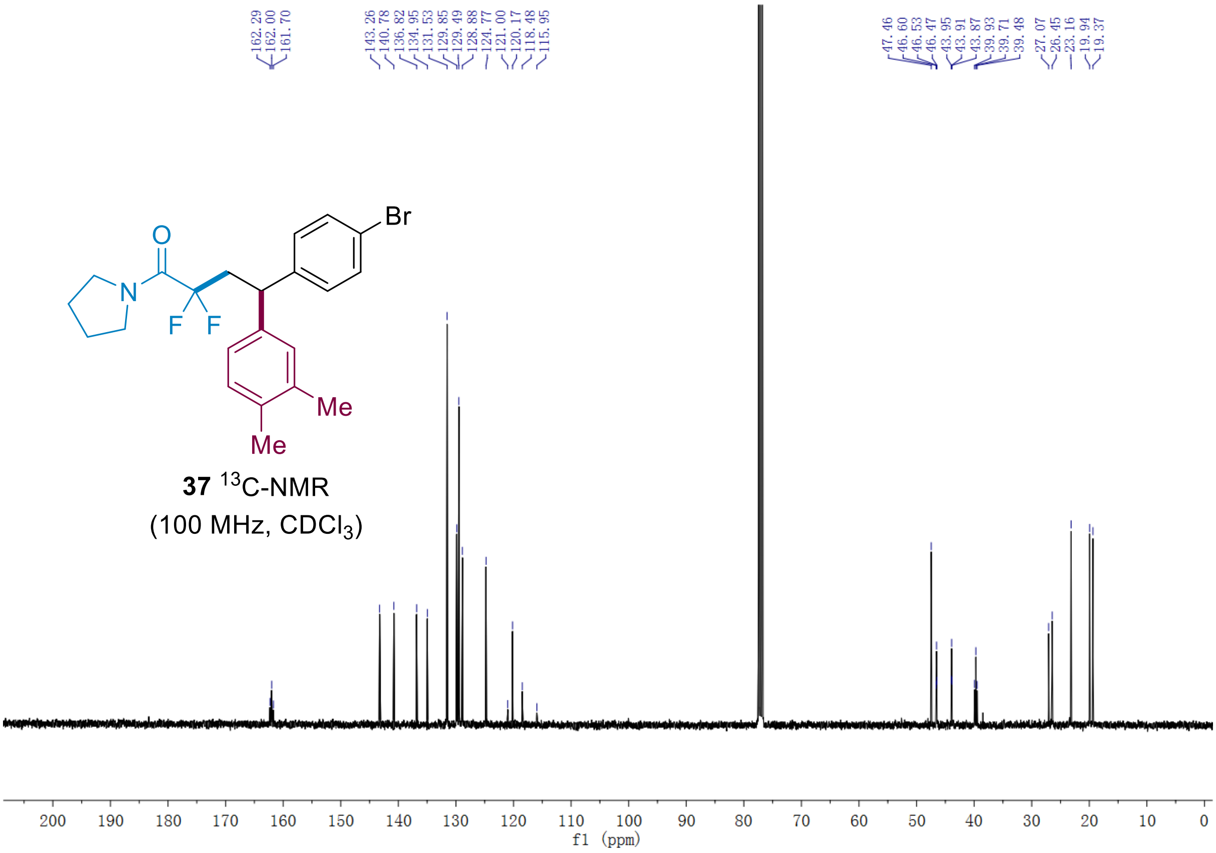
162.29
162.00
161.70
143.26
140.78
136.82
134.95
131.53
129.85
129.49
128.88
124.77
121.00
120.17
118.48
115.95
47.46
46.60
46.53
46.47
43.95
43.91
43.87
39.93
39.71
39.48
27.07
26.45
23.16
19.94
19.37
O
N
F F
Br
Me
Me
37 13C-NMR
(100 MHz, CDCl3)
f1 (ppm)

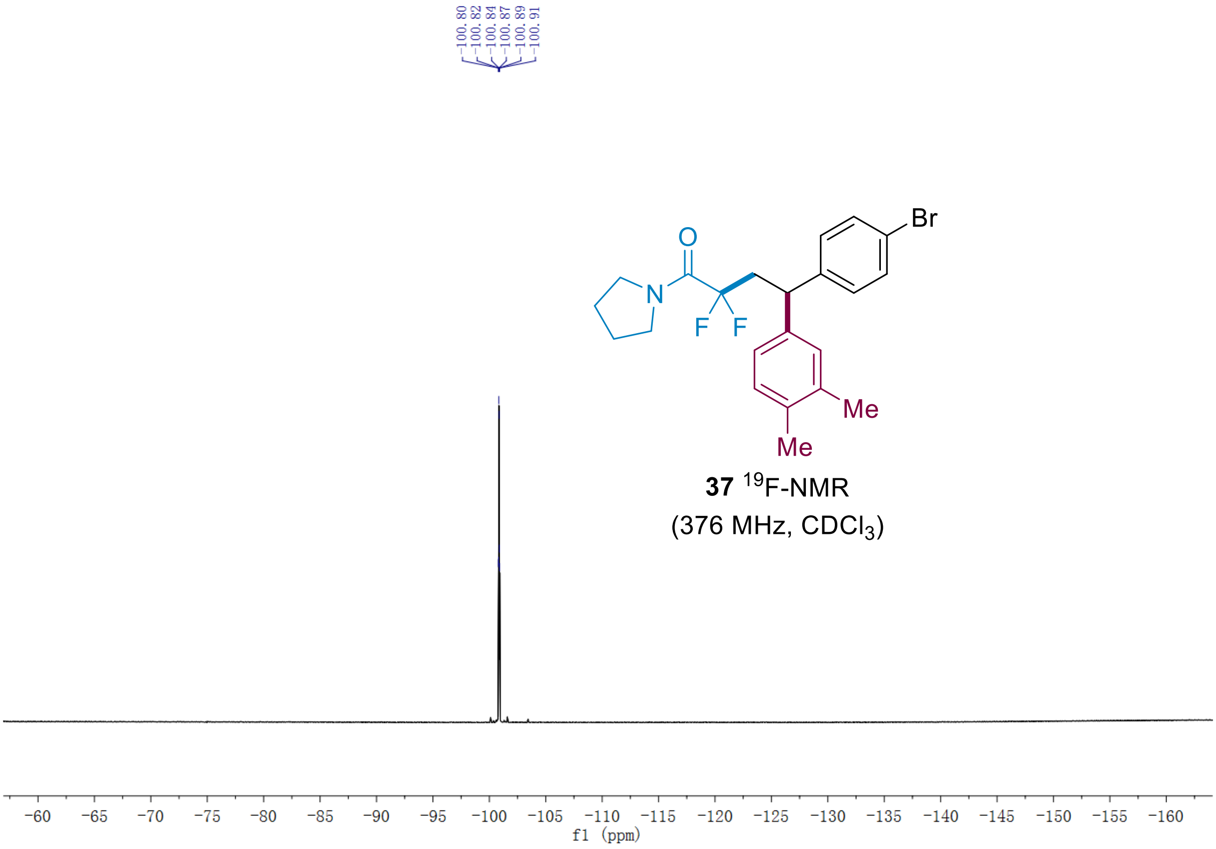
-100.80
-100.82
-100.84
-100.87
-100.89
-100.91
O
N
F F
Br
Me
Me
37 19F-NMR
(376 MHz, CDCl3)
f1 (ppm)

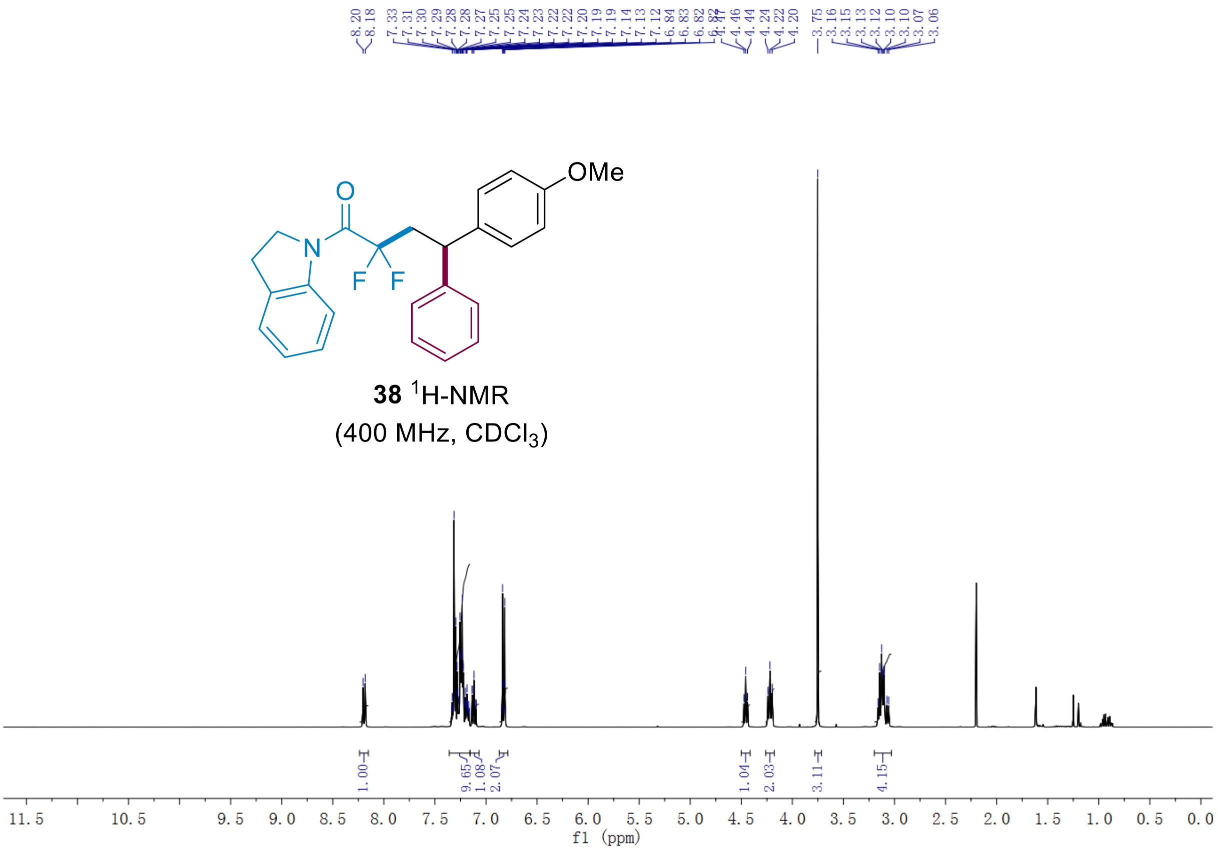

38 ^{1}H-NMR
(400 MHz, $CDCl_3$)

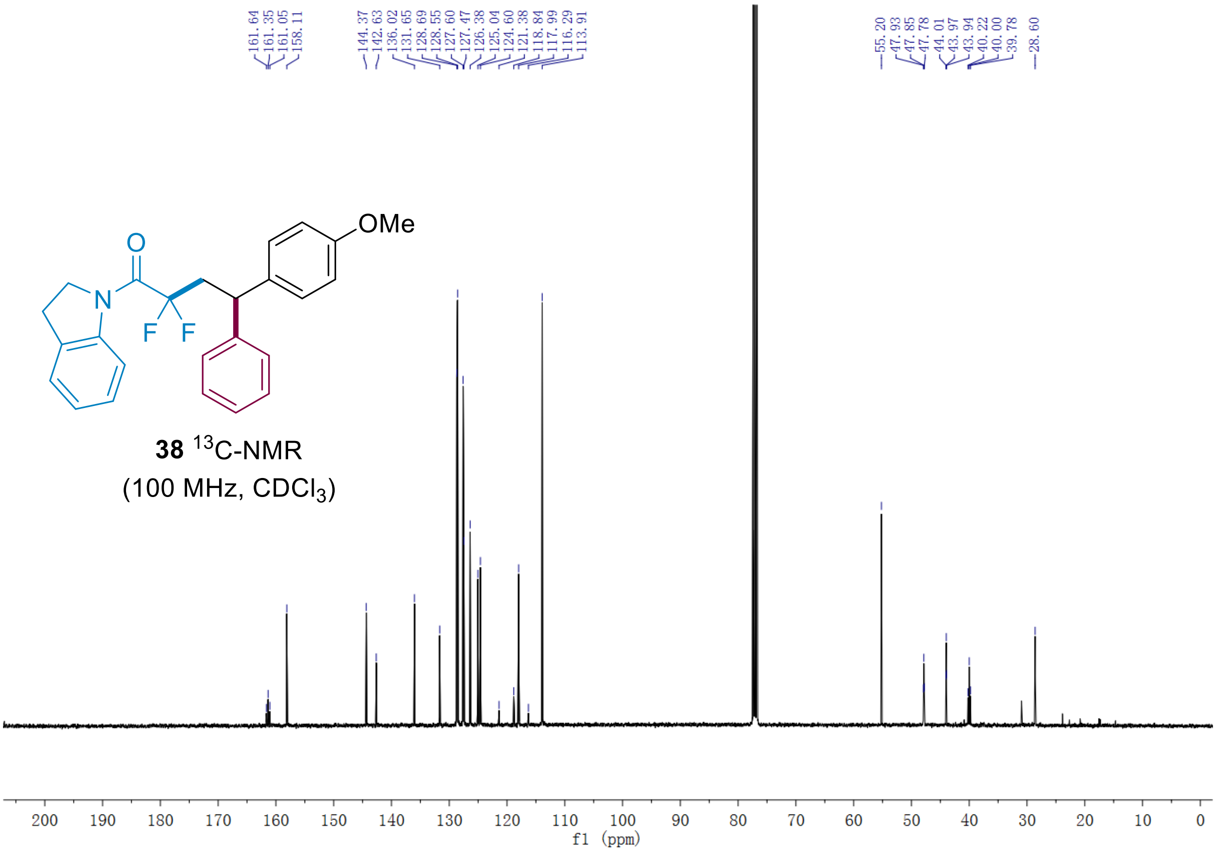

38 ^{13}C-NMR
(100 MHz, $CDCl_3$)

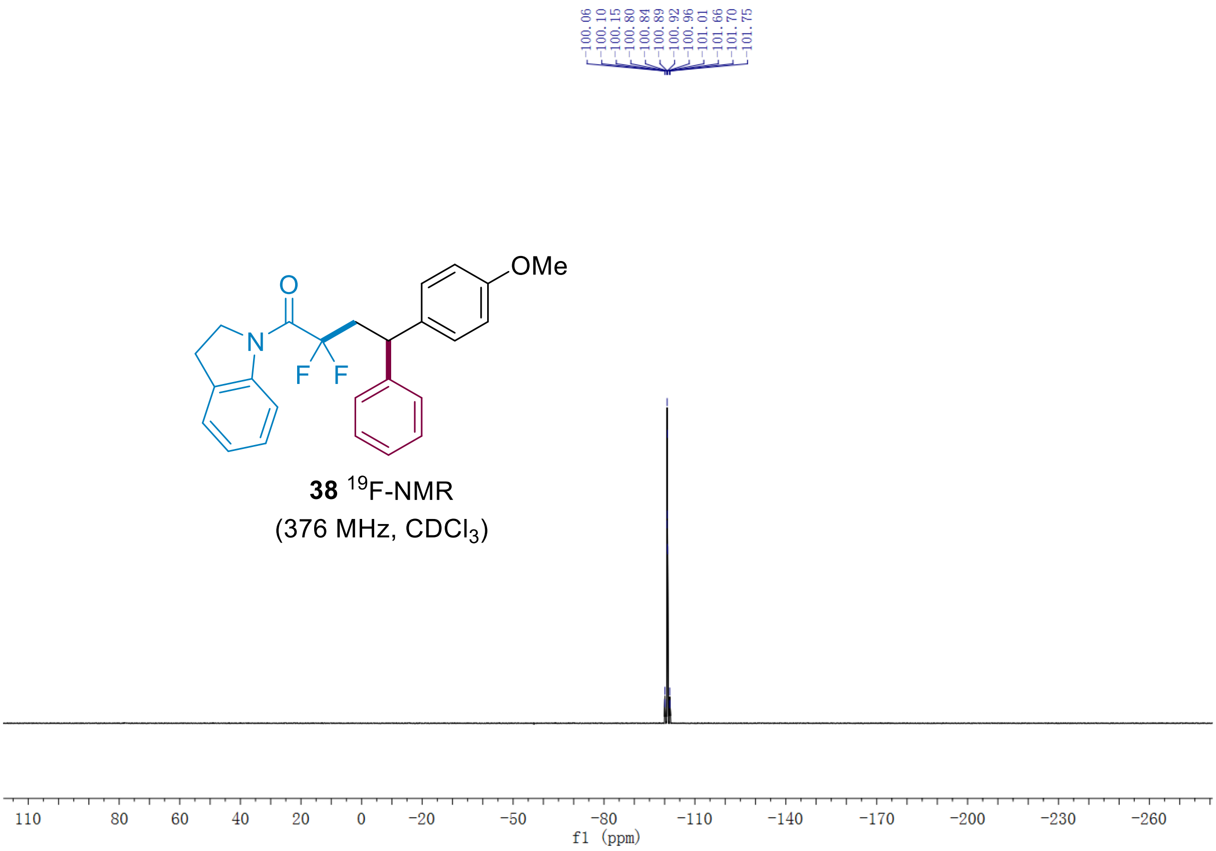
-100.06
-100.10
-100.15
-100.80
-100.84
-100.89
-100.92
-100.96
-101.01
-101.66
-101.70
-101.75
OMe
O
N
F F
38 19F-NMR
(376 MHz, CDCl3)
f1 (ppm)

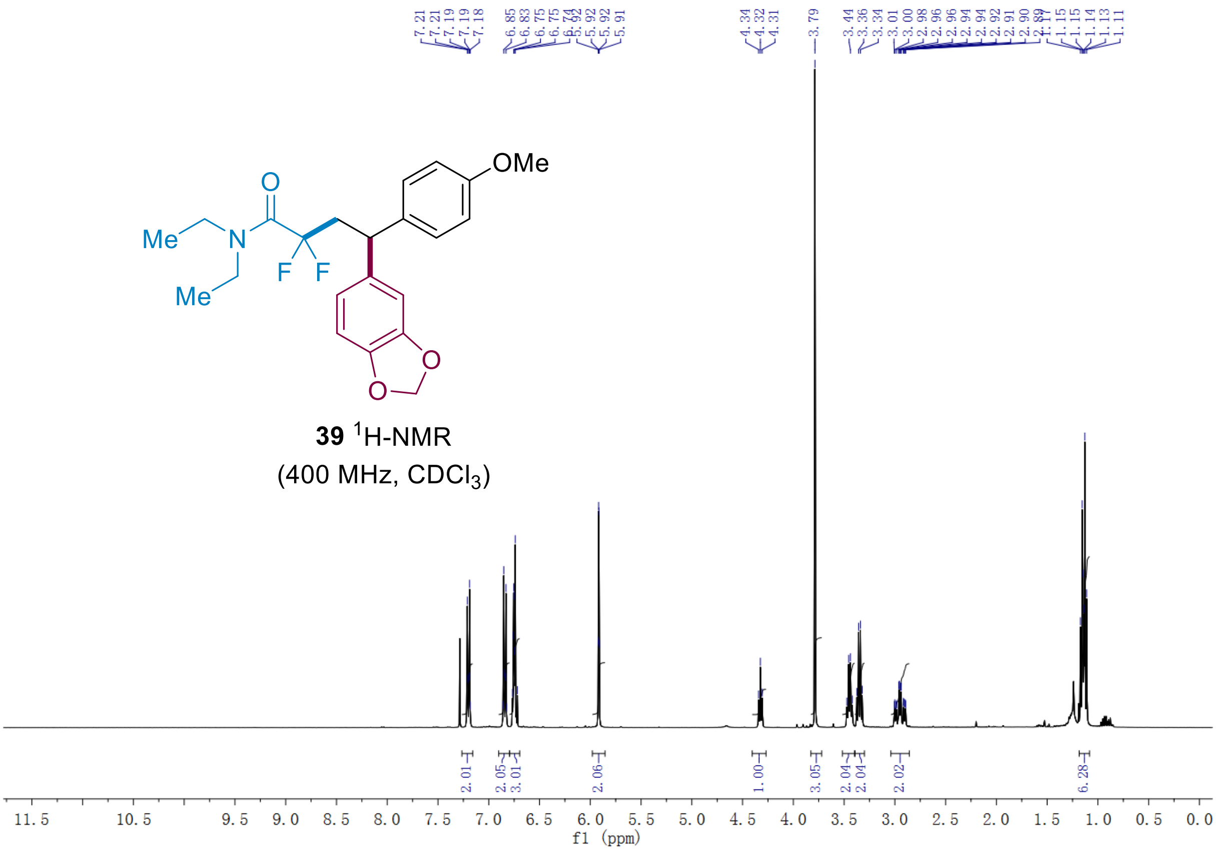
7.21
7.21
7.19
7.18
6.85
6.83
6.75
6.75
6.74
6.72
5.92
5.92
5.91
4.34
4.32
4.31
3.79
3.44
3.36
3.34
3.01
3.00
2.98
2.96
2.94
2.94
2.92
2.91
2.90
1.17
1.15
1.15
1.14
1.13
1.11
OMe
O
Me
N
Me
F F
O
O
39 1H-NMR
(400 MHz, CDCl3)
2.01
2.05
3.01
2.06
1.00
3.05
2.04
2.04
2.02
6.28
f1 (ppm)

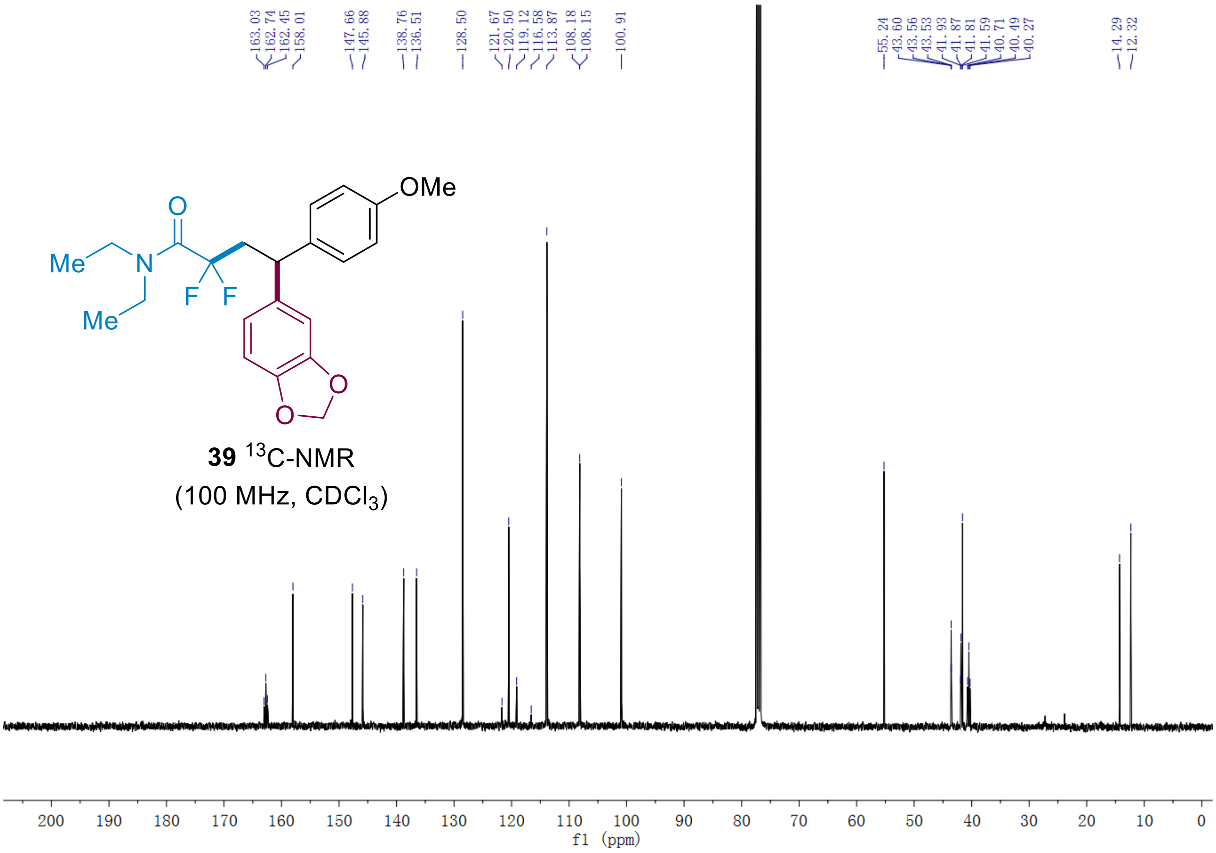

39 ^{13}C-NMR
(100 MHz, $CDCl_3$)

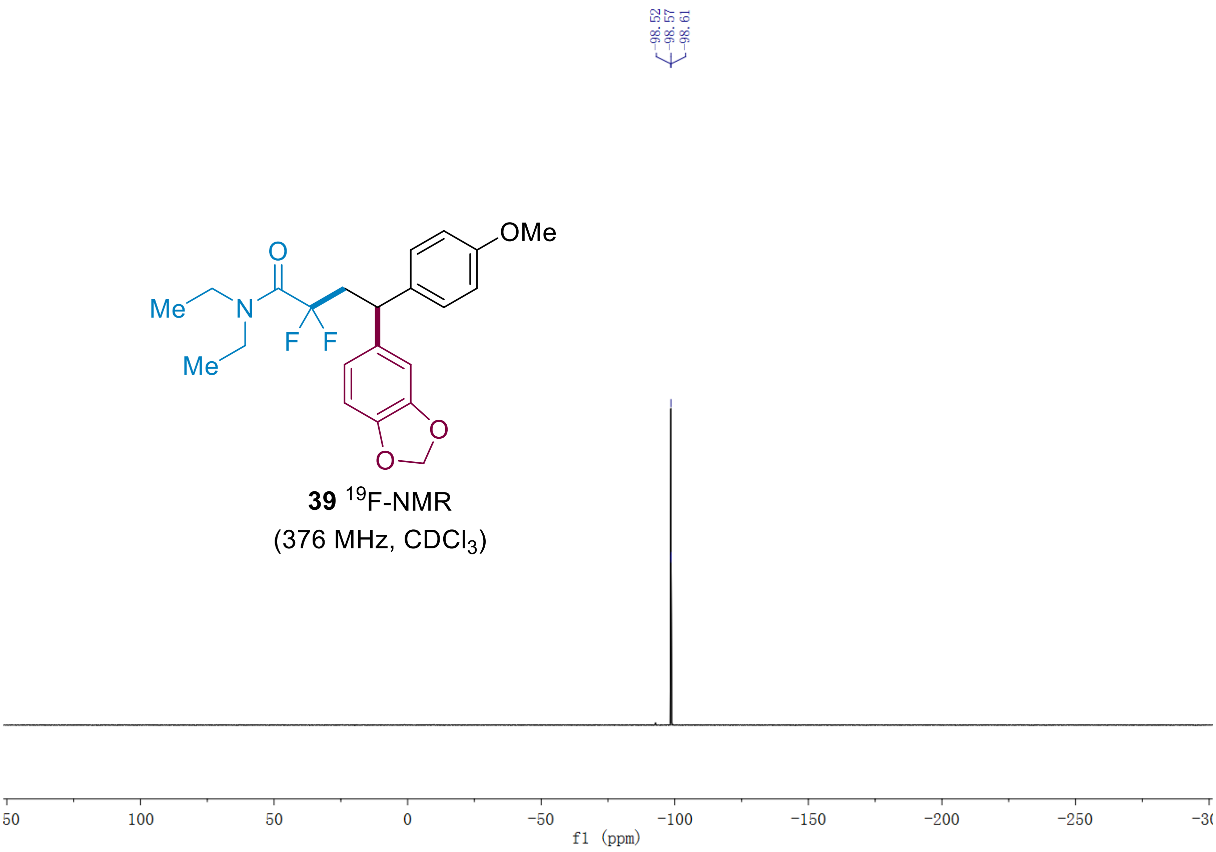

39 ^{19}F-NMR
(376 MHz, $CDCl_3$)

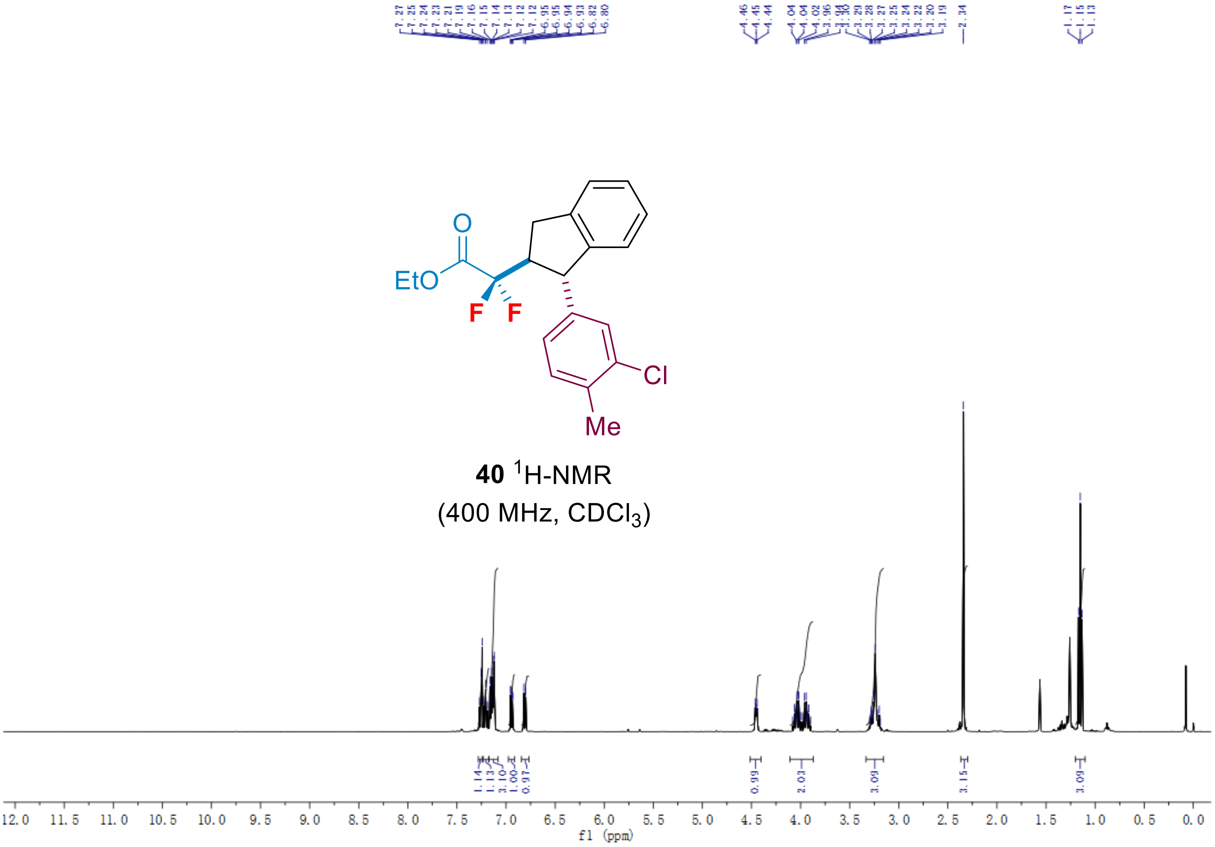
EtO
O
F
F
Cl
Me
40 ¹H-NMR
(400 MHz, CDCl3)
f1 (ppm)

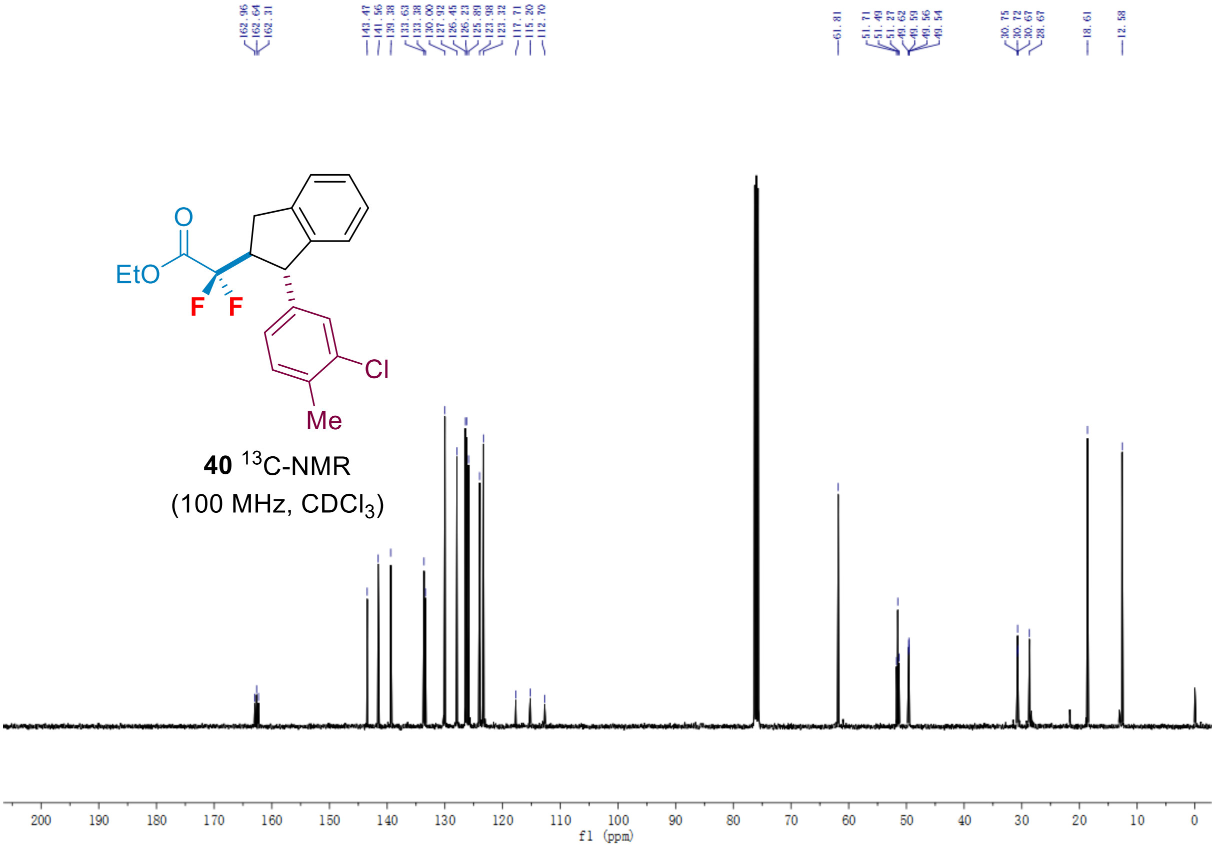
EtO
O
F
F
Cl
Me
40 ¹³C-NMR
(100 MHz, CDCl3)
f1 (ppm)

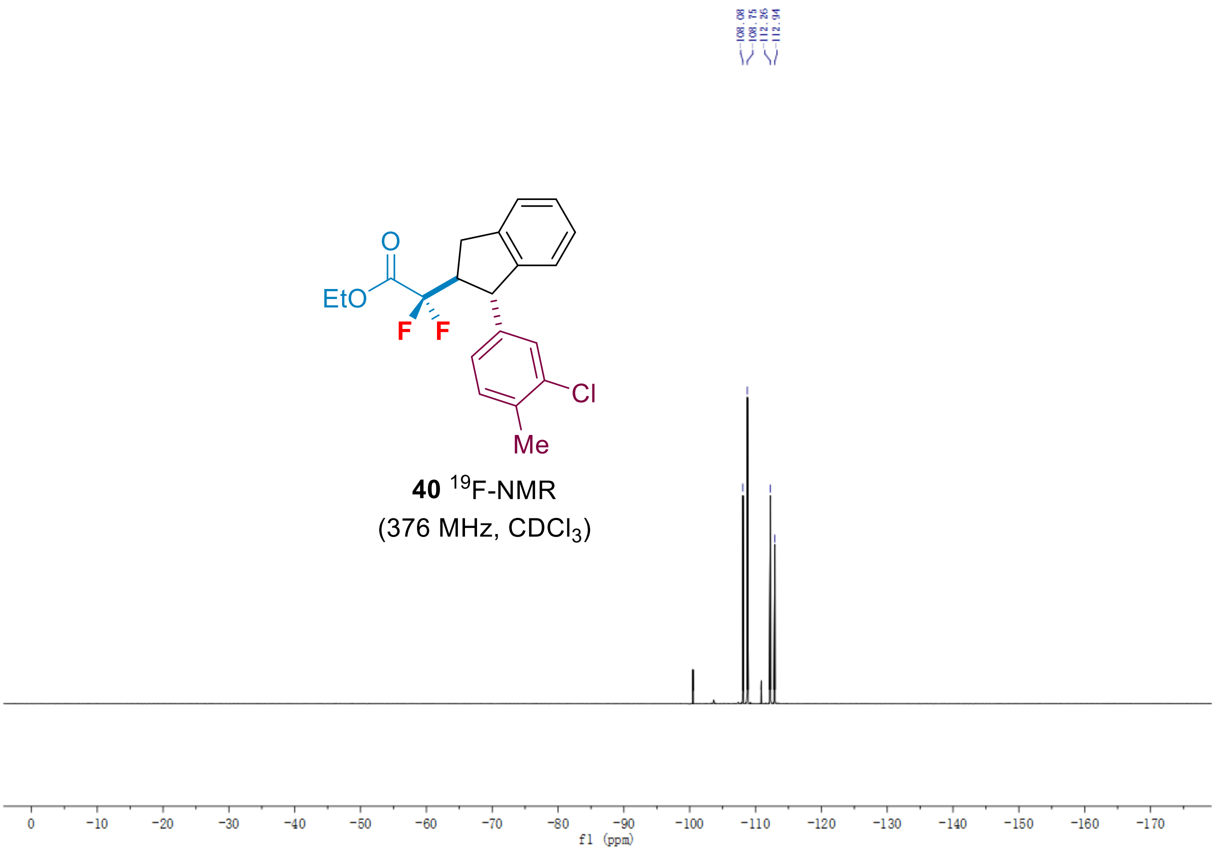
-108.08
-108.75
-112.26
-112.94
EtO
O
F
F
Cl
Me
40 19F-NMR
(376 MHz, CDCl3)
f1 (ppm)

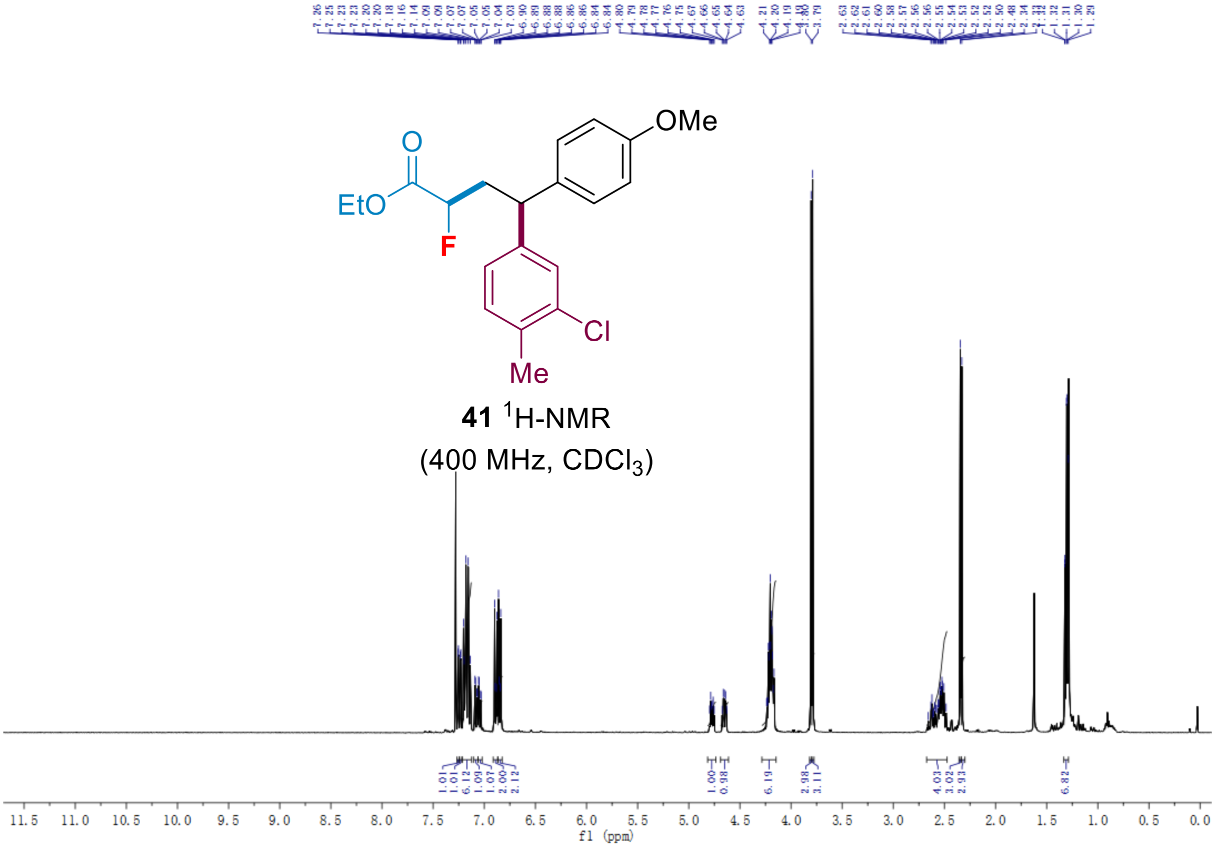
OMe
EtO
O
F
Cl
Me
41 1H-NMR
(400 MHz, CDCl3)
f1 (ppm)

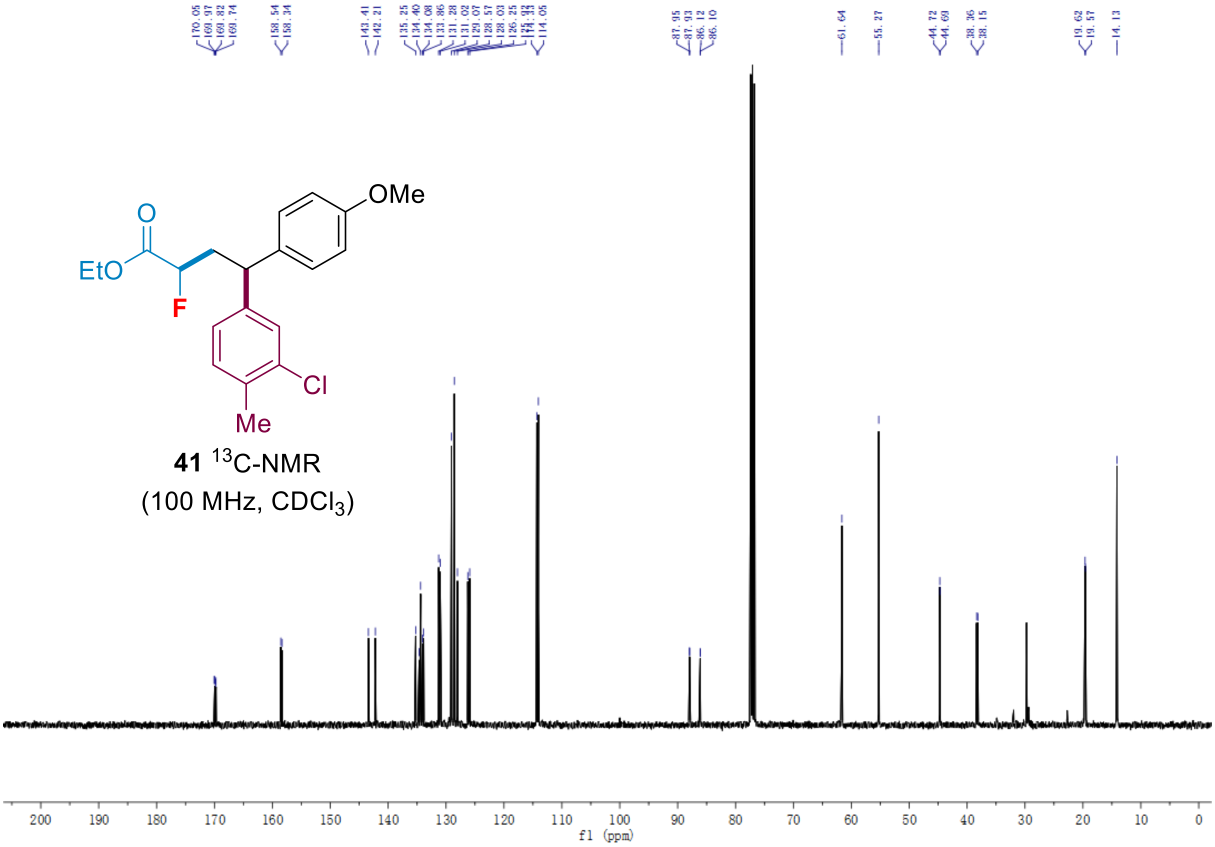
OMe
O
EtO
F
Cl
Me
41 13C-NMR
(100 MHz, CDCl3)
f1 (ppm)

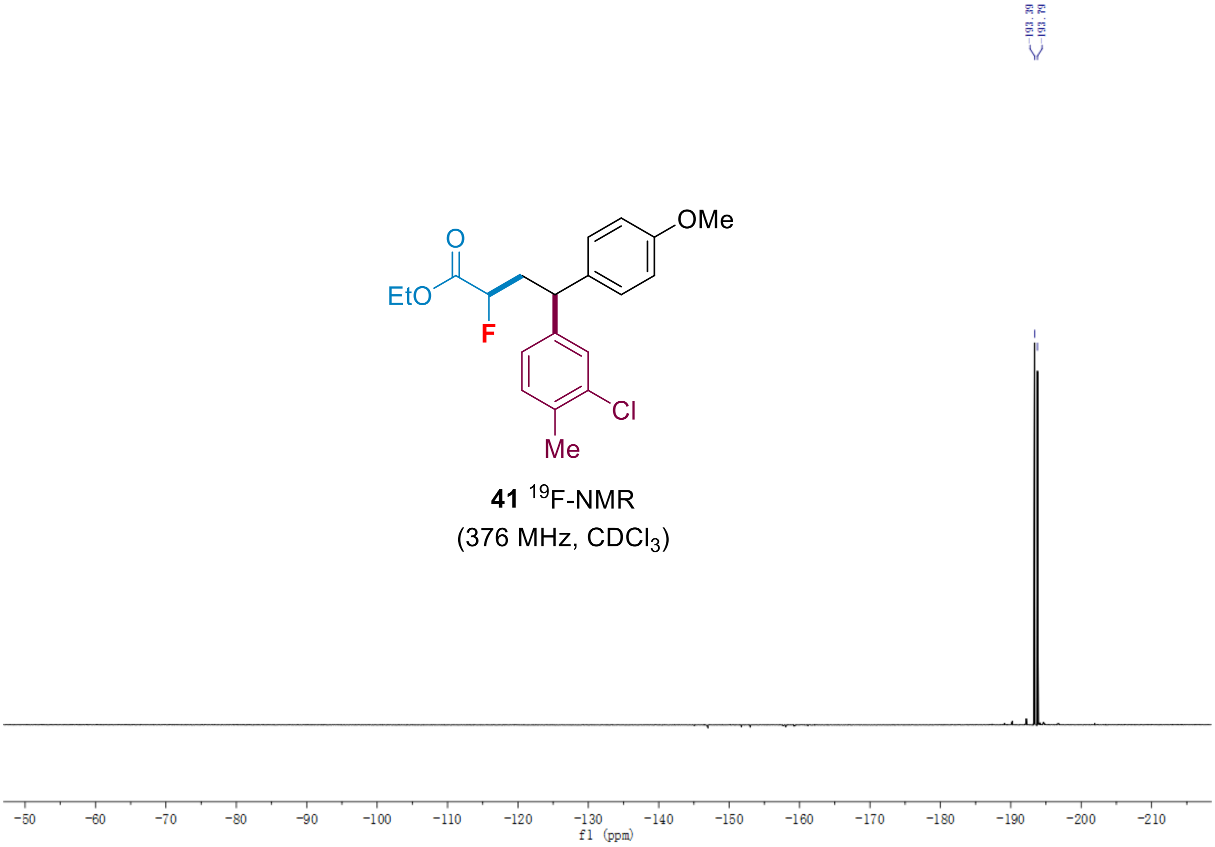
OMe
O
EtO
F
Cl
Me
41 19F-NMR
(376 MHz, CDCl3)
f1 (ppm)

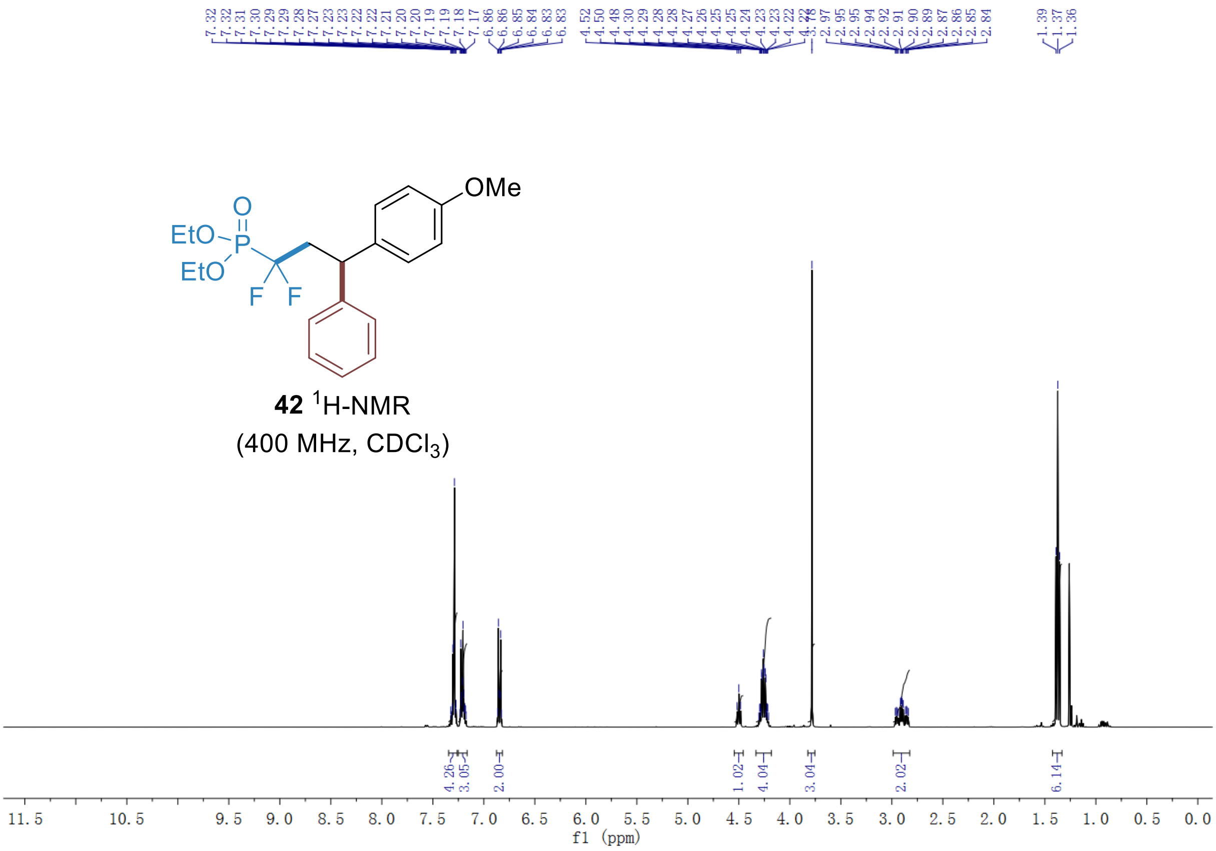
OMe
EtO
EtO
P
O
F F
42 ^{1}H-NMR
(400 MHz, $CDCl_3$)
f1 (ppm)

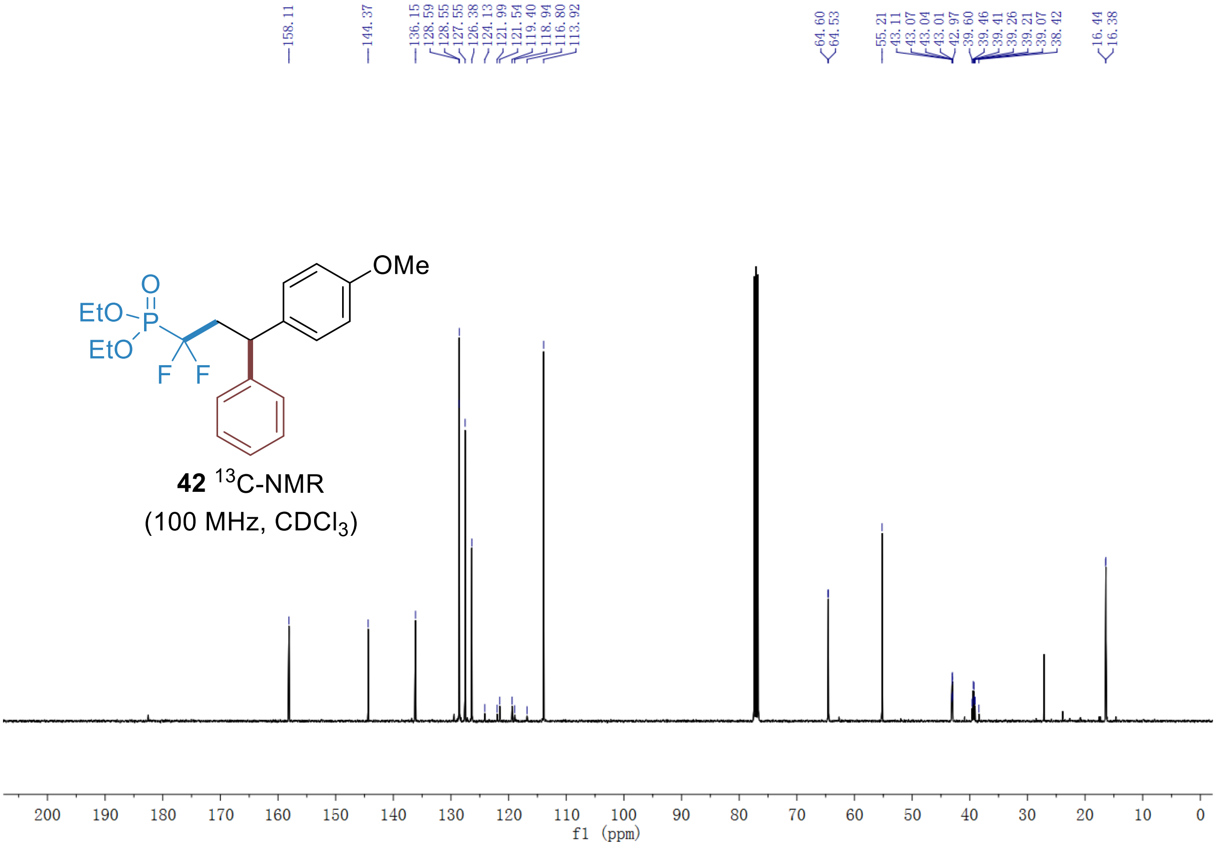
OMe
EtO
EtO
P
O
F F
42 ^{13}C-NMR
(100 MHz, $CDCl_3$)
f1 (ppm)

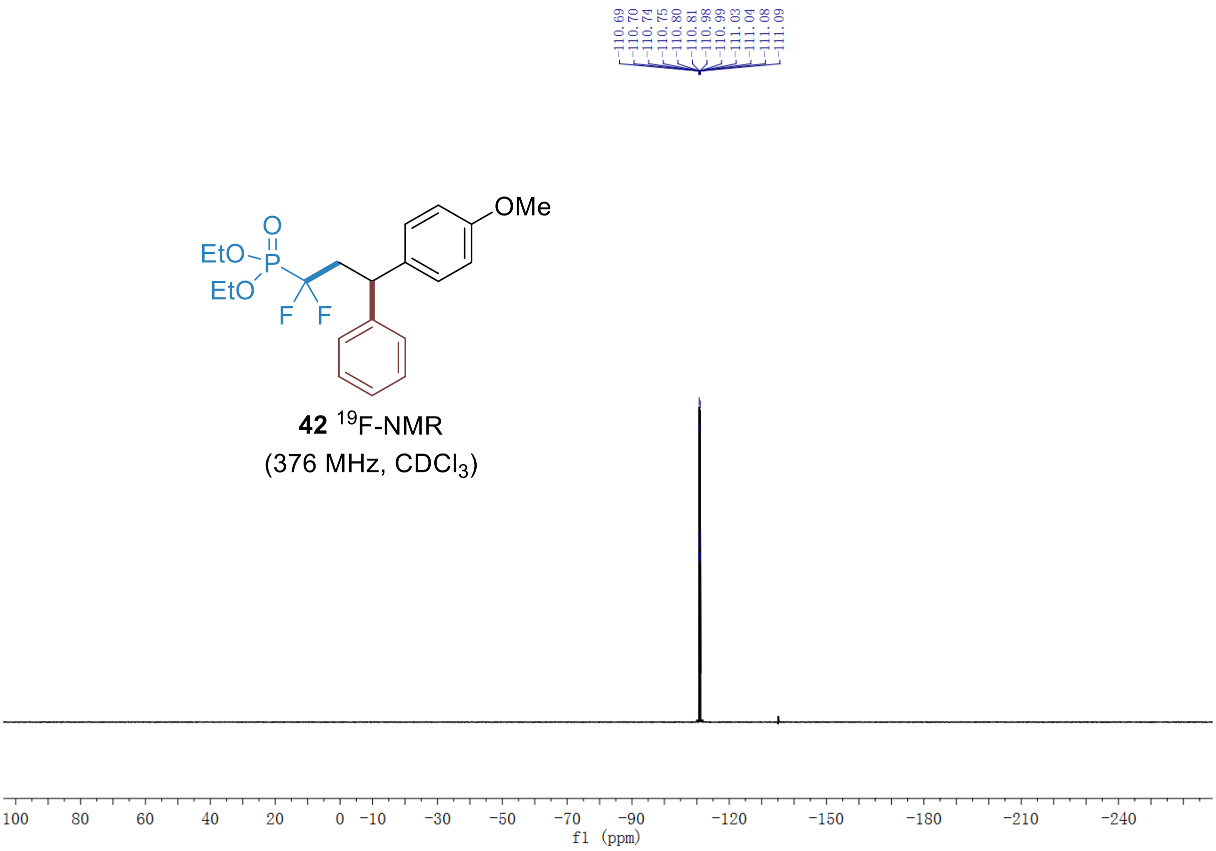

EtO
EtO
P
O
F F
OMe
42 ^{19}F-NMR
(376 MHz, CDCl$_3$)
f1 (ppm)

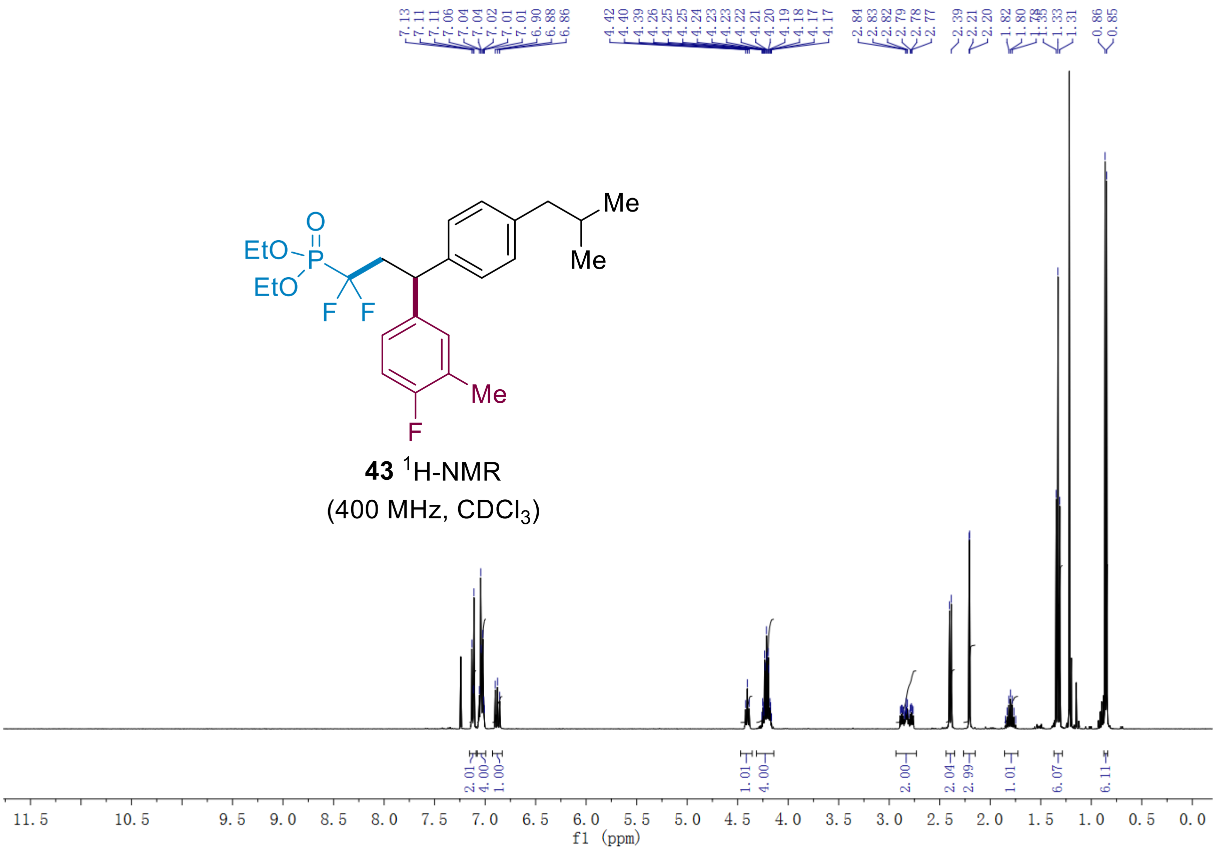

EtO
EtO
P
O
F F
Me
Me
Me
F
43 ^{1}H-NMR
(400 MHz, CDCl$_3$)
f1 (ppm)

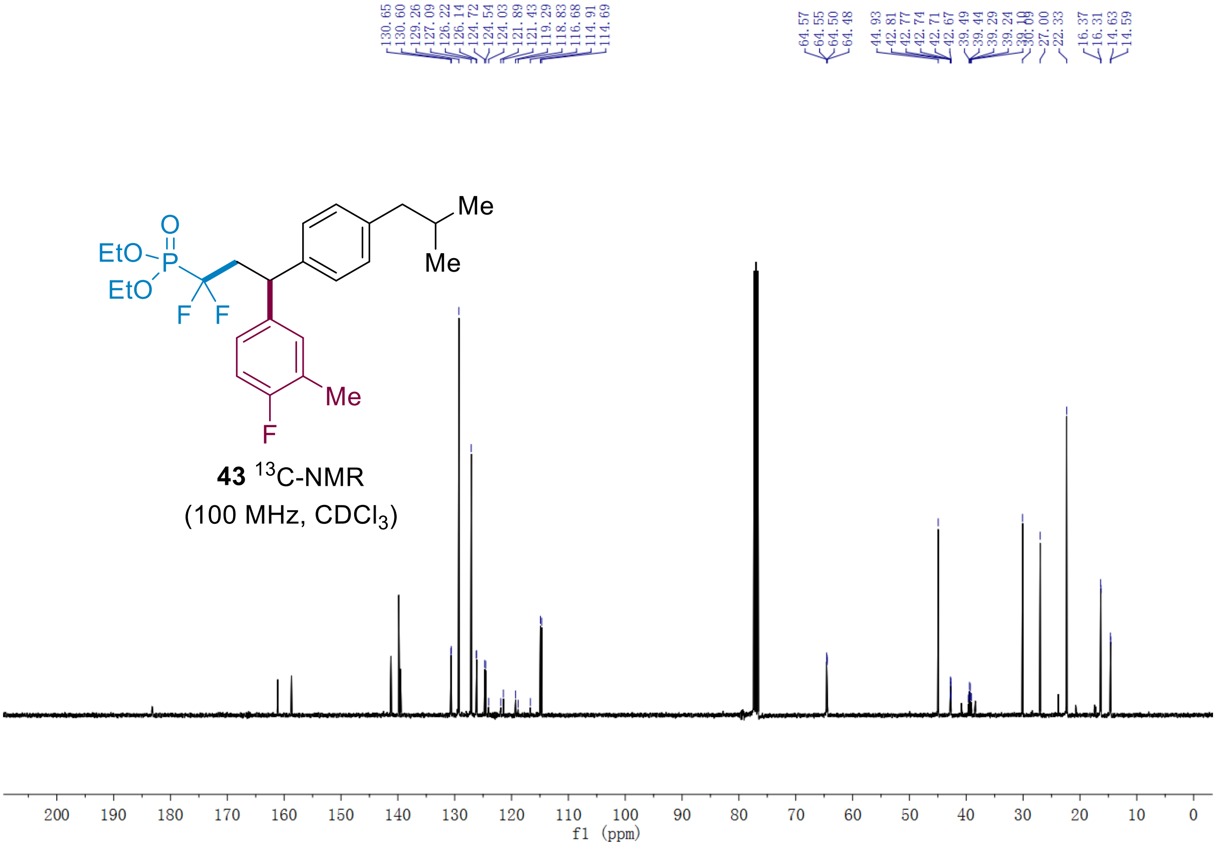
130.65 130.60 129.26 127.09 126.22 126.14 124.72 124.54 124.03 121.89 121.43 119.29 118.83 116.68 114.91 114.69
64.57 64.55 64.50 64.48
44.93 42.81 42.77 42.74 42.71 42.67 39.49 39.44 39.29 39.24 30.10 30.09 27.00 22.33 16.37 16.31 14.63 14.59
EtO
EtO
O
P
F F
Me
Me
Me
F
43 ^{13}C-NMR
(100 MHz, $CDCl_3$)
200 190 180 170 160 150 140 130 120 110 100 90 80 70 60 50 40 30 20 10 0
f1 (ppm)

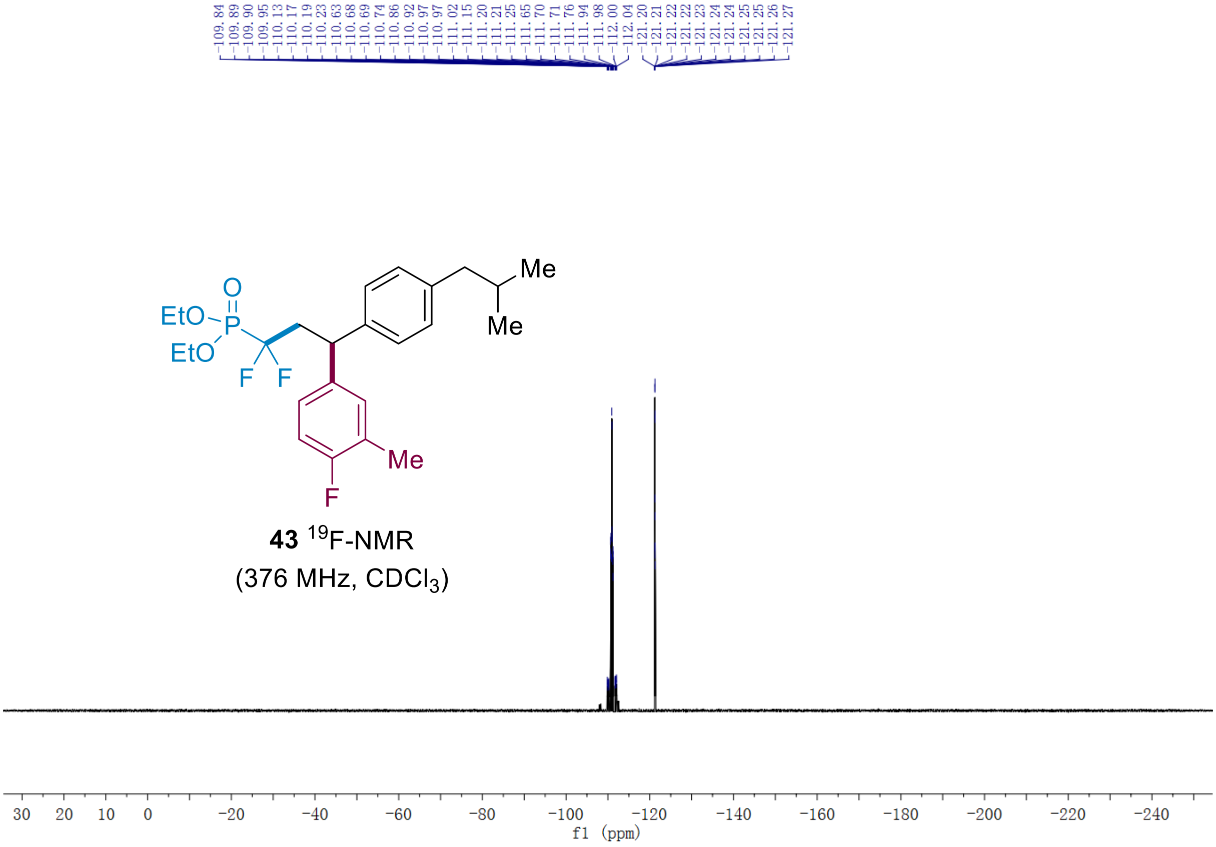
-109.84 -109.89 -109.90 -109.95 -110.13 -110.17 -110.19 -110.23 -110.63 -110.68 -110.69 -110.74 -110.86 -110.92 -110.97 -110.97 -111.02 -111.15 -111.20 -111.21 -111.25 -111.65 -111.70 -111.71 -111.76 -111.94 -111.98 -112.00 -112.04 -121.20 -121.21 -121.22 -121.22 -121.23 -121.24 -121.24 -121.25 -121.25 -121.26 -121.27
EtO
EtO
O
P
F F
Me
Me
Me
F
43 ^{19}F-NMR
(376 MHz, $CDCl_3$)
30 20 10 0 -20 -40 -60 -80 -100 -120 -140 -160 -180 -200 -220 -240
f1 (ppm)

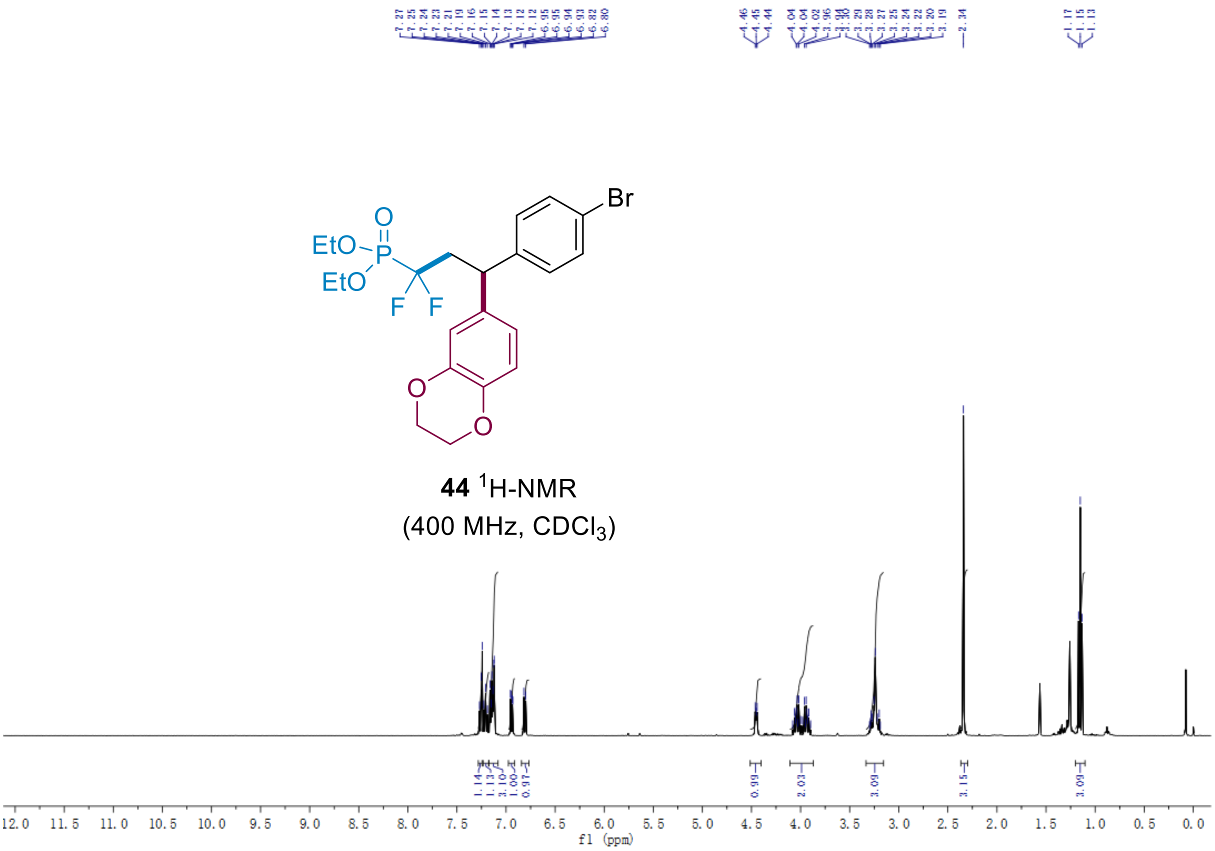

44 ^{1}H-NMR
(400 MHz, $CDCl_3$)

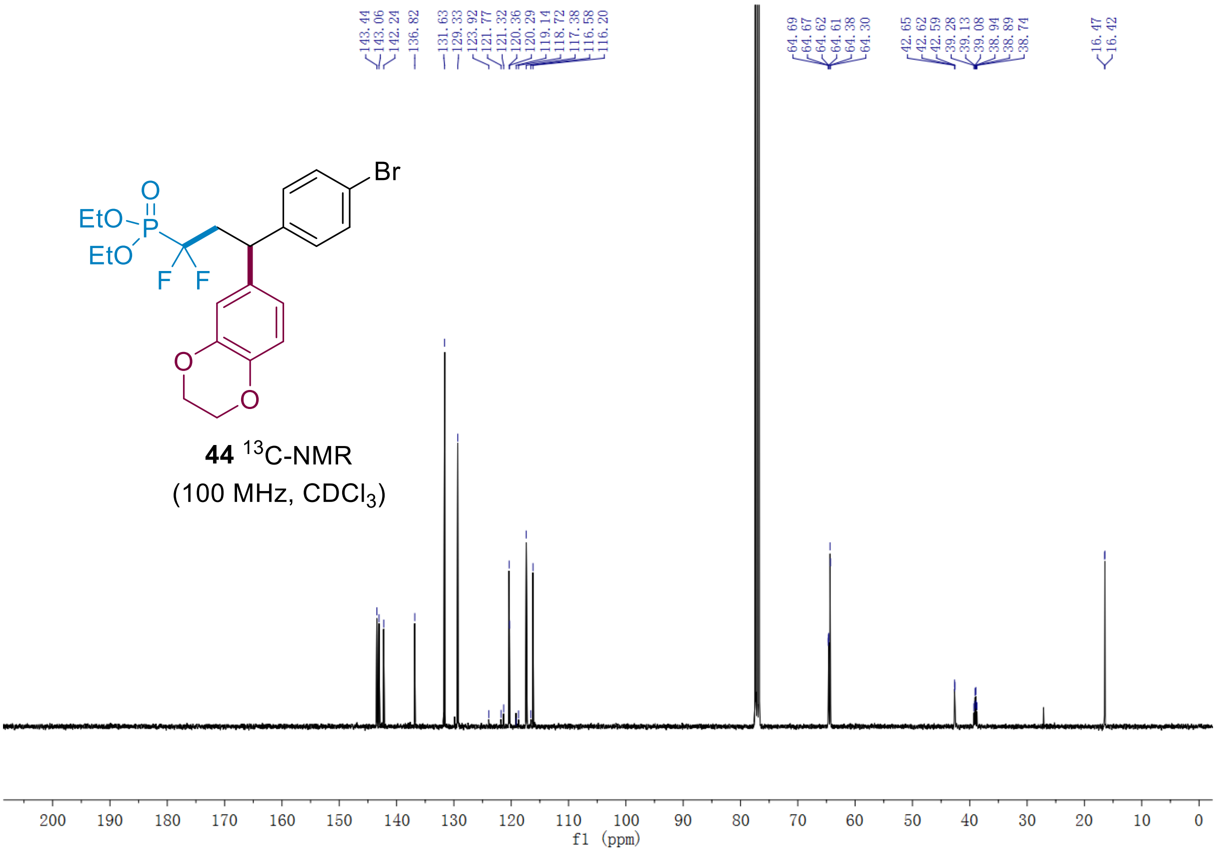

44 ^{13}C-NMR
(100 MHz, $CDCl_3$)

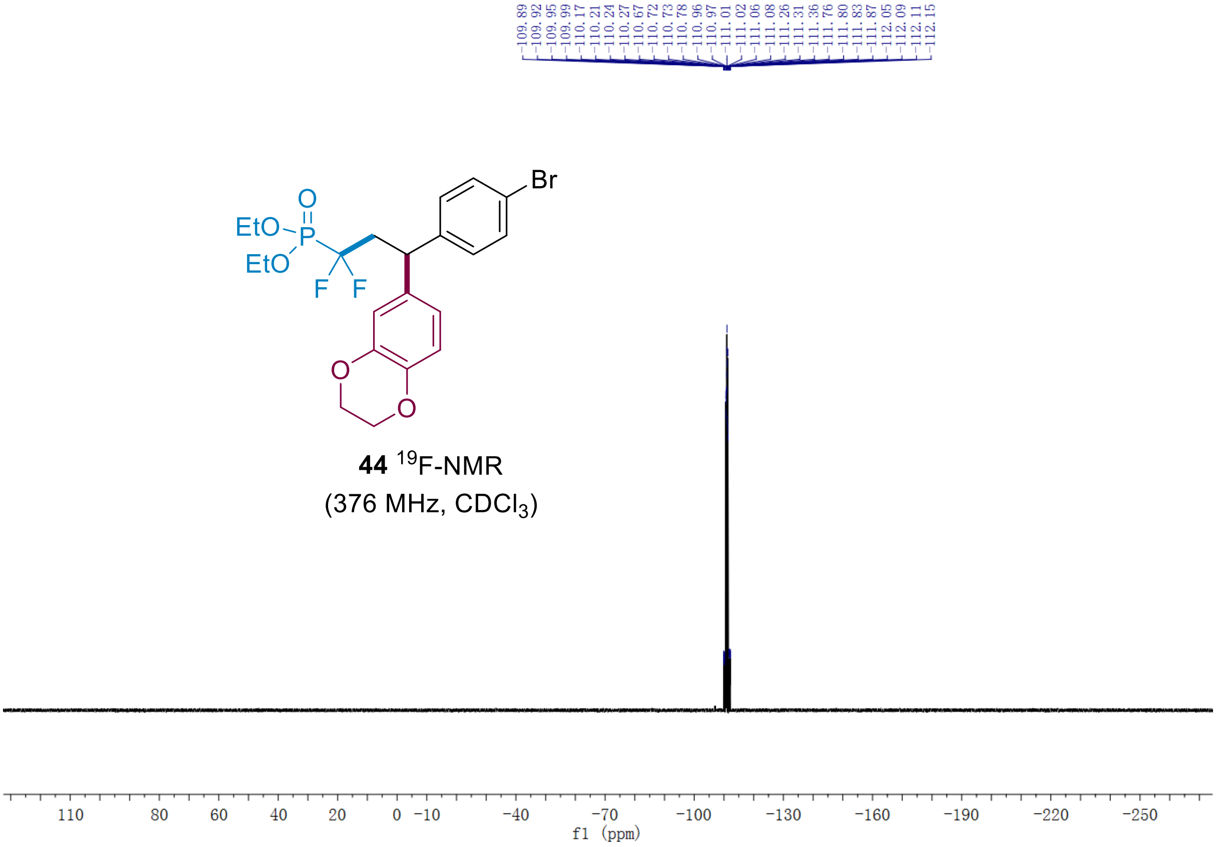

44 ^{19}F-NMR
(376 MHz, $CDCl_3$)

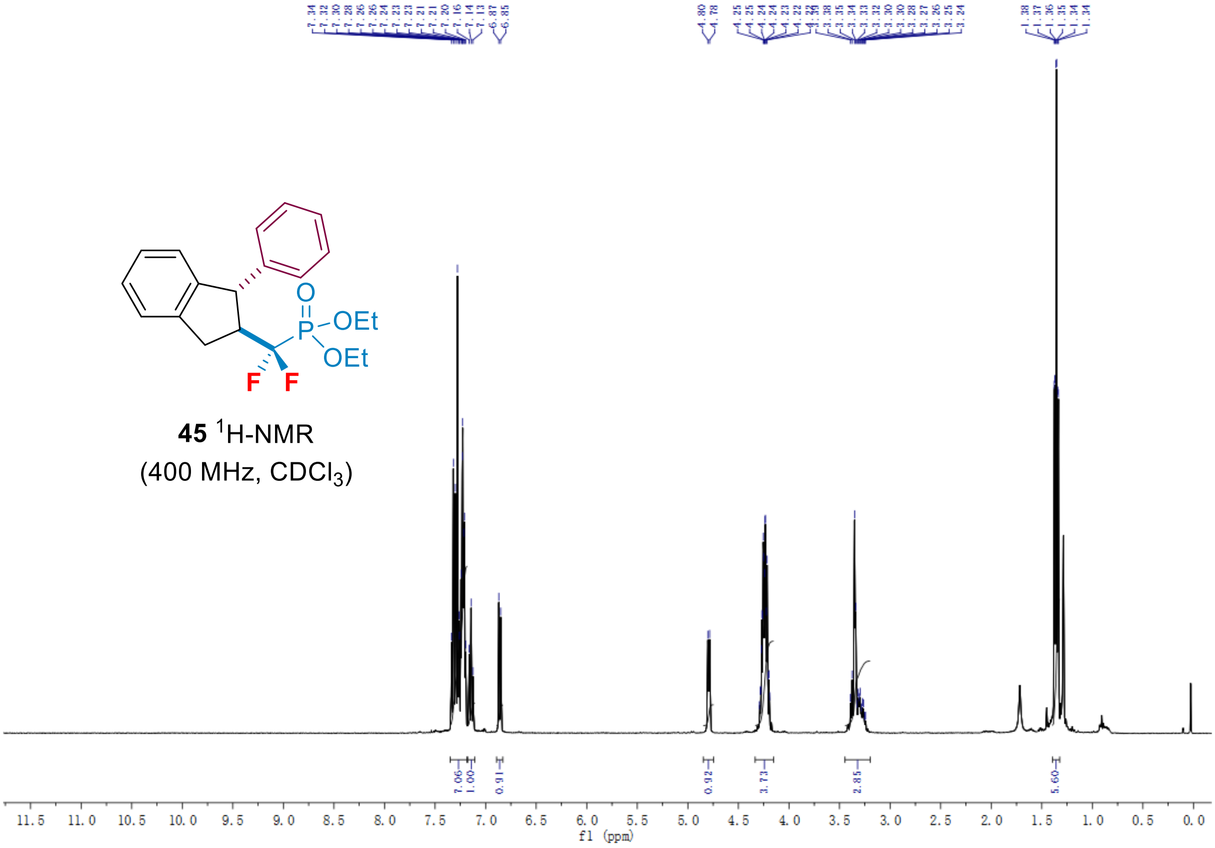

45 ^{1}H-NMR
(400 MHz, $CDCl_3$)

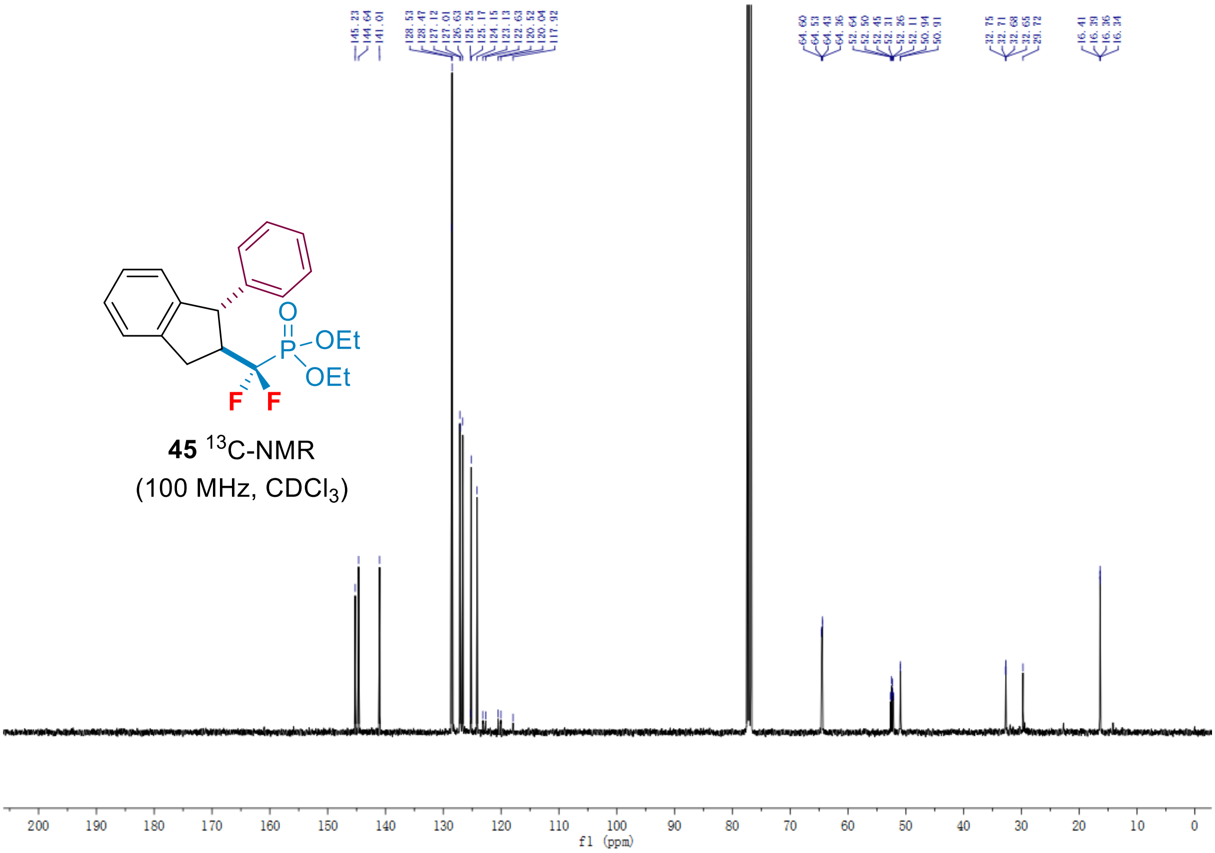
145.23
144.64
141.01
128.53
128.47
127.12
127.01
126.63
125.25
125.17
124.15
123.13
122.63
120.52
120.04
117.92
64.60
64.53
64.43
64.36
52.64
52.50
52.45
52.31
52.26
52.11
50.94
50.91
32.75
32.71
32.68
32.65
29.72
16.41
16.39
16.36
16.34
OEt
OEt
O
P
F
F
45 13C-NMR
(100 MHz, CDCl3)
f1 (ppm)

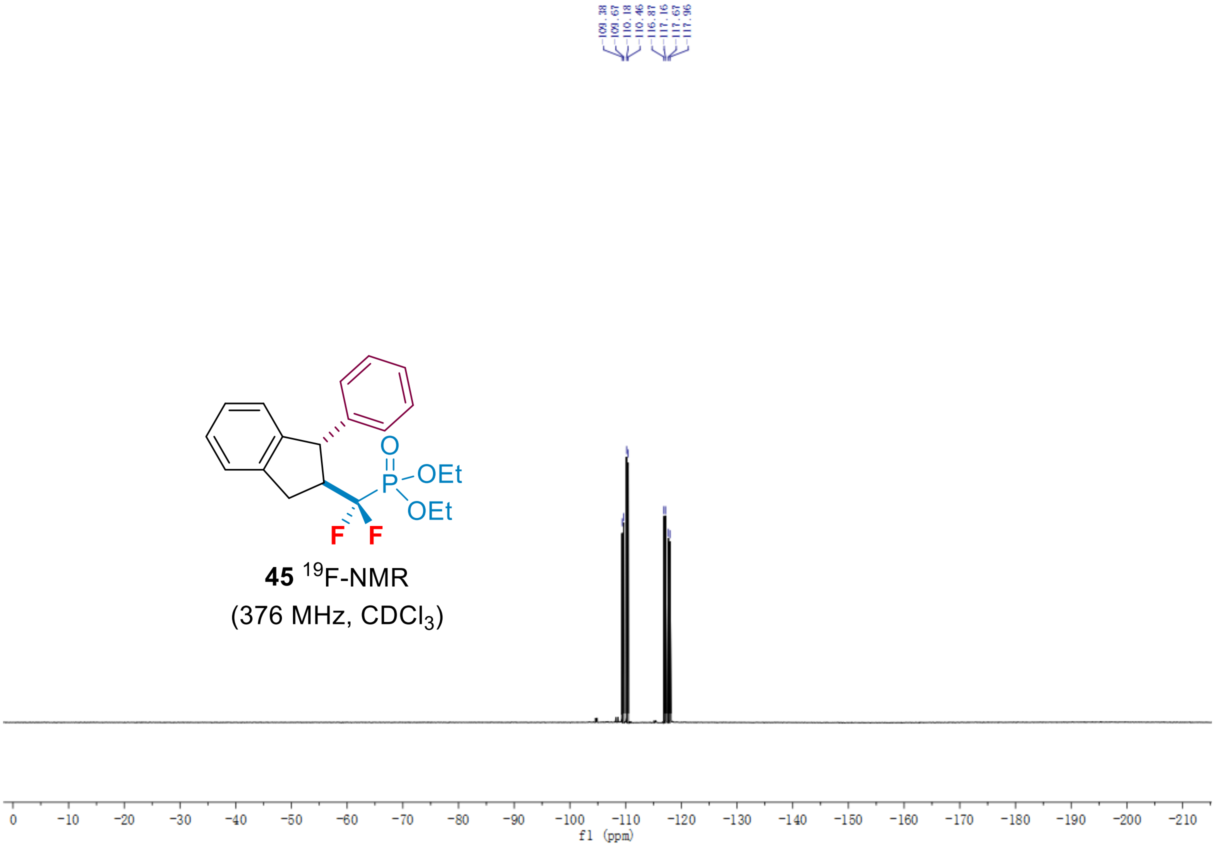
-109.38
-109.67
-110.18
-110.46
-116.87
-117.16
-117.67
-117.96
O
P
OEt
OEt
F
F
45 19F-NMR
(376 MHz, CDCl3)
f1 (ppm)

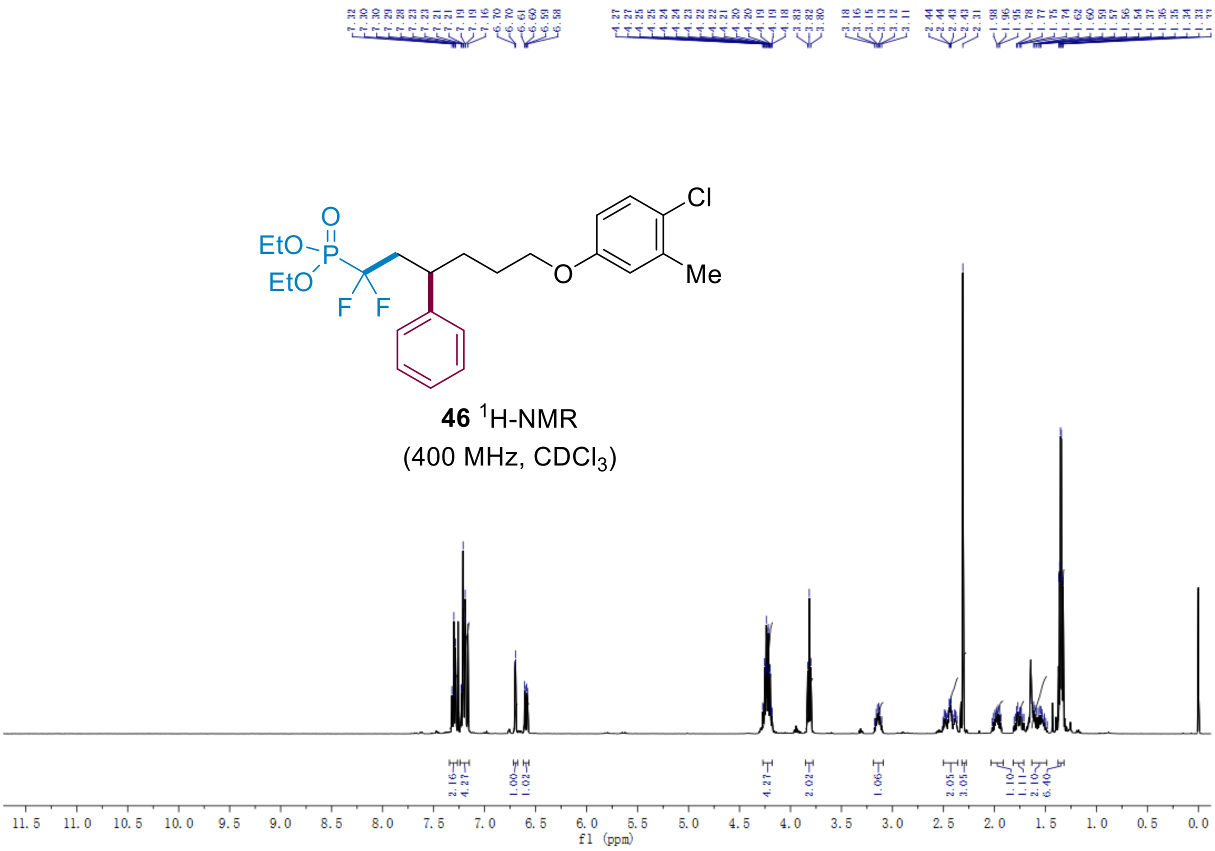
Cl
Me
O
EtO
EtO
F F
46 1H-NMR
(400 MHz, CDCl3)
f1 (ppm)

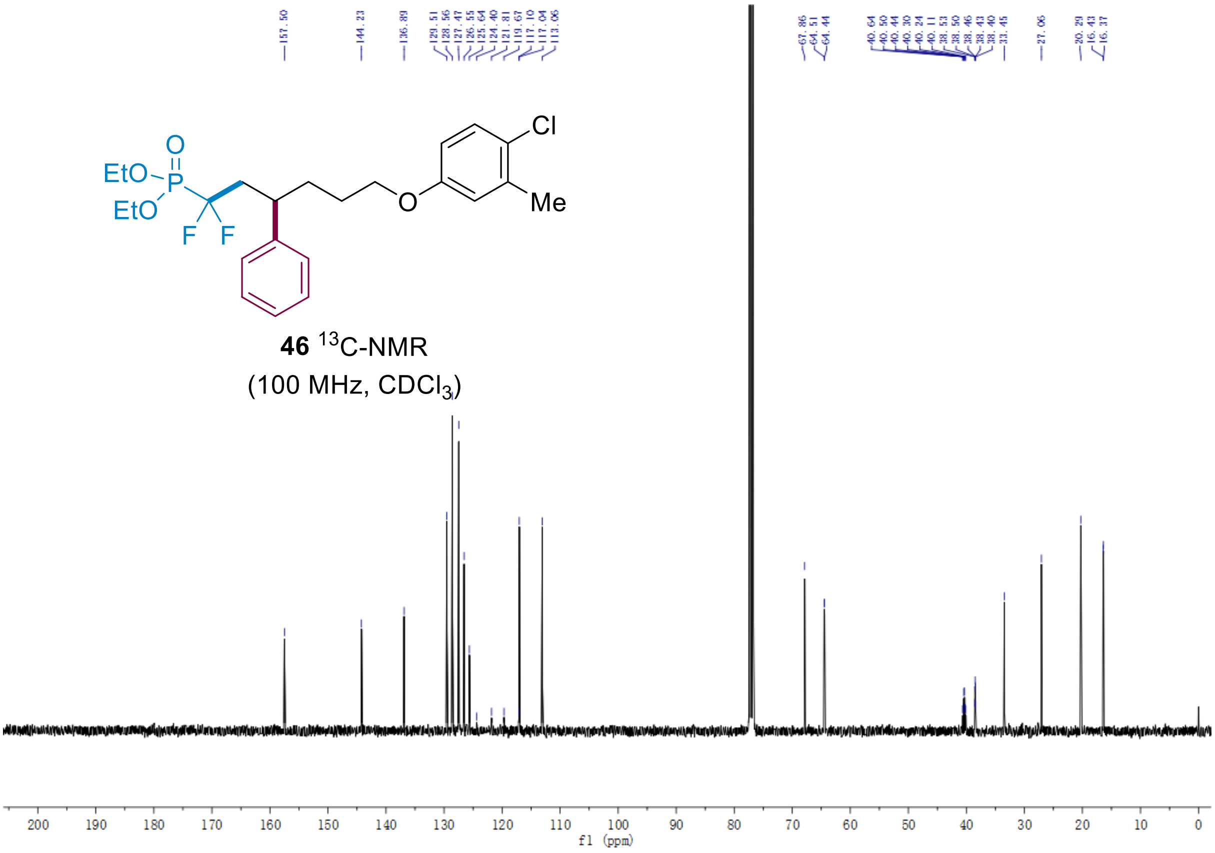
Cl
Me
O
EtO
EtO
F F
46 13C-NMR
(100 MHz, CDCl3)
f1 (ppm)

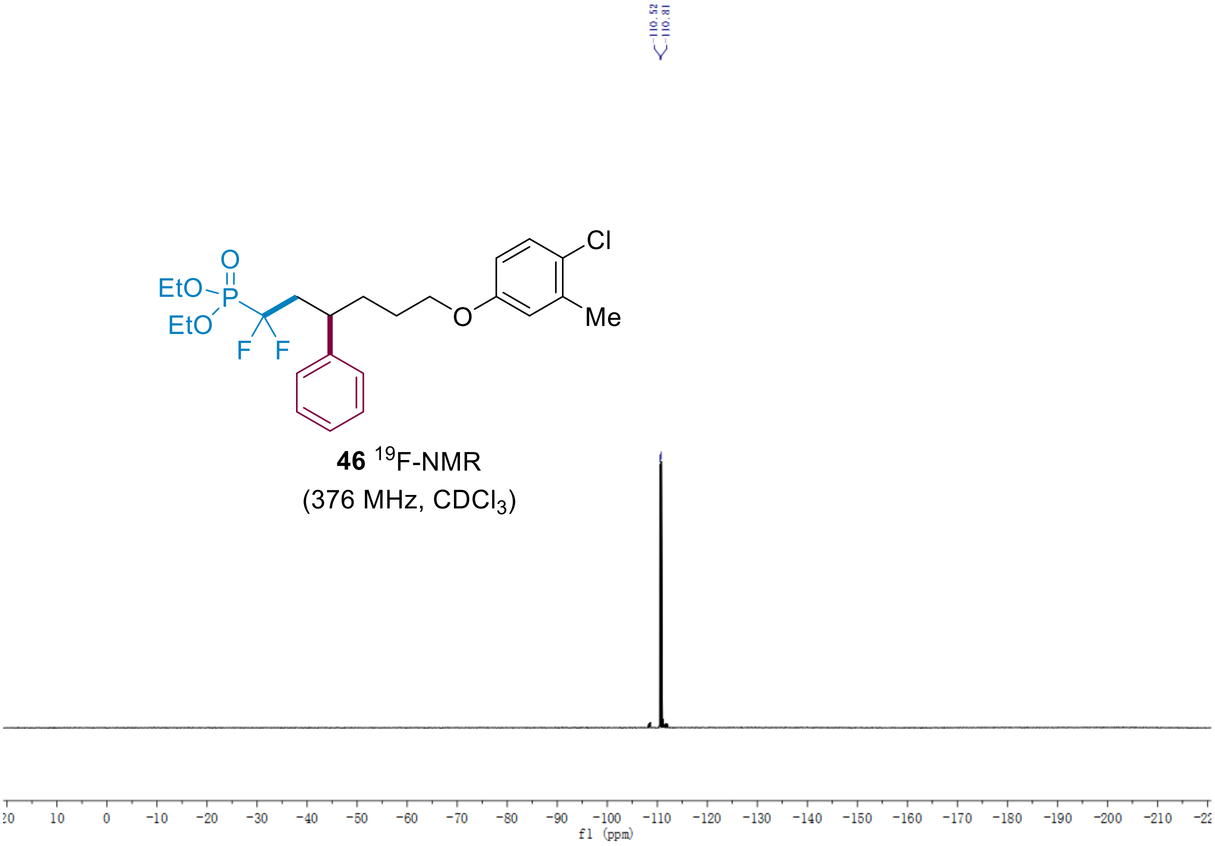

46 ^{19}F-NMR
(376 MHz, $CDCl_3$)

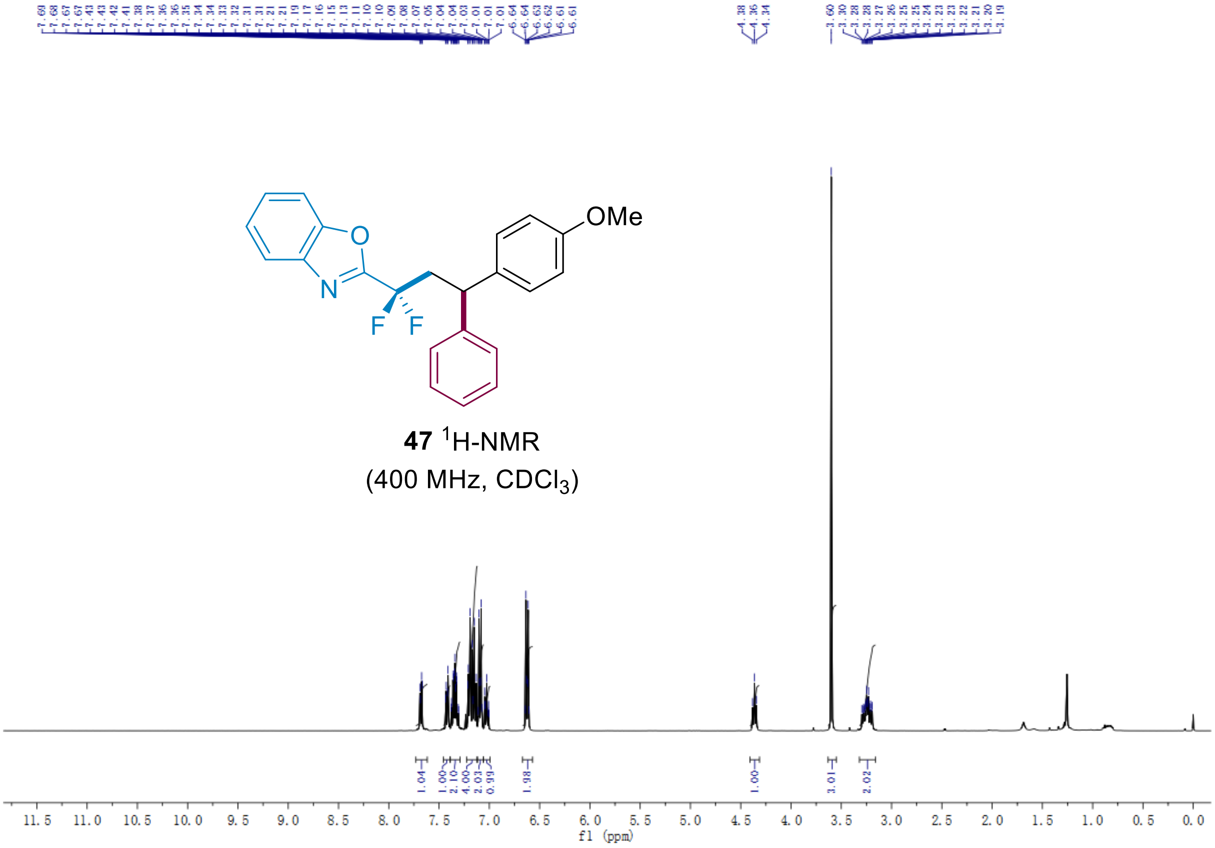

47 ^{1}H-NMR
(400 MHz, $CDCl_3$)

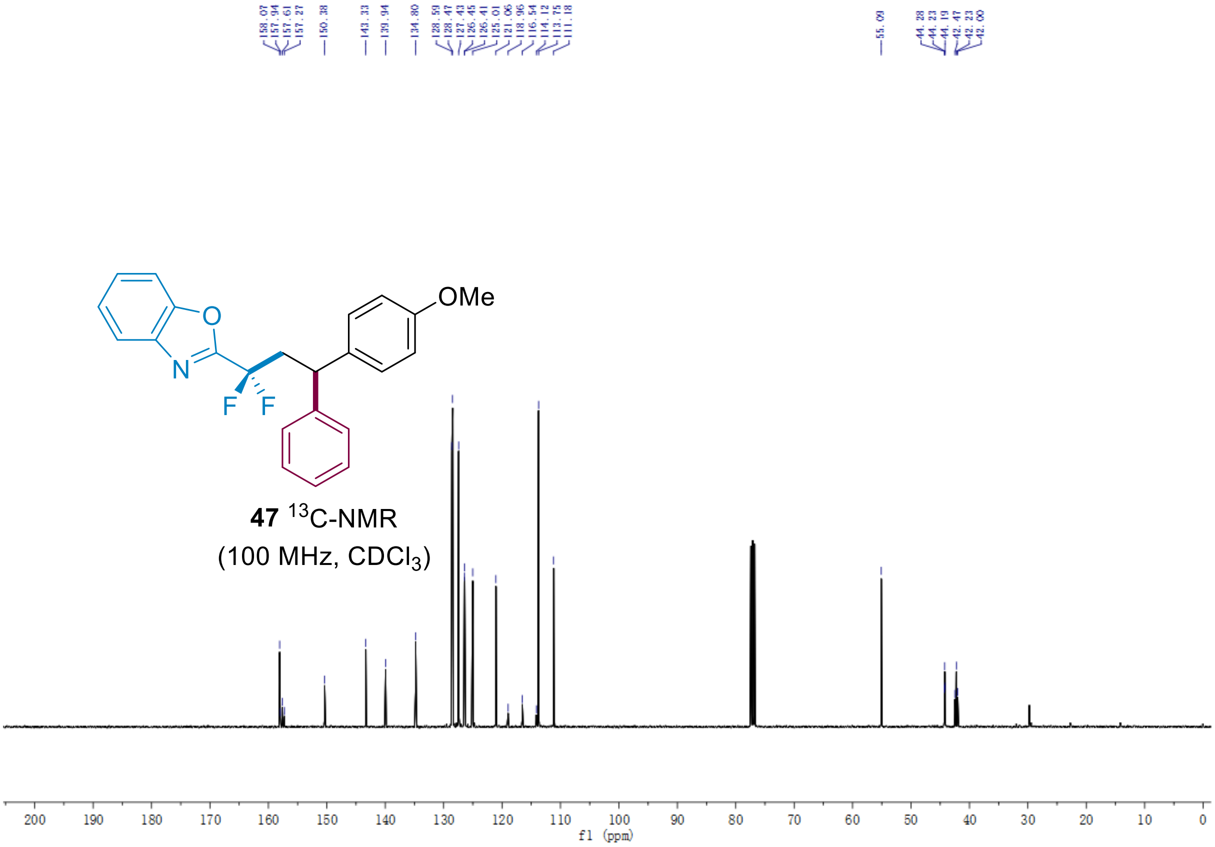
158.07
157.94
157.61
157.27
150.38
143.33
139.94
134.80
128.59
128.47
127.43
126.45
126.41
125.01
121.06
118.96
116.54
114.12
113.75
111.18
55.09
44.28
44.23
44.19
42.47
42.23
42.00
OMe
O
N
F
F
47 13C-NMR
(100 MHz, CDCl3)
f1 (ppm)

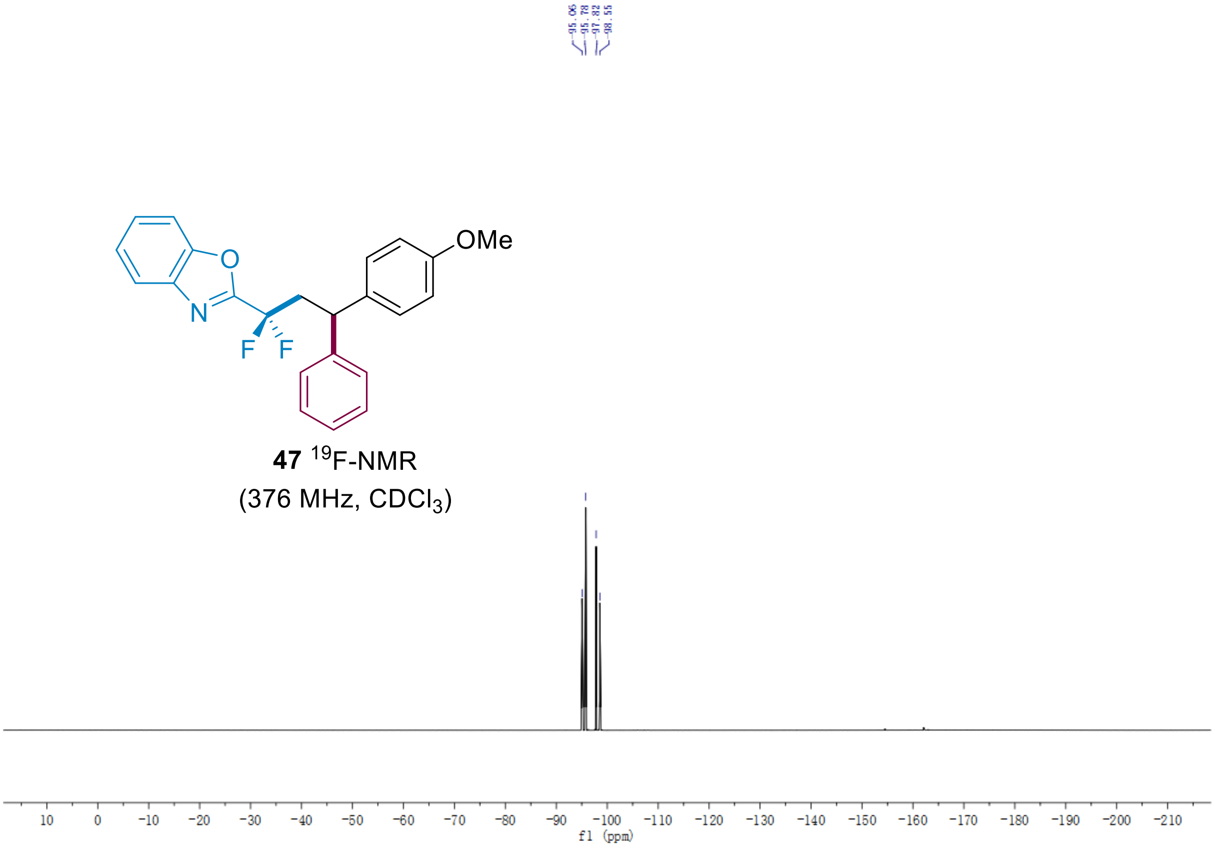
-95.06
-95.78
-97.82
-98.55
OMe
O
N
F
F
47 19F-NMR
(376 MHz, CDCl3)
f1 (ppm)

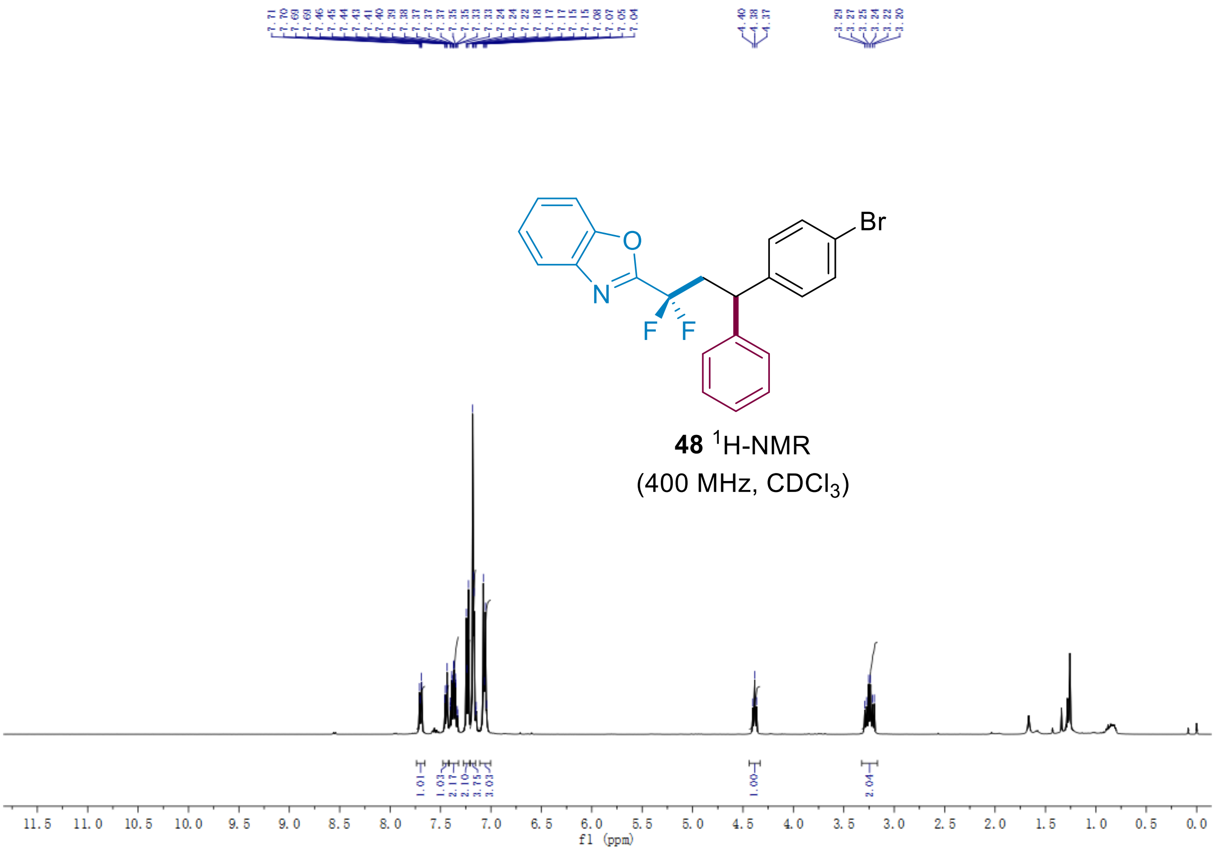

48 ^{1}H-NMR
(400 MHz, $CDCl_3$)

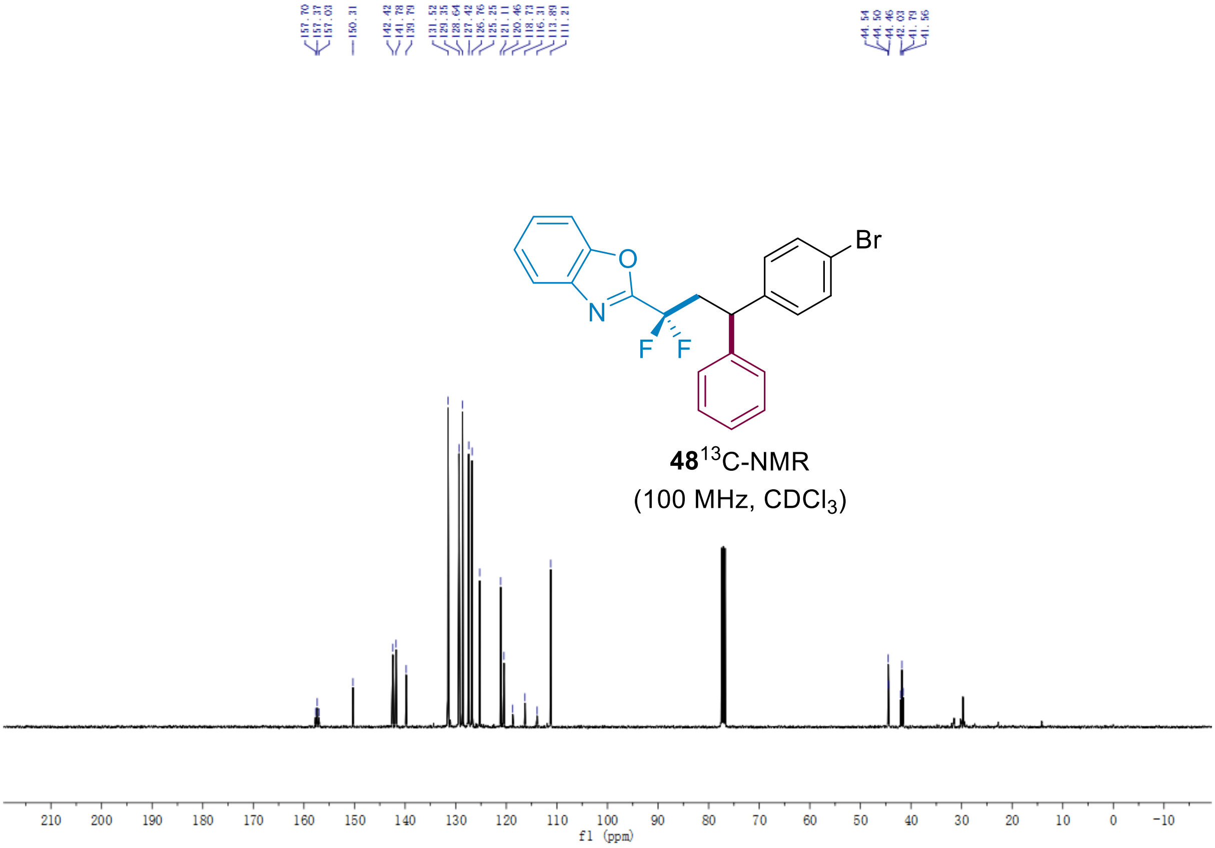

48 ^{13}C-NMR
(100 MHz, $CDCl_3$)

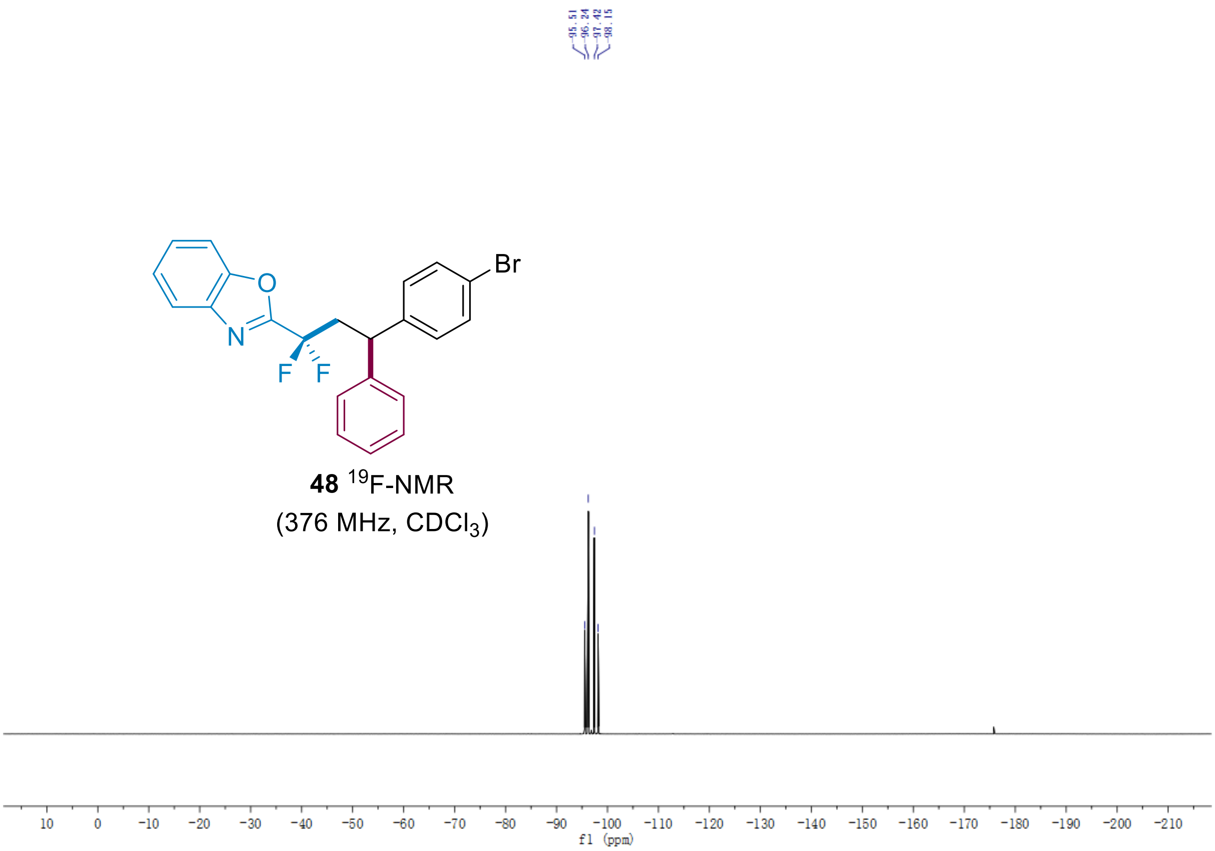

48 ^{19}F-NMR
(376 MHz, $CDCl_3$)

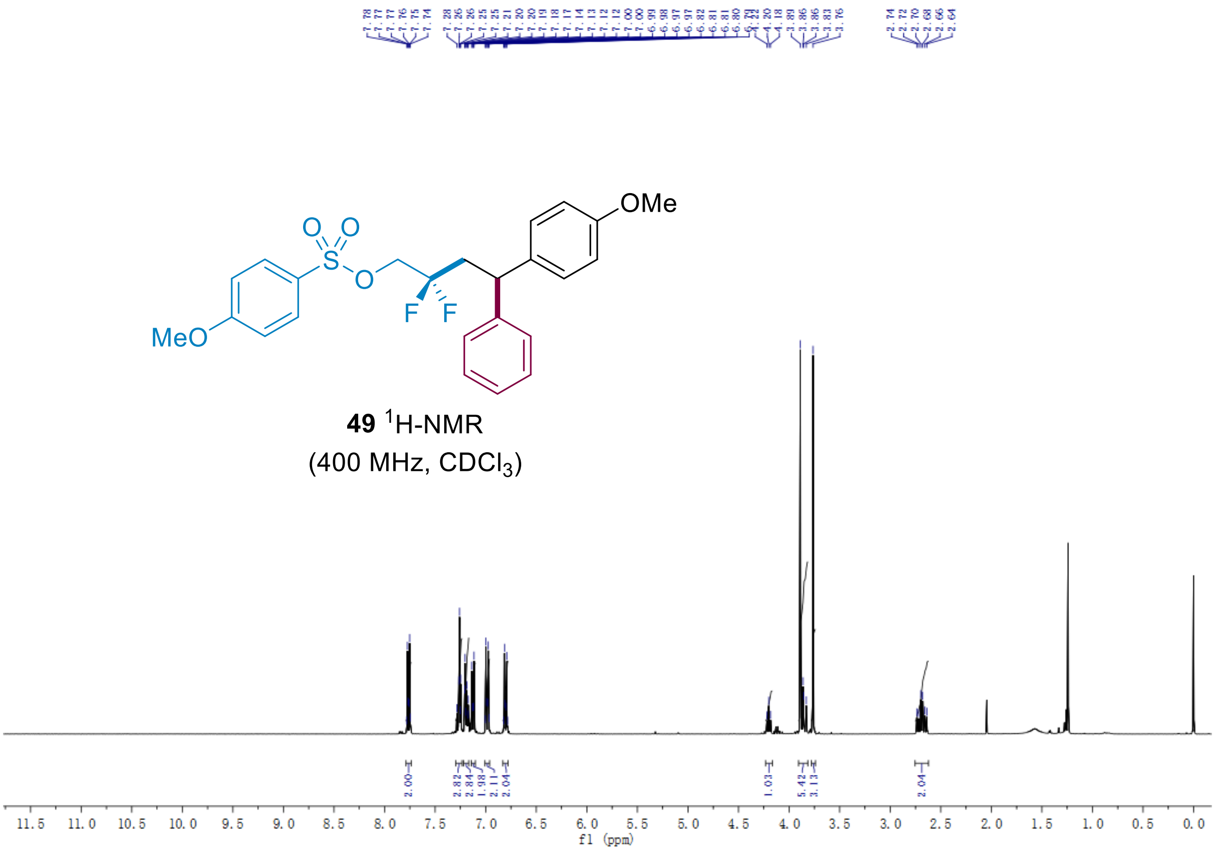

49 ^{1}H-NMR
(400 MHz, $CDCl_3$)

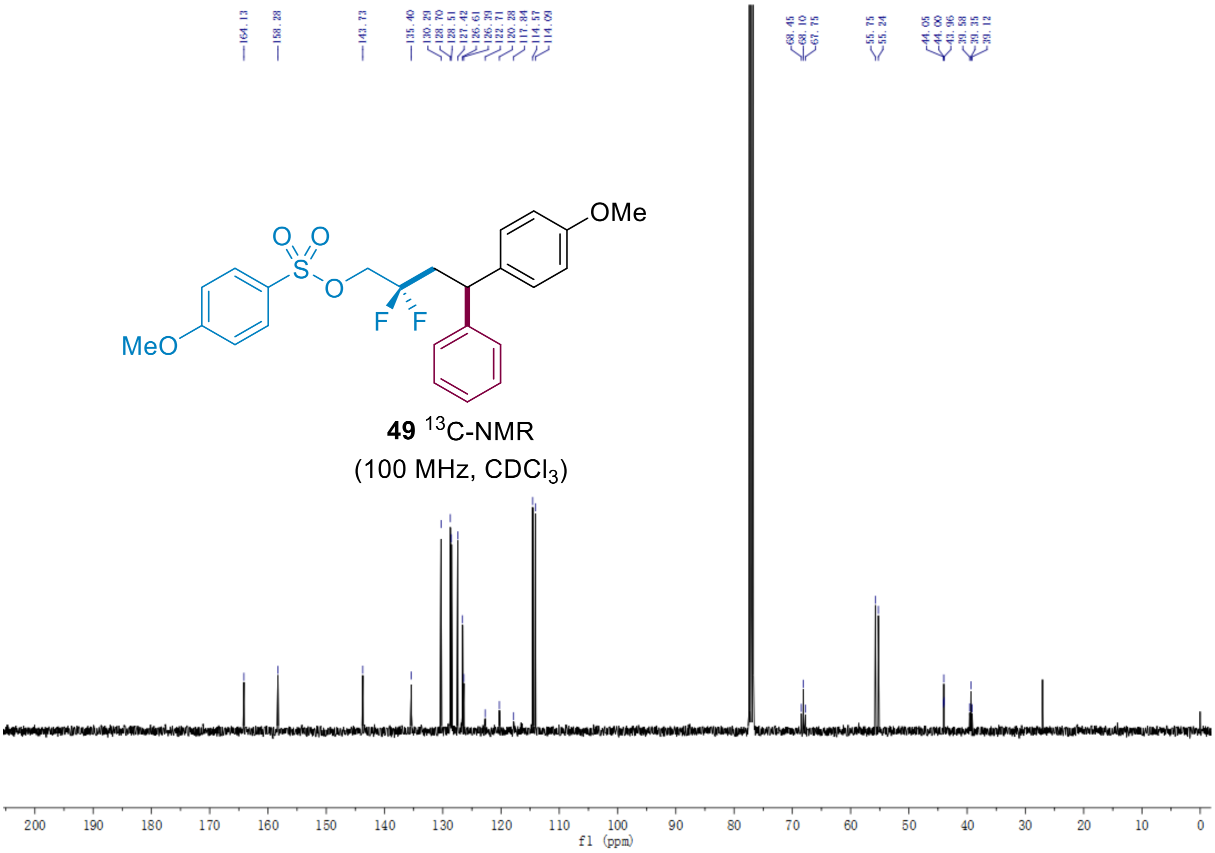
OMe
MeO
F F
49 ^{13}C-NMR
(100 MHz, $CDCl_3$)
f1 (ppm)

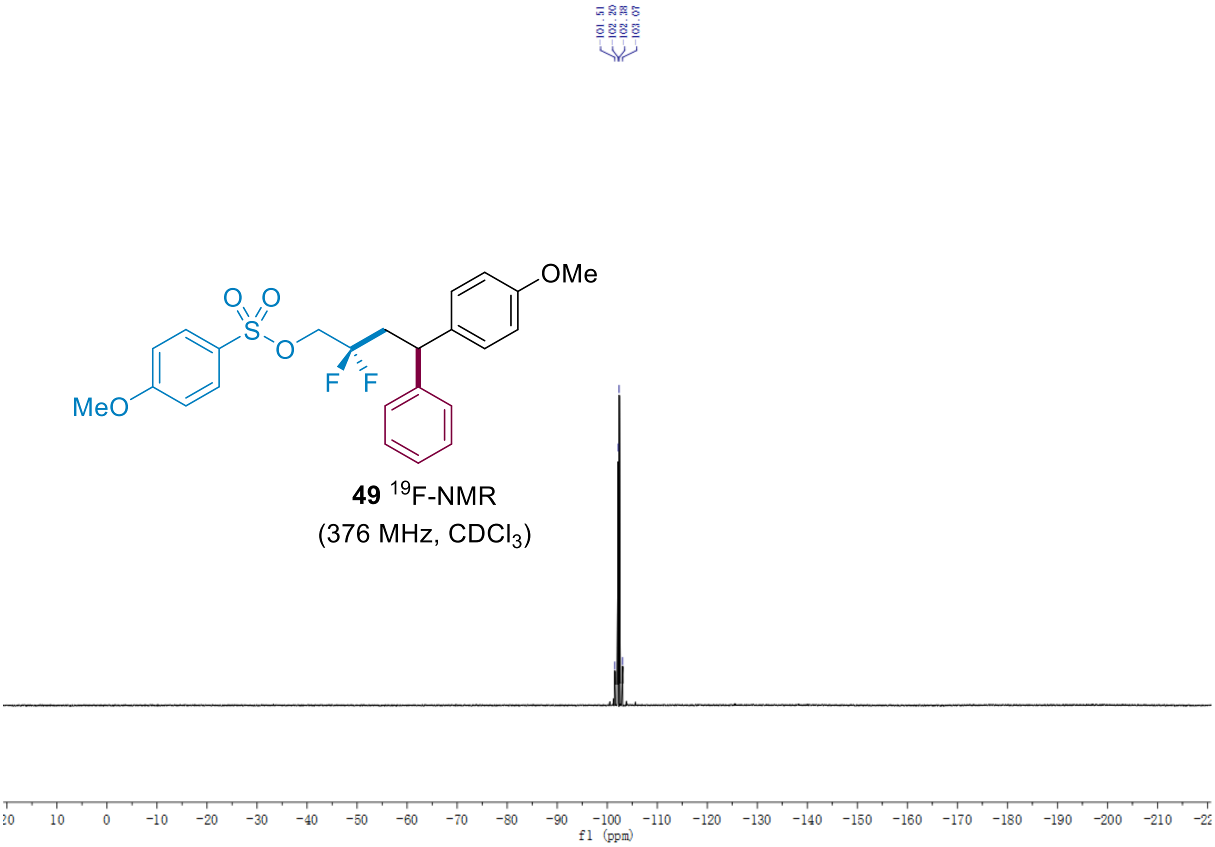
OMe
MeO
F F
49 ^{19}F-NMR
(376 MHz, $CDCl_3$)
f1 (ppm)

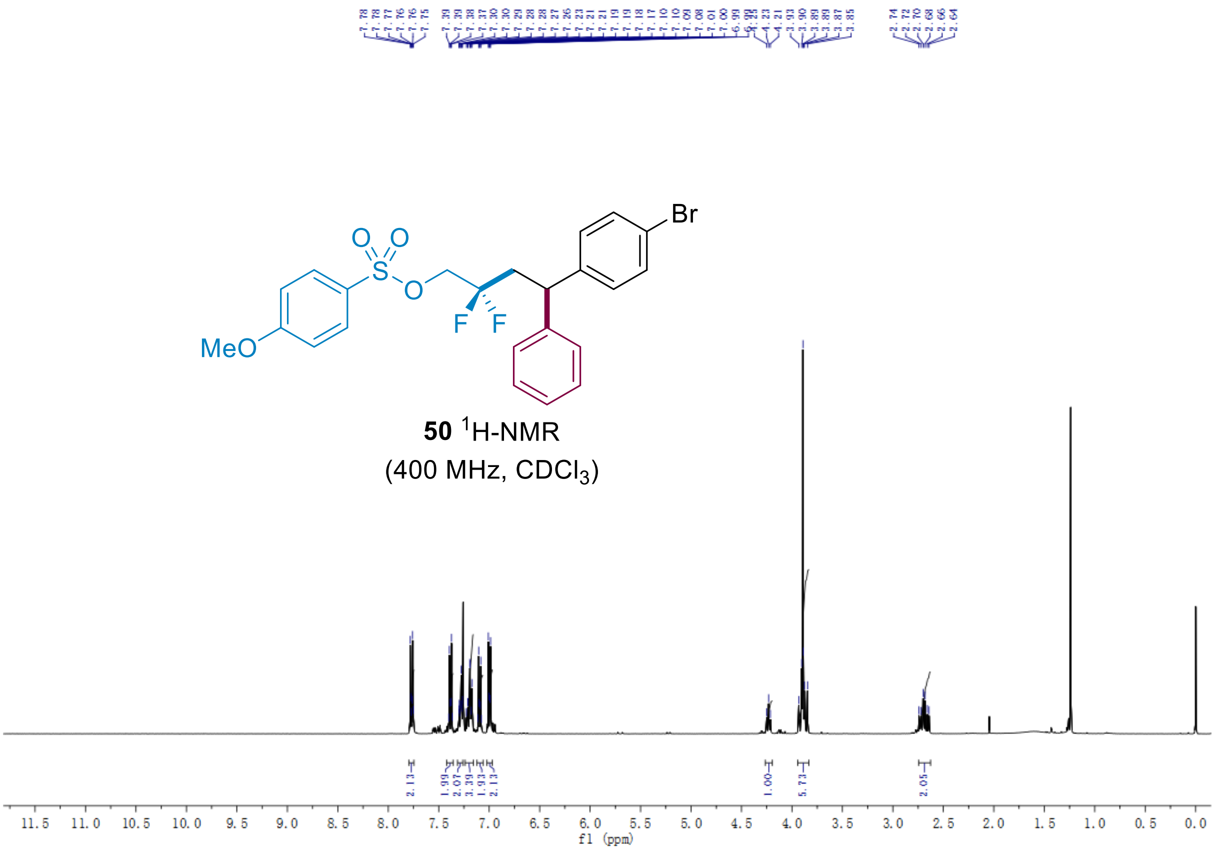

50 ^{1}H-NMR
(400 MHz, $CDCl_3$)

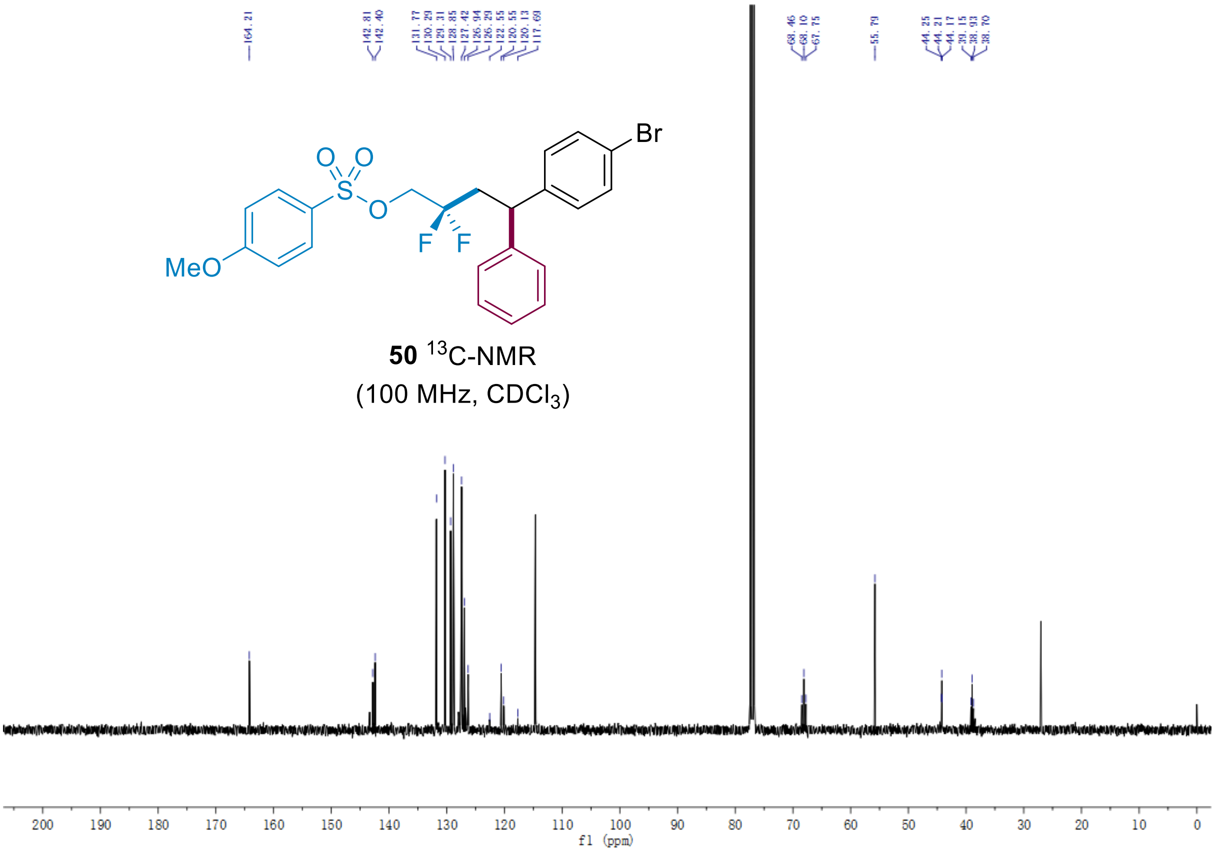

50 ^{13}C-NMR
(100 MHz, $CDCl_3$)

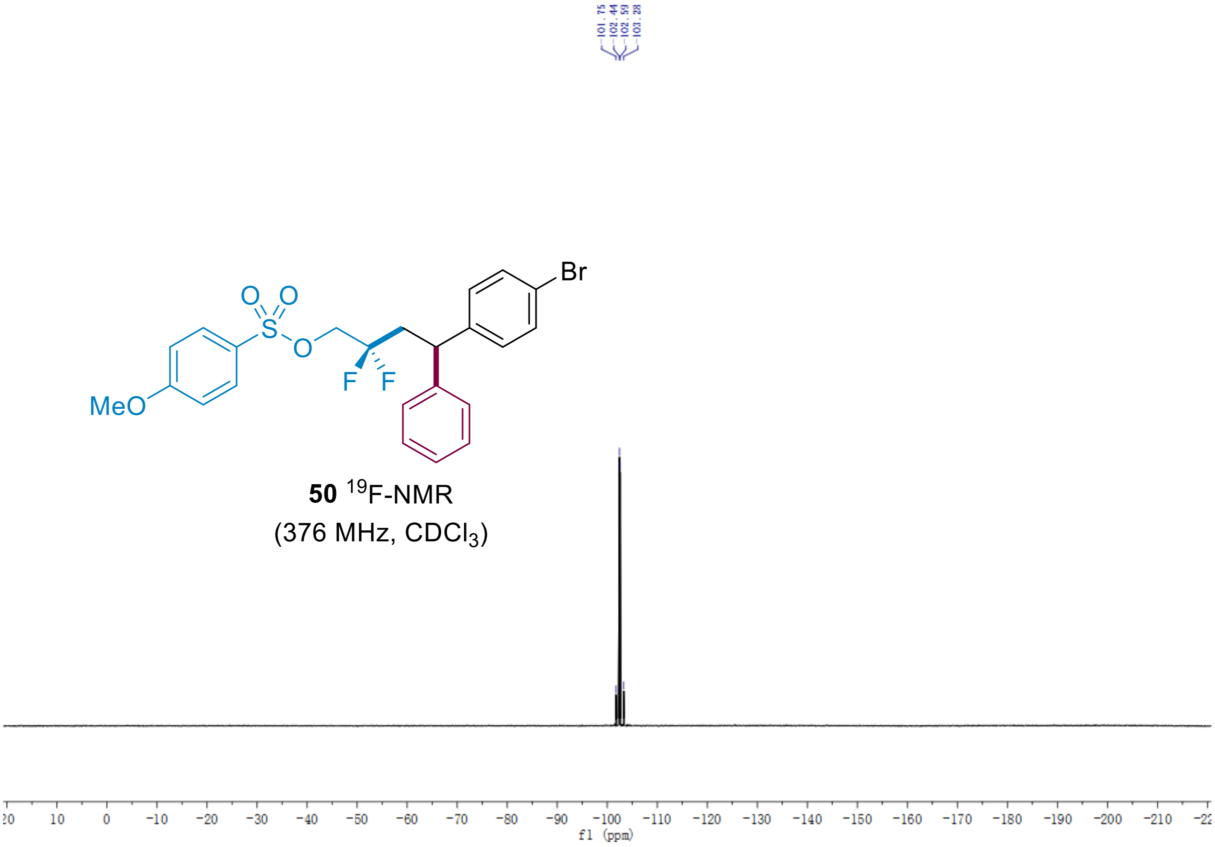

Br
MeO
F F
50 ^{19}F-NMR
(376 MHz, $CDCl_3$)
f1 (ppm)

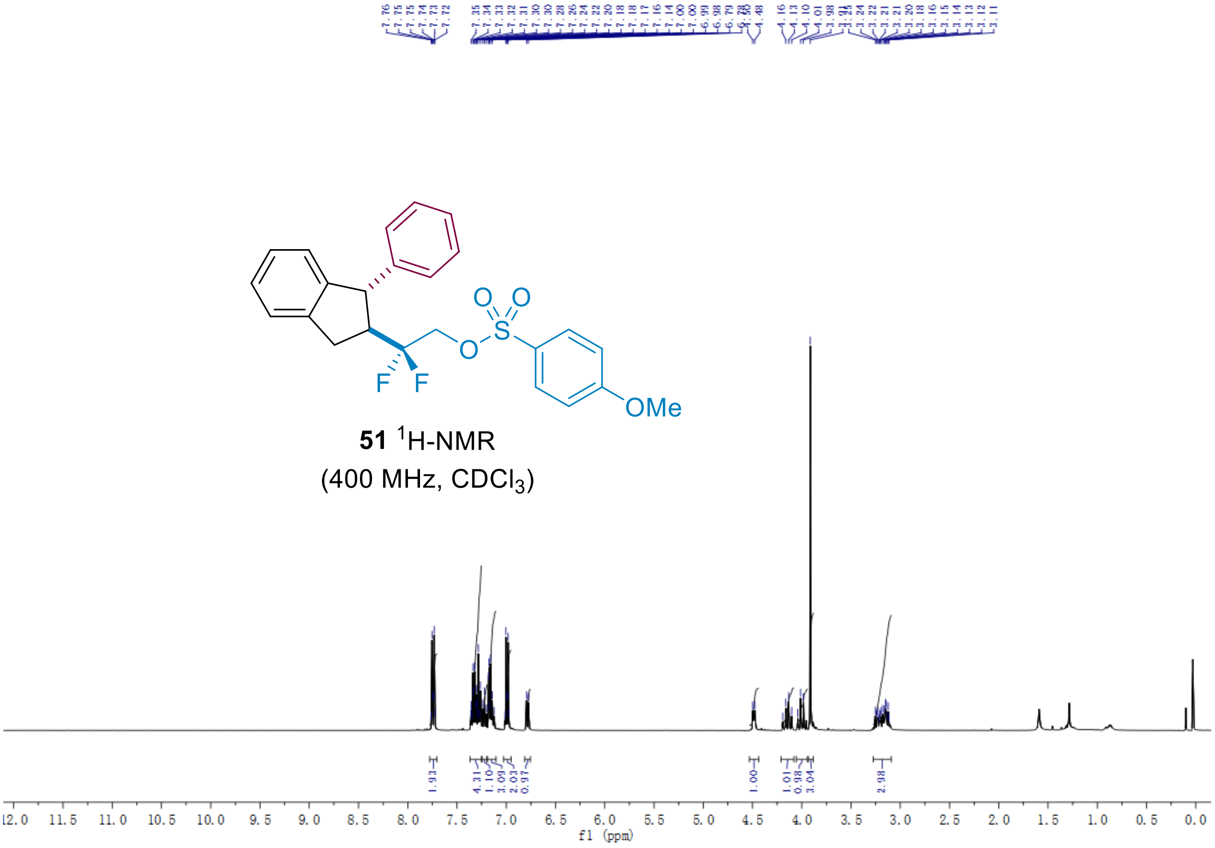

F F
OMe
51 ^{1}H-NMR
(400 MHz, $CDCl_3$)
f1 (ppm)

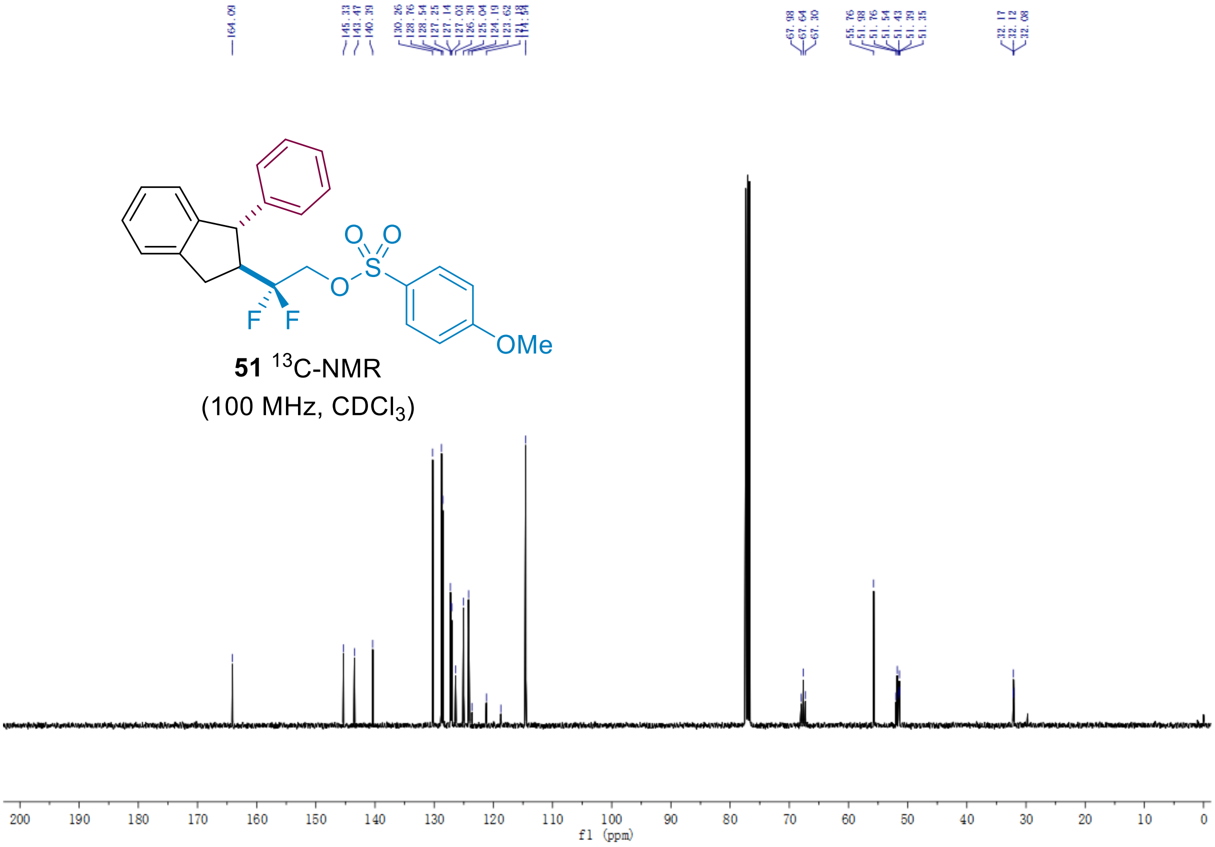
51 ^{13}C-NMR
(100 MHz, $CDCl_3$)
OMe
f1 (ppm)

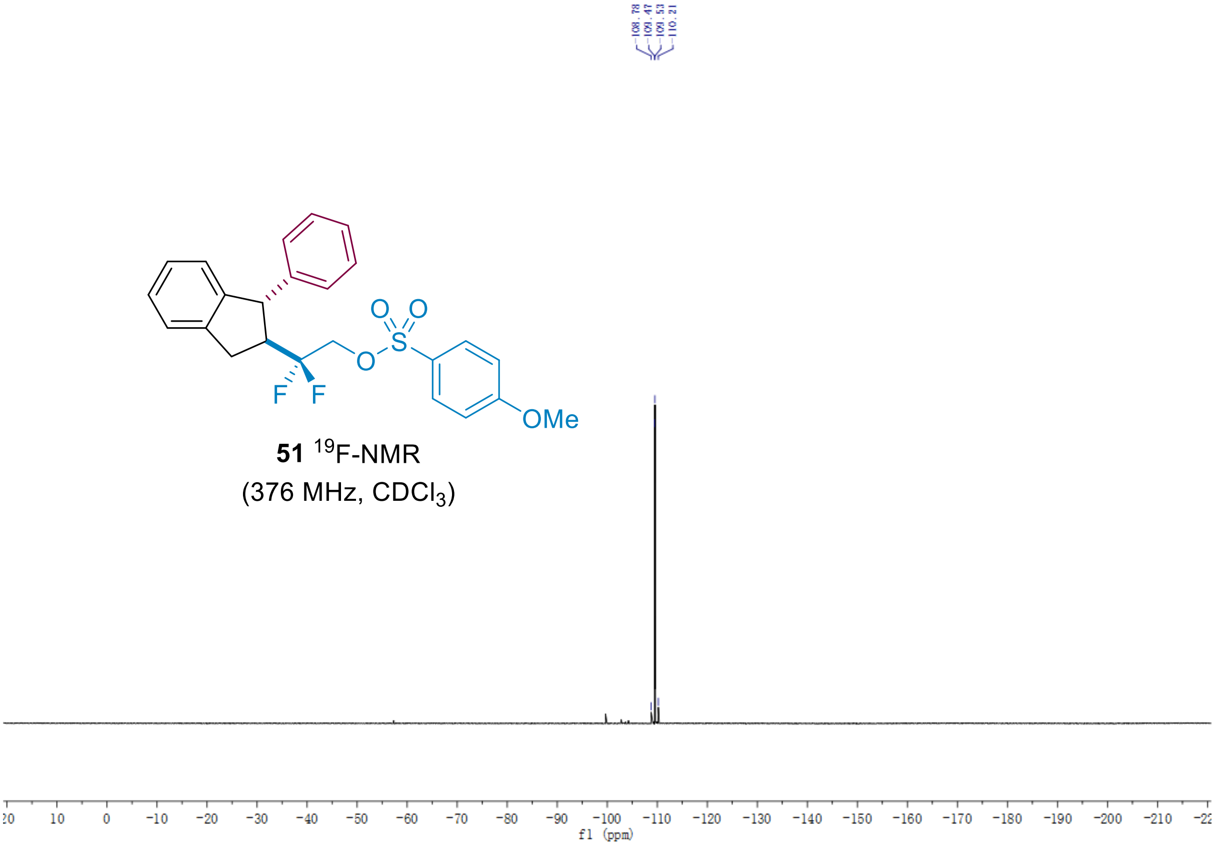
51 ^{19}F-NMR
(376 MHz, $CDCl_3$)
OMe
f1 (ppm)

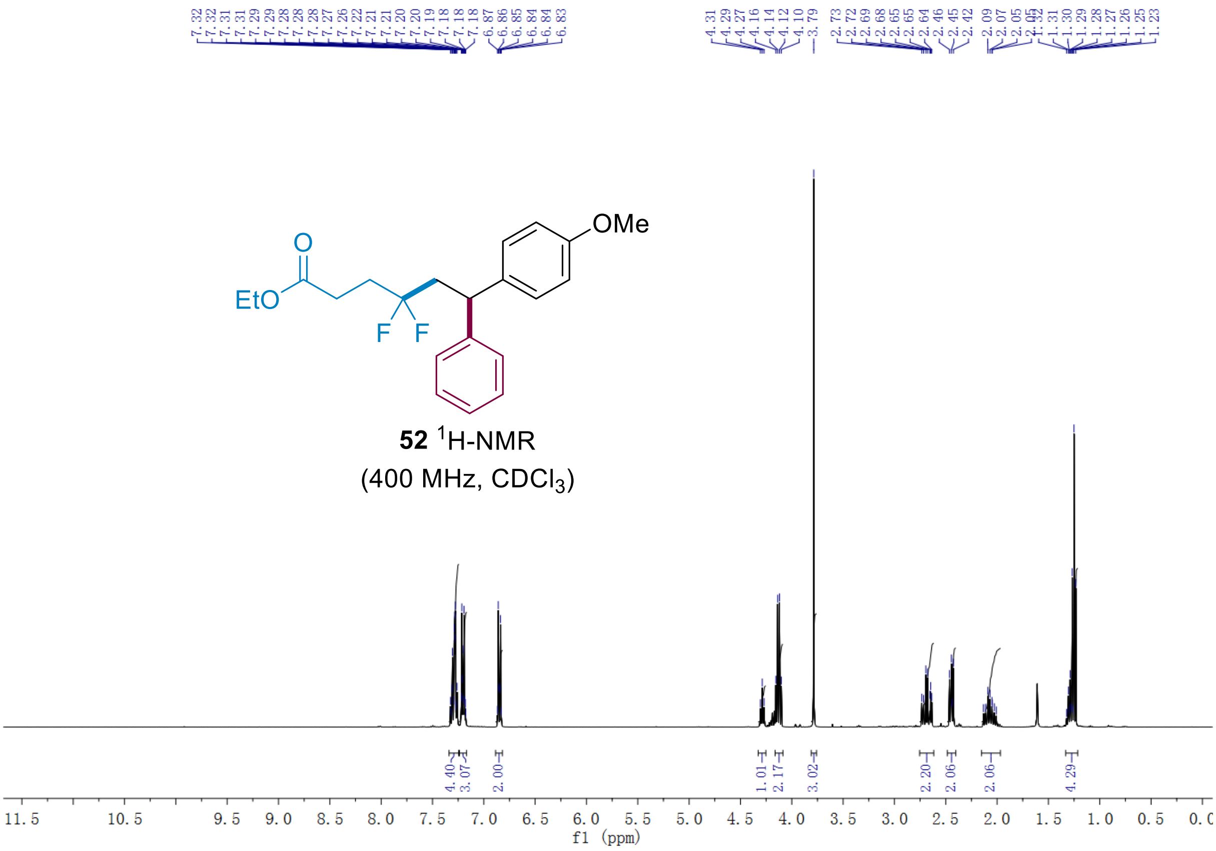

OMe
O
EtO
F F
52 ^{1}H-NMR
(400 MHz, $CDCl_3$)
f1 (ppm)

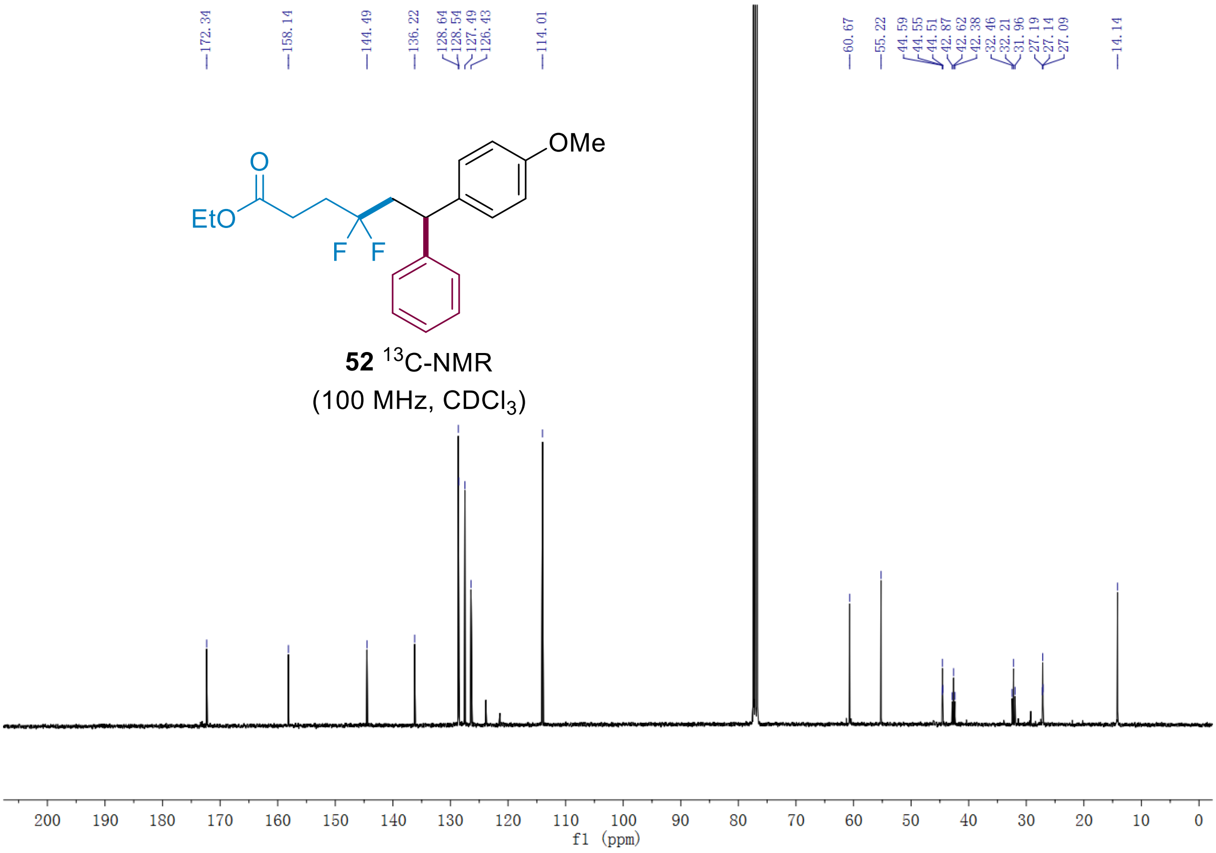

OMe
O
EtO
F F
52 ^{13}C-NMR
(100 MHz, $CDCl_3$)
f1 (ppm)

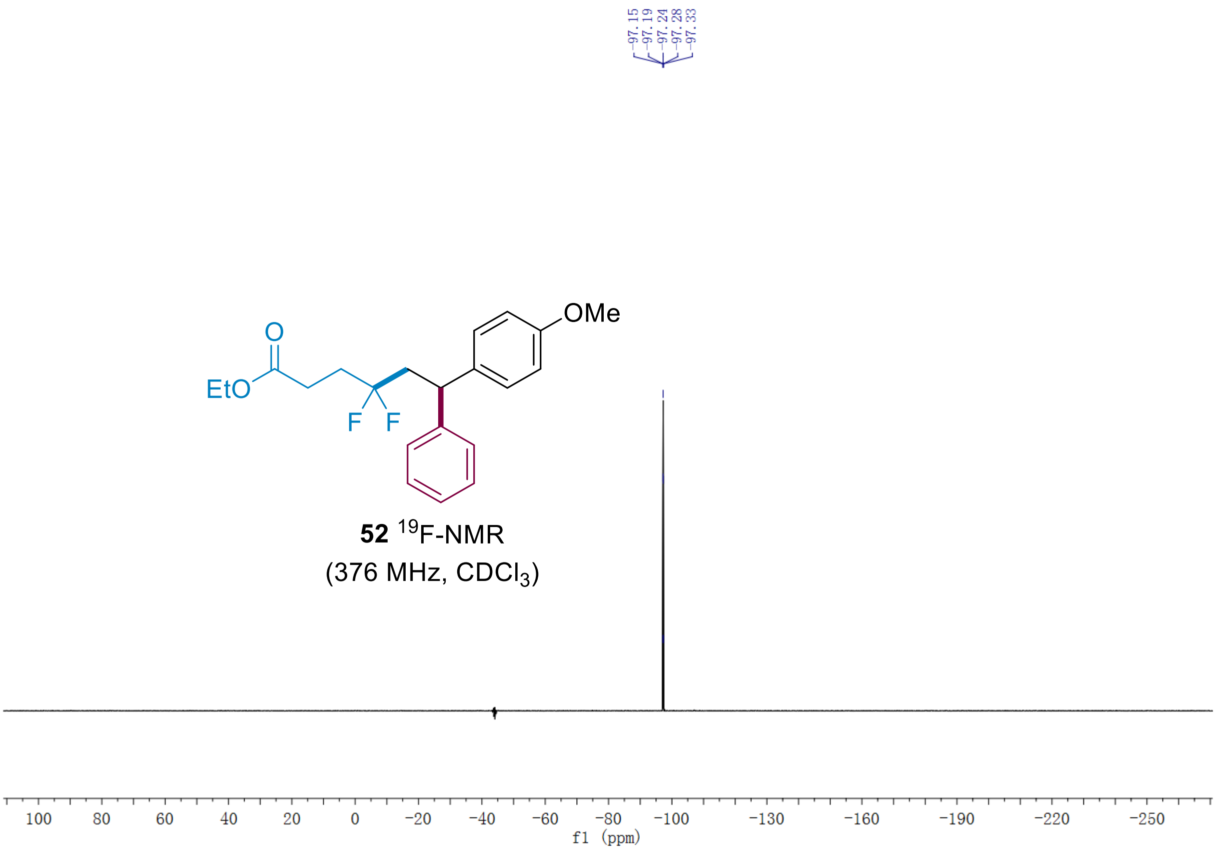

52 ^{19}F-NMR
(376 MHz, $CDCl_3$)

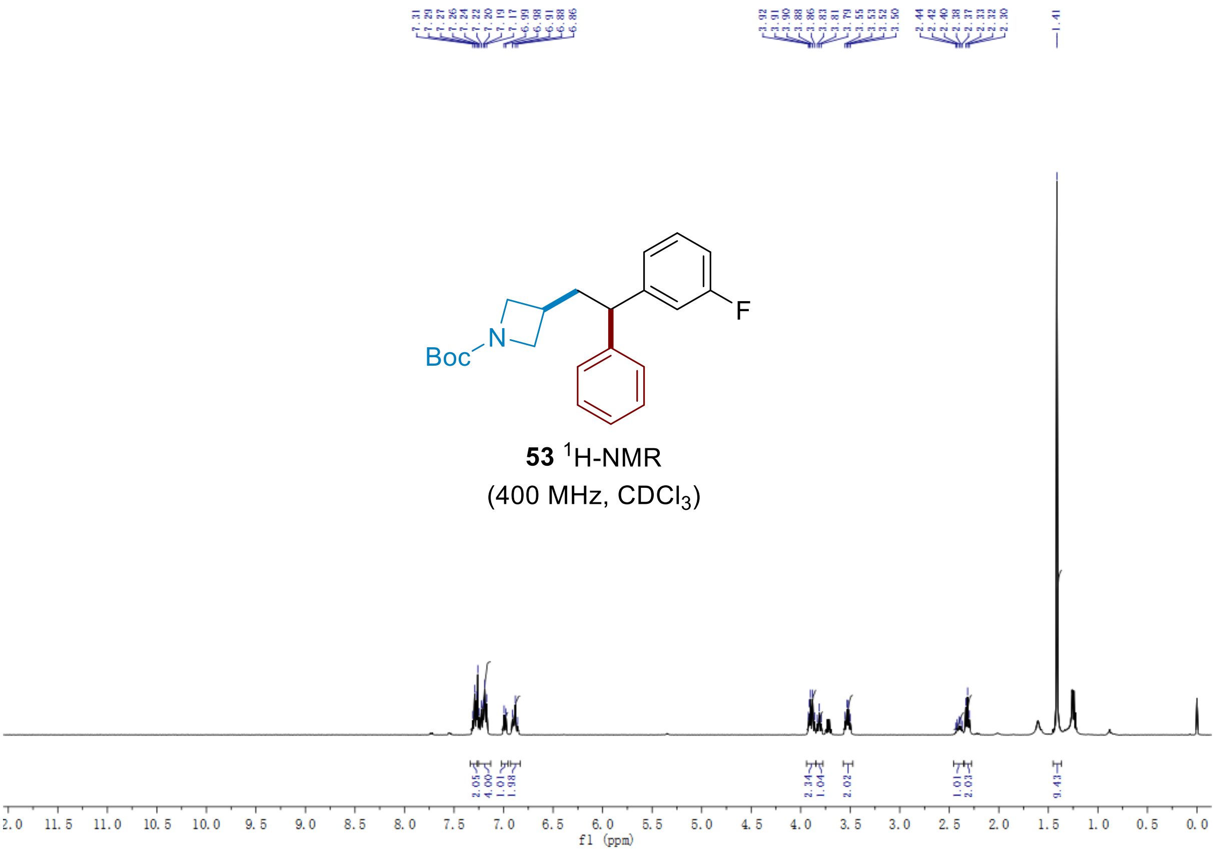

53 ^{1}H-NMR
(400 MHz, $CDCl_3$)

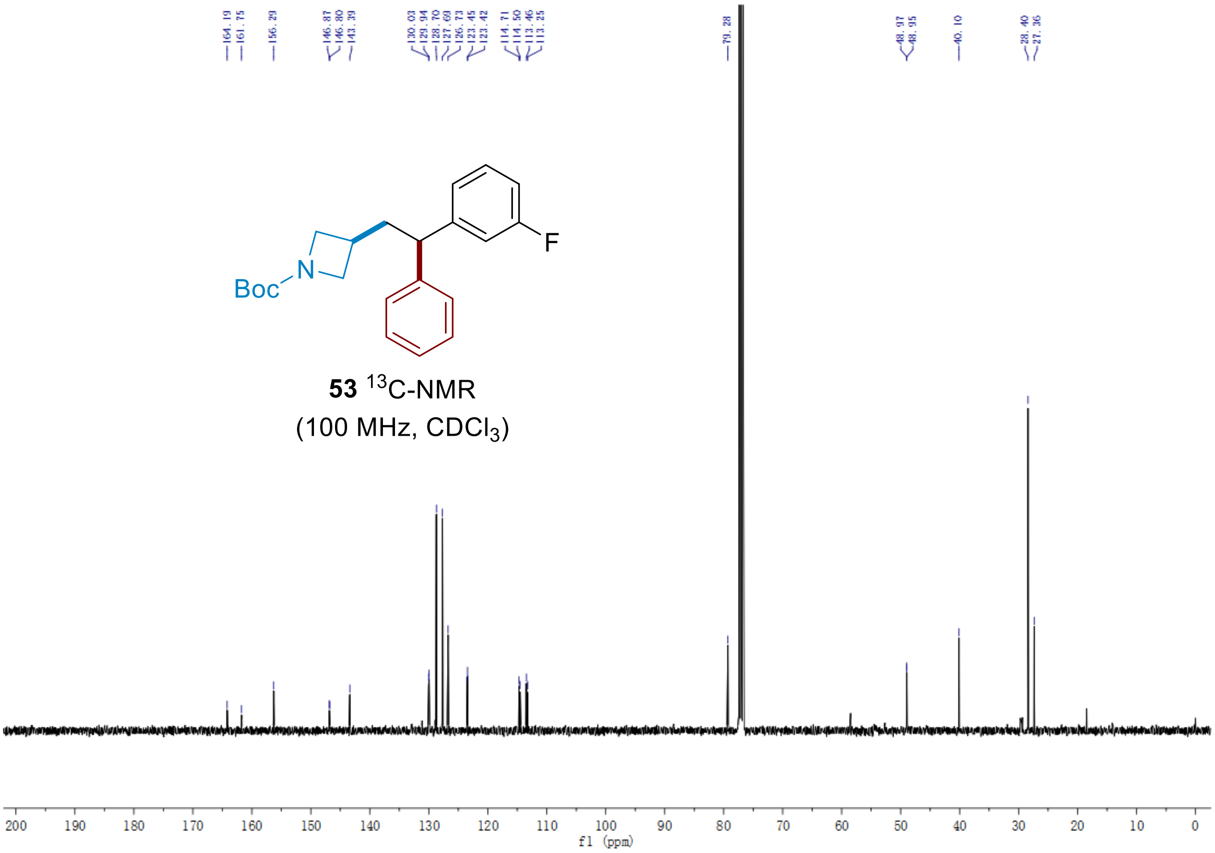
164.19
161.75
156.29
146.87
146.80
143.39
130.03
129.94
128.70
127.69
126.73
123.45
123.42
114.71
114.50
113.46
113.25
79.28
48.97
48.95
40.10
28.40
27.36
Boc
N
F
53 ^{13}C-NMR
(100 MHz, $CDCl_3$)
f1 (ppm)

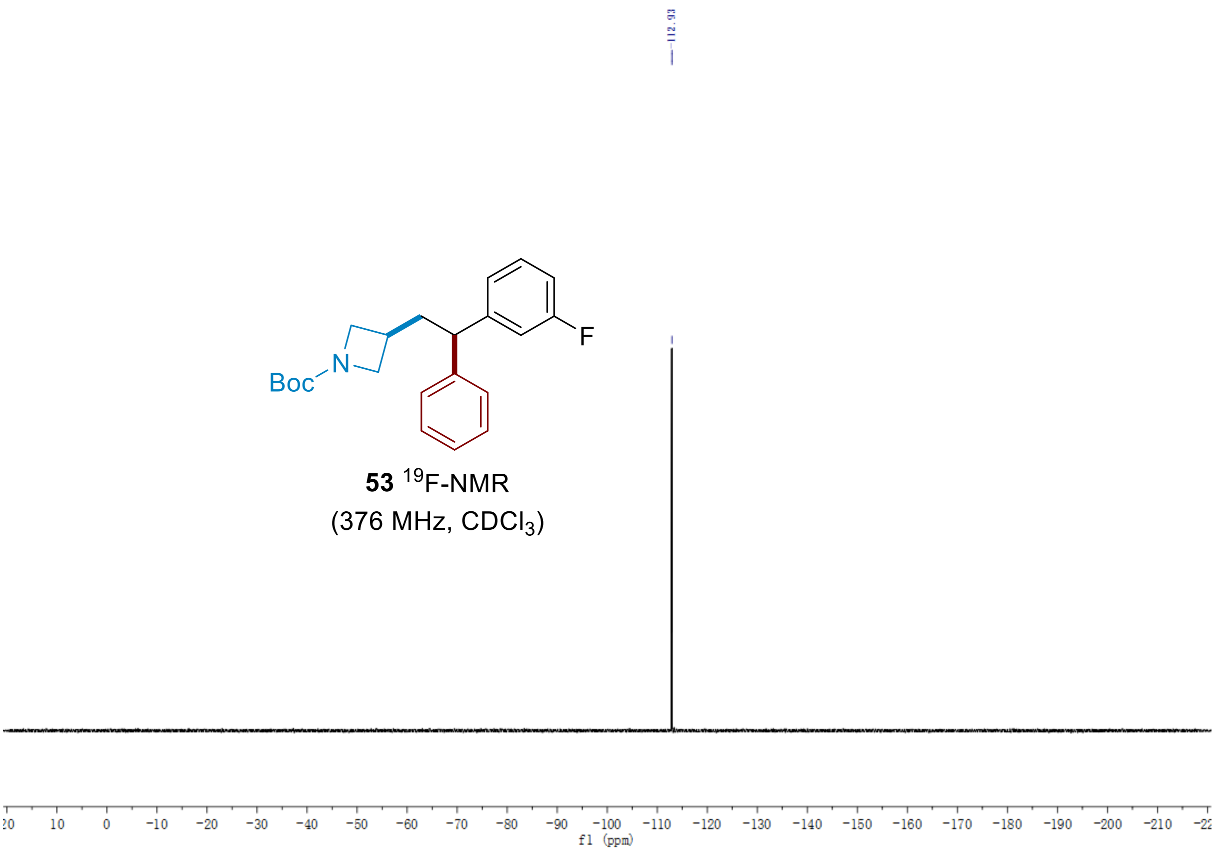
-112.93
Boc
N
F
53 ^{19}F-NMR
(376 MHz, $CDCl_3$)
f1 (ppm)

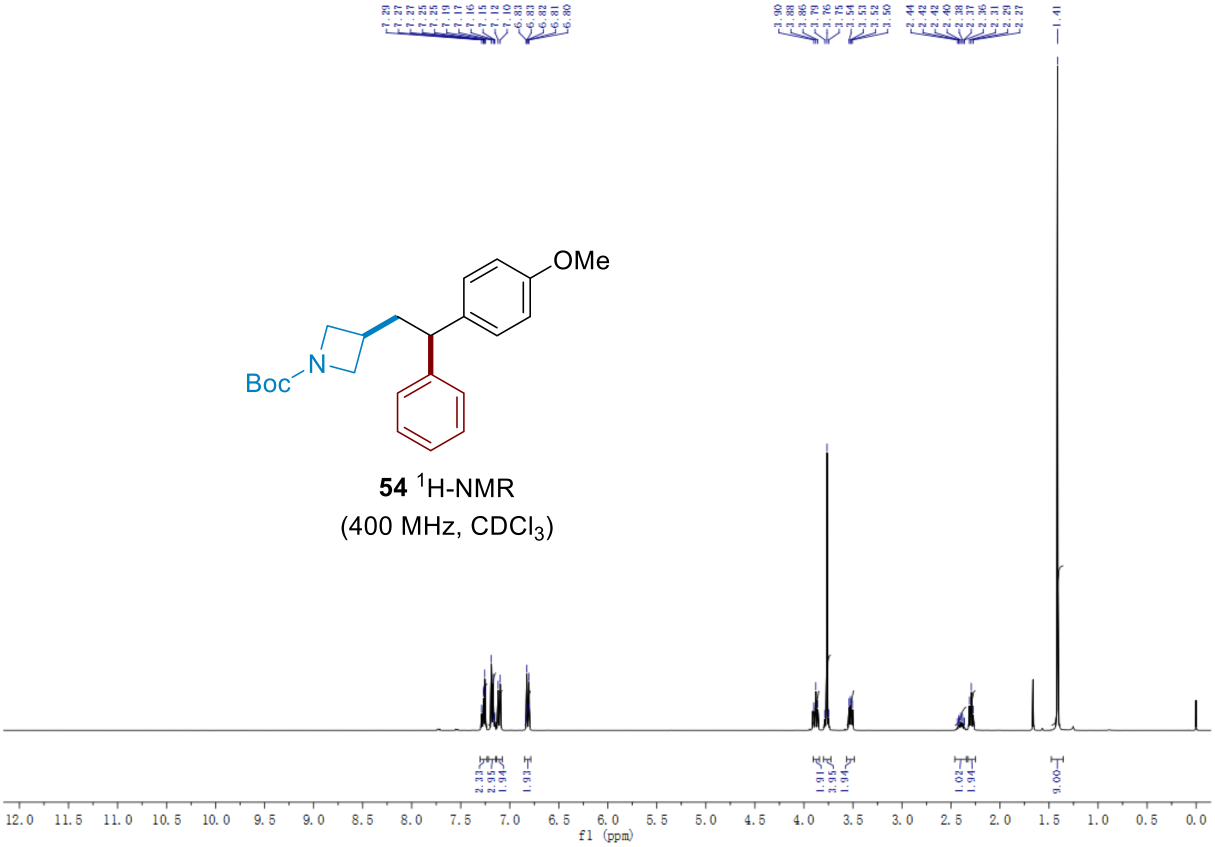
7.29 7.27 7.27 7.25 7.25 7.19 7.17 7.16 7.15 7.12 7.10 6.83 6.83 6.82 6.81 6.80
3.90 3.88 3.86 3.79 3.76 3.75 3.54 3.53 3.52 3.50
2.44 2.42 2.42 2.40 2.38 2.37 2.36 2.31 2.29 2.27
1.41
OMe
Boc
N
54 1H-NMR
(400 MHz, CDCl3)
2.33 2.95 1.94 1.93 1.91 3.95 1.94 1.02 1.94 9.00
12.0 11.5 11.0 10.5 10.0 9.5 9.0 8.5 8.0 7.5 7.0 6.5 6.0 5.5 5.0 4.5 4.0 3.5 3.0 2.5 2.0 1.5 1.0 0.5 0.0
f1 (ppm)

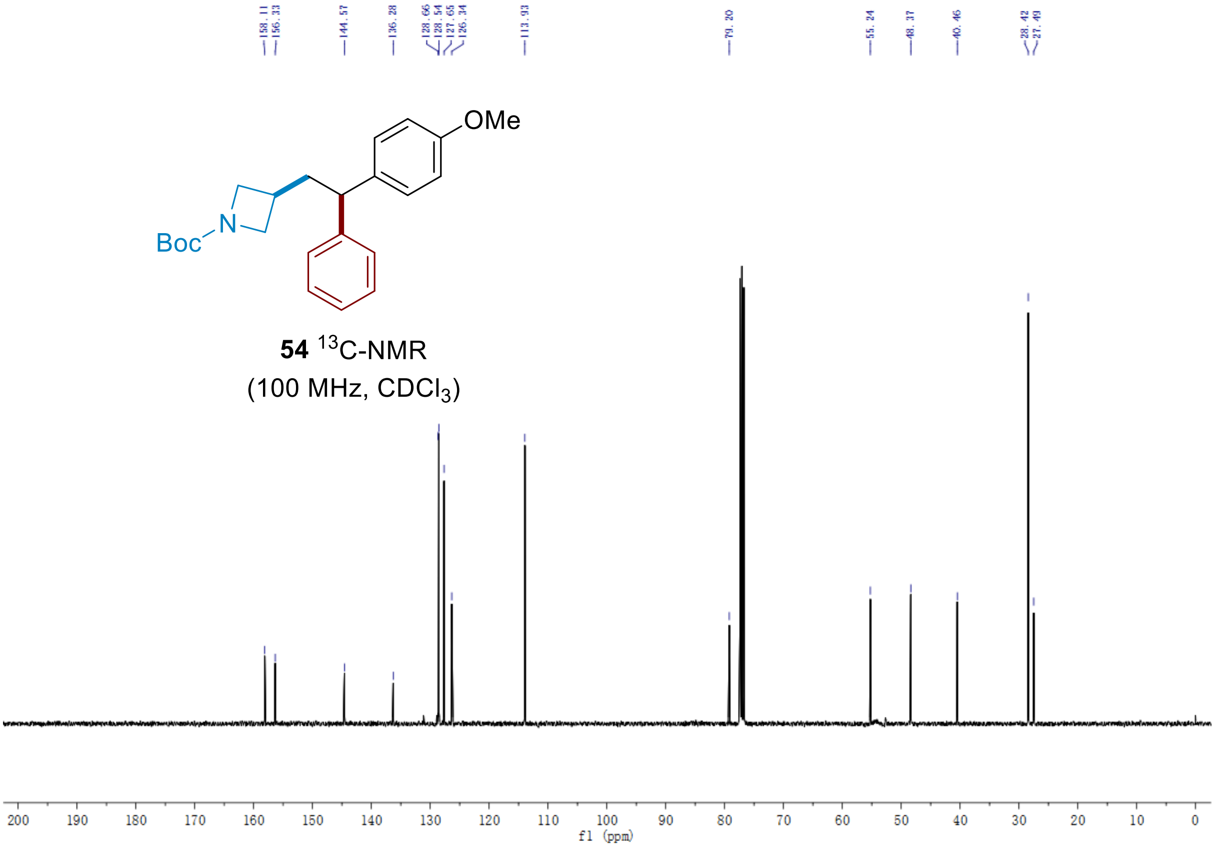
158.11 156.33 144.57 136.28 128.66 128.54 127.65 126.34 113.93 79.20 55.24 48.37 40.46 28.42 27.49
OMe
Boc
N
54 13C-NMR
(100 MHz, CDCl3)
200 190 180 170 160 150 140 130 120 110 100 90 80 70 60 50 40 30 20 10 0
f1 (ppm)

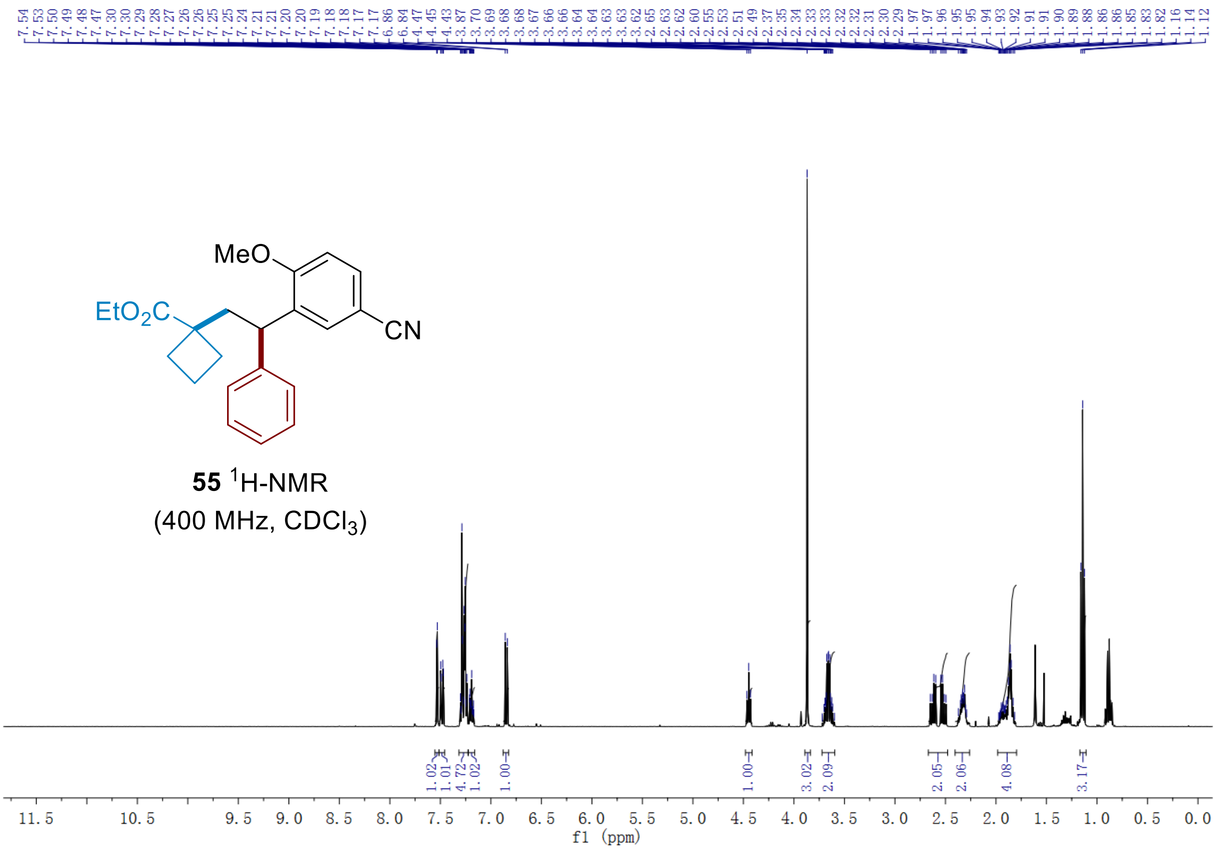
7.54 7.53 7.50 7.49 7.48 7.47 7.30 7.29 7.28 7.27 7.26 7.26 7.25 7.25 7.24 7.21 7.21 7.20 7.19 7.18 7.18 7.17 7.17 6.86 6.84 4.47 4.45 4.43 3.87 3.70 3.69 3.68 3.68 3.67 3.66 3.66 3.64 3.64 3.63 3.63 3.62 2.65 2.63 2.62 2.60 2.55 2.53 2.51 2.49 2.37 2.35 2.34 2.33 2.33 2.32 2.32 2.31 2.30 2.29 1.97 1.97 1.96 1.95 1.95 1.94 1.93 1.92 1.91 1.91 1.90 1.89 1.88 1.86 1.86 1.85 1.83 1.82 1.16 1.14 1.12
MeO
EtO2C
CN
55 1H-NMR
(400 MHz, CDCl3)
1.02 1.01 4.72 1.02 1.00 1.00 3.02 2.09 2.05 2.06 4.08 3.17
f1 (ppm)

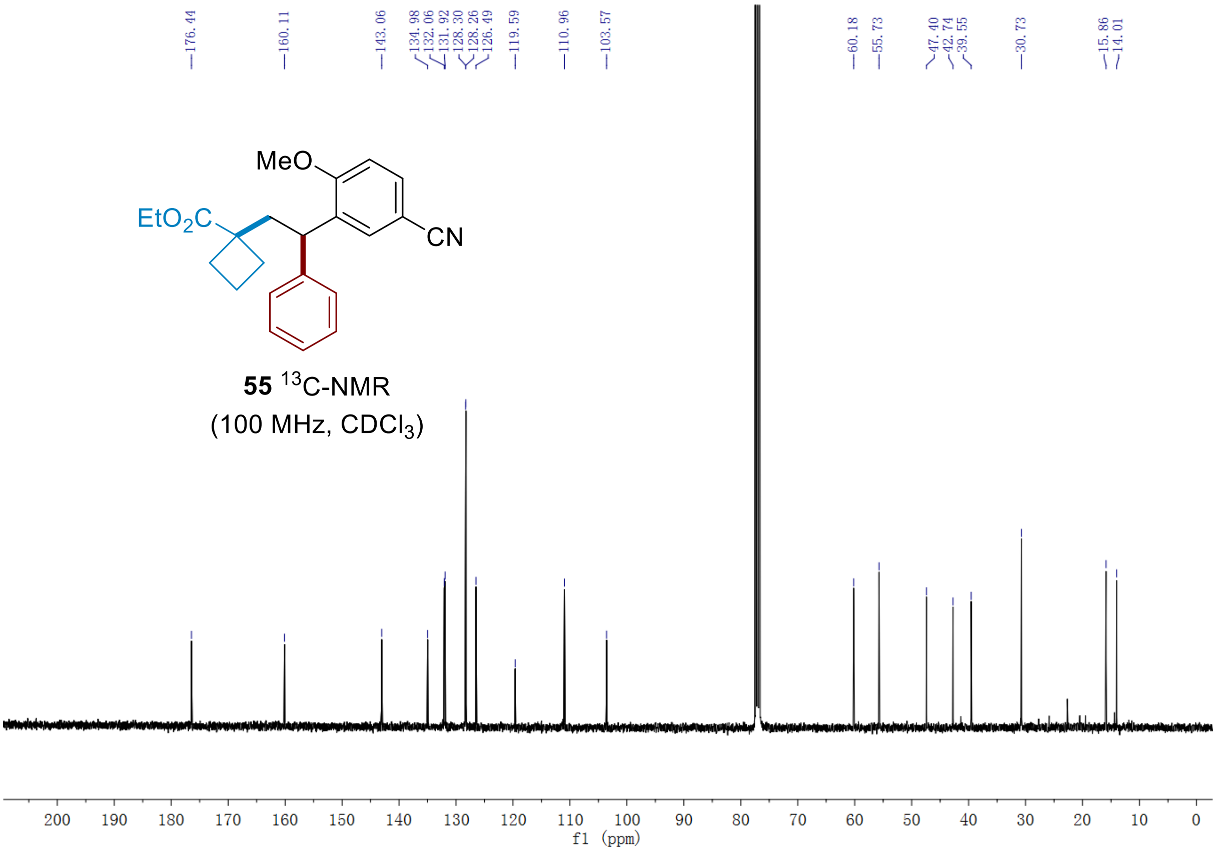
176.44
160.11
143.06
134.98 132.06 131.92 128.30 128.26 126.49
119.59
110.96
103.57
60.18
55.73
47.40 42.74 39.55
30.73
15.86 14.01
MeO
EtO2C
CN
55 13C-NMR
(100 MHz, CDCl3)
f1 (ppm)

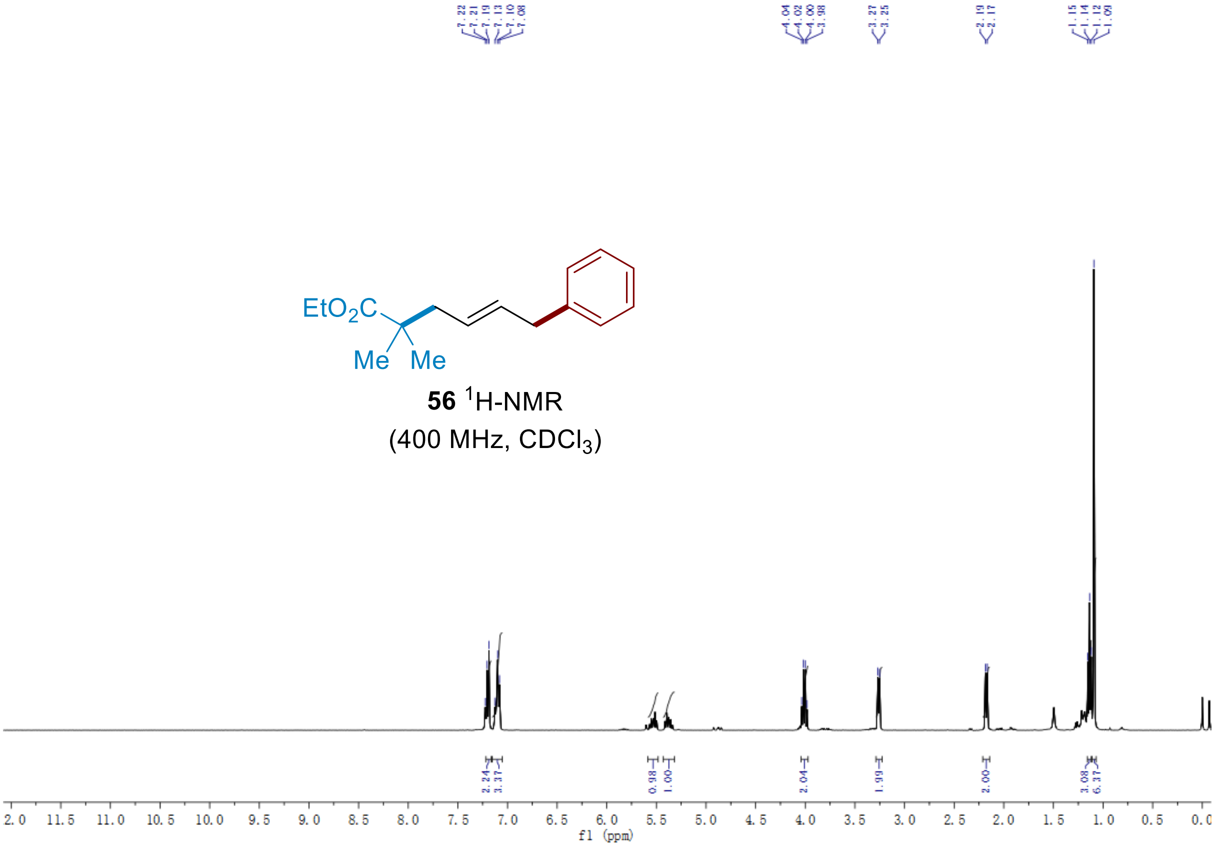
7.22 7.21 7.19 7.13 7.10 7.08
4.04 4.02 4.00 3.98
3.27 3.25
2.19 2.17
1.15 1.14 1.12 1.09
EtO2C
Me Me
56 1H-NMR
(400 MHz, CDCl3)
2.24 3.37
0.98 1.00
2.04
1.99
2.00
3.08 6.37
f1 (ppm)

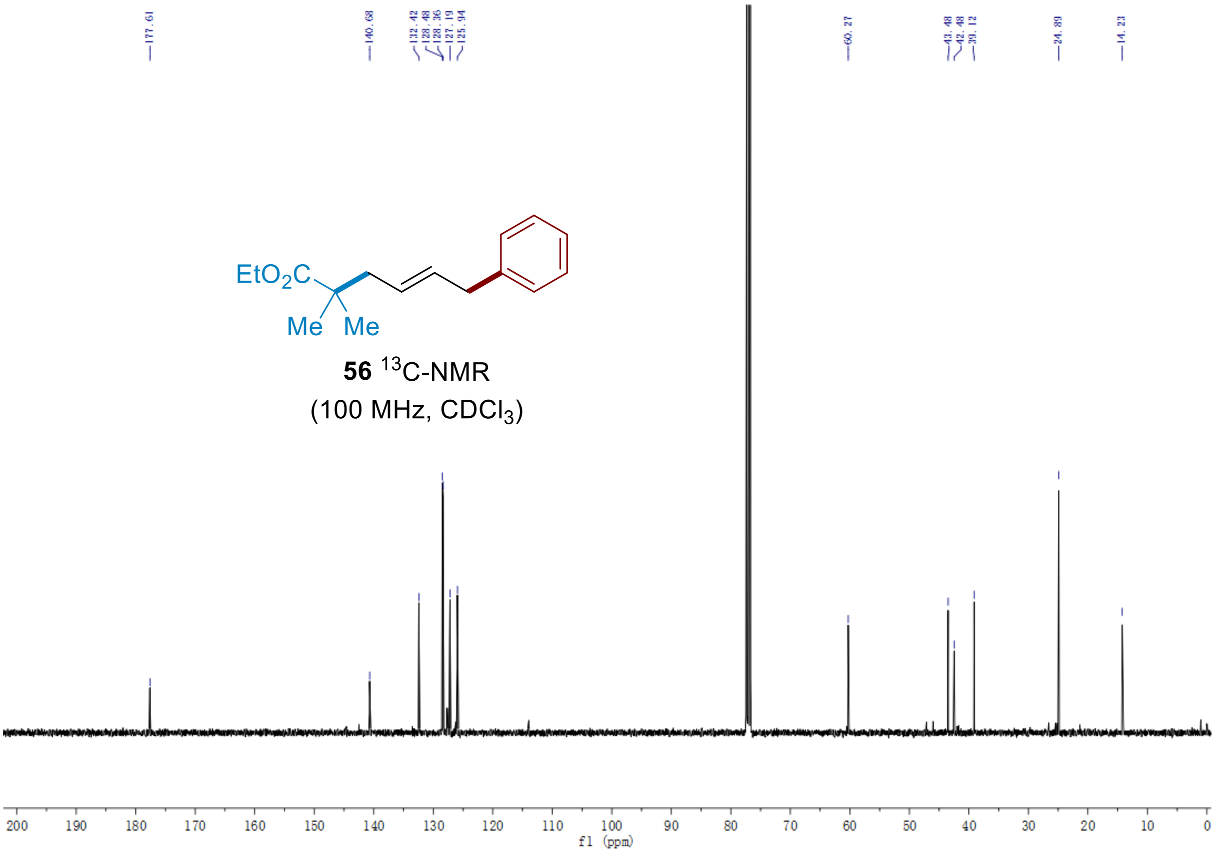
177.61
140.68
132.42 128.48 128.36 127.19 125.94
60.27
43.48 42.48 39.12
24.89
14.23
EtO2C
Me Me
56 13C-NMR
(100 MHz, CDCl3)
f1 (ppm)

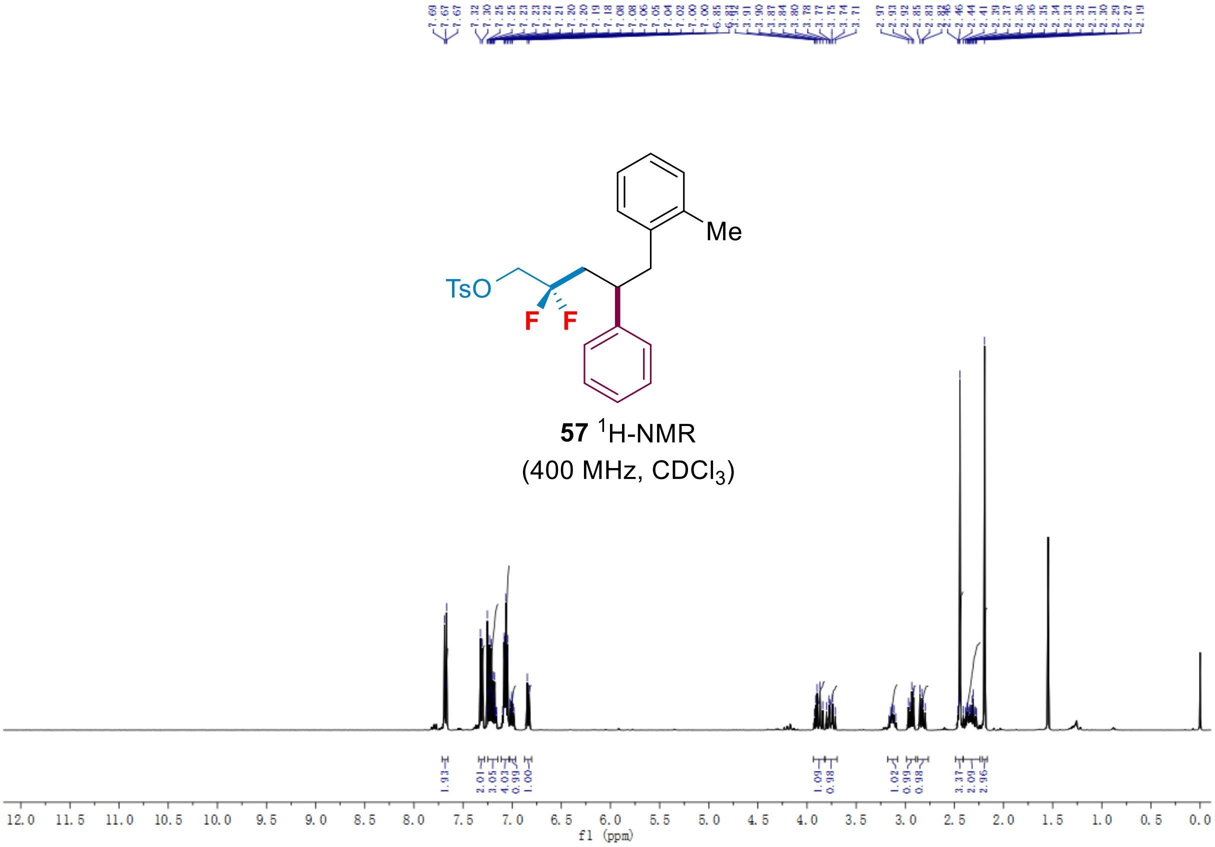

TsO
F F
Me
57 ¹H-NMR
(400 MHz, CDCl₃)
f1 (ppm)

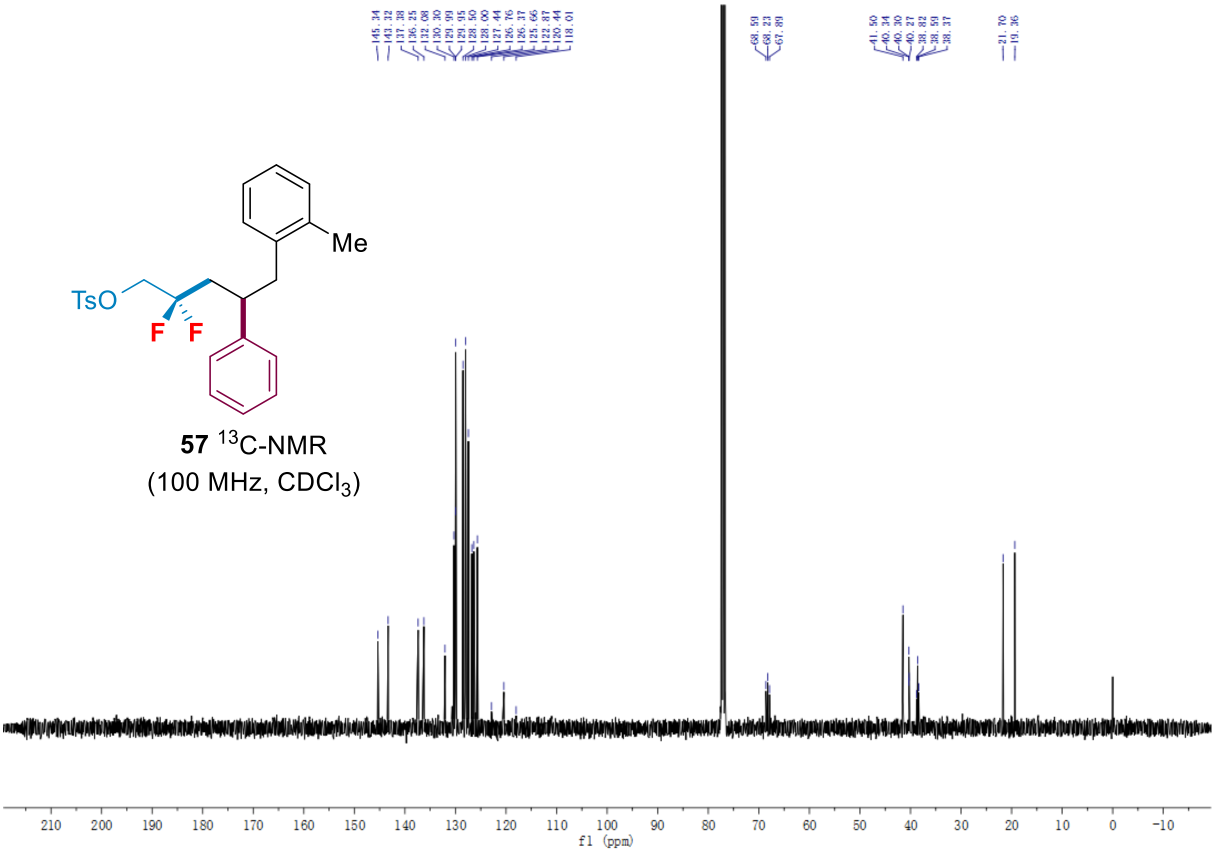

TsO
F F
Me
57 ¹³C-NMR
(100 MHz, CDCl₃)
f1 (ppm)

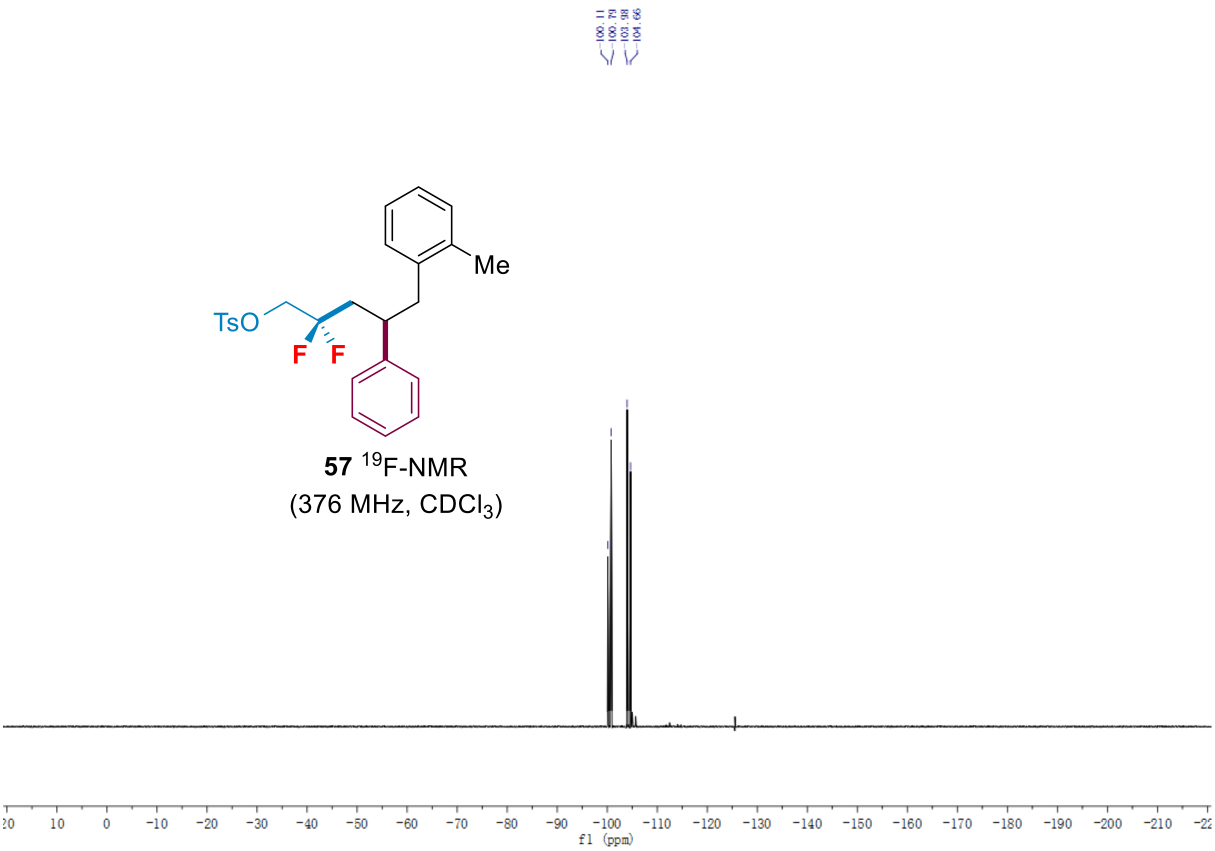

57 ^{19}F-NMR
(376 MHz, $CDCl_3$)

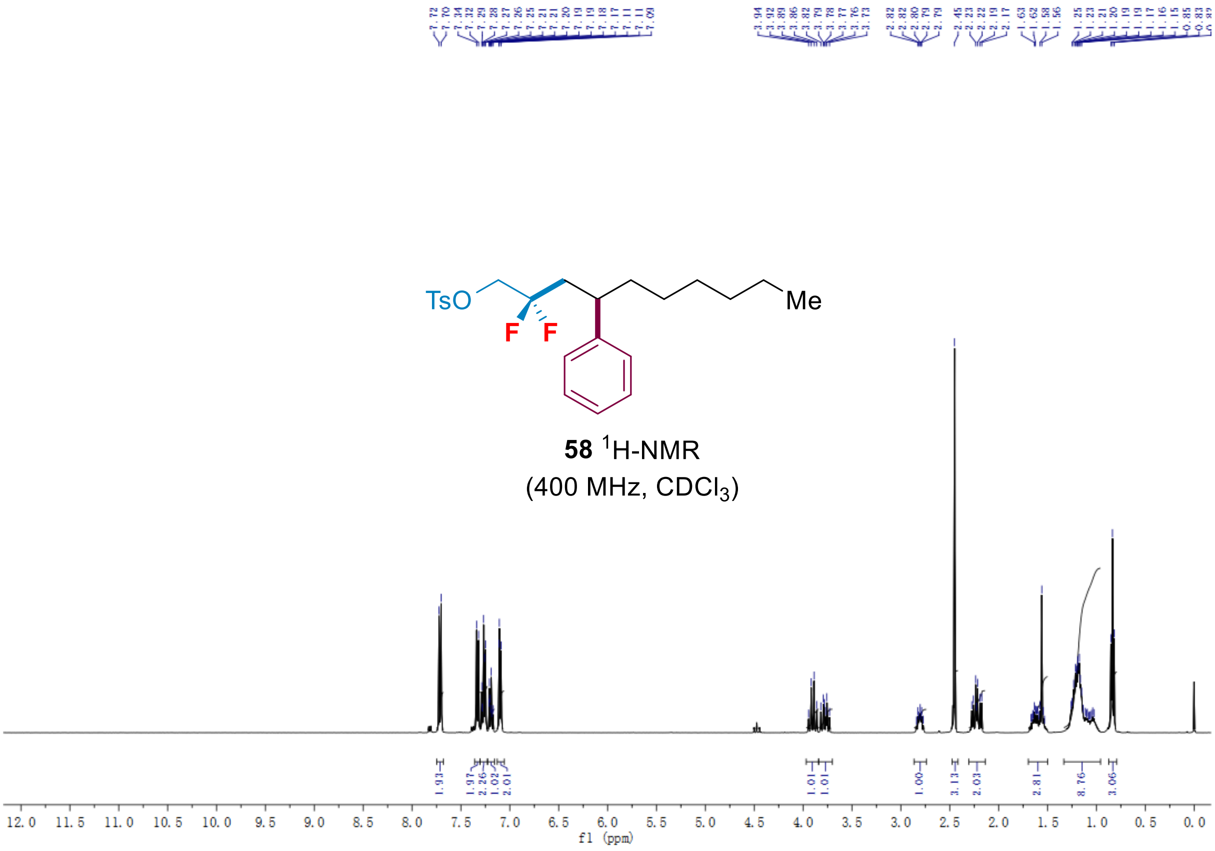

58 ^{1}H-NMR
(400 MHz, $CDCl_3$)

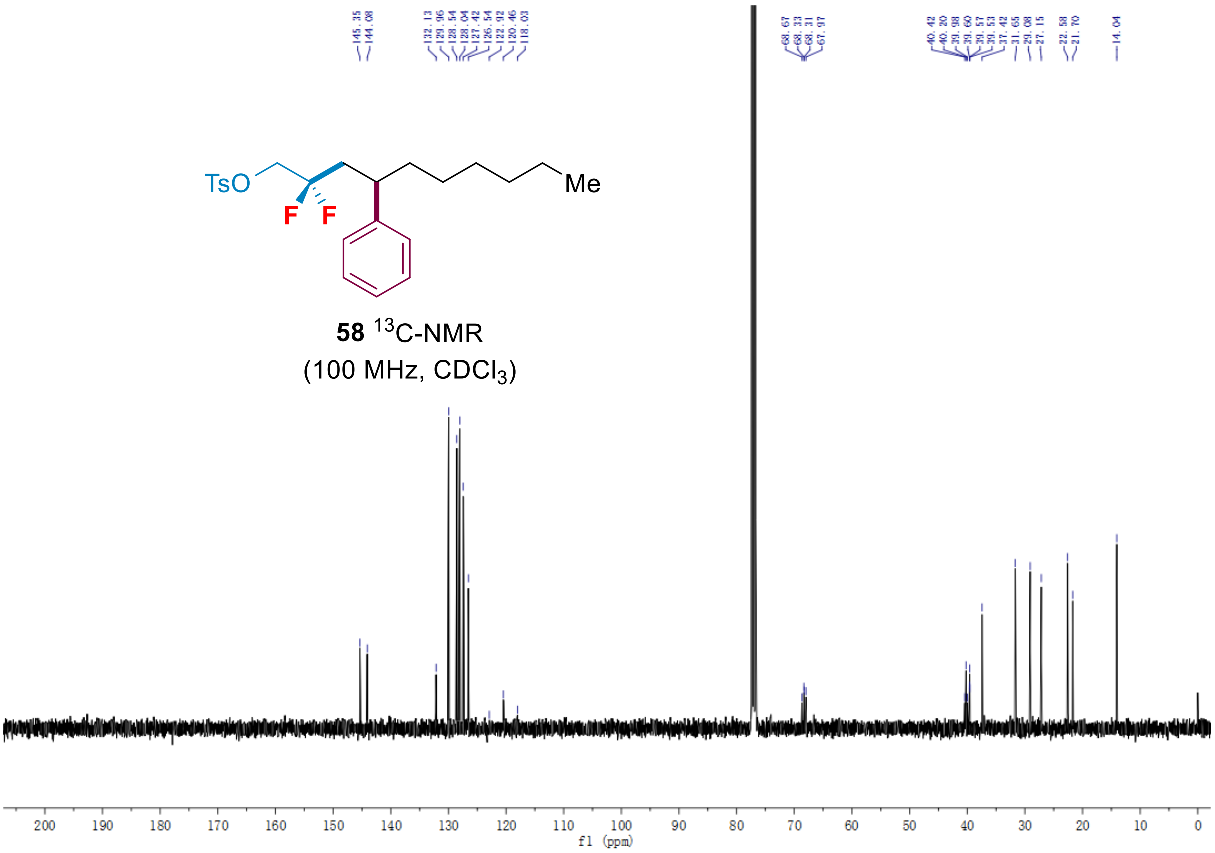

TsO
F F
Me
58 13C-NMR
(100 MHz, CDCl3)
f1 (ppm)

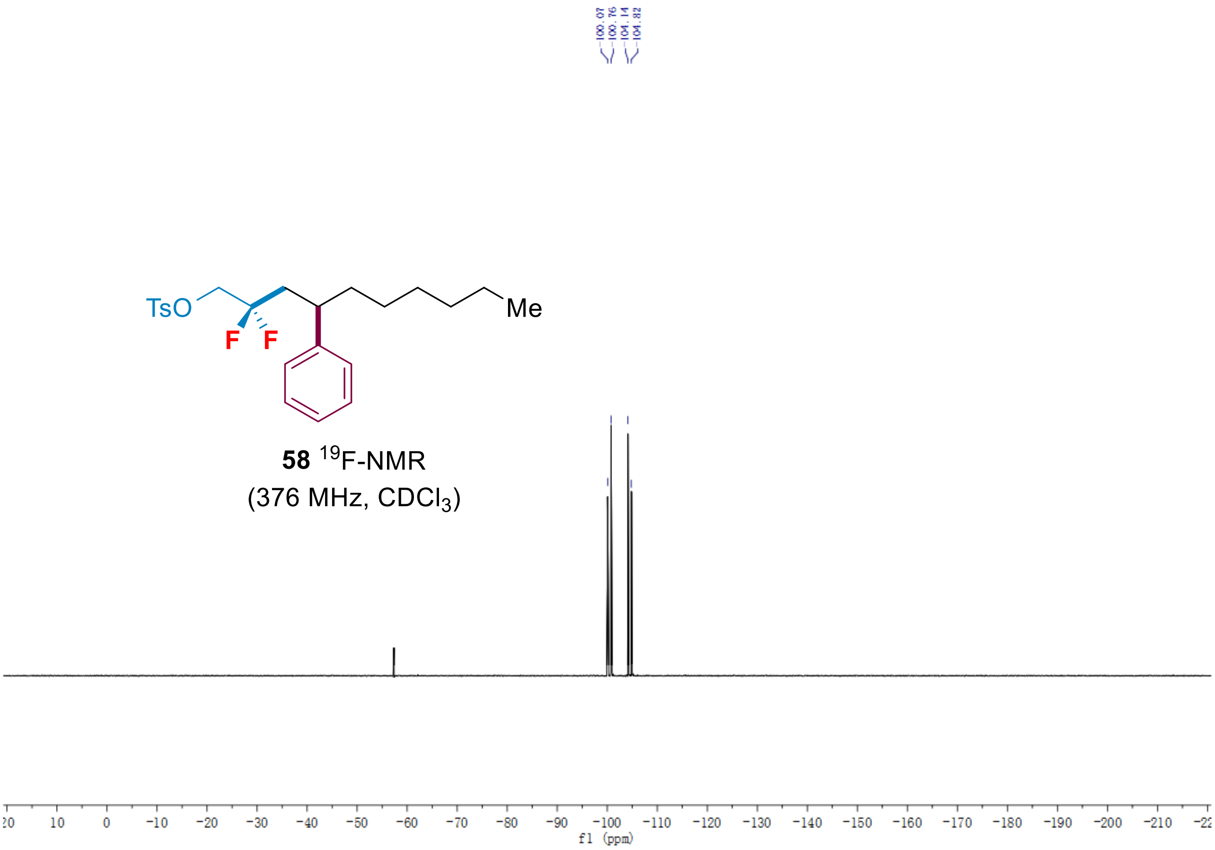

TsO
F F
Me
58 19F-NMR
(376 MHz, CDCl3)
f1 (ppm)

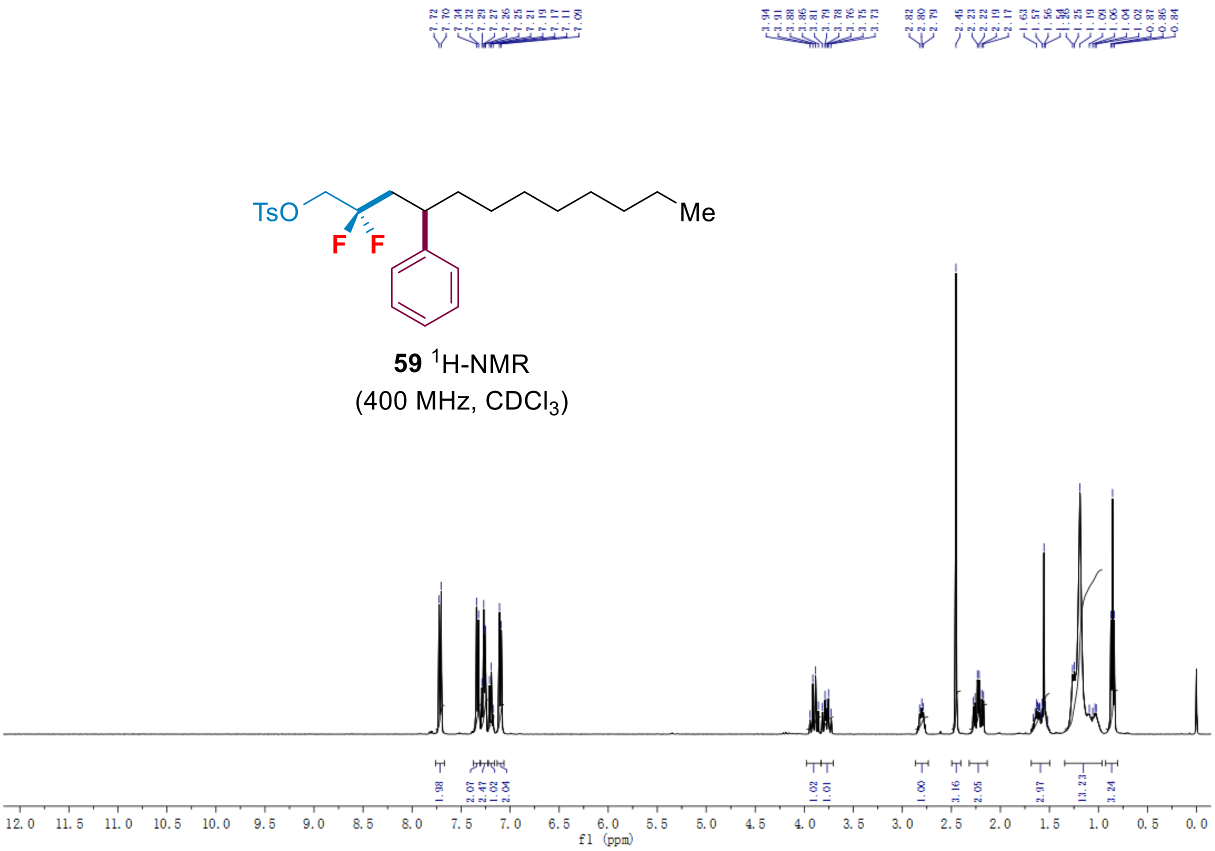
TsO
F F
Me
59 ^{1}H-NMR
(400 MHz, $CDCl_3$)
f1 (ppm)

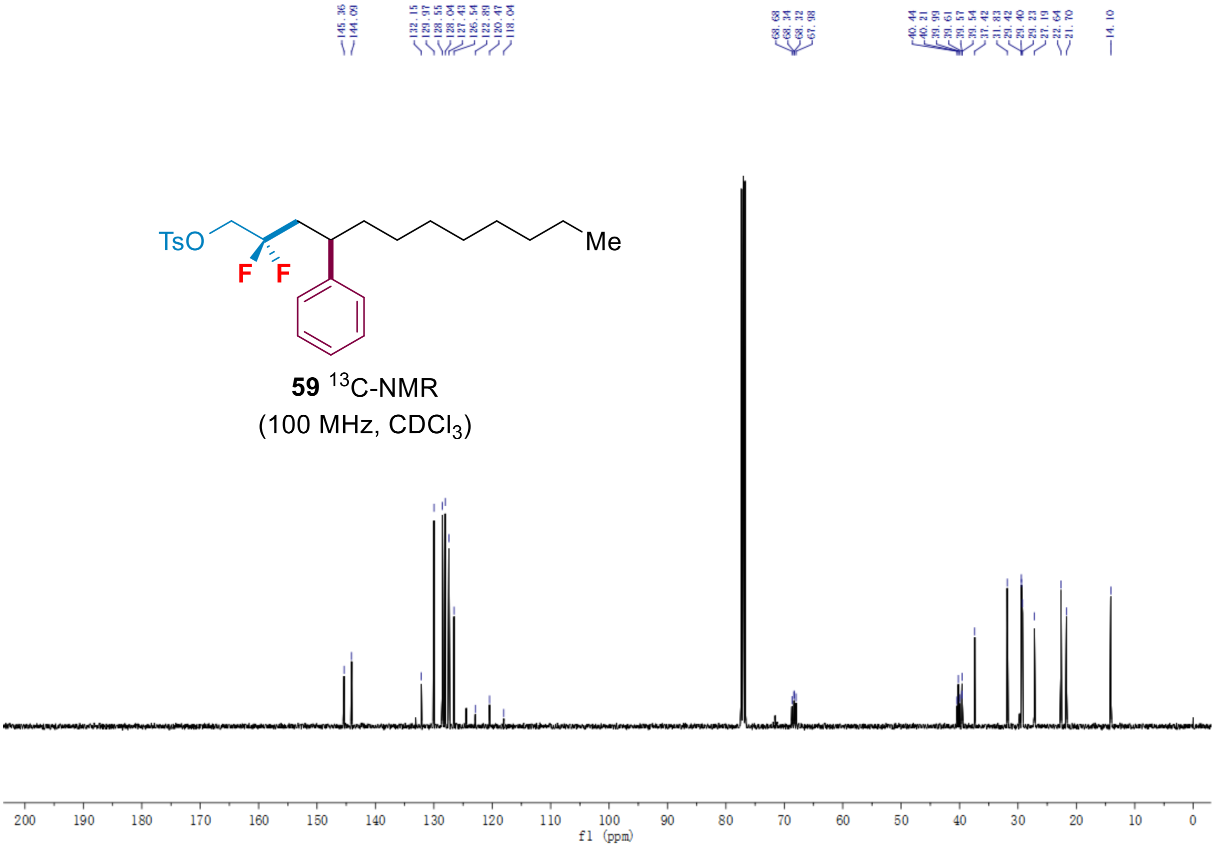
TsO
F F
Me
59 ^{13}C-NMR
(100 MHz, $CDCl_3$)
f1 (ppm)

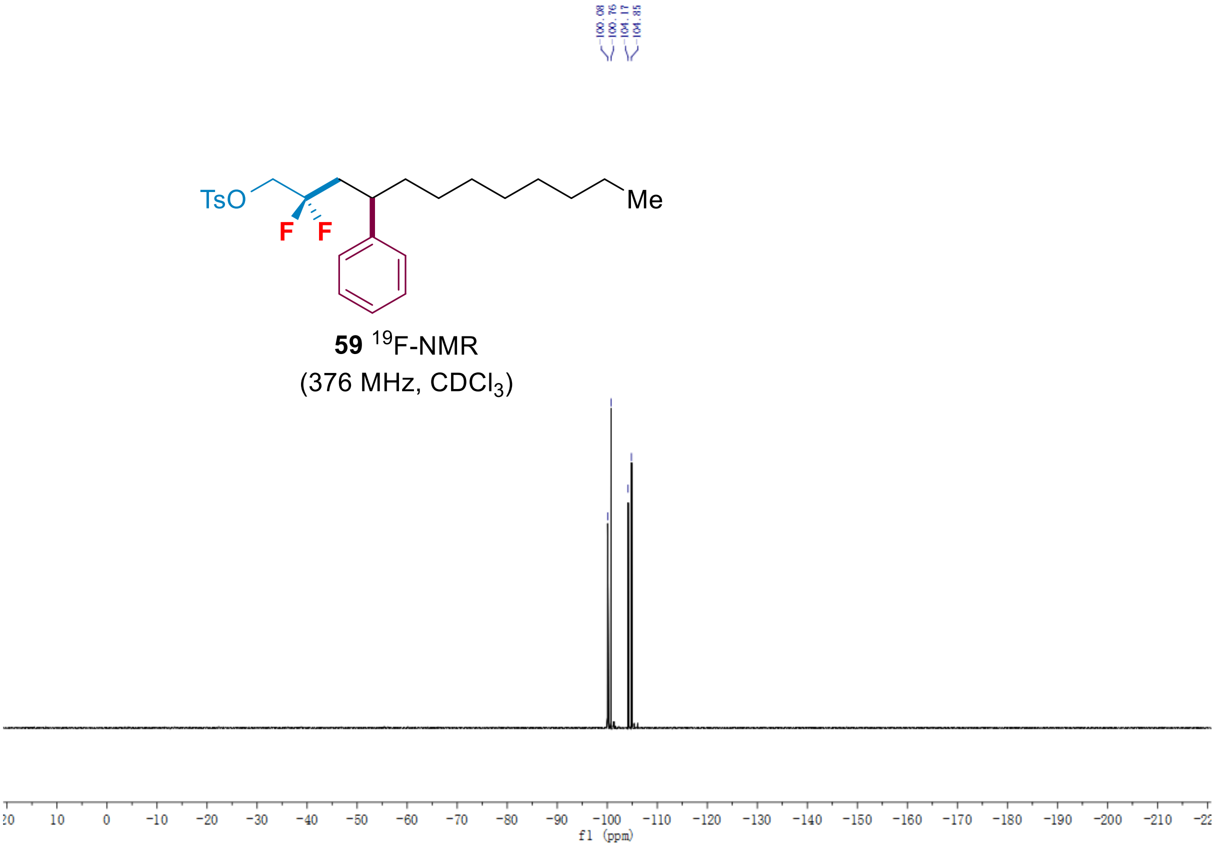

59 ^{19}F-NMR
(376 MHz, $CDCl_3$)

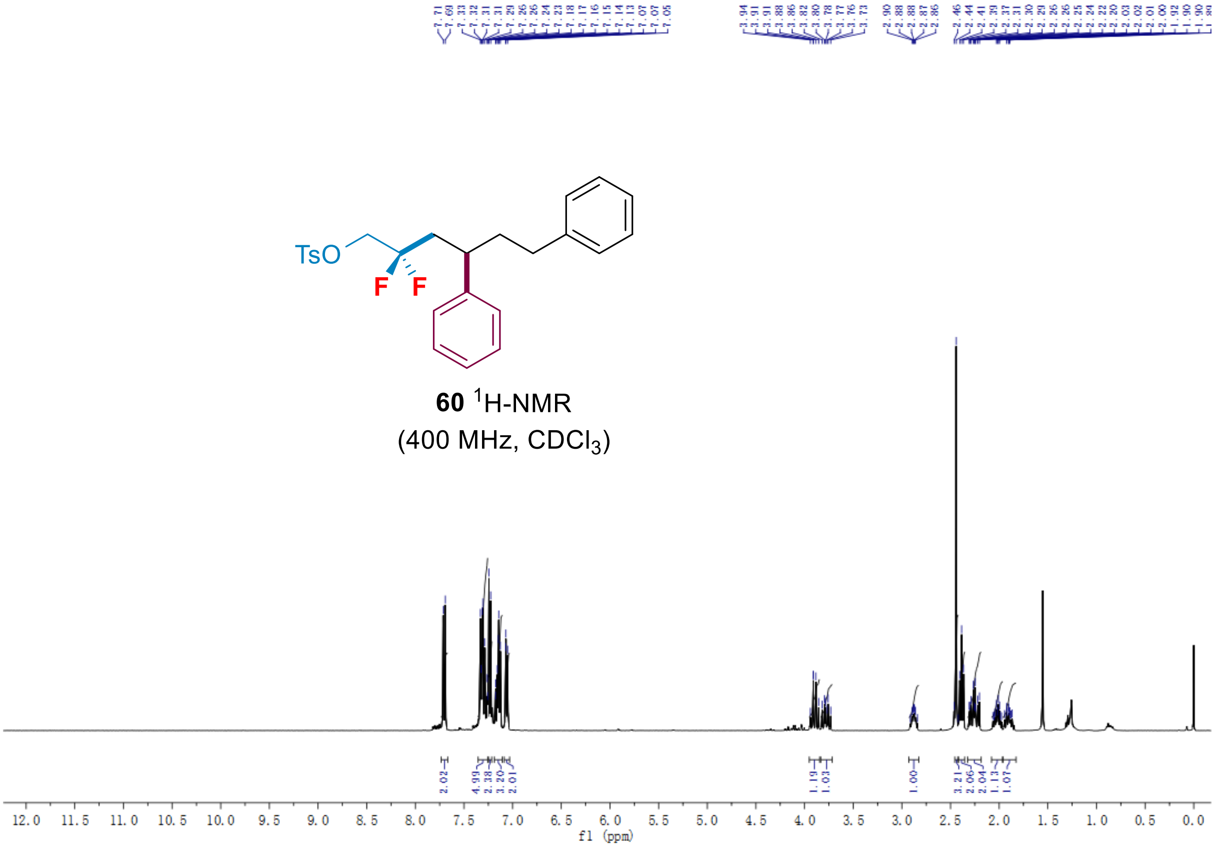

60 ^{1}H-NMR
(400 MHz, $CDCl_3$)

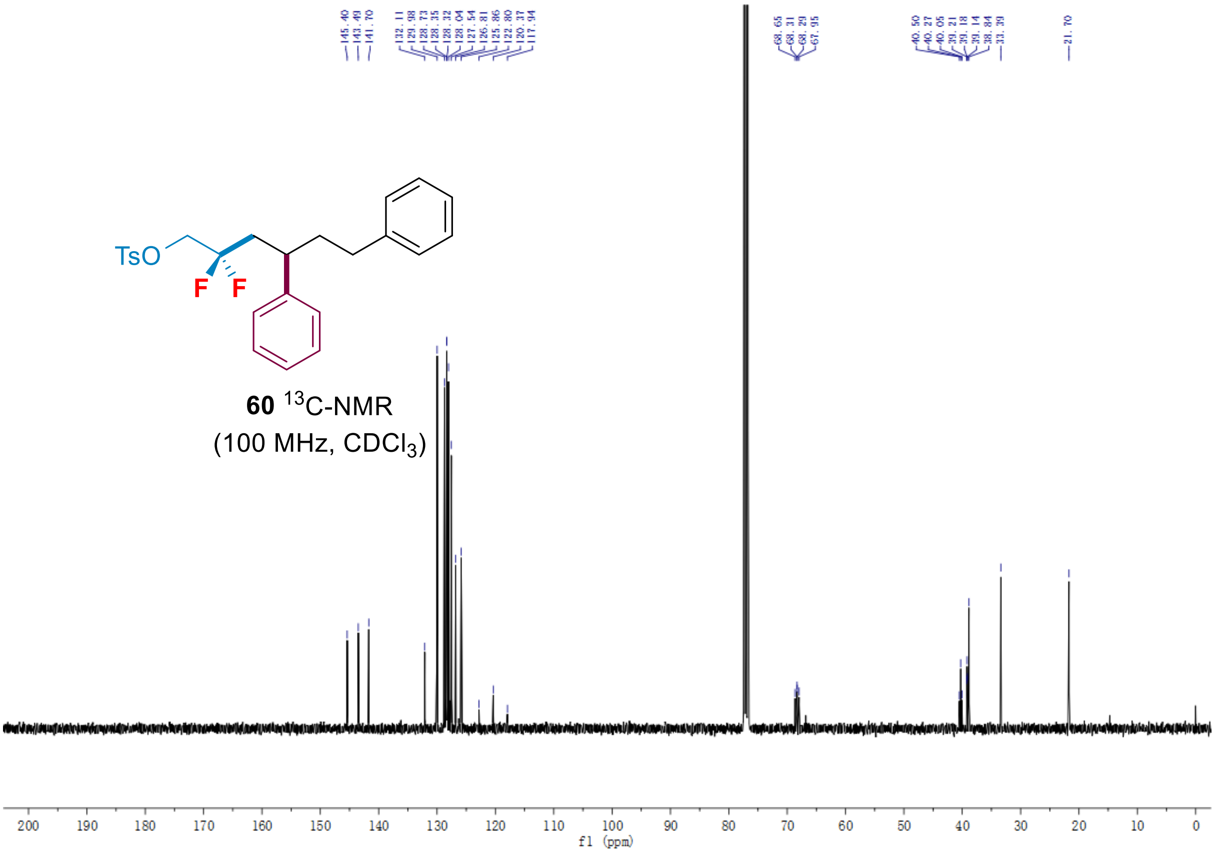

60 ^{13}C-NMR
(100 MHz, $CDCl_3$)

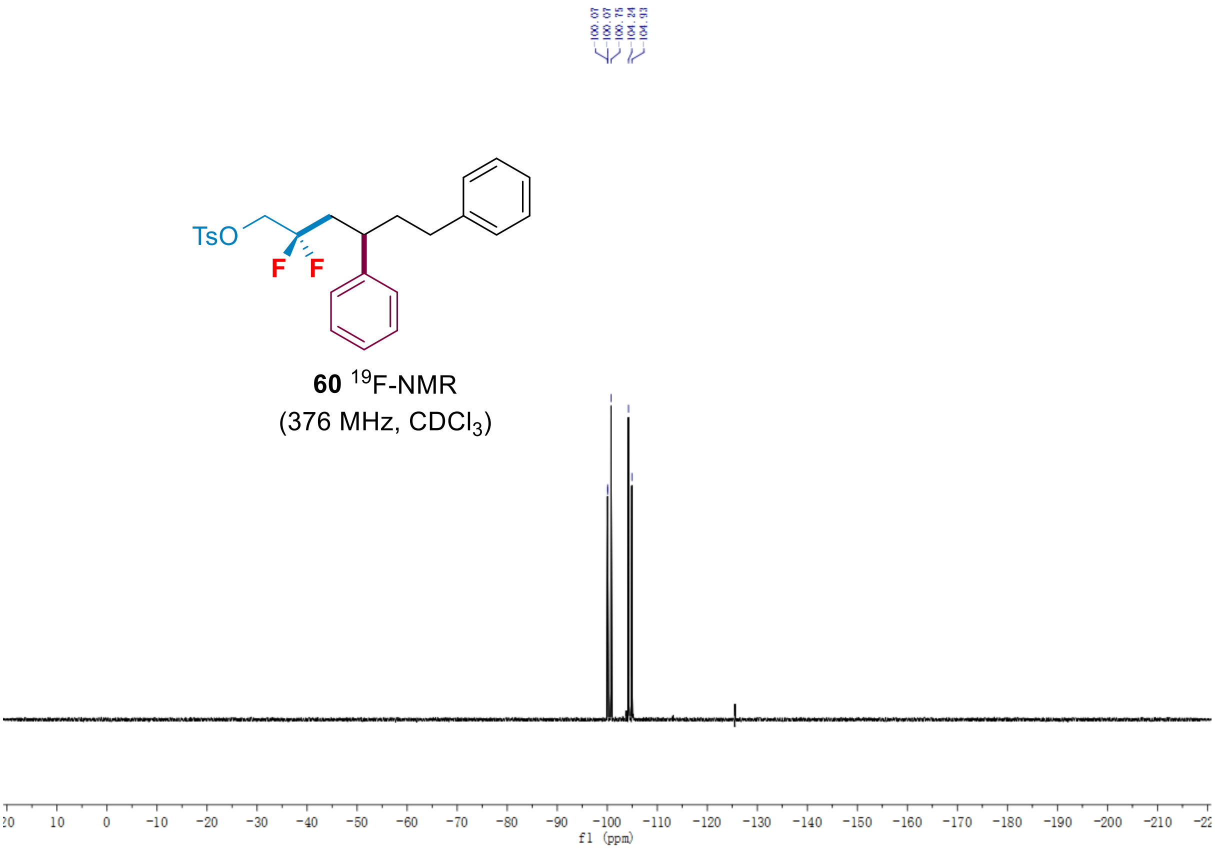

60 ^{19}F-NMR
(376 MHz, $CDCl_3$)

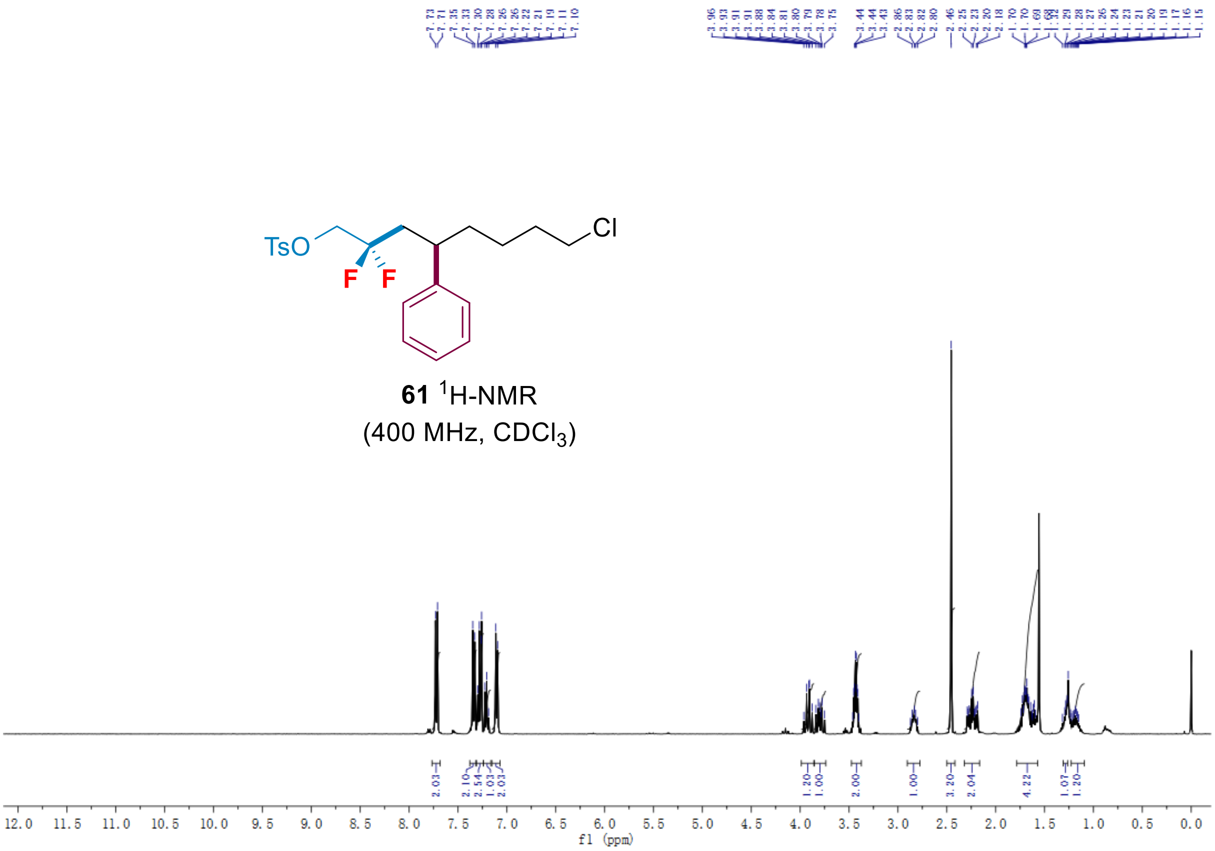
TsO
F
F
Cl
61 1H-NMR
(400 MHz, CDCl3)
f1 (ppm)

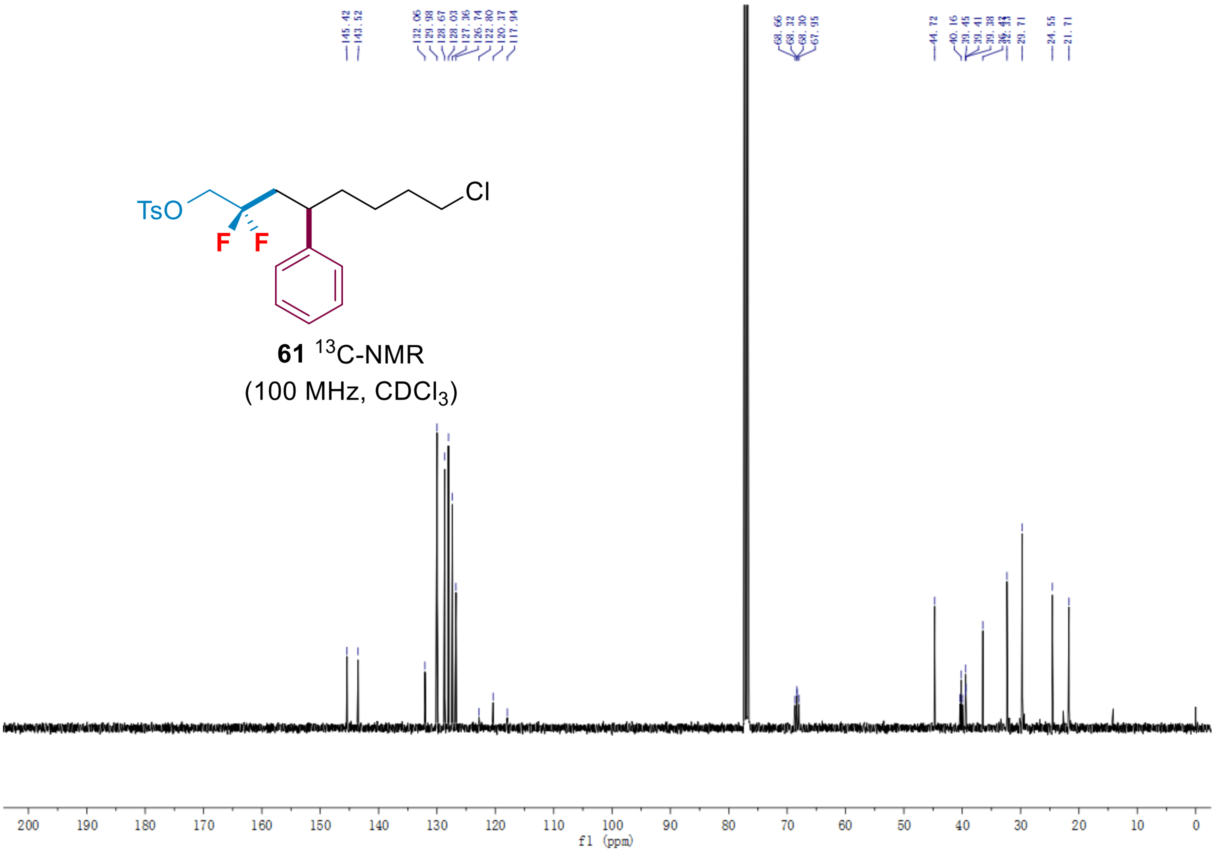
TsO
F
F
Cl
61 13C-NMR
(100 MHz, CDCl3)
f1 (ppm)

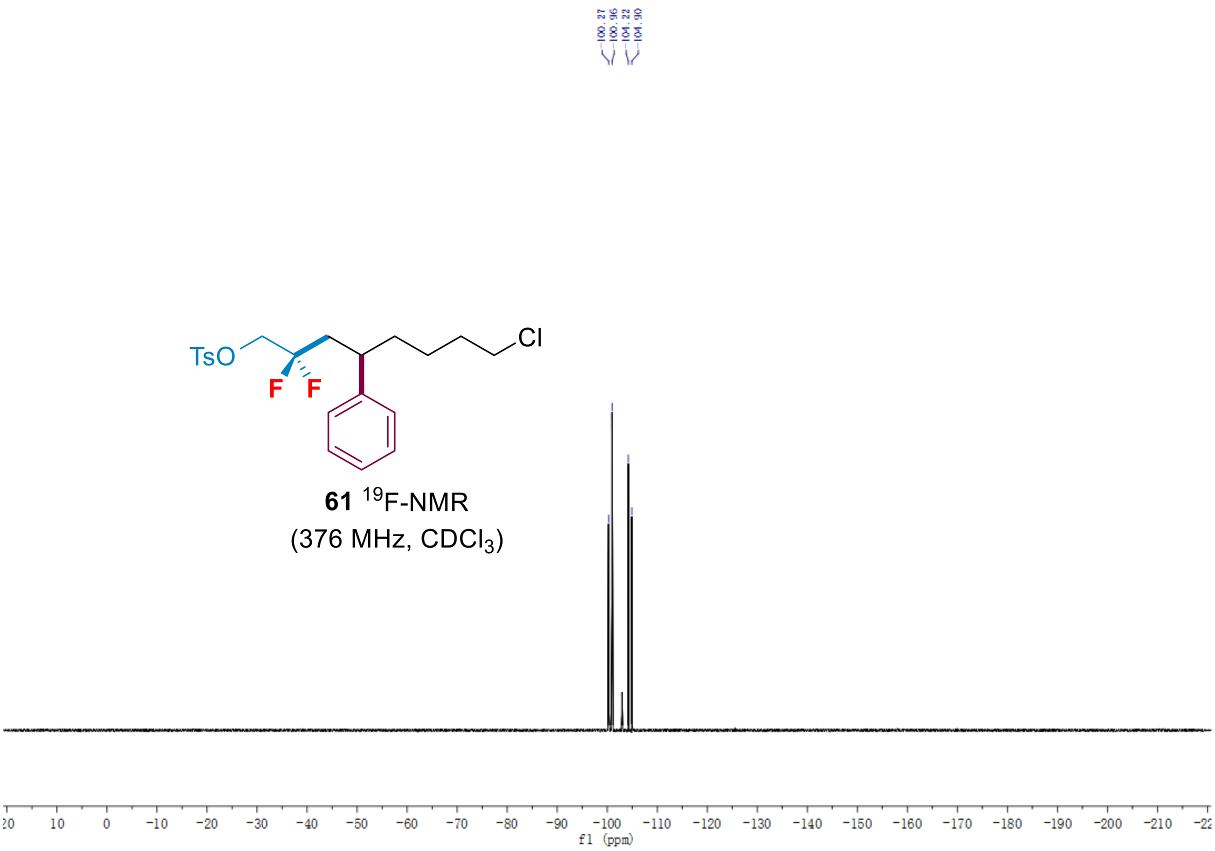

61 ^{19}F-NMR
(376 MHz, $CDCl_3$)

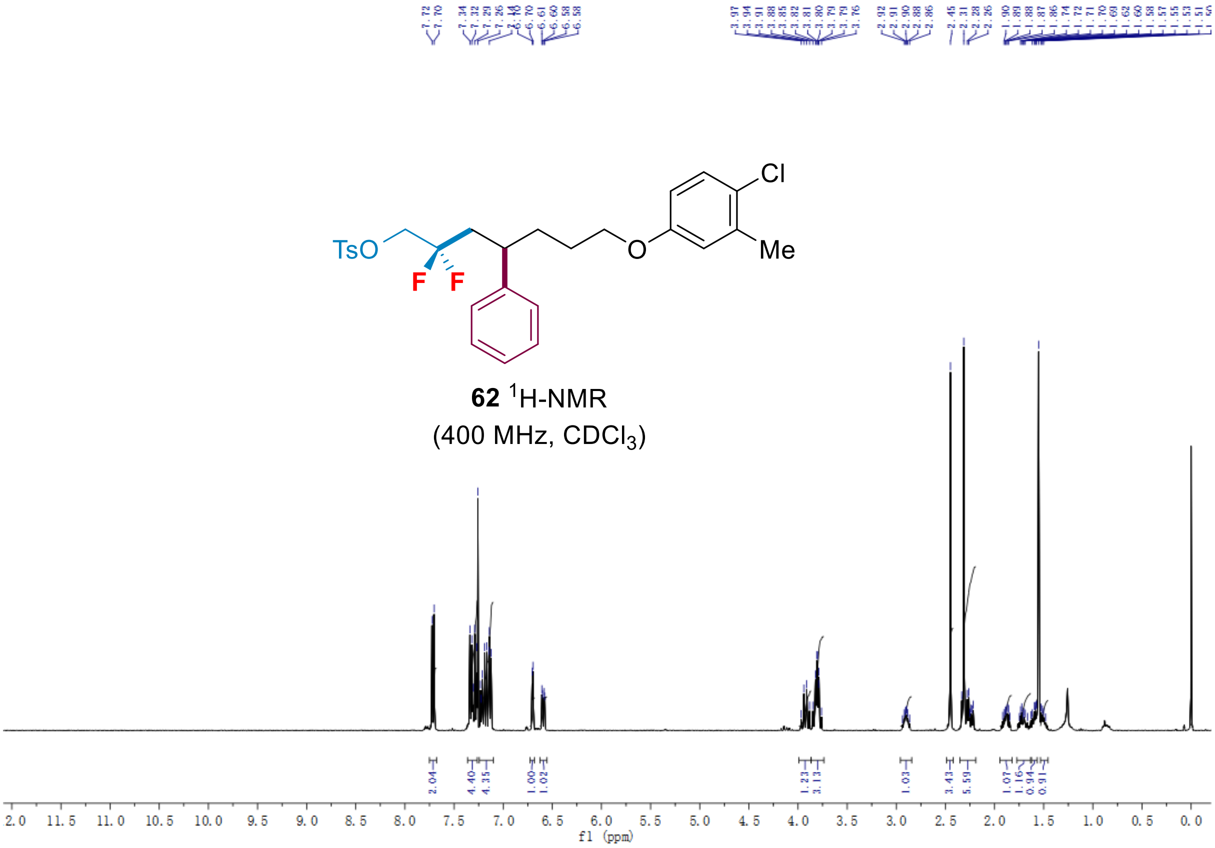

62 ^{1}H-NMR
(400 MHz, $CDCl_3$)

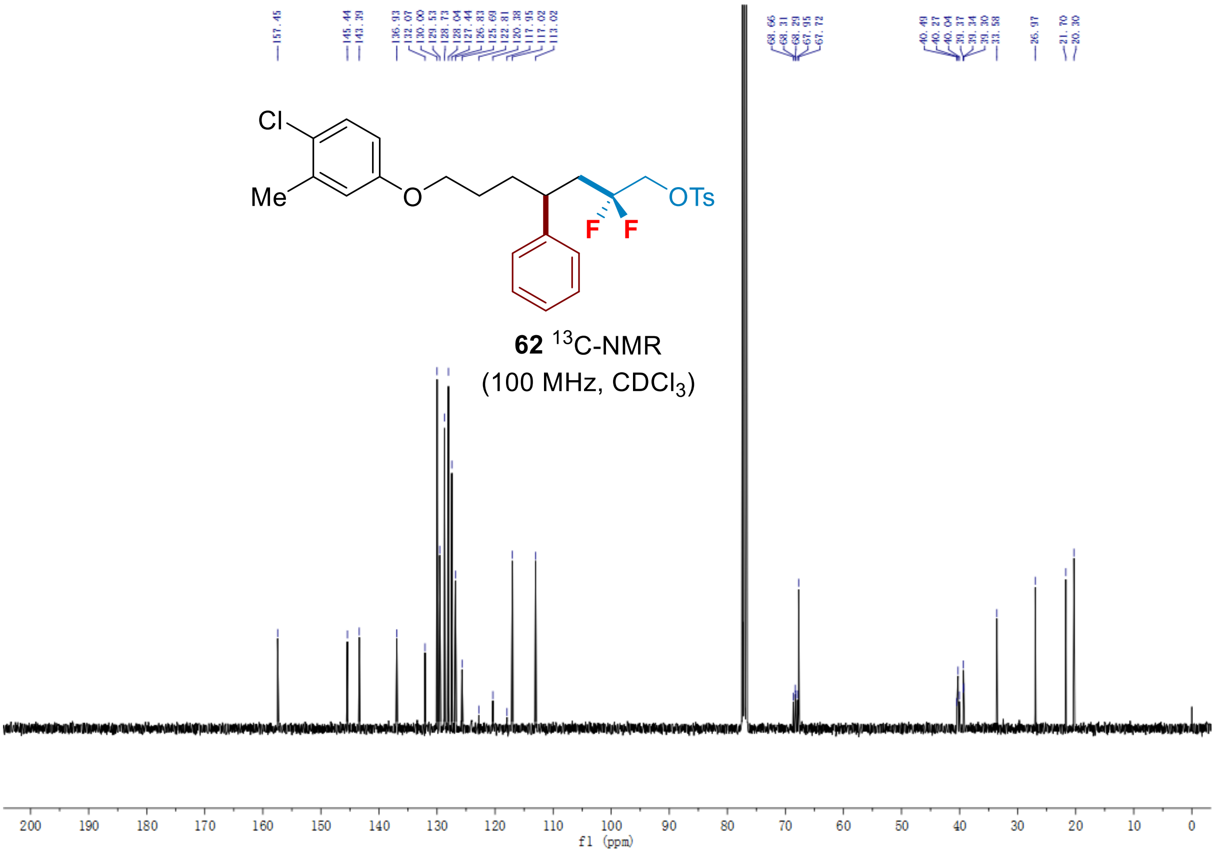
157.45
145.44
143.39
136.93
132.07
130.00
129.53
128.73
128.04
127.44
126.83
125.69
122.81
120.38
117.95
117.02
113.02
68.66
68.31
68.29
67.95
67.72
40.49
40.27
40.04
39.37
39.34
39.30
33.58
26.97
21.70
20.30
Cl
Me
O
OTs
F
F
62 13C-NMR
(100 MHz, CDCl3)
f1 (ppm)

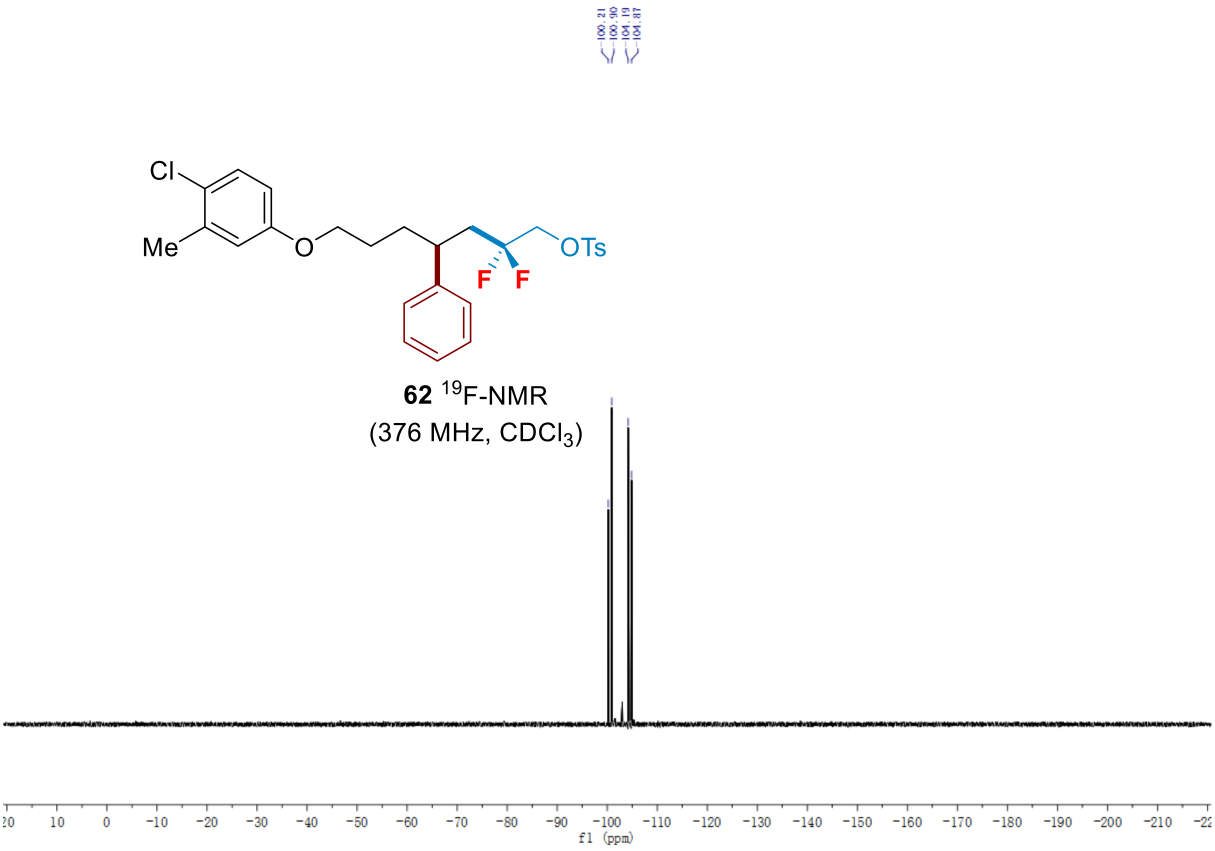
-100.21
-100.90
-104.19
-104.87
Cl
Me
O
OTs
F
F
62 19F-NMR
(376 MHz, CDCl3)
f1 (ppm)

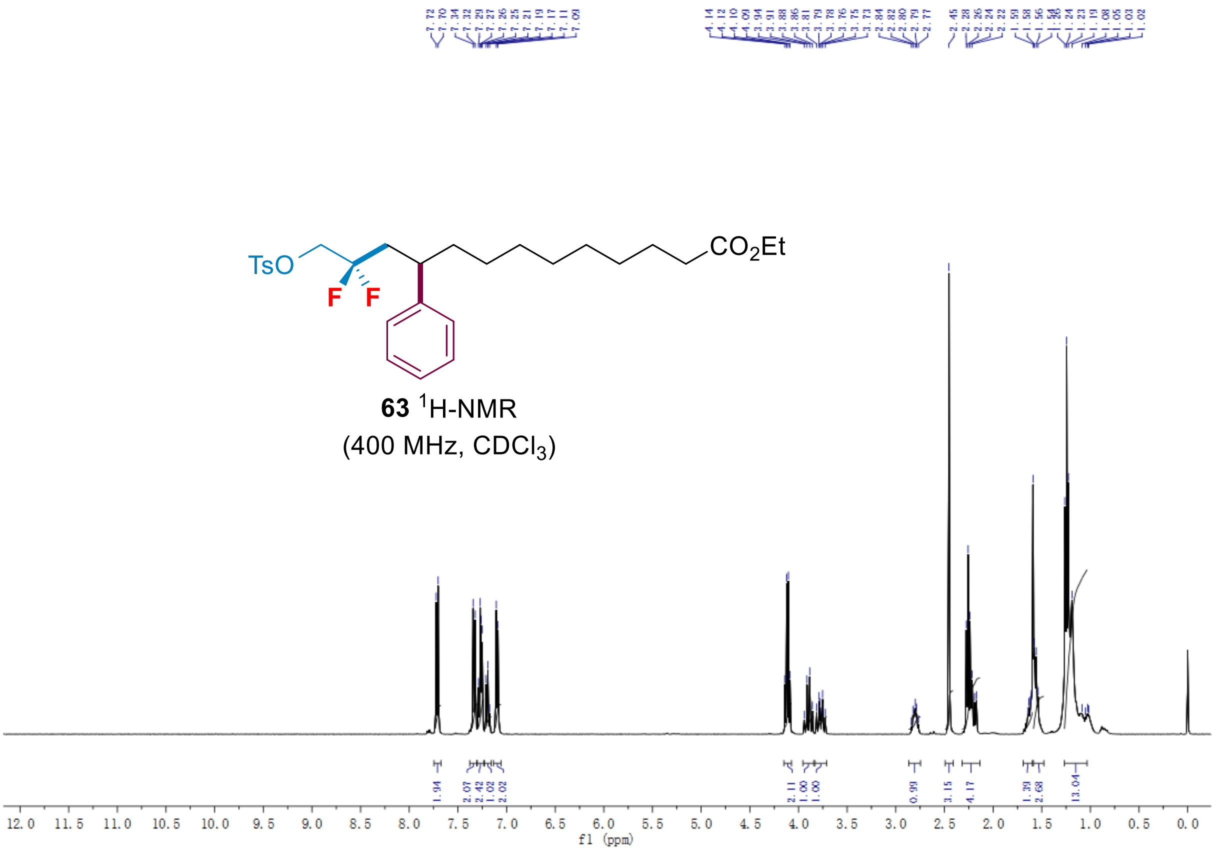

63 ^{1}H-NMR
(400 MHz, $CDCl_3$)

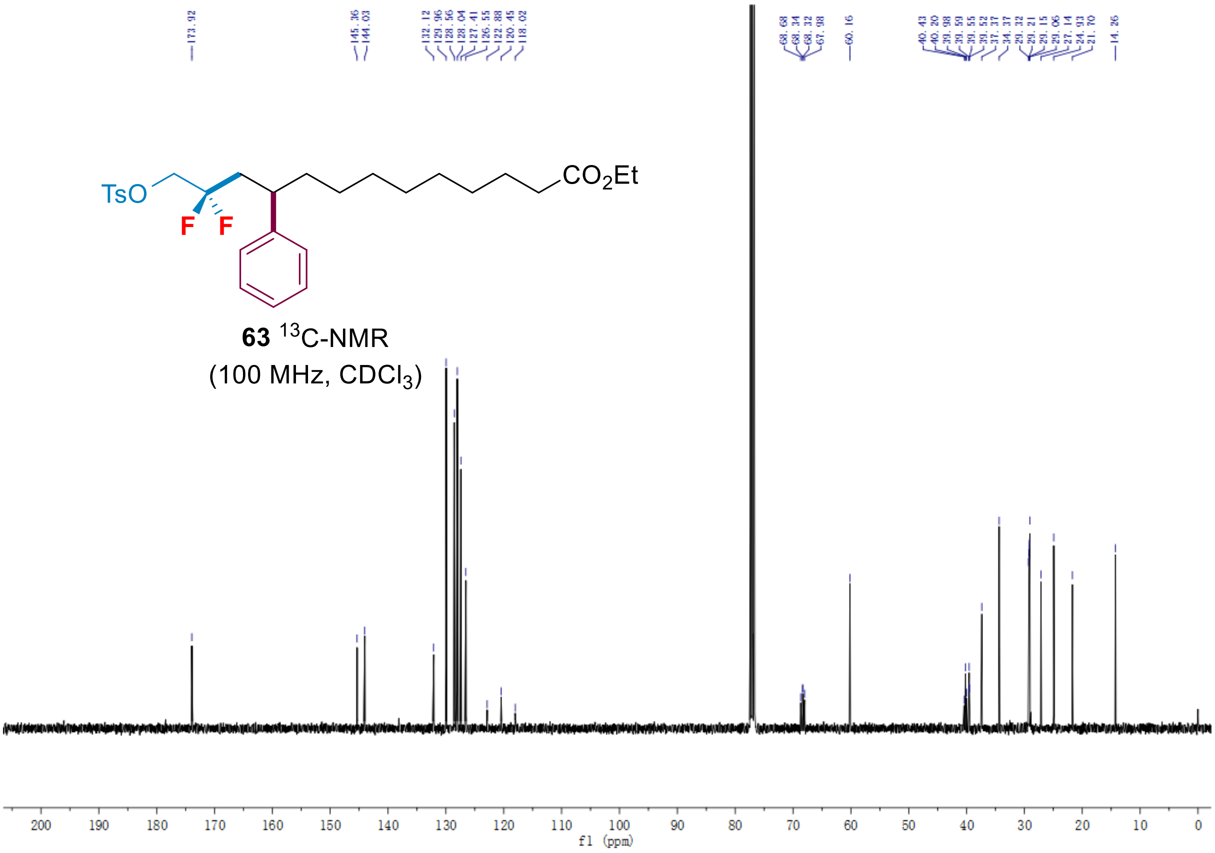

63 ^{13}C-NMR
(100 MHz, $CDCl_3$)

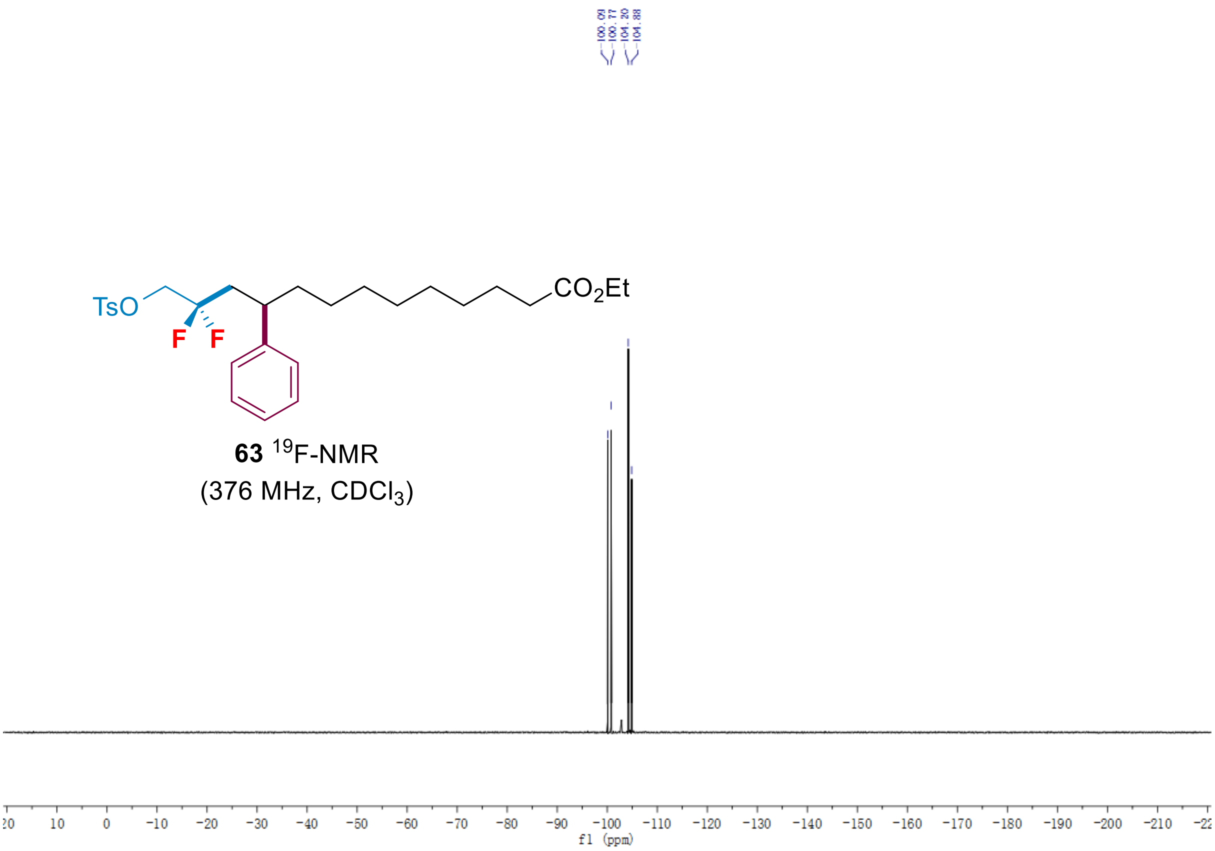

TsO
F
F
CO_2Et
63 ^{19}F-NMR
(376 MHz, $CDCl_3$)
f1 (ppm)

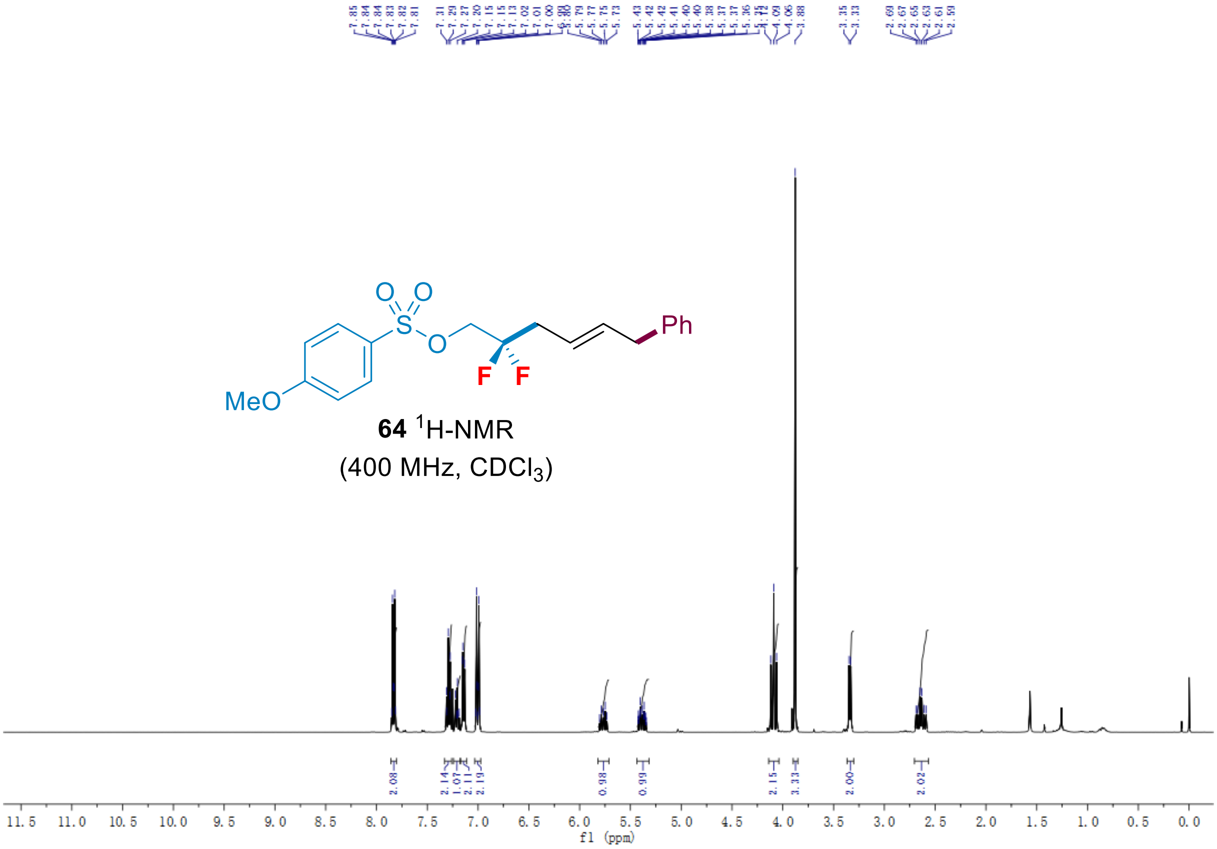

MeO
O
O
S
O
F
F
Ph
64 ^{1}H-NMR
(400 MHz, $CDCl_3$)
f1 (ppm)

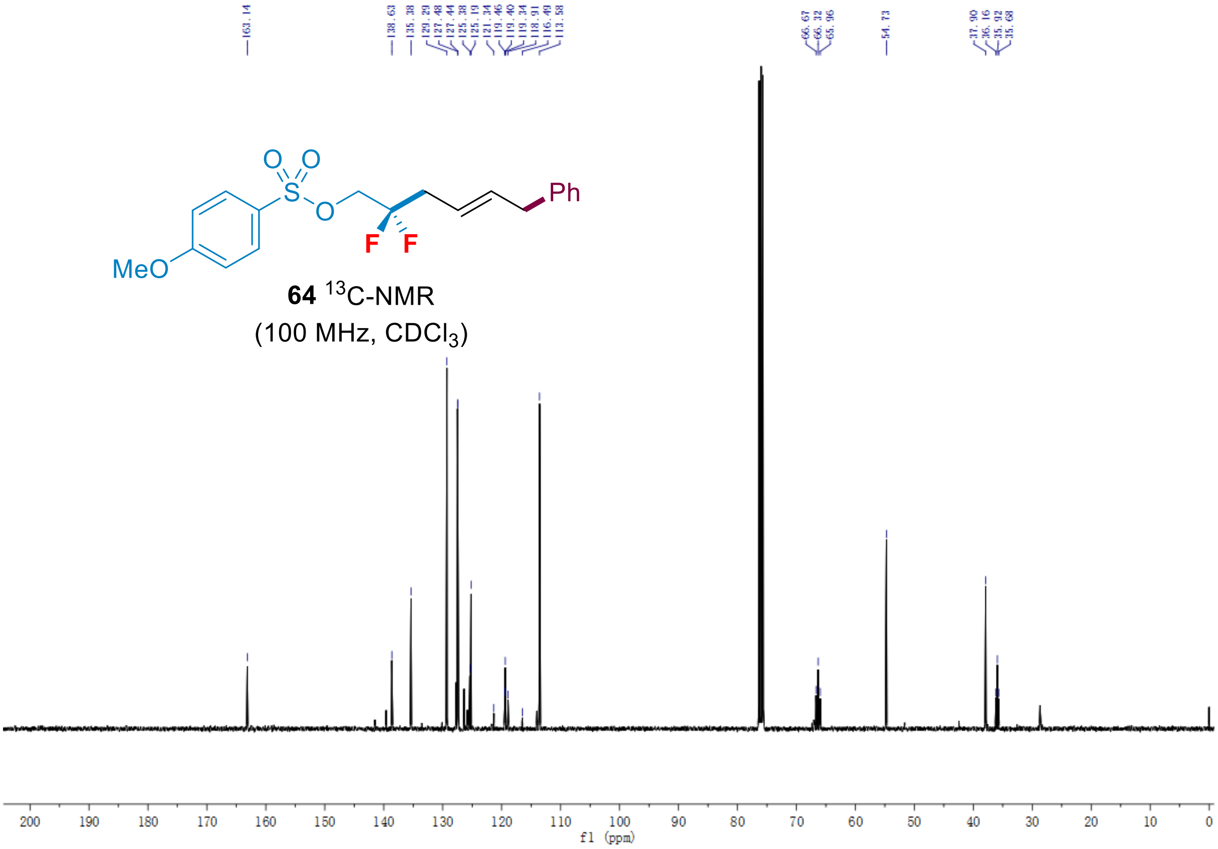

64 ^{13}C-NMR
(100 MHz, $CDCl_3$)

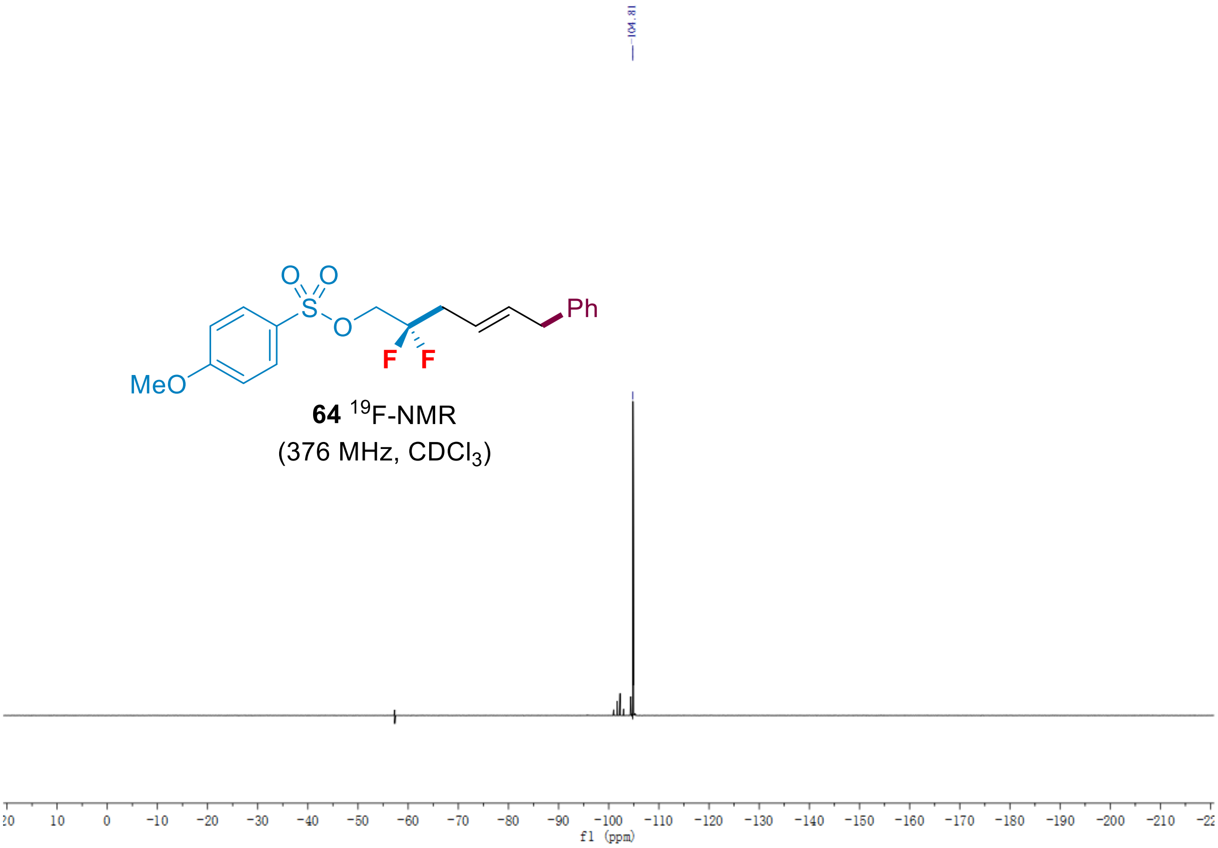

64 ^{19}F-NMR
(376 MHz, $CDCl_3$)

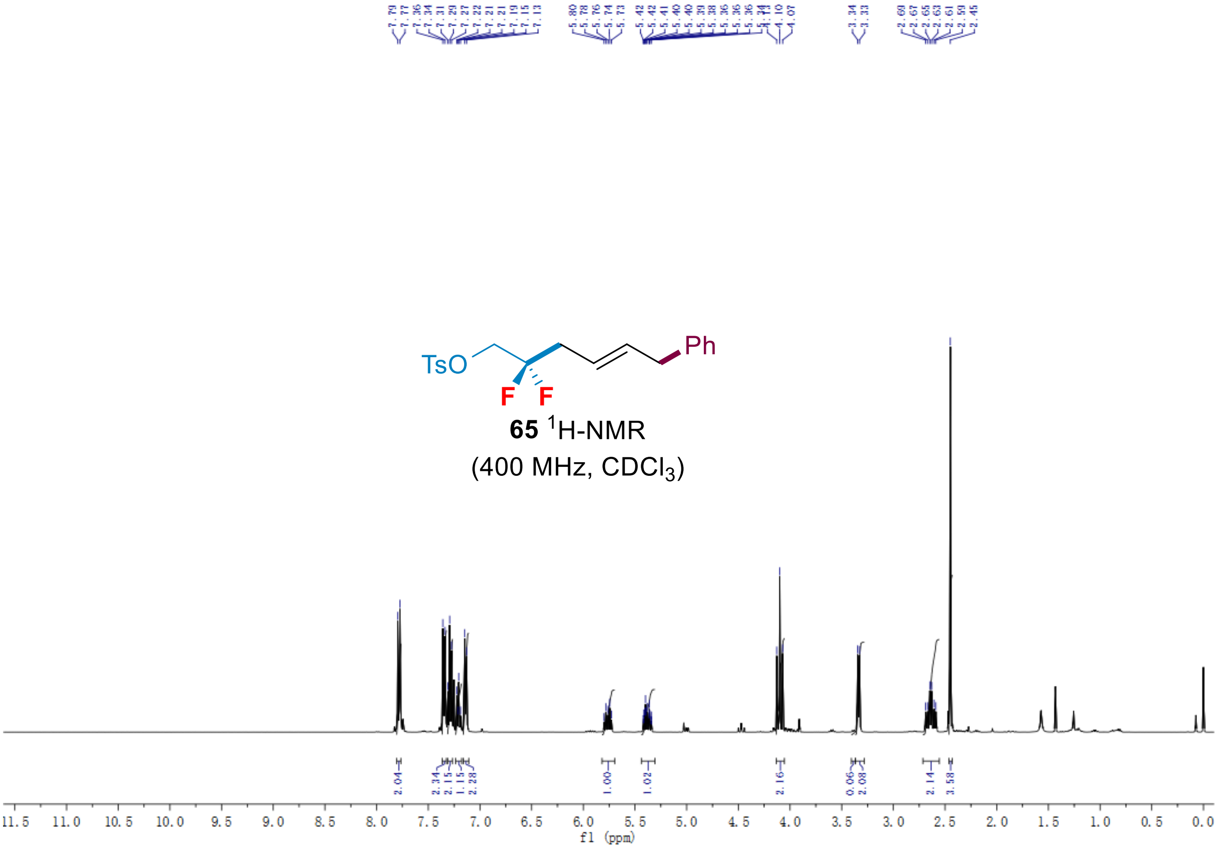
TsO
F F
Ph
65 ¹H-NMR
(400 MHz, CDCl₃)
f1 (ppm)

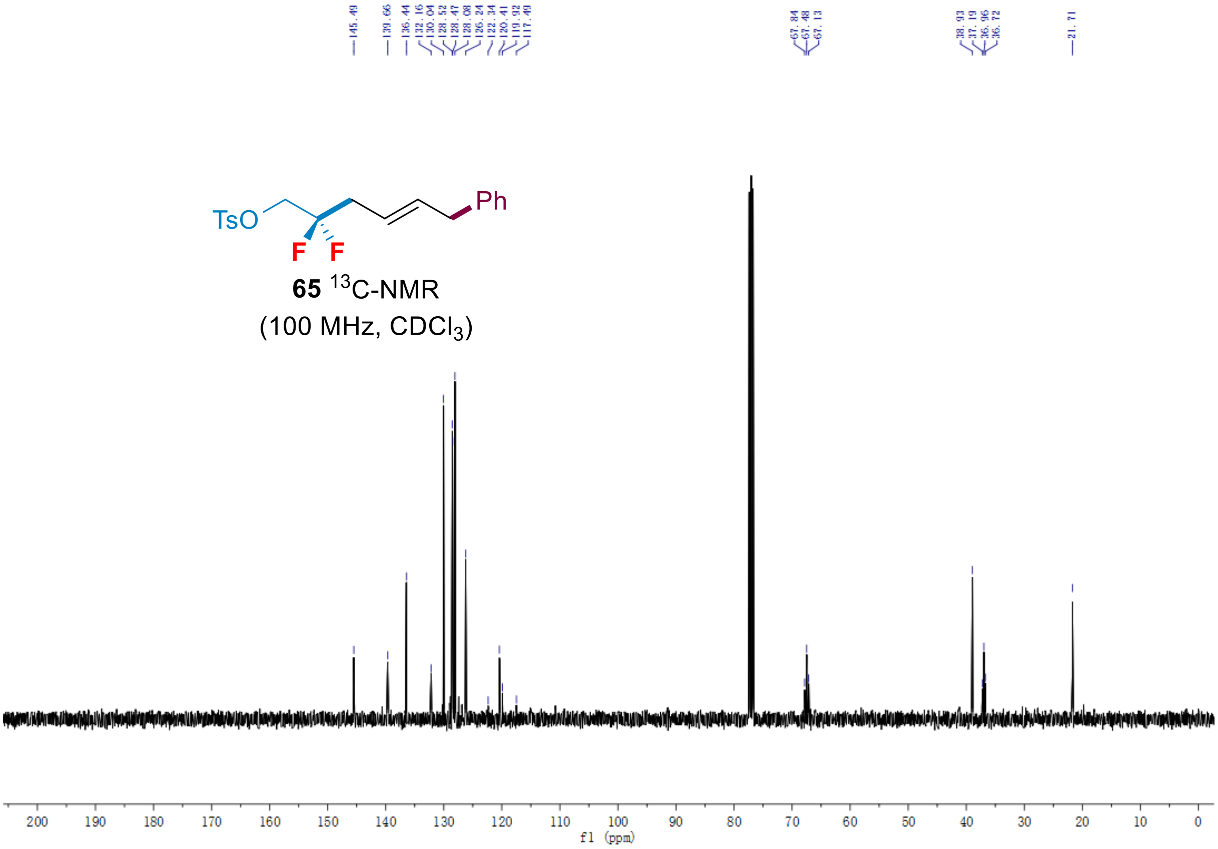
TsO
F F
Ph
65 ¹³C-NMR
(100 MHz, CDCl₃)
f1 (ppm)

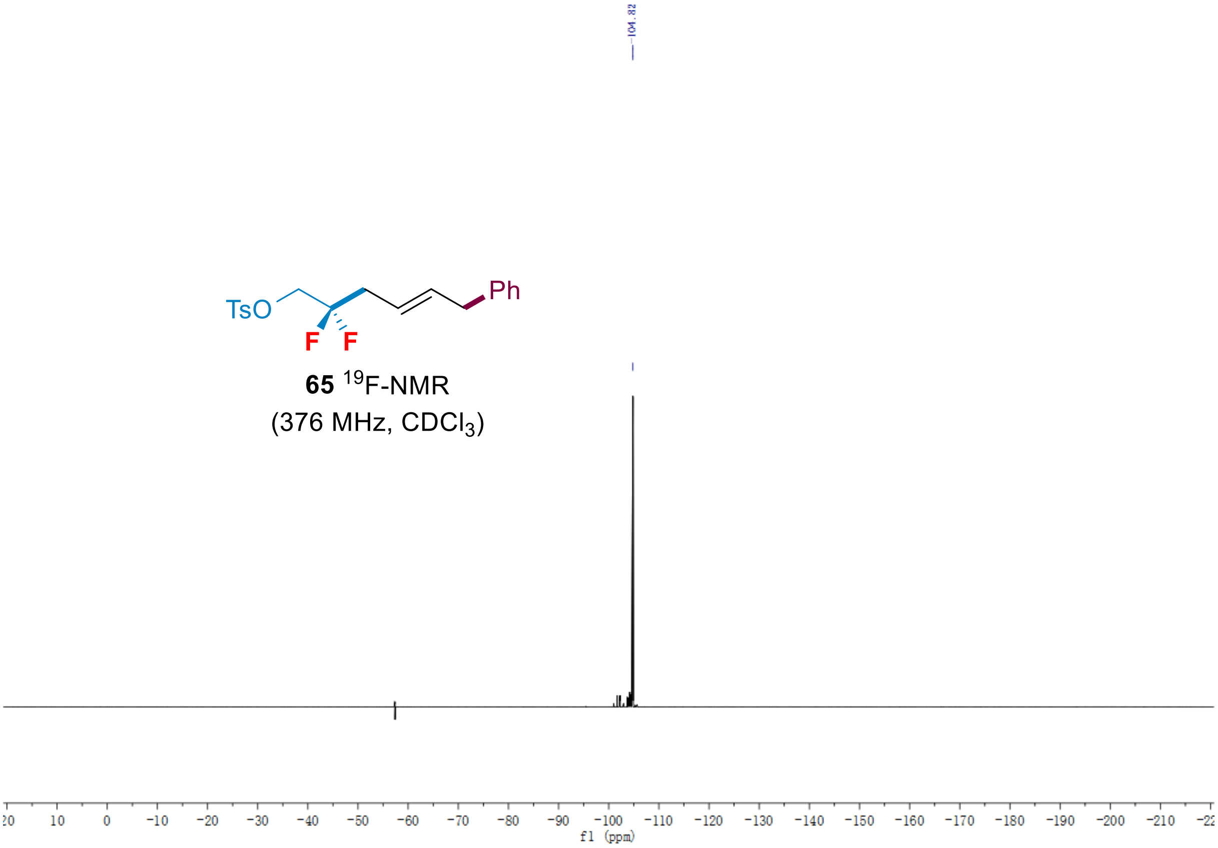

-104.82
TsO
F F
Ph
65 19F-NMR
(376 MHz, CDCl3)
f1 (ppm)

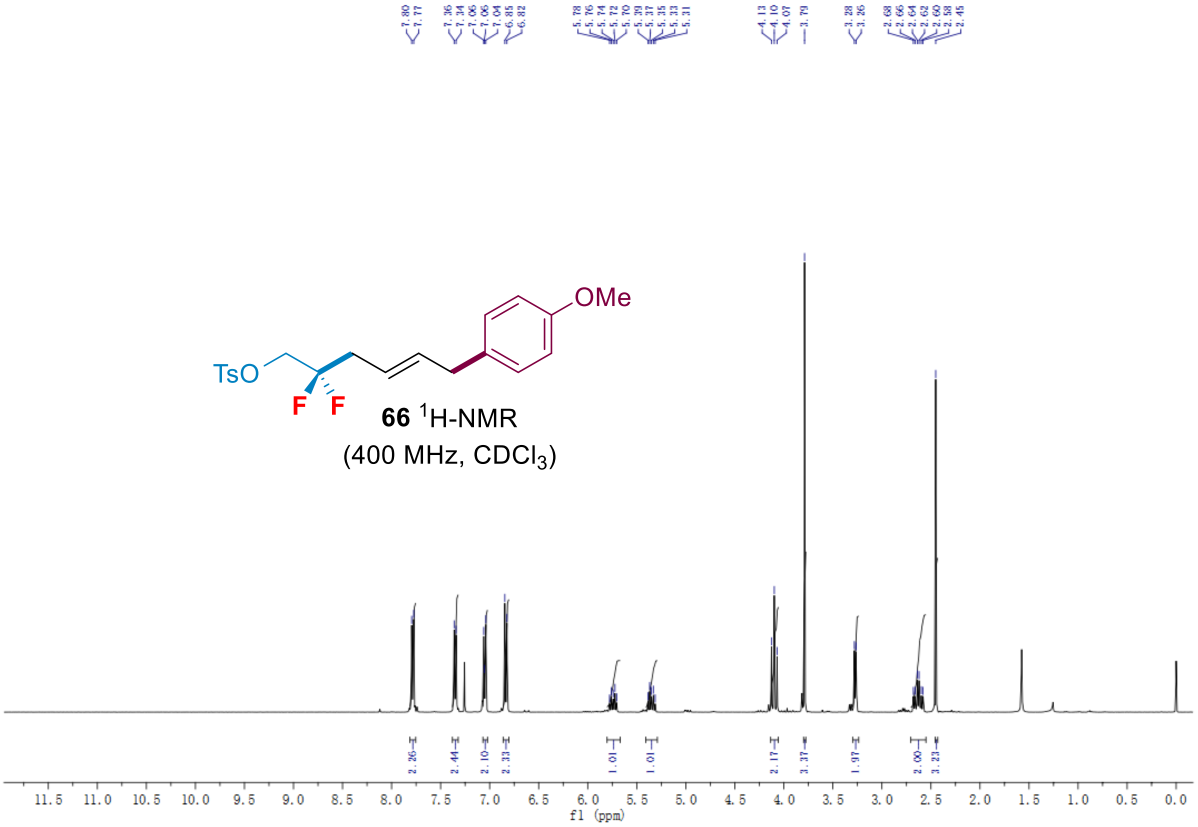

7.80 7.77 7.36 7.34 7.06 7.06 7.04 6.85 6.82 5.78 5.76 5.74 5.72 5.70 5.39 5.37 5.35 5.33 5.31 4.13 4.10 4.07 3.79 3.28 3.26 2.68 2.66 2.64 2.62 2.60 2.58 2.45
TsO
F F
OMe
66 1H-NMR
(400 MHz, CDCl3)
2.26 2.44 2.10 2.33 1.01 1.01 2.17 3.37 1.97 2.00 3.23
f1 (ppm)

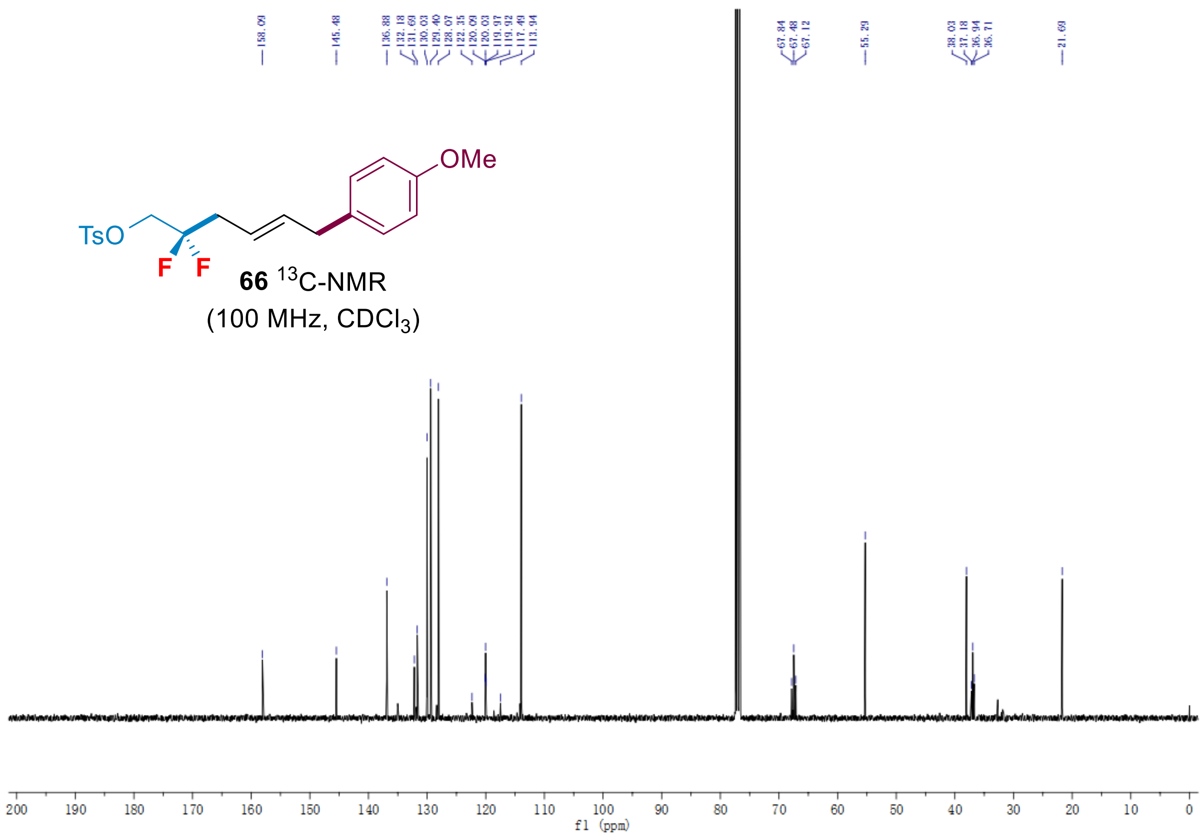
158.09
145.48
136.88
132.18
131.69
130.03
129.40
128.07
122.35
120.09
120.03
119.97
119.92
117.49
113.94
67.84
67.48
67.12
55.29
38.03
37.18
36.94
36.71
21.69
TsO
OMe
F
F
66 13C-NMR
(100 MHz, CDCl3)
f1 (ppm)

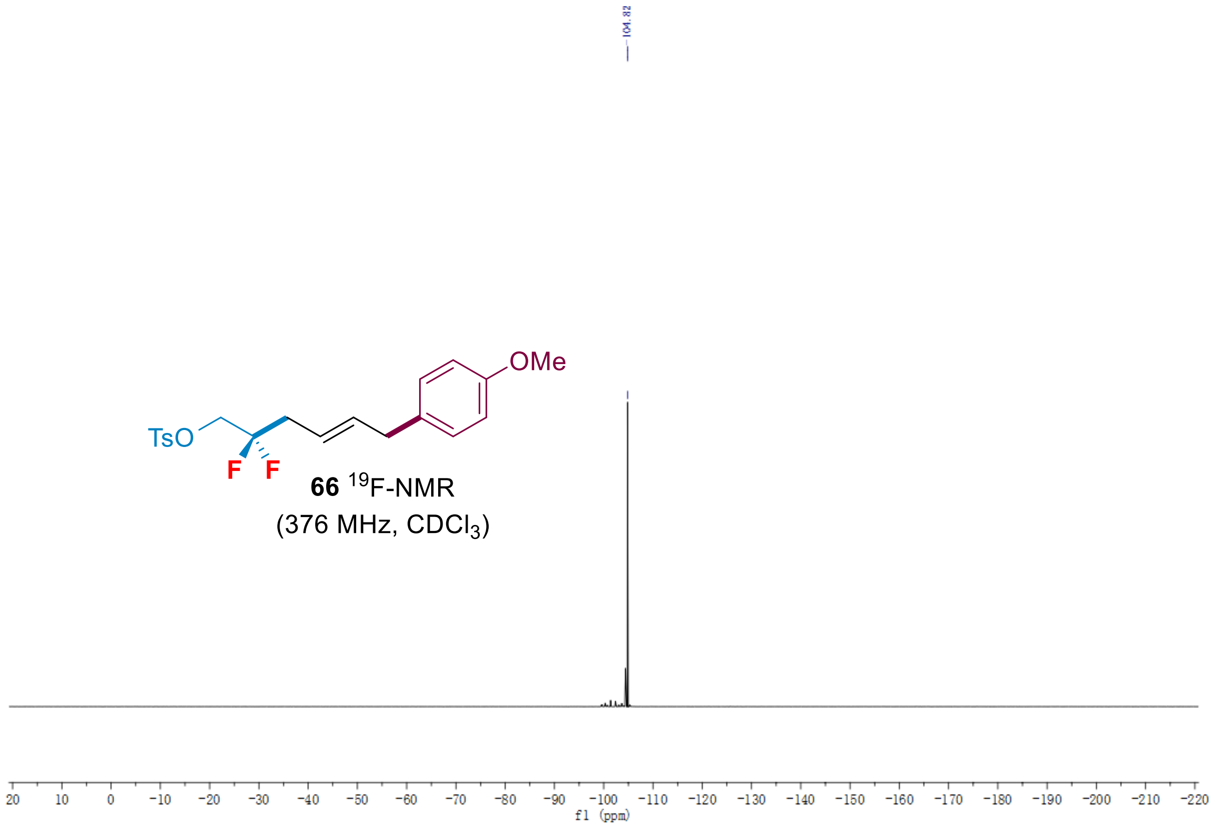
-104.82
TsO
OMe
F
F
66 19F-NMR
(376 MHz, CDCl3)
f1 (ppm)

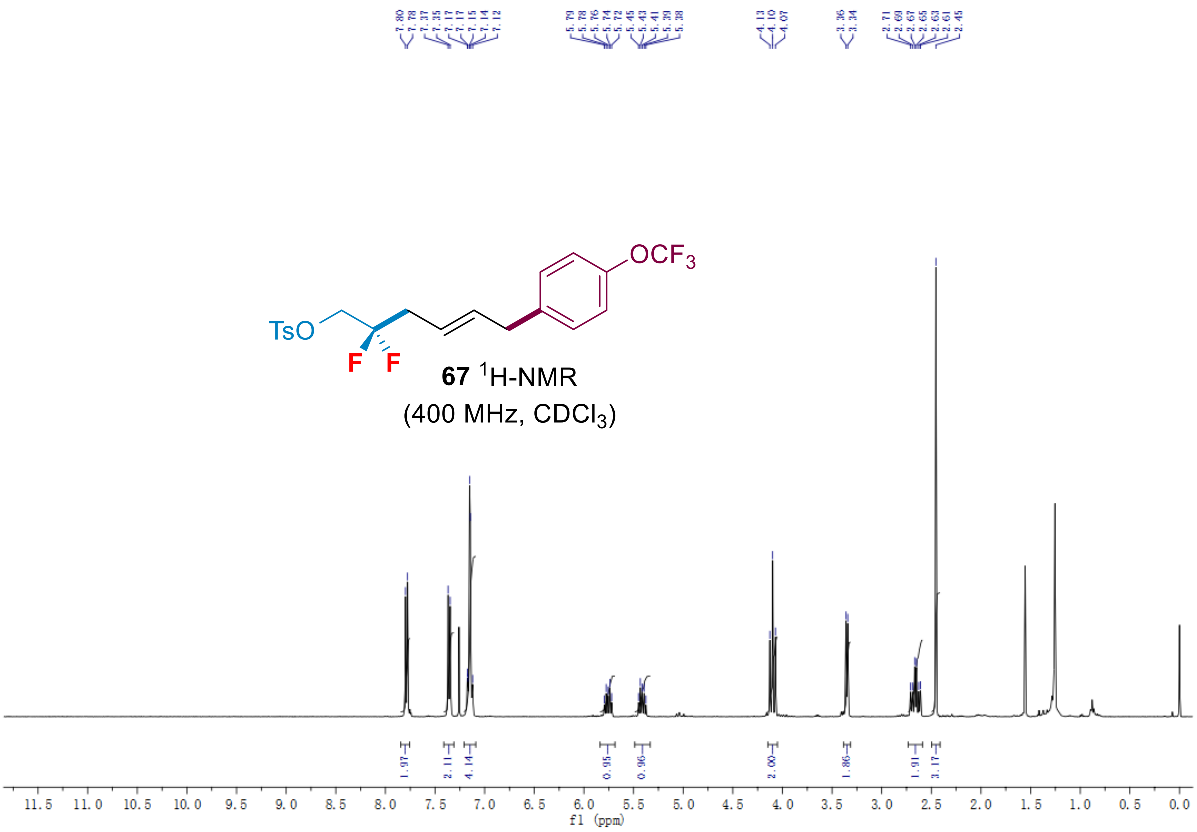
7.80 7.78 7.37 7.35 7.17 7.17 7.15 7.14 7.12
5.79 5.78 5.76 5.74 5.72 5.45 5.43 5.41 5.39 5.38
4.13 4.10 4.07
3.36 3.34
2.71 2.69 2.67 2.65 2.63 2.61 2.45
OCF3
TsO
F F
67 1H-NMR
(400 MHz, CDCl3)
1.97 2.11 4.14 0.95 0.96 2.00 1.86 1.91 3.17
11.5 11.0 10.5 10.0 9.5 9.0 8.5 8.0 7.5 7.0 6.5 6.0 5.5 5.0 4.5 4.0 3.5 3.0 2.5 2.0 1.5 1.0 0.5 0.0
f1 (ppm)

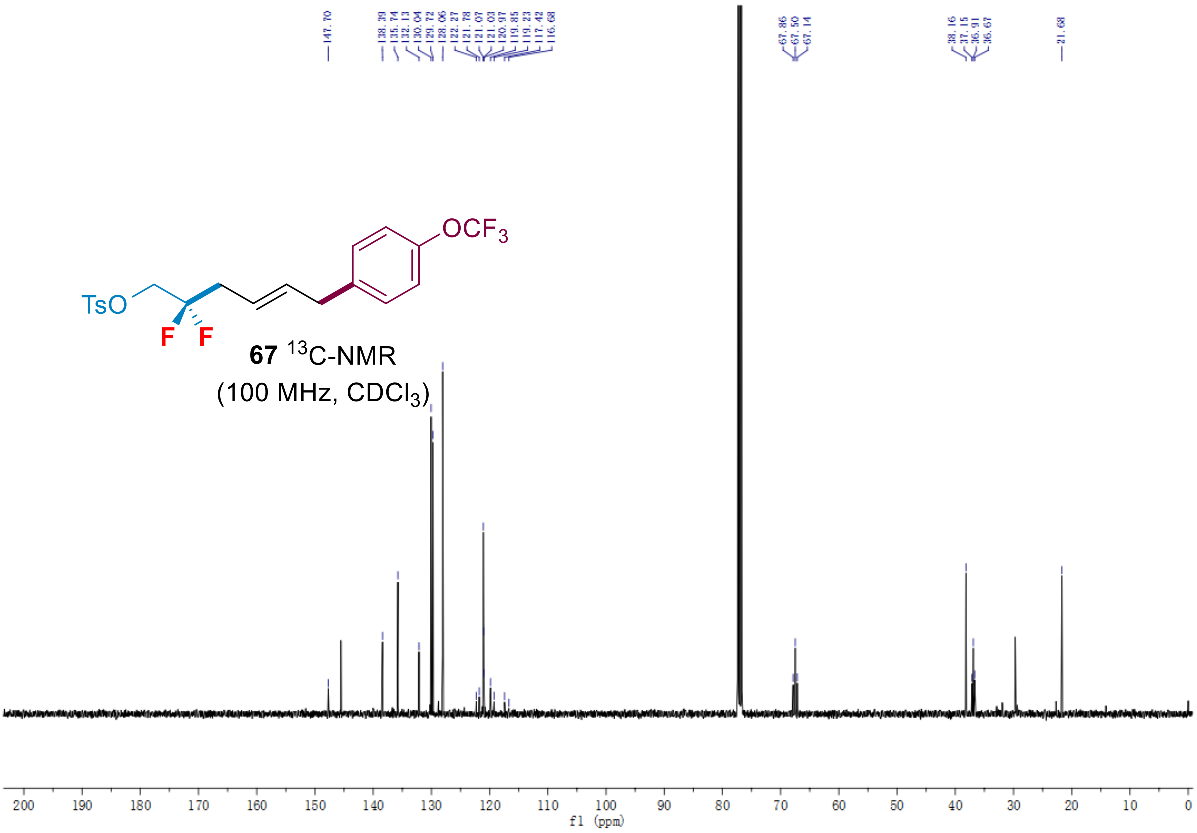
147.70
138.39 135.74 132.13 130.04 129.72 128.06 122.27 121.78 121.07 121.03 120.37 119.85 119.23 117.42 116.68
67.86 67.50 67.14
38.16 37.15 36.91 36.67
21.68
OCF3
TsO
F F
67 13C-NMR
(100 MHz, CDCl3)
200 190 180 170 160 150 140 130 120 110 100 90 80 70 60 50 40 30 20 10 0
f1 (ppm)

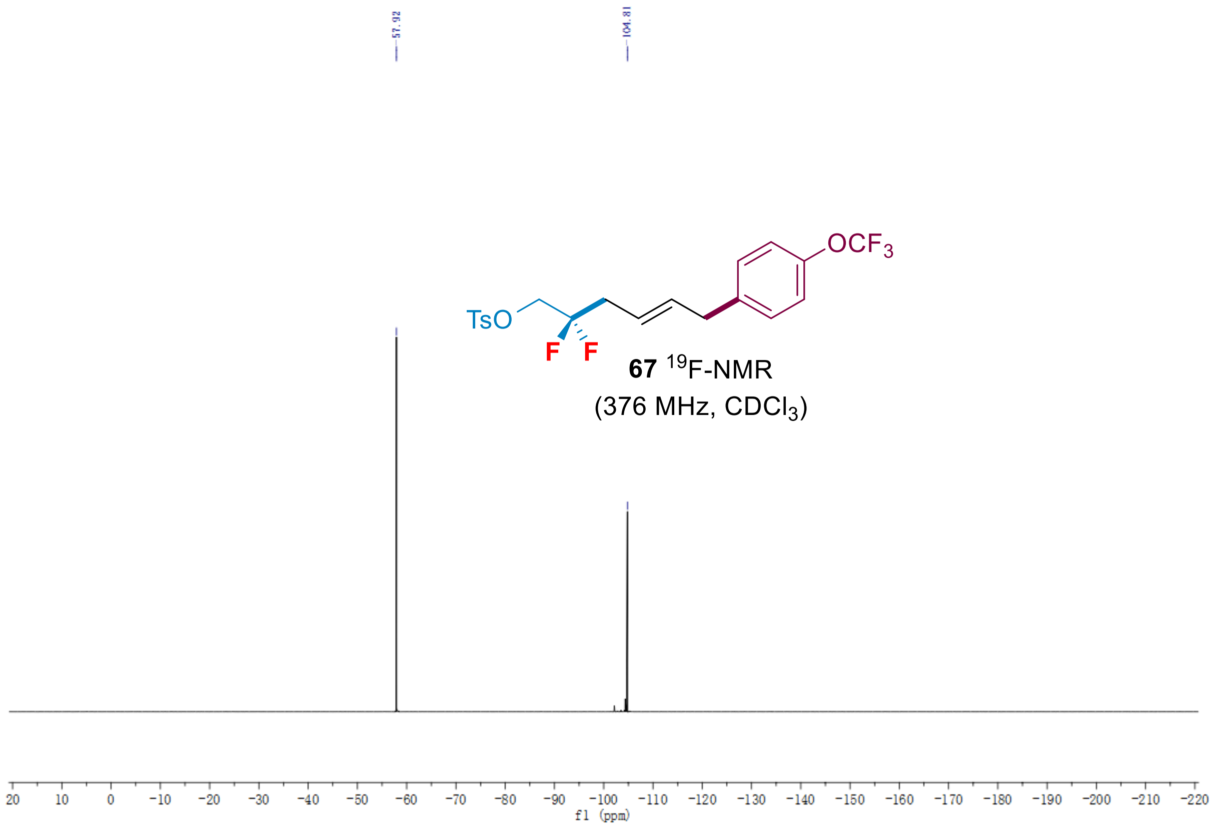
-57.92
-104.81
OCF3
TsO
F F
67 19F-NMR
(376 MHz, CDCl3)
f1 (ppm)

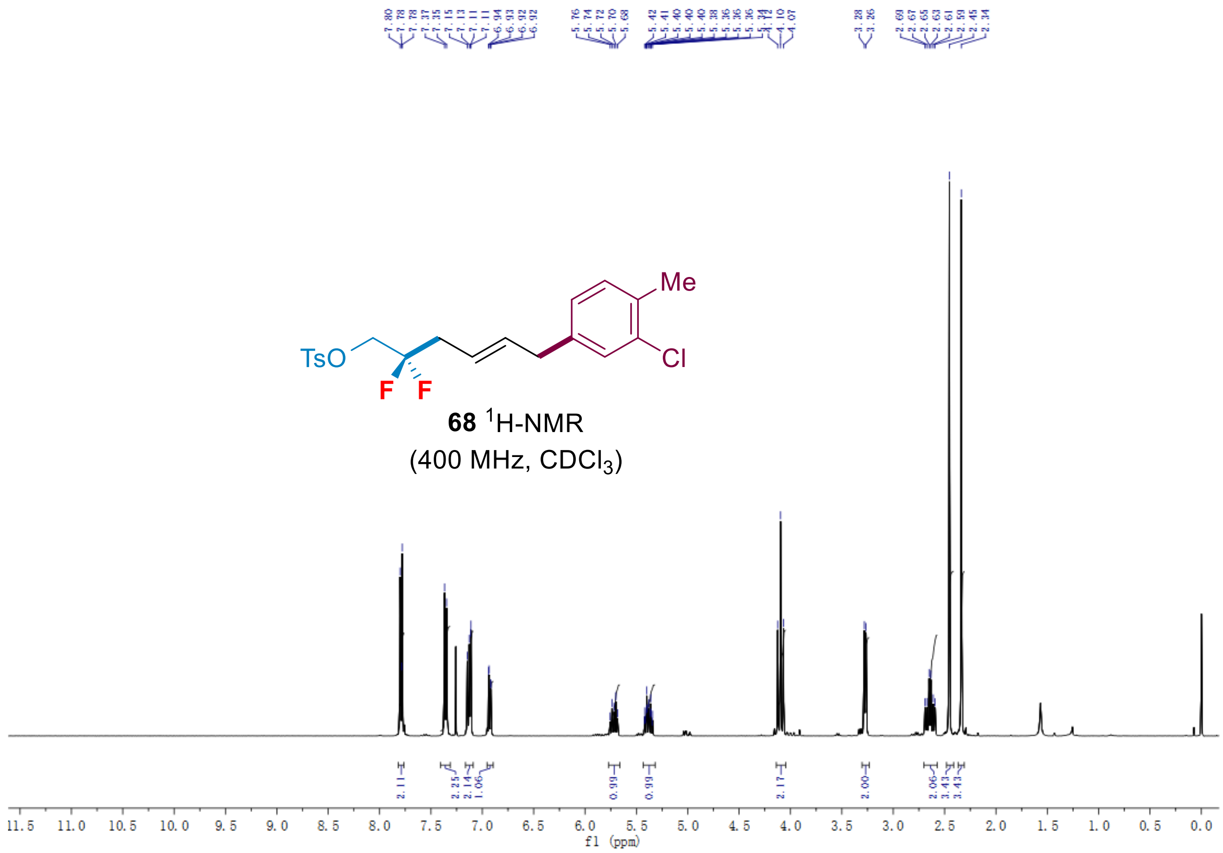
Me
TsO
Cl
F F
68 1H-NMR
(400 MHz, CDCl3)
f1 (ppm)

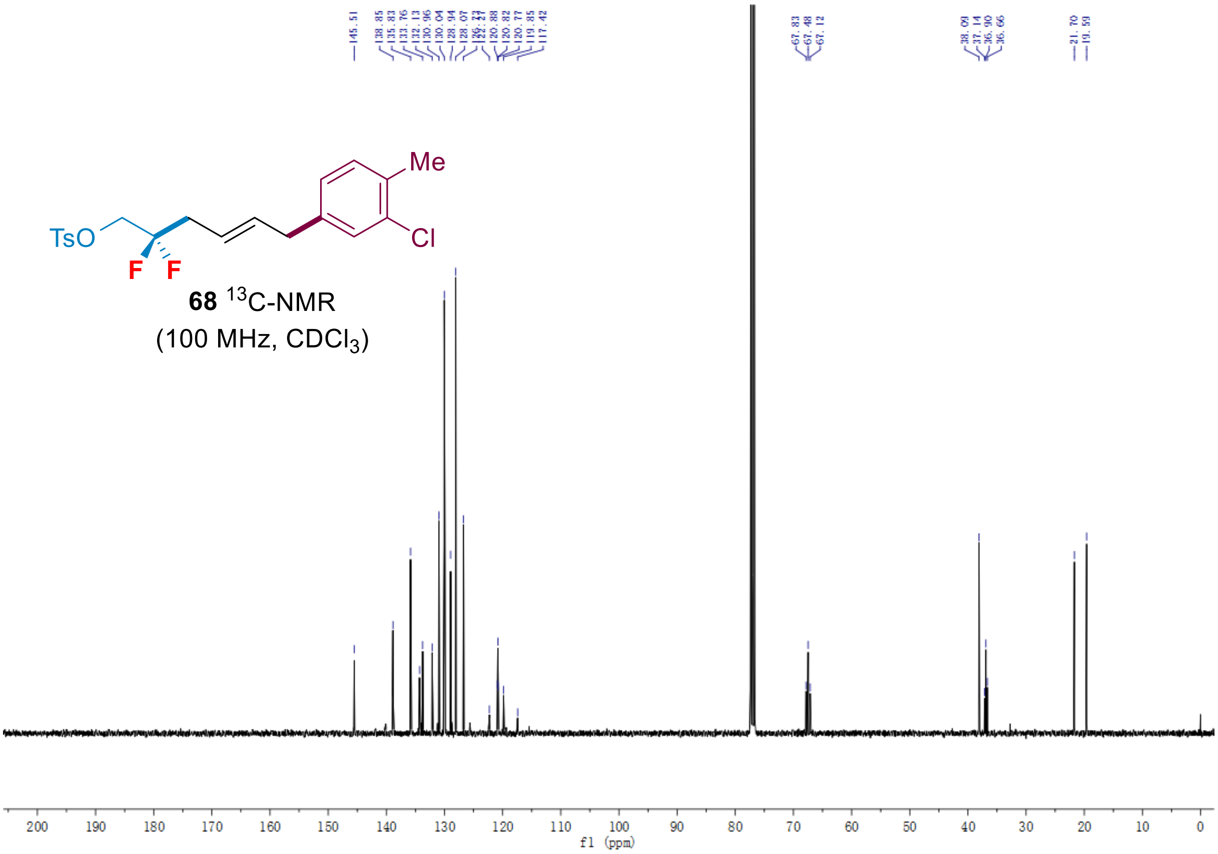

145.51
138.85
135.83
133.76
132.13
130.96
130.04
128.94
128.07
120.88
120.82
120.77
119.85
117.42
67.83
67.48
67.12
38.09
37.14
36.90
36.66
21.70
19.59
TsO
F
F
Me
Cl
68 ^{13}C-NMR
(100 MHz, $CDCl_3$)
f1 (ppm)

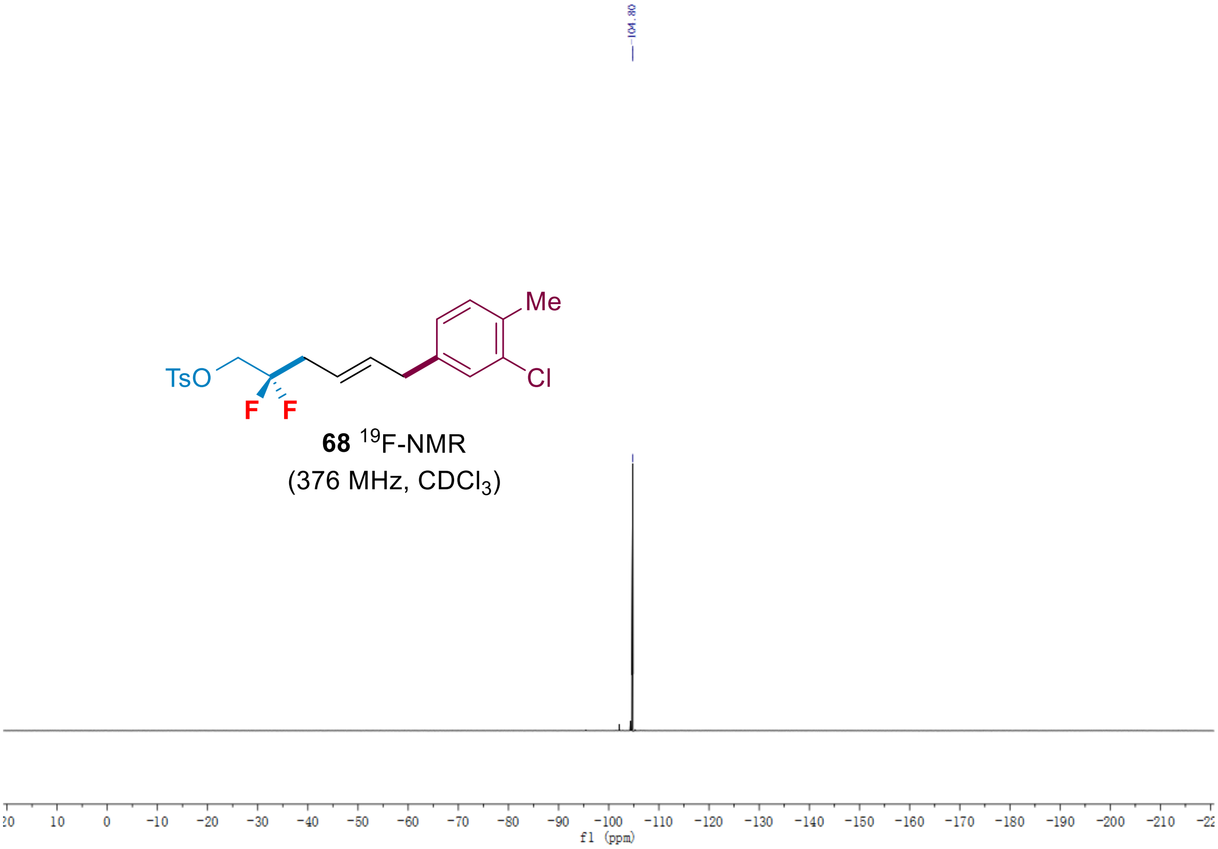

-104.80
TsO
F
F
Me
Cl
68 ^{19}F-NMR
(376 MHz, $CDCl_3$)
f1 (ppm)

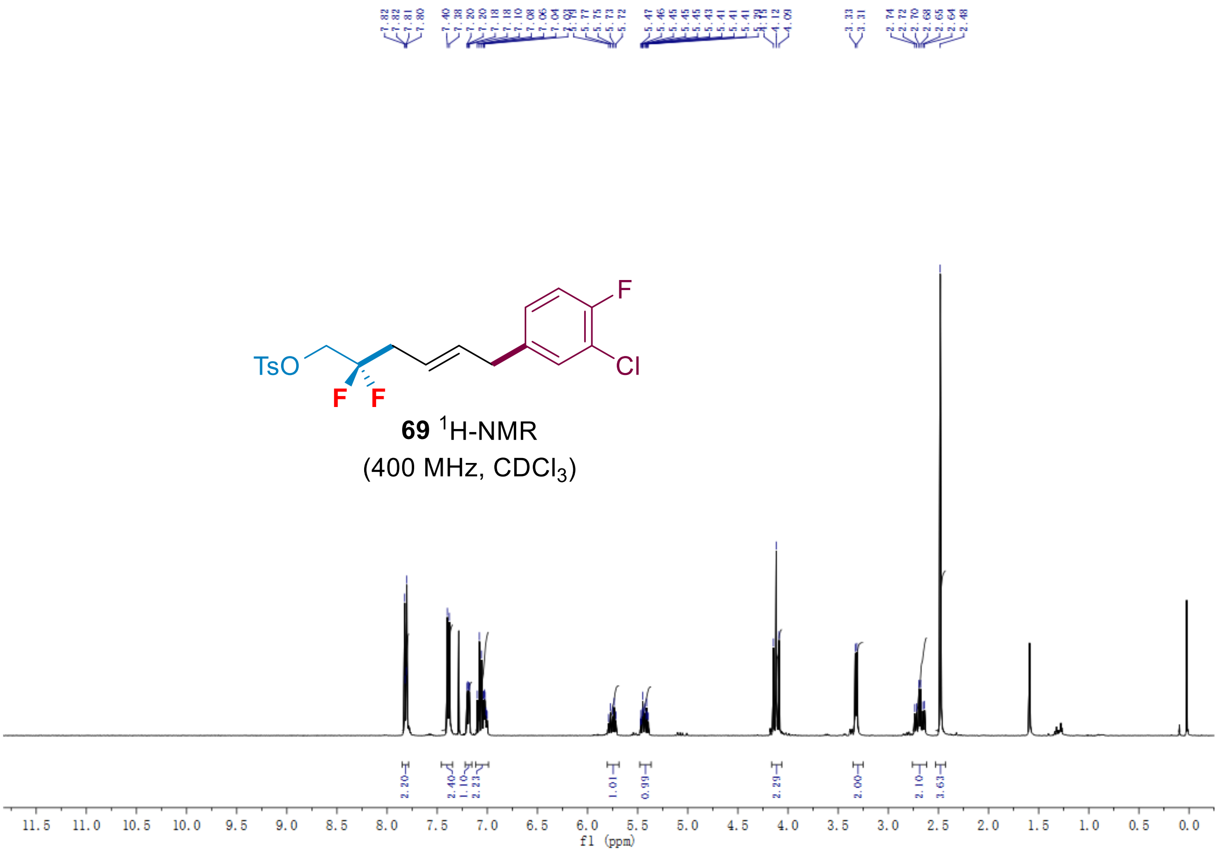
TsO
F
F
F
Cl
69 1H-NMR
(400 MHz, CDCl3)
f1 (ppm)

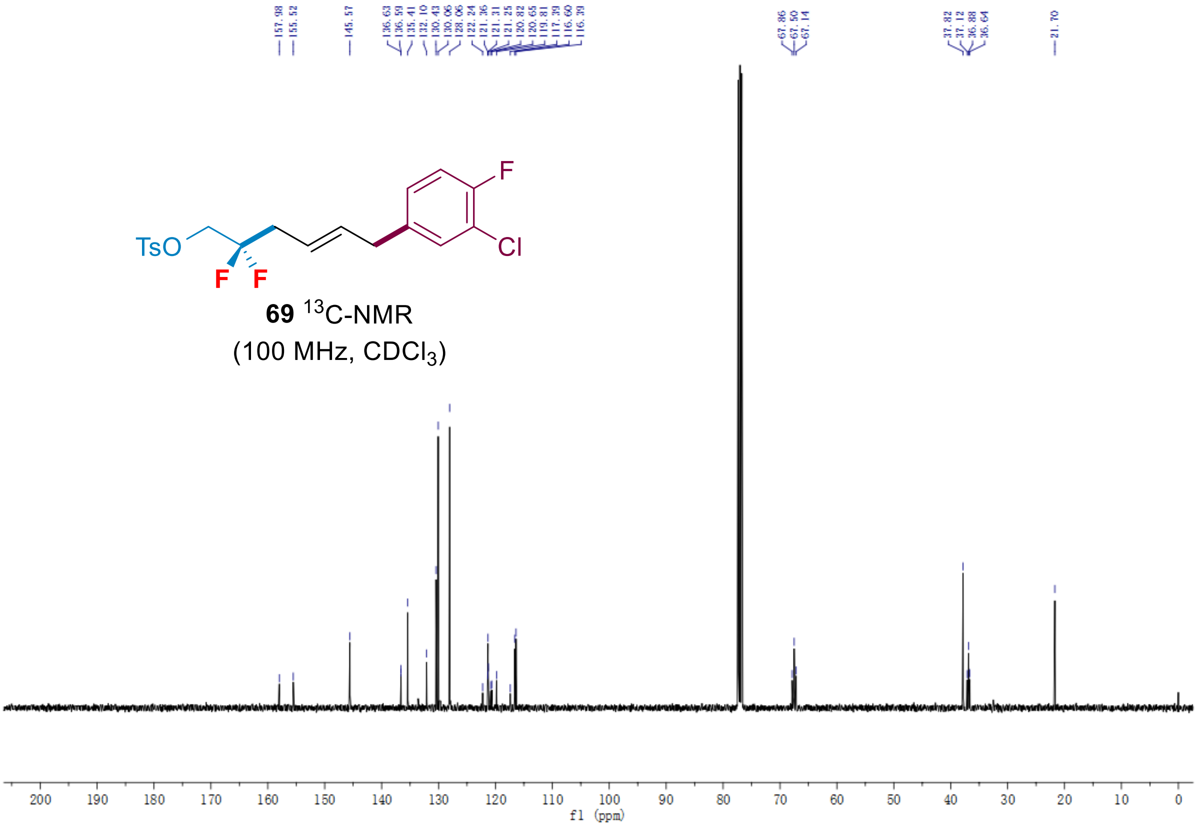
TsO
F
F
F
Cl
69 13C-NMR
(100 MHz, CDCl3)
f1 (ppm)

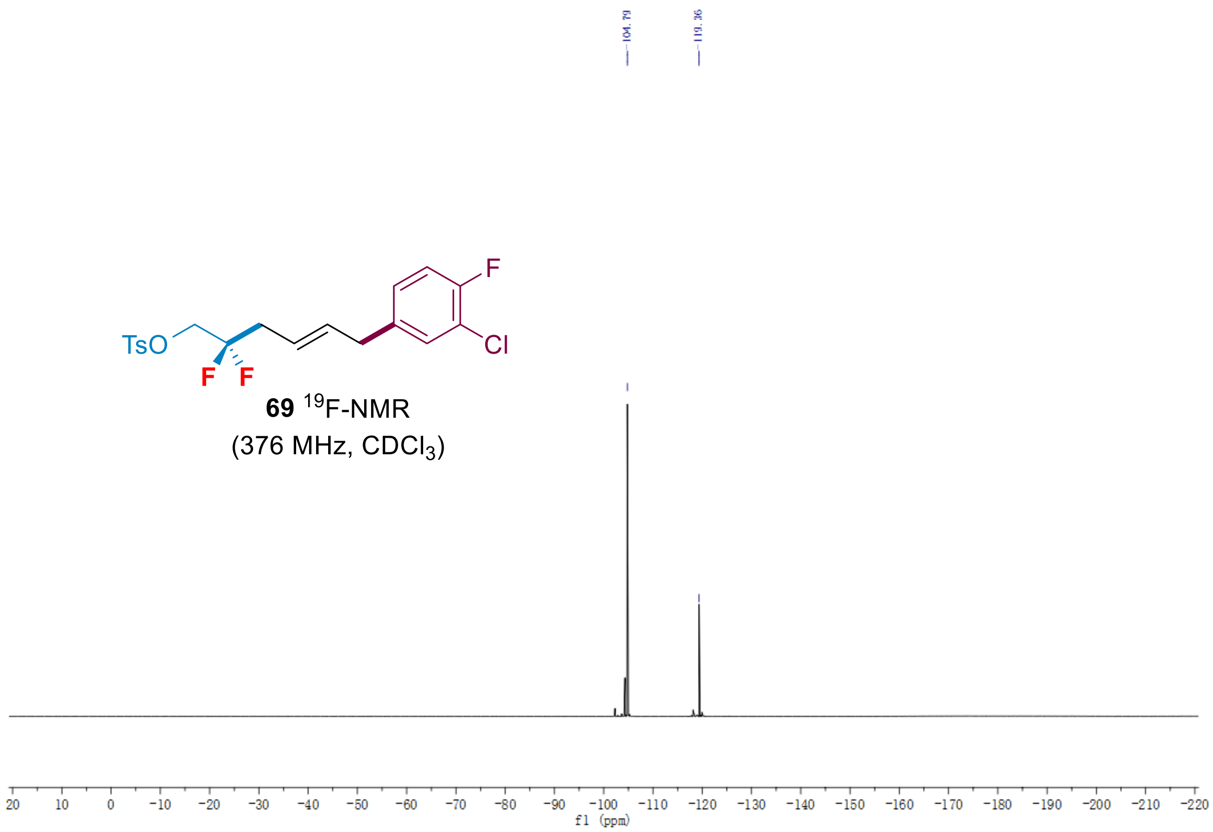
TsO
F F
F
Cl
69 ^{19}F-NMR
(376 MHz, $CDCl_3$)
-104.79
-119.36
f1 (ppm)

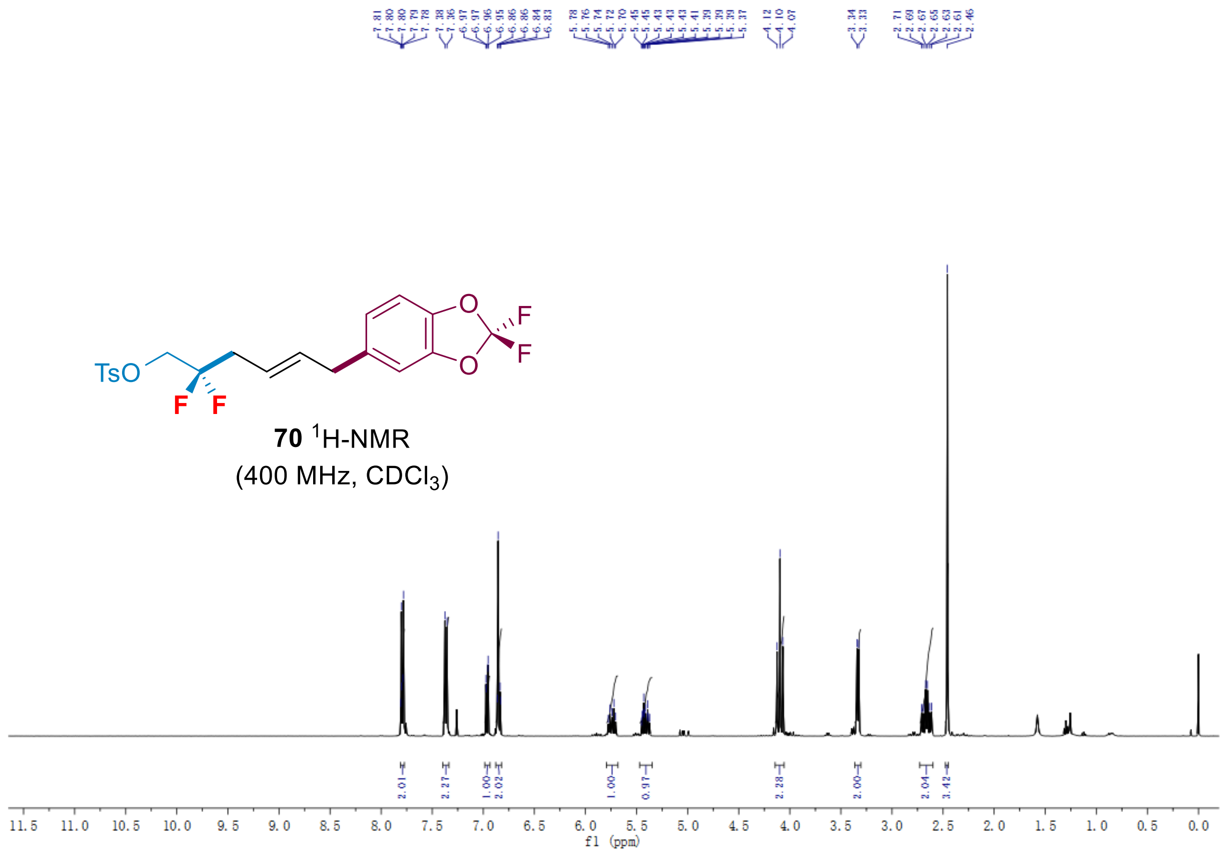
TsO
F F
O
O
F
F
70 ^{1}H-NMR
(400 MHz, $CDCl_3$)
f1 (ppm)

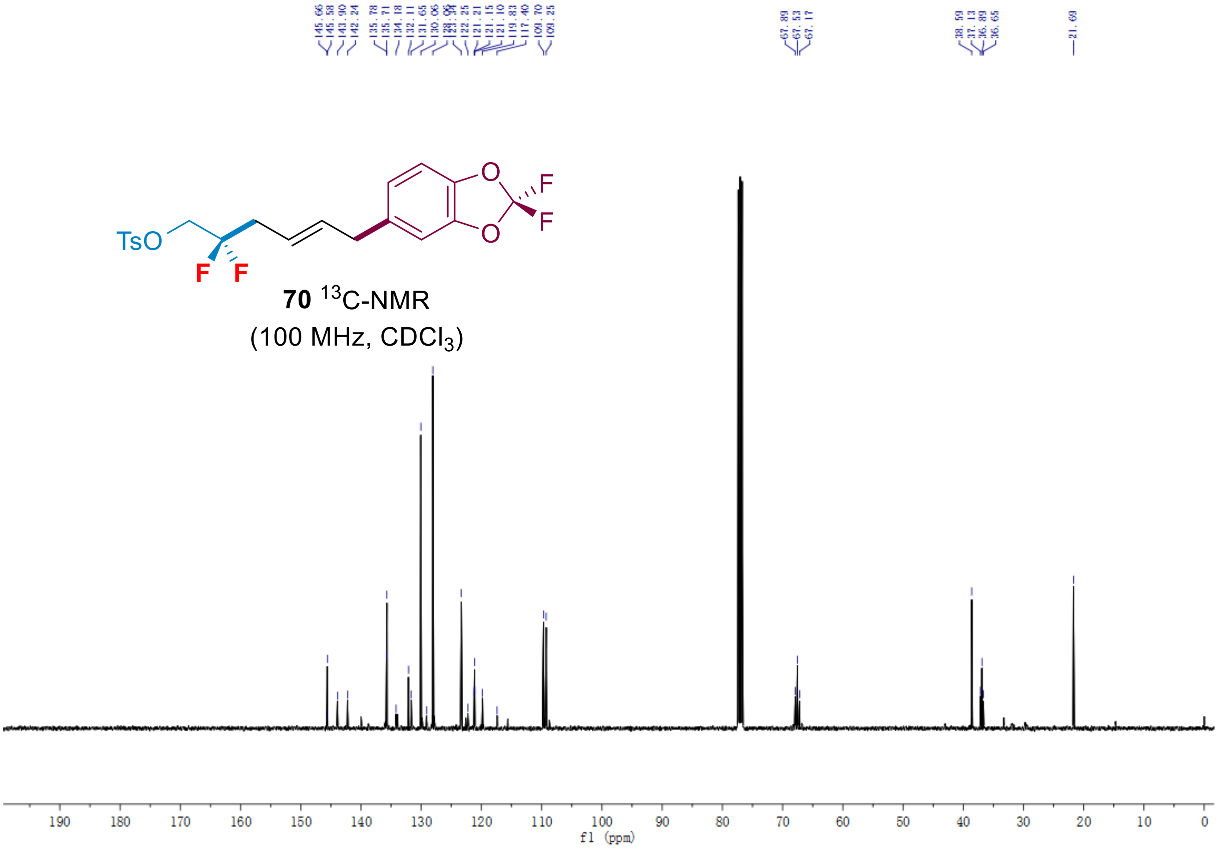
TsO
F
F
O
O
F
F
70 13C-NMR
(100 MHz, CDCl3)
f1 (ppm)

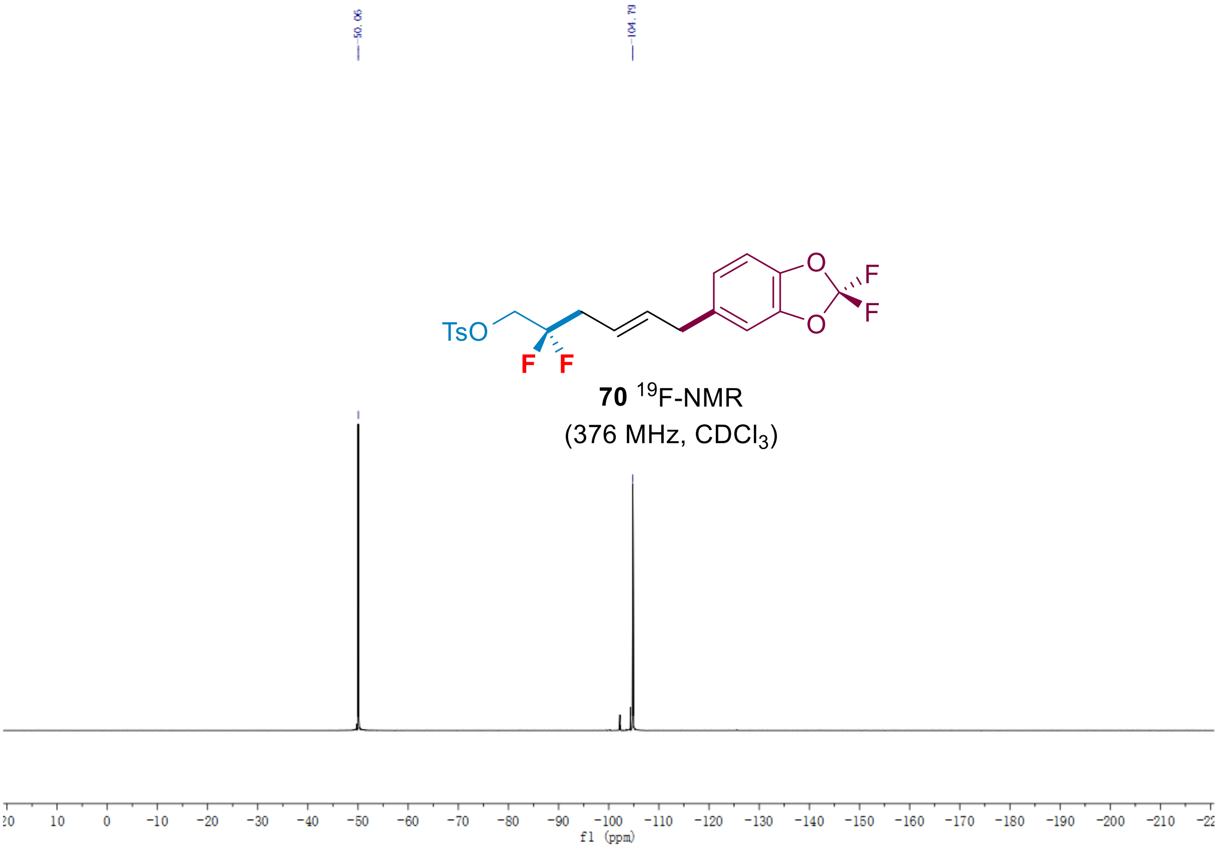
TsO
F
F
O
O
F
F
70 19F-NMR
(376 MHz, CDCl3)
f1 (ppm)

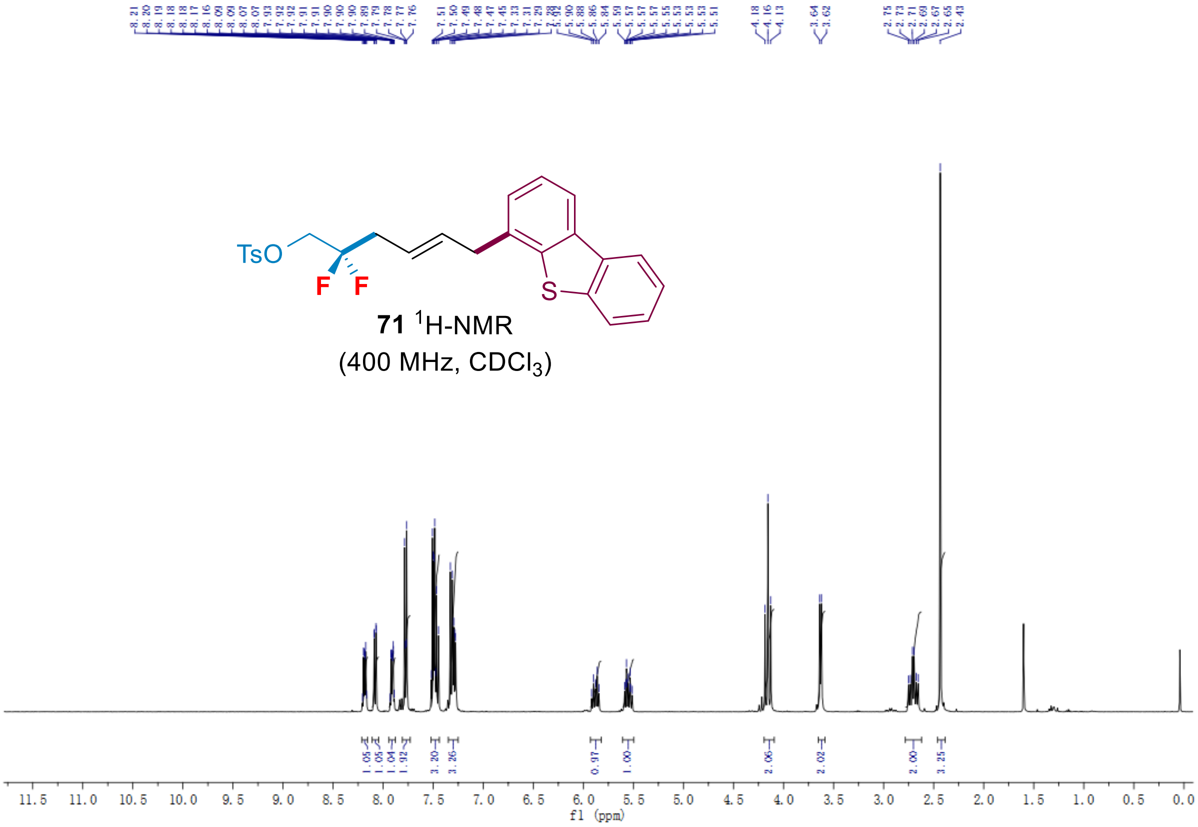
TsO
F F
S
71 ¹H-NMR
(400 MHz, CDCl3)
f1 (ppm)

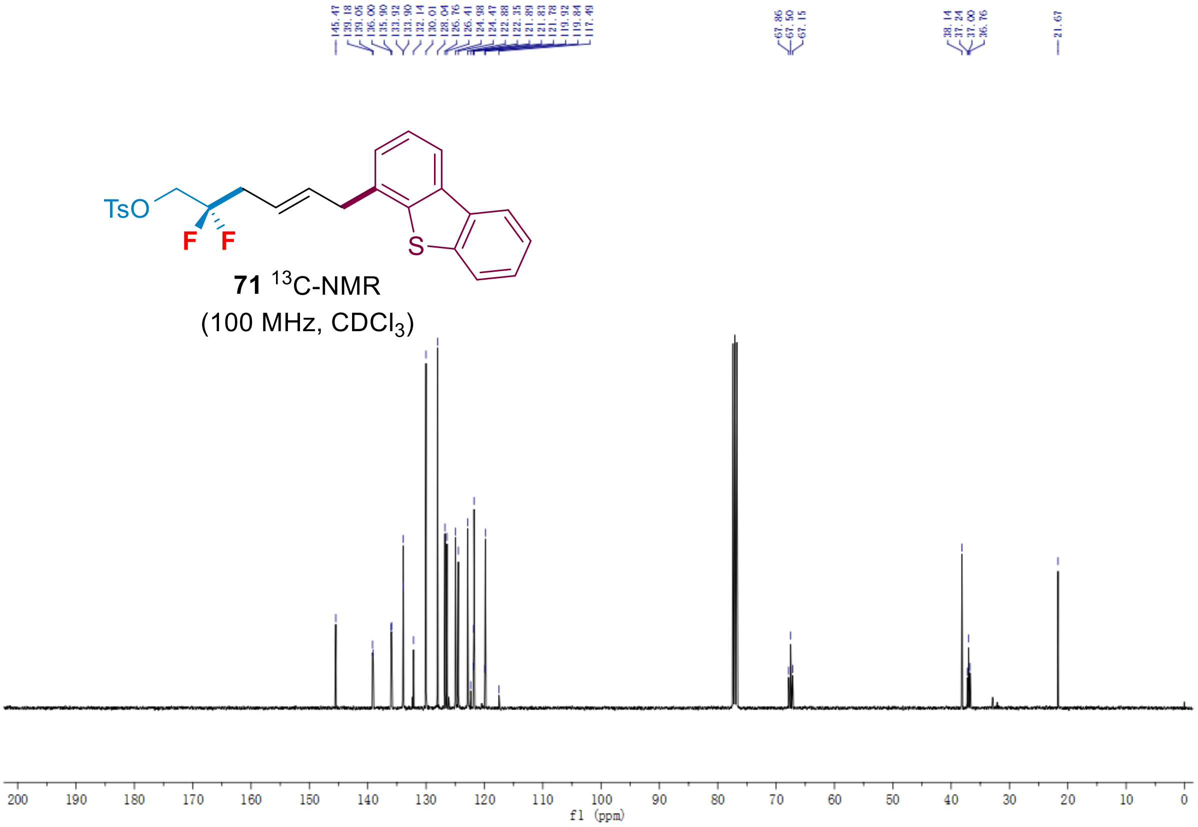
TsO
F F
S
71 ¹³C-NMR
(100 MHz, CDCl3)
f1 (ppm)

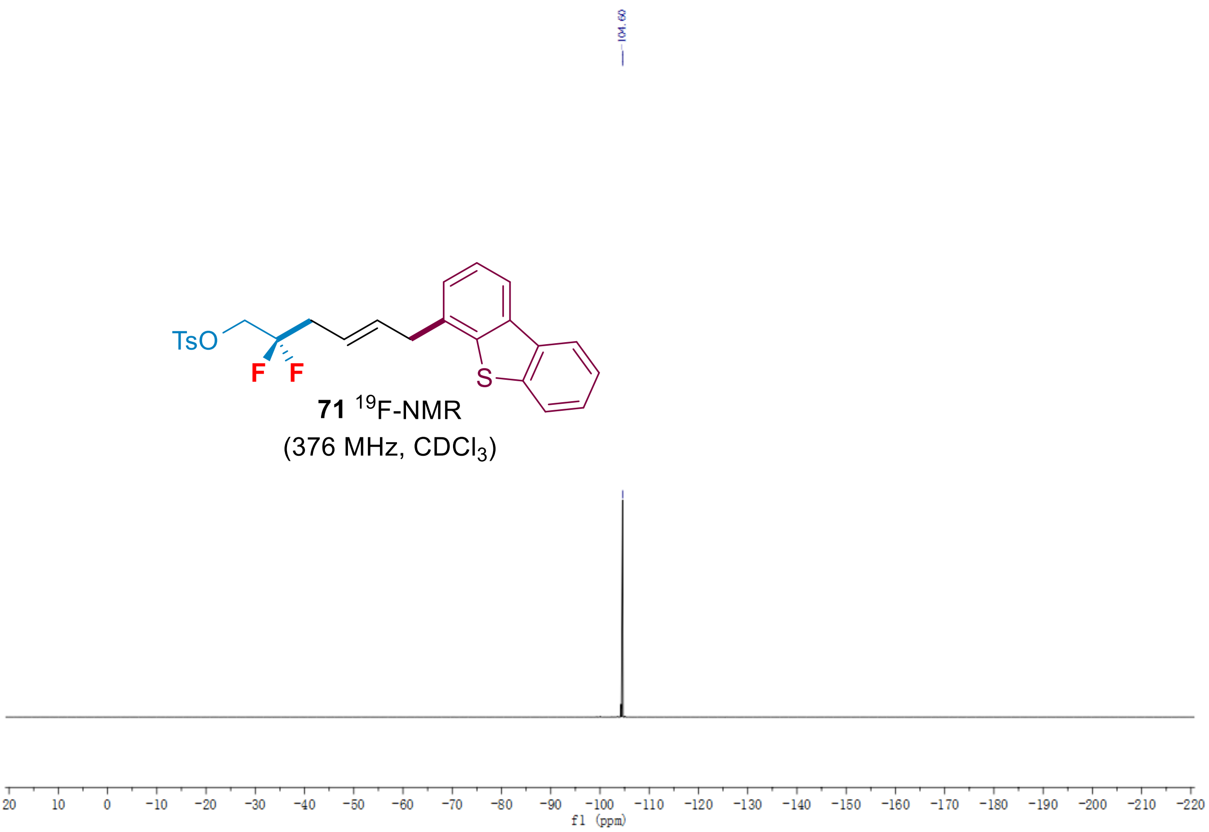

-104.60
TsO
F F
S
71 ^{19}F-NMR
(376 MHz, $CDCl_3$)
20 10 0 −10 −20 −30 −40 −50 −60 −70 −80 −90 −100 −110 −120 −130 −140 −150 −160 −170 −180 −190 −200 −210 −220
f1 (ppm)

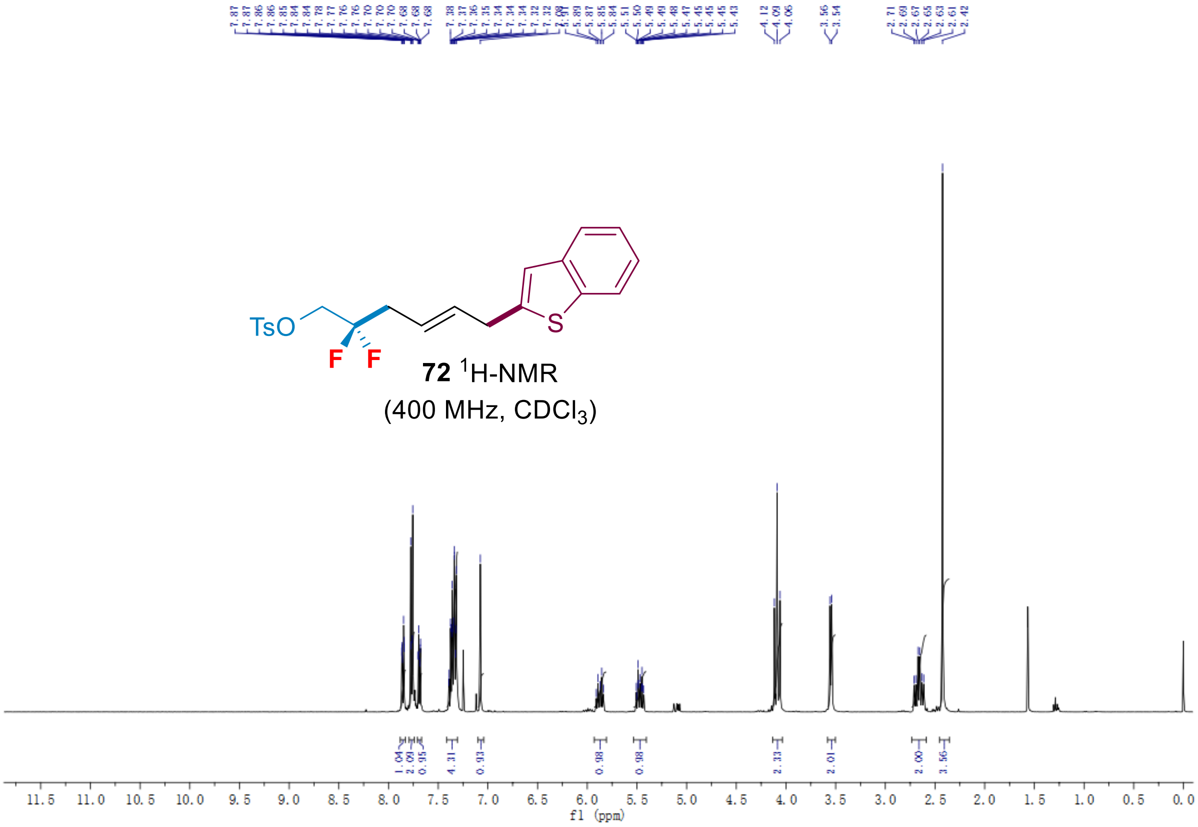

TsO
F F
S
72 ^{1}H-NMR
(400 MHz, $CDCl_3$)
1.04 2.09 0.95 4.31 0.93 0.98 0.98 2.33 2.01 2.00 3.56
11.5 11.0 10.5 10.0 9.5 9.0 8.5 8.0 7.5 7.0 6.5 6.0 5.5 5.0 4.5 4.0 3.5 3.0 2.5 2.0 1.5 1.0 0.5 0.0
f1 (ppm)

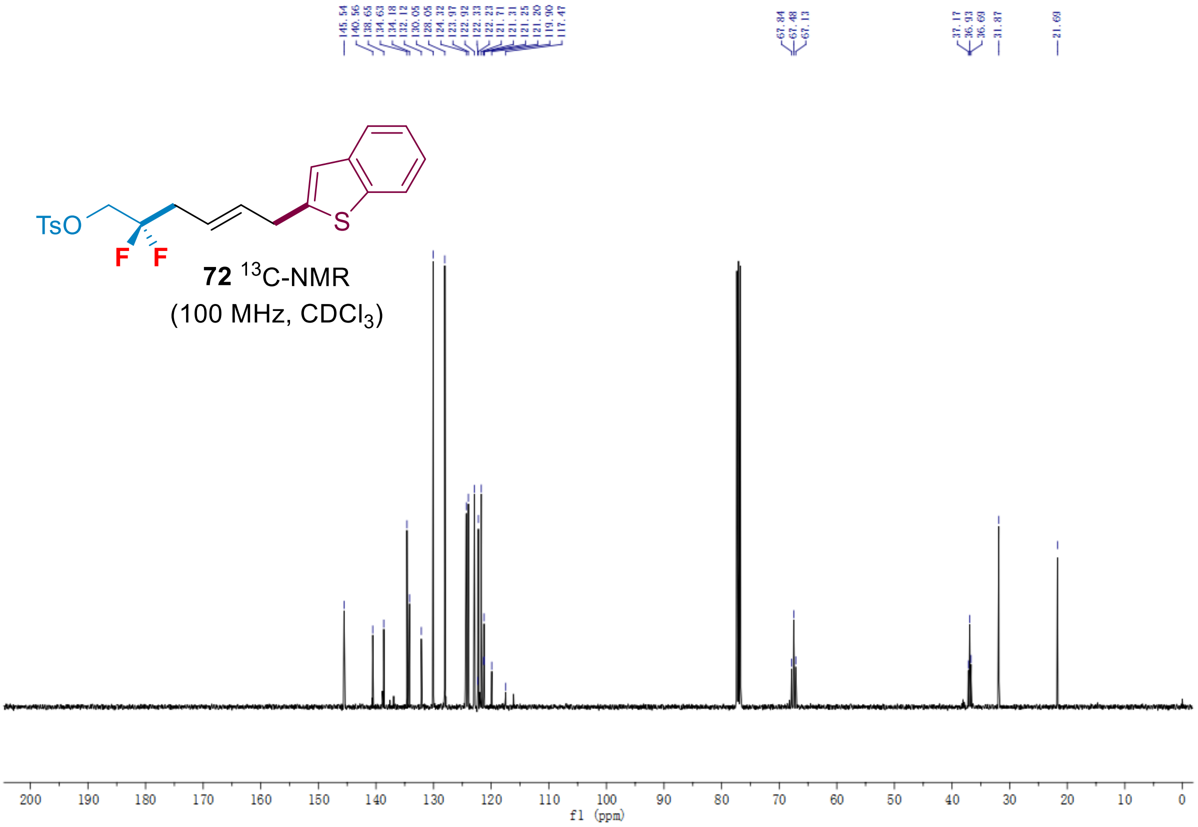
145.54
140.56
138.65
134.63
134.18
132.12
130.05
128.05
124.32
123.97
122.92
122.33
122.23
121.71
121.31
121.25
121.20
119.90
117.47
67.84
67.48
67.13
37.17
36.93
36.69
31.87
21.69
TsO
F
F
S
72 ^{13}C-NMR
(100 MHz, $CDCl_3$)
200 190 180 170 160 150 140 130 120 110 100 90 80 70 60 50 40 30 20 10 0
f1 (ppm)

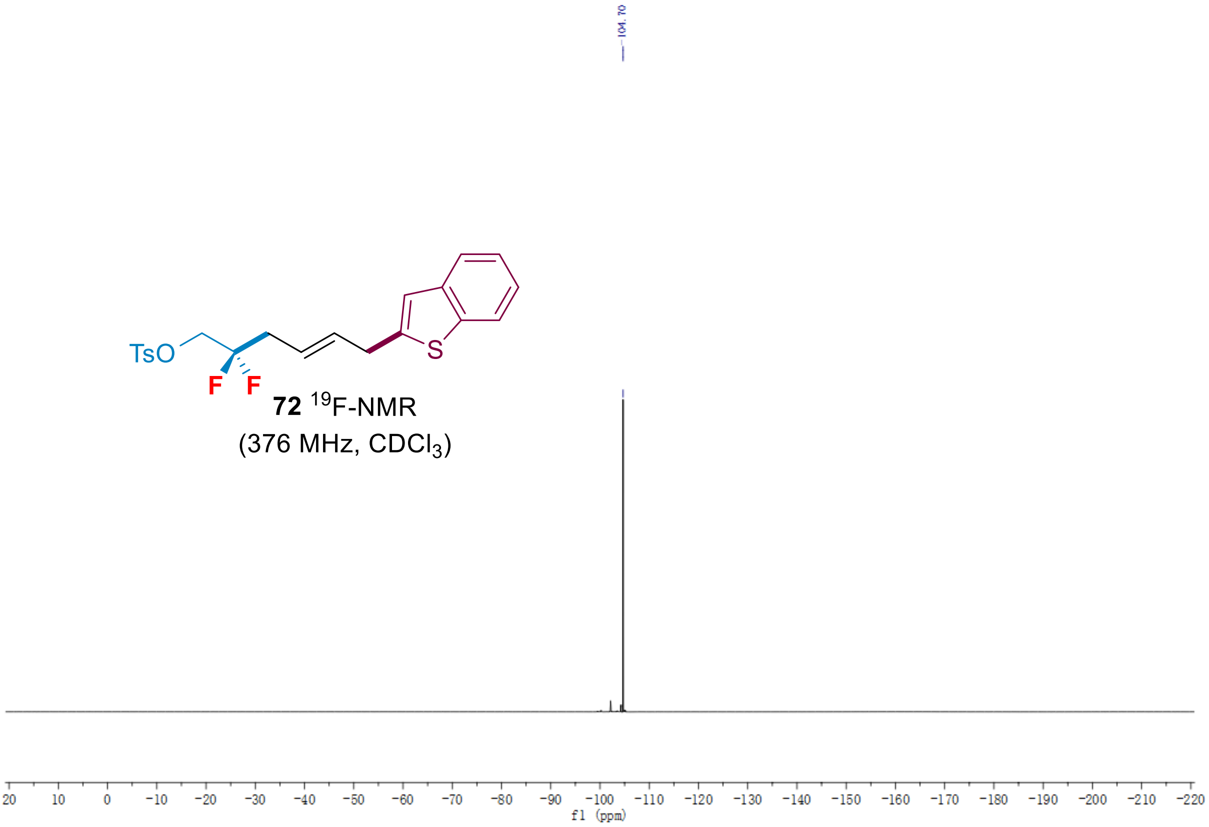
-104.70
TsO
F
F
S
72 ^{19}F-NMR
(376 MHz, $CDCl_3$)
20 10 0 -10 -20 -30 -40 -50 -60 -70 -80 -90 -100 -110 -120 -130 -140 -150 -160 -170 -180 -190 -200 -210 -220
f1 (ppm)

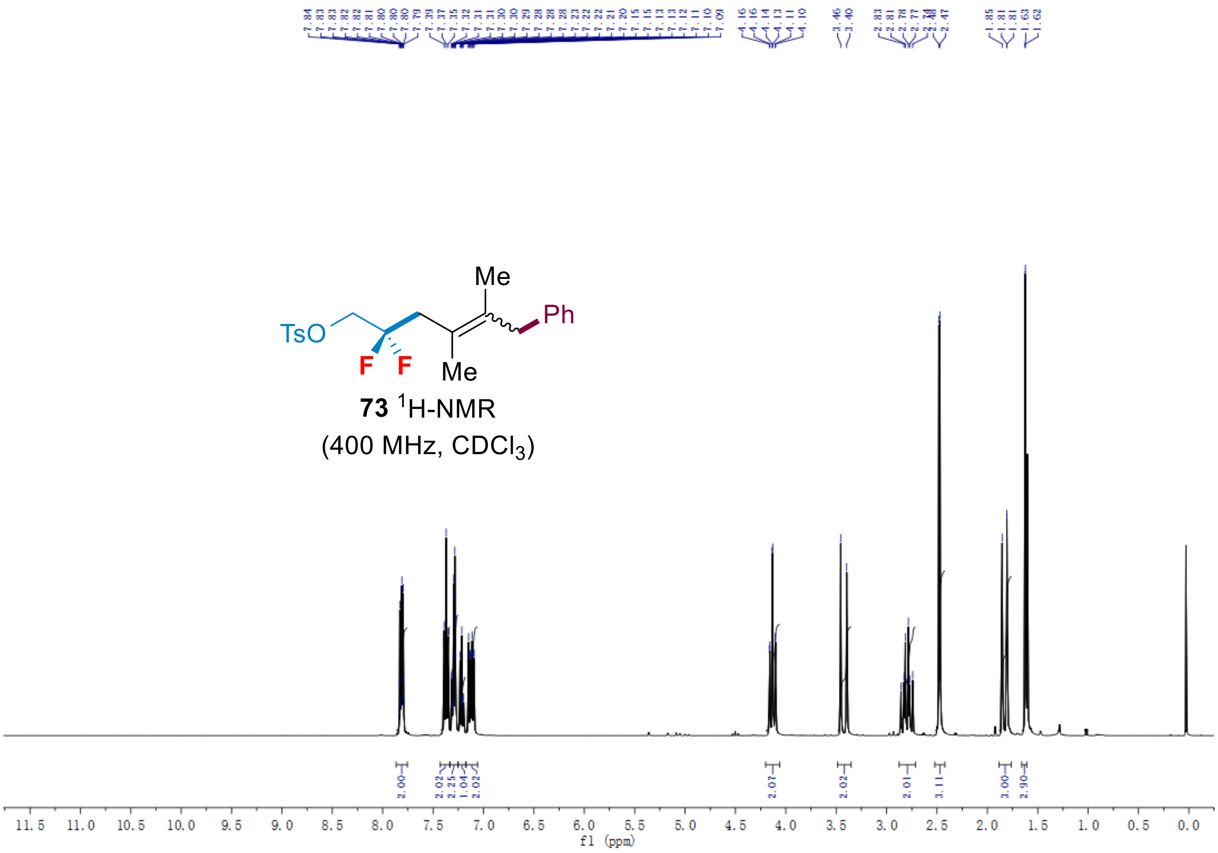
TsO
F F
Me
Me
Ph
73 ^{1}H-NMR
(400 MHz, $CDCl_3$)
f1 (ppm)

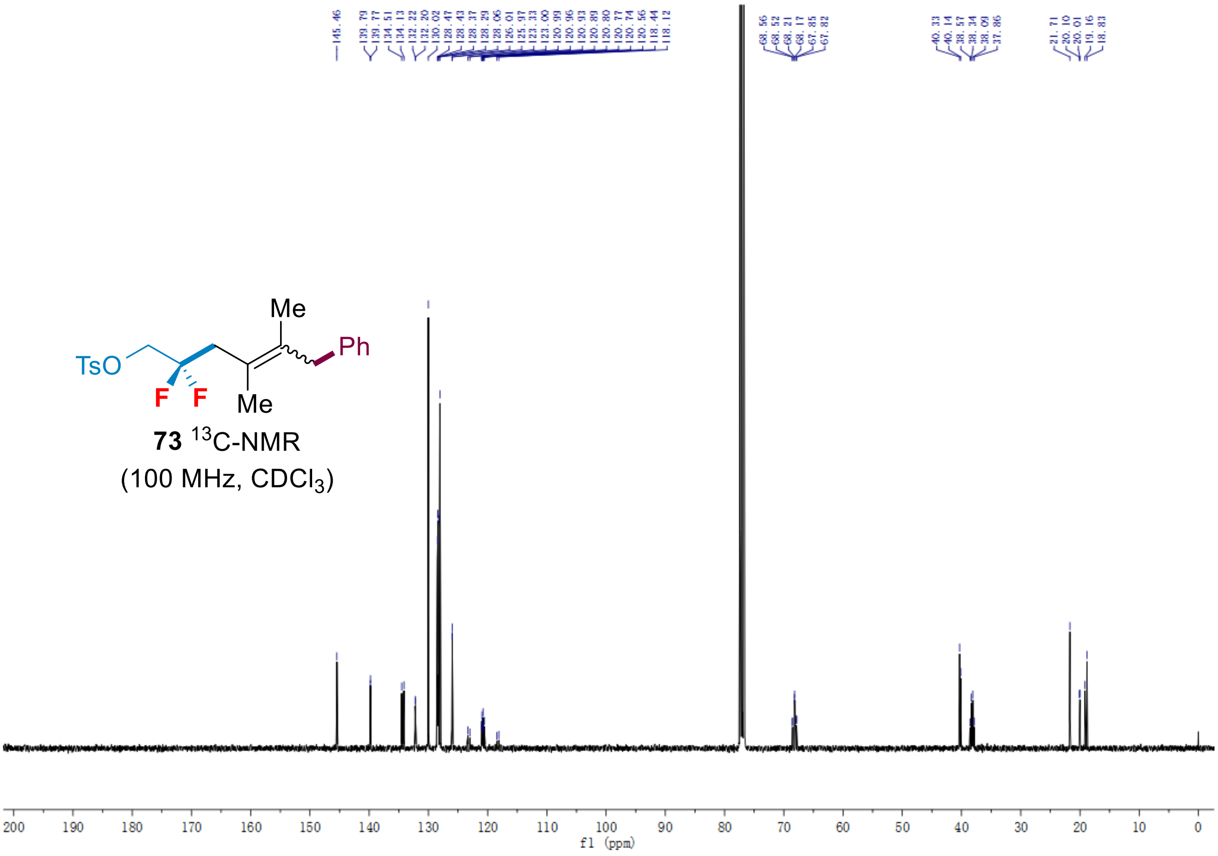
TsO
F F
Me
Me
Ph
73 ^{13}C-NMR
(100 MHz, $CDCl_3$)
f1 (ppm)

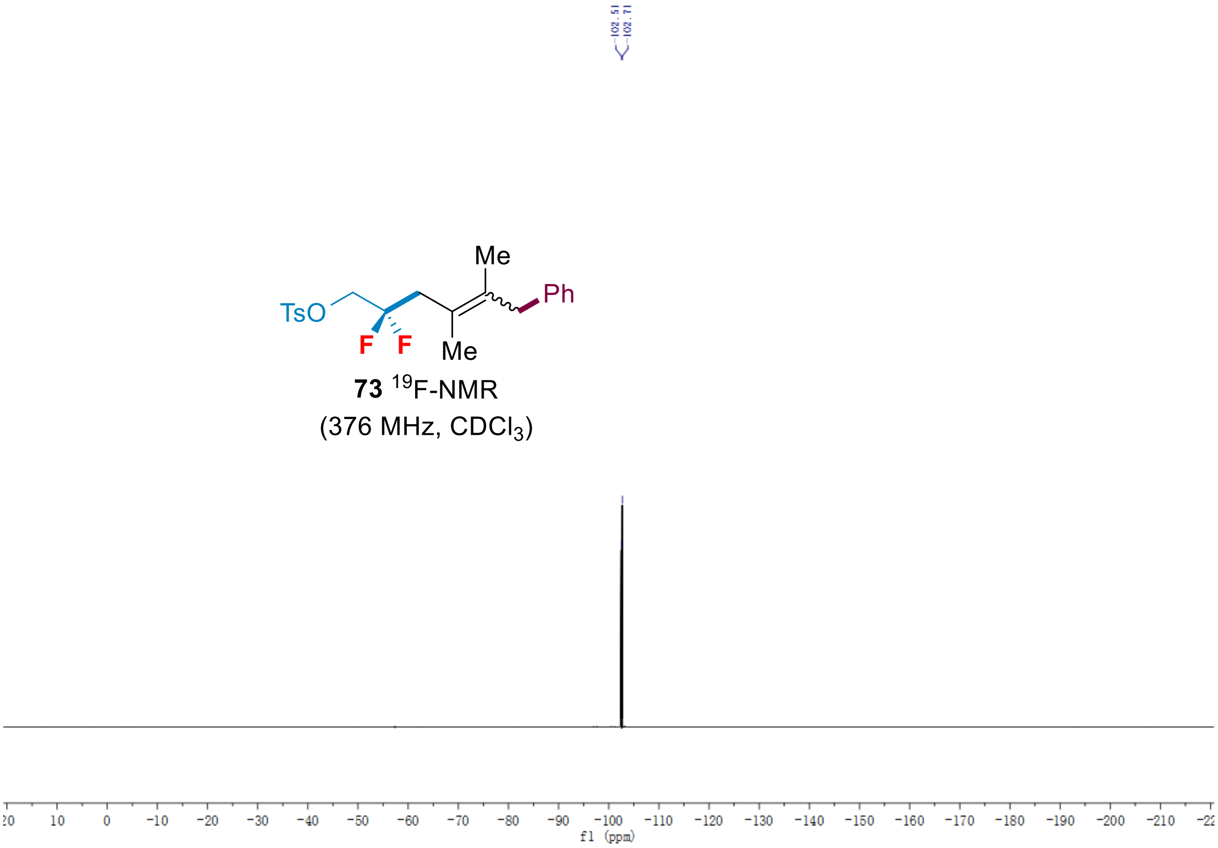
-102.51
-102.71
TsO
F F
Me
Me
Ph
73 ^{19}F-NMR
(376 MHz, CDCl$_3$)
f1 (ppm)

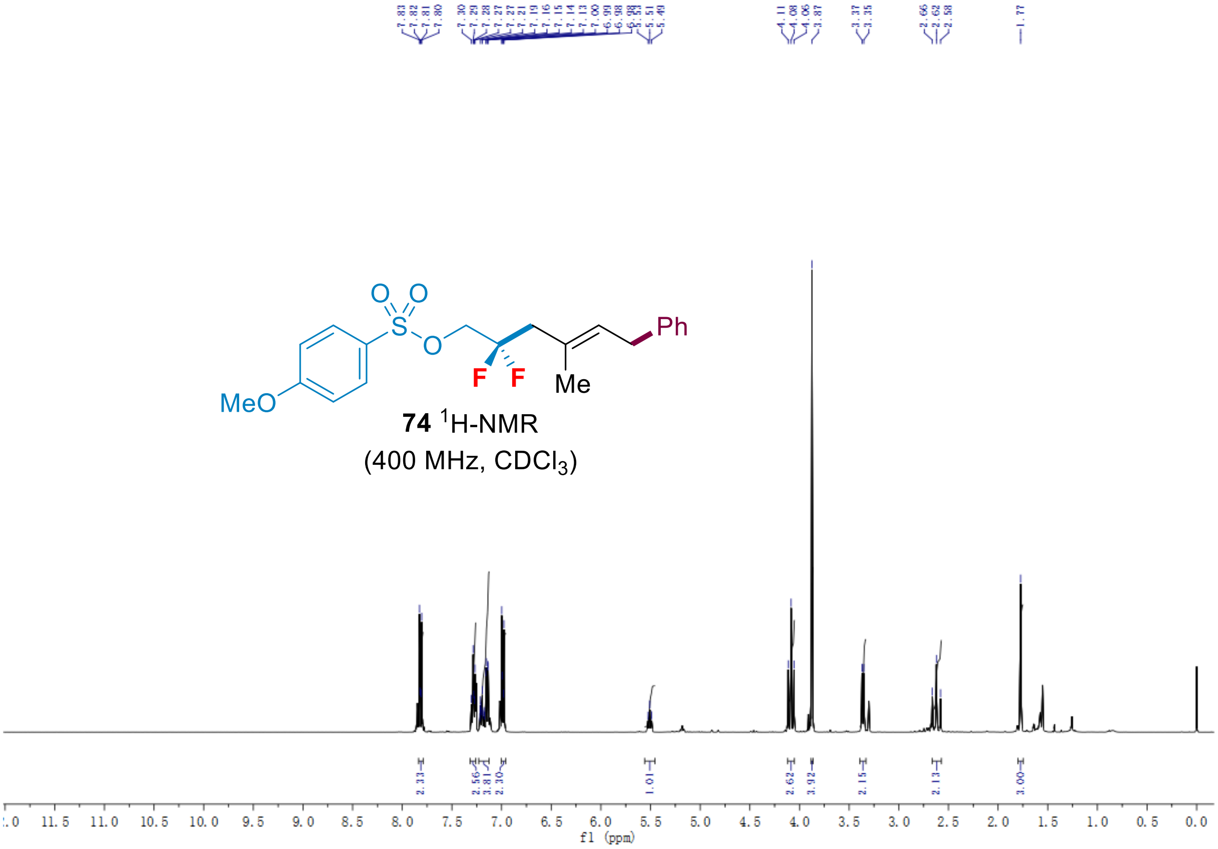
7.83
7.82
7.81
7.80
5.51
5.49
4.11
4.08
4.06
3.87
3.37
3.35
2.66
2.62
2.58
1.77
MeO
O O
S
O
F F
Me
Ph
74 ^{1}H-NMR
(400 MHz, CDCl$_3$)
2.33
2.56
3.81
2.30
1.01
2.62
3.92
2.15
2.13
3.00
f1 (ppm)

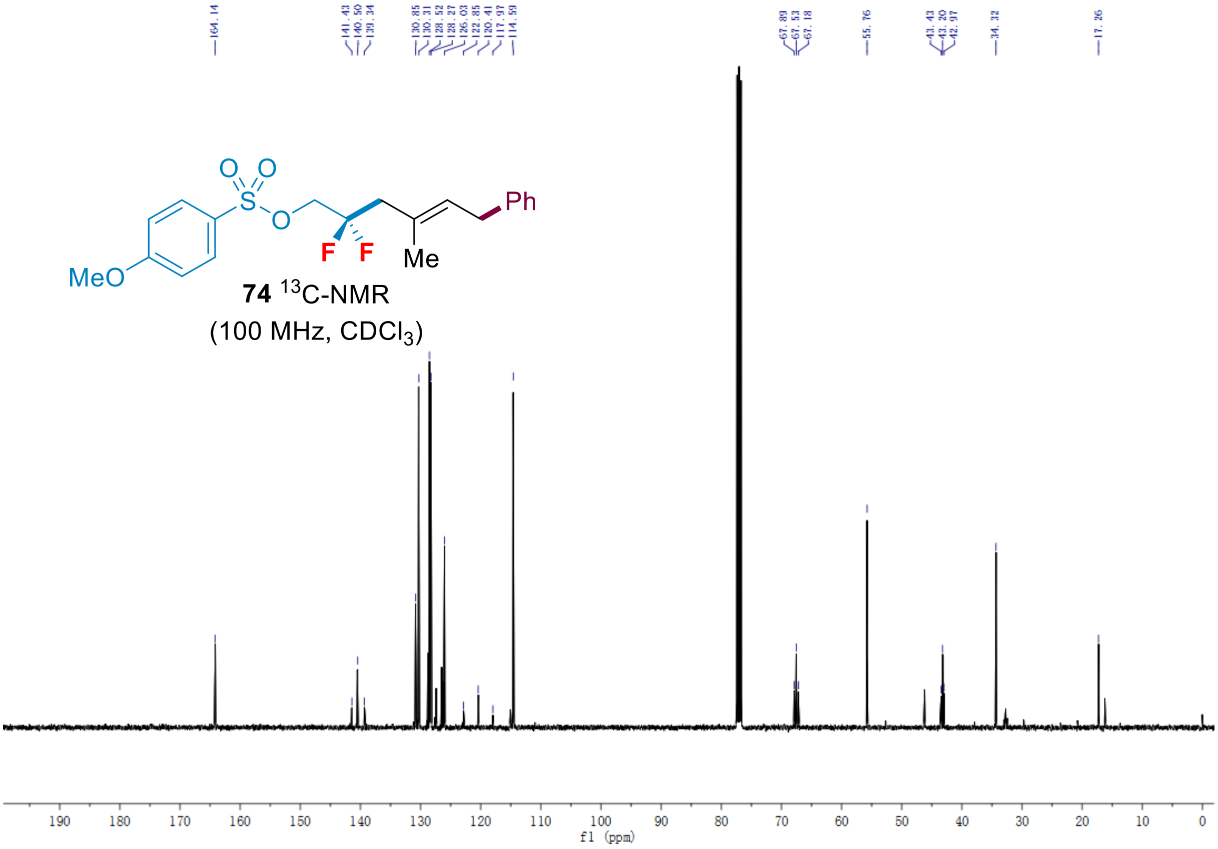
MeO
F F
Me
Ph
74 13C-NMR
(100 MHz, CDCl3)
164.14
141.43
140.50
139.34
130.85
130.31
128.52
128.27
126.03
122.85
120.41
117.97
114.59
67.89
67.53
67.18
55.76
43.43
43.20
42.97
34.32
17.26
f1 (ppm)

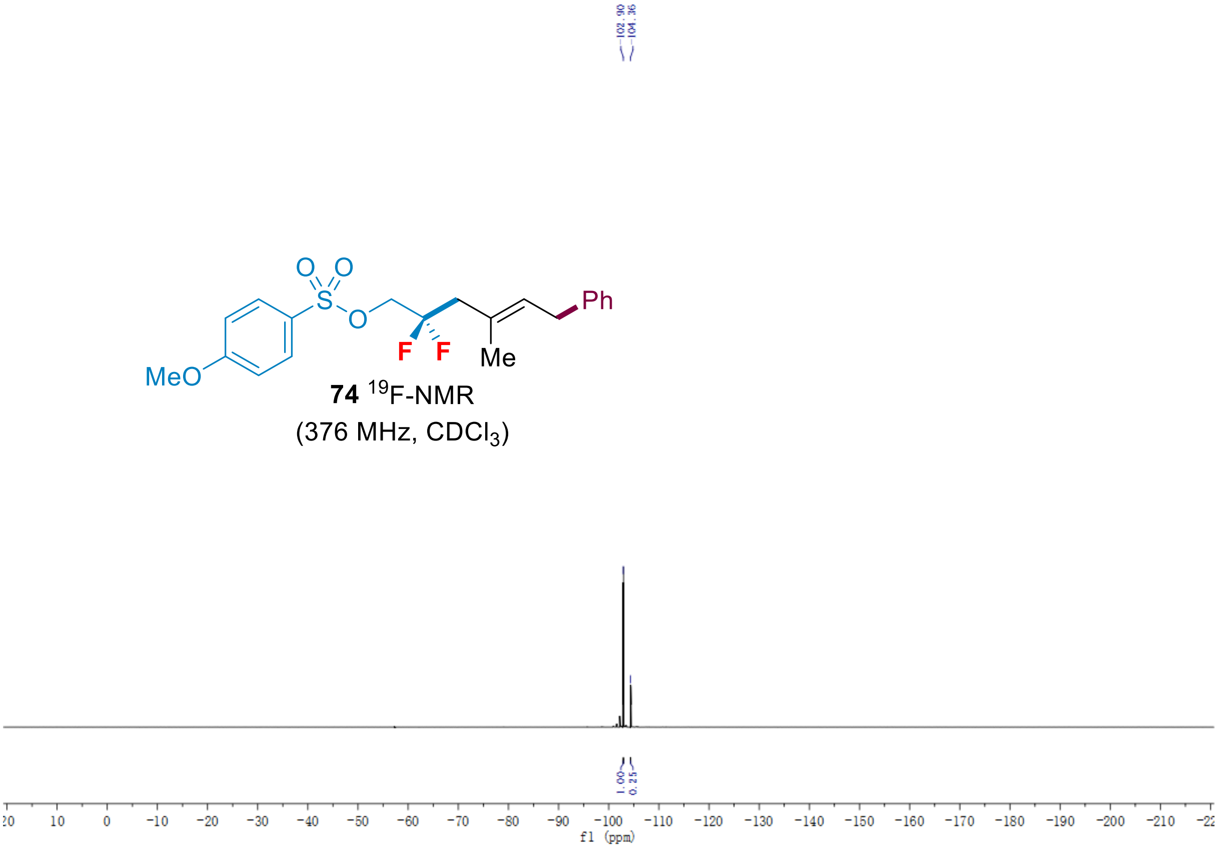
MeO
F F
Me
Ph
74 19F-NMR
(376 MHz, CDCl3)
-102.99
-104.36
1.00
0.25
f1 (ppm)

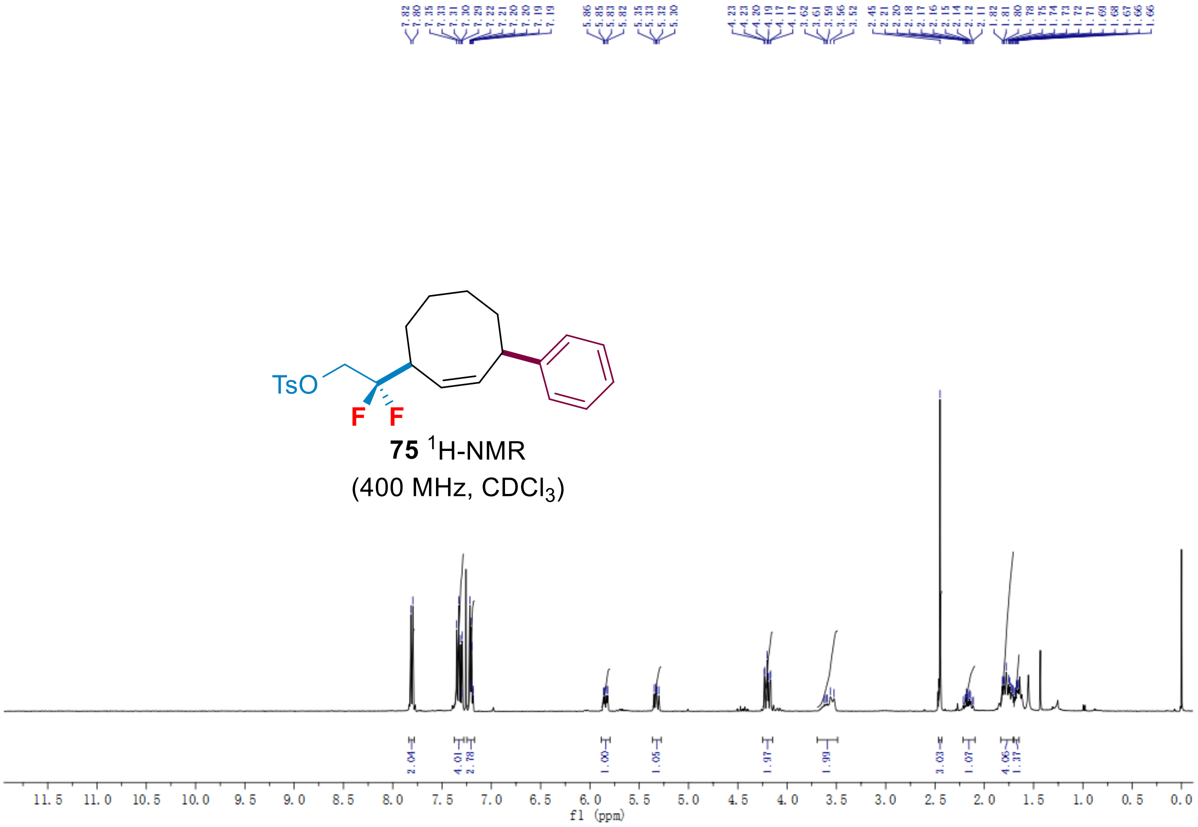

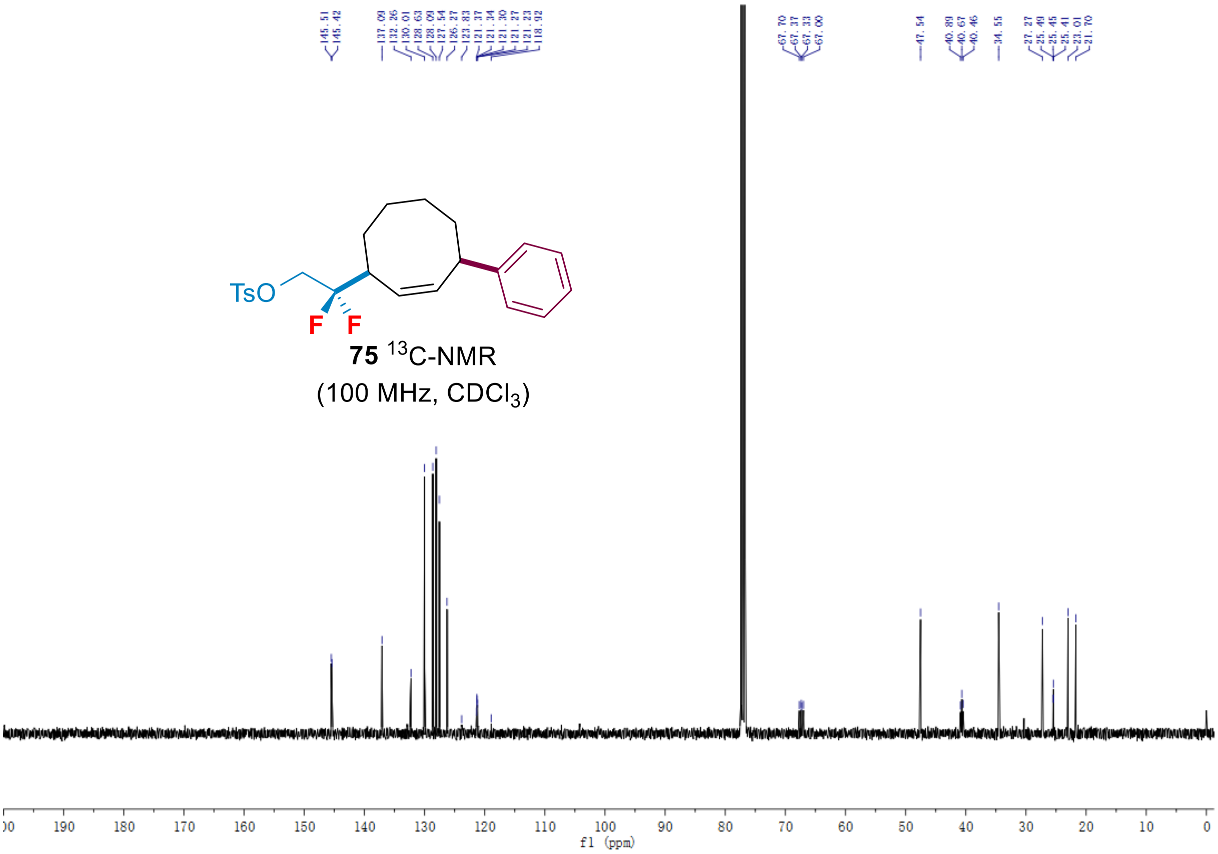

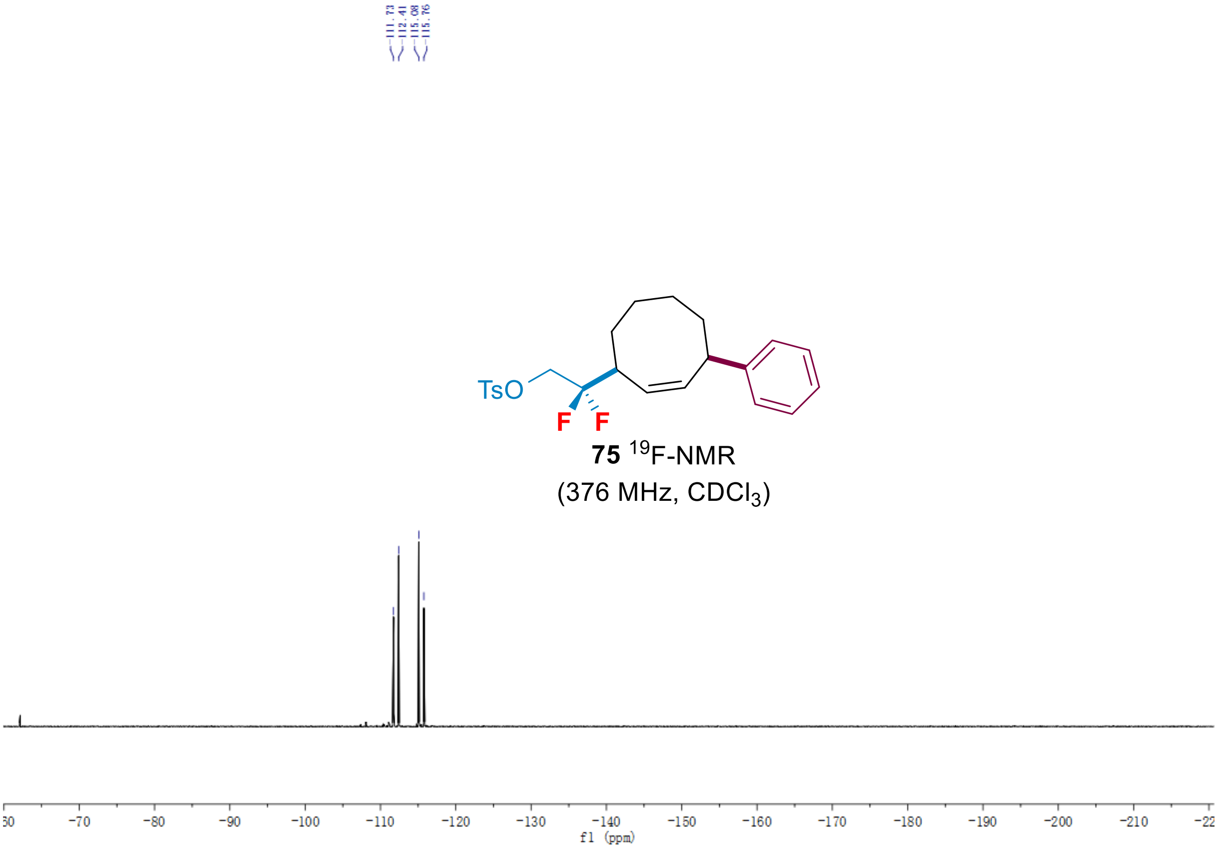

75 ^{19}F-NMR
(376 MHz, $CDCl_3$)

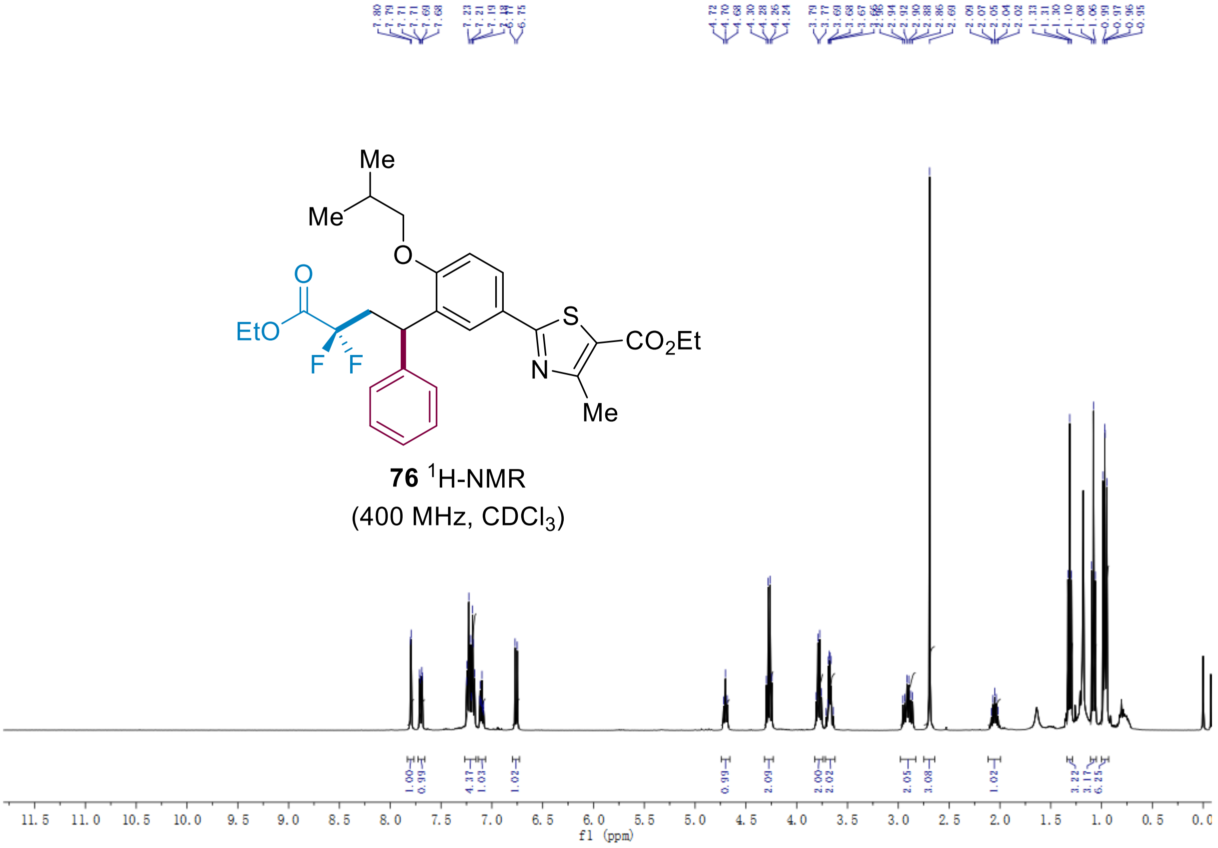

76 ^{1}H-NMR
(400 MHz, $CDCl_3$)

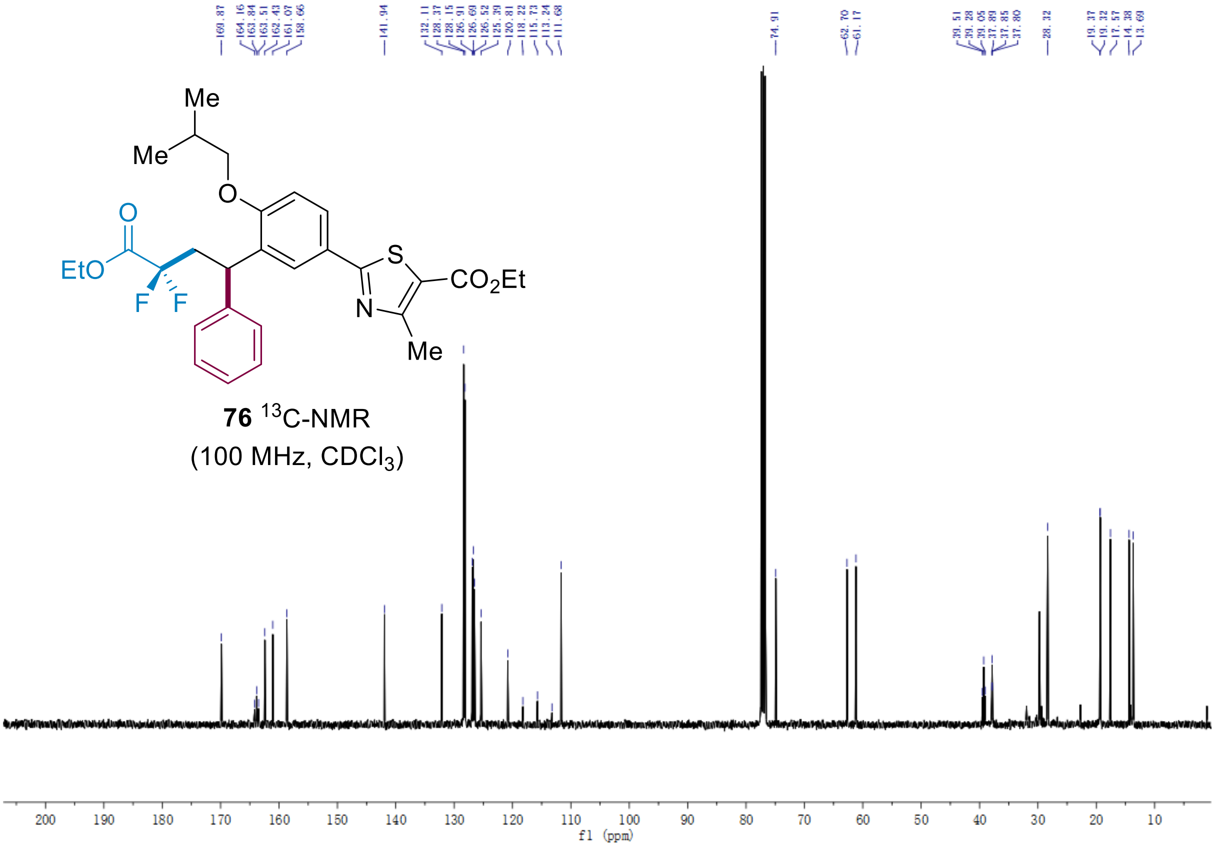
Me
Me
O
O
EtO
F F
S
N
CO2Et
Me
76 13C-NMR
(100 MHz, CDCl3)
f1 (ppm)

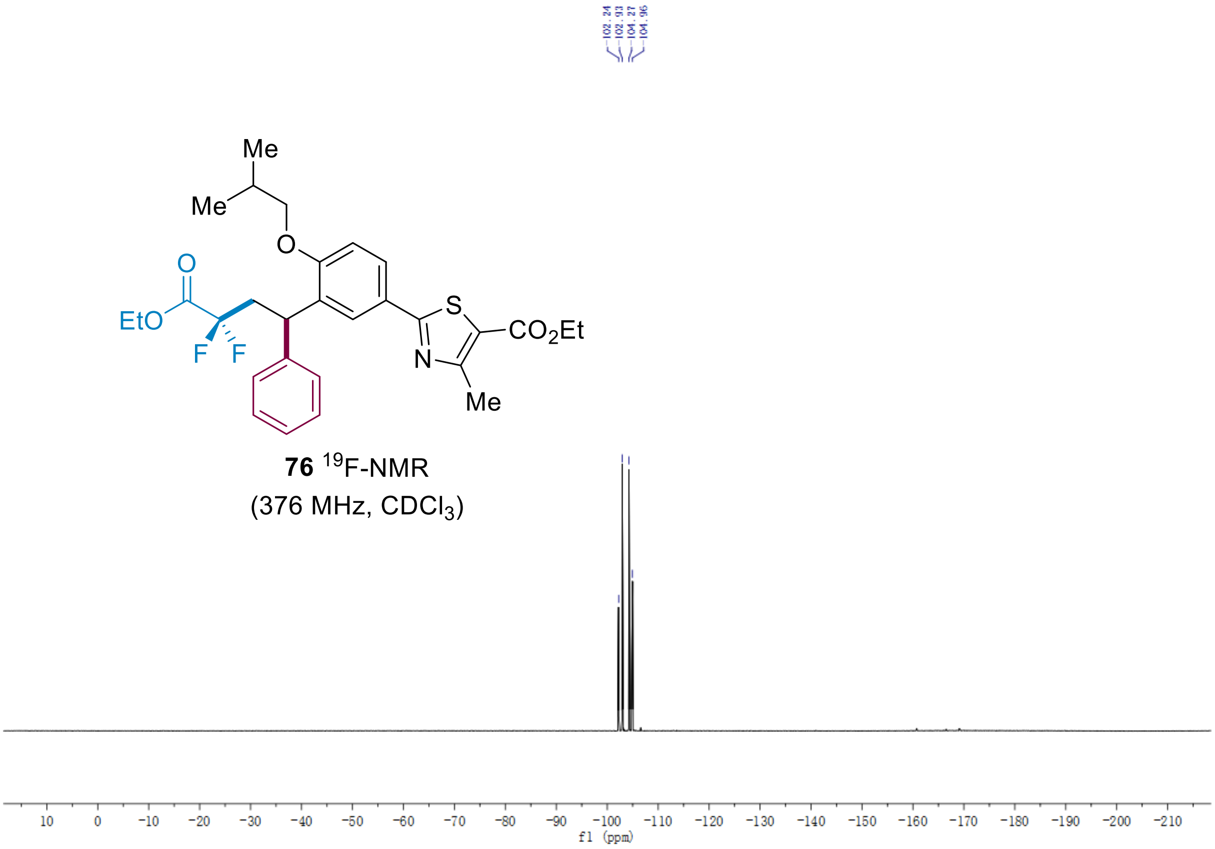
Me
Me
O
O
EtO
F F
S
N
CO2Et
Me
76 19F-NMR
(376 MHz, CDCl3)
f1 (ppm)

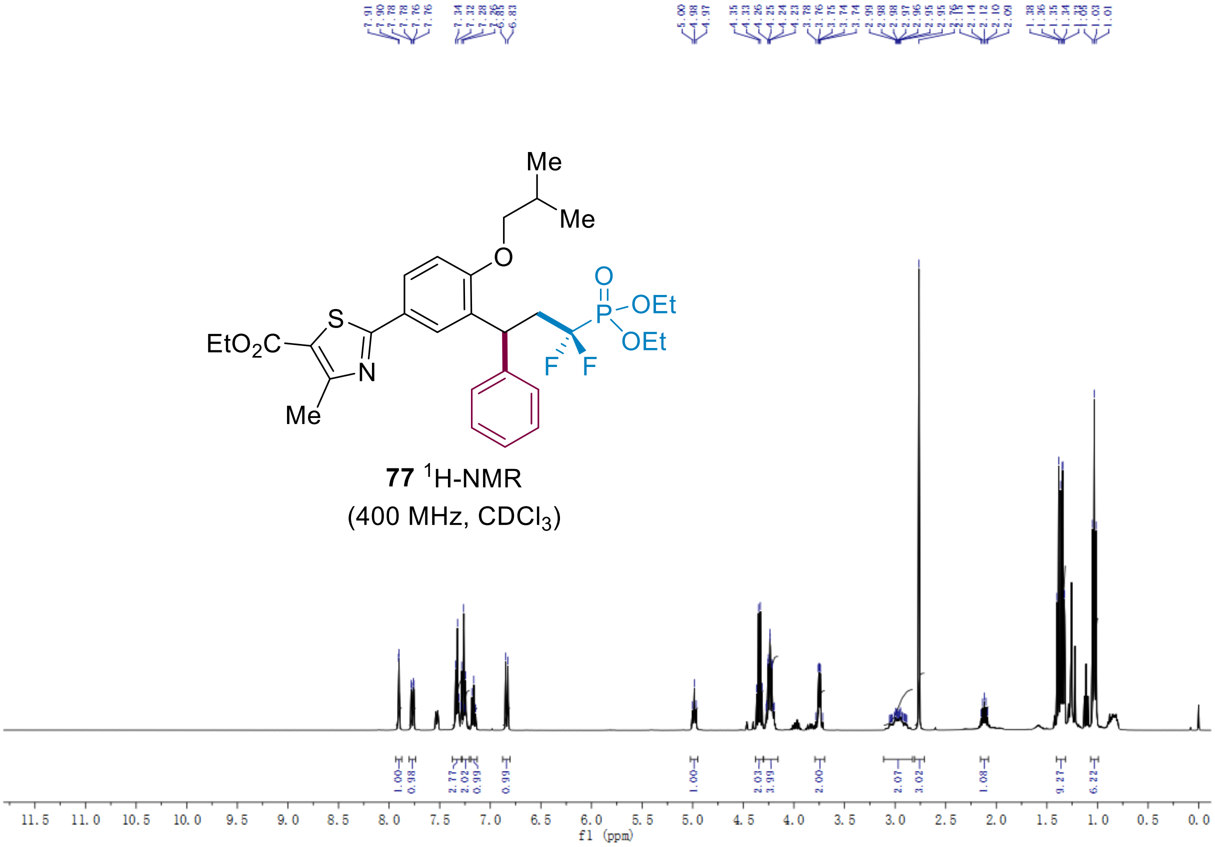

77 ^{1}H-NMR (400 MHz, $CDCl_3$)

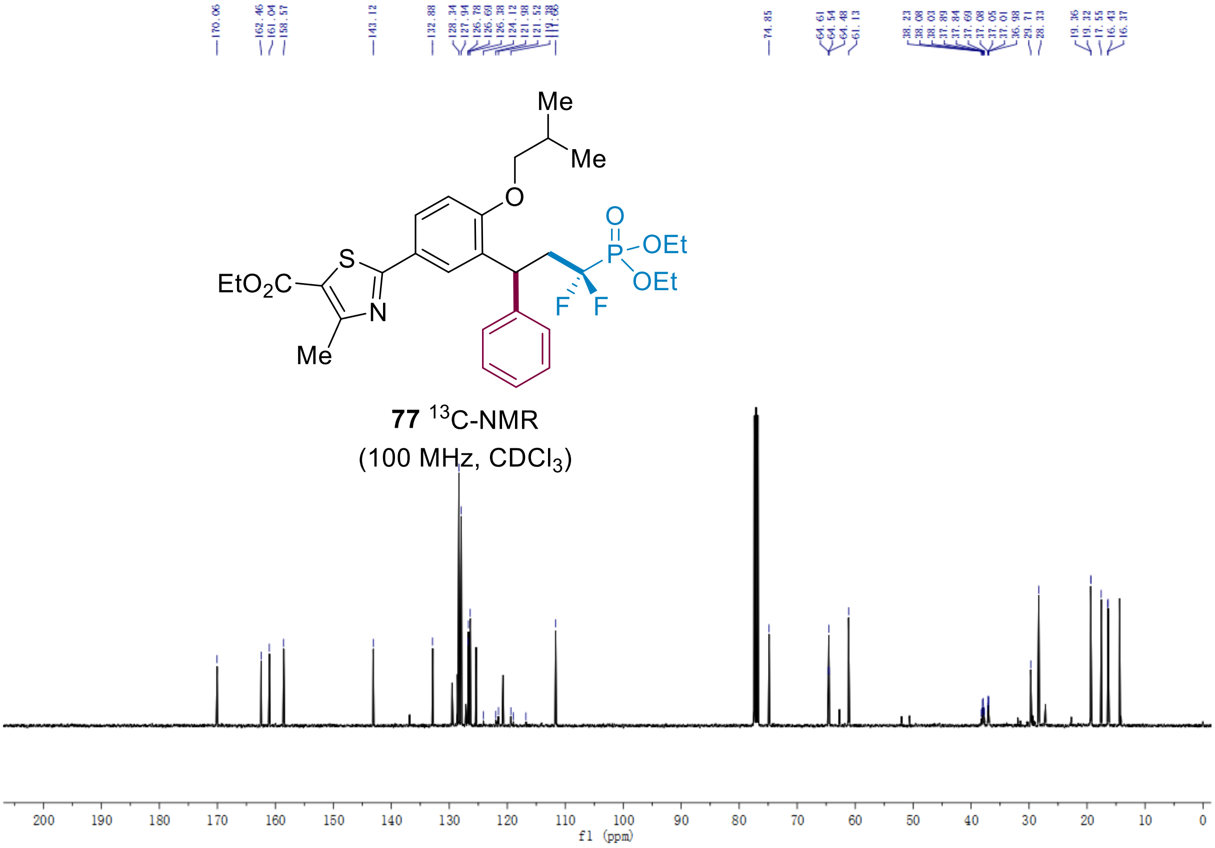

77 ^{13}C-NMR (100 MHz, $CDCl_3$)

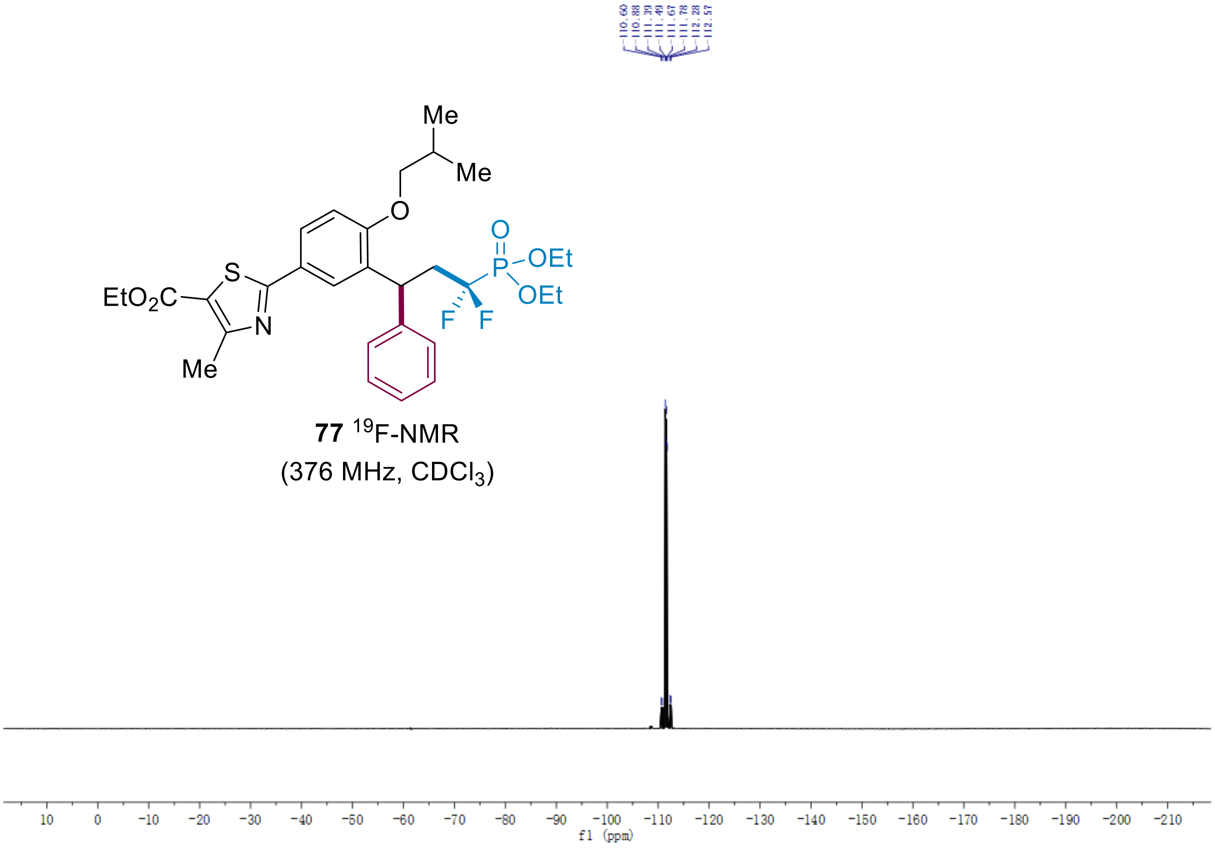

77 ^{19}F-NMR
(376 MHz, $CDCl_3$)

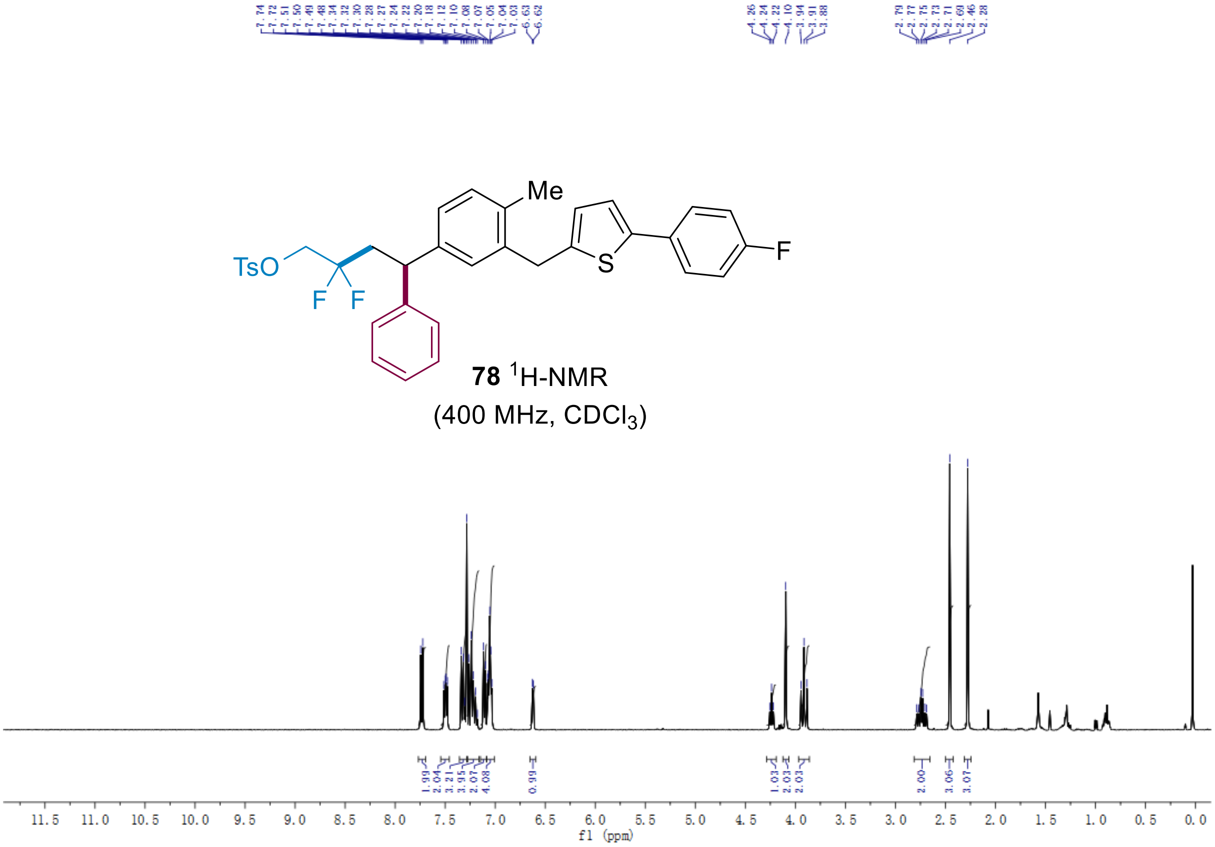

78 ^{1}H-NMR
(400 MHz, $CDCl_3$)

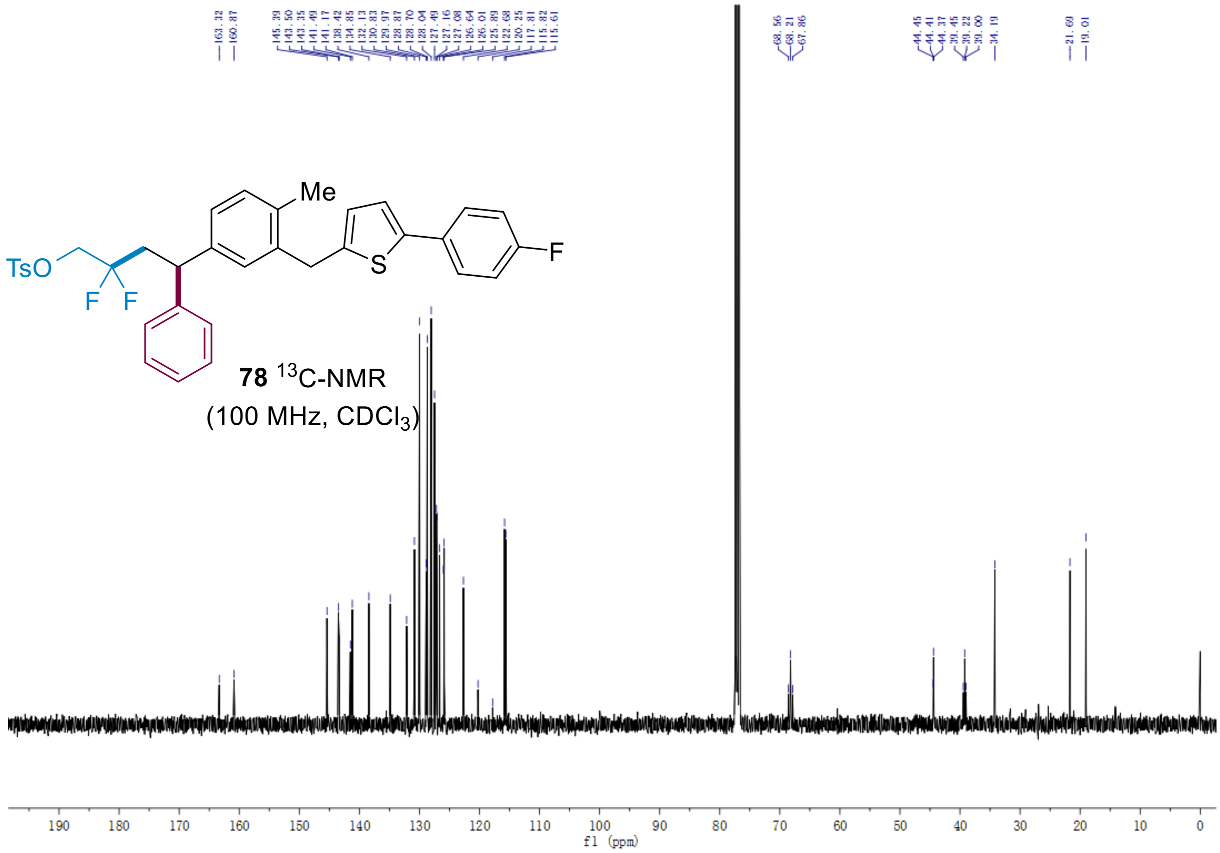

TsO
F F
Me
S
F
78 13C-NMR
(100 MHz, CDCl3)
f1 (ppm)

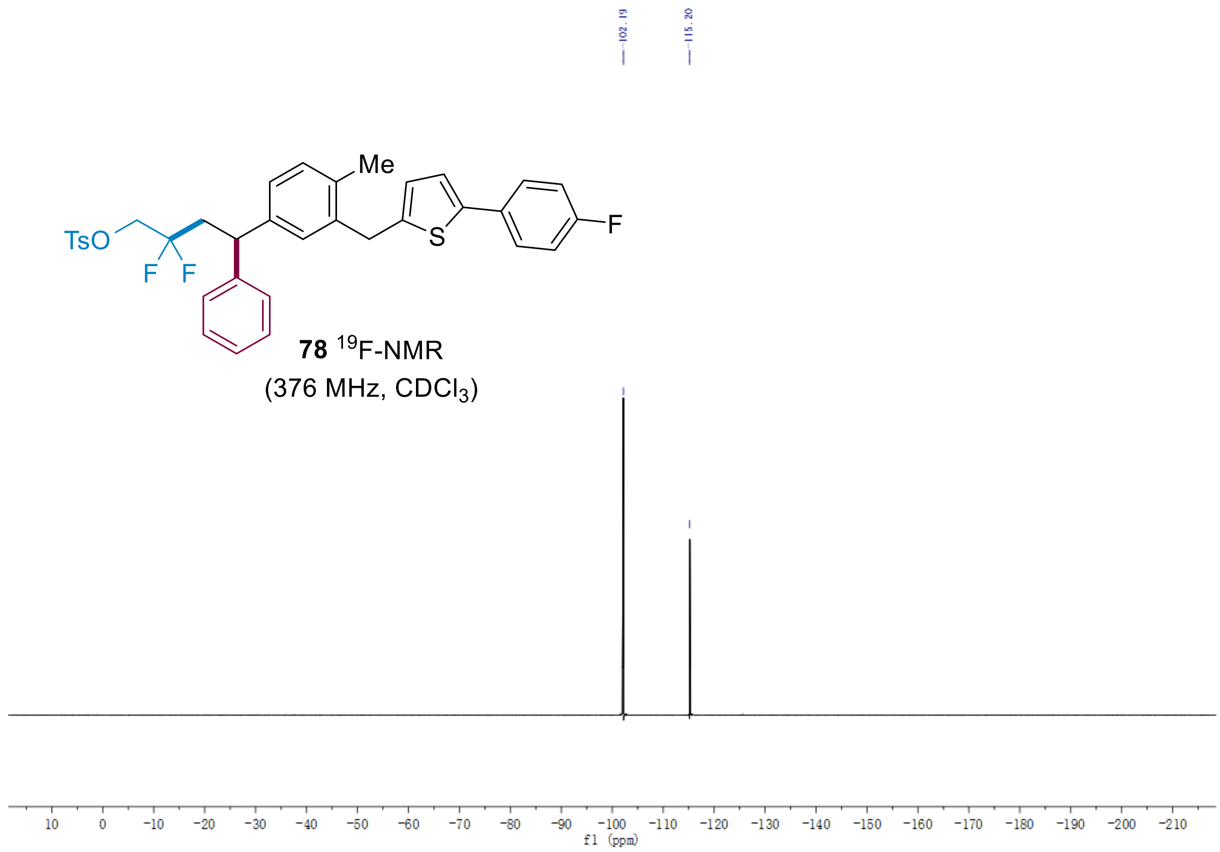

TsO
F F
Me
S
F
78 19F-NMR
(376 MHz, CDCl3)
f1 (ppm)

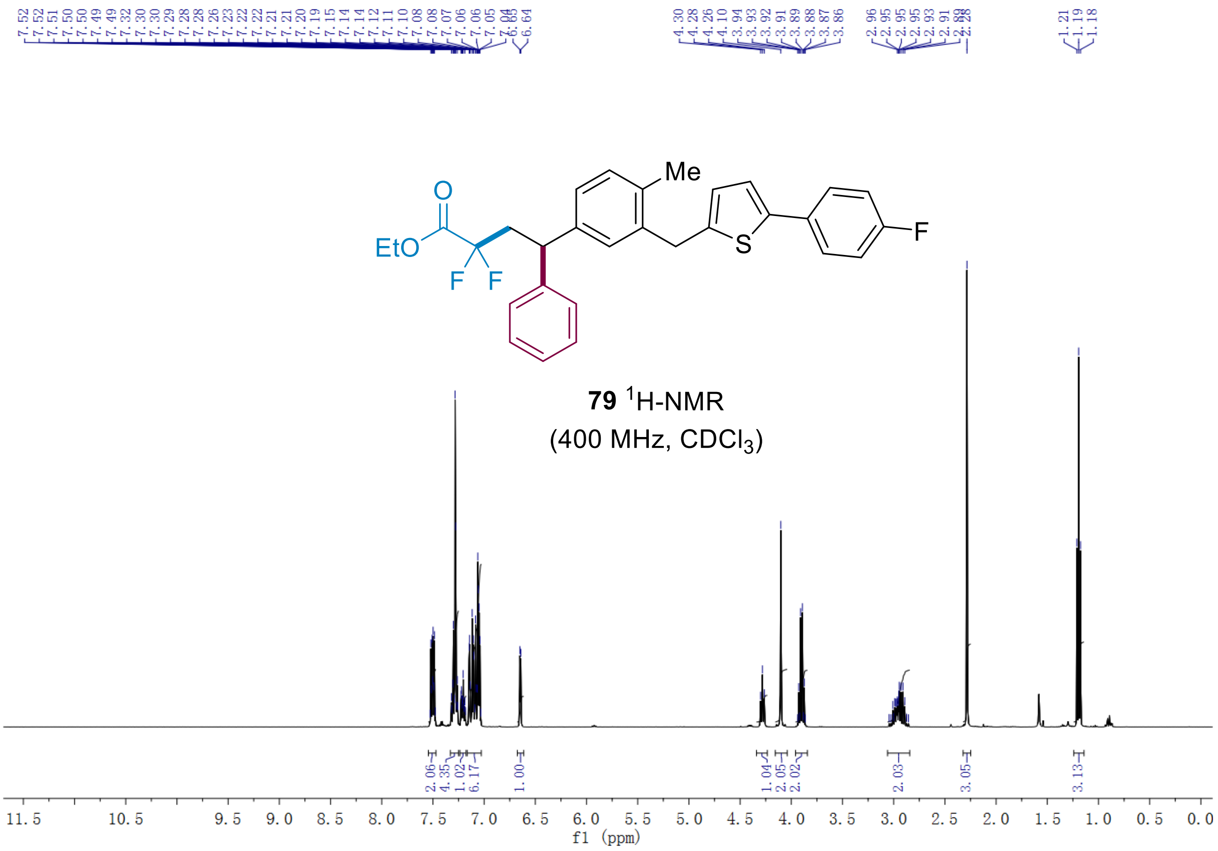
EtO
O
F F
Me
S
F
79 1H-NMR
(400 MHz, CDCl3)
f1 (ppm)

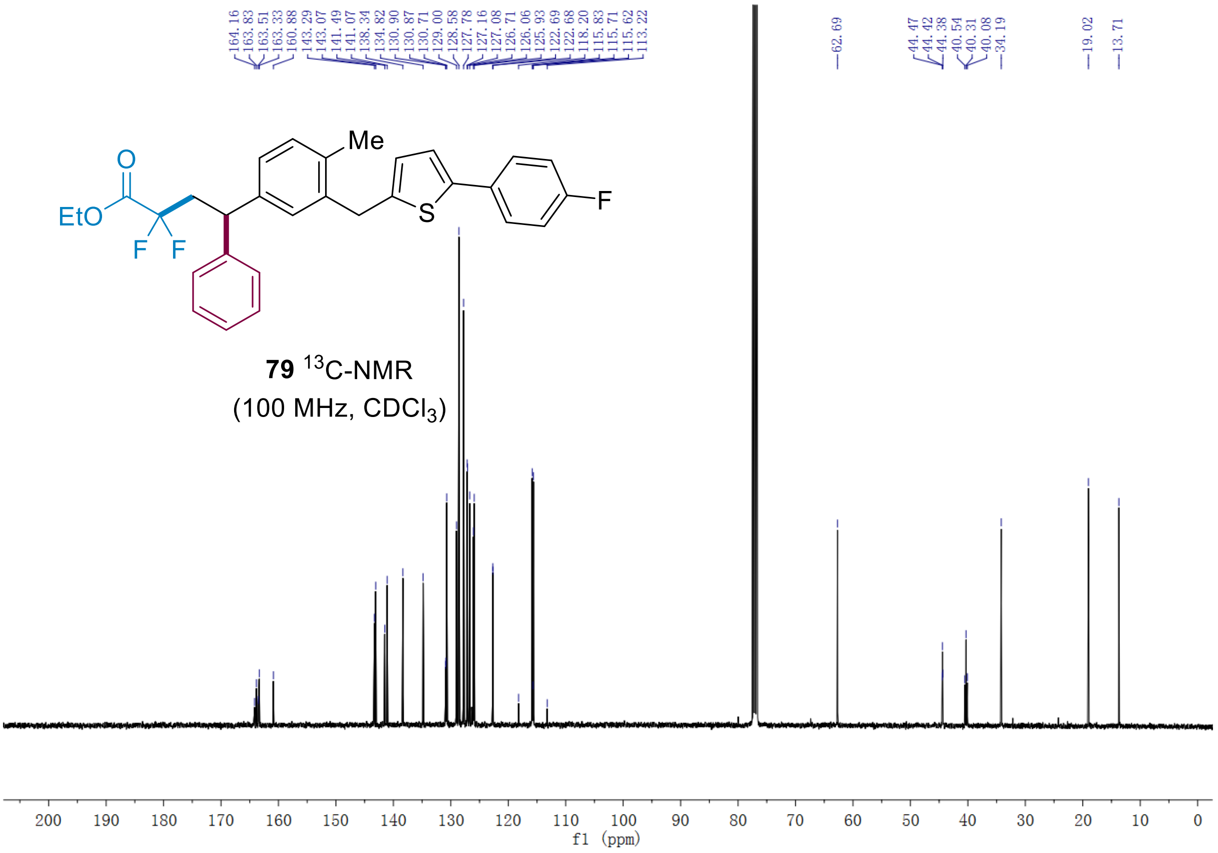
EtO
O
F F
Me
S
F
79 13C-NMR
(100 MHz, CDCl3)
f1 (ppm)

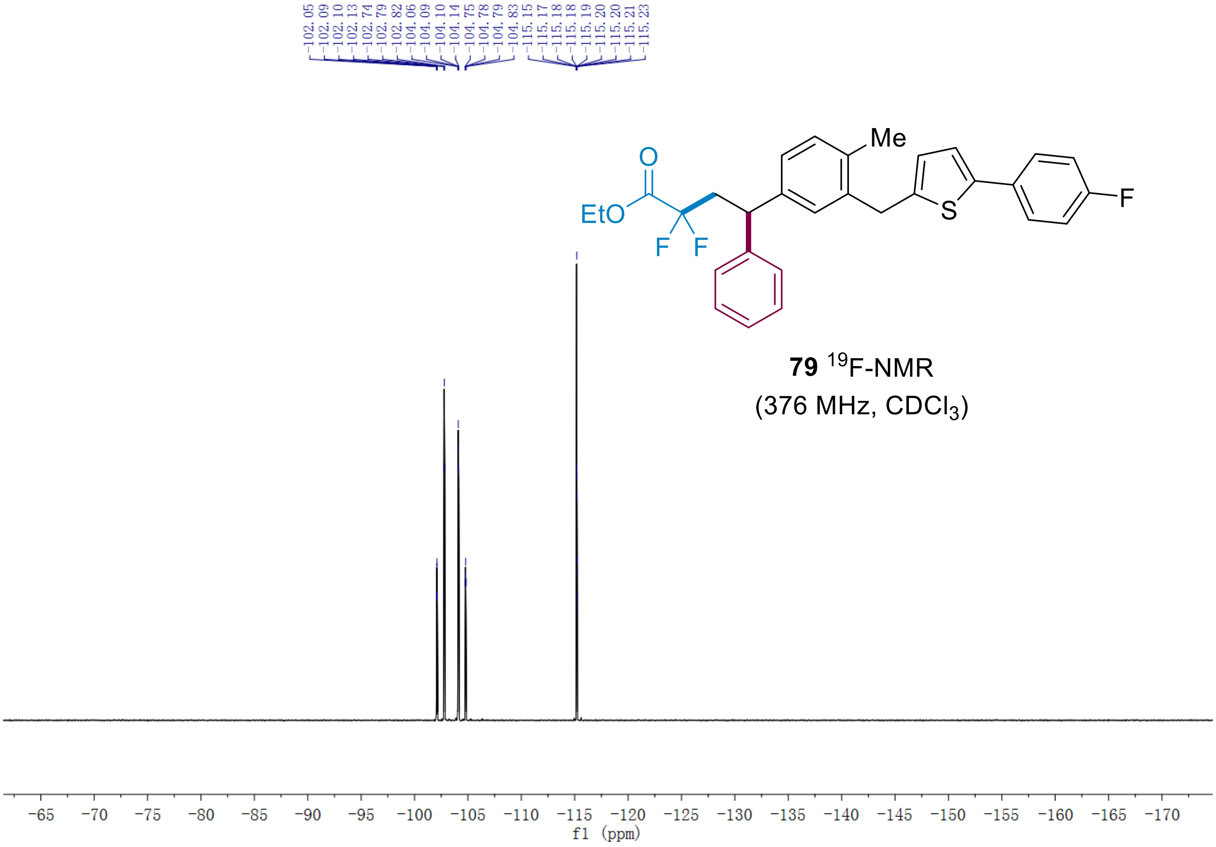

−102.05 −102.09 −102.10 −102.13 −102.74 −102.79 −102.82 −104.06 −104.09 −104.10 −104.14 −104.75 −104.78 −104.79 −104.83 −115.15 −115.17 −115.18 −115.18 −115.19 −115.20 −115.20 −115.21 −115.23
Me
O
EtO
F F
S
F
79 19F-NMR
(376 MHz, CDCl3)
−65 −70 −75 −80 −85 −90 −95 −100 −105 −110 −115 −120 −125 −130 −135 −140 −145 −150 −155 −160 −165 −170
f1 (ppm)

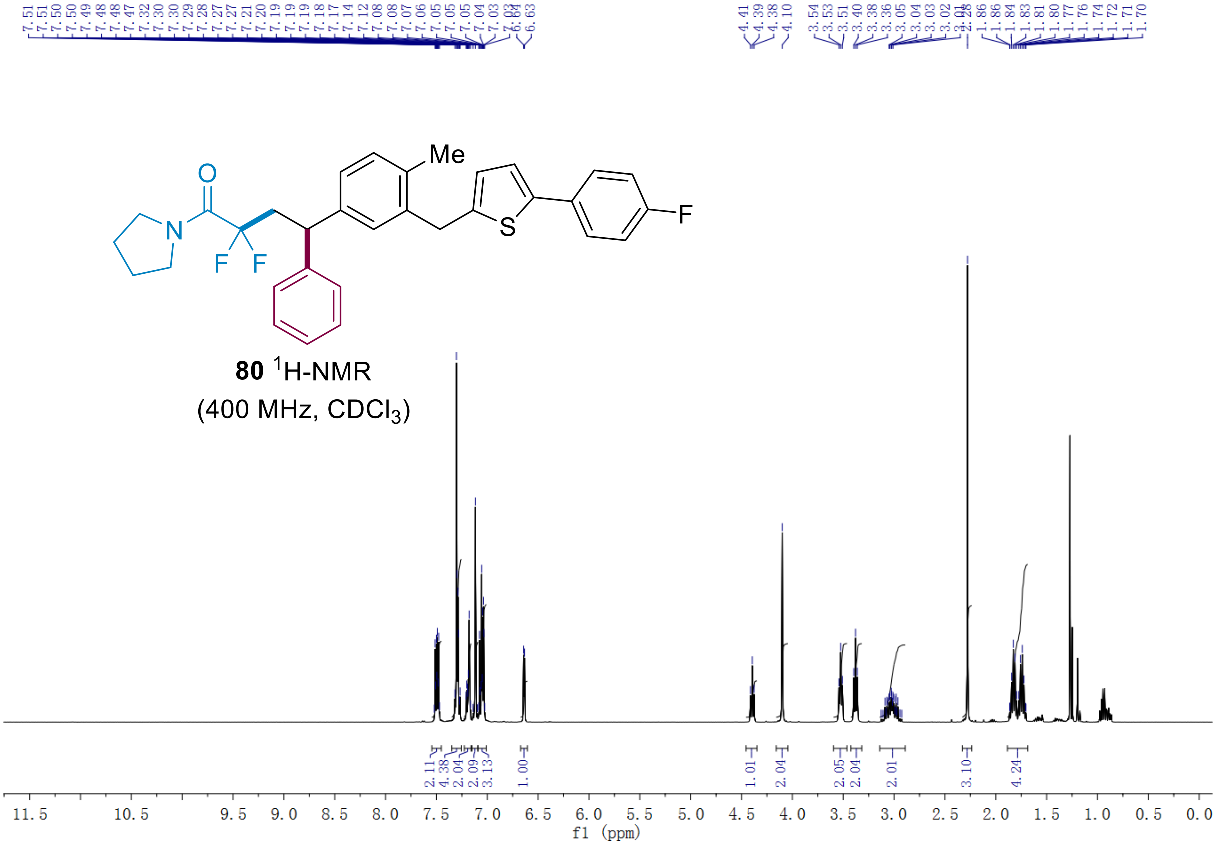

7.51 7.51 7.50 7.49 7.48 7.48 7.47 7.32 7.30 7.30 7.29 7.28 7.27 7.27 7.21 7.20 7.19 7.19 7.19 7.18 7.17 7.14 7.12 7.08 7.08 7.07 7.06 7.05 7.05 7.05 7.04 7.03 6.64 6.63
4.41 4.39 4.38 4.10 3.54 3.53 3.51 3.40 3.38 3.36 3.05 3.04 3.03 3.02 3.01 2.28 1.86 1.86 1.84 1.83 1.81 1.80 1.77 1.76 1.74 1.72 1.71 1.70
Me
O
N
F F
S
F
80 1H-NMR
(400 MHz, CDCl3)
2.11 4.38 2.04 2.09 3.13 1.00 1.01 2.04 2.05 2.04 2.01 3.10 4.24
11.5 10.5 9.5 9.0 8.5 8.0 7.5 7.0 6.5 6.0 5.5 5.0 4.5 4.0 3.5 3.0 2.5 2.0 1.5 1.0 0.5 0.0
f1 (ppm)

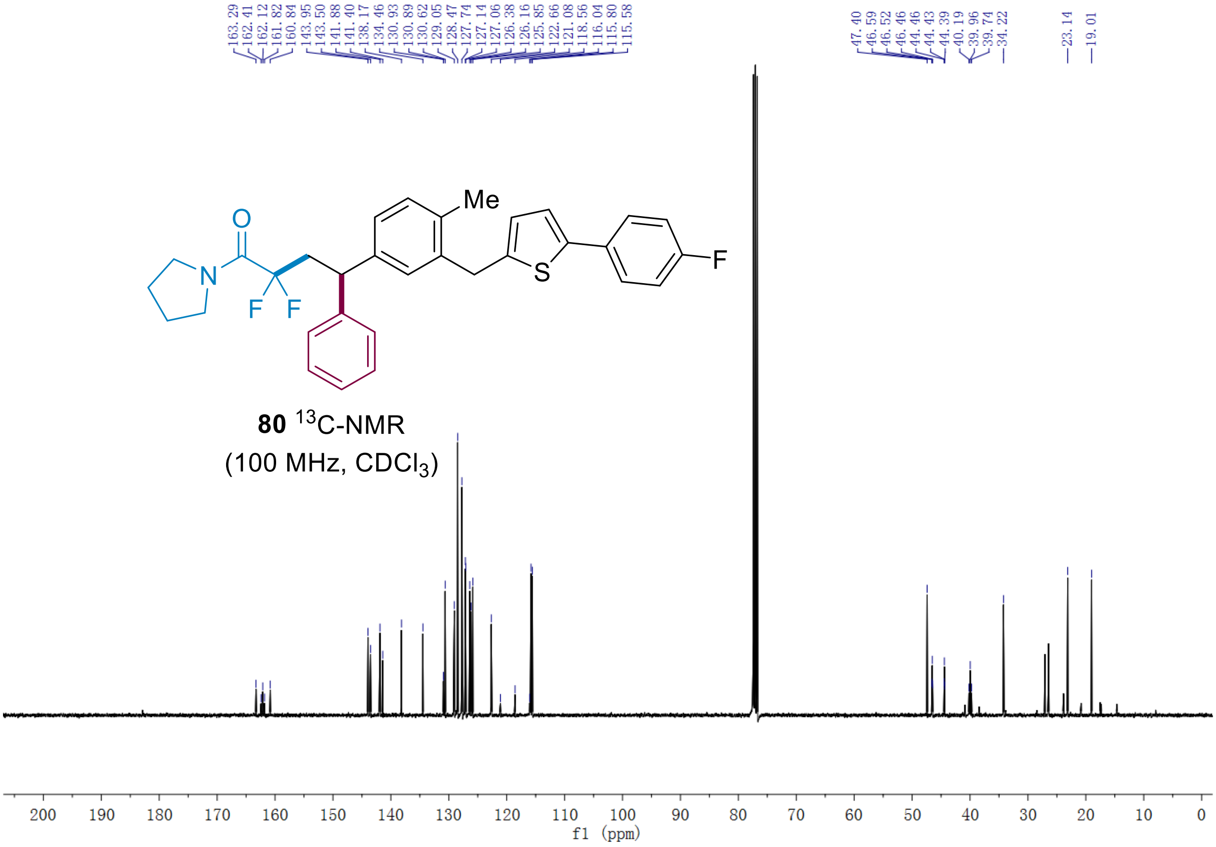

80 ^{13}C-NMR
(100 MHz, $CDCl_3$)

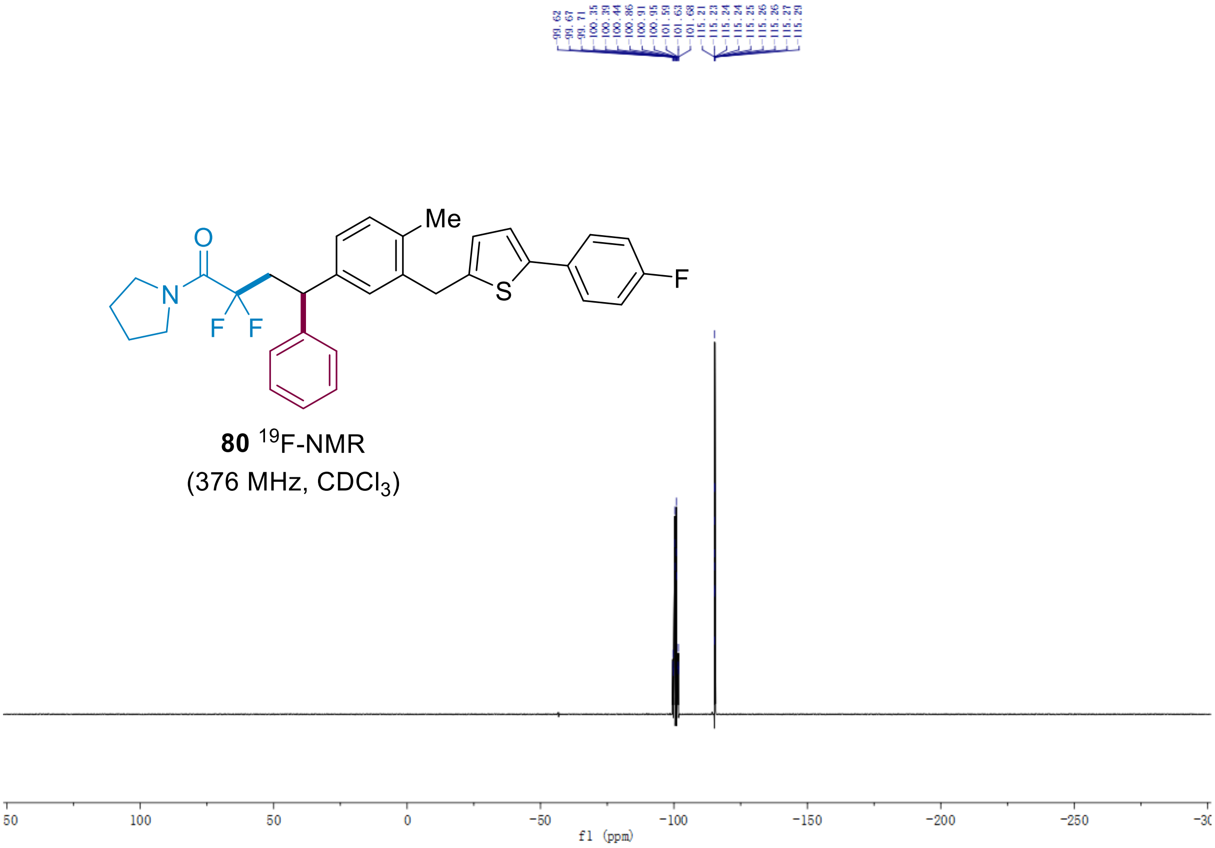

80 ^{19}F-NMR
(376 MHz, $CDCl_3$)

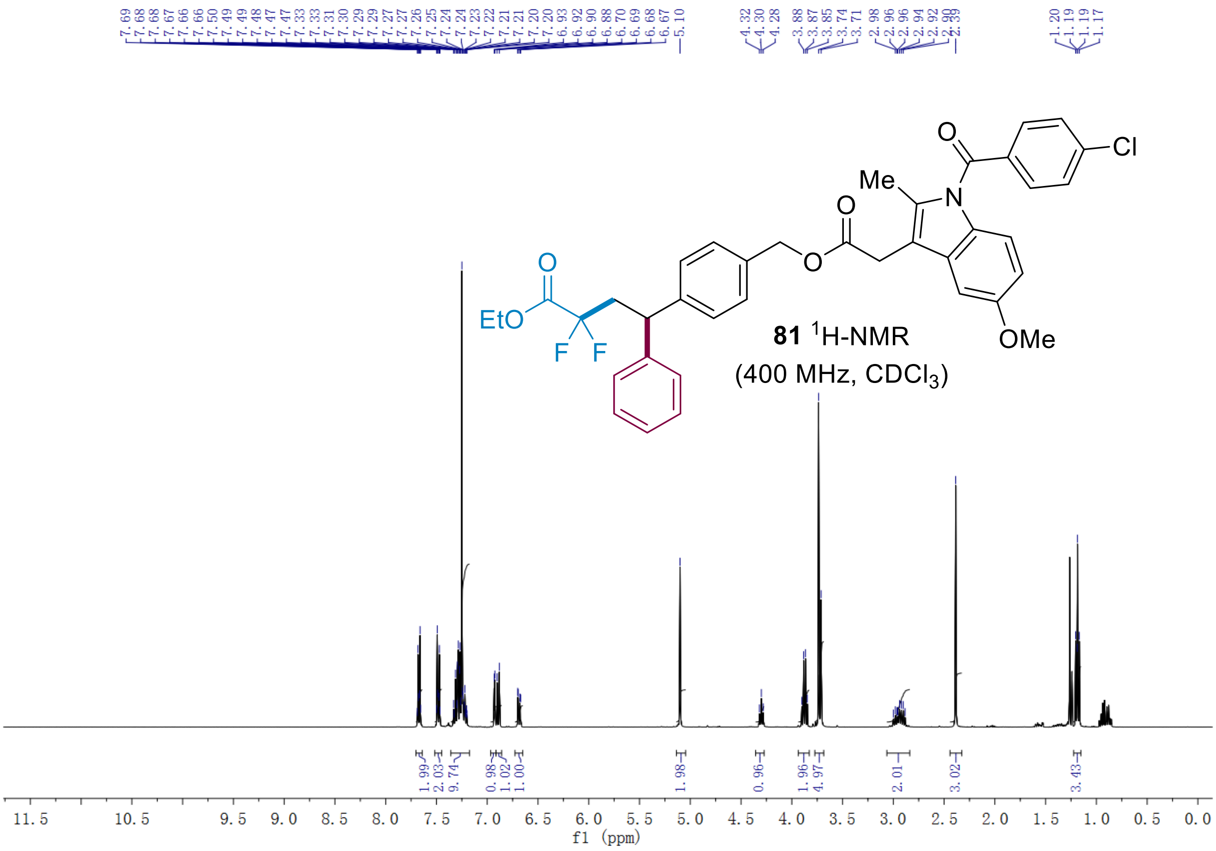

EtO
O
F F
Me
N
O
Cl
OMe
81 1H-NMR
(400 MHz, CDCl3)
f1 (ppm)

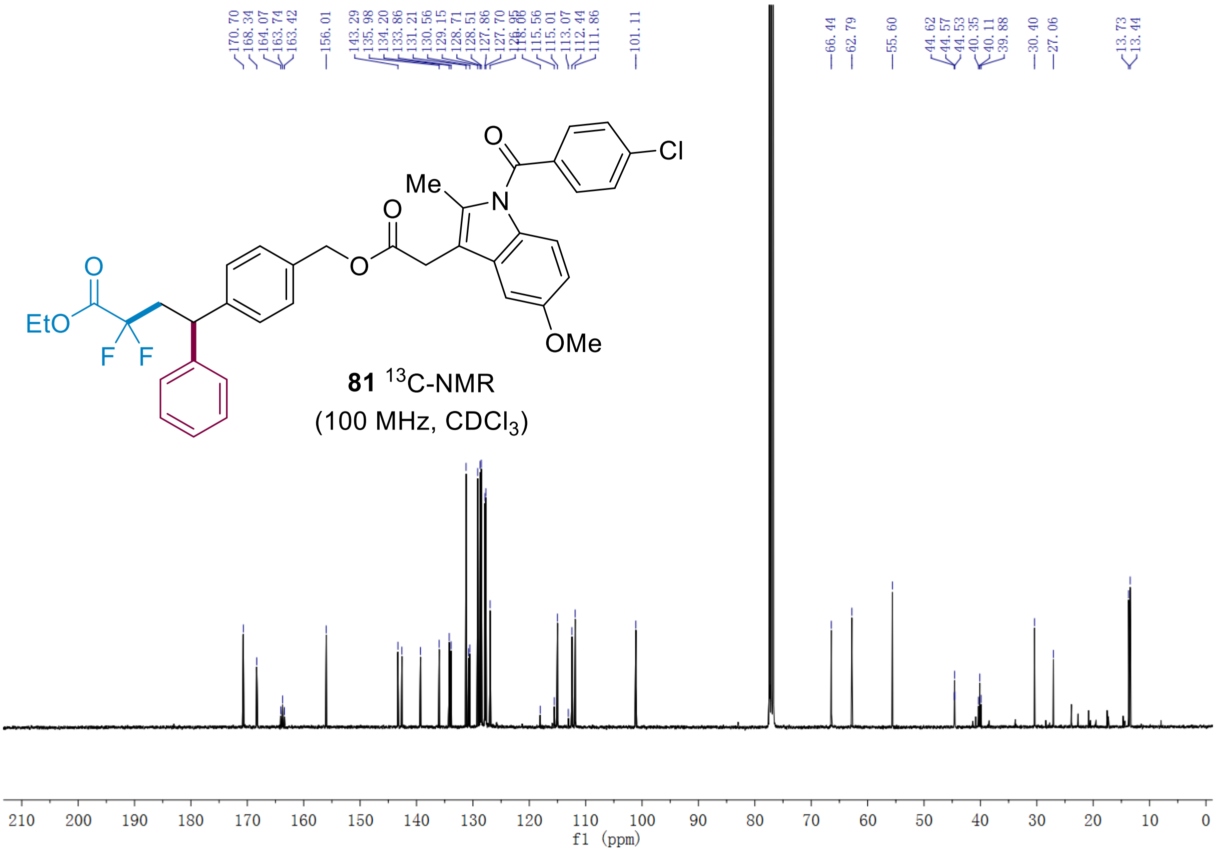

EtO
O
F F
Me
N
O
Cl
OMe
81 13C-NMR
(100 MHz, CDCl3)
f1 (ppm)

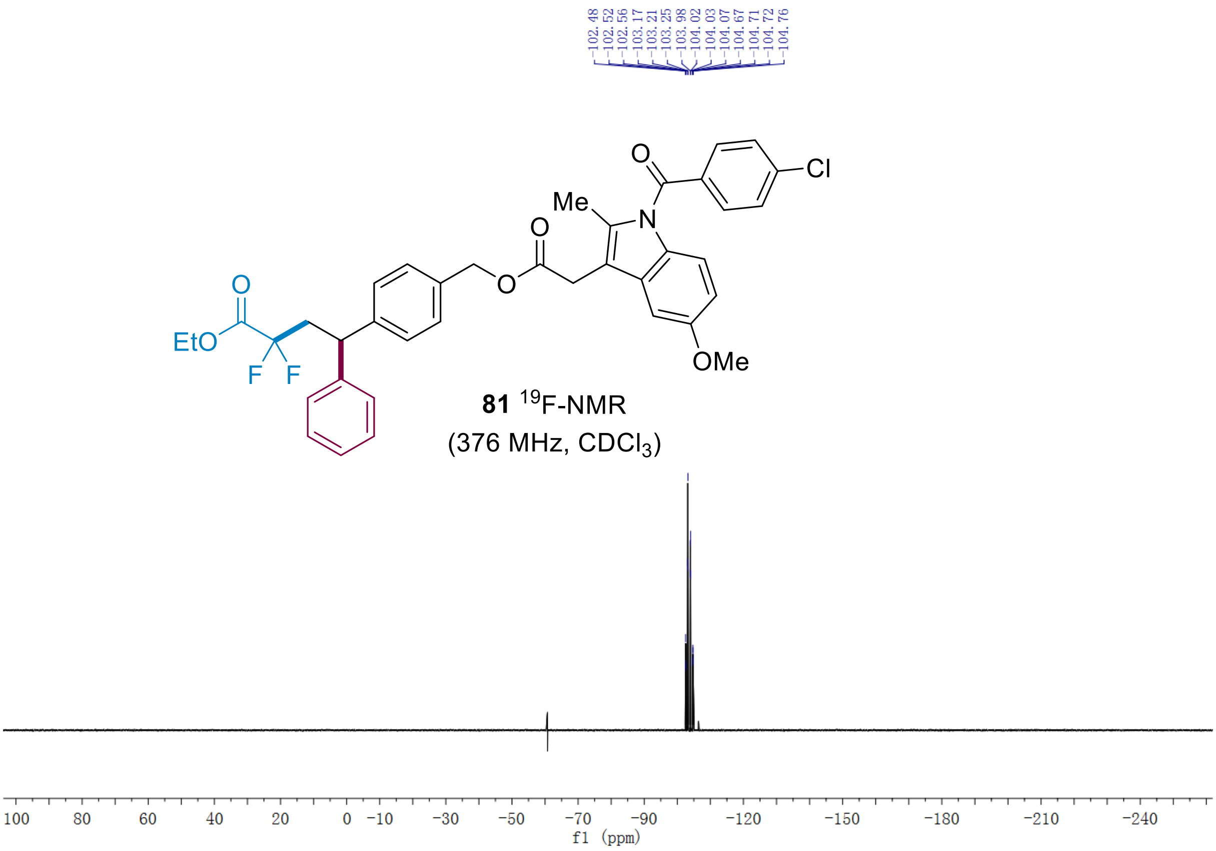
−102.48
−102.52
−102.56
−103.17
−103.21
−103.25
−103.98
−104.02
−104.03
−104.07
−104.67
−104.71
−104.72
−104.76
O
Cl
Me
N
O
O
EtO
F F
OMe
81 ^{19}F-NMR
(376 MHz, $CDCl_3$)
f1 (ppm)

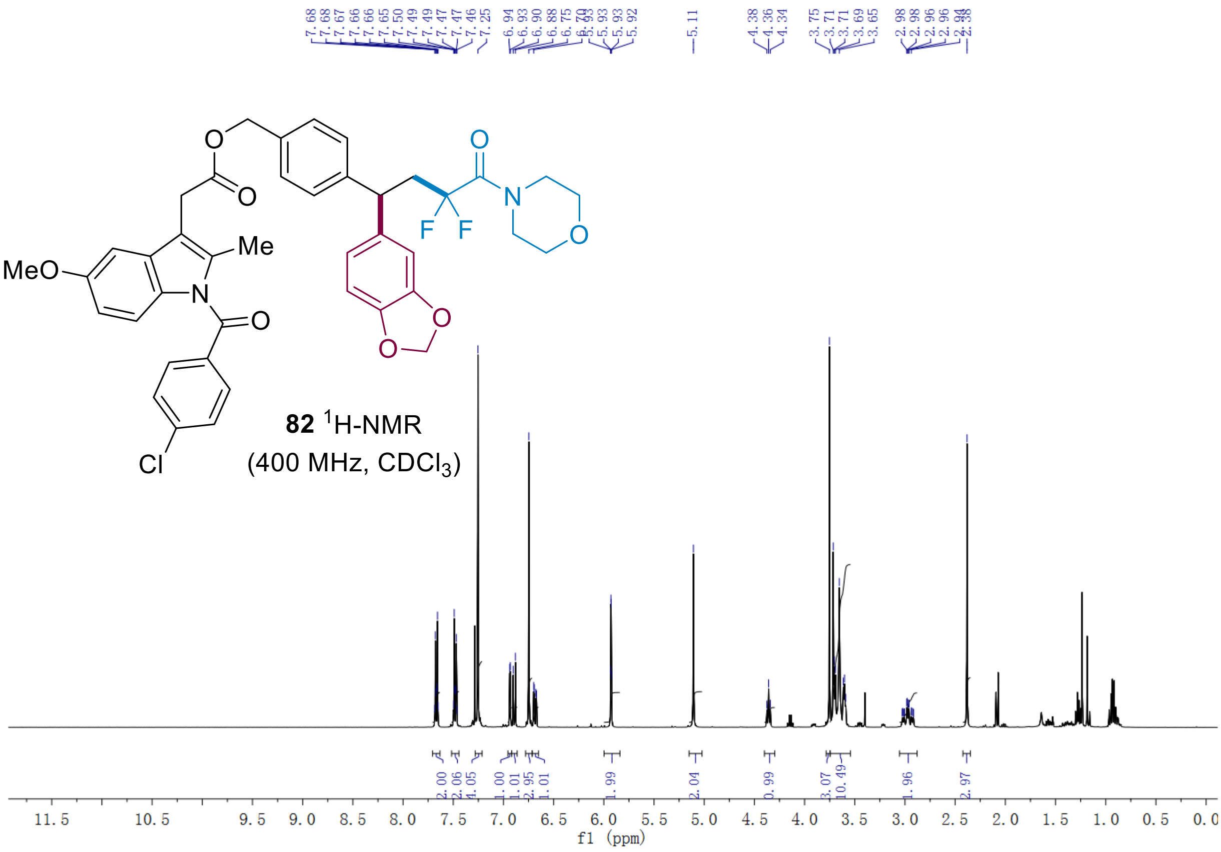
O
O
Me
MeO
N
O
Cl
F F
N
O
O
O
82 ^{1}H-NMR
(400 MHz, $CDCl_3$)
f1 (ppm)

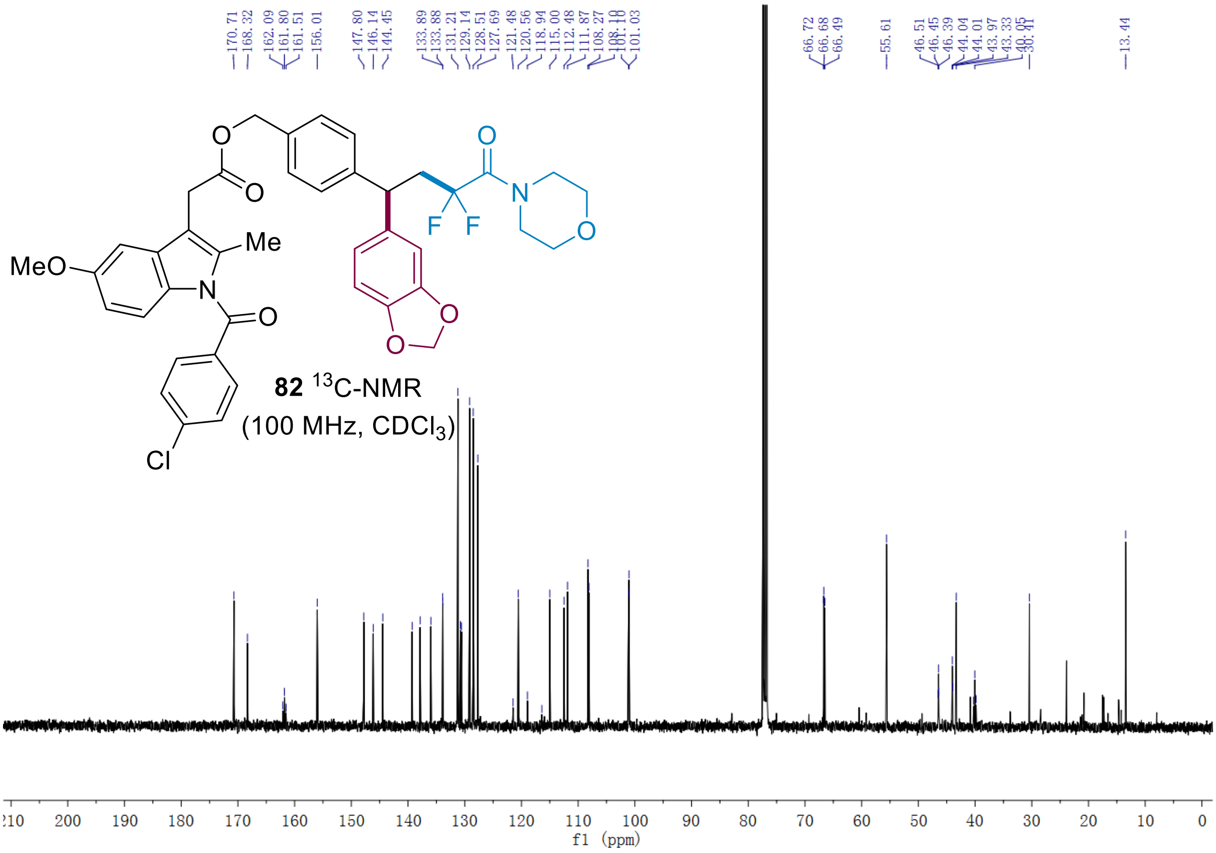
MeO
Me
N
O
Cl
F F
82 ^{13}C-NMR
(100 MHz, $CDCl_3$)
f1 (ppm)

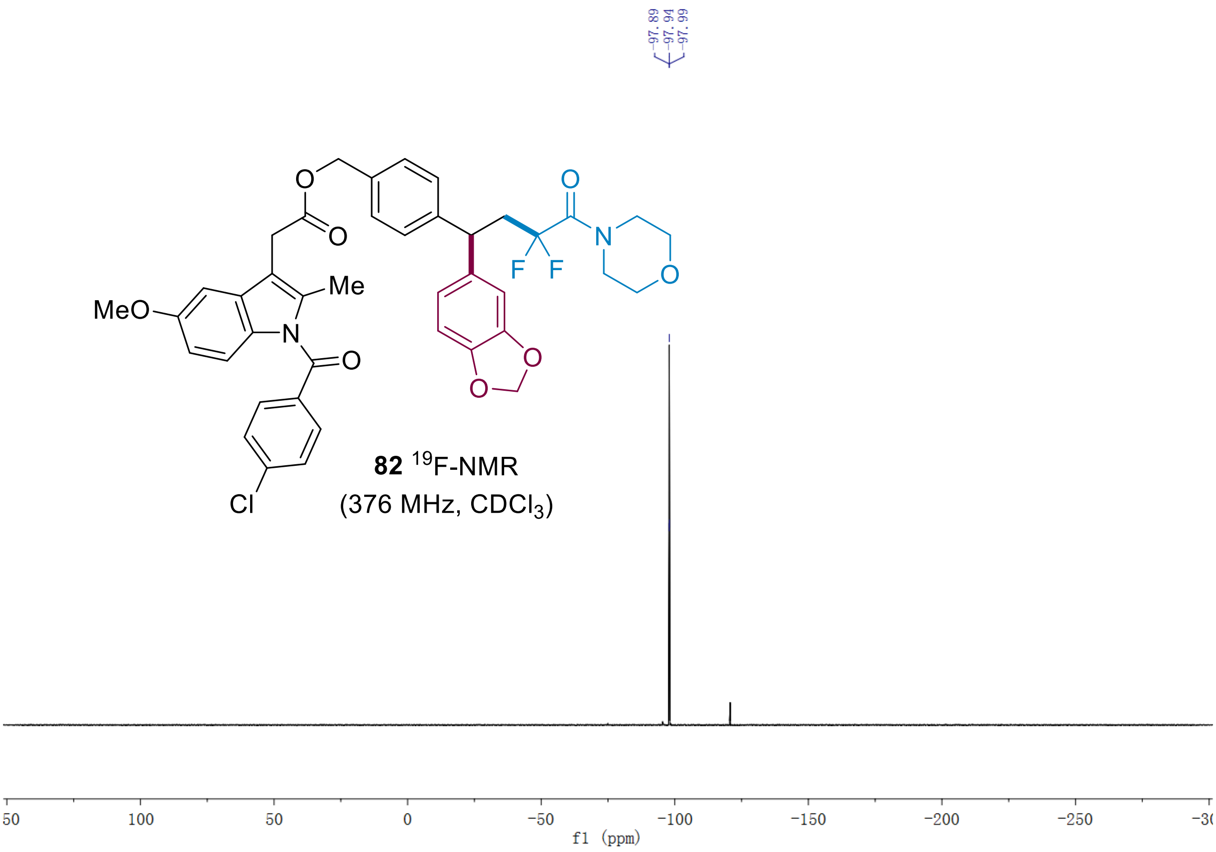
MeO
Me
N
O
Cl
F F
-97.89
-97.94
-97.99
82 ^{19}F-NMR
(376 MHz, $CDCl_3$)
f1 (ppm)

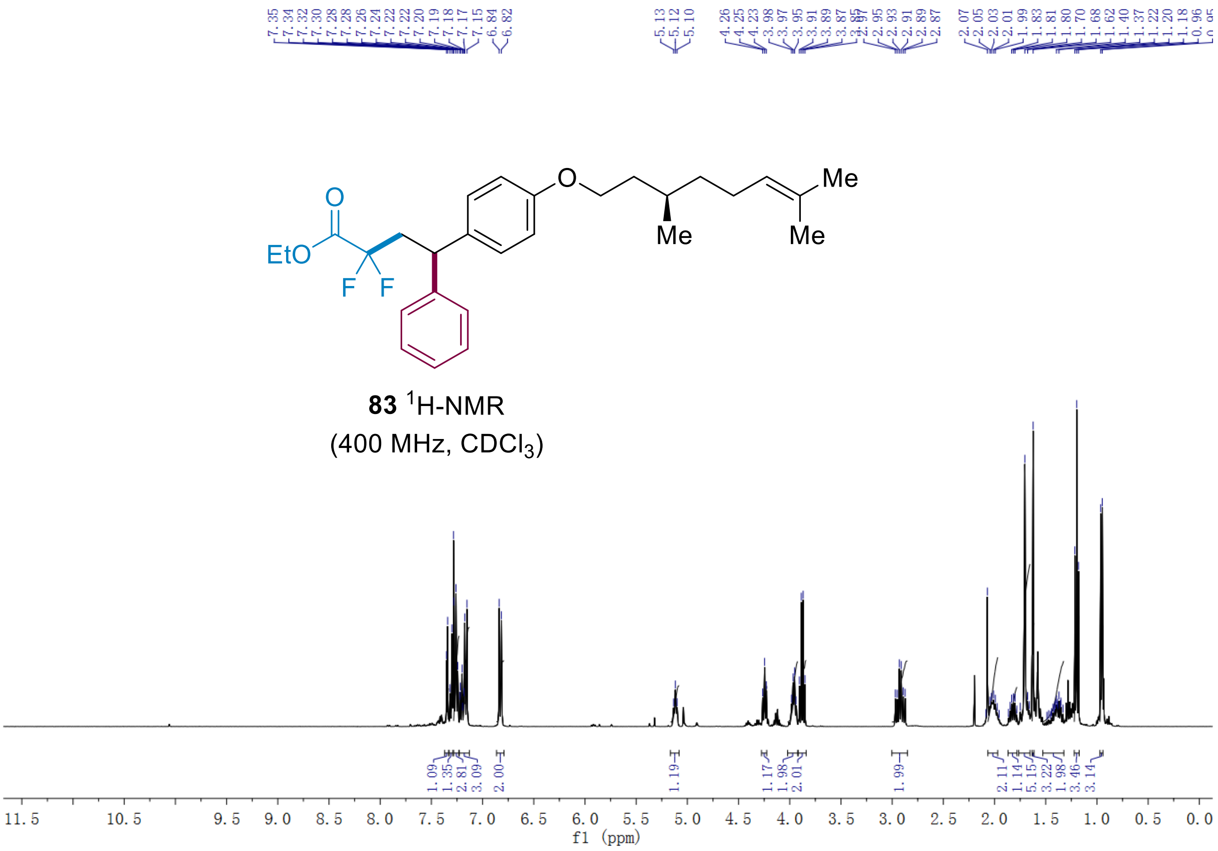

83 ^{1}H-NMR
(400 MHz, $CDCl_3$)

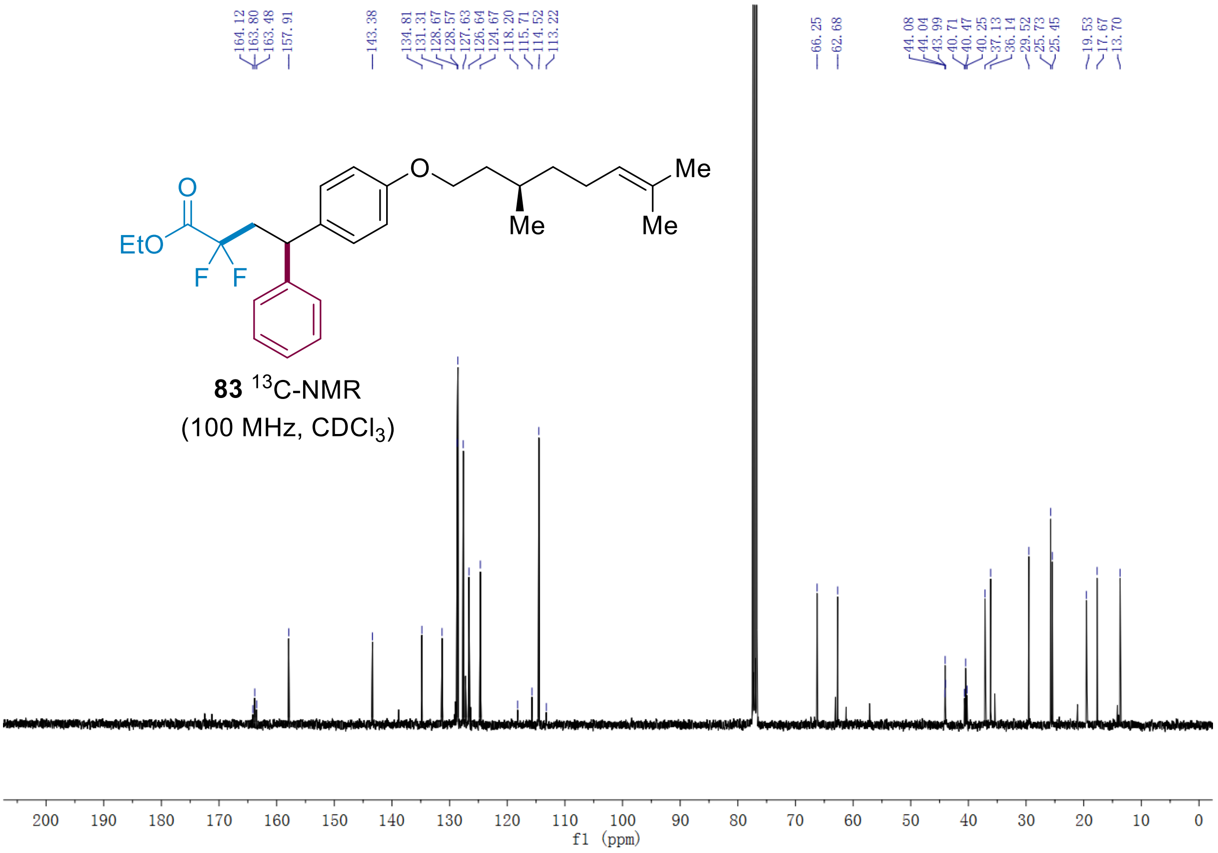

83 ^{13}C-NMR
(100 MHz, $CDCl_3$)

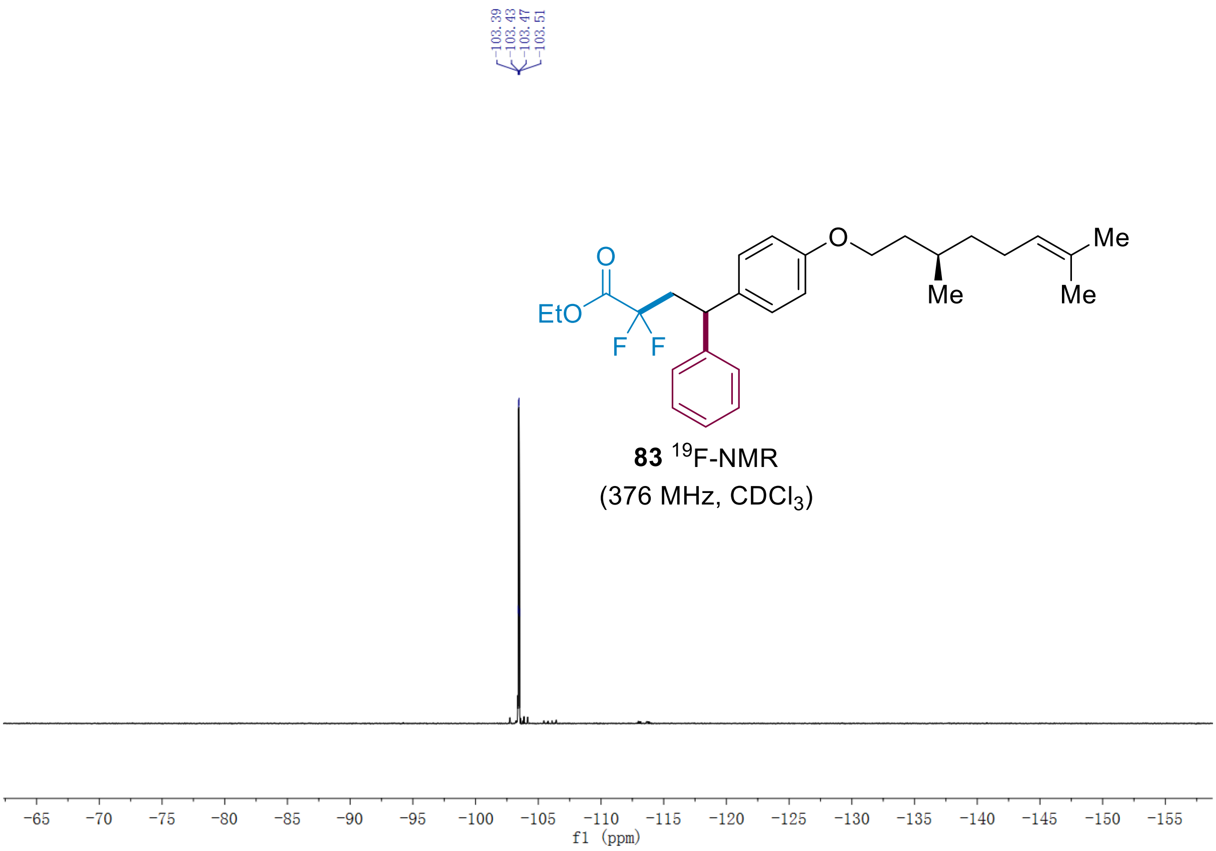

83 ^{19}F-NMR
(376 MHz, $CDCl_3$)

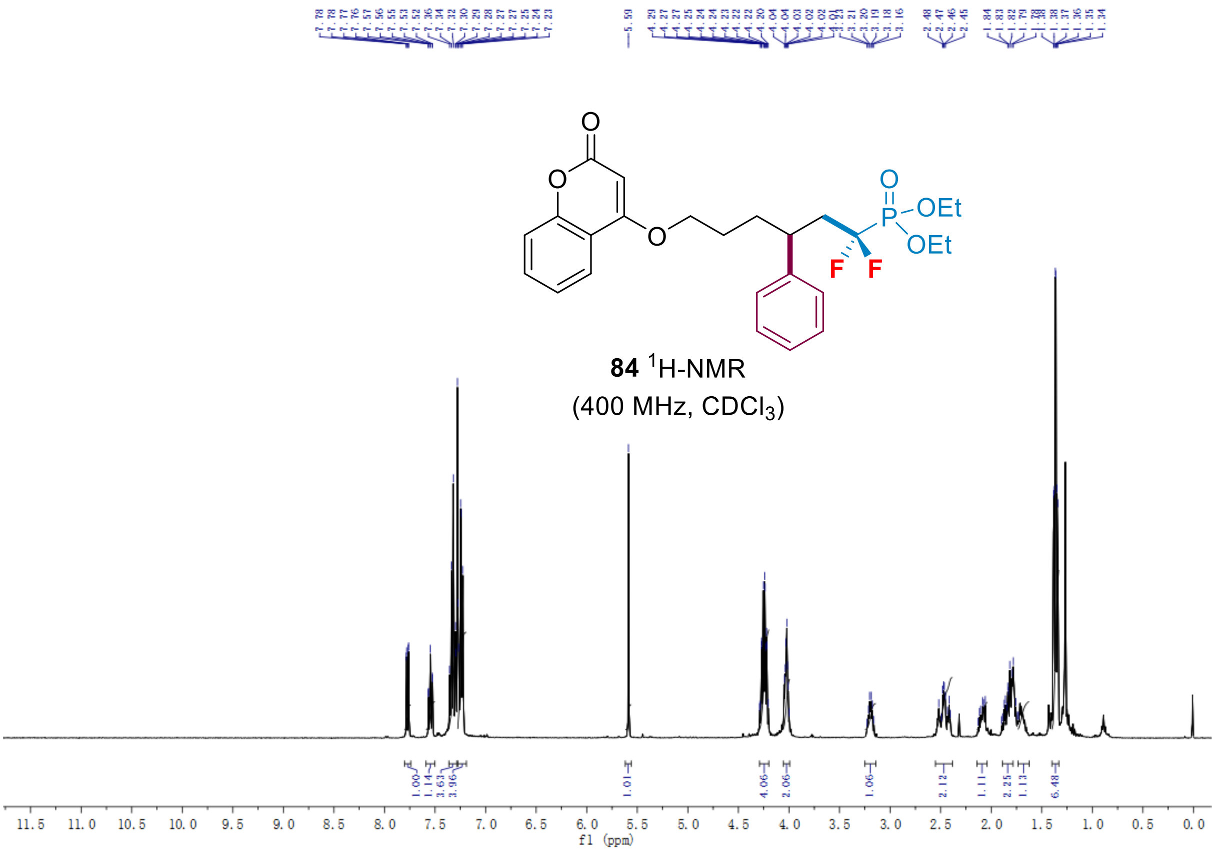

84 ^{1}H-NMR
(400 MHz, $CDCl_3$)

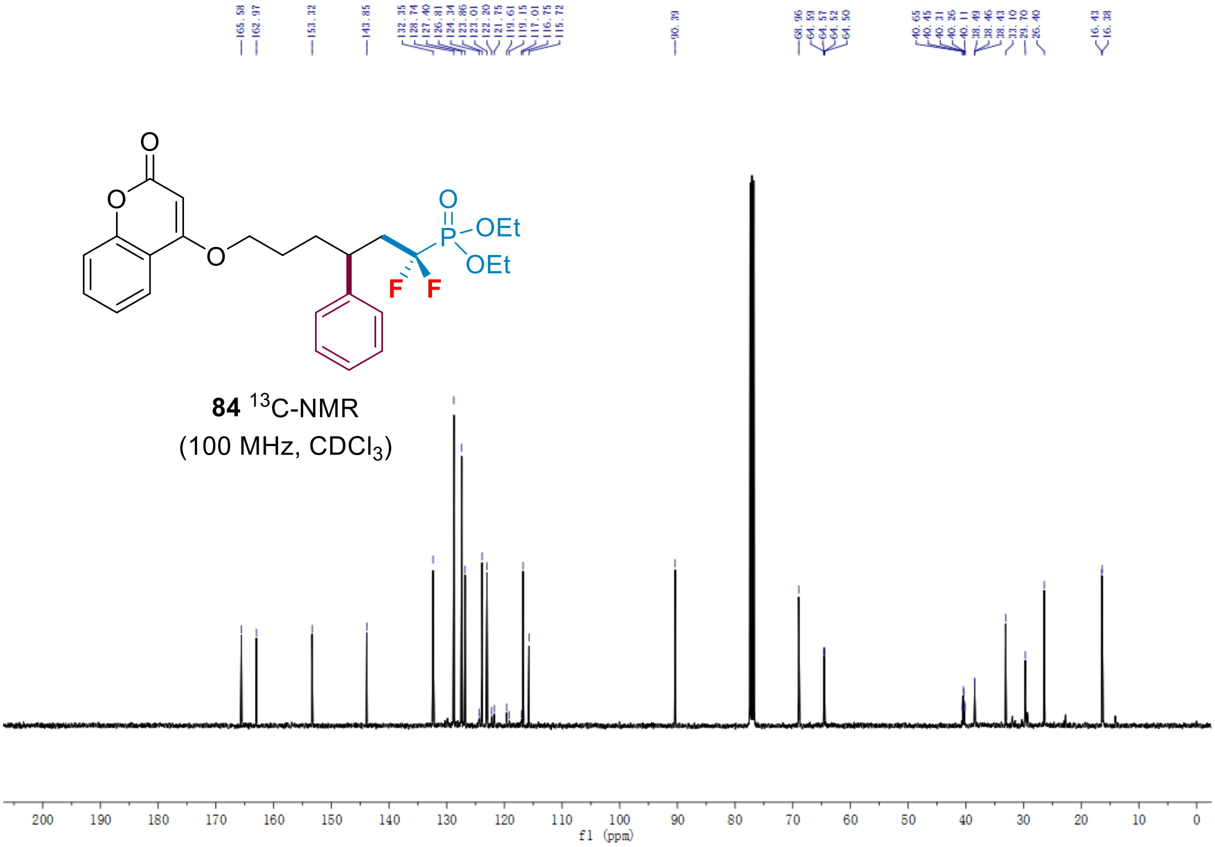

84 ^{13}C-NMR
(100 MHz, $CDCl_3$)

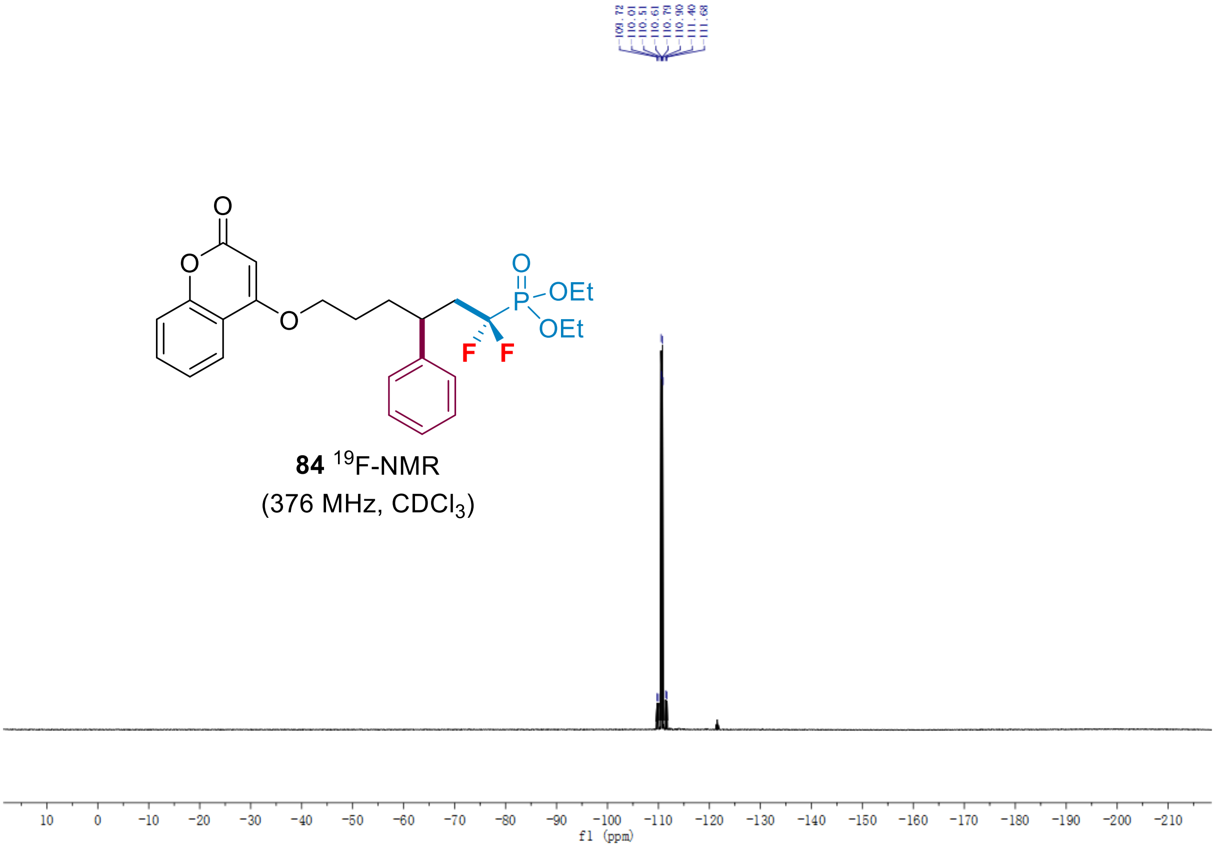

84 ^{19}F-NMR
(376 MHz, $CDCl_3$)

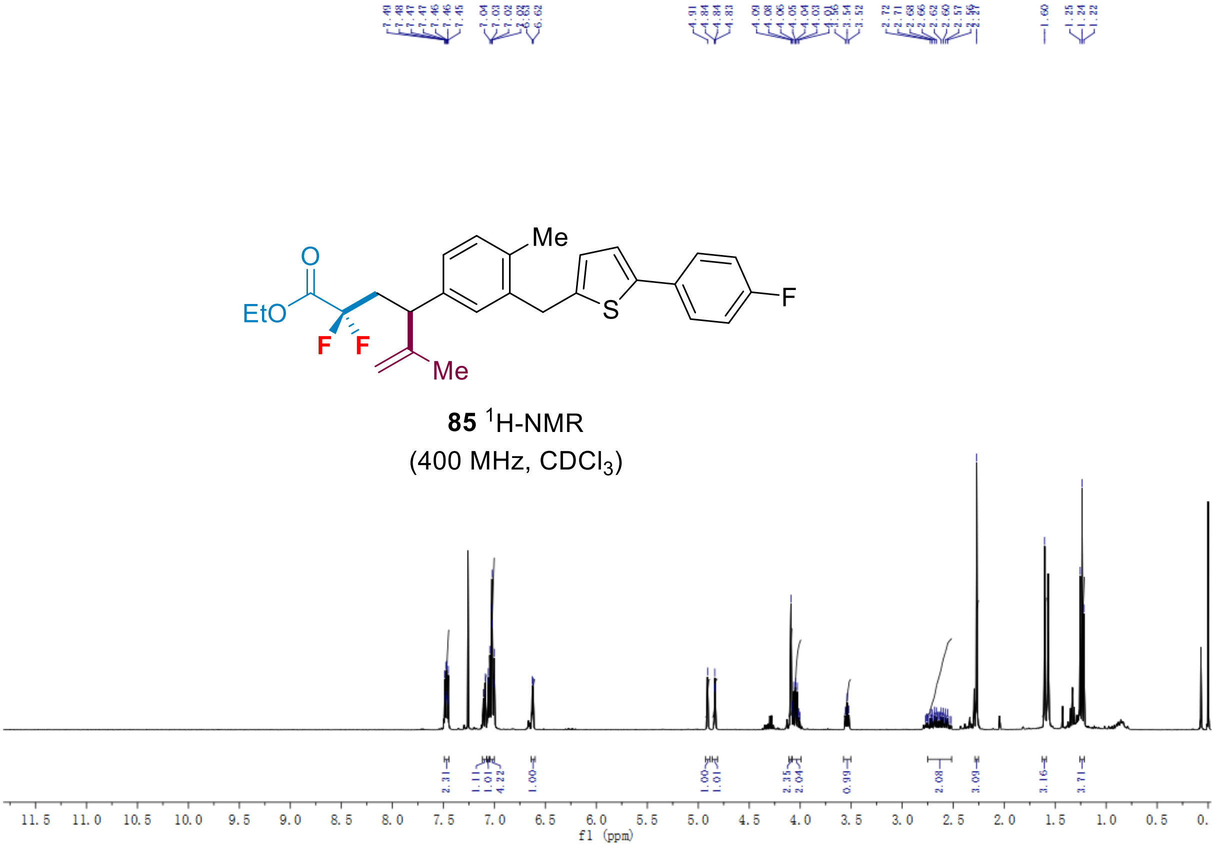
Me
EtO
O
F F
Me
S
F
85 1H-NMR
(400 MHz, CDCl3)
f1 (ppm)

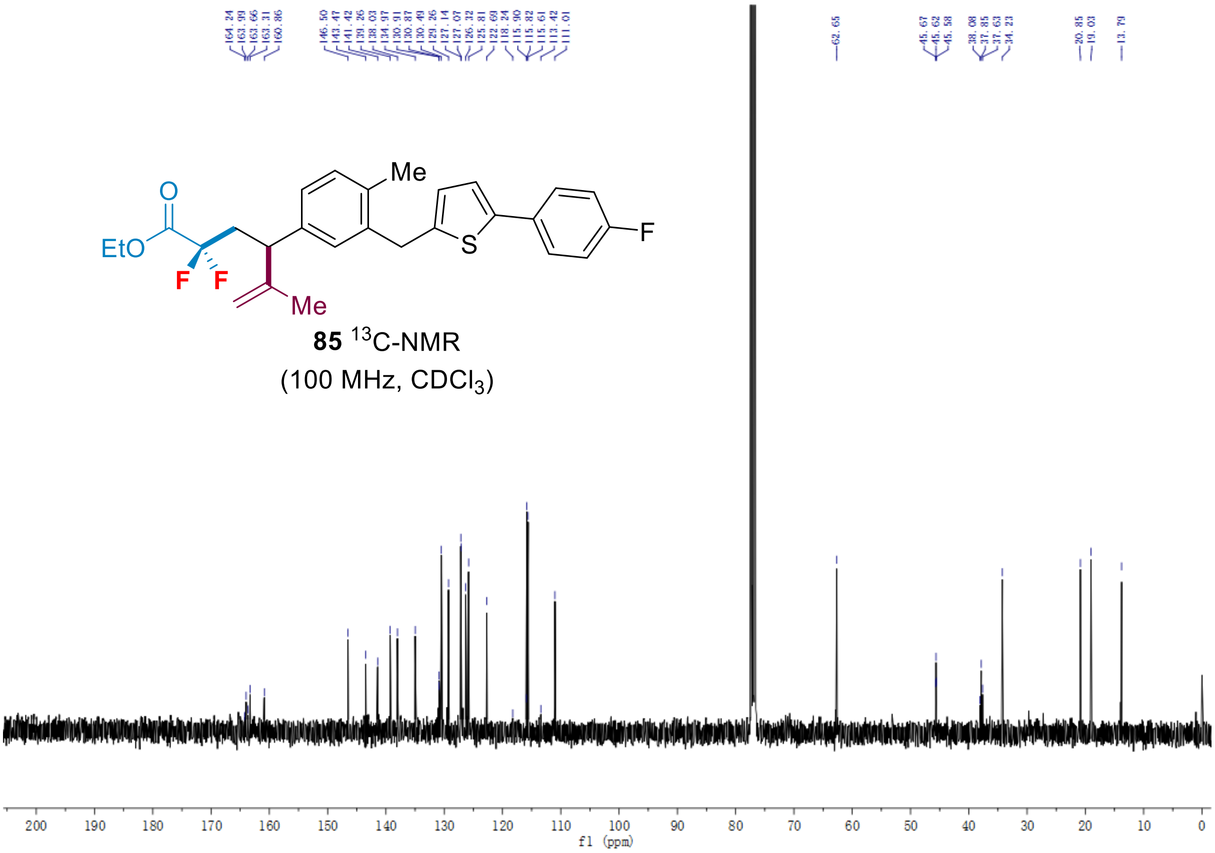
Me
EtO
O
F F
Me
S
F
85 13C-NMR
(100 MHz, CDCl3)
f1 (ppm)

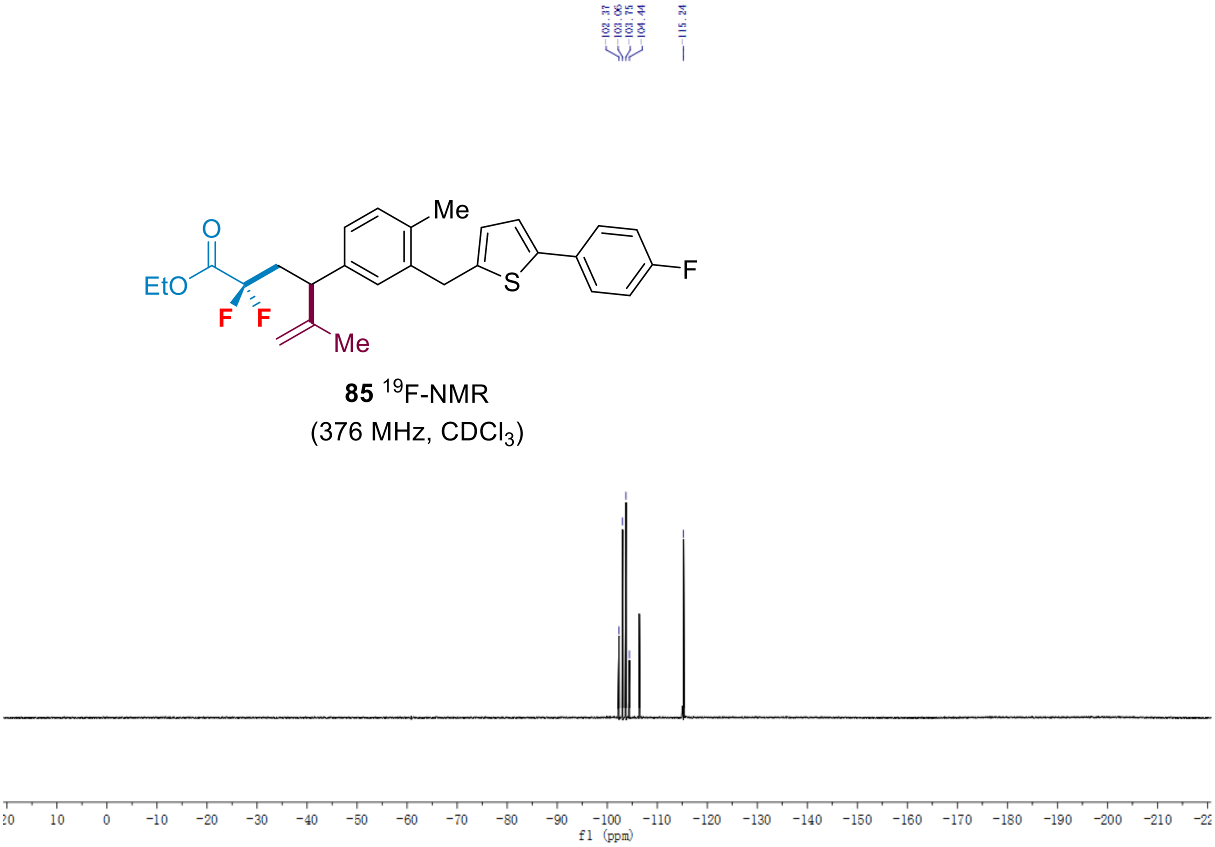

85 ^{19}F-NMR
(376 MHz, $CDCl_3$)

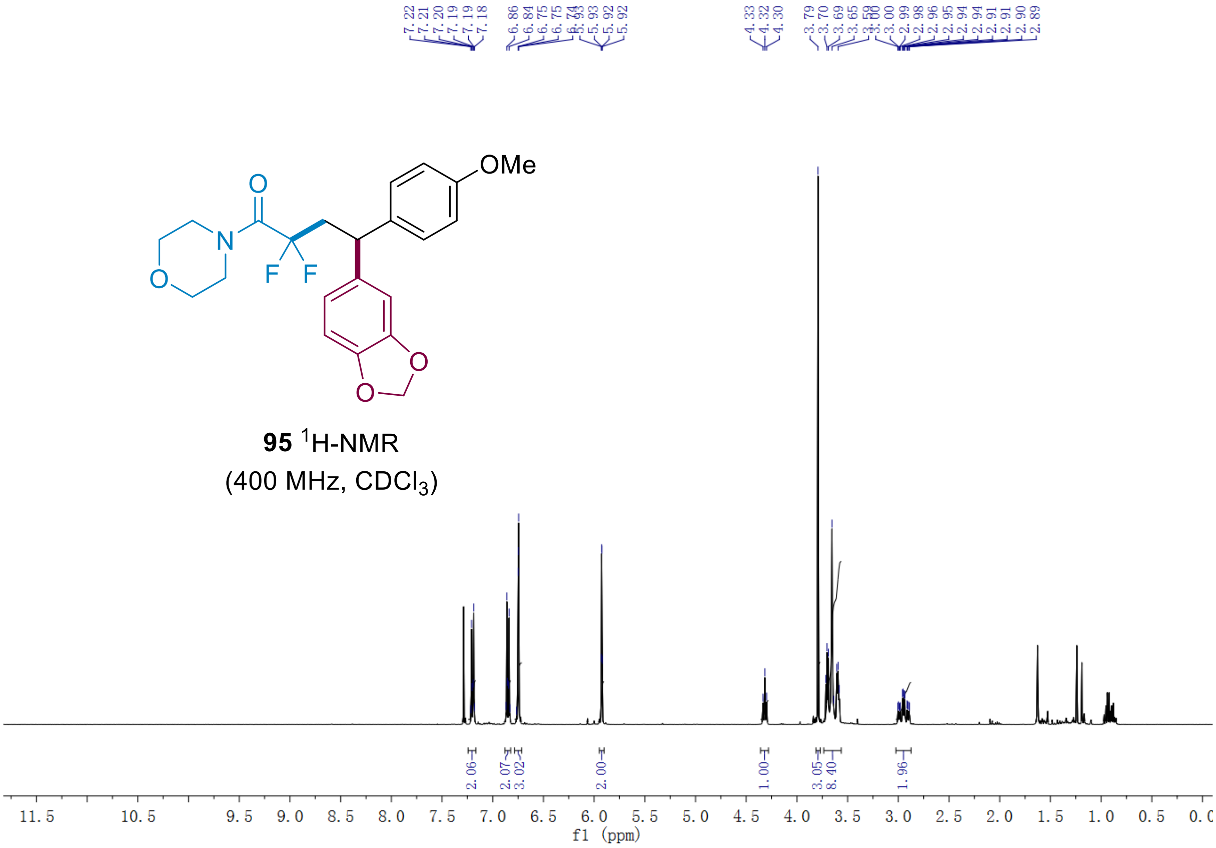

95 ^{1}H-NMR
(400 MHz, $CDCl_3$)

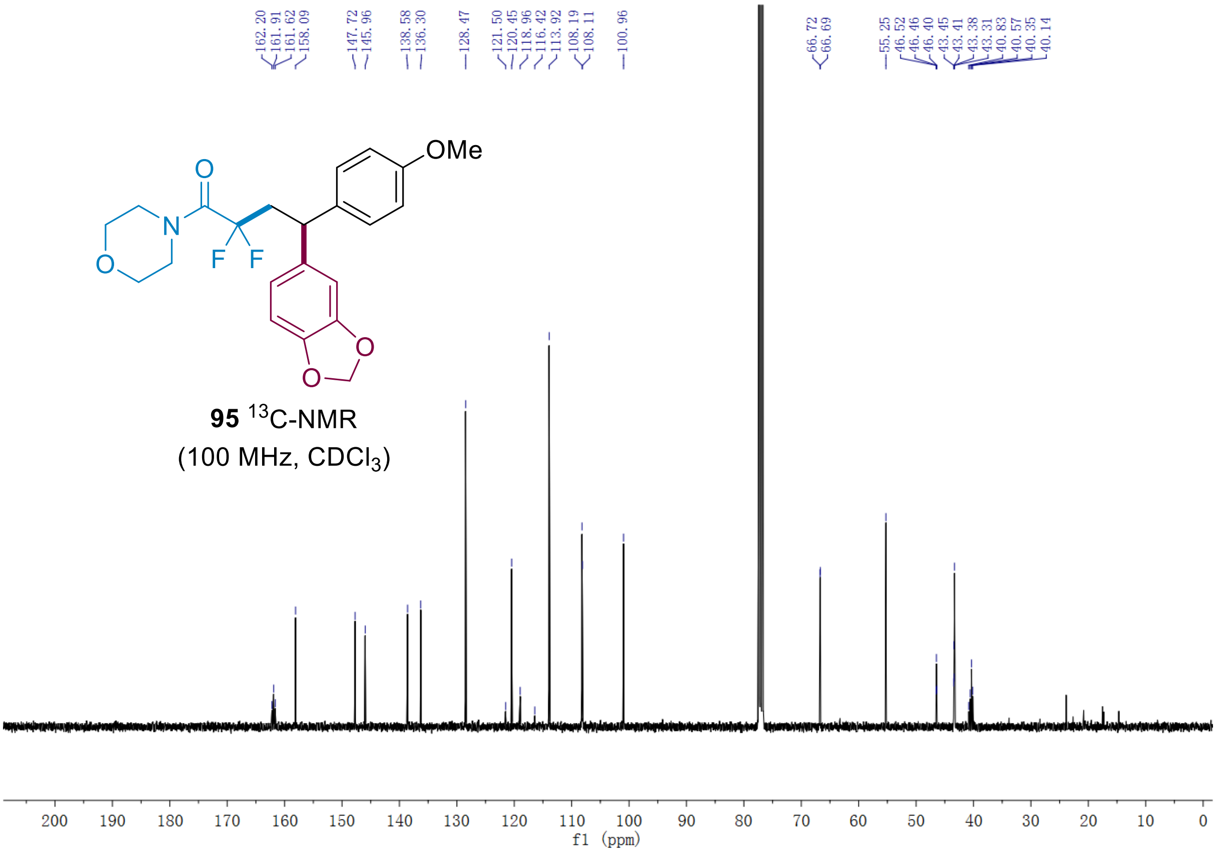
162.20
161.91
161.62
158.09
147.72
145.96
138.58
136.30
128.47
121.50
120.45
118.96
116.42
113.92
108.19
108.11
100.96
66.72
66.69
55.25
46.52
46.46
46.40
43.45
43.41
43.38
43.31
40.83
40.57
40.35
40.14
OMe
O
N
O
F F
O
O
95 13C-NMR
(100 MHz, CDCl3)
f1 (ppm)

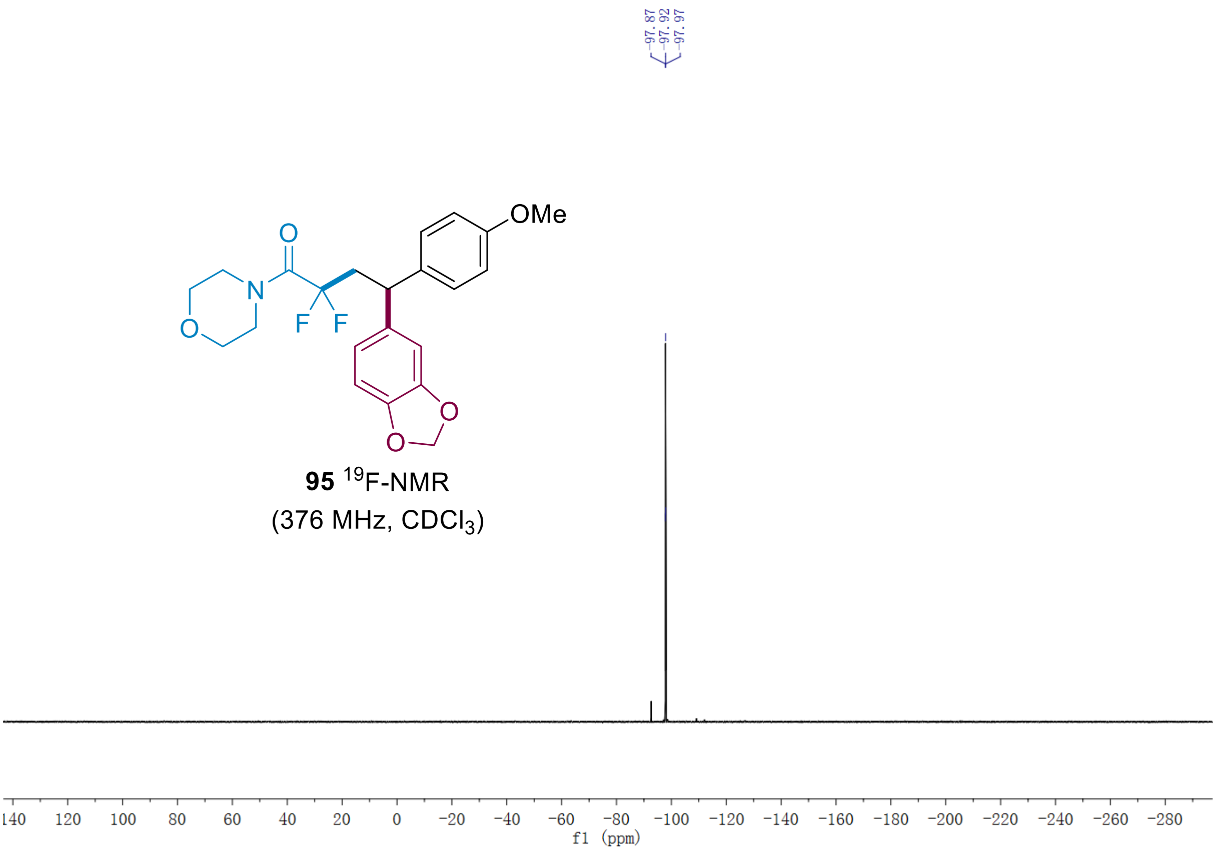
-97.87
-97.92
-97.97
OMe
O
N
O
F F
O
O
95 19F-NMR
(376 MHz, CDCl3)
f1 (ppm)

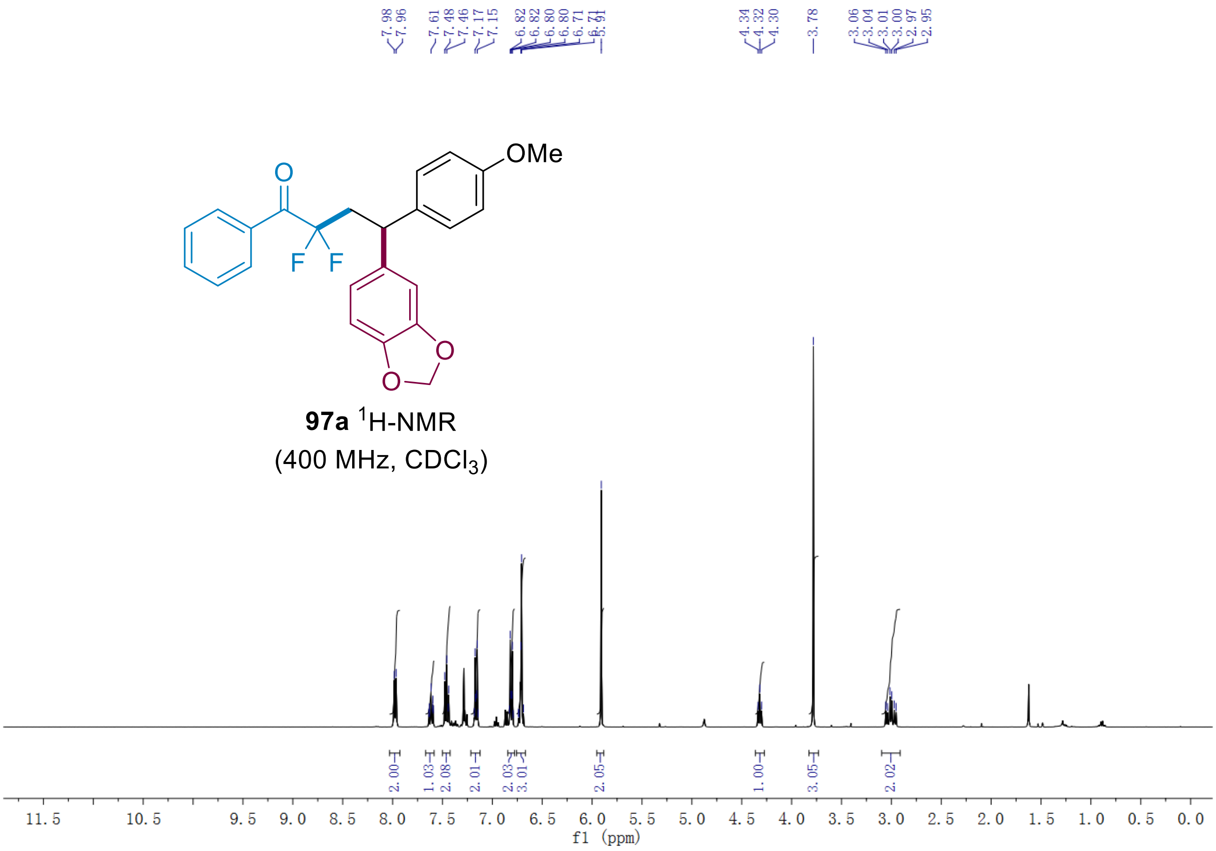
OMe
O
F F
O
O
97a ^{1}H-NMR
(400 MHz, CDCl$_3$)
f1 (ppm)

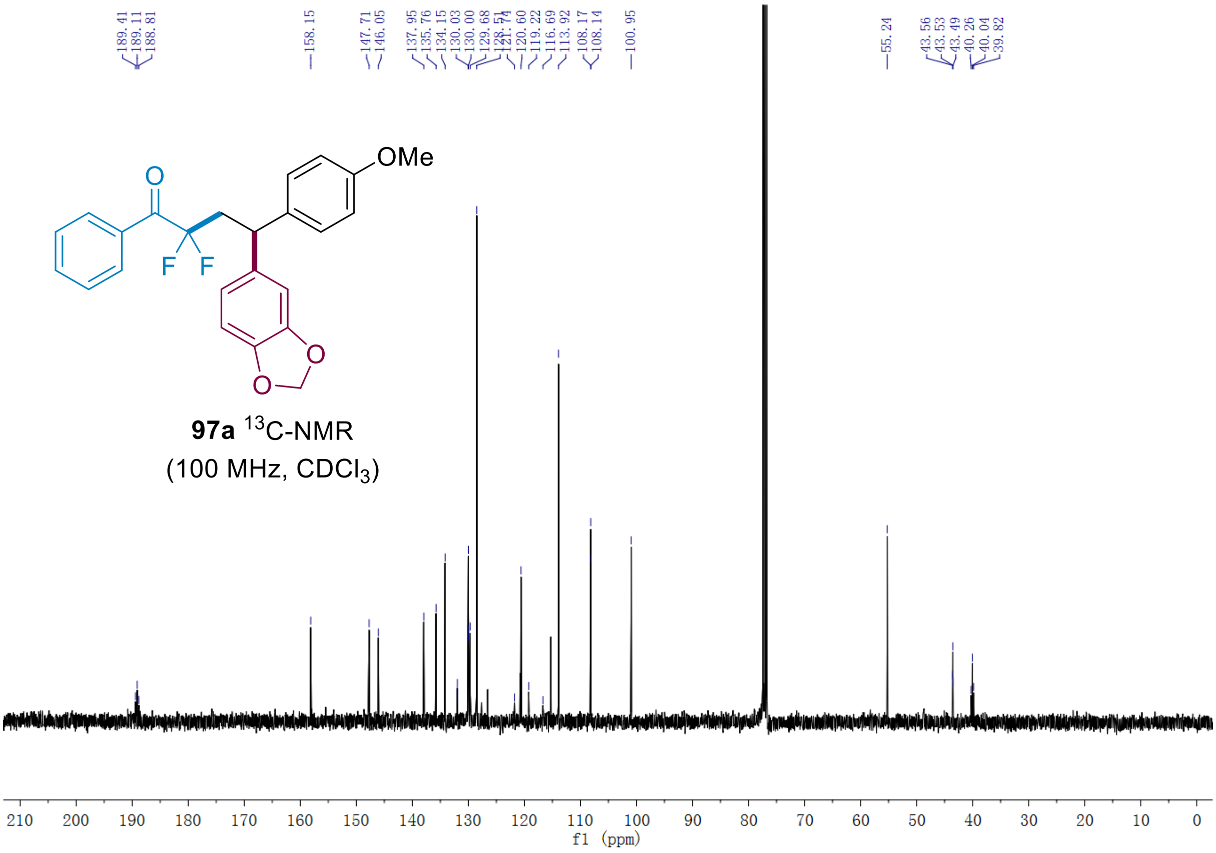
OMe
O
F F
O
O
97a ^{13}C-NMR
(100 MHz, CDCl$_3$)
f1 (ppm)

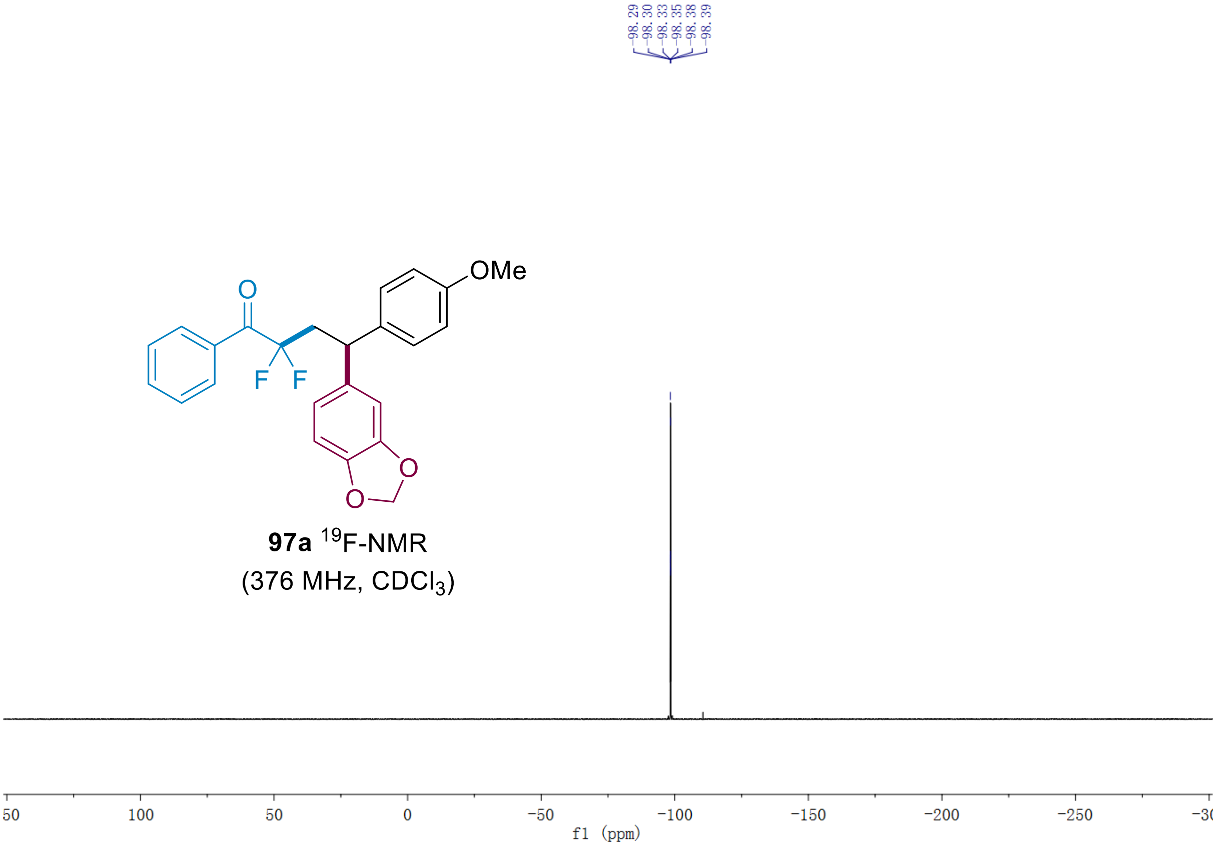

97a ^{19}F-NMR
(376 MHz, $CDCl_3$)

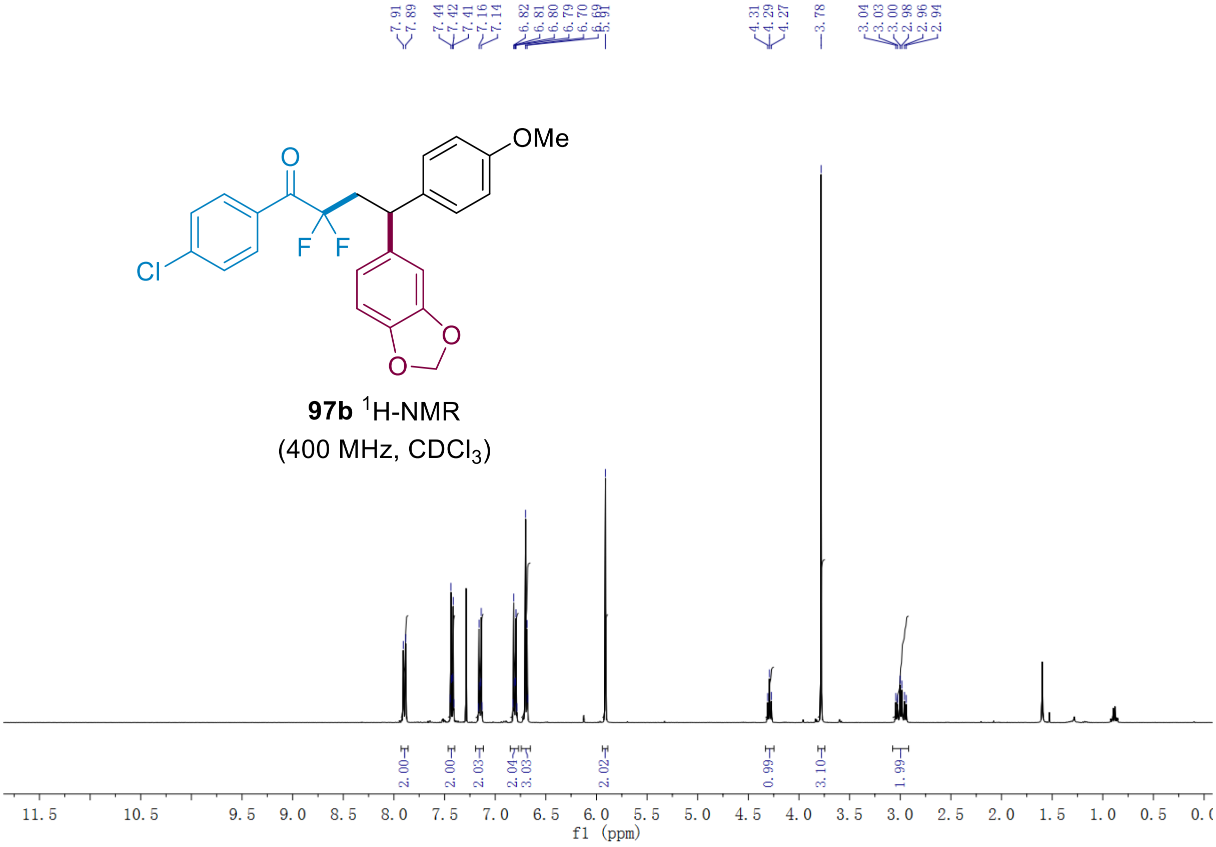

97b ^{1}H-NMR
(400 MHz, $CDCl_3$)

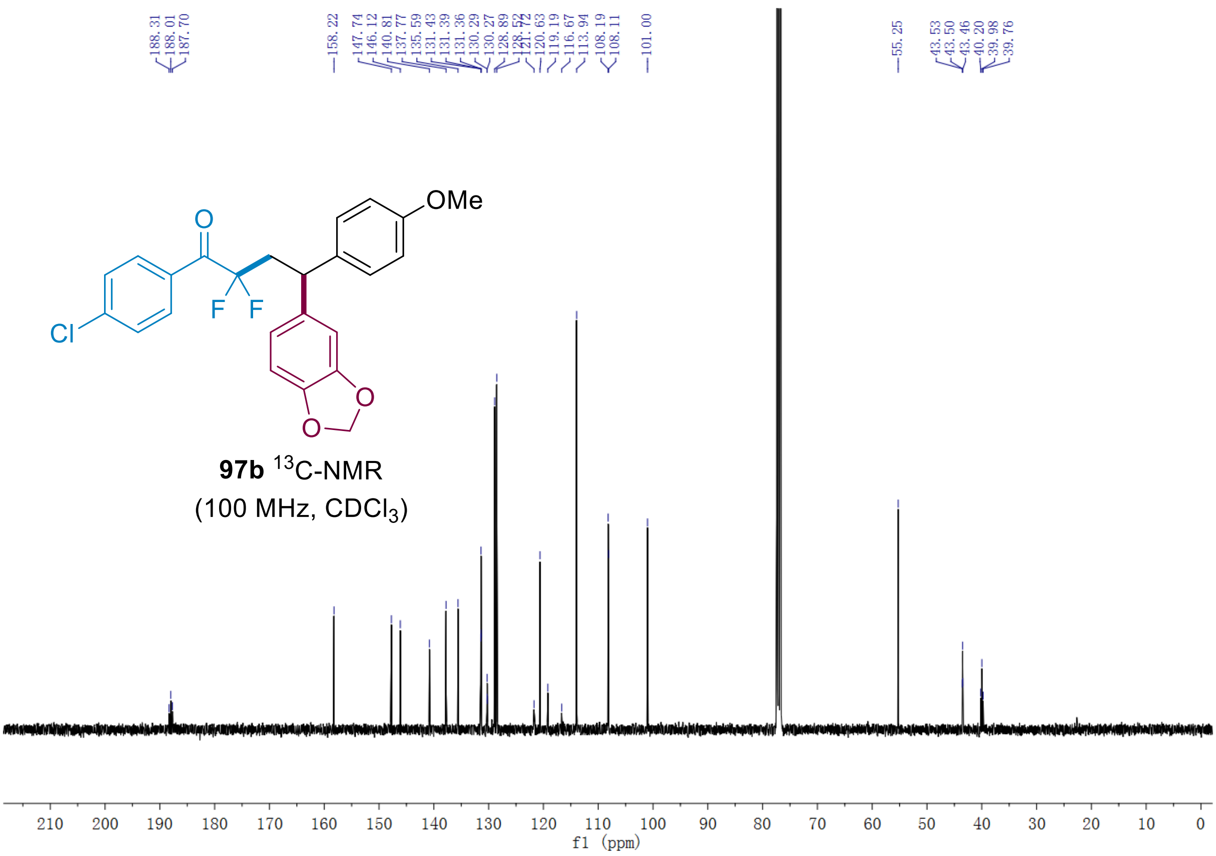

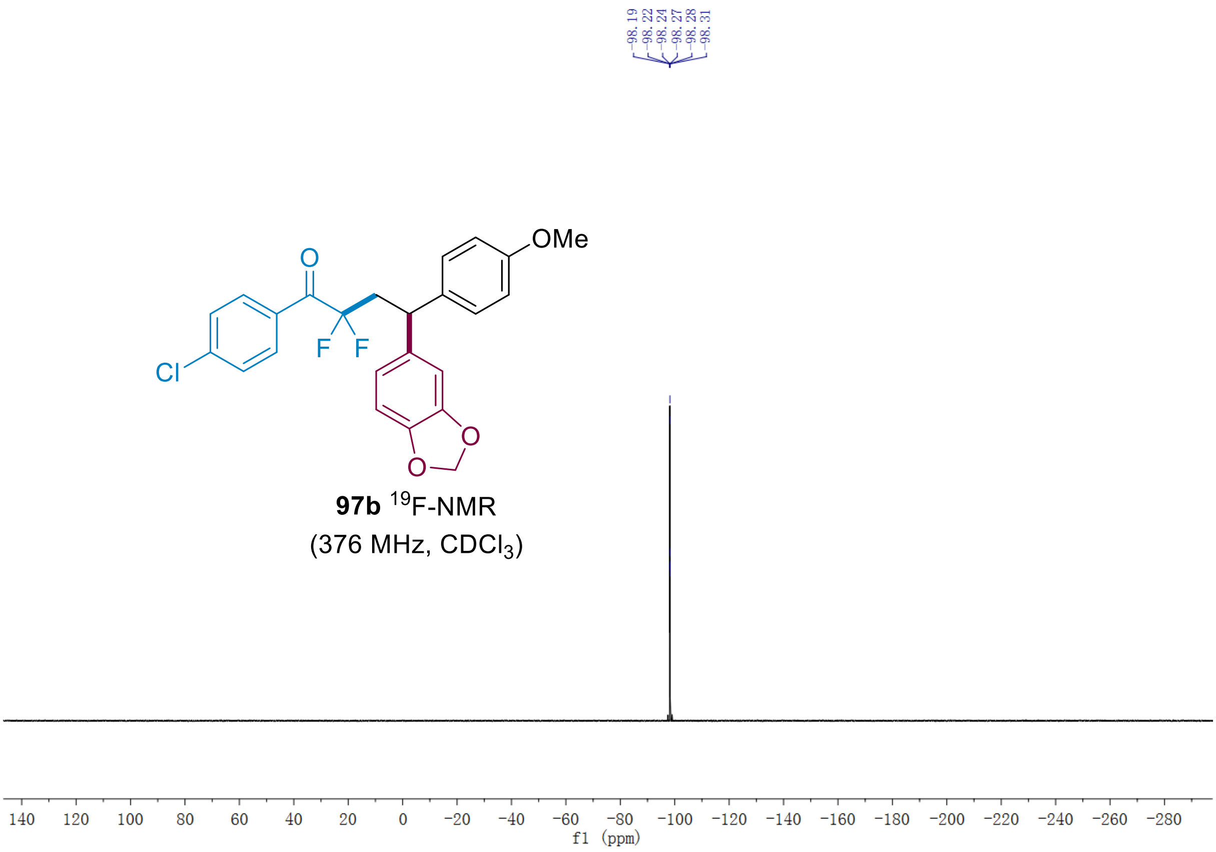

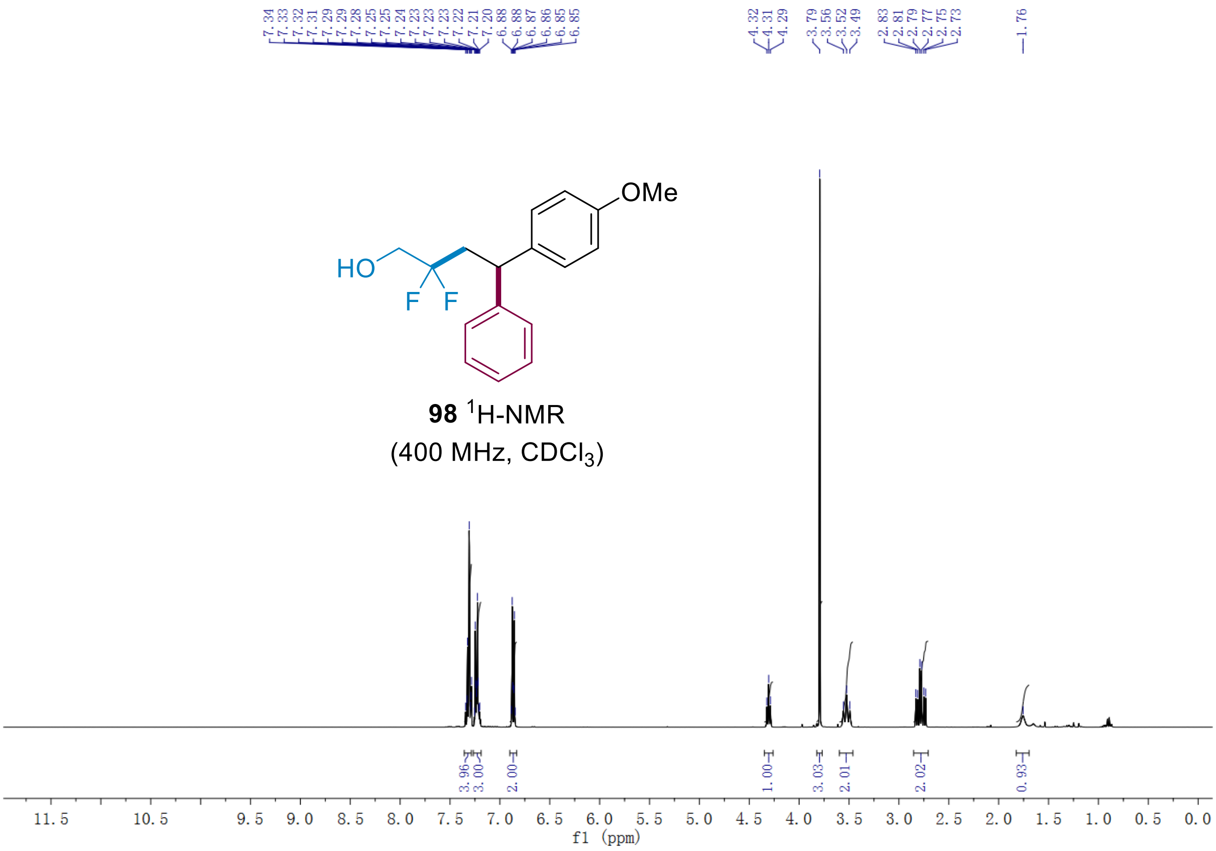
7.34
7.33
7.32
7.31
7.29
7.29
7.28
7.25
7.25
7.24
7.23
7.23
7.23
7.22
7.22
7.21
7.20
6.88
6.88
6.87
6.86
6.85
6.85
4.32
4.31
4.29
3.79
3.56
3.52
3.49
2.83
2.81
2.79
2.77
2.75
2.73
1.76
OMe
HO
F F
98 1H-NMR
(400 MHz, CDCl3)
3.96
3.00
2.00
1.00
3.03
2.01
2.02
0.93
f1 (ppm)

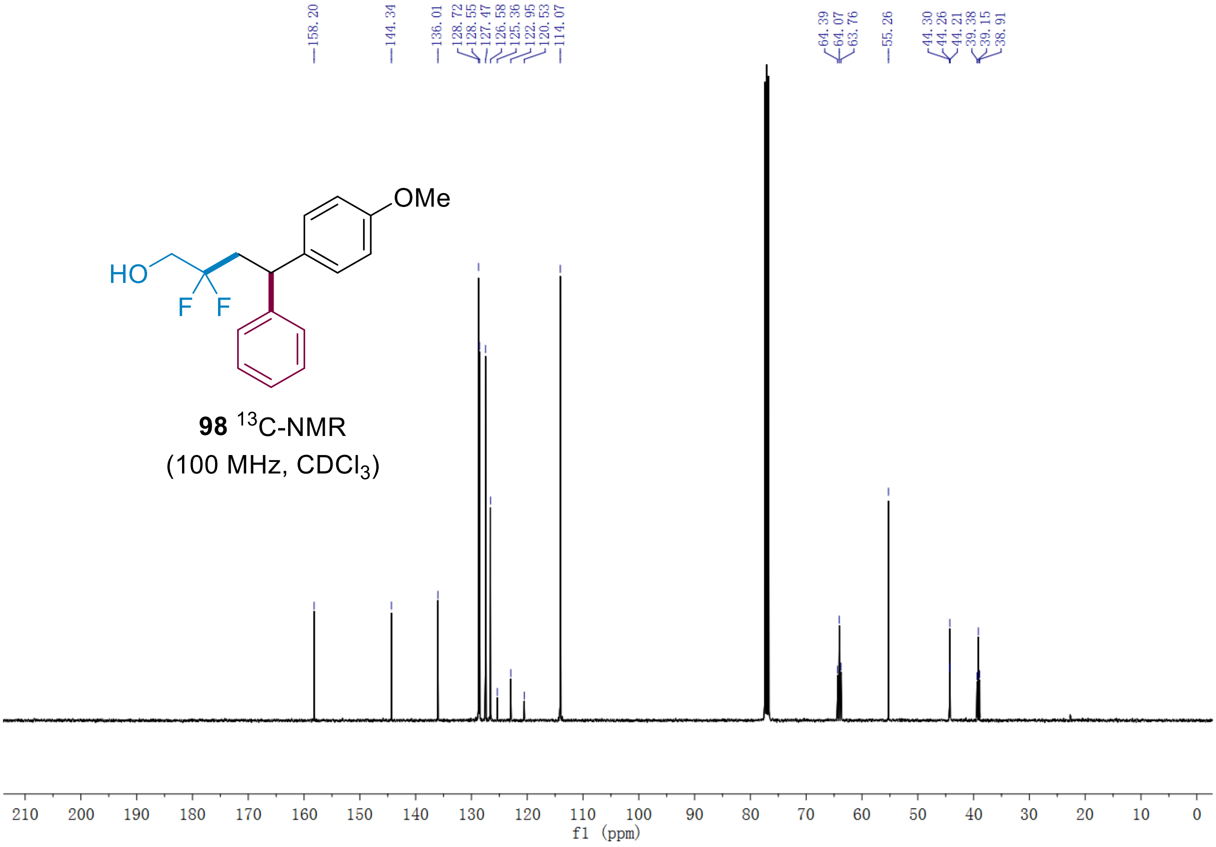
158.20
144.34
136.01
128.72
128.55
127.47
126.58
125.36
122.95
120.53
114.07
64.39
64.07
63.76
55.26
44.30
44.26
44.21
39.38
39.15
38.91
OMe
HO
F F
98 13C-NMR
(100 MHz, CDCl3)
f1 (ppm)

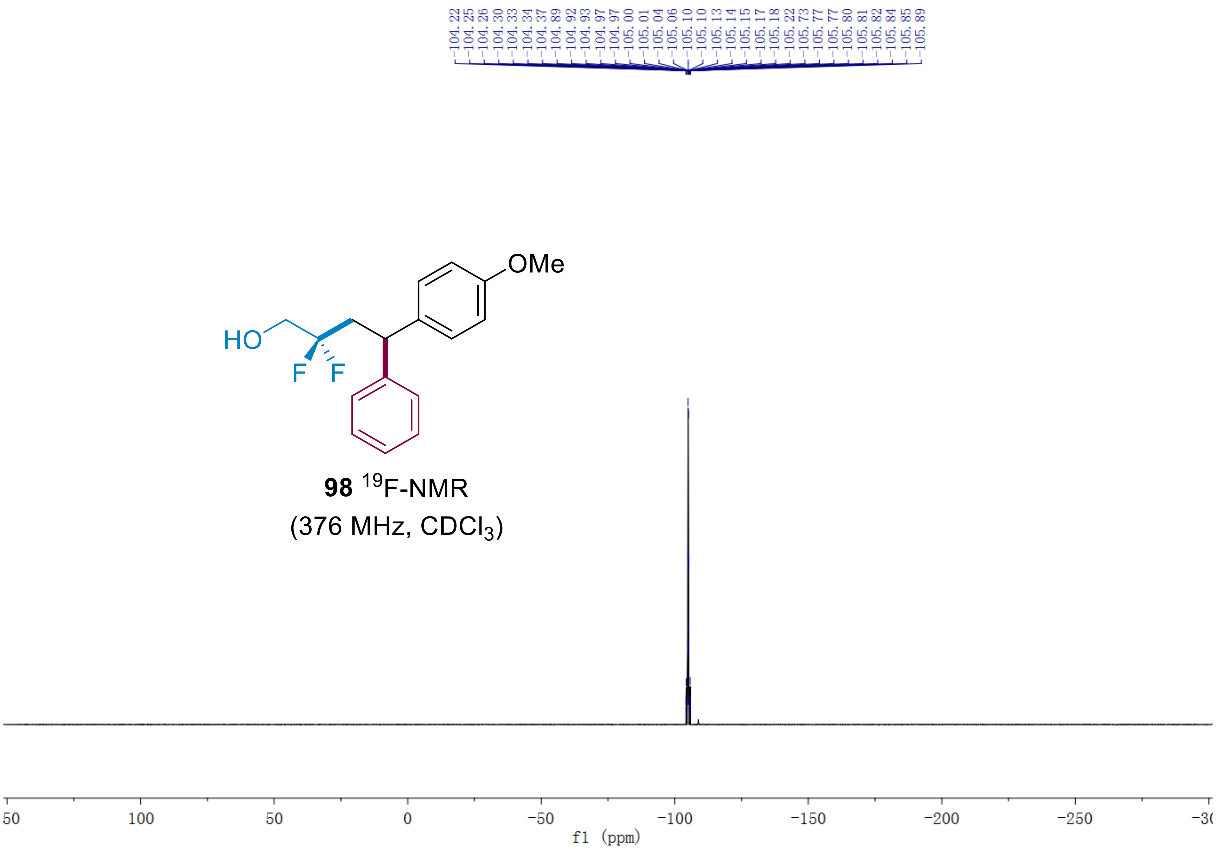
-104.22 -104.25 -104.26 -104.30 -104.33 -104.34 -104.37 -104.89 -104.92 -104.93 -104.97 -104.97 -105.00 -105.01 -105.04 -105.06 -105.10 -105.10 -105.13 -105.14 -105.15 -105.17 -105.18 -105.22 -105.73 -105.77 -105.77 -105.80 -105.81 -105.82 -105.84 -105.85 -105.89
OMe
HO
F
F
98 19F-NMR
(376 MHz, CDCl3)
f1 (ppm)

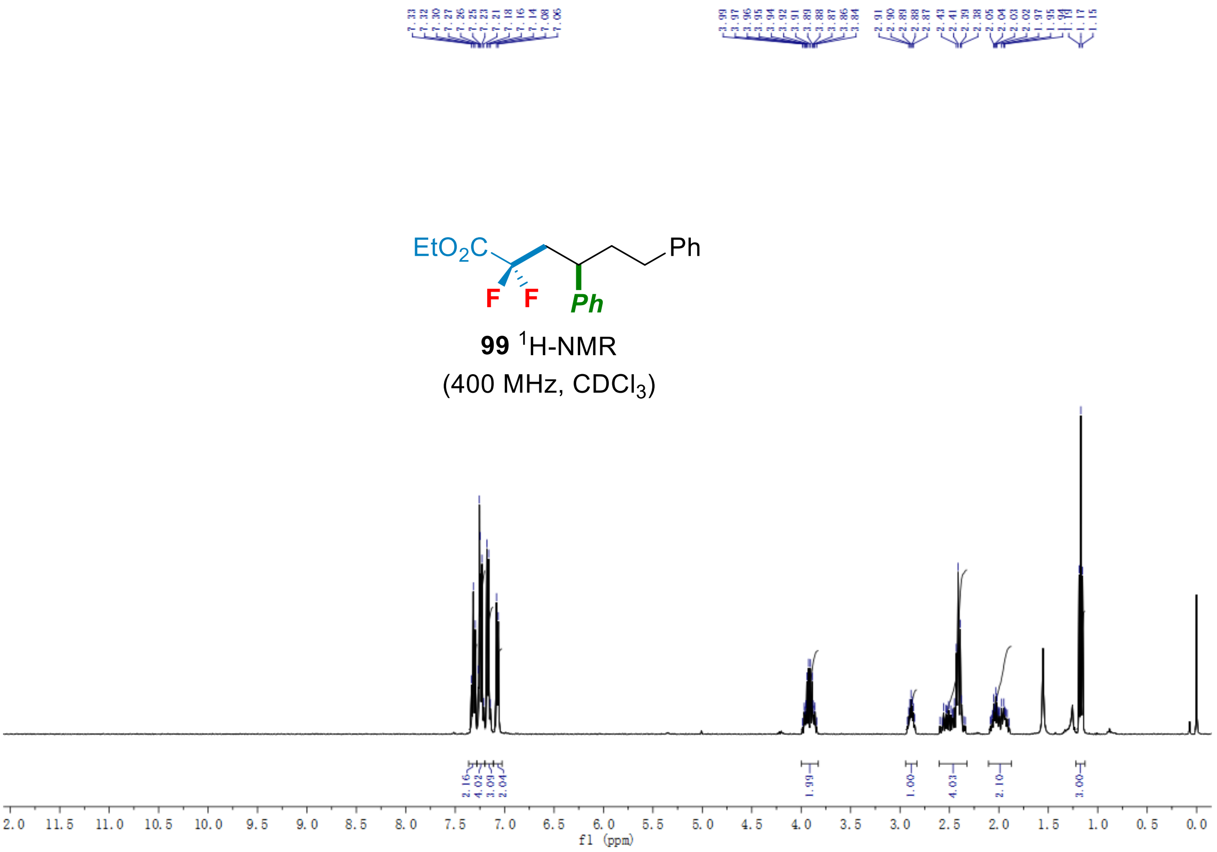
EtO2C
Ph
F
F
Ph
99 1H-NMR
(400 MHz, CDCl3)
2.16 4.02 3.09 2.04
1.99
1.00
4.03
2.10
3.00
f1 (ppm)

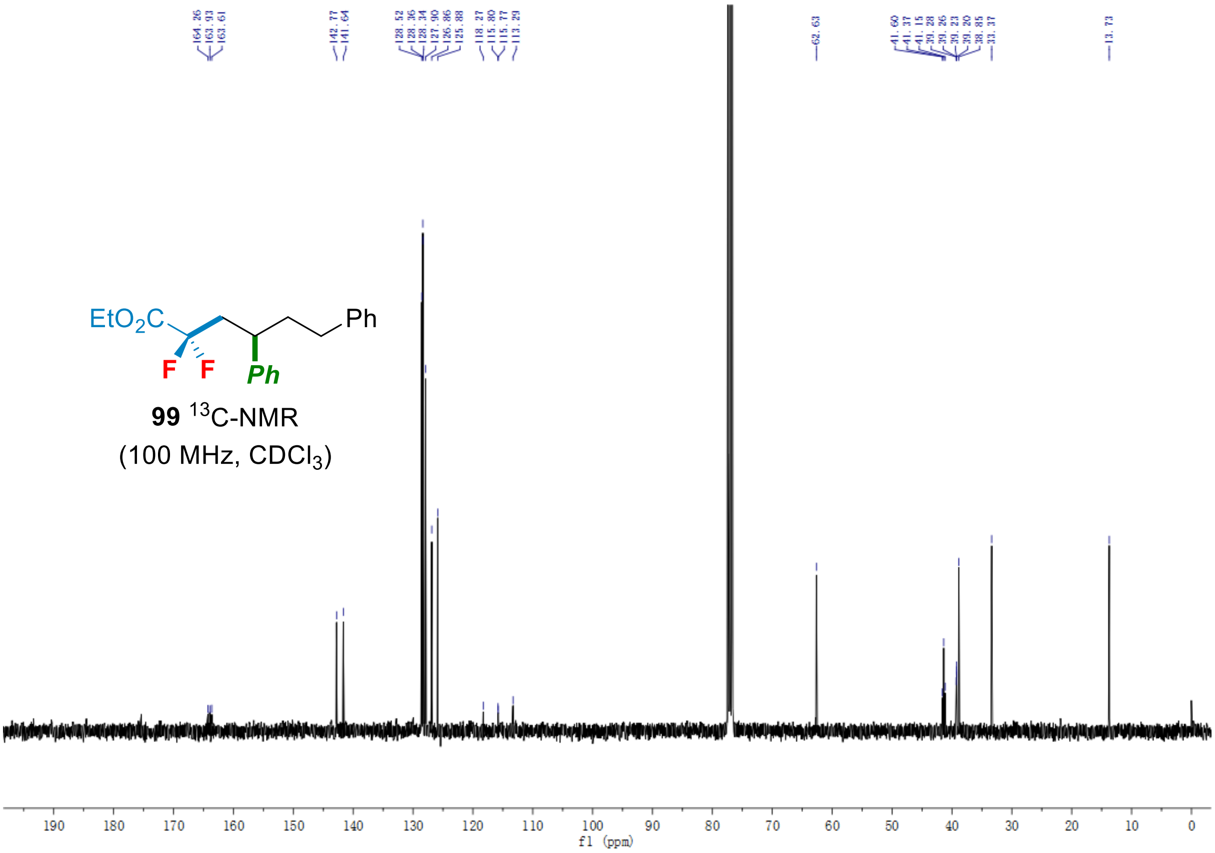
164.26
163.93
163.61
142.77
141.64
128.52
128.36
128.34
127.90
126.86
125.88
118.27
115.80
115.77
113.29
62.63
41.60
41.37
41.15
39.28
39.26
39.23
39.20
38.85
33.37
13.73
EtO2C
Ph
F
F
Ph
99 13C-NMR
(100 MHz, CDCl3)
190
180
170
160
150
140
130
120
110
100
90
80
70
60
50
40
30
20
10
0
f1 (ppm)

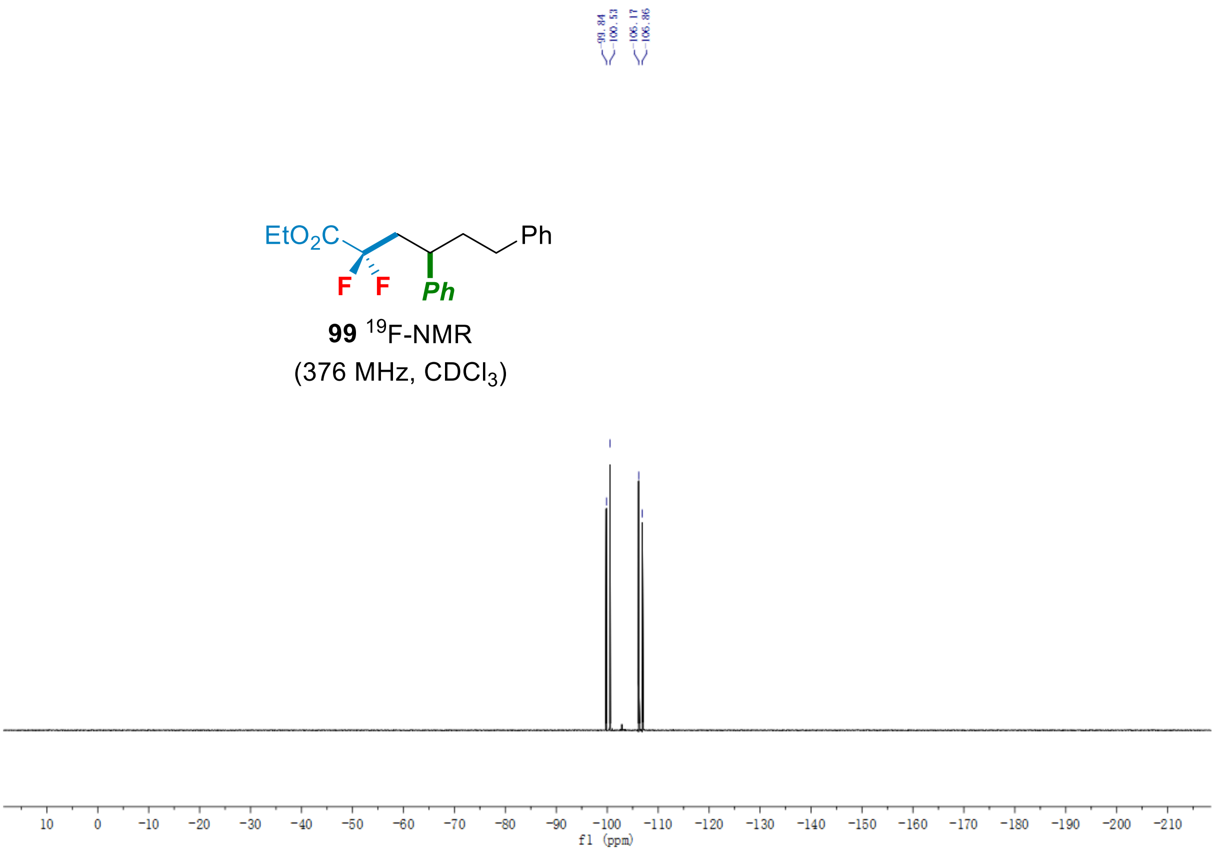
-99.84
-100.53
-106.17
-106.86
EtO2C
Ph
F
F
Ph
99 19F-NMR
(376 MHz, CDCl3)
10
0
-10
-20
-30
-40
-50
-60
-70
-80
-90
-100
-110
-120
-130
-140
-150
-160
-170
-180
-190
-200
-210
f1 (ppm)

Supplementary References:

[1] Yu, J.; Wu, Z.; Zhu, C. *Angew. Chem., Int. Ed.* **2018**, *57*, 17156-17160.

[2] (a) Han, S.; Liu, S.; Liu, L.; Ackermann, L.; Li, J. *Org. Lett.* **2019**, *21*, 5387-5391. (b) Da, Y.; Han, S.; Du, X.; Liu, S.; Liu, L.; Li, J. *Org. Lett.* **2018**, *20*, 5149-5152. (c) Tarui, A.; Shinohara, S.; Sato, K.; Omote, M. *Org. Lett.* **2016**, *18*, 1128.
